# An ultra-high-throughput screen for the evaluation of peptide HLA-Binder interactions

**DOI:** 10.1038/s41598-023-32384-z

**Published:** 2023-03-31

**Authors:** Stefan Krämer, Andreas Moritz, Luca Stehl, Meike Hutt, Martin Hofmann, Claudia Wagner, Sebastian Bunk, Dominik Maurer, Günter Roth, Johannes Wöhrle

**Affiliations:** 1 BioCopy GmbH, 79312 Emmendingen, Germany; 2 BioCopy AG, 4123 Basel, Switzerland; 3 Immatics Biotechnologies GmbH, 72076 Tübingen, Germany

**Keywords:** Immunotherapy, Drug safety, High-throughput screening, High-throughput screening, Sensors and probes

## Abstract

Peptide human leukocyte antigen (pHLA) targeting therapeutics like T-cell receptor based adoptive cell therapy or bispecific T cell engaging receptor molecules hold great promise for the treatment of cancer. Comprehensive pre-clinical screening of therapeutic candidates is important to ensure patient safety but is challenging because of the size of the potential off-target space. By combining stabilized peptide-receptive HLA molecules with microarray printing and screening, we have developed an ultra-high-throughput screening platform named ValidaTe that enables large scale evaluation of pHLA-binder interactions. We demonstrate its potential by measuring and analyzing over 30.000 binding curves for a high-affinity T cell Engaging Receptor towards a large pHLA library. Compared to a dataset obtained by conventional bio-layer interferometry measurements, we illustrate that a massively increased throughput (over 650 fold) is obtained by our microarray screening, paving the way for use in pre-clinical safety screening of pHLA-targeting drugs.

## Introduction

Recent developments in adoptive cell therapy (ACT)^1–3^ as well as in the field of bispecific T-cell engagers (BiTE)^4^ have shown that peptide-human leukocyte antigen (pHLA)-targeting therapies represent valid strategies to treat cancer^5^, with a first bispecific T cell receptor (TCR)-based therapy recently approved in uveal melanoma^6–9^. Peptide-HLA class I complexes are trimeric complexes that consist of a polymorphic heavy chain, the light chain beta-2 microglobulin ($\beta$2m) and a peptide ligand, typically between 8 and 10 amino acids long and derived from cellular proteins by degradation^10^. T cells can recognize specific peptide-HLA complexes with the TCR and initiate immune responses. Similarly, high-affinity TCR-based bispecific molecules or TCR-like antibodies bind to the respective pHLA target and trigger immune cell activity through a second binder, e.g. an anti-CD3 antibody that recruits T cells to the specific pHLA-expressing tumor cell. While these therapeutic approaches offer great potential, targeting pHLA molecules also bear the risk of off-target reactivities as experienced in some clinical trials^11–14^. Therefore, there is a clear need for comprehensive high-throughput off-target screenings for pHLA-targeting therapeutics, that can also be applied early in development to discriminate off-target profiles of different candidates. A great deal of energy has been invested in the development of soluble forms of the pHLA-reactive moiety of pHLA specific therapeutics, such as soluble T-cell receptors or TCR-like antibodies^15–17^, in order to enable interaction measurements at the molecular level. Directly determining the kinetic parameters of TCR-pHLA interactions can be a valuable addition to complex and laborious cellular screening approaches that usually require less direct readouts like cytokine expression detection. Label-free measurement methods, such as surface plasmon resonance (SPR)^18^, biolayer interference (BLI)^19^ or reflectometric interferometry spectroscopy (RIfS)^20,21^ offer a convenient way to measure the kinetic parameters ($k_{ass}$, $k_{diss}$ and $K_D$). Grating–coupled interferometry (GCI) even demonstrated the possibility of determining the binding kinetics of ions to biomolecules^22^. Consequently, these techniques have become a standard tool in the early phase of the development of novel therapeutics in recent years. However, the immunopeptidome is estimated to contain at least 150.000 different pHLA complexes for the class I HLA molecules alone^23^. This illustrates the strong need and potential for a new ultra-high throughput approach to characterize as many interactions in parallel as possible. Currently, state of the art technologies can only realize such a high-throughput to a limited extend. Moritz et. al.^24^ were able to remove the obstacle of large scale high quality pHLA library generation for kinetic screening purposes by introducing disulfide-stabilized HLA molecules. These empty HLA complexes make it possible to bypass the time-consuming step of refolding every individual pHLA complex with simple peptide loading reactions. Nevertheless, their measurements were limited to 16 different pHLA-analyte interactions in parallel using a BLI system. This throughput could at best be increased to 96 interactions in parallel on a different BLI machine with lower polling rates. Array-based SPR systems such as IBIS-MX96 have also been used successfully, but are limited to 96 parallel measurements, which is still far from a true ultra-high throughput approach^25^. Since microarrays were originally developed for high-throughput analysis, they are an excellent tool to determine interactions quickly and cost-efficiently^26,27^. Yet, standard microarrays also face some challenges. On the one hand, kinetic data is typically not easy to evaluate using microarrays^28–30^. On the other hand, in the case of peptide-HLA complexes, the peptide exchange must be performed beforehand in a microtiter plate format, which often consumes a lot of material (in this study, the microarray production method consumed over 1,000 times less material). Additionally, with increased numbers of different pHLA molecules and spots, the spotting duration also increases and molecule stability becomes a challenge particularly with comparatively unstable complexes like pHLAs. Furthermore, the storage of microarrays after their production and ensuring the functionality of the molecular complexes is an additional challenge that should not be underestimated.

In this study, we present a new method for the on demand generation of high-density pHLA microarrays on special substrates using microfluidic chips that solves many of the previously outlined challenges^31^. In a first step, the pHLA complex arrays are generated fresh and on demand using disulfide-stabilized HLA molecules and a novel loading and sandwich spotting technique using Polydimethylsiloxan (PDMS) cavity chips. In a second step, the high density pHLA microarrays are utilized in an ultra-high-throughput binding study using single color reflectometry (highSCORE)^32^. With this workflow, we were able to analyze the binding of a model TCER to an exemplary pHLA library of 127 different peptides, generating over 5,000 spots per array and binding curves per measurement. Comparing this data set to a smaller scale dataset obtained on a BLI platform, we found great correlation between both measurements, validating the accuracy of our new method.

## Results and discussion

### Next generation pHLA microarray generation

The generation of pHLA complexes and high-density pHLA microarrays in particular can be a difficult task. Ideally, every single spot consists of a distinct peptide-HLA complex combination, is spatially separated from its neighboring spots and shows a good homogeneity. While MHC class 1 HLA-A*02:01 molecules as used in this study are stable when cognate peptides are bound in the peptide binding groove formed by the $\alpha$1 and $\alpha$2 subunit of the heavy chain, the complex degrades quickly when the peptide dissociates. The rate of dissociation and complex degradation can be quick even for relevant immunogenic peptides, with some complexes displaying half-lives below 2 hours^33^. Therefore, the measurement of the pHLA microarrays should ideally take place as soon as possible after opening the cavity chip. Although disulfide-stabilized HLA molecules improve the peptide exchange reaction speed as well as the stability of the complex, large microarray printing or storage duration in unfavorable conditions must be avoided to preserve the integrity of the molecules on the surface. This can be difficult when microarrays with thousands of spots are produced using classic microarray printers. They can take a long time to print a large number of different spots from microtiter plates. Additionally, the deposition of tiny amounts of volumes on the surface can cause drying effects of the spots which may harm the pHLA complexes. Another downside of classical microarray printing is the fact that each pHLA complex needs to be prepared and exchanged in a rather high volume within a microtiter plate format. Yet, only a fraction of this volume is used for the actual print. Consequently, such an approach is only economical when many arrays are generated simultaneously, which in turn further increases the printing duration and in addition raises the challenge of storing the finished microarrays.

**Figure 1:**
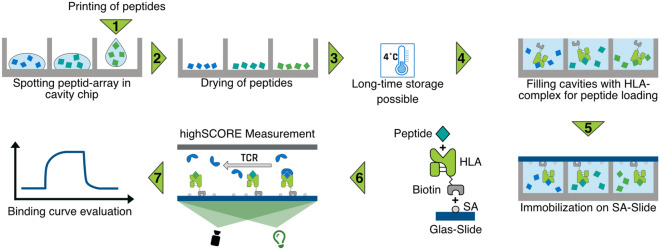
Different peptides are spotted into unique cavities of a microcavity chip (1). Peptide solution is dried out (2) and the arrays are ready for long time storage (3). To form the biomolecular complexes, biotinylated HLA is printed into the individual cavities and the complex formation takes place (4). The complexes are then transferred to a streptavidin (SA) coated substrate (5). The substrate can then be used for the label-free analysis of different analytes (6) and the kinetic values can be determined from the binding curves (7).

To overcome these drawbacks, we have developed a completely new technique for on demand production of pHLA microarrays (Fig. 1). By separating the printing of the different peptides from the pHLA complex generation, much of the time-consuming printing process can be done in advance while the microarray itself is generated very quickly immediately before an actual measurement without introducing time discrepancy between spots. This is made possible by using cavity chips and peptide-receptive HLA molecules: In a first step, individual peptides are printed into single cavities of a cavity chip. The cavity chip itself is made out of silicone (PDMS)^31^ and consists of 5,226 cavities within a 1.4 x 1.4 cm square. Each cavity has a very small volume of about 500 pL, a diameter of 150 $\upmu$m and a spacing of 50 $\upmu$m. After the printing, the peptides are dried and the peptide chips can be stored at 4$^{\circ }$C until further usage. Prior to measurement, the cavities of the peptide chips are filled with biotinylated peptide-receptive HLA molecules. This process needs to be performed with care. It is important to fill the cavities with the exact amount of volume. An overfilling will result in a cross contamination of the different cavities and an underfilling will result in a bad immobilization of the molecules on the surface. After the HLA filling, the cavity chip is closed with a streptavidin-coated glass slide in order to capture the pHLA complexes. Afterwards, the slide is removed from the chip, washed and blocked and can be used directly for a subsequent experiment.

### Ultra high troughput pHLA binding measurement

**Figure 2:**
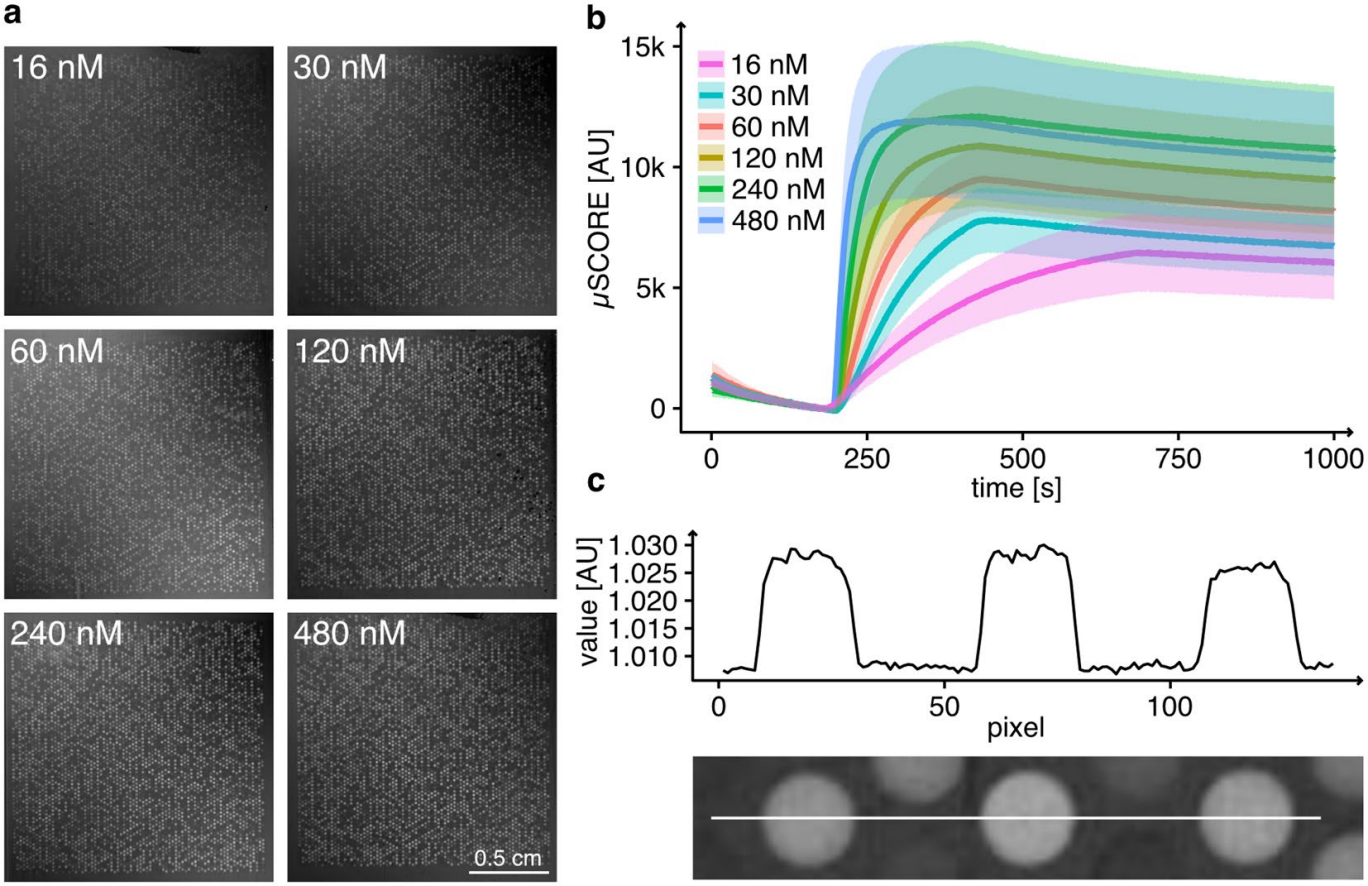
(**a**) highSCORE images illustrating the total binding of TCR against the pHLA spots of the microarray for different concentrations of analyte. The brighter the spots are, the more TCER molecules bound to the spots. Every image originates from an individual highSCORE measurement. A small area of crosscontamination is visible in the upper left corner of 240 nM image (at the image label). Small areas like this are not critical for the evaluation of the bindng kinetic values since a high number of replicates (n=26) was randomly distributed over the array for each peptide-HLA combination.(**b**) highSCORE binding curves of the TCER binding to its WT peptide (SLLQHLIGL). The curves are illustrated as mean curves with sd error bands (n=26 per binding curve). The association time was increased for the 16 nM concentration. (**c**) Line scan over 3 spots of the 480 nM microarray. The graph illustrates the gray pixel values of the white line in the image below.

While the generation of complex pHLA microarrays represents an achievement in itself, they are only of value for pHLA-analyte interaction screenings with a similarly high throughput measurement platform. The highSCORE technology offers the possibility to measure thousands of binding interactions in high throughput^32,34,35^. We used this technology for an exemplary analysis of the model TCER IMAHiAff#1, a soluble high-affinity T-cell receptor directed against a peptide derived from the cancer target PRAME^36^. By using a positional scanning approach with single amino acid mutational variants of the WT excluding the HLA anchor positions, we can generate a diverse library most likely covering a wide range of different binding affinities between TCER and resulting pHLA complexes. In total, 6 identical pHLA microarrays were produced as described in the section above and each microarray was measured with a distinct concentration of analyte (16 nM to 480 nM) in a highSCORE instrument. Every microarray contains 26 replicates of each peptide-HLA combination and controls which resulted in 5226 binding curves per array and 31.356 binding curves overall. All binding curves were fitted with a 1:1 binding model and the kinetic parameters were calculated ($k_{ass}$, $k_{diss}$, $K_D$). Since highSCORE is an image based spectroscopic technique, it is possible to illustrate the total binding signal of the TCR against the different pHLA spots for all measured microarrays (Fig. 2a). The binding affinity against the WT target peptide sequence resulted in a $K_D$ value of 1.27E-8M (Fig. 2b). Overall, the $K_D$ values against the different peptide variants ranged from 8.98E-9 M to 1.01E-6 M with a median of 1.36E-8 M. No $K_D$ value could be determined for 11 peptide variants because no binding was observed. A table of all kinetic values against all pHLA variants (Supplementary Data S1) is provided in the supplement. The produced microarrays showed no cross contamination between the different microcavities (Fig. 2c). All spots show sharp edges with signals that very quickly reach background level again and the general spot homogeneity is good. We did not observe dry in effects which are typically observed on spotted microarray approaches.

### Evaluation of the peptide target specificity

**Figure 3:**
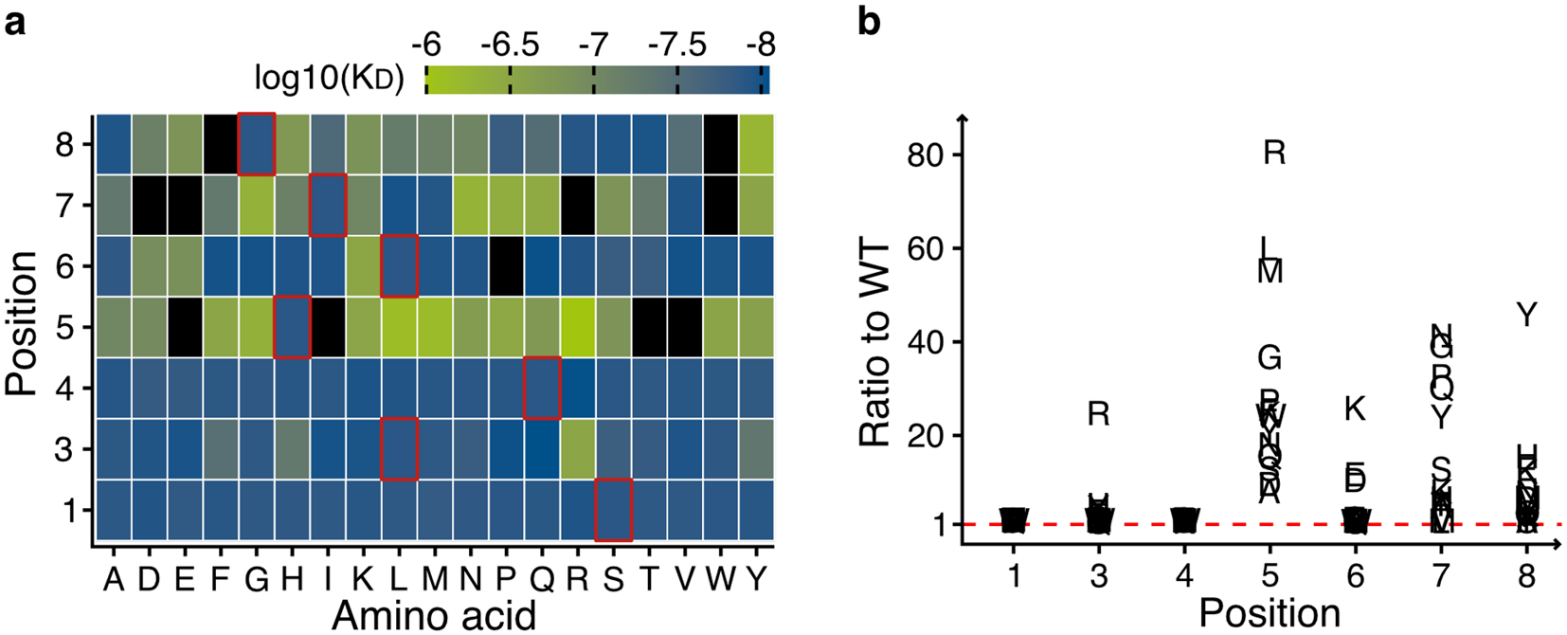
(**a**) Heat map of the $K_D$ values obtained from the positional scanning peptide library of the WT peptide (SLLQHLIGL). High $K_D$ are illustrated in green and lower $K_D$ values are illustrated in blue. Positions that are identical to the WT peptide sequence are highlighted with red boarders. Black tiles mean that no $K_D$ value could be determined. (**b**) Sequence plot of the $K_D$ values from the scanning peptide library. All $K_D$ values in both figures are illustrated as weighted means across all replicates. The number of replicates as well as the exact $K_D$ values for each peptide is provided in the Supplementary Data S1.

The dataset gathered with the highSCORE experiments and such a peptide library scan can also be used to derive information about the pHLA binding specificity of the model TCER molecule (Fig. 3). Based on this profile, mutations at position 5 lead to a strong increase in the $K_D$ value and in 4 of 19 cases, no $K_D$ value could be determined. This suggests that position 5 appears to be important for the TCR binding. This effect of increased $K_D$ value can also be observed for positions 7 and 8, although not as strongly as in the case of position 5. Position 3 shows a strong $K_D$ increase for R and position 6 a strong increase for K, E and D. Overall, the data obtained suggest, that position 5, 7 and 8 seem to be the most relevant for the binding of the TCR molecule, which is in line with the described binding mechanism of many TCRs.

### Correlation of highSCORE and BLI measurements

**Figure 4:**
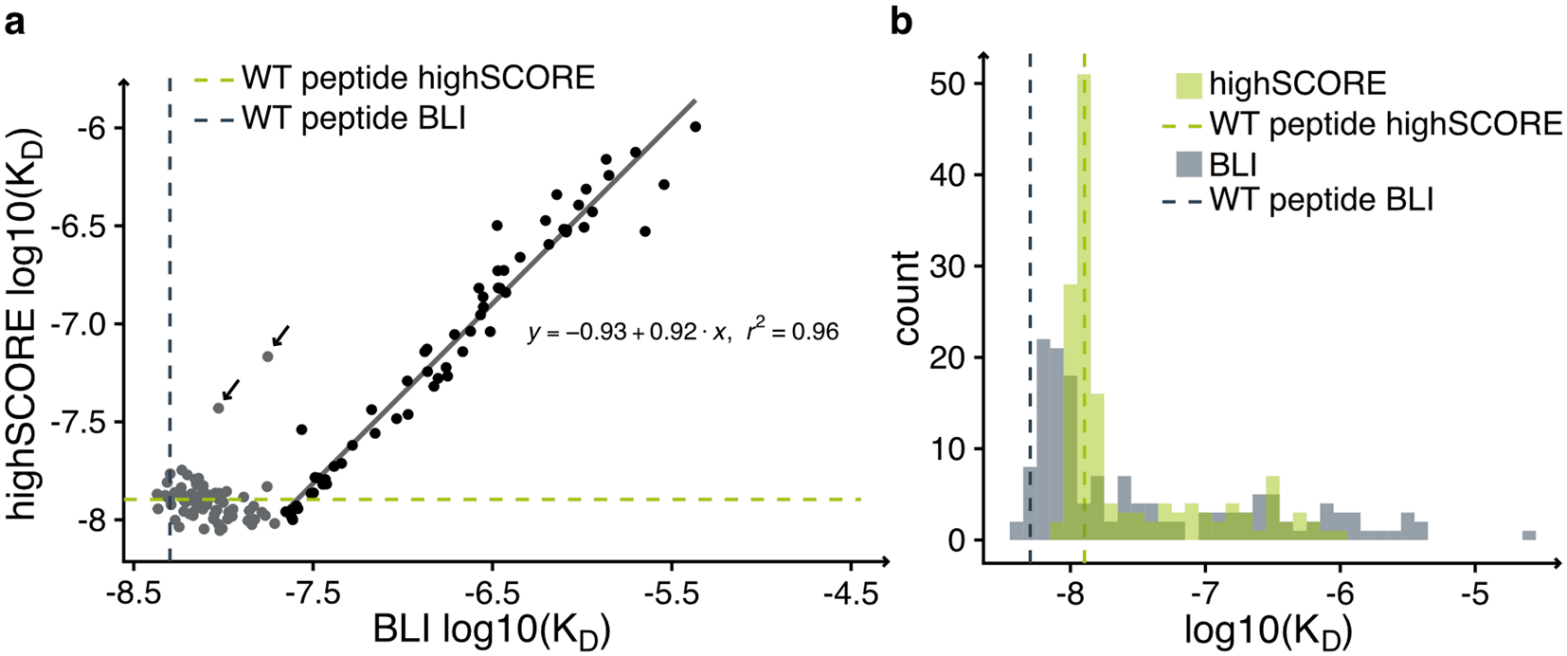
(**a**) Correlation between $K_D$ values obtained from highSCORE and BLI measurements. The BLI WT $K_D$ value is illustrated as blue dotted line and highSCORE WT $K_D$ as green dotted line. Arrows highlight points that stand out. Points colored in gray were excluded from the linear fit. (**b**) Histogram of the $log10(K_D)$ values for highSCORE (green) and BLI (blue) measurements. The BLI WT $K_D$ value is illustrated as blue dotted line and highSCORE WT $K_D$ as green dotted line.

In order to confirm our results obtained from the ultra high throughput highSCORE measurements, we compared the $K_D$ values with the corresponding ones from BLI measurements (Fig. 4). The direct correlation between the two methods can be described as a hockey stick. Many peptides with single amino acid mutations show similar $K_D$ values as the WT peptide. This is especially the case for $K_D$ values obtained from the highSCORE measurements. Within the BLI data set, most of the library peptides show a higher $K_D$ value compared to the corresponding WT peptide (Fig. 4a). This relation results in a flat region within the correlation plot. All other peptide, with the exception of the ones highlighted with an arrow, show a good linear correlation between both data sets. It is important to note that while the spectroscopic principle behind BLI and highSCORE is similar, the experimental procedure is different. Using BLI, the pHLA complexes are bound to a sensor tip, which is dipped into a microtiter plate well containing the analyte. Thereafter, the sensor tip is wiggled within the well to ensure that the analyte is not depleted on the surface. In contrast, during a highSCORE measurement, the analyte is flushed over the microarray using a thin flow chamber. This makes the experimental procedure of a highSCORE measurement more similar to that of an SPR device. Consequently, differences in the $K_D$ values between BLI and highSCORE measurements are to be expected and might explain the shift in the overall distribution of the $K_D$ values (Fig. 4b). According to the BLI method, the WT peptide has one of the lowest $K_D$ values and mutations in the amino acid sequence often lead to an increase in the $K_D$ value. In contrast, our hihghSCORE data suggests that some mutations of the WT peptide did not cause a noticeable difference in the binding of the TCR in the highSCORE measurements. Since both measurement methods have their own advantages and disadvantages, it is difficult to tell which of the two methods provides the values that are closest to reality.

### Conclution and outlook

We were able to demonstrate that a microarray-based approach can be used for the high-throughput characterization of pHLA-TCR interactions and thus off-target profiling. The current set-up allows scale up to 500 or more distinct pHLA complexes to be screened for, and 1000 or more are envisioned with a slightly improved cavity chip design in the future. Hence, using our technology, it is possible to screen a much larger number of pHLA complexes than with conventional methods, being of great value for in-depth preclinical safety profile assessments of pHLA binding therapeutic molecules such as TCR bispecifics or pHLA-specific antibodies. In the future, we will also investigate whether it is feasible to determine the stability of the individual pHLA complexes. Moreover, we are exploring the possibilities of using the pHLA arrays for cellular assays as well.

## Methods

### Peptides

All peptides were generated in-house using standard 9-fluorenyl methoxycarbonyl chemistry with a Syro II peptide synthesizer. Peptides were subsequently analyzed using high-performance liquid chromatography (HPLC). Before use, peptides were dissolved in dimethyl sulfoxide (DMSO; catalog no. 41640, Sigma-Aldrich) and 0.5% trifluoroacetic acid (TFA; catalog no. T6508, Sigma-Aldrich).

### Disulfide-stabilized HLA complexes

Disulfide-stabilized HLA complexes were generated as previously described^24^. In short, HLA-A*02:01 heavy chain with disulfide-introducing mutations C-terminal BirA signal sequences or His6-Tag and human $\beta$2m light chain was produced in E. coli as inclusion bodies and purified. Heavy chain, $\beta$2m light chain, and GM dipeptide were diluted in refolding buffer [100 mM tris-Cl (pH 8), 0.5 M arginine, 2 mM EDTA, 0.5 mM oxidized glutathione, and 5 mM reduced glutathione] and incubated for 2 to 8 days at 4 $^{\circ }$C while stirring before concentration. The concentrated protein was purified by SEC in 20 mM tris-HCl (pH 8)/150 mM NaCl on an ÄKTAprime system (GE Healthcare) using a HiLoad 26/600 Superdex 200 pg column (GE Healthcare). Peak fraction was either concentrated directly to 2000 $\upmu$g/ml, aliquoted and frozen at -80$^{\circ }$C or biotinylated by BirA biotin protein ligase (Avidity) overnight at 4 $^{\circ }$C according to the manufacturer’s instructions, and subjected to a second gel filtration before final concentration to 2000 $\upmu$g/ml, aliquoting and storage at -80 $^{\circ }$C.

### Model TCER molecules

The soluble model TCER molecule IMAHiAff#1 was generated as previously described^36^.

### Production of peptide arrays

Polydimethlysiloxan (PDMS) cavity chips with a total of 5226-cavities (5k-PDMS chips) were fabricated by BioCopy GmbH. 5k-PDMS chips were produced as described by Wöhrle et al.^31^. PDMS Elastosil RT 601 A/B used to produce 5k-PDMS chips was purchased from Wacker, Germany. Peptide arrays were manufactured by INTER-ARRAY fzmb GmbH (Bad Langensalza, Germany) in 5k-PDMS chips. Peptides were spotted using a non-contact microarray printer (iOne, M2-Automation). Peptides were used at a concentration of 200 $\upmu$g/ml in a dimethylsulfoxid (DMSO, Carl Roth, Germany) solution (5% DMSO v/v, 95% water v/v). 300pl of peptide solution was spotted into each well of the 5k-PDMS chip. A total of 127 different peptides were spotted on the array in a random distribution and with 26 replicates per peptide per array. After spotting, the peptide solution was dried out at RT. Peptide arrays were stored at 4 $^{\circ }$C until further processing.

### Production of 3D-streptavidin highscore slides

3D-Streptavidin highSCORE slides were produced by BioCopy GmbH. highSCORE slides were washed with acetone (Carl Roth, Germany), isopropanol (VWR International, Germany) and water. The slides were then dried in an N_2_ stream. Subsequently, slides were plasma activated using a plasma generator (ZEPTO, Diener electronics) for 1 min at 0.3mbar gas and 100% generator power. Then, 80 $\upmu$l of (3-glydiyloxypropyl)trimethoxysilane (GOPTS, Sigma Aldrich, Germany) solution was pipetted onto a slide. A second slide was then placed on the slide containing GOPTS to form a sandwich. The sandwich was then incubated for 1h at RT. The slides were then separated and rinsed two times in anhydrous acetone (Merck, Germany) and dried using N_2_. Then, 80 $\upmu$l of aminodextran (AMD, Innovent, Germany) solution (14.3% AMD w/v, 85,7% water w/v) was pipetted onto a slide. Another slide was placed on top of the slide containing aminodextran. The sandwiches were then incubated overnight in a humid water chamber at RT. Subsequently, slides were separated from each other, rinsed twice with water and dried in N_2_ stream. Slides were then put into a p-phenylene diisothiocyanate (PDITC, Sigma-Aldrich, Germany) solution (10,4 mM PDITC in 90% DMF [v/v] and 10% pyridine [v/v]), followed by an incubation for 2h at RT. Subsequently, the slides were flushed with ethanol (Carl Roth, Germany) and washed two times in ethanol for 5 min, followed by acetone for 5 min. Slides were dried in a N_2_ stream and put under vacuum in a desiccator for 15 min. Then, 80 $\upmu$l of streptavidin (SA, Sigma Aldrich, Germany) solution (1mg/ml in PBS) was pipetted onto a slide. A second slide was then placed on the slide containing SA to form a sandwich. The sandwiches incubated at 4 $^{\circ }$C in a humid water chamber overnight. The slides were then separated and washed two times in water. Afterwards, the slides were dried with N_2_ stream. The finished 3D-SA highSCORE slides were stored under nitrogen atmosphere at 4 $^{\circ }$C.

### Production of peptide-HLA arrays

Peptide HLA arrays were produced by BioCopy GmbH. For this, 5k-PDMS chips containing the peptide arrays were warmed up to RT. Peptide arrays were then cleaned with an N_2_ gas stream to remove dust. Subsequently, 900 pl of HLA solution (200 $\upmu$g/mL HLA, 1M betaine (Sigma Aldrich, Germany), 0.07% BSA (Carl Roth, Germany), 0.035% Tween 20 (Sigma Aldrich, Germany) 1xPBS, pH 7,4 (Sigma Aldrich, Germany)) was spotted into each well of the peptide arrays using a non-contact microarray printer (iTWO, M2-Automation). The final HLA to peptide ration was 1/11. Spotting was carried out at 80% humidity to prevent evaporation of the HLA solution during the process. Next, 3D-SA highSCORE slides were placed on the 5k-PDMS chips containing the peptide-HLA mix, resulting in chip-highSCORE slide sandwiches. To transfer the newly formed peptide-HLA complexes from the cavities of the 5k-PDMS chips onto the highSCORE slides, slides were pressed onto the chips with a force of 165 N using custom-made presses. To completely immobilize the peptide-HLA complexes on the 3D-SA surface of the highSCORE slides via the biotinylated HLA, the sandwiches were incubated overnight at 4 $^{\circ }$C in the presses. highSCORE measurements of the peptide-HLA arrays were carried out on the following day.

### highSCORE-measurement of peptide-HLA arrays

Single color reflectometry (highSCORE) measurements of peptide-HLA arrays were carried out by BioCopy GmbH using the proprietary highSCORE devices. The highSCORE technology detects molecule-molecule interactions in a low-volume and label-free manner^32^. For this work, highSCORE was used to measure the interaction of a soluble TCR with peptide-HLA complexes immobilized on a 3D-SA coated highSCORE slide. Prior to measurement, the Chip-highSCORE slide sandwiches were opened in a biotin blocking solution (10 $\upmu$g/mL biotin (Carl Roth, Germany), 0.05% Tween 20 in 1xPBS, pH 7.4). After opening the sandwiches, highSCORE slides including the peptide-HLA arrays were washed with water and dried in an N_2_ gas stream. In total 6 peptide-HLA arrays were measured in 6 independent highSCORE measurements each with a different TCR concentration. For this, soluble TCR was diluted in running buffer (0.05% Tween 20, 1xPBS, pH 7.4) to concentrations of 16 nM, 30 nM, 60 nM, 120 nM, 240 nM and 480 nM, respectively. highSCORE measurements were performed in running buffer (0.05% Tween 20, 1xPBS, pH 7.4) and consisted of 180 s baseline, 250 s association, 300 s dissociation, and 300 s endline at a flow rate of 2 $\upmu$L/s each. For highSCORE measurement with 16 nM TCR, association was extended to 500 s and dissociation to 600 s at a flow rate of 2 $\upmu$L/s. All six highSCORE-measurements were conducted on the same day, using the same highSCORE-device. The data acquired during the SCORE measurement was analysed using BioCopy’s software calcSCORE V1.0 to obtain a distinct binding curve for each pHLA complex spot of the microarray. Subsequently, a 1:1 binding model, including a global exponential decay, was fitted to all individual binding curves and the resulting data was used for the calculation of the kinetic parameters. The binding curves were fitted individually to account for the differences in the total binding signals per array (free $R_{max}$). Thereafter, the kinetic values were determined via a $k_{obs}$-linearisation. The fitting and subsequent analysis was performed using the software package Anabel V3.0, which will be published soon as an online version and an R-package (named anabel). The Software is also available upon request. The analysis of the spot intensities as shown in Fig. 2c was performed using the “Plot profile” feature of ImageJ V2.1.0. The plots shown in Figs. 3 and 4 were produced in R from the data provided in Supplementary Data S1.

### BLI measurements

BLI measurements were performed on an Octet RED384 system using anti-Penta-His biosensors. In short, disulfide-stabilized molecules were refolded and peptide-loaded as previously described. All analytes or ligands were diluted to their final concentration in kinetics buffer (PBS, 0.1% BSA, 0.05% Tween-20) if not specified otherwise. All biosensors were hydrated for at least 10 minutes in kinetics buffer before use. Loadings and measurements were performed in 384 tilted well plates (Pall Fortébio) with at least 40 $\upmu$l at a 3 mm sensor offset. Plate temperature was set at 30 $^{\circ }$C and shaker speed at 1000 rpm. To generate a baseline, sensors were dipped in kinetics buffer for 60 s before being loaded with 10 ug/ml of peptide loaded disulfide-stabilized pHLA for 120 s. Afterwards, a second 120 s baseline was recorded before a 60 s association and a 240 s dissociation phase. After individual measurements, sensors were regenerated using 10 mM Glycine pH 1.5 4 times for 5s each before another cycle was performed. 4 TCER concentrations (500nM, 158.1nM, 50nM and 15.8nM) were measured for each pHLA complex. A sensor loaded with an irrelevant pHLA was measured in line for reference sensor subtraction. All sensorgrams were analyzed using the OctetRED software “Data Analysis HT” version 11.0.0.50 (Pall Fortébio). Raw sensor data was aligned at the Y axis by aligning the data to the end of the baseline step, and inter-step correction was used to align the start of the dissociation to the end of the association phase. Savitzky-Golay filtering was applied. Resulting sensorgrams were then fitted using a 1:1 Langmuir kinetics binding model. In case of lower affinity binding events, the lower two concentrations were excluded from the fitting.

## Supplementary Information

Supplementary Information 1.

Supplementary Information 2.

## Data Availability

The highSCORE datasets generated during and/or analyzed during the current study are available for download as supplement data.
