# Supplementary material for: An ultra-high-throughput screen for the evaluation of peptide HLA-Binder interactions
Source: Sci Rep. 2023 Mar 31;13:5290. doi: 10.1038/s41598-023-32384-z (PMC10066226; doi:10.1038/s41598-023-32384-z)
Supplement: Supplementary file 2 — Supplementary Information 2. [file 41598_2023_32384_MOESM2_ESM.zip › raw_data_package/K02027_480nM/K02027_480nM_binding_curve_plots.pdf]

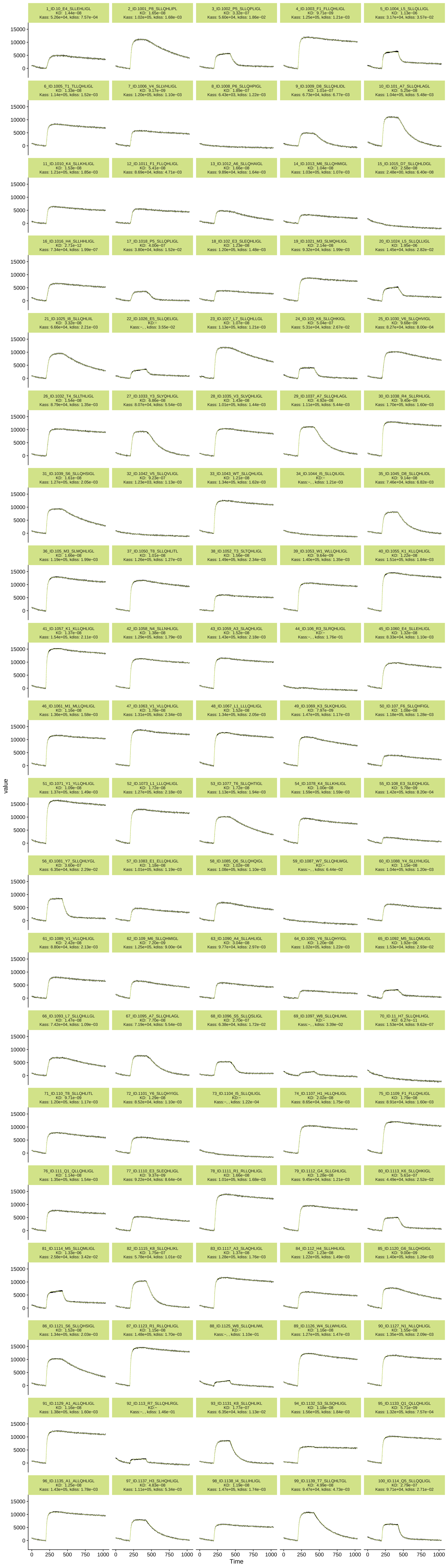

value
Time
0
250
500
750
1000
5000
10000
15000

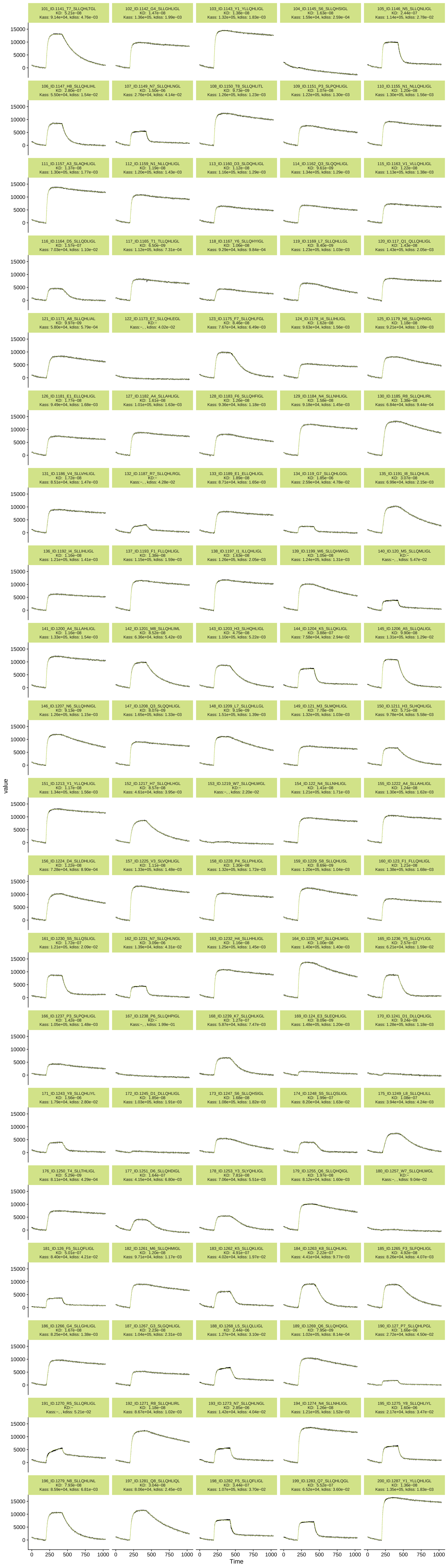

101_ID.1141_T7_SLLQHLTGL KD: 5.21e-08 Kass: 9.14e+04, kdiss: 4.76e-03
102_ID.1142_G4_SLLGHLIGL KD: 1.47e-08 Kass: 1.36e+05, kdiss: 1.99e-03
103_ID.1143_Y1_YLLQHLIGL KD: 1.38e-08 Kass: 1.32e+05, kdiss: 1.83e-03
104_ID.1145_S6_SLLQHSIGL KD: 1.63e-08 Kass: 1.59e+04, kdiss: 2.59e-04
105_ID.1146_N5_SLLQNLIGL KD: 2.44e-07 Kass: 1.14e+05, kdiss: 2.78e-02
106_ID.1147_H8_SLLQHLIHL KD: 2.80e-07 Kass: 5.50e+04, kdiss: 1.54e-02
107_ID.1149_N7_SLLQHLNGL KD: 1.50e-06 Kass: 2.76e+04, kdiss: 4.14e-02
108_ID.1150_T8_SLLQHLITL KD: 9.78e-09 Kass: 1.26e+05, kdiss: 1.23e-03
109_ID.1151_P3_SLPQHLIGL KD: 1.07e-08 Kass: 1.22e+05, kdiss: 1.30e-03
110_ID.1155_N1_NLLQHLIGL KD: 1.20e-08 Kass: 1.30e+05, kdiss: 1.56e-03
111_ID.1157_A3_SLAQHLIGL KD: 9.97e-09 Kass: 1.30e+05, kdiss: 1.77e-03
112_ID.1159_N1_NLLQHLIGL KD: 1.19e-08 Kass: 1.20e+05, kdiss: 1.43e-03
113_ID.1160_D3_SLDQHLIGL KD: 1.12e-08 Kass: 1.16e+05, kdiss: 1.29e-03
114_ID.1162_Q3_SLQQHLIGL KD: 9.61e-09 Kass: 1.34e+05, kdiss: 1.29e-03
115_ID.1163_V1_VLLQHLIGL KD: 1.22e-08 Kass: 1.13e+05, kdiss: 1.38e-03
116_ID.1164_D5_SLLQDLIGL KD: 1.57e-08 Kass: 7.03e+04, kdiss: 1.10e-02
117_ID.1165_T1_TLLQHLIGL KD: 6.52e-09 Kass: 1.12e+05, kdiss: 7.31e-04
118_ID.1167_Y6_SLLQHYIGL KD: 1.06e-08 Kass: 9.29e+04, kdiss: 9.84e-04
119_ID.1169_L7_SLLQHLLGL KD: 8.40e-09 Kass: 1.23e+05, kdiss: 1.03e-03
120_ID.117_Q1_QLLQHLIGL KD: 1.43e-08 Kass: 1.43e+05, kdiss: 2.05e-03
121_ID.1171_A8_SLLQHLIAL KD: 9.97e-09 Kass: 5.80e+04, kdiss: 5.79e-04
122_ID.1173_E7_SLLQHLEGL KD:~ Kass:~, kdiss: 4.02e-02
123_ID.1175_F7_SLLQHLFGL KD: 8.46e-08 Kass: 7.67e+04, kdiss: 6.49e-03
124_ID.1178_I4_SLLIHLIGL KD: 1.62e-08 Kass: 9.63e+04, kdiss: 1.56e-03
125_ID.1179_N6_SLLQHNIGL KD: 1.18e-08 Kass: 9.21e+04, kdiss: 1.09e-03
126_ID.1181_E1_ELLQHLIGL KD: 1.77e-08 Kass: 9.49e+04, kdiss: 1.68e-03
127_ID.1182_A4_SLLAHLIGL KD: 1.61e-08 Kass: 1.01e+05, kdiss: 1.63e-03
128_ID.1183_F6_SLLQHFIGL KD: 1.26e-08 Kass: 9.36e+04, kdiss: 1.18e-03
129_ID.1184_N4_SLLNHLIGL KD: 1.58e-08 Kass: 9.18e+04, kdiss: 1.45e-03
130_ID.1185_R8_SLLQHLIRL KD: 1.38e-08 Kass: 6.84e+04, kdiss: 9.44e-04
131_ID.1186_V4_SLLVHLIGL KD: 1.72e-08 Kass: 8.51e+04, kdiss: 1.47e-03
132_ID.1187_R7_SLLQHLRGL KD:~ Kass:~, kdiss: 4.28e-02
133_ID.1189_E1_ELLQHLIGL KD: 1.89e-08 Kass: 8.71e+04, kdiss: 1.65e-03
134_ID.119_G7_SLLQHLGGL KD: 1.85e-06 Kass: 2.59e+04, kdiss: 4.78e-02
135_ID.1191_I8_SLLQHLIIL KD: 3.07e-08 Kass: 6.99e+04, kdiss: 2.15e-03
136_ID.1192_I4_SLLIHLIGL KD: 1.16e-08 Kass: 1.21e+05, kdiss: 1.41e-03
137_ID.1193_F1_FLLQHLIGL KD: 1.38e-08 Kass: 1.15e+05, kdiss: 1.59e-03
138_ID.1197_I1_ILLQHLIGL KD: 1.63e-08 Kass: 1.26e+05, kdiss: 2.05e-03
139_ID.1199_W6_SLLQHWIGL KD: 1.05e-08 Kass: 1.24e+05, kdiss: 1.31e-03
140_ID.120_M5_SLLQMLIGL KD:~ Kass:~, kdiss: 5.47e-02
141_ID.1200_A4_SLLAHLIGL KD: 1.16e-08 Kass: 1.33e+05, kdiss: 1.54e-03
142_ID.1201_M8_SLLQHLIML KD: 8.52e-08 Kass: 6.36e+04, kdiss: 5.42e-03
143_ID.1203_H3_SLHQHLIGL KD: 4.75e-08 Kass: 1.10e+05, kdiss: 5.22e-03
144_ID.1204_K5_SLLQKLIGL KD: 3.88e-07 Kass: 7.58e+04, kdiss: 2.94e-02
145_ID.1206_A5_SLLQALIGL KD: 9.90e-08 Kass: 1.31e+05, kdiss: 1.29e-02
146_ID.1207_N6_SLLQHNIGL KD: 9.13e-09 Kass: 1.26e+05, kdiss: 1.15e-03
147_ID.1208_Q3_SLQQHLIGL KD: 8.07e-09 Kass: 1.65e+05, kdiss: 1.33e-03
148_ID.1209_L7_SLLQHLLGL KD: 9.19e-09 Kass: 1.51e+05, kdiss: 1.39e-03
149_ID.121_M3_SLMQHLIGL KD: 7.78e-09 Kass: 1.32e+05, kdiss: 1.03e-03
150_ID.1211_H3_SLHQHLIGL KD: 5.71e-08 Kass: 9.78e+04, kdiss: 5.58e-03
151_ID.1213_Y1_YLLQHLIGL KD: 1.17e-08 Kass: 1.34e+05, kdiss: 1.56e-03
152_ID.1217_H7_SLLQHLHGL KD: 8.57e-08 Kass: 4.61e+04, kdiss: 3.95e-03
153_ID.1219_W7_SLLQHLWGL KD:~ Kass:~, kdiss: 2.20e-02
154_ID.122_N4_SLLNHLIGL KD: 1.41e-08 Kass: 1.21e+05, kdiss: 1.71e-03
155_ID.1222_A4_SLLAHLIGL KD: 1.24e-08 Kass: 1.30e+05, kdiss: 1.62e-03
156_ID.1224_D4_SLLDHLIGL KD: 1.22e-08 Kass: 7.28e+04, kdiss: 8.90e-04
157_ID.1225_V3_SLVQHLIGL KD: 1.11e-08 Kass: 1.33e+05, kdiss: 1.48e-03
158_ID.1228_P4_SLLPHLIGL KD: 1.30e-08 Kass: 1.32e+05, kdiss: 1.72e-03
159_ID.1229_S8_SLLQHLISL KD: 8.69e-09 Kass: 1.20e+05, kdiss: 1.04e-03
160_ID.123_F1_FLLQHLIGL KD: 1.21e-08 Kass: 1.38e+05, kdiss: 1.68e-03
161_ID.1230_S5_SLLQSLIGL KD: 1.72e-07 Kass: 1.21e+05, kdiss: 2.09e-02
162_ID.1231_N7_SLLQHLNGL KD: 3.09e-07 Kass: 1.39e+04, kdiss: 4.31e-02
163_ID.1232_H4_SLLHHLIGL KD: 1.16e-08 Kass: 1.25e+05, kdiss: 1.45e-03
164_ID.1235_M7_SLLQHLMGL KD: 1.00e-08 Kass: 1.40e+05, kdiss: 1.40e-03
165_ID.1236_Y5_SLLQYLIGL KD: 2.57e-07 Kass: 6.21e+04, kdiss: 1.59e-02
166_ID.1237_P3_SLPQHLIGL KD: 1.42e-08 Kass: 1.05e+05, kdiss: 1.48e-03
167_ID.1238_P6_SLLQHPIGL KD:~ Kass:~, kdiss: 1.99e-01
168_ID.1239_K7_SLLQHLKGL KD: 1.27e-07 Kass: 5.87e+04, kdiss: 7.47e-03
169_ID.124_E3_SLEQHLIGL KD: 8.09e-09 Kass: 1.48e+05, kdiss: 1.20e-03
170_ID.1241_D1_DLLQHLIGL KD: 9.24e-09 Kass: 1.28e+05, kdiss: 1.18e-03
171_ID.1243_Y8_SLLQHLIYL KD: 1.56e-07 Kass: 1.79e+04, kdiss: 2.80e-02
172_ID.1245_D1_DLLQHLIGL KD: 1.85e-08 Kass: 1.03e+05, kdiss: 1.91e-03
173_ID.1247_S6_SLLQHSIGL KD: 1.68e-08 Kass: 1.08e+05, kdiss: 1.82e-03
174_ID.1248_S5_SLLQSLIGL KD: 1.99e-07 Kass: 8.20e+04, kdiss: 1.63e-02
175_ID.1249_L8_SLLQHLILL KD: 1.08e-07 Kass: 3.94e+04, kdiss: 4.24e-03
176_ID.1250_T4_SLLTHLIGL KD: 5.29e-09 Kass: 8.11e+04, kdiss: 4.29e-04
177_ID.1251_D6_SLLQHDIGL KD: 1.64e-07 Kass: 4.15e+04, kdiss: 6.80e-03
178_ID.1253_Y3_SLYQHLIGL KD: 7.81e-08 Kass: 7.06e+04, kdiss: 5.51e-03
179_ID.1255_Q6_SLLQHQIGL KD: 1.97e-08 Kass: 8.12e+04, kdiss: 1.60e-03
180_ID.1257_W7_SLLQHLWGL KD:~ Kass:~, kdiss: 9.04e-02
181_ID.126_F5_SLLQFLIGL KD: 5.01e-07 Kass: 8.40e+04, kdiss: 4.21e-02
182_ID.1261_M6_SLLQHMIGL KD: 1.20e-08 Kass: 9.71e+04, kdiss: 1.17e-03
183_ID.1262_K5_SLLQKLIGL KD: 4.91e-07 Kass: 4.02e+04, kdiss: 1.97e-02
184_ID.1263_K8_SLLQHLIKL KD: 2.22e-07 Kass: 4.41e+04, kdiss: 9.77e-03
185_ID.1265_F3_SLFQHLIGL KD: 4.92e-08 Kass: 8.26e+04, kdiss: 4.07e-03
186_ID.1266_G4_SLLGHLIGL KD: 1.67e-08 Kass: 8.25e+04, kdiss: 1.38e-03
187_ID.1267_G3_SLGQHLIGL KD: 2.23e-08 Kass: 1.04e+05, kdiss: 2.31e-03
188_ID.1268_L5_SLLQLLIGL KD: 2.44e-06 Kass: 1.27e+04, kdiss: 3.10e-02
189_ID.1269_Q6_SLLQHQIGL KD: 7.95e-09 Kass: 1.02e+05, kdiss: 8.14e-04
190_ID.127_P7_SLLQHLPGL KD: 1.66e-06 Kass: 2.72e+04, kdiss: 4.50e-02
191_ID.1270_R5_SLLQRLIGL KD:~ Kass:~, kdiss: 5.21e-02
192_ID.1271_R8_SLLQHLIRL KD: 1.18e-08 Kass: 8.67e+04, kdiss: 1.02e-03
193_ID.1273_N7_SLLQHLNGL KD: 2.85e-06 Kass: 1.42e+04, kdiss: 4.04e-02
194_ID.1274_N4_SLLNHLIGL KD: 1.26e-08 Kass: 1.21e+05, kdiss: 1.52e-03
195_ID.1275_Y8_SLLQHLIYL KD: 1.60e-06 Kass: 2.17e+04, kdiss: 3.47e-02
196_ID.1279_N8_SLLQHLINL KD: 7.93e-08 Kass: 8.59e+04, kdiss: 6.81e-03
197_ID.1281_Q8_SLLQHLIQL KD: 3.04e-08 Kass: 8.06e+04, kdiss: 2.45e-03
198_ID.1282_F5_SLLQFLIGL KD: 3.44e-07 Kass: 1.07e+05, kdiss: 3.70e-02
199_ID.1283_Q7_SLLQHLQGL KD: 5.52e-07 Kass: 6.52e+04, kdiss: 3.60e-02
200_ID.1287_Y1_YLLQHLIGL KD: 1.36e-08 Kass: 1.35e+05, kdiss: 1.83e-03
value
15000
10000
5000
0
0
250
500
750
1000
Time

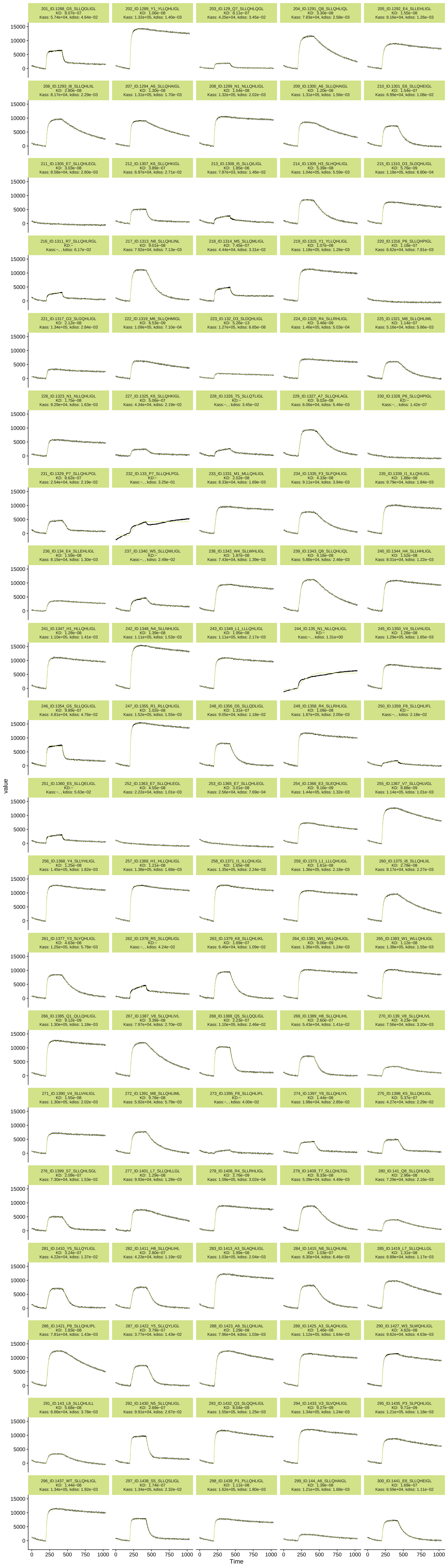
value
Time

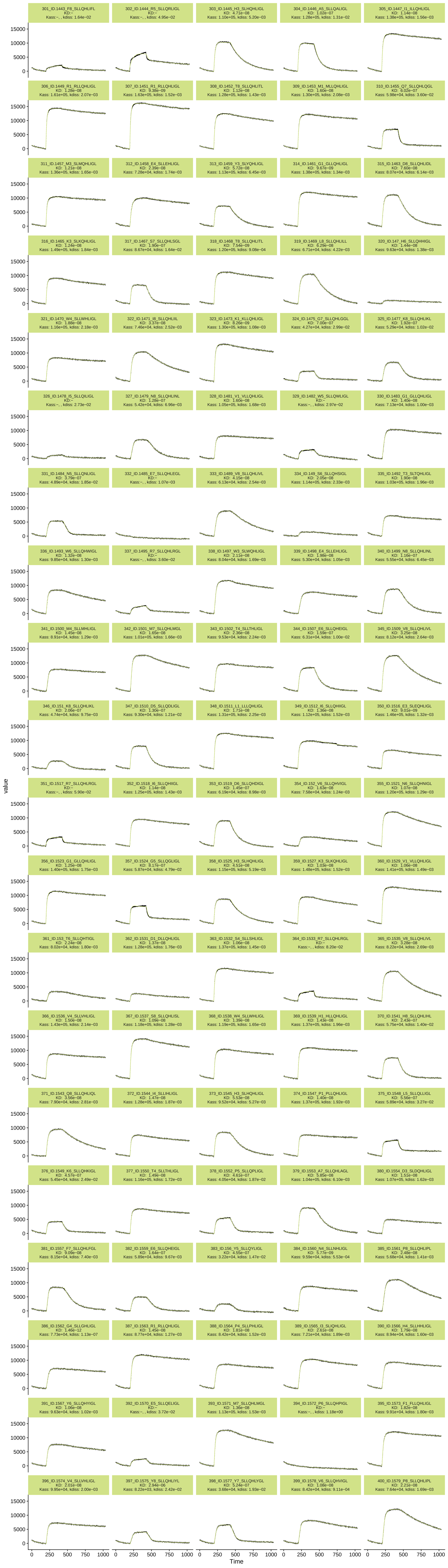

value
Time
0
250
500
750
1000
5000
10000
15000

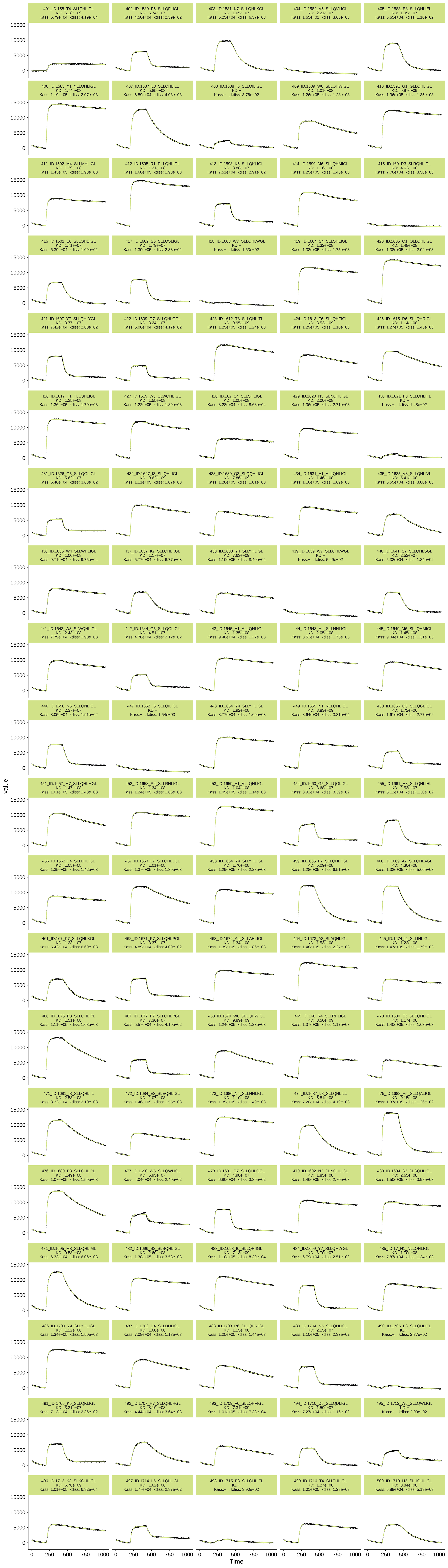
401_ID.158_T4_SLLTHLIGL KD: 6.18e−09 Kass: 6.79e+04, kdiss: 4.19e−04
402_ID.1580_F5_SLLQFLIGL KD: 5.74e−07 Kass: 4.50e+04, kdiss: 2.59e−02
403_ID.1581_K7_SLLQHLKGL KD: 1.05e−07 Kass: 6.25e+04, kdiss: 6.57e−03
404_ID.1582_V5_SLLQVLIGL KD: 2.21e−07 Kass: 1.65e−01, kdiss: 3.65e−08
405_ID.1583_E8_SLLQHLIEL KD: 1.95e−07 Kass: 5.65e+04, kdiss: 1.10e−02
406_ID.1585_Y1_YLLQHLIGL KD: 1.74e−08 Kass: 1.19e+05, kdiss: 2.07e−03
407_ID.1587_L8_SLLQHLILL KD: 5.85e−08 Kass: 6.89e+04, kdiss: 4.03e−03
408_ID.1588_I5_SLLQILIGL KD:~ Kass:~, , kdiss: 3.76e−02
409_ID.1589_W6_SLLQHWIGL KD: 1.01e−08 Kass: 1.26e+05, kdiss: 1.28e−03
410_ID.1591_G1_GLLQHLIGL KD: 9.97e−09 Kass: 1.36e+05, kdiss: 1.35e−03
411_ID.1592_M4_SLLMHLIGL KD: 1.39e−08 Kass: 1.43e+05, kdiss: 1.98e−03
412_ID.1595_R1_RLLQHLIGL KD: 1.21e−08 Kass: 1.60e+05, kdiss: 1.93e−03
413_ID.1598_K5_SLLQKLIGL KD: 3.88e−07 Kass: 7.51e+04, kdiss: 2.91e−02
414_ID.1599_M6_SLLQHMIGL KD: 1.16e−08 Kass: 1.25e+05, kdiss: 1.45e−03
415_ID.160_R3_SLRQHLIGL KD: 4.62e−08 Kass: 7.76e+04, kdiss: 3.58e−03
416_ID.1601_E6_SLLQHEIGL KD: 1.71e−07 Kass: 6.39e+04, kdiss: 1.09e−02
417_ID.1602_S5_SLLQSLIGL KD: 1.79e−07 Kass: 1.30e+05, kdiss: 2.33e−02
418_ID.1603_W7_SLLQHLWGL KD:~ Kass:~, , kdiss: 1.63e−02
419_ID.1604_S4_SLLSHLIGL KD: 1.32e−08 Kass: 1.32e+05, kdiss: 1.75e−03
420_ID.1605_Q1_QLLQHLIGL KD: 1.48e−08 Kass: 1.38e+05, kdiss: 2.04e−03
421_ID.1607_Y7_SLLQHLYGL KD: 3.77e−07 Kass: 7.42e+04, kdiss: 2.80e−02
422_ID.1609_G7_SLLQHLGGL KD: 8.24e−07 Kass: 5.06e+04, kdiss: 4.17e−02
423_ID.1612_T8_SLLQHLITL KD: 9.95e−09 Kass: 1.25e+05, kdiss: 1.24e−03
424_ID.1613_F6_SLLQHFIGL KD: 8.53e−09 Kass: 1.29e+05, kdiss: 1.10e−03
425_ID.1615_R6_SLLQHRIGL KD: 1.14e−08 Kass: 1.27e+05, kdiss: 1.45e−03
426_ID.1617_T1_TLLQHLIGL KD: 1.25e−08 Kass: 1.36e+05, kdiss: 1.70e−03
427_ID.1619_W3_SLWQHLIGL KD: 1.55e−08 Kass: 1.22e+05, kdiss: 1.89e−03
428_ID.162_S4_SLLSHLIGL KD: 1.05e−08 Kass: 8.28e+04, kdiss: 8.68e−04
429_ID.1620_N3_SLNQHLIGL KD: 2.00e−08 Kass: 1.36e+05, kdiss: 2.71e−03
430_ID.1621_F8_SLLQHLIFL KD:~ Kass:~, , kdiss: 1.48e−02
431_ID.1626_G5_SLLQGLIGL KD: 5.62e−07 Kass: 6.46e+04, kdiss: 3.63e−02
432_ID.1627_I3_SLIQHLIGL KD: 9.62e−09 Kass: 1.11e+05, kdiss: 1.07e−03
433_ID.1630_Q3_SLQQHLIGL KD: 7.86e−09 Kass: 1.28e+05, kdiss: 1.01e−03
434_ID.1631_A1_ALLQHLIGL KD: 1.46e−08 Kass: 1.16e+05, kdiss: 1.69e−03
435_ID.1635_V8_SLLQHLIVL KD: 5.41e−08 Kass: 5.55e+04, kdiss: 3.00e−03
436_ID.1636_W4_SLLWHLIGL KD: 1.00e−08 Kass: 9.71e+04, kdiss: 9.75e−04
437_ID.1637_K7_SLLQHLKGL KD: 1.17e−07 Kass: 5.77e+04, kdiss: 6.77e−03
438_ID.1638_Y4_SLLYHLIGL KD: 7.63e−09 Kass: 1.10e+05, kdiss: 8.40e−04
439_ID.1639_W7_SLLQHLWGL KD:~ Kass:~, , kdiss: 5.49e−02
440_ID.1641_S7_SLLQHLSGL KD: 2.52e−07 Kass: 5.32e+04, kdiss: 1.34e−02
441_ID.1643_W3_SLWQHLIGL KD: 2.43e−08 Kass: 7.79e+04, kdiss: 1.90e−03
442_ID.1644_G5_SLLQGLIGL KD: 4.51e−07 Kass: 4.70e+04, kdiss: 2.12e−02
443_ID.1645_A1_ALLQHLIGL KD: 1.35e−08 Kass: 9.40e+04, kdiss: 1.27e−03
444_ID.1648_H4_SLLHHLIGL KD: 2.05e−08 Kass: 8.52e+04, kdiss: 1.75e−03
445_ID.1649_M6_SLLQHMIGL KD: 1.45e−08 Kass: 9.04e+04, kdiss: 1.31e−03
446_ID.1650_N5_SLLQNLIGL KD: 2.37e−07 Kass: 8.05e+04, kdiss: 1.91e−02
447_ID.1652_I5_SLLQILIGL KD:~ Kass:~, , kdiss: 1.54e−03
448_ID.1654_Y4_SLLYHLIGL KD: 1.92e−08 Kass: 8.77e+04, kdiss: 1.69e−03
449_ID.1655_N1_NLLQHLIGL KD: 3.83e−09 Kass: 8.64e+04, kdiss: 3.31e−04
450_ID.1656_G5_SLLQGLIGL KD: 1.72e−06 Kass: 1.61e+04, kdiss: 2.77e−02
451_ID.1657_M7_SLLQHLMGL KD: 1.47e−08 Kass: 1.01e+05, kdiss: 1.48e−03
452_ID.1658_R4_SLLRHLIGL KD: 1.34e−08 Kass: 1.24e+05, kdiss: 1.66e−03
453_ID.1659_V1_VLLQHLIGL KD: 1.04e−08 Kass: 1.09e+05, kdiss: 1.14e−03
454_ID.1660_G5_SLLQGLIGL KD: 8.68e−07 Kass: 3.91e+04, kdiss: 3.39e−02
455_ID.1661_H8_SLLQHLIHL KD: 2.53e−07 Kass: 5.12e+04, kdiss: 1.30e−02
456_ID.1662_L4_SLLLHLIGL KD: 1.05e−08 Kass: 1.35e+05, kdiss: 1.42e−03
457_ID.1663_L7_SLLQHLLGL KD: 1.01e−08 Kass: 1.37e+05, kdiss: 1.39e−03
458_ID.1664_Y4_SLLYHLIGL KD: 1.76e−08 Kass: 1.29e+05, kdiss: 2.28e−03
459_ID.1665_F7_SLLQHLFGL KD: 5.09e−08 Kass: 1.28e+05, kdiss: 6.51e−03
460_ID.1669_A7_SLLQHLAGL KD: 4.30e−08 Kass: 1.32e+05, kdiss: 5.66e−03
461_ID.167_K7_SLLQHLKGL KD: 1.23e−07 Kass: 5.43e+04, kdiss: 6.69e−03
462_ID.1671_P7_SLLQHLPGL KD: 8.37e−07 Kass: 4.89e+04, kdiss: 4.09e−02
463_ID.1672_A4_SLLAHLIGL KD: 1.34e−08 Kass: 1.39e+05, kdiss: 1.86e−03
464_ID.1673_A3_SLAQHLIGL KD: 1.53e−08 Kass: 1.48e+05, kdiss: 2.27e−03
465_ID.1674_I4_SLLIHLIGL KD: 1.22e−08 Kass: 1.47e+05, kdiss: 1.79e−03
466_ID.1675_P8_SLLQHLIPL KD: 1.51e−08 Kass: 1.11e+05, kdiss: 1.68e−03
467_ID.1677_P7_SLLQHLPGL KD: 7.36e−07 Kass: 5.57e+04, kdiss: 4.10e−02
468_ID.1679_W6_SLLQHWIGL KD: 9.89e−09 Kass: 1.24e+05, kdiss: 1.23e−03
469_ID.168_R4_SLLRHLIGL KD: 8.56e−09 Kass: 1.37e+05, kdiss: 1.17e−03
470_ID.1680_E3_SLEQHLIGL KD: 1.17e−08 Kass: 1.40e+05, kdiss: 1.63e−03
471_ID.1681_I8_SLLQHLIIL KD: 2.53e−08 Kass: 8.32e+04, kdiss: 2.10e−03
472_ID.1684_E3_SLEQHLIGL KD: 1.06e−08 Kass: 1.46e+05, kdiss: 1.55e−03
473_ID.1686_N4_SLLNHLIGL KD: 1.10e−08 Kass: 1.35e+05, kdiss: 1.49e−03
474_ID.1687_L8_SLLQHLILL KD: 5.81e−08 Kass: 7.20e+04, kdiss: 4.19e−03
475_ID.1688_A5_SLLQALIGL KD: 9.15e−08 Kass: 1.37e+05, kdiss: 1.26e−02
476_ID.1689_P8_SLLQHLIPL KD: 1.49e−08 Kass: 1.07e+05, kdiss: 1.59e−03
477_ID.1690_W5_SLLQWLIGL KD: 5.95e−07 Kass: 4.04e+04, kdiss: 2.40e−02
478_ID.1691_Q7_SLLQHLQGL KD: 4.98e−07 Kass: 6.80e+04, kdiss: 3.39e−02
479_ID.1692_N3_SLNQHLIGL KD: 1.85e−08 Kass: 1.46e+05, kdiss: 2.70e−03
480_ID.1694_S3_SLSQHLIGL KD: 2.65e−08 Kass: 1.50e+05, kdiss: 3.98e−03
481_ID.1695_M8_SLLQHLIML KD: 9.58e−08 Kass: 6.33e+04, kdiss: 6.06e−03
482_ID.1696_S3_SLSQHLIGL KD: 2.60e−08 Kass: 1.38e+05, kdiss: 3.58e−03
483_ID.1698_I6_SLLQHIIGL KD: 7.13e−09 Kass: 1.18e+05, kdiss: 8.39e−04
484_ID.1699_Y7_SLLQHLYGL KD: 3.70e−07 Kass: 6.79e+04, kdiss: 2.51e−02
485_ID.17_N1_NLLQHLIGL KD: 1.70e−08 Kass: 7.87e+04, kdiss: 1.34e−03
486_ID.1700_Y4_SLLYHLIGL KD: 1.12e−08 Kass: 1.34e+05, kdiss: 1.50e−03
487_ID.1702_D4_SLLDHLIGL KD: 1.60e−08 Kass: 7.08e+04, kdiss: 1.13e−03
488_ID.1703_R6_SLLQHRIGL KD: 1.15e−08 Kass: 1.25e+05, kdiss: 1.44e−03
489_ID.1704_N5_SLLQNLIGL KD: 2.15e−07 Kass: 1.10e+05, kdiss: 2.37e−02
490_ID.1705_F8_SLLQHLIFL KD:~ Kass:~, , kdiss: 2.37e−02
491_ID.1706_K5_SLLQKLIGL KD: 3.31e−07 Kass: 7.13e+04, kdiss: 2.36e−02
492_ID.1707_H7_SLLQHLHGL KD: 8.19e−08 Kass: 4.44e+04, kdiss: 3.64e−03
493_ID.1709_F6_SLLQHFIGL KD: 7.31e−09 Kass: 1.01e+05, kdiss: 7.38e−04
494_ID.1710_D5_SLLQDLIGL KD: 1.59e−07 Kass: 7.27e+04, kdiss: 1.16e−02
495_ID.1712_W5_SLLQWLIGL KD:~ Kass:~, , kdiss: 2.93e−02
496_ID.1713_K3_SLKQHLIGL KD: 6.78e−09 Kass: 1.01e+05, kdiss: 6.82e−04
497_ID.1714_L5_SLLQLLIGL KD: 1.62e−06 Kass: 1.77e+04, kdiss: 2.87e−02
498_ID.1715_F8_SLLQHLIFL KD:~ Kass:~, , kdiss: 3.90e−02
499_ID.1716_T4_SLLTHLIGL KD: 1.27e−08 Kass: 1.01e+05, kdiss: 1.28e−03
500_ID.1719_H3_SLHQHLIGL KD: 8.84e−08 Kass: 5.88e+04, kdiss: 5.19e−03
value
Time
0
5000
10000
15000
0 250 500 750 1000

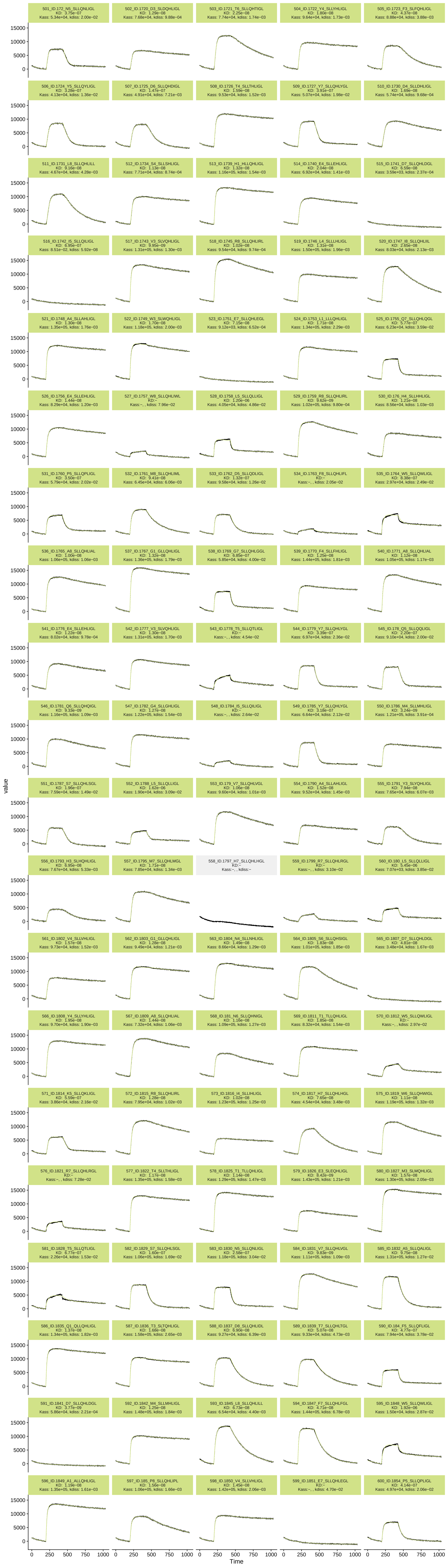

501_ID.172_N5_SLLQNLIGL KD: 3.75e−07 Kass: 5.34e+04, kdiss: 2.00e−02
502_ID.1720_D3_SLDQHLIGL KD: 1.29e−08 Kass: 7.68e+04, kdiss: 9.88e−04
503_ID.1721_T6_SLLQHTIGL KD: 2.25e−08 Kass: 7.74e+04, kdiss: 1.74e−03
504_ID.1722_Y4_SLLYHLIGL KD: 1.80e−08 Kass: 9.64e+04, kdiss: 1.73e−03
505_ID.1723_F3_SLFQHLIGL KD: 4.37e−08 Kass: 8.88e+04, kdiss: 3.88e−03
506_ID.1724_Y5_SLLQYLIGL KD: 3.28e−07 Kass: 4.13e+04, kdiss: 1.36e−02
507_ID.1725_D6_SLLQHDIGL KD: 1.47e−07 Kass: 4.91e+04, kdiss: 7.21e−03
508_ID.1726_T4_SLLTHLIGL KD: 1.59e−08 Kass: 9.53e+04, kdiss: 1.52e−03
509_ID.1727_Y7_SLLQHLYGL KD: 3.91e−07 Kass: 5.07e+04, kdiss: 1.98e−02
510_ID.1730_D4_SLLDHLIGL KD: 1.69e−08 Kass: 5.74e+04, kdiss: 9.68e−04
511_ID.1731_L8_SLLQHLILL KD: 9.16e−08 Kass: 4.67e+04, kdiss: 4.28e−03
512_ID.1734_S4_SLLSHLIGL KD: 1.13e−08 Kass: 7.71e+04, kdiss: 8.74e−04
513_ID.1739_H1_HLLQHLIGL KD: 1.32e−08 Kass: 1.16e+05, kdiss: 1.54e−03
514_ID.1740_E4_SLLEHLIGL KD: 2.04e−08 Kass: 6.92e+04, kdiss: 1.41e−03
515_ID.1741_D7_SLLQHLDGL KD: 6.59e−08 Kass: 3.59e+03, kdiss: 2.37e−04
516_ID.1742_I5_SLLQILIGL KD: 6.95e−07 Kass: 8.51e−02, kdiss: 5.92e−08
517_ID.1743_V3_SLVQHLIGL KD: 9.95e−09 Kass: 1.31e+05, kdiss: 1.30e−03
518_ID.1745_R8_SLLQHLIRL KD: 1.02e−08 Kass: 9.54e+04, kdiss: 9.74e−04
519_ID.1746_L4_SLLLHLIGL KD: 1.31e−08 Kass: 1.50e+05, kdiss: 1.96e−03
520_ID.1747_I8_SLLQHLIIL KD: 2.65e−08 Kass: 8.03e+04, kdiss: 2.13e−03
521_ID.1748_A4_SLLAHLIGL KD: 1.30e−08 Kass: 1.35e+05, kdiss: 1.76e−03
522_ID.1749_W3_SLWQHLIGL KD: 1.70e−08 Kass: 1.18e+05, kdiss: 2.00e−03
523_ID.1751_E7_SLLQHLEGL KD: 7.15e−08 Kass: 9.12e+03, kdiss: 6.52e−04
524_ID.1753_L1_LLLQHLIGL KD: 1.71e−08 Kass: 1.34e+05, kdiss: 2.29e−03
525_ID.1755_Q7_SLLQHLQGL KD: 5.77e−07 Kass: 6.23e+04, kdiss: 3.59e−02
526_ID.1756_E4_SLLEHLIGL KD: 1.44e−08 Kass: 8.29e+04, kdiss: 1.20e−03
527_ID.1757_W8_SLLQHLIWL KD:− Kass:−, , kdiss: 7.96e−02
528_ID.1758_L5_SLLQLLIGL KD: 1.20e−06 Kass: 4.05e+04, kdiss: 4.86e−02
529_ID.1759_R8_SLLQHLIRL KD: 9.62e−09 Kass: 1.02e+05, kdiss: 9.80e−04
530_ID.176_H4_SLLHHLIGL KD: 1.21e−08 Kass: 8.56e+04, kdiss: 1.03e−03
531_ID.1760_P5_SLLQPLIGL KD: 3.50e−07 Kass: 5.79e+04, kdiss: 2.02e−02
532_ID.1761_M8_SLLQHLIML KD: 9.41e−08 Kass: 6.45e+04, kdiss: 6.06e−03
533_ID.1762_D5_SLLQDLIGL KD: 1.32e−07 Kass: 9.58e+04, kdiss: 1.26e−02
534_ID.1763_F8_SLLQHLIFL KD:− Kass:−, , kdiss: 2.05e−02
535_ID.1764_W5_SLLQWLIGL KD: 8.38e−07 Kass: 2.97e+04, kdiss: 2.49e−02
536_ID.1765_A8_SLLQHLIAL KD: 1.00e−08 Kass: 1.06e+05, kdiss: 1.06e−03
537_ID.1767_G1_GLLQHLIGL KD: 1.32e−08 Kass: 1.36e+05, kdiss: 1.79e−03
538_ID.1769_G7_SLLQHLGGL KD: 6.85e−07 Kass: 5.85e+04, kdiss: 4.00e−02
539_ID.1770_F4_SLLFHLIGL KD: 1.25e−08 Kass: 1.44e+05, kdiss: 1.81e−03
540_ID.1771_A8_SLLQHLIAL KD: 1.12e−08 Kass: 1.05e+05, kdiss: 1.17e−03
541_ID.1776_E4_SLLEHLIGL KD: 1.22e−08 Kass: 8.02e+04, kdiss: 9.78e−04
542_ID.1777_V3_SLVQHLIGL KD: 1.30e−08 Kass: 1.31e+05, kdiss: 1.70e−03
543_ID.1778_T5_SLLQTLIGL KD:− Kass:−, , kdiss: 4.54e−02
544_ID.1779_Y7_SLLQHLYGL KD: 3.39e−07 Kass: 6.97e+04, kdiss: 2.36e−02
545_ID.178_Q5_SLLQQLIGL KD: 2.20e−07 Kass: 9.10e+04, kdiss: 2.00e−02
546_ID.1781_Q6_SLLQHQIGL KD: 9.33e−09 Kass: 1.16e+05, kdiss: 1.09e−03
547_ID.1782_G4_SLLGHLIGL KD: 1.27e−08 Kass: 1.22e+05, kdiss: 1.54e−03
548_ID.1784_I5_SLLQILIGL KD:− Kass:−, , kdiss: 2.64e−02
549_ID.1785_Y7_SLLQHLYGL KD: 3.18e−07 Kass: 6.64e+04, kdiss: 2.12e−02
550_ID.1786_M4_SLLMHLIGL KD: 3.24e−09 Kass: 1.21e+05, kdiss: 3.91e−04
551_ID.1787_S7_SLLQHLSGL KD: 1.96e−07 Kass: 7.59e+04, kdiss: 1.49e−02
552_ID.1788_L5_SLLQLLIGL KD: 1.62e−06 Kass: 1.90e+04, kdiss: 3.09e−02
553_ID.179_V7_SLLQHLVGL KD: 1.06e−08 Kass: 9.60e+04, kdiss: 1.01e−03
554_ID.1790_A4_SLLAHLIGL KD: 1.52e−08 Kass: 9.52e+04, kdiss: 1.45e−03
555_ID.1791_Y3_SLYQHLIGL KD: 7.94e−08 Kass: 7.65e+04, kdiss: 6.07e−03
556_ID.1793_H3_SLHQHLIGL KD: 6.95e−08 Kass: 7.67e+04, kdiss: 5.33e−03
557_ID.1795_M7_SLLQHLMGL KD: 1.71e−08 Kass: 7.85e+04, kdiss: 1.34e−03
558_ID.1797_H7_SLLQHLHGL KD:− Kass:−, , kdiss:−
559_ID.1799_R7_SLLQHLRGL KD:− Kass:−, , kdiss: 3.10e−02
560_ID.180_L5_SLLQLLIGL KD: 5.45e−06 Kass: 7.07e+03, kdiss: 3.85e−02
561_ID.1802_V4_SLLVHLIGL KD: 1.57e−08 Kass: 9.73e+04, kdiss: 1.52e−03
562_ID.1803_G1_GLLQHLIGL KD: 1.28e−08 Kass: 9.49e+04, kdiss: 1.21e−03
563_ID.1804_N4_SLLNHLIGL KD: 1.49e−08 Kass: 8.66e+04, kdiss: 1.29e−03
564_ID.1805_S6_SLLQHSIGL KD: 1.83e−08 Kass: 1.01e+05, kdiss: 1.85e−03
565_ID.1807_D7_SLLQHLDGL KD: 4.81e−08 Kass: 3.48e+04, kdiss: 1.67e−03
566_ID.1808_Y4_SLLYHLIGL KD: 1.95e−08 Kass: 9.70e+04, kdiss: 1.90e−03
567_ID.1809_A8_SLLQHLIAL KD: 1.44e−08 Kass: 7.32e+04, kdiss: 1.06e−03
568_ID.181_N6_SLLQHNIGL KD: 1.16e−08 Kass: 1.09e+05, kdiss: 1.27e−03
569_ID.1811_T1_TLLQHLIGL KD: 1.85e−08 Kass: 8.32e+04, kdiss: 1.54e−03
570_ID.1812_W5_SLLQWLIGL KD:− Kass:−, , kdiss: 2.97e−02
571_ID.1814_K5_SLLQKLIGL KD: 5.59e−07 Kass: 3.86e+04, kdiss: 2.16e−02
572_ID.1815_R8_SLLQHLIRL KD: 1.28e−08 Kass: 7.95e+04, kdiss: 1.02e−03
573_ID.1816_I4_SLLIHLIGL KD: 1.02e−08 Kass: 1.23e+05, kdiss: 1.25e−03
574_ID.1817_H7_SLLQHLHGL KD: 7.65e−08 Kass: 4.54e+04, kdiss: 3.48e−03
575_ID.1819_W6_SLLQHWIGL KD: 1.11e−08 Kass: 1.19e+05, kdiss: 1.32e−03
576_ID.1821_R7_SLLQHLRGL KD:− Kass:−, , kdiss: 7.28e−02
577_ID.1822_T4_SLLTHLIGL KD: 1.17e−08 Kass: 1.35e+05, kdiss: 1.58e−03
578_ID.1825_T1_TLLQHLIGL KD: 1.14e−08 Kass: 1.29e+05, kdiss: 1.47e−03
579_ID.1826_E3_SLEQHLIGL KD: 8.42e−09 Kass: 1.43e+05, kdiss: 1.21e−03
580_ID.1827_M3_SLMQHLIGL KD: 1.57e−08 Kass: 1.30e+05, kdiss: 2.05e−03
581_ID.1828_T5_SLLQTLIGL KD: 6.77e−07 Kass: 2.26e+04, kdiss: 1.53e−02
582_ID.1829_S7_SLLQHLSGL KD: 1.60e−07 Kass: 1.06e+05, kdiss: 1.69e−02
583_ID.1830_N5_SLLQNLIGL KD: 2.58e−07 Kass: 1.18e+05, kdiss: 3.04e−02
584_ID.1831_V7_SLLQHLVGL KD: 9.83e−09 Kass: 1.11e+05, kdiss: 1.09e−03
585_ID.1832_A5_SLLQALIGL KD: 9.75e−08 Kass: 1.31e+05, kdiss: 1.27e−02
586_ID.1835_Q1_QLLQHLIGL KD: 1.37e−08 Kass: 1.34e+05, kdiss: 1.82e−03
587_ID.1836_T3_SLTQHLIGL KD: 1.68e−08 Kass: 1.58e+05, kdiss: 2.65e−03
588_ID.1837_D8_SLLQHLIDL KD: 6.90e−08 Kass: 9.27e+04, kdiss: 6.39e−03
589_ID.1839_T7_SLLQHLTGL KD: 5.07e−08 Kass: 9.33e+04, kdiss: 4.73e−03
590_ID.184_F5_SLLQFLIGL KD: 4.77e−07 Kass: 7.94e+04, kdiss: 3.78e−02
591_ID.1841_D7_SLLQHLDGL KD: 3.77e−09 Kass: 5.86e+04, kdiss: 2.21e−04
592_ID.1842_M4_SLLMHLIGL KD: 1.25e−08 Kass: 1.48e+05, kdiss: 1.84e−03
593_ID.1845_L8_SLLQHLILL KD: 6.73e−08 Kass: 6.54e+04, kdiss: 4.40e−03
594_ID.1847_F7_SLLQHLFGL KD: 4.71e−08 Kass: 1.44e+05, kdiss: 6.78e−03
595_ID.1848_W5_SLLQWLIGL KD: 1.92e−06 Kass: 1.50e+04, kdiss: 2.87e−02
596_ID.1849_A1_ALLQHLIGL KD: 1.19e−08 Kass: 1.35e+05, kdiss: 1.61e−03
597_ID.185_P8_SLLQHLIPL KD: 1.56e−08 Kass: 1.06e+05, kdiss: 1.66e−03
598_ID.1850_V4_SLLVHLIGL KD: 1.45e−08 Kass: 1.42e+05, kdiss: 2.06e−03
599_ID.1851_E7_SLLQHLEGL KD:− Kass:−, , kdiss: 4.70e−02
600_ID.1854_P5_SLLQPLIGL KD: 4.14e−07 Kass: 4.97e+04, kdiss: 2.06e−02
value
0
5000
10000
15000
Time
0
250
500
750
1000

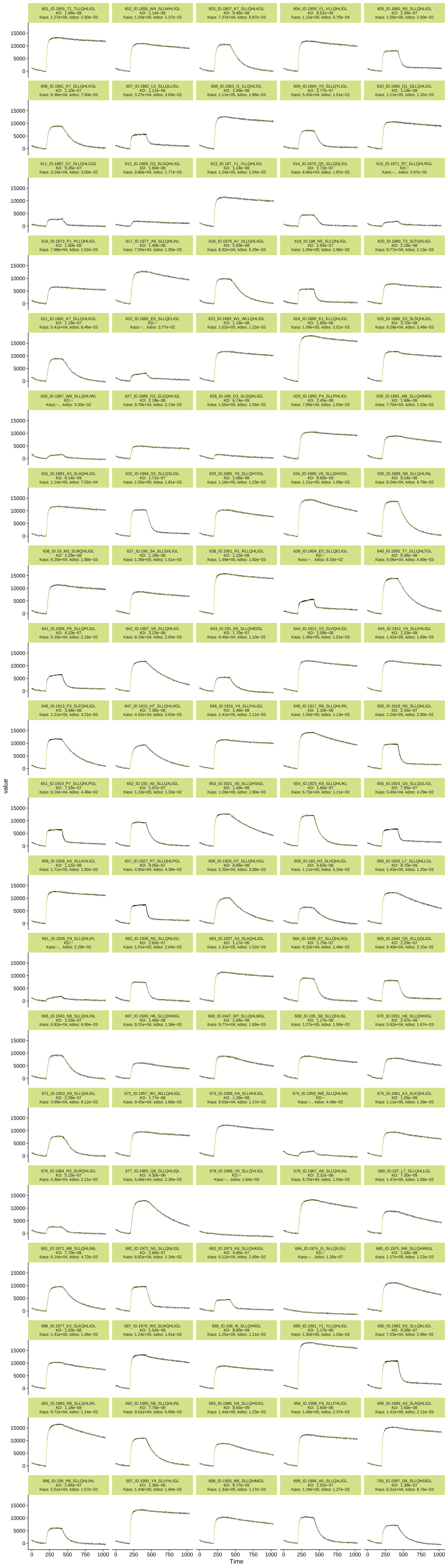

value
Time
0
5000
10000
15000
0
250
500
750
1000

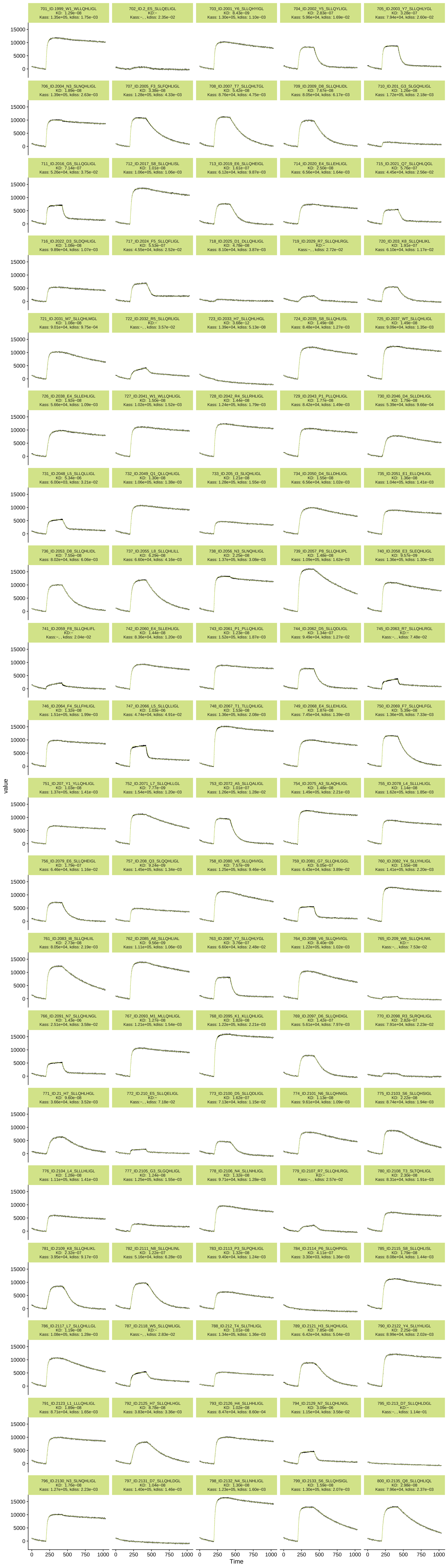

701_ID.1999_W1_WLLQHLIGL KD: 1.29e−08 Kass: 1.35e+05, kdiss: 1.75e−03
702_ID.2_E5_SLLQELIGL KD:− Kass:−, , kdiss: 2.35e−02
703_ID.2001_Y6_SLLQHYIGL KD: 8.43e−09 Kass: 1.30e+05, kdiss: 1.10e−03
704_ID.2002_Y5_SLLQYLIGL KD: 2.83e−07 Kass: 5.96e+04, kdiss: 1.69e−02
705_ID.2003_Y7_SLLQHLYGL KD: 3.28e−07 Kass: 7.94e+04, kdiss: 2.60e−02
706_ID.2004_N3_SLNQHLIGL KD: 1.89e−08 Kass: 1.39e+05, kdiss: 2.63e−03
707_ID.2005_F3_SLFQHLIGL KD: 3.38e−08 Kass: 1.28e+05, kdiss: 4.33e−03
708_ID.2007_T7_SLLQHLTGL KD: 5.42e−08 Kass: 8.76e+04, kdiss: 4.75e−03
709_ID.2009_D8_SLLQHLIDL KD: 7.67e−08 Kass: 8.05e+04, kdiss: 6.17e−03
710_ID.201_G3_SLGQHLIGL KD: 1.26e−08 Kass: 1.72e+05, kdiss: 2.18e−03
711_ID.2016_G5_SLLQGLIGL KD: 7.14e−07 Kass: 5.26e+04, kdiss: 3.75e−02
712_ID.2017_S8_SLLQHLISL KD: 1.01e−08 Kass: 1.06e+05, kdiss: 1.06e−03
713_ID.2019_E6_SLLQHEIGL KD: 1.61e−07 Kass: 6.12e+04, kdiss: 9.87e−03
714_ID.2020_E4_SLLEHLIGL KD: 2.50e−08 Kass: 6.56e+04, kdiss: 1.64e−03
715_ID.2021_Q7_SLLQHLQGL KD: 5.76e−07 Kass: 4.45e+04, kdiss: 2.56e−02
716_ID.2022_D3_SLDQHLIGL KD: 1.08e−08 Kass: 9.89e+04, kdiss: 1.07e−03
717_ID.2024_F5_SLLQFLIGL KD: 5.53e−07 Kass: 4.55e+04, kdiss: 2.52e−02
718_ID.2025_D1_DLLQHLIGL KD: 4.78e−08 Kass: 8.10e+04, kdiss: 3.87e−03
719_ID.2029_R7_SLLQHLRGL KD:− Kass:−, , kdiss: 2.72e−02
720_ID.203_K8_SLLQHLIKL KD: 1.91e−07 Kass: 6.10e+04, kdiss: 1.17e−02
721_ID.2031_M7_SLLQHLMGL KD: 1.08e−08 Kass: 9.01e+04, kdiss: 9.75e−04
722_ID.2032_R5_SLLQRLIGL KD:− Kass:−, , kdiss: 3.57e−02
723_ID.2033_H7_SLLQHLHGL KD: 3.68e−12 Kass: 1.39e+04, kdiss: 5.13e−08
724_ID.2035_S8_SLLQHLISL KD: 1.49e−08 Kass: 8.48e+04, kdiss: 1.27e−03
725_ID.2037_WT_SLLQHLIGL KD: 1.49e−08 Kass: 9.09e+04, kdiss: 1.35e−03
726_ID.2038_E4_SLLEHLIGL KD: 1.92e−08 Kass: 5.66e+04, kdiss: 1.09e−03
727_ID.2041_W1_WLLQHLIGL KD: 1.50e−08 Kass: 1.02e+05, kdiss: 1.52e−03
728_ID.2042_R4_SLLRHLIGL KD: 1.44e−08 Kass: 1.24e+05, kdiss: 1.79e−03
729_ID.2043_P1_PLLQHLIGL KD: 1.77e−08 Kass: 8.42e+04, kdiss: 1.49e−03
730_ID.2046_D4_SLLDHLIGL KD: 1.79e−08 Kass: 5.39e+04, kdiss: 9.66e−04
731_ID.2048_L5_SLLQLLIGL KD: 5.34e−06 Kass: 6.00e+03, kdiss: 3.21e−02
732_ID.2049_Q1_QLLQHLIGL KD: 1.30e−08 Kass: 1.06e+05, kdiss: 1.38e−03
733_ID.205_I3_SLIQHLIGL KD: 1.21e−08 Kass: 1.28e+05, kdiss: 1.55e−03
734_ID.2050_D4_SLLDHLIGL KD: 1.55e−08 Kass: 6.56e+04, kdiss: 1.02e−03
735_ID.2051_E1_ELLQHLIGL KD: 1.36e−08 Kass: 1.04e+05, kdiss: 1.41e−03
736_ID.2053_D8_SLLQHLIDL KD: 7.55e−08 Kass: 8.02e+04, kdiss: 6.06e−03
737_ID.2055_L8_SLLQHLILL KD: 6.29e−08 Kass: 6.60e+04, kdiss: 4.16e−03
738_ID.2056_N3_SLNQHLIGL KD: 2.25e−08 Kass: 1.37e+05, kdiss: 3.08e−03
739_ID.2057_P8_SLLQHLIPL KD: 1.48e−08 Kass: 1.09e+05, kdiss: 1.62e−03
740_ID.2058_E3_SLEQHLIGL KD: 9.57e−09 Kass: 1.36e+05, kdiss: 1.30e−03
741_ID.2059_F8_SLLQHLIFL KD:− Kass:−, , kdiss: 2.04e−02
742_ID.2060_E4_SLLEHLIGL KD: 1.44e−08 Kass: 8.36e+04, kdiss: 1.20e−03
743_ID.2061_P1_PLLQHLIGL KD: 1.23e−08 Kass: 1.52e+05, kdiss: 1.87e−03
744_ID.2062_D5_SLLQDLIGL KD: 1.34e−07 Kass: 9.49e+04, kdiss: 1.27e−02
745_ID.2063_R7_SLLQHLRGL KD:− Kass:−, , kdiss: 7.48e−02
746_ID.2064_F4_SLLFHLIGL KD: 1.32e−08 Kass: 1.51e+05, kdiss: 1.99e−03
747_ID.2066_L5_SLLQLLIGL KD: 1.03e−06 Kass: 4.74e+04, kdiss: 4.91e−02
748_ID.2067_T1_TLLQHLIGL KD: 1.53e−08 Kass: 1.36e+05, kdiss: 2.08e−03
749_ID.2068_E4_SLLEHLIGL KD: 1.87e−08 Kass: 7.45e+04, kdiss: 1.39e−03
750_ID.2069_F7_SLLQHLFGL KD: 5.39e−08 Kass: 1.36e+05, kdiss: 7.33e−03
751_ID.207_Y1_YLLQHLIGL KD: 1.03e−08 Kass: 1.37e+05, kdiss: 1.41e−03
752_ID.2071_L7_SLLQHLLGL KD: 7.77e−09 Kass: 1.54e+05, kdiss: 1.20e−03
753_ID.2072_A5_SLLQALIGL KD: 1.01e−07 Kass: 1.26e+05, kdiss: 1.28e−02
754_ID.2075_A3_SLAQHLIGL KD: 1.48e−08 Kass: 1.49e+05, kdiss: 2.21e−03
755_ID.2078_L4_SLLLHLIGL KD: 1.14e−08 Kass: 1.62e+05, kdiss: 1.85e−03
756_ID.2079_E6_SLLQHEIGL KD: 1.79e−07 Kass: 6.46e+04, kdiss: 1.16e−02
757_ID.208_Q3_SLQQHLIGL KD: 9.24e−09 Kass: 1.45e+05, kdiss: 1.34e−03
758_ID.2080_V6_SLLQHVIGL KD: 7.57e−09 Kass: 1.25e+05, kdiss: 9.46e−04
759_ID.2081_G7_SLLQHLGGL KD: 6.05e−07 Kass: 6.43e+04, kdiss: 3.89e−02
760_ID.2082_Y4_SLLYHLIGL KD: 1.55e−08 Kass: 1.41e+05, kdiss: 2.20e−03
761_ID.2083_I8_SLLQHLIIL KD: 2.73e−08 Kass: 8.05e+04, kdiss: 2.19e−03
762_ID.2085_A8_SLLQHLIAL KD: 9.56e−09 Kass: 1.11e+05, kdiss: 1.06e−03
763_ID.2087_Y7_SLLQHLYGL KD: 3.76e−07 Kass: 6.60e+04, kdiss: 2.48e−02
764_ID.2088_V6_SLLQHVIGL KD: 8.40e−09 Kass: 1.22e+05, kdiss: 1.02e−03
765_ID.209_W8_SLLQHLIWL KD:− Kass:−, , kdiss: 7.53e−02
766_ID.2091_N7_SLLQHLNGL KD: 1.43e−06 Kass: 2.51e+04, kdiss: 3.58e−02
767_ID.2093_M1_MLLQHLIGL KD: 1.27e−08 Kass: 1.21e+05, kdiss: 1.54e−03
768_ID.2095_K1_KLLQHLIGL KD: 1.82e−08 Kass: 1.22e+05, kdiss: 2.21e−03
769_ID.2097_D6_SLLQHDIGL KD: 1.42e−07 Kass: 5.61e+04, kdiss: 7.97e−03
770_ID.2098_R3_SLRQHLIGL KD: 2.82e−07 Kass: 7.91e+04, kdiss: 2.23e−02
771_ID.21_H7_SLLQHLHGL KD: 9.60e−08 Kass: 3.66e+04, kdiss: 3.52e−03
772_ID.210_E5_SLLQELIGL KD:− Kass:−, , kdiss: 7.18e−02
773_ID.2100_D5_SLLQDLIGL KD: 1.62e−07 Kass: 7.13e+04, kdiss: 1.15e−02
774_ID.2101_N6_SLLQHNIGL KD: 1.13e−08 Kass: 9.61e+04, kdiss: 1.09e−03
775_ID.2103_S6_SLLQHSIGL KD: 2.22e−08 Kass: 8.74e+04, kdiss: 1.94e−03
776_ID.2104_L4_SLLLHLIGL KD: 1.28e−08 Kass: 1.11e+05, kdiss: 1.41e−03
777_ID.2105_G3_SLGQHLIGL KD: 1.24e−08 Kass: 1.25e+05, kdiss: 1.55e−03
778_ID.2106_N4_SLLNHLIGL KD: 1.32e−08 Kass: 9.71e+04, kdiss: 1.28e−03
779_ID.2107_R7_SLLQHLRGL KD:− Kass:−, , kdiss: 2.57e−02
780_ID.2108_T3_SLTQHLIGL KD: 2.30e−08 Kass: 8.31e+04, kdiss: 1.91e−03
781_ID.2109_K8_SLLQHLIKL KD: 2.32e−07 Kass: 3.95e+04, kdiss: 9.17e−03
782_ID.2111_N8_SLLQHLINL KD: 1.22e−07 Kass: 5.16e+04, kdiss: 6.28e−03
783_ID.2113_P3_SLPQHLIGL KD: 1.32e−08 Kass: 9.40e+04, kdiss: 1.24e−03
784_ID.2114_P6_SLLQHPIGL KD: 4.11e−07 Kass: 3.30e+03, kdiss: 1.36e−03
785_ID.2115_S8_SLLQHLISL KD: 1.79e−08 Kass: 8.08e+04, kdiss: 1.44e−03
786_ID.2117_L7_SLLQHLLGL KD: 1.19e−08 Kass: 1.08e+05, kdiss: 1.28e−03
787_ID.2118_W5_SLLQWLIGL KD:− Kass:−, , kdiss: 2.83e−02
788_ID.212_T4_SLLTHLIGL KD: 1.01e−08 Kass: 1.34e+05, kdiss: 1.36e−03
789_ID.2121_H3_SLHQHLIGL KD: 7.85e−08 Kass: 6.42e+04, kdiss: 5.04e−03
790_ID.2122_Y4_SLLYHLIGL KD: 2.25e−08 Kass: 8.99e+04, kdiss: 2.02e−03
791_ID.2123_L1_LLLQHLIGL KD: 1.89e−08 Kass: 8.71e+04, kdiss: 1.65e−03
792_ID.2125_H7_SLLQHLHGL KD: 8.78e−08 Kass: 3.83e+04, kdiss: 3.36e−03
793_ID.2126_H4_SLLHHLIGL KD: 1.02e−08 Kass: 8.47e+04, kdiss: 8.60e−04
794_ID.2129_N7_SLLQHLNGL KD: 3.09e−06 Kass: 1.15e+04, kdiss: 3.56e−02
795_ID.213_D7_SLLQHLDGL KD:− Kass:−, , kdiss: 1.14e−01
796_ID.2130_N3_SLNQHLIGL KD: 1.76e−08 Kass: 1.27e+05, kdiss: 2.23e−03
797_ID.2131_D7_SLLQHLDGL KD: 1.04e−08 Kass: 1.40e+05, kdiss: 1.46e−03
798_ID.2132_N4_SLLNHLIGL KD: 1.30e−08 Kass: 1.23e+05, kdiss: 1.60e−03
799_ID.2133_S6_SLLQHSIGL KD: 1.59e−08 Kass: 1.30e+05, kdiss: 2.07e−03
800_ID.2135_Q8_SLLQHLIQL KD: 2.98e−08 Kass: 7.96e+04, kdiss: 2.37e−03
value
0 5000 10000 15000
0 250 500 750 1000
Time

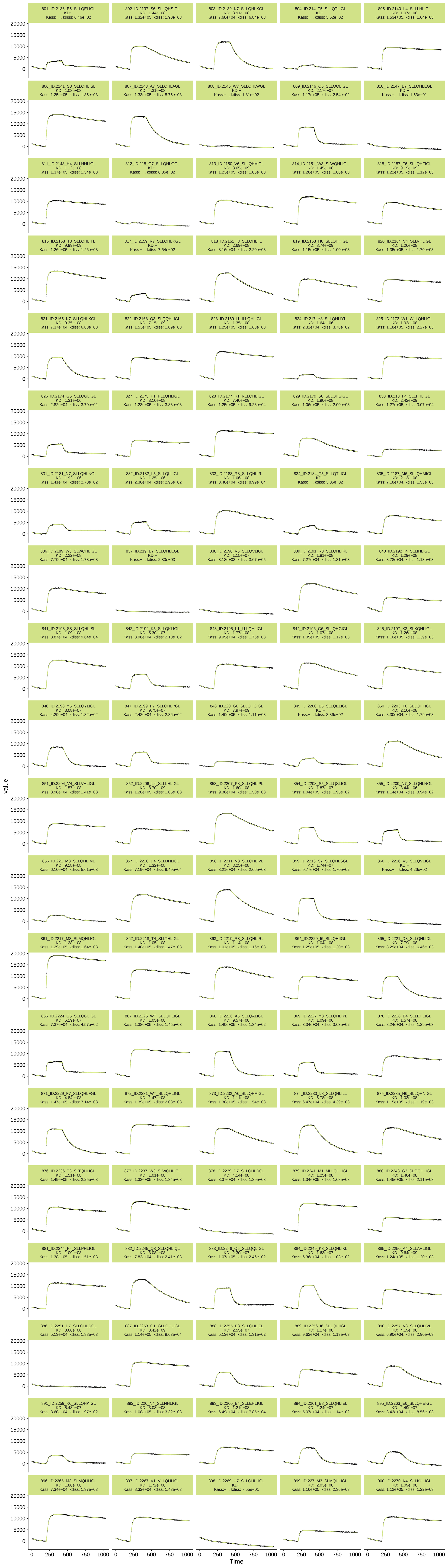
801_ID.2136_E5_SLLQELIGL
KD:−
Kass:−, , kdiss: 6.46e−02
802_ID.2137_S6_SLLQHSIGL
KD: 1.44e−08
Kass: 1.32e+05, kdiss: 1.90e−03
803_ID.2139_K7_SLLQHLKGL
KD: 8.91e−08
Kass: 7.68e+04, kdiss: 6.84e−03
804_ID.214_T5_SLLQTLIGL
KD:−
Kass:−, , kdiss: 3.62e−02
805_ID.2140_L4_SLLLHLIGL
KD: 1.07e−08
Kass: 1.53e+05, kdiss: 1.64e−03
806_ID.2141_S8_SLLQHLISL
KD: 1.08e−08
Kass: 1.25e+05, kdiss: 1.35e−03
807_ID.2143_A7_SLLQHLAGL
KD: 4.31e−08
Kass: 1.33e+05, kdiss: 5.75e−03
808_ID.2145_W7_SLLQHLWGL
KD:−
Kass:−, , kdiss: 1.81e−02
809_ID.2146_Q5_SLLQQLIGL
KD: 2.17e−07
Kass: 1.17e+05, kdiss: 2.54e−02
810_ID.2147_E7_SLLQHLEGL
KD:−
Kass:−, , kdiss: 1.53e−01
811_ID.2148_H4_SLLHHLIGL
KD: 1.12e−08
Kass: 1.37e+05, kdiss: 1.54e−03
812_ID.215_G7_SLLQHLGGL
KD:−
Kass:−, , kdiss: 6.05e−02
813_ID.2150_V6_SLLQHVIGL
KD: 8.65e−09
Kass: 1.23e+05, kdiss: 1.06e−03
814_ID.2151_W3_SLWQHLIGL
KD: 1.45e−08
Kass: 1.28e+05, kdiss: 1.86e−03
815_ID.2157_F6_SLLQHFIGL
KD: 9.19e−09
Kass: 1.22e+05, kdiss: 1.12e−03
816_ID.2158_T8_SLLQHLITL
KD: 9.99e−09
Kass: 1.26e+05, kdiss: 1.26e−03
817_ID.2159_R7_SLLQHLRGL
KD:−
Kass:−, , kdiss: 7.64e−02
818_ID.2161_I8_SLLQHLIIL
KD: 2.69e−08
Kass: 8.16e+04, kdiss: 2.20e−03
819_ID.2163_H6_SLLQHHIGL
KD: 8.74e−09
Kass: 1.15e+05, kdiss: 1.00e−03
820_ID.2164_V4_SLLVHLIGL
KD: 1.26e−08
Kass: 1.35e+05, kdiss: 1.70e−03
821_ID.2165_K7_SLLQHLKGL
KD: 9.35e−08
Kass: 7.37e+04, kdiss: 6.88e−03
822_ID.2168_Q3_SLQQHLIGL
KD: 7.15e−09
Kass: 1.53e+05, kdiss: 1.09e−03
823_ID.2169_I1_ILLQHLIGL
KD: 1.35e−08
Kass: 1.25e+05, kdiss: 1.68e−03
824_ID.217_Y8_SLLQHLIYL
KD: 1.64e−06
Kass: 2.31e+04, kdiss: 3.78e−02
825_ID.2173_W1_WLLQHLIGL
KD: 1.93e−08
Kass: 1.18e+05, kdiss: 2.27e−03
826_ID.2174_G5_SLLQGLIGL
KD: 1.31e−06
Kass: 2.82e+04, kdiss: 3.70e−02
827_ID.2175_P1_PLLQHLIGL
KD: 3.10e−08
Kass: 1.23e+05, kdiss: 3.83e−03
828_ID.2177_R1_RLLQHLIGL
KD: 7.40e−09
Kass: 1.25e+05, kdiss: 9.23e−04
829_ID.2179_S6_SLLQHSIGL
KD: 1.90e−08
Kass: 1.06e+05, kdiss: 2.00e−03
830_ID.218_F4_SLLFHLIGL
KD: 2.42e−09
Kass: 1.27e+05, kdiss: 3.07e−04
831_ID.2181_N7_SLLQHLNGL
KD: 1.92e−06
Kass: 1.41e+04, kdiss: 2.70e−02
832_ID.2182_L5_SLLQLLIGL
KD: 1.25e−06
Kass: 2.36e+04, kdiss: 2.95e−02
833_ID.2183_R8_SLLQHLIRL
KD: 1.06e−08
Kass: 8.48e+04, kdiss: 8.99e−04
834_ID.2184_T5_SLLQTLIGL
KD:−
Kass:−, , kdiss: 3.05e−02
835_ID.2187_M6_SLLQHMIGL
KD: 2.13e−08
Kass: 7.18e+04, kdiss: 1.53e−03
836_ID.2189_W3_SLWQHLIGL
KD: 2.22e−08
Kass: 7.79e+04, kdiss: 1.73e−03
837_ID.219_E7_SLLQHLEGL
KD:−
Kass:−, , kdiss: 2.80e−03
838_ID.2190_V5_SLLQVLIGL
KD: 1.15e−07
Kass: 3.18e+02, kdiss: 3.67e−05
839_ID.2191_R8_SLLQHLIRL
KD: 1.81e−08
Kass: 7.27e+04, kdiss: 1.31e−03
840_ID.2192_I4_SLLIHLIGL
KD: 1.29e−08
Kass: 8.78e+04, kdiss: 1.13e−03
841_ID.2193_S8_SLLQHLISL
KD: 1.09e−08
Kass: 8.87e+04, kdiss: 9.64e−04
842_ID.2194_K5_SLLQKLIGL
KD: 5.30e−07
Kass: 3.96e+04, kdiss: 2.10e−02
843_ID.2195_L1_LLLQHLIGL
KD: 1.77e−08
Kass: 9.95e+04, kdiss: 1.76e−03
844_ID.2196_G6_SLLQHGIGL
KD: 1.07e−08
Kass: 1.05e+05, kdiss: 1.12e−03
845_ID.2197_K3_SLKQHLIGL
KD: 1.26e−08
Kass: 1.10e+05, kdiss: 1.39e−03
846_ID.2198_Y5_SLLQYLIGL
KD: 3.08e−07
Kass: 4.29e+04, kdiss: 1.32e−02
847_ID.2199_P7_SLLQHLPGL
KD: 9.75e−07
Kass: 2.42e+04, kdiss: 2.36e−02
848_ID.220_G6_SLLQHGIGL
KD: 7.97e−09
Kass: 1.40e+05, kdiss: 1.11e−03
849_ID.2200_E5_SLLQELIGL
KD:−
Kass:−, , kdiss: 3.36e−02
850_ID.2203_T6_SLLQHTIGL
KD: 2.16e−08
Kass: 8.30e+04, kdiss: 1.79e−03
851_ID.2204_V4_SLLVHLIGL
KD: 1.57e−08
Kass: 8.98e+04, kdiss: 1.41e−03
852_ID.2206_L4_SLLLHLIGL
KD: 8.70e−09
Kass: 1.20e+05, kdiss: 1.05e−03
853_ID.2207_P8_SLLQHLIPL
KD: 1.60e−08
Kass: 9.36e+04, kdiss: 1.50e−03
854_ID.2208_S5_SLLQSLIGL
KD: 1.87e−07
Kass: 1.04e+05, kdiss: 1.95e−02
855_ID.2209_N7_SLLQHLNGL
KD: 3.44e−06
Kass: 1.14e+04, kdiss: 3.94e−02
856_ID.221_M8_SLLQHLIML
KD: 9.18e−08
Kass: 6.10e+04, kdiss: 5.61e−03
857_ID.2210_D4_SLLDHLIGL
KD: 1.32e−08
Kass: 7.19e+04, kdiss: 9.49e−04
858_ID.2211_V8_SLLQHLIVL
KD: 3.25e−08
Kass: 8.21e+04, kdiss: 2.66e−03
859_ID.2213_S7_SLLQHLSGL
KD: 1.74e−07
Kass: 9.77e+04, kdiss: 1.70e−02
860_ID.2216_V5_SLLQVLIGL
KD:−
Kass:−, , kdiss: 4.26e−02
861_ID.2217_M3_SLMQHLIGL
KD: 1.28e−08
Kass: 1.29e+05, kdiss: 1.64e−03
862_ID.2218_T4_SLLTHLIGL
KD: 1.05e−08
Kass: 1.40e+05, kdiss: 1.47e−03
863_ID.2219_R8_SLLQHLIRL
KD: 1.14e−08
Kass: 1.01e+05, kdiss: 1.16e−03
864_ID.2220_I6_SLLQHIIGL
KD: 1.04e−08
Kass: 1.25e+05, kdiss: 1.30e−03
865_ID.2221_D8_SLLQHLIDL
KD: 7.79e−08
Kass: 8.29e+04, kdiss: 6.46e−03
866_ID.2224_G5_SLLQGLIGL
KD: 6.19e−07
Kass: 7.37e+04, kdiss: 4.57e−02
867_ID.2225_WT_SLLQHLIGL
KD: 1.05e−08
Kass: 1.38e+05, kdiss: 1.45e−03
868_ID.2226_A5_SLLQALIGL
KD: 9.57e−08
Kass: 1.40e+05, kdiss: 1.34e−02
869_ID.2227_Y8_SLLQHLIYL
KD: 1.09e−06
Kass: 3.34e+04, kdiss: 3.63e−02
870_ID.2228_E4_SLLEHLIGL
KD: 1.57e−08
Kass: 8.24e+04, kdiss: 1.29e−03
871_ID.2229_F7_SLLQHLFGL
KD: 4.84e−08
Kass: 1.47e+05, kdiss: 7.14e−03
872_ID.2231_WT_SLLQHLIGL
KD: 1.47e−08
Kass: 1.39e+05, kdiss: 2.03e−03
873_ID.2232_A6_SLLQHAIGL
KD: 1.11e−08
Kass: 1.38e+05, kdiss: 1.54e−03
874_ID.2233_L8_SLLQHLILL
KD: 6.78e−08
Kass: 6.47e+04, kdiss: 4.39e−03
875_ID.2235_N6_SLLQHNIGL
KD: 1.03e−08
Kass: 1.15e+05, kdiss: 1.19e−03
876_ID.2236_T3_SLTQHLIGL
KD: 1.51e−08
Kass: 1.49e+05, kdiss: 2.25e−03
877_ID.2237_W3_SLWQHLIGL
KD: 1.01e−08
Kass: 1.33e+05, kdiss: 1.34e−03
878_ID.2239_D7_SLLQHLDGL
KD: 4.14e−08
Kass: 3.37e+04, kdiss: 1.39e−03
879_ID.2241_M1_MLLQHLIGL
KD: 1.25e−08
Kass: 1.34e+05, kdiss: 1.68e−03
880_ID.2243_G3_SLGQHLIGL
KD: 1.46e−08
Kass: 1.45e+05, kdiss: 2.11e−03
881_ID.2244_P4_SLLPHLIGL
KD: 1.09e−08
Kass: 1.38e+05, kdiss: 1.51e−03
882_ID.2245_Q8_SLLQHLIQL
KD: 3.08e−08
Kass: 7.83e+04, kdiss: 2.41e−03
883_ID.2246_Q5_SLLQQLIGL
KD: 2.30e−07
Kass: 1.07e+05, kdiss: 2.46e−02
884_ID.2249_K8_SLLQHLIKL
KD: 1.63e−07
Kass: 6.36e+04, kdiss: 1.03e−02
885_ID.2250_A4_SLLAHLIGL
KD: 9.64e−09
Kass: 1.24e+05, kdiss: 1.20e−03
886_ID.2251_D7_SLLQHLDGL
KD: 3.66e−08
Kass: 5.13e+04, kdiss: 1.88e−03
887_ID.2253_G1_GLLQHLIGL
KD: 8.42e−09
Kass: 1.14e+05, kdiss: 9.63e−04
888_ID.2255_E8_SLLQHLIEL
KD: 2.55e−07
Kass: 5.13e+04, kdiss: 1.31e−02
889_ID.2256_I6_SLLQHIIGL
KD: 1.17e−08
Kass: 9.62e+04, kdiss: 1.13e−03
890_ID.2257_V8_SLLQHLIVL
KD: 4.19e−08
Kass: 6.90e+04, kdiss: 2.90e−03
891_ID.2259_K6_SLLQHKIGL
KD: 5.48e−07
Kass: 3.60e+04, kdiss: 1.97e−02
892_ID.226_N4_SLLNHLIGL
KD: 3.08e−08
Kass: 1.08e+05, kdiss: 3.32e−03
893_ID.2260_E4_SLLEHLIGL
KD: 1.21e−08
Kass: 6.49e+04, kdiss: 7.85e−04
894_ID.2261_E8_SLLQHLIEL
KD: 2.24e−07
Kass: 5.07e+04, kdiss: 1.14e−02
895_ID.2263_E6_SLLQHEIGL
KD: 2.49e−07
Kass: 3.43e+04, kdiss: 8.56e−03
896_ID.2265_M3_SLMQHLIGL
KD: 1.86e−08
Kass: 7.34e+04, kdiss: 1.37e−03
897_ID.2267_V1_VLLQHLIGL
KD: 1.72e−08
Kass: 8.32e+04, kdiss: 1.43e−03
898_ID.2269_H7_SLLQHLHGL
KD:−
Kass:−, , kdiss: 7.55e−01
899_ID.227_M3_SLMQHLIGL
KD: 2.03e−08
Kass: 1.16e+05, kdiss: 2.36e−03
900_ID.2270_K4_SLLKHLIGL
KD: 1.09e−08
Kass: 1.12e+05, kdiss: 1.22e−03
value
Time

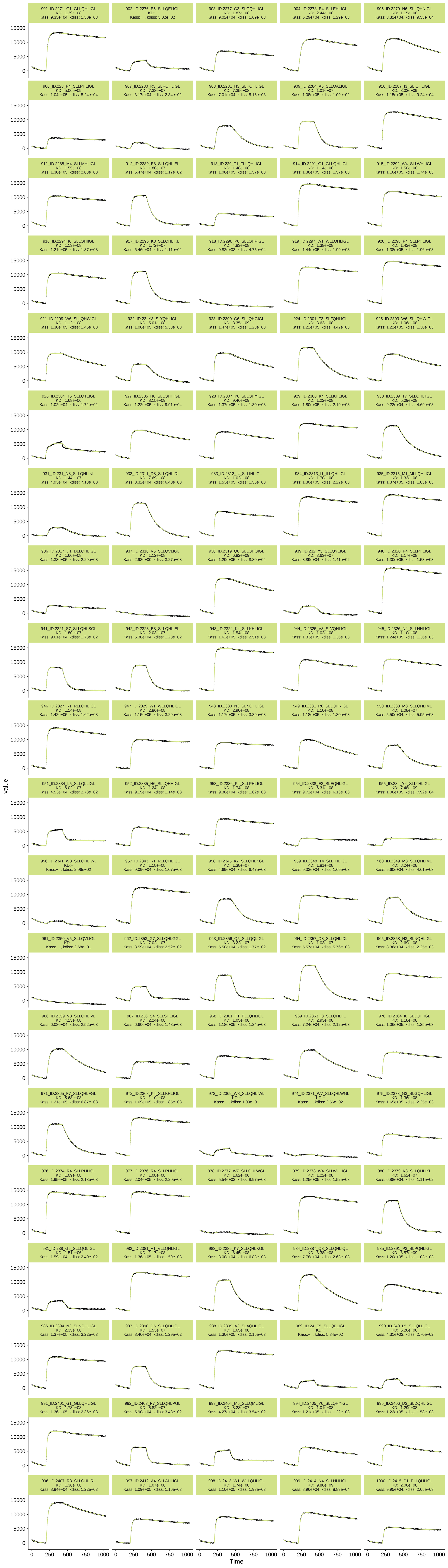

901_ID.2271_G1_LLLQHLIGL KD: 1.39e-08 Kass: 9.33e+04, kdiss: 1.30e-03
902_ID.2276_E5_SLLQELIGL KD:~ Kass:~ , kdiss: 3.02e-02
903_ID.2277_G3_SLGQHLIGL KD: 1.87e-08 Kass: 9.02e+04, kdiss: 1.69e-03
904_ID.2278_E4_SLLEHLIGL KD: 2.44e-08 Kass: 5.29e+04, kdiss: 1.29e-03
905_ID.2279_N6_SLLQHNIGL KD: 1.15e-08 Kass: 8.31e+04, kdiss: 9.53e-04
906_ID.228_P4_SLLPHLIGL KD: 5.06e-09 Kass: 1.04e+05, kdiss: 5.24e-04
907_ID.2280_R3_SLRQHLIGL KD: 7.38e-07 Kass: 3.17e+04, kdiss: 2.34e-02
908_ID.2281_H3_SLHQHLIGL KD: 7.35e-08 Kass: 7.01e+04, kdiss: 5.16e-03
909_ID.2284_A5_SLLQALIGL KD: 1.01e-07 Kass: 1.08e+05, kdiss: 1.09e-02
910_ID.2287_I3_SLIQHLIGL KD: 8.02e-09 Kass: 1.15e+05, kdiss: 9.24e-04
911_ID.2288_M4_SLLMHLIGL KD: 1.55e-08 Kass: 1.30e+05, kdiss: 2.03e-03
912_ID.2289_E8_SLLQHLIEL KD: 1.80e-07 Kass: 6.47e+04, kdiss: 1.17e-02
913_ID.229_T1_TLLQHLIGL KD: 1.48e-08 Kass: 1.06e+05, kdiss: 1.57e-03
914_ID.2291_G1_GLLQHLIGL KD: 1.14e-08 Kass: 1.38e+05, kdiss: 1.57e-03
915_ID.2292_W4_SLLWHLIGL KD: 1.50e-08 Kass: 1.16e+05, kdiss: 1.74e-03
916_ID.2294_I6_SLLQHIIGL KD: 1.13e-08 Kass: 1.21e+05, kdiss: 1.37e-03
917_ID.2295_K8_SLLQHLIKL KD: 1.72e-07 Kass: 6.46e+04, kdiss: 1.11e-02
918_ID.2296_P6_SLLQHPIGL KD: 4.83e-09 Kass: 9.82e+03, kdiss: 4.75e-04
919_ID.2297_W1_WLLQHLIGL KD: 1.38e-08 Kass: 1.44e+05, kdiss: 1.99e-03
920_ID.2298_P4_SLLPHLIGL KD: 1.42e-08 Kass: 1.38e+05, kdiss: 1.96e-03
921_ID.2299_W6_SLLQHWIGL KD: 1.11e-08 Kass: 1.30e+05, kdiss: 1.45e-03
922_ID.23_Y3_SLYQHLIGL KD: 5.01e-08 Kass: 1.06e+05, kdiss: 5.33e-03
923_ID.2300_G6_SLLQHGIGL KD: 8.35e-09 Kass: 1.47e+05, kdiss: 1.23e-03
924_ID.2301_F3_SLFQHLIGL KD: 3.63e-08 Kass: 1.22e+05, kdiss: 4.42e-03
925_ID.2303_W6_SLLQHWIGL KD: 1.06e-08 Kass: 1.22e+05, kdiss: 1.30e-03
926_ID.2304_T5_SLLQTLIGL KD: 1.68e-07 Kass: 1.02e+04, kdiss: 1.72e-02
927_ID.2305_H6_SLLQHHIGL KD: 8.15e-09 Kass: 1.22e+05, kdiss: 9.91e-04
928_ID.2307_Y6_SLLQHYIGL KD: 9.46e-09 Kass: 1.37e+05, kdiss: 1.30e-03
929_ID.2308_K4_SLLKHLIGL KD: 1.22e-08 Kass: 1.80e+05, kdiss: 2.19e-03
930_ID.2309_T7_SLLQHLTGL KD: 5.09e-08 Kass: 9.22e+04, kdiss: 4.69e-03
931_ID.231_N8_SLLQHLINL KD: 1.44e-07 Kass: 4.93e+04, kdiss: 7.13e-03
932_ID.2311_D8_SLLQHLIDL KD: 7.69e-08 Kass: 8.32e+04, kdiss: 6.40e-03
933_ID.2312_I4_SLLIHLIGL KD: 1.02e-08 Kass: 1.53e+05, kdiss: 1.56e-03
934_ID.2313_I1_ILLQHLIGL KD: 1.70e-08 Kass: 1.30e+05, kdiss: 2.22e-03
935_ID.2315_M1_MLLQHLIGL KD: 1.33e-08 Kass: 1.37e+05, kdiss: 1.83e-03
936_ID.2317_D1_DLLQHLIGL KD: 1.66e-08 Kass: 1.38e+05, kdiss: 2.29e-03
937_ID.2318_V5_SLLQVLIGL KD: 1.12e-08 Kass: 2.93e+00, kdiss: 3.27e-08
938_ID.2319_Q6_SLLQHQIGL KD: 6.82e-09 Kass: 1.29e+05, kdiss: 8.80e-04
939_ID.232_Y5_SLLQYLIGL KD: 3.63e-07 Kass: 3.89e+04, kdiss: 1.41e-02
940_ID.2320_P4_SLLPHLIGL KD: 1.17e-08 Kass: 1.30e+05, kdiss: 1.53e-03
941_ID.2321_S7_SLLQHLSGL KD: 1.80e-07 Kass: 9.61e+04, kdiss: 1.73e-02
942_ID.2323_E8_SLLQHLIEL KD: 2.03e-07 Kass: 6.30e+04, kdiss: 1.28e-02
943_ID.2324_K4_SLLKHLIGL KD: 1.54e-08 Kass: 1.62e+05, kdiss: 2.51e-03
944_ID.2325_V3_SLVQHLIGL KD: 1.02e-08 Kass: 1.33e+05, kdiss: 1.36e-03
945_ID.2326_N4_SLLNHLIGL KD: 1.10e-08 Kass: 1.24e+05, kdiss: 1.36e-03
946_ID.2327_R1_RLLQHLIGL KD: 1.14e-08 Kass: 1.42e+05, kdiss: 1.62e-03
947_ID.2329_W1_WLLQHLIGL KD: 2.86e-08 Kass: 1.15e+05, kdiss: 3.29e-03
948_ID.2330_N3_SLNQHLIGL KD: 2.90e-08 Kass: 1.17e+05, kdiss: 3.39e-03
949_ID.2331_R6_SLLQHRIGL KD: 1.10e-08 Kass: 1.18e+05, kdiss: 1.30e-03
950_ID.2333_M8_SLLQHLIML KD: 1.08e-07 Kass: 5.50e+04, kdiss: 5.95e-03
951_ID.2334_L5_SLLQLLIGL KD: 6.02e-07 Kass: 4.53e+04, kdiss: 2.73e-02
952_ID.2335_H6_SLLQHHIGL KD: 1.24e-08 Kass: 9.19e+04, kdiss: 1.14e-03
953_ID.2336_P4_SLLPHLIGL KD: 1.74e-08 Kass: 9.30e+04, kdiss: 1.62e-03
954_ID.2338_E3_SLEQHLIGL KD: 6.31e-08 Kass: 9.71e+04, kdiss: 6.13e-03
955_ID.234_Y4_SLLYHLIGL KD: 7.48e-09 Kass: 1.06e+05, kdiss: 7.92e-04
956_ID.2341_W8_SLLQHLIWL KD:~ Kass:~ , kdiss: 2.96e-02
957_ID.2343_R1_RLLQHLIGL KD: 1.18e-08 Kass: 9.09e+04, kdiss: 1.07e-03
958_ID.2345_K7_SLLQHLKGL KD: 1.38e-07 Kass: 4.69e+04, kdiss: 6.47e-03
959_ID.2348_T4_SLLTHLIGL KD: 1.81e-08 Kass: 9.33e+04, kdiss: 1.69e-03
960_ID.2349_M8_SLLQHLIML KD: 8.24e-08 Kass: 5.60e+04, kdiss: 4.61e-03
961_ID.2350_V5_SLLQVLIGL KD:~ Kass:~ , kdiss: 2.68e-01
962_ID.2353_G7_SLLQHLGGL KD: 7.02e-07 Kass: 3.59e+04, kdiss: 2.52e-02
963_ID.2356_Q5_SLLQQLIGL KD: 3.22e-07 Kass: 5.50e+04, kdiss: 1.77e-02
964_ID.2357_D8_SLLQHLIDL KD: 1.03e-07 Kass: 5.57e+04, kdiss: 5.76e-03
965_ID.2358_N3_SLNQHLIGL KD: 2.69e-08 Kass: 8.36e+04, kdiss: 2.25e-03
966_ID.2359_V8_SLLQHLIVL KD: 4.15e-07 Kass: 6.08e+04, kdiss: 2.52e-02
967_ID.236_S4_SLLSHLIGL KD: 2.24e-08 Kass: 6.60e+04, kdiss: 1.48e-03
968_ID.2361_P1_PLLQHLIGL KD: 1.05e-08 Kass: 1.18e+05, kdiss: 1.24e-03
969_ID.2363_I8_SLLQHLIIL KD: 2.93e-08 Kass: 7.24e+04, kdiss: 2.12e-03
970_ID.2364_I6_SLLQHIIGL KD: 1.18e-08 Kass: 1.06e+05, kdiss: 1.25e-03
971_ID.2365_F7_SLLQHLFGL KD: 5.68e-08 Kass: 1.21e+05, kdiss: 6.87e-03
972_ID.2368_K4_SLLKHLIGL KD: 1.10e-08 Kass: 1.69e+05, kdiss: 1.85e-03
973_ID.2369_W8_SLLQHLIWL KD:~ Kass:~ , kdiss: 1.09e-01
974_ID.2371_W7_SLLQHLWGL KD:~ Kass:~ , kdiss: 2.56e-02
975_ID.2373_G3_SLGQHLIGL KD: 1.36e-08 Kass: 1.65e+05, kdiss: 2.25e-03
976_ID.2374_R4_SLLRHLIGL KD: 1.09e-08 Kass: 1.95e+05, kdiss: 2.13e-03
977_ID.2376_R4_SLLRHLIGL KD: 1.08e-08 Kass: 2.04e+05, kdiss: 2.20e-03
978_ID.2377_W7_SLLQHLWGL KD: 1.62e-06 Kass: 5.54e+03, kdiss: 8.97e-03
979_ID.2378_W4_SLLWHLIGL KD: 1.22e-08 Kass: 1.25e+05, kdiss: 1.52e-03
980_ID.2379_K8_SLLQHLIKL KD: 1.62e-07 Kass: 6.88e+04, kdiss: 1.11e-02
981_ID.238_Q5_SLLQQLIGL KD: 1.51e-06 Kass: 1.59e+04, kdiss: 2.40e-02
982_ID.2381_V1_VLLQHLIGL KD: 1.17e-08 Kass: 1.36e+05, kdiss: 1.59e-03
983_ID.2385_K7_SLLQHLKGL KD: 8.45e-08 Kass: 8.08e+04, kdiss: 6.83e-03
984_ID.2387_Q8_SLLQHLIQL KD: 3.38e-08 Kass: 7.78e+04, kdiss: 2.63e-03
985_ID.2391_P3_SLPQHLIGL KD: 8.57e-09 Kass: 1.20e+05, kdiss: 1.03e-03
986_ID.2394_N3_SLNQHLIGL KD: 2.35e-08 Kass: 1.37e+05, kdiss: 3.22e-03
987_ID.2398_D5_SLLQDLIGL KD: 1.53e-07 Kass: 8.46e+04, kdiss: 1.29e-02
988_ID.2399_A3_SLAQHLIGL KD: 1.65e-08 Kass: 1.30e+05, kdiss: 2.15e-03
989_ID.24_E5_SLLQELIGL KD:~ Kass:~ , kdiss: 5.84e-02
990_ID.240_L5_SLLQLLIGL KD: 6.26e-06 Kass: 4.31e+03, kdiss: 2.70e-02
991_ID.2401_G1_GLLQHLIGL KD: 1.73e-08 Kass: 1.36e+05, kdiss: 2.36e-03
992_ID.2403_P7_SLLQHLPGL KD: 5.82e-07 Kass: 5.90e+04, kdiss: 3.43e-02
993_ID.2404_M5_SLLQMLIGL KD: 8.28e-07 Kass: 4.27e+04, kdiss: 3.54e-02
994_ID.2405_Y6_SLLQHYIGL KD: 1.01e-08 Kass: 1.21e+05, kdiss: 1.22e-03
995_ID.2406_D3_SLDQHLIGL KD: 1.29e-08 Kass: 1.22e+05, kdiss: 1.58e-03
996_ID.2407_R8_SLLQHLIRL KD: 1.36e-08 Kass: 8.94e+04, kdiss: 1.22e-03
997_ID.2412_A4_SLLAHLIGL KD: 1.07e-08 Kass: 1.09e+05, kdiss: 1.16e-03
998_ID.2413_W1_WLLQHLIGL KD: 1.74e-08 Kass: 1.10e+05, kdiss: 1.93e-03
999_ID.2414_N4_SLLNHLIGL KD: 9.86e-09 Kass: 8.96e+04, kdiss: 8.83e-04
1000_ID.2415_P1_PLLQHLIGL KD: 2.06e-08 Kass: 9.95e+04, kdiss: 2.05e-03
value
Time
0
5000
10000
15000
0
250
500
750
1000

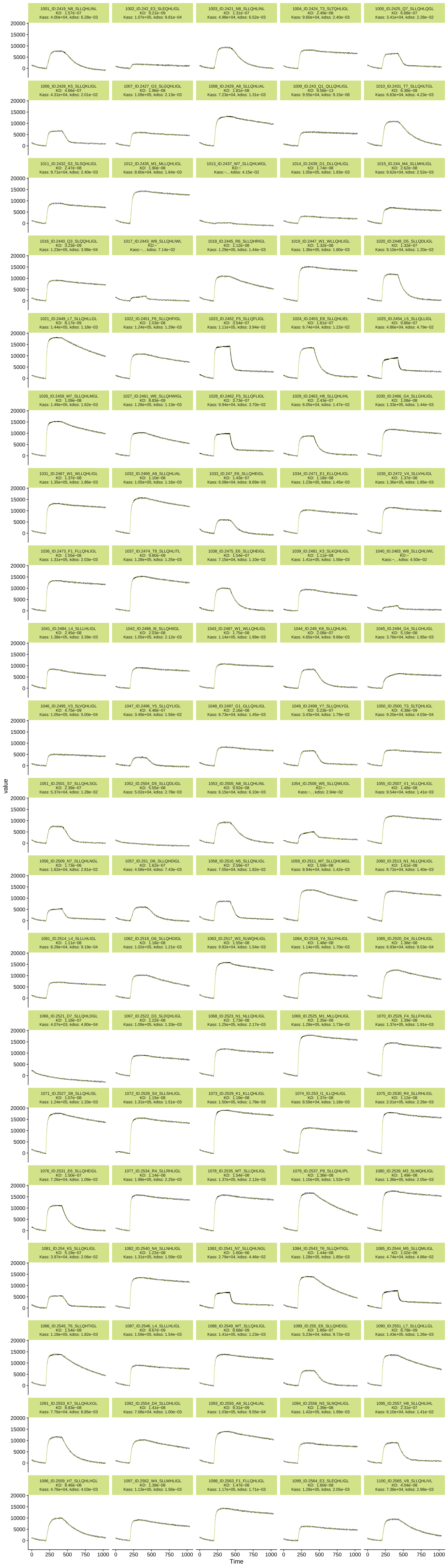
1001_ID.2419_N8_SLLQHLINL
KD: 1.57e−07
Kass: 4.00e+04, kdiss: 6.28e−03
1002_ID.242_E3_SLEQHLIGL
KD: 9.21e−09
Kass: 1.07e+05, kdiss: 9.81e−04
1003_ID.2421_N8_SLLQHLINL
KD: 1.31e−07
Kass: 4.98e+04, kdiss: 6.52e−03
1004_ID.2424_T3_SLTQHLIGL
KD: 2.49e−08
Kass: 9.60e+04, kdiss: 2.40e−03
1005_ID.2425_Q7_SLLQHLQGL
KD: 6.68e−07
Kass: 3.41e+04, kdiss: 2.28e−02
1006_ID.2426_K5_SLLQKLIGL
KD: 4.66e−07
Kass: 4.31e+04, kdiss: 2.01e−02
1007_ID.2427_G3_SLGQHLIGL
KD: 1.96e−08
Kass: 1.09e+05, kdiss: 2.13e−03
1008_ID.2429_A8_SLLQHLIAL
KD: 1.81e−08
Kass: 7.23e+04, kdiss: 1.31e−03
1009_ID.243_Q1_QLLQHLIGL
KD: 9.58e−13
Kass: 9.55e+04, kdiss: 9.15e−08
1010_ID.2431_T7_SLLQHLTGL
KD: 6.38e−08
Kass: 6.63e+04, kdiss: 4.23e−03
1011_ID.2432_S3_SLSQHLIGL
KD: 2.47e−08
Kass: 9.71e+04, kdiss: 2.40e−03
1012_ID.2435_M1_MLLQHLIGL
KD: 1.90e−08
Kass: 8.60e+04, kdiss: 1.64e−03
1013_ID.2437_W7_SLLQHLWGL
KD:−
Kass:−, , kdiss: 4.15e−02
1014_ID.2439_D1_DLLQHLIGL
KD: 1.74e−08
Kass: 1.05e+05, kdiss: 1.83e−03
1015_ID.244_M4_SLLMHLIGL
KD: 2.62e−08
Kass: 9.62e+04, kdiss: 2.52e−03
1016_ID.2440_Q3_SLQQHLIGL
KD: 3.23e−09
Kass: 1.23e+05, kdiss: 3.98e−04
1017_ID.2443_W8_SLLQHLIWL
KD:−
Kass:−, , kdiss: 7.14e−02
1018_ID.2445_R6_SLLQHRIGL
KD: 1.12e−08
Kass: 1.29e+05, kdiss: 1.44e−03
1019_ID.2447_W1_WLLQHLIGL
KD: 1.32e−08
Kass: 1.36e+05, kdiss: 1.80e−03
1020_ID.2448_D5_SLLQDLIGL
KD: 1.32e−07
Kass: 9.10e+04, kdiss: 1.20e−02
1021_ID.2449_L7_SLLQHLLGL
KD: 8.17e−09
Kass: 1.44e+05, kdiss: 1.18e−03
1022_ID.2451_F6_SLLQHFIGL
KD: 1.03e−08
Kass: 1.24e+05, kdiss: 1.29e−03
1023_ID.2452_F5_SLLQFLIGL
KD: 3.54e−07
Kass: 1.11e+05, kdiss: 3.94e−02
1024_ID.2453_E8_SLLQHLIEL
KD: 1.81e−07
Kass: 6.74e+04, kdiss: 1.22e−02
1025_ID.2454_L5_SLLQLLIGL
KD: 9.86e−07
Kass: 4.86e+04, kdiss: 4.79e−02
1026_ID.2459_M7_SLLQHLMGL
KD: 1.09e−08
Kass: 1.49e+05, kdiss: 1.62e−03
1027_ID.2461_W6_SLLQHWIGL
KD: 8.83e−09
Kass: 1.28e+05, kdiss: 1.13e−03
1028_ID.2462_F5_SLLQFLIGL
KD: 3.73e−07
Kass: 9.94e+04, kdiss: 3.70e−02
1029_ID.2463_H8_SLLQHLIHL
KD: 2.43e−07
Kass: 6.05e+04, kdiss: 1.47e−02
1030_ID.2466_G4_SLLGHLIGL
KD: 1.08e−08
Kass: 1.33e+05, kdiss: 1.44e−03
1031_ID.2467_W1_WLLQHLIGL
KD: 1.37e−08
Kass: 1.35e+05, kdiss: 1.86e−03
1032_ID.2469_A8_SLLQHLIAL
KD: 1.10e−08
Kass: 1.05e+05, kdiss: 1.16e−03
1033_ID.247_E6_SLLQHEIGL
KD: 1.43e−07
Kass: 6.08e+04, kdiss: 8.69e−03
1034_ID.2471_E1_ELLQHLIGL
KD: 1.18e−08
Kass: 1.23e+05, kdiss: 1.45e−03
1035_ID.2472_V4_SLLVHLIGL
KD: 1.37e−08
Kass: 1.36e+05, kdiss: 1.85e−03
1036_ID.2473_F1_FLLQHLIGL
KD: 1.55e−08
Kass: 1.31e+05, kdiss: 2.03e−03
1037_ID.2474_T8_SLLQHLITL
KD: 9.80e−09
Kass: 1.28e+05, kdiss: 1.25e−03
1038_ID.2475_E6_SLLQHEIGL
KD: 1.54e−07
Kass: 7.15e+04, kdiss: 1.10e−02
1039_ID.2481_K3_SLKQHLIGL
KD: 1.11e−08
Kass: 1.41e+05, kdiss: 1.56e−03
1040_ID.2483_W8_SLLQHLIWL
KD:−
Kass:−, , kdiss: 4.50e−02
1041_ID.2484_L4_SLLLHLIGL
KD: 2.45e−08
Kass: 1.38e+05, kdiss: 3.39e−03
1042_ID.2486_I6_SLLQHIIGL
KD: 2.03e−08
Kass: 1.05e+05, kdiss: 2.12e−03
1043_ID.2487_W1_WLLQHLIGL
KD: 1.75e−08
Kass: 1.14e+05, kdiss: 1.99e−03
1044_ID.249_K8_SLLQHLIKL
KD: 2.08e−07
Kass: 4.65e+04, kdiss: 9.66e−03
1045_ID.2494_G4_SLLGHLIGL
KD: 5.19e−08
Kass: 3.76e+04, kdiss: 1.95e−03
1046_ID.2495_V3_SLVQHLIGL
KD: 4.75e−09
Kass: 1.05e+05, kdiss: 5.00e−04
1047_ID.2496_Y5_SLLQYLIGL
KD: 4.48e−07
Kass: 3.49e+04, kdiss: 1.56e−02
1048_ID.2497_G1_GLLQHLIGL
KD: 2.16e−08
Kass: 6.73e+04, kdiss: 1.45e−03
1049_ID.2499_Y7_SLLQHLYGL
KD: 5.23e−07
Kass: 3.43e+04, kdiss: 1.79e−02
1050_ID.2500_T3_SLTQHLIGL
KD: 4.38e−09
Kass: 9.20e+04, kdiss: 4.03e−04
1051_ID.2501_S7_SLLQHLSGL
KD: 2.39e−07
Kass: 5.37e+04, kdiss: 1.28e−02
1052_ID.2504_D5_SLLQDLIGL
KD: 5.55e−08
Kass: 5.02e+04, kdiss: 2.78e−03
1053_ID.2505_N8_SLLQHLINL
KD: 9.92e−08
Kass: 6.15e+04, kdiss: 6.10e−03
1054_ID.2506_W5_SLLQWLIGL
KD:−
Kass:−, , kdiss: 2.94e−02
1055_ID.2507_V1_VLLQHLIGL
KD: 1.48e−08
Kass: 9.54e+04, kdiss: 1.41e−03
1056_ID.2509_N7_SLLQHLNGL
KD: 1.73e−06
Kass: 1.62e+04, kdiss: 2.81e−02
1057_ID.251_D6_SLLQHDIGL
KD: 1.62e−07
Kass: 4.58e+04, kdiss: 7.43e−03
1058_ID.2510_N5_SLLQNLIGL
KD: 2.59e−07
Kass: 7.05e+04, kdiss: 1.82e−02
1059_ID.2511_M7_SLLQHLMGL
KD: 1.59e−08
Kass: 8.94e+04, kdiss: 1.42e−03
1060_ID.2513_N1_NLLQHLIGL
KD: 1.61e−08
Kass: 8.72e+04, kdiss: 1.40e−03
1061_ID.2514_L4_SLLLHLIGL
KD: 1.11e−08
Kass: 8.29e+04, kdiss: 9.19e−04
1062_ID.2516_G6_SLLQHGIGL
KD: 1.18e−08
Kass: 1.02e+05, kdiss: 1.21e−03
1063_ID.2517_W3_SLWQHLIGL
KD: 1.55e−08
Kass: 9.92e+04, kdiss: 1.54e−03
1064_ID.2518_Y4_SLLYHLIGL
KD: 1.48e−08
Kass: 1.14e+05, kdiss: 1.70e−03
1065_ID.2520_D4_SLLDHLIGL
KD: 1.38e−08
Kass: 6.93e+04, kdiss: 9.53e−04
1066_ID.2521_D7_SLLQHLDGL
KD: 1.18e−07
Kass: 4.07e+03, kdiss: 4.80e−04
1067_ID.2522_D3_SLDQHLIGL
KD: 1.22e−08
Kass: 1.09e+05, kdiss: 1.33e−03
1068_ID.2523_N1_NLLQHLIGL
KD: 1.73e−08
Kass: 1.25e+05, kdiss: 2.17e−03
1069_ID.2525_M1_MLLQHLIGL
KD: 1.35e−08
Kass: 1.28e+05, kdiss: 1.73e−03
1070_ID.2526_F4_SLLFHLIGL
KD: 1.39e−08
Kass: 1.37e+05, kdiss: 1.91e−03
1071_ID.2527_S8_SLLQHLISL
KD: 1.07e−08
Kass: 1.24e+05, kdiss: 1.33e−03
1072_ID.2528_S4_SLLSHLIGL
KD: 1.15e−08
Kass: 1.31e+05, kdiss: 1.51e−03
1073_ID.2529_K1_KLLQHLIGL
KD: 1.19e−08
Kass: 1.50e+05, kdiss: 1.78e−03
1074_ID.253_I1_ILLQHLIGL
KD: 1.37e−08
Kass: 8.59e+04, kdiss: 1.18e−03
1075_ID.2530_R4_SLLRHLIGL
KD: 1.12e−08
Kass: 2.01e+05, kdiss: 2.26e−03
1076_ID.2531_E6_SLLQHEIGL
KD: 1.50e−07
Kass: 7.26e+04, kdiss: 1.09e−02
1077_ID.2534_R4_SLLRHLIGL
KD: 1.14e−08
Kass: 1.98e+05, kdiss: 2.25e−03
1078_ID.2535_WT_SLLQHLIGL
KD: 1.54e−08
Kass: 1.37e+05, kdiss: 2.12e−03
1079_ID.2537_P8_SLLQHLIPL
KD: 1.38e−08
Kass: 1.10e+05, kdiss: 1.52e−03
1080_ID.2539_M3_SLMQHLIGL
KD: 1.49e−08
Kass: 1.38e+05, kdiss: 2.05e−03
1081_ID.254_K5_SLLQKLIGL
KD: 5.19e−07
Kass: 3.97e+04, kdiss: 2.06e−02
1082_ID.2540_N4_SLLNHLIGL
KD: 1.22e−08
Kass: 1.31e+05, kdiss: 1.59e−03
1083_ID.2541_N7_SLLQHLNGL
KD: 1.60e−06
Kass: 2.79e+04, kdiss: 4.46e−02
1084_ID.2543_T6_SLLQHTIGL
KD: 1.44e−08
Kass: 1.28e+05, kdiss: 1.85e−03
1085_ID.2544_M5_SLLQMLIGL
KD: 1.02e−06
Kass: 4.74e+04, kdiss: 4.86e−02
1086_ID.2545_T6_SLLQHTIGL
KD: 1.54e−08
Kass: 1.18e+05, kdiss: 1.82e−03
1087_ID.2546_L4_SLLLHLIGL
KD: 9.67e−09
Kass: 1.59e+05, kdiss: 1.54e−03
1088_ID.2549_WT_SLLQHLIGL
KD: 8.68e−09
Kass: 1.41e+05, kdiss: 1.23e−03
1089_ID.255_E6_SLLQHEIGL
KD: 1.86e−07
Kass: 5.23e+04, kdiss: 9.72e−03
1090_ID.2551_L7_SLLQHLLGL
KD: 8.79e−09
Kass: 1.43e+05, kdiss: 1.26e−03
1091_ID.2553_K7_SLLQHLKGL
KD: 8.83e−08
Kass: 7.76e+04, kdiss: 6.85e−03
1092_ID.2554_D4_SLLDHLIGL
KD: 1.41e−08
Kass: 7.08e+04, kdiss: 1.00e−03
1093_ID.2555_A8_SLLQHLIAL
KD: 9.31e−09
Kass: 1.03e+05, kdiss: 9.55e−04
1094_ID.2556_N3_SLNQHLIGL
KD: 1.39e−08
Kass: 1.42e+05, kdiss: 1.99e−03
1095_ID.2557_H8_SLLQHLIHL
KD: 2.31e−07
Kass: 6.10e+04, kdiss: 1.41e−02
1096_ID.2559_H7_SLLQHLHGL
KD: 8.46e−08
Kass: 4.76e+04, kdiss: 4.03e−03
1097_ID.2562_W4_SLLWHLIGL
KD: 1.39e−08
Kass: 1.13e+05, kdiss: 1.56e−03
1098_ID.2563_F1_FLLQHLIGL
KD: 1.47e−08
Kass: 1.17e+05, kdiss: 1.71e−03
1099_ID.2564_E3_SLEQHLIGL
KD: 1.60e−08
Kass: 1.28e+05, kdiss: 2.05e−03
1100_ID.2565_V8_SLLQHLIVL
KD: 4.04e−08
Kass: 7.38e+04, kdiss: 2.98e−03
value
20000
15000
10000
5000
0
0
250
500
750
1000
Time

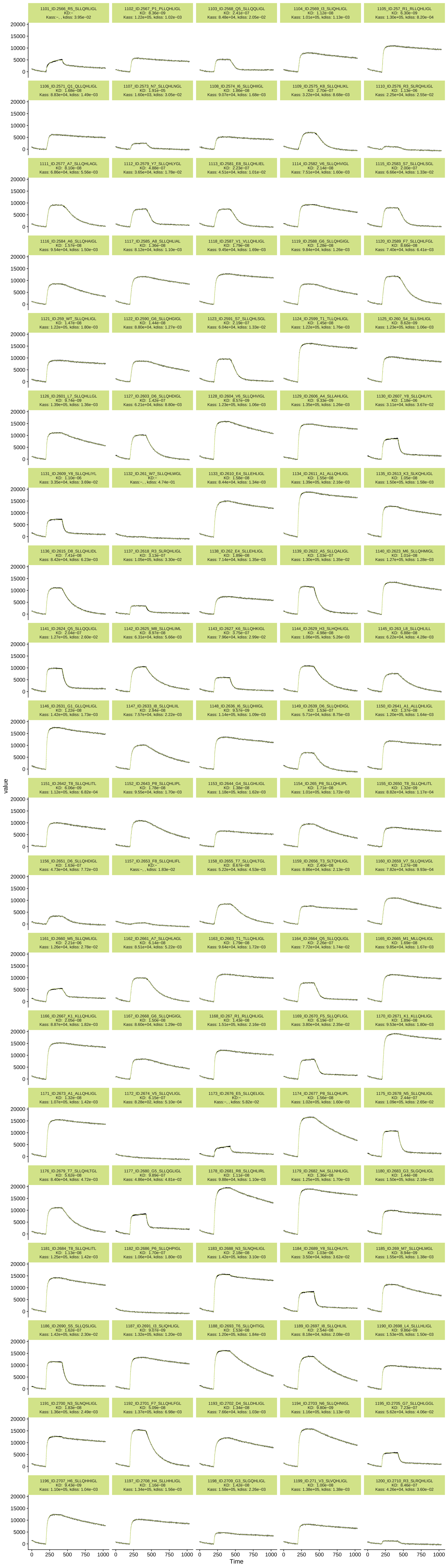
1101_ID.2566_R5_SLLQRLIGL
KD:–
Kass:–, , kdiss: 3.95e–02
1102_ID.2567_P1_PLLQHLIGL
KD: 8.36e–09
Kass: 1.22e+05, kdiss: 1.02e–03
1103_ID.2568_Q5_SLLQQLIGL
KD: 2.41e–07
Kass: 8.48e+04, kdiss: 2.05e–02
1104_ID.2569_I3_SLIQHLIGL
KD: 1.12e–08
Kass: 1.01e+05, kdiss: 1.13e–03
1105_ID.257_R1_RLLQHLIGL
KD: 6.30e–09
Kass: 1.30e+05, kdiss: 8.20e–04
1106_ID.2571_Q1_QLLQHLIGL
KD: 1.68e–08
Kass: 8.83e+04, kdiss: 1.49e–03
1107_ID.2573_N7_SLLQHLNGL
KD: 1.91e–05
Kass: 1.60e+03, kdiss: 3.05e–02
1108_ID.2574_I6_SLLQHIIGL
KD: 1.86e–08
Kass: 9.07e+04, kdiss: 1.68e–03
1109_ID.2575_K8_SLLQHLIKL
KD: 2.70e–07
Kass: 3.22e+04, kdiss: 8.68e–03
1110_ID.2576_R3_SLRQHLIGL
KD: 1.13e–06
Kass: 2.25e+04, kdiss: 2.55e–02
1111_ID.2577_A7_SLLQHLAGL
KD: 8.10e–08
Kass: 6.86e+04, kdiss: 5.56e–03
1112_ID.2579_Y7_SLLQHLYGL
KD: 4.88e–07
Kass: 3.65e+04, kdiss: 1.78e–02
1113_ID.2581_E8_SLLQHLIEL
KD: 2.23e–07
Kass: 4.51e+04, kdiss: 1.01e–02
1114_ID.2582_V6_SLLQHVIGL
KD: 2.14e–08
Kass: 7.51e+04, kdiss: 1.60e–03
1115_ID.2583_S7_SLLQHLSGL
KD: 2.00e–07
Kass: 6.66e+04, kdiss: 1.33e–02
1116_ID.2584_A6_SLLQHAIGL
KD: 1.57e–08
Kass: 9.54e+04, kdiss: 1.50e–03
1117_ID.2585_A8_SLLQHLIAL
KD: 1.36e–08
Kass: 8.12e+04, kdiss: 1.10e–03
1118_ID.2587_V1_VLLQHLIGL
KD: 1.79e–08
Kass: 9.45e+04, kdiss: 1.69e–03
1119_ID.2588_G6_SLLQHGIGL
KD: 1.28e–08
Kass: 9.84e+04, kdiss: 1.26e–03
1120_ID.2589_F7_SLLQHLFGL
KD: 8.66e–08
Kass: 7.40e+04, kdiss: 6.41e–03
1121_ID.259_WT_SLLQHLIGL
KD: 1.47e–08
Kass: 1.22e+05, kdiss: 1.80e–03
1122_ID.2590_G6_SLLQHGIGL
KD: 1.44e–08
Kass: 8.80e+04, kdiss: 1.27e–03
1123_ID.2591_S7_SLLQHLSGL
KD: 2.19e–07
Kass: 6.04e+04, kdiss: 1.33e–02
1124_ID.2599_T1_TLLQHLIGL
KD: 1.45e–08
Kass: 1.22e+05, kdiss: 1.76e–03
1125_ID.260_S4_SLLSHLIGL
KD: 8.62e–09
Kass: 1.23e+05, kdiss: 1.06e–03
1126_ID.2601_L7_SLLQHLLGL
KD: 9.74e–09
Kass: 1.39e+05, kdiss: 1.36e–03
1127_ID.2603_D6_SLLQHDIGL
KD: 1.42e–07
Kass: 6.21e+04, kdiss: 8.80e–03
1128_ID.2604_V6_SLLQHVIGL
KD: 8.57e–09
Kass: 1.23e+05, kdiss: 1.06e–03
1129_ID.2606_A4_SLLAHLIGL
KD: 9.33e–09
Kass: 1.35e+05, kdiss: 1.26e–03
1130_ID.2607_Y8_SLLQHLIYL
KD: 1.18e–06
Kass: 3.11e+04, kdiss: 3.67e–02
1131_ID.2609_Y8_SLLQHLIYL
KD: 1.10e–06
Kass: 3.35e+04, kdiss: 3.69e–02
1132_ID.261_W7_SLLQHLWGL
KD:–
Kass:–, , kdiss: 4.74e–01
1133_ID.2610_E4_SLLEHLIGL
KD: 1.58e–08
Kass: 8.44e+04, kdiss: 1.34e–03
1134_ID.2611_A1_ALLQHLIGL
KD: 1.55e–08
Kass: 1.39e+05, kdiss: 2.16e–03
1135_ID.2613_K3_SLKQHLIGL
KD: 1.05e–08
Kass: 1.50e+05, kdiss: 1.58e–03
1136_ID.2615_D8_SLLQHLIDL
KD: 7.41e–08
Kass: 8.42e+04, kdiss: 6.23e–03
1137_ID.2618_R3_SLRQHLIGL
KD: 3.13e–07
Kass: 1.05e+05, kdiss: 3.30e–02
1138_ID.262_E4_SLLEHLIGL
KD: 1.89e–08
Kass: 7.14e+04, kdiss: 1.35e–03
1139_ID.2622_A5_SLLQALIGL
KD: 1.03e–07
Kass: 1.30e+05, kdiss: 1.35e–02
1140_ID.2623_M6_SLLQHMIGL
KD: 1.01e–08
Kass: 1.27e+05, kdiss: 1.28e–03
1141_ID.2624_Q5_SLLQQLIGL
KD: 2.04e–07
Kass: 1.27e+05, kdiss: 2.60e–02
1142_ID.2625_M8_SLLQHLIML
KD: 8.97e–08
Kass: 6.31e+04, kdiss: 5.66e–03
1143_ID.2627_K6_SLLQHKIGL
KD: 3.75e–07
Kass: 7.96e+04, kdiss: 2.99e–02
1144_ID.2629_H3_SLHQHLIGL
KD: 4.98e–08
Kass: 1.06e+05, kdiss: 5.26e–03
1145_ID.263_L8_SLLQHLILL
KD: 6.88e–08
Kass: 6.22e+04, kdiss: 4.28e–03
1146_ID.2631_G1_GLLQHLIGL
KD: 1.22e–08
Kass: 1.42e+05, kdiss: 1.73e–03
1147_ID.2633_I8_SLLQHLIIL
KD: 2.94e–08
Kass: 7.57e+04, kdiss: 2.22e–03
1148_ID.2636_I6_SLLQHIIGL
KD: 9.57e–09
Kass: 1.14e+05, kdiss: 1.09e–03
1149_ID.2639_D6_SLLQHDIGL
KD: 1.53e–07
Kass: 5.71e+04, kdiss: 8.75e–03
1150_ID.2641_A1_ALLQHLIGL
KD: 1.37e–08
Kass: 1.20e+05, kdiss: 1.64e–03
1151_ID.2642_T8_SLLQHLITL
KD: 6.06e–09
Kass: 1.12e+05, kdiss: 6.82e–04
1152_ID.2643_P8_SLLQHLIPL
KD: 1.78e–08
Kass: 9.55e+04, kdiss: 1.70e–03
1153_ID.2644_G4_SLLGHLIGL
KD: 1.38e–08
Kass: 1.18e+05, kdiss: 1.62e–03
1154_ID.265_P8_SLLQHLIPL
KD: 1.71e–08
Kass: 1.01e+05, kdiss: 1.72e–03
1155_ID.2650_T8_SLLQHLITL
KD: 1.32e–09
Kass: 8.82e+04, kdiss: 1.17e–04
1156_ID.2651_D6_SLLQHDIGL
KD: 1.63e–07
Kass: 4.73e+04, kdiss: 7.72e–03
1157_ID.2653_F8_SLLQHLIFL
KD:–
Kass:–, , kdiss: 1.83e–02
1158_ID.2655_T7_SLLQHLTGL
KD: 8.67e–08
Kass: 5.22e+04, kdiss: 4.53e–03
1159_ID.2656_T3_SLTQHLIGL
KD: 2.40e–08
Kass: 8.86e+04, kdiss: 2.13e–03
1160_ID.2659_V7_SLLQHLVGL
KD: 1.27e–08
Kass: 7.82e+04, kdiss: 9.93e–04
1161_ID.2660_M5_SLLQMLIGL
KD: 2.21e–06
Kass: 1.26e+04, kdiss: 2.78e–02
1162_ID.2661_A7_SLLQHLAGL
KD: 6.14e–08
Kass: 8.51e+04, kdiss: 5.22e–03
1163_ID.2663_T1_TLLQHLIGL
KD: 1.79e–08
Kass: 9.64e+04, kdiss: 1.72e–03
1164_ID.2664_Q5_SLLQQLIGL
KD: 2.26e–07
Kass: 7.72e+04, kdiss: 1.74e–02
1165_ID.2665_M1_MLLQHLIGL
KD: 1.69e–08
Kass: 9.85e+04, kdiss: 1.67e–03
1166_ID.2667_K1_KLLQHLIGL
KD: 2.05e–08
Kass: 8.87e+04, kdiss: 1.82e–03
1167_ID.2668_G6_SLLQHGIGL
KD: 1.50e–08
Kass: 8.60e+04, kdiss: 1.29e–03
1168_ID.267_R1_RLLQHLIGL
KD: 1.43e–08
Kass: 1.51e+05, kdiss: 2.16e–03
1169_ID.2670_F5_SLLQFLIGL
KD: 6.19e–07
Kass: 3.80e+04, kdiss: 2.35e–02
1170_ID.2671_K1_KLLQHLIGL
KD: 1.89e–08
Kass: 9.53e+04, kdiss: 1.80e–03
1171_ID.2673_A1_ALLQHLIGL
KD: 1.32e–08
Kass: 1.07e+05, kdiss: 1.42e–03
1172_ID.2674_V5_SLLQVLIGL
KD: 6.15e–07
Kass: 8.28e+02, kdiss: 5.10e–04
1173_ID.2676_E5_SLLQELIGL
KD:–
Kass:–, , kdiss: 5.82e–02
1174_ID.2677_P8_SLLQHLIPL
KD: 1.56e–08
Kass: 1.02e+05, kdiss: 1.60e–03
1175_ID.2678_N5_SLLQNLIGL
KD: 2.44e–07
Kass: 1.09e+05, kdiss: 2.65e–02
1176_ID.2679_T7_SLLQHLTGL
KD: 5.62e–08
Kass: 8.40e+04, kdiss: 4.72e–03
1177_ID.2680_G5_SLLQGLIGL
KD: 9.89e–07
Kass: 4.86e+04, kdiss: 4.81e–02
1178_ID.2681_R8_SLLQHLIRL
KD: 1.11e–08
Kass: 9.88e+04, kdiss: 1.10e–03
1179_ID.2682_N4_SLLNHLIGL
KD: 1.36e–08
Kass: 1.25e+05, kdiss: 1.70e–03
1180_ID.2683_G3_SLGQHLIGL
KD: 1.44e–08
Kass: 1.50e+05, kdiss: 2.16e–03
1181_ID.2684_T8_SLLQHLITL
KD: 1.13e–08
Kass: 1.25e+05, kdiss: 1.42e–03
1182_ID.2686_P6_SLLQHPIGL
KD: 1.70e–07
Kass: 1.06e+04, kdiss: 1.80e–03
1183_ID.2688_N3_SLNQHLIGL
KD: 2.18e–08
Kass: 1.42e+05, kdiss: 3.10e–03
1184_ID.2689_Y8_SLLQHLIYL
KD: 1.03e–06
Kass: 3.50e+04, kdiss: 3.62e–02
1185_ID.269_M7_SLLQHLMGL
KD: 8.94e–09
Kass: 1.55e+05, kdiss: 1.38e–03
1186_ID.2690_S5_SLLQSLIGL
KD: 1.62e–07
Kass: 1.42e+05, kdiss: 2.30e–02
1187_ID.2691_I3_SLIQHLIGL
KD: 9.07e–09
Kass: 1.32e+05, kdiss: 1.20e–03
1188_ID.2693_T6_SLLQHTIGL
KD: 1.53e–08
Kass: 1.20e+05, kdiss: 1.84e–03
1189_ID.2697_I8_SLLQHLIIL
KD: 2.54e–08
Kass: 8.18e+04, kdiss: 2.08e–03
1190_ID.2698_L4_SLLLHLIGL
KD: 9.86e–09
Kass: 1.53e+05, kdiss: 1.50e–03
1191_ID.2700_N3_SLNQHLIGL
KD: 1.83e–08
Kass: 1.36e+05, kdiss: 2.49e–03
1192_ID.2701_F7_SLLQHLFGL
KD: 5.09e–08
Kass: 1.37e+05, kdiss: 6.98e–03
1193_ID.2702_D4_SLLDHLIGL
KD: 1.34e–08
Kass: 7.66e+04, kdiss: 1.03e–03
1194_ID.2703_N6_SLLQHNIGL
KD: 9.80e–09
Kass: 1.16e+05, kdiss: 1.13e–03
1195_ID.2705_G7_SLLQHLGGL
KD: 7.23e–07
Kass: 5.62e+04, kdiss: 4.06e–02
1196_ID.2707_H6_SLLQHHIGL
KD: 9.43e–09
Kass: 1.10e+05, kdiss: 1.04e–03
1197_ID.2708_H4_SLLHHLIGL
KD: 1.16e–08
Kass: 1.34e+05, kdiss: 1.56e–03
1198_ID.2709_G3_SLGQHLIGL
KD: 1.42e–08
Kass: 1.58e+05, kdiss: 2.26e–03
1199_ID.271_V3_SLVQHLIGL
KD: 1.00e–08
Kass: 1.38e+05, kdiss: 1.38e–03
1200_ID.2710_R3_SLRQHLIGL
KD: 8.46e–07
Kass: 4.26e+04, kdiss: 3.60e–02
value
0
5000
10000
15000
20000
Time
0
250
500
750
1000

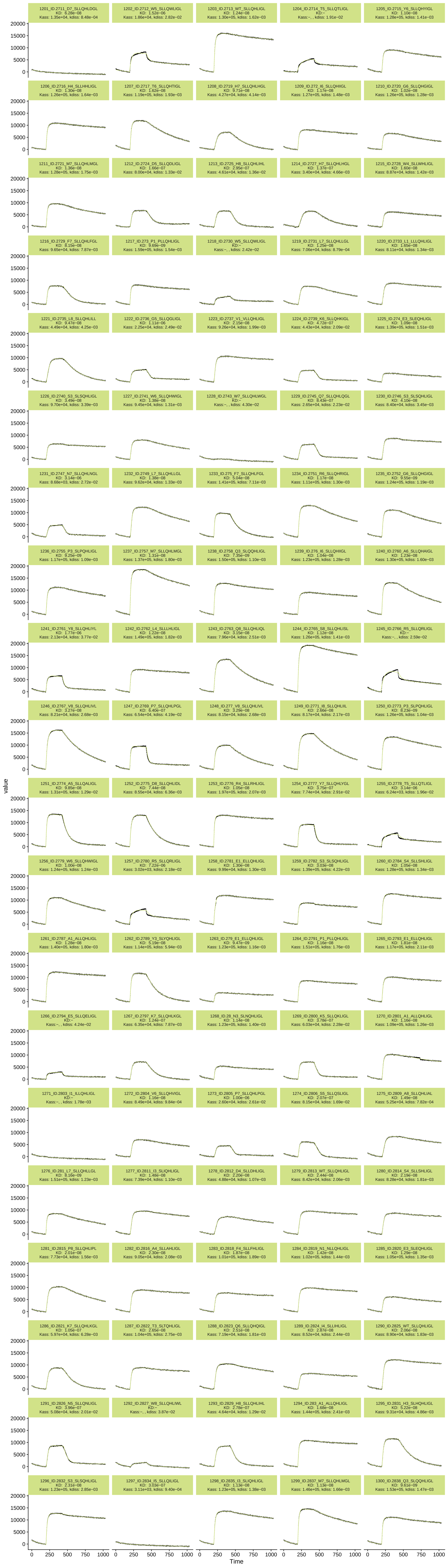
1201_ID.2711_D7_SLLQHLDGL
KD: 6.28e−08
Kass: 1.35e+04, kdiss: 8.48e−04
1202_ID.2712_W5_SLLQWLIGL
KD: 1.52e−06
Kass: 1.86e+04, kdiss: 2.82e−02
1203_ID.2713_WT_SLLQHLIGL
KD: 1.24e−08
Kass: 1.30e+05, kdiss: 1.62e−03
1204_ID.2714_T5_SLLQTLIGL
KD:−
Kass:−, , kdiss: 1.91e−02
1205_ID.2715_Y6_SLLQHYIGL
KD: 1.10e−08
Kass: 1.28e+05, kdiss: 1.41e−03
1206_ID.2716_H4_SLLHHLIGL
KD: 1.30e−08
Kass: 1.26e+05, kdiss: 1.64e−03
1207_ID.2717_T6_SLLQHTIGL
KD: 1.62e−08
Kass: 1.19e+05, kdiss: 1.93e−03
1208_ID.2719_H7_SLLQHLHGL
KD: 9.71e−08
Kass: 4.27e+04, kdiss: 4.14e−03
1209_ID.272_I6_SLLQHIIGL
KD: 1.17e−08
Kass: 1.27e+05, kdiss: 1.48e−03
1210_ID.2720_G6_SLLQHGIGL
KD: 1.02e−08
Kass: 1.26e+05, kdiss: 1.28e−03
1211_ID.2721_M7_SLLQHLMGL
KD: 1.36e−08
Kass: 1.28e+05, kdiss: 1.75e−03
1212_ID.2724_D5_SLLQDLIGL
KD: 1.66e−07
Kass: 8.00e+04, kdiss: 1.33e−02
1213_ID.2725_H8_SLLQHLIHL
KD: 2.95e−07
Kass: 4.61e+04, kdiss: 1.36e−02
1214_ID.2727_H7_SLLQHLHGL
KD: 1.37e−07
Kass: 3.40e+04, kdiss: 4.66e−03
1215_ID.2728_W4_SLLWHLIGL
KD: 1.60e−08
Kass: 8.87e+04, kdiss: 1.42e−03
1216_ID.2729_F7_SLLQHLFGL
KD: 8.15e−08
Kass: 9.65e+04, kdiss: 7.87e−03
1217_ID.273_P1_PLLQHLIGL
KD: 9.69e−09
Kass: 1.59e+05, kdiss: 1.54e−03
1218_ID.2730_W5_SLLQWLIGL
KD:−
Kass:−, , kdiss: 2.42e−02
1219_ID.2731_L7_SLLQHLLGL
KD: 1.25e−08
Kass: 7.06e+04, kdiss: 8.79e−04
1220_ID.2733_L1_LLLQHLIGL
KD: 1.65e−08
Kass: 8.11e+04, kdiss: 1.34e−03
1221_ID.2735_L8_SLLQHLILL
KD: 9.47e−08
Kass: 4.49e+04, kdiss: 4.25e−03
1222_ID.2736_G5_SLLQGLIGL
KD: 1.11e−06
Kass: 2.25e+04, kdiss: 2.49e−02
1223_ID.2737_V1_VLLQHLIGL
KD: 2.15e−08
Kass: 9.26e+04, kdiss: 1.99e−03
1224_ID.2739_K6_SLLQHKIGL
KD: 4.72e−07
Kass: 4.43e+04, kdiss: 2.09e−02
1225_ID.274_E3_SLEQHLIGL
KD: 1.09e−08
Kass: 1.39e+05, kdiss: 1.51e−03
1226_ID.2740_S3_SLSQHLIGL
KD: 3.49e−08
Kass: 9.70e+04, kdiss: 3.39e−03
1227_ID.2741_W6_SLLQHWIGL
KD: 1.38e−08
Kass: 9.45e+04, kdiss: 1.31e−03
1228_ID.2743_W7_SLLQHLWGL
KD:−
Kass:−, , kdiss: 4.30e−02
1229_ID.2745_Q7_SLLQHLQGL
KD: 8.43e−07
Kass: 2.65e+04, kdiss: 2.23e−02
1230_ID.2746_S3_SLSQHLIGL
KD: 4.10e−08
Kass: 8.40e+04, kdiss: 3.45e−03
1231_ID.2747_N7_SLLQHLNGL
KD: 3.14e−06
Kass: 8.68e+03, kdiss: 2.72e−02
1232_ID.2749_L7_SLLQHLLGL
KD: 1.38e−08
Kass: 9.62e+04, kdiss: 1.33e−03
1233_ID.275_F7_SLLQHLFGL
KD: 5.04e−08
Kass: 1.41e+05, kdiss: 7.11e−03
1234_ID.2751_R6_SLLQHRIGL
KD: 1.17e−08
Kass: 1.11e+05, kdiss: 1.30e−03
1235_ID.2752_G6_SLLQHGIGL
KD: 9.55e−09
Kass: 1.24e+05, kdiss: 1.19e−03
1236_ID.2755_P3_SLPQHLIGL
KD: 9.25e−09
Kass: 1.17e+05, kdiss: 1.09e−03
1237_ID.2757_M7_SLLQHLMGL
KD: 1.31e−08
Kass: 1.37e+05, kdiss: 1.80e−03
1238_ID.2758_Q3_SLQQHLIGL
KD: 7.35e−09
Kass: 1.50e+05, kdiss: 1.10e−03
1239_ID.276_I6_SLLQHIIGL
KD: 1.04e−08
Kass: 1.23e+05, kdiss: 1.28e−03
1240_ID.2760_A6_SLLQHAIGL
KD: 1.23e−08
Kass: 1.30e+05, kdiss: 1.60e−03
1241_ID.2761_Y8_SLLQHLIYL
KD: 1.77e−06
Kass: 2.13e+04, kdiss: 3.77e−02
1242_ID.2762_L4_SLLLHLIGL
KD: 1.22e−08
Kass: 1.49e+05, kdiss: 1.82e−03
1243_ID.2763_Q8_SLLQHLIQL
KD: 3.15e−08
Kass: 7.96e+04, kdiss: 2.51e−03
1244_ID.2765_S8_SLLQHLISL
KD: 1.12e−08
Kass: 1.26e+05, kdiss: 1.41e−03
1245_ID.2766_R5_SLLQRLIGL
KD:−
Kass:−, , kdiss: 2.59e−02
1246_ID.2767_V8_SLLQHLIVL
KD: 3.27e−08
Kass: 8.21e+04, kdiss: 2.68e−03
1247_ID.2769_P7_SLLQHLPGL
KD: 6.40e−07
Kass: 6.54e+04, kdiss: 4.19e−02
1248_ID.277_V8_SLLQHLIVL
KD: 3.29e−08
Kass: 8.15e+04, kdiss: 2.68e−03
1249_ID.2771_I8_SLLQHLIIL
KD: 2.66e−08
Kass: 8.17e+04, kdiss: 2.17e−03
1250_ID.2773_P3_SLPQHLIGL
KD: 8.23e−09
Kass: 1.26e+05, kdiss: 1.04e−03
1251_ID.2774_A5_SLLQALIGL
KD: 9.85e−08
Kass: 1.31e+05, kdiss: 1.29e−02
1252_ID.2775_D8_SLLQHLIDL
KD: 7.44e−08
Kass: 8.55e+04, kdiss: 6.36e−03
1253_ID.2776_R4_SLLRHLIGL
KD: 1.05e−08
Kass: 1.97e+05, kdiss: 2.07e−03
1254_ID.2777_Y7_SLLQHLYGL
KD: 3.75e−07
Kass: 7.74e+04, kdiss: 2.91e−02
1255_ID.2778_T5_SLLQTLIGL
KD: 3.14e−06
Kass: 6.24e+03, kdiss: 1.96e−02
1256_ID.2779_W6_SLLQHWIGL
KD: 1.00e−08
Kass: 1.24e+05, kdiss: 1.24e−03
1257_ID.2780_R5_SLLQRLIGL
KD: 7.22e−06
Kass: 3.02e+03, kdiss: 2.18e−02
1258_ID.2781_E1_ELLQHLIGL
KD: 1.30e−08
Kass: 9.99e+04, kdiss: 1.30e−03
1259_ID.2782_S3_SLSQHLIGL
KD: 3.03e−08
Kass: 1.39e+05, kdiss: 4.22e−03
1260_ID.2784_S4_SLLSHLIGL
KD: 1.05e−08
Kass: 1.28e+05, kdiss: 1.34e−03
1261_ID.2787_A1_ALLQHLIGL
KD: 1.28e−08
Kass: 1.40e+05, kdiss: 1.80e−03
1262_ID.2789_Y3_SLYQHLIGL
KD: 5.19e−08
Kass: 1.14e+05, kdiss: 5.94e−03
1263_ID.279_E1_ELLQHLIGL
KD: 9.47e−09
Kass: 1.23e+05, kdiss: 1.16e−03
1264_ID.2791_P1_PLLQHLIGL
KD: 1.16e−08
Kass: 1.51e+05, kdiss: 1.76e−03
1265_ID.2793_E1_ELLQHLIGL
KD: 1.81e−08
Kass: 1.17e+05, kdiss: 2.11e−03
1266_ID.2794_E5_SLLQELIGL
KD:−
Kass:−, , kdiss: 4.24e−02
1267_ID.2797_K7_SLLQHLKGL
KD: 1.24e−07
Kass: 6.35e+04, kdiss: 7.87e−03
1268_ID.28_N3_SLNQHLIGL
KD: 1.14e−08
Kass: 1.23e+05, kdiss: 1.40e−03
1269_ID.2800_K5_SLLQKLIGL
KD: 3.78e−07
Kass: 6.03e+04, kdiss: 2.28e−02
1270_ID.2801_A1_ALLQHLIGL
KD: 1.16e−08
Kass: 1.09e+05, kdiss: 1.26e−03
1271_ID.2803_I1_ILLQHLIGL
KD:−
Kass:−, , kdiss: 1.78e−03
1272_ID.2804_V6_SLLQHVIGL
KD: 1.16e−08
Kass: 8.49e+04, kdiss: 9.84e−04
1273_ID.2805_P7_SLLQHLPGL
KD: 1.00e−06
Kass: 2.60e+04, kdiss: 2.61e−02
1274_ID.2806_S5_SLLQSLIGL
KD: 2.07e−07
Kass: 8.15e+04, kdiss: 1.69e−02
1275_ID.2809_A8_SLLQHLIAL
KD: 1.49e−08
Kass: 5.25e+04, kdiss: 7.82e−04
1276_ID.281_L7_SLLQHLLGL
KD: 8.16e−09
Kass: 1.51e+05, kdiss: 1.23e−03
1277_ID.2811_I3_SLIQHLIGL
KD: 1.48e−08
Kass: 7.39e+04, kdiss: 1.10e−03
1278_ID.2812_D4_SLLDHLIGL
KD: 2.20e−08
Kass: 4.88e+04, kdiss: 1.07e−03
1279_ID.2813_WT_SLLQHLIGL
KD: 2.44e−08
Kass: 8.42e+04, kdiss: 2.06e−03
1280_ID.2814_S4_SLLSHLIGL
KD: 2.19e−08
Kass: 8.28e+04, kdiss: 1.81e−03
1281_ID.2815_P8_SLLQHLIPL
KD: 2.01e−08
Kass: 7.73e+04, kdiss: 1.56e−03
1282_ID.2816_A4_SLLAHLIGL
KD: 2.30e−08
Kass: 9.05e+04, kdiss: 2.08e−03
1283_ID.2818_F4_SLLFHLIGL
KD: 1.87e−08
Kass: 1.01e+05, kdiss: 1.89e−03
1284_ID.2819_N1_NLLQHLIGL
KD: 1.42e−08
Kass: 1.02e+05, kdiss: 1.44e−03
1285_ID.2820_E3_SLEQHLIGL
KD: 1.29e−08
Kass: 1.05e+05, kdiss: 1.35e−03
1286_ID.2821_K7_SLLQHLKGL
KD: 1.05e−07
Kass: 5.97e+04, kdiss: 6.28e−03
1287_ID.2822_T3_SLTQHLIGL
KD: 2.65e−08
Kass: 1.04e+05, kdiss: 2.75e−03
1288_ID.2823_Q6_SLLQHQIGL
KD: 2.51e−08
Kass: 7.19e+04, kdiss: 1.81e−03
1289_ID.2824_I4_SLLIHLIGL
KD: 2.87e−08
Kass: 8.52e+04, kdiss: 2.44e−03
1290_ID.2825_WT_SLLQHLIGL
KD: 2.06e−08
Kass: 8.90e+04, kdiss: 1.83e−03
1291_ID.2826_N5_SLLQNLIGL
KD: 3.96e−07
Kass: 5.08e+04, kdiss: 2.01e−02
1292_ID.2827_W8_SLLQHLIWL
KD:−
Kass:−, , kdiss: 3.87e−02
1293_ID.2829_H8_SLLQHLIHL
KD: 2.78e−07
Kass: 4.64e+04, kdiss: 1.29e−02
1294_ID.283_A1_ALLQHLIGL
KD: 1.68e−08
Kass: 1.44e+05, kdiss: 2.41e−03
1295_ID.2831_H3_SLHQHLIGL
KD: 5.22e−08
Kass: 9.31e+04, kdiss: 4.86e−03
1296_ID.2832_S3_SLSQHLIGL
KD: 2.31e−08
Kass: 1.23e+05, kdiss: 2.85e−03
1297_ID.2834_I5_SLLQILIGL
KD: 3.03e−07
Kass: 3.11e+03, kdiss: 9.40e−04
1298_ID.2835_I3_SLIQHLIGL
KD: 1.13e−08
Kass: 1.23e+05, kdiss: 1.38e−03
1299_ID.2837_M7_SLLQHLMGL
KD: 1.13e−08
Kass: 1.46e+05, kdiss: 1.66e−03
1300_ID.2838_Q3_SLQQHLIGL
KD: 9.61e−09
Kass: 1.53e+05, kdiss: 1.47e−03
value
20000
15000
10000
5000
0
0 250 500 750 1000
Time

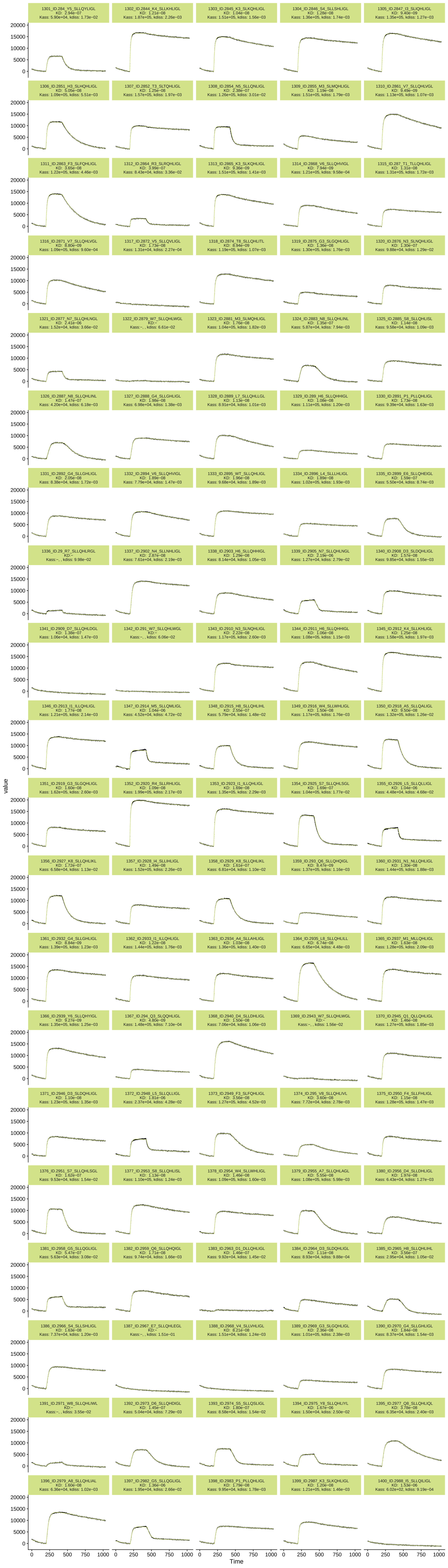

1301_ID.284_Y5_SLLQYLIGL
KD: 2.94e−07
Kass: 5.90e+04, kdiss: 1.73e−02
1302_ID.2844_K4_SLLKHLIGL
KD: 1.21e−08
Kass: 1.87e+05, kdiss: 2.26e−03
1303_ID.2845_K3_SLKQHLIGL
KD: 1.04e−08
Kass: 1.51e+05, kdiss: 1.56e−03
1304_ID.2846_S4_SLLSHLIGL
KD: 1.28e−08
Kass: 1.36e+05, kdiss: 1.74e−03
1305_ID.2847_I3_SLIQHLIGL
KD: 9.40e−09
Kass: 1.35e+05, kdiss: 1.27e−03
1306_ID.2851_H3_SLHQHLIGL
KD: 5.05e−08
Kass: 1.09e+05, kdiss: 5.51e−03
1307_ID.2852_T3_SLTQHLIGL
KD: 1.25e−08
Kass: 1.57e+05, kdiss: 1.97e−03
1308_ID.2854_N5_SLLQNLIGL
KD: 2.38e−07
Kass: 1.26e+05, kdiss: 3.01e−02
1309_ID.2855_M3_SLMQHLIGL
KD: 1.19e−08
Kass: 1.51e+05, kdiss: 1.79e−03
1310_ID.2861_V7_SLLQHLVGL
KD: 9.49e−09
Kass: 1.13e+05, kdiss: 1.07e−03
1311_ID.2863_F3_SLFQHLIGL
KD: 3.65e−08
Kass: 1.22e+05, kdiss: 4.46e−03
1312_ID.2864_R3_SLRQHLIGL
KD: 3.99e−07
Kass: 8.43e+04, kdiss: 3.36e−02
1313_ID.2865_K3_SLKQHLIGL
KD: 9.36e−09
Kass: 1.51e+05, kdiss: 1.41e−03
1314_ID.2868_V6_SLLQHVIGL
KD: 7.94e−09
Kass: 1.21e+05, kdiss: 9.58e−04
1315_ID.287_T1_TLLQHLIGL
KD: 1.31e−08
Kass: 1.31e+05, kdiss: 1.72e−03
1316_ID.2871_V7_SLLQHLVGL
KD: 8.80e−09
Kass: 1.09e+05, kdiss: 9.60e−04
1317_ID.2872_V5_SLLQVLIGL
KD: 1.73e−08
Kass: 1.31e+04, kdiss: 2.27e−04
1318_ID.2874_T8_SLLQHLITL
KD: 8.94e−09
Kass: 1.19e+05, kdiss: 1.07e−03
1319_ID.2875_G3_SLGQHLIGL
KD: 1.36e−08
Kass: 1.30e+05, kdiss: 1.76e−03
1320_ID.2876_N3_SLNQHLIGL
KD: 1.30e−07
Kass: 9.88e+04, kdiss: 1.29e−02
1321_ID.2877_N7_SLLQHLNGL
KD: 2.41e−06
Kass: 1.52e+04, kdiss: 3.66e−02
1322_ID.2879_W7_SLLQHLWGL
KD:−
Kass:−, , kdiss: 6.61e−02
1323_ID.2881_M3_SLMQHLIGL
KD: 1.76e−08
Kass: 1.04e+05, kdiss: 1.82e−03
1324_ID.2883_N8_SLLQHLINL
KD: 1.35e−07
Kass: 5.87e+04, kdiss: 7.94e−03
1325_ID.2885_S8_SLLQHLISL
KD: 1.14e−08
Kass: 9.58e+04, kdiss: 1.09e−03
1326_ID.2887_N8_SLLQHLINL
KD: 1.47e−07
Kass: 4.20e+04, kdiss: 6.18e−03
1327_ID.2888_G4_SLLGHLIGL
KD: 1.98e−08
Kass: 6.98e+04, kdiss: 1.38e−03
1328_ID.2889_L7_SLLQHLLGL
KD: 1.13e−08
Kass: 8.91e+04, kdiss: 1.01e−03
1329_ID.289_H6_SLLQHHIGL
KD: 1.08e−08
Kass: 1.11e+05, kdiss: 1.20e−03
1330_ID.2891_P1_PLLQHLIGL
KD: 1.73e−08
Kass: 9.39e+04, kdiss: 1.63e−03
1331_ID.2892_G4_SLLGHLIGL
KD: 2.05e−08
Kass: 8.38e+04, kdiss: 1.72e−03
1332_ID.2894_V6_SLLQHVIGL
KD: 1.89e−08
Kass: 7.79e+04, kdiss: 1.47e−03
1333_ID.2895_WT_SLLQHLIGL
KD: 1.96e−08
Kass: 9.68e+04, kdiss: 1.89e−03
1334_ID.2896_L4_SLLLHLIGL
KD: 1.89e−08
Kass: 1.02e+05, kdiss: 1.93e−03
1335_ID.2899_E6_SLLQHEIGL
KD: 1.59e−07
Kass: 5.50e+04, kdiss: 8.74e−03
1336_ID.29_R7_SLLQHLRGL
KD:−
Kass:−, , kdiss: 9.98e−02
1337_ID.2902_N4_SLLNHLIGL
KD: 2.87e−08
Kass: 7.61e+04, kdiss: 2.19e−03
1338_ID.2903_H6_SLLQHHIGL
KD: 1.29e−08
Kass: 8.14e+04, kdiss: 1.05e−03
1339_ID.2905_N7_SLLQHLNGL
KD: 2.19e−06
Kass: 1.27e+04, kdiss: 2.79e−02
1340_ID.2908_D3_SLDQHLIGL
KD: 1.57e−08
Kass: 9.85e+04, kdiss: 1.55e−03
1341_ID.2909_D7_SLLQHLDGL
KD: 1.38e−07
Kass: 1.06e+04, kdiss: 1.47e−03
1342_ID.291_W7_SLLQHLWGL
KD:−
Kass:−, , kdiss: 6.06e−02
1343_ID.2910_N3_SLNQHLIGL
KD: 2.22e−08
Kass: 1.17e+05, kdiss: 2.60e−03
1344_ID.2911_H6_SLLQHHIGL
KD: 1.06e−08
Kass: 1.08e+05, kdiss: 1.15e−03
1345_ID.2912_K4_SLLKHLIGL
KD: 1.25e−08
Kass: 1.58e+05, kdiss: 1.97e−03
1346_ID.2913_I1_ILLQHLIGL
KD: 1.77e−08
Kass: 1.21e+05, kdiss: 2.14e−03
1347_ID.2914_M5_SLLQMLIGL
KD: 1.04e−06
Kass: 4.52e+04, kdiss: 4.72e−02
1348_ID.2915_H8_SLLQHLIHL
KD: 2.55e−07
Kass: 5.79e+04, kdiss: 1.48e−02
1349_ID.2916_W4_SLLWHLIGL
KD: 1.50e−08
Kass: 1.17e+05, kdiss: 1.76e−03
1350_ID.2918_A5_SLLQALIGL
KD: 9.50e−08
Kass: 1.32e+05, kdiss: 1.26e−02
1351_ID.2919_G3_SLGQHLIGL
KD: 1.60e−08
Kass: 1.62e+05, kdiss: 2.60e−03
1352_ID.2920_R4_SLLRHLIGL
KD: 1.09e−08
Kass: 1.99e+05, kdiss: 2.17e−03
1353_ID.2923_I1_ILLQHLIGL
KD: 1.69e−08
Kass: 1.35e+05, kdiss: 2.29e−03
1354_ID.2925_S7_SLLQHLSGL
KD: 1.69e−07
Kass: 1.04e+05, kdiss: 1.77e−02
1355_ID.2926_L5_SLLQLLIGL
KD: 1.04e−06
Kass: 4.48e+04, kdiss: 4.68e−02
1356_ID.2927_K8_SLLQHLIKL
KD: 1.72e−07
Kass: 6.58e+04, kdiss: 1.13e−02
1357_ID.2928_I4_SLLIHLIGL
KD: 1.49e−08
Kass: 1.52e+05, kdiss: 2.26e−03
1358_ID.2929_K8_SLLQHLIKL
KD: 1.61e−07
Kass: 6.81e+04, kdiss: 1.10e−02
1359_ID.293_Q6_SLLQHQIGL
KD: 8.47e−09
Kass: 1.37e+05, kdiss: 1.16e−03
1360_ID.2931_N1_NLLQHLIGL
KD: 1.30e−08
Kass: 1.44e+05, kdiss: 1.88e−03
1361_ID.2932_G4_SLLGHLIGL
KD: 8.84e−09
Kass: 1.39e+05, kdiss: 1.23e−03
1362_ID.2933_I1_ILLQHLIGL
KD: 1.22e−08
Kass: 1.44e+05, kdiss: 1.76e−03
1363_ID.2934_A4_SLLAHLIGL
KD: 1.03e−08
Kass: 1.36e+05, kdiss: 1.40e−03
1364_ID.2935_L8_SLLQHLILL
KD: 6.74e−08
Kass: 6.65e+04, kdiss: 4.48e−03
1365_ID.2937_M1_MLLQHLIGL
KD: 1.63e−08
Kass: 1.28e+05, kdiss: 2.09e−03
1366_ID.2939_Y6_SLLQHYIGL
KD: 9.27e−09
Kass: 1.35e+05, kdiss: 1.25e−03
1367_ID.294_Q3_SLQQHLIGL
KD: 4.80e−09
Kass: 1.48e+05, kdiss: 7.10e−04
1368_ID.2940_D4_SLLDHLIGL
KD: 1.50e−08
Kass: 7.06e+04, kdiss: 1.06e−03
1369_ID.2943_W7_SLLQHLWGL
KD:−
Kass:−, , kdiss: 1.56e−02
1370_ID.2945_Q1_QLLQHLIGL
KD: 1.46e−08
Kass: 1.27e+05, kdiss: 1.85e−03
1371_ID.2946_D3_SLDQHLIGL
KD: 1.10e−08
Kass: 1.23e+05, kdiss: 1.35e−03
1372_ID.2948_L5_SLLQLLIGL
KD: 1.81e−06
Kass: 2.37e+04, kdiss: 4.28e−02
1373_ID.2949_F3_SLFQHLIGL
KD: 3.56e−08
Kass: 1.27e+05, kdiss: 4.52e−03
1374_ID.295_V8_SLLQHLIVL
KD: 3.60e−08
Kass: 7.72e+04, kdiss: 2.78e−03
1375_ID.2950_F4_SLLFHLIGL
KD: 1.15e−08
Kass: 1.28e+05, kdiss: 1.47e−03
1376_ID.2951_S7_SLLQHLSGL
KD: 1.62e−07
Kass: 9.53e+04, kdiss: 1.54e−02
1377_ID.2953_S8_SLLQHLISL
KD: 1.13e−08
Kass: 1.10e+05, kdiss: 1.24e−03
1378_ID.2954_W4_SLLWHLIGL
KD: 1.46e−08
Kass: 1.09e+05, kdiss: 1.60e−03
1379_ID.2955_A7_SLLQHLAGL
KD: 5.55e−08
Kass: 1.08e+05, kdiss: 5.98e−03
1380_ID.2956_D4_SLLDHLIGL
KD: 1.97e−08
Kass: 6.43e+04, kdiss: 1.27e−03
1381_ID.2958_G5_SLLQGLIGL
KD: 5.47e−07
Kass: 5.63e+04, kdiss: 3.08e−02
1382_ID.2959_Q6_SLLQHQIGL
KD: 1.71e−08
Kass: 9.74e+04, kdiss: 1.66e−03
1383_ID.2963_D1_DLLQHLIGL
KD: 1.46e−07
Kass: 9.92e+04, kdiss: 1.45e−02
1384_ID.2964_D3_SLDQHLIGL
KD: 1.11e−08
Kass: 8.93e+04, kdiss: 9.88e−04
1385_ID.2965_H8_SLLQHLIHL
KD: 3.56e−07
Kass: 2.95e+04, kdiss: 1.05e−02
1386_ID.2966_S4_SLLSHLIGL
KD: 1.63e−08
Kass: 7.37e+04, kdiss: 1.20e−03
1387_ID.2967_E7_SLLQHLEGL
KD:−
Kass:−, , kdiss: 1.51e−01
1388_ID.2968_V4_SLLVHLIGL
KD: 8.21e−08
Kass: 1.51e+04, kdiss: 1.24e−03
1389_ID.2969_G3_SLGQHLIGL
KD: 2.36e−08
Kass: 1.01e+05, kdiss: 2.38e−03
1390_ID.2970_G4_SLLGHLIGL
KD: 1.84e−08
Kass: 8.37e+04, kdiss: 1.54e−03
1391_ID.2971_W8_SLLQHLIWL
KD:−
Kass:−, , kdiss: 3.55e−02
1392_ID.2973_D6_SLLQHDIGL
KD: 1.45e−07
Kass: 5.04e+04, kdiss: 7.29e−03
1393_ID.2974_S5_SLLQSLIGL
KD: 1.80e−07
Kass: 8.58e+04, kdiss: 1.54e−02
1394_ID.2975_Y8_SLLQHLIYL
KD: 1.67e−06
Kass: 1.50e+04, kdiss: 2.50e−02
1395_ID.2977_Q8_SLLQHLIQL
KD: 3.78e−08
Kass: 6.35e+04, kdiss: 2.40e−03
1396_ID.2979_A8_SLLQHLIAL
KD: 1.60e−08
Kass: 6.36e+04, kdiss: 1.02e−03
1397_ID.2982_G5_SLLQGLIGL
KD: 1.36e−06
Kass: 1.95e+04, kdiss: 2.66e−02
1398_ID.2983_P1_PLLQHLIGL
KD: 1.79e−08
Kass: 9.95e+04, kdiss: 1.78e−03
1399_ID.2987_K3_SLKQHLIGL
KD: 1.20e−08
Kass: 1.21e+05, kdiss: 1.46e−03
1400_ID.2988_I5_SLLQILIGL
KD: 1.53e−06
Kass: 6.02e+02, kdiss: 9.19e−04
value
20000
15000
10000
5000
0
0
250
500
750
1000
Time

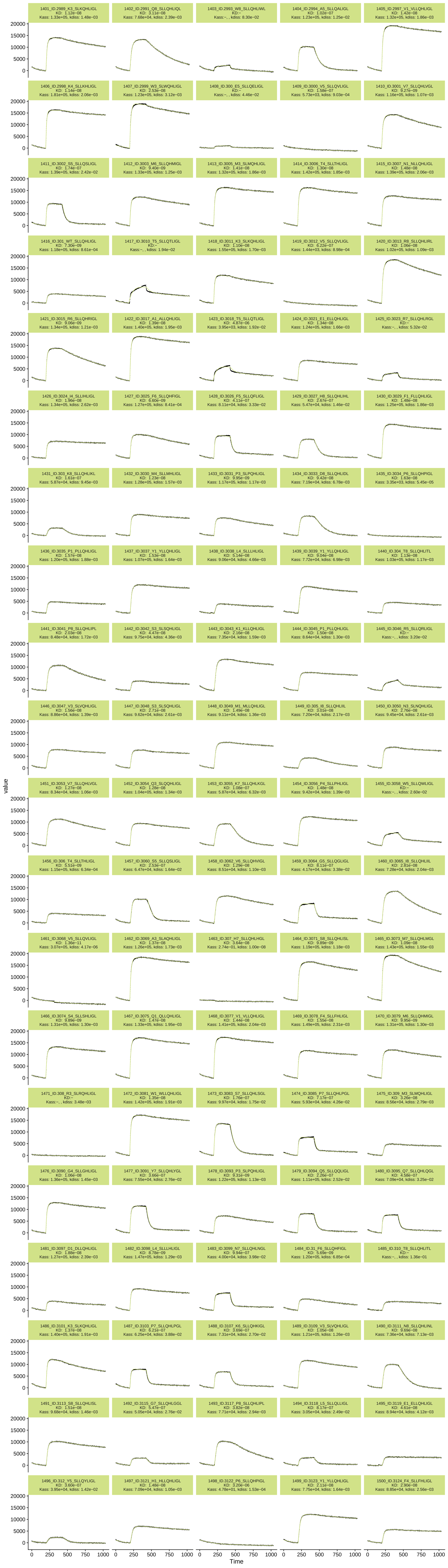

value
Time
20000
15000
10000
5000
0
0
250
500
750
1000

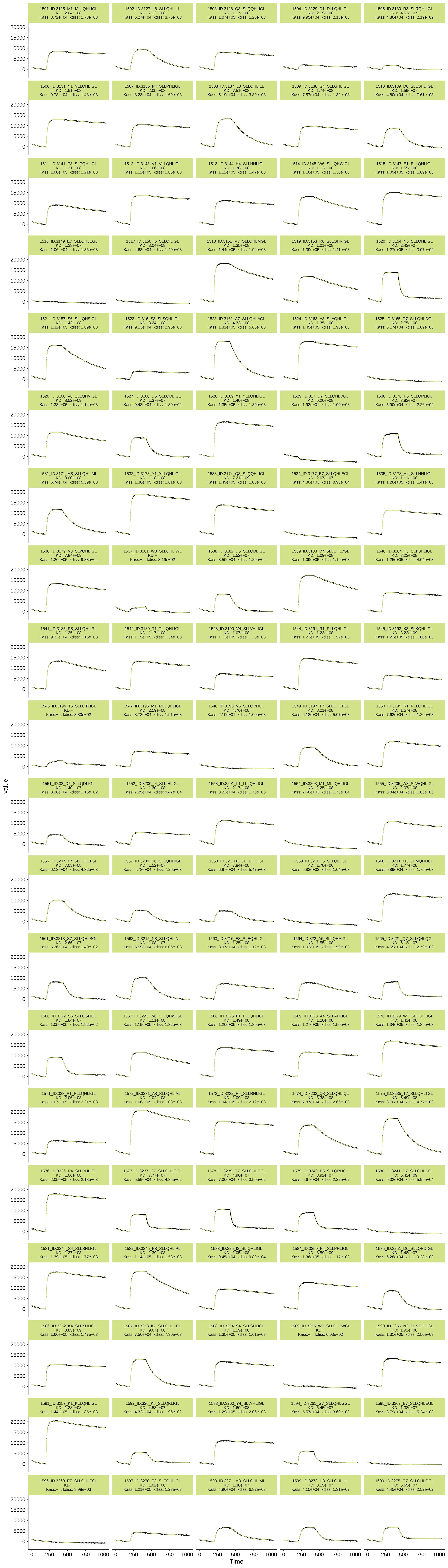

1501_ID.3125_M1_MLLQHLIGL KD: 2.04e−08 Kass: 8.72e+04, kdiss: 1.78e−03
1502_ID.3127_L8_SLLQHLILL KD: 7.13e−08 Kass: 5.27e+04, kdiss: 3.76e−03
1503_ID.3128_Q3_SLQQHLIGL KD: 1.17e−08 Kass: 1.07e+05, kdiss: 1.25e−03
1504_ID.3129_D1_DLLQHLIGL KD: 2.19e−08 Kass: 9.95e+04, kdiss: 2.18e−03
1505_ID.3130_R3_SLRQHLIGL KD: 4.51e−07 Kass: 4.86e+04, kdiss: 2.19e−02
1506_ID.3131_Y1_YLLQHLIGL KD: 1.51e−08 Kass: 9.78e+04, kdiss: 1.48e−03
1507_ID.3136_P4_SLLPHLIGL KD: 2.05e−08 Kass: 8.23e+04, kdiss: 1.69e−03
1508_ID.3137_L8_SLLQHLILL KD: 7.51e−08 Kass: 5.19e+04, kdiss: 3.89e−03
1509_ID.3138_G4_SLLGHLIGL KD: 1.74e−08 Kass: 7.57e+04, kdiss: 1.32e−03
1510_ID.3139_D6_SLLQHDIGL KD: 1.59e−07 Kass: 4.80e+04, kdiss: 7.61e−03
1511_ID.3141_P3_SLPQHLIGL KD: 1.21e−08 Kass: 1.00e+05, kdiss: 1.21e−03
1512_ID.3143_V1_VLLQHLIGL KD: 1.66e−08 Kass: 1.12e+05, kdiss: 1.86e−03
1513_ID.3144_H4_SLLHHLIGL KD: 1.30e−08 Kass: 1.12e+05, kdiss: 1.47e−03
1514_ID.3145_W8_SLLQHLIWGL KD: 1.35e−08 Kass: 1.16e+05, kdiss: 1.30e−03
1515_ID.3147_E1_ELLQHLIGL KD: 1.55e−08 Kass: 1.09e+05, kdiss: 1.69e−03
1516_ID.3149_E7_SLLQHLEGL KD: 1.28e−08 Kass: 1.06e+04, kdiss: 1.36e−03
1517_ID.3150_I5_SLLQILIGL KD: 3.04e−08 Kass: 4.63e+04, kdiss: 1.40e−03
1518_ID.3151_M7_SLLQHLMGL KD: 1.35e−08 Kass: 1.44e+05, kdiss: 1.94e−03
1519_ID.3153_R6_SLLQHRIGL KD: 1.01e−08 Kass: 1.39e+05, kdiss: 1.41e−03
1520_ID.3154_N5_SLLQNLIGL KD: 2.42e−07 Kass: 1.27e+05, kdiss: 3.07e−02
1521_ID.3157_S6_SLLQHSIGL KD: 1.43e−08 Kass: 1.32e+05, kdiss: 1.89e−03
1522_ID.316_S3_SLSQHLIGL KD: 3.24e−08 Kass: 9.13e+04, kdiss: 2.96e−03
1523_ID.3161_A7_SLLQHLAGL KD: 4.33e−08 Kass: 1.31e+05, kdiss: 5.65e−03
1524_ID.3163_A3_SLAQHLIGL KD: 1.35e−08 Kass: 1.45e+05, kdiss: 1.95e−03
1525_ID.3165_D7_SLLQHLDGL KD: 2.75e−08 Kass: 6.17e+04, kdiss: 1.69e−03
1526_ID.3166_V6_SLLQHVIGL KD: 8.52e−09 Kass: 1.33e+05, kdiss: 1.14e−03
1527_ID.3168_D5_SLLQDLIGL KD: 1.37e−07 Kass: 9.48e+04, kdiss: 1.30e−02
1528_ID.3169_Y1_YLLQHLIGL KD: 1.40e−08 Kass: 1.35e+05, kdiss: 1.89e−03
1529_ID.317_D7_SLLQHLDGL KD: 5.20e−08 Kass: 1.92e−01, kdiss: 1.00e−08
1530_ID.3170_P5_SLLQPLIGL KD: 3.82e−07 Kass: 5.90e+04, kdiss: 2.26e−02
1531_ID.3171_M8_SLLQHLIML KD: 8.00e−08 Kass: 6.74e+04, kdiss: 5.39e−03
1532_ID.3173_Y1_YLLQHLIGL KD: 1.18e−08 Kass: 1.36e+05, kdiss: 1.61e−03
1533_ID.3174_Q3_SLQQHLIGL KD: 7.21e−09 Kass: 1.49e+05, kdiss: 1.08e−03
1534_ID.3177_E7_SLLQHLEGL KD: 2.07e−07 Kass: 4.30e+03, kdiss: 8.93e−04
1535_ID.3178_H4_SLLHHLIGL KD: 1.11e−08 Kass: 1.28e+05, kdiss: 1.41e−03
1536_ID.3179_V3_SLVQHLIGL KD: 7.84e−09 Kass: 1.26e+05, kdiss: 9.88e−04
1537_ID.3181_W8_SLLQHLIWL KD:~ Kass:~, , kdiss: 8.19e−02
1538_ID.3182_D5_SLLQDLIGL KD: 1.52e−07 Kass: 8.50e+04, kdiss: 1.29e−02
1539_ID.3183_V7_SLLQHLVGL KD: 1.09e−08 Kass: 1.09e+05, kdiss: 1.19e−03
1540_ID.3184_T3_SLTQHLIGL KD: 3.22e−08 Kass: 1.25e+05, kdiss: 4.04e−03
1541_ID.3185_R8_SLLQHLIRL KD: 1.25e−08 Kass: 9.32e+04, kdiss: 1.16e−03
1542_ID.3189_T1_TLLQHLIGL KD: 1.17e−08 Kass: 1.15e+05, kdiss: 1.34e−03
1543_ID.3190_V4_SLLVHLIGL KD: 1.07e−08 Kass: 1.13e+05, kdiss: 1.20e−03
1544_ID.3191_R1_RLLQHLIGL KD: 1.23e−08 Kass: 1.23e+05, kdiss: 1.52e−03
1545_ID.3193_K3_SLKQHLIGL KD: 8.22e−09 Kass: 1.22e+05, kdiss: 1.00e−03
1546_ID.3194_T5_SLLQTLIGL KD:~ Kass:~, , kdiss: 3.80e−02
1547_ID.3195_M1_MLLQHLIGL KD: 2.19e−08 Kass: 8.73e+04, kdiss: 1.91e−03
1548_ID.3196_V5_SLLQVLIGL KD: 4.76e−08 Kass: 2.10e−01, kdiss: 1.00e−08
1549_ID.3197_T7_SLLQHLTGL KD: 8.21e−08 Kass: 6.18e+04, kdiss: 5.07e−03
1550_ID.3199_R1_RLLQHLIGL KD: 1.57e−08 Kass: 7.62e+04, kdiss: 1.20e−03
1551_ID.32_D5_SLLQDLIGL KD: 1.40e−07 Kass: 8.28e+04, kdiss: 1.16e−02
1552_ID.3200_I4_SLLIHLIGL KD: 1.30e−08 Kass: 7.29e+04, kdiss: 9.47e−04
1553_ID.3201_L1_LLLQHLIGL KD: 2.17e−08 Kass: 8.22e+04, kdiss: 1.78e−03
1554_ID.3203_M1_MLLQHLIGL KD: 2.25e−08 Kass: 7.68e+03, kdiss: 1.73e−04
1555_ID.3205_W3_SLWQHLIGL KD: 2.07e−08 Kass: 8.84e+04, kdiss: 1.83e−03
1556_ID.3207_T7_SLLQHLTGL KD: 7.05e−08 Kass: 6.13e+04, kdiss: 4.32e−03
1557_ID.3209_D6_SLLQHDIGL KD: 1.52e−07 Kass: 4.78e+04, kdiss: 7.26e−03
1558_ID.321_H3_SLHQHLIGL KD: 7.84e−08 Kass: 6.97e+04, kdiss: 5.47e−03
1559_ID.3210_I5_SLLQILIGL KD: 1.78e−06 Kass: 5.83e+02, kdiss: 1.04e−03
1560_ID.3211_M3_SLMQHLIGL KD: 1.77e−08 Kass: 9.89e+04, kdiss: 1.75e−03
1561_ID.3213_S7_SLLQHLSGL KD: 2.66e−07 Kass: 5.26e+04, kdiss: 1.40e−02
1562_ID.3215_N8_SLLQHLINL KD: 1.08e−07 Kass: 5.59e+04, kdiss: 6.06e−03
1563_ID.3216_E3_SLEQHLIGL KD: 1.25e−08 Kass: 8.97e+04, kdiss: 1.12e−03
1564_ID.322_A6_SLLQHAIGL KD: 1.55e−08 Kass: 1.03e+05, kdiss: 1.59e−03
1565_ID.3221_Q7_SLLQHLQGL KD: 6.13e−07 Kass: 4.55e+04, kdiss: 2.79e−02
1566_ID.3222_S5_SLLQSLIGL KD: 1.84e−07 Kass: 1.05e+05, kdiss: 1.92e−02
1567_ID.3223_W6_SLLQHWIGL KD: 1.11e−08 Kass: 1.19e+05, kdiss: 1.32e−03
1568_ID.3225_F1_FLLQHLIGL KD: 1.49e−08 Kass: 1.26e+05, kdiss: 1.89e−03
1569_ID.3228_A4_SLLAHLIGL KD: 1.18e−08 Kass: 1.27e+05, kdiss: 1.50e−03
1570_ID.3229_W7_SLLQHLWGL KD: 1.41e−08 Kass: 1.34e+05, kdiss: 1.89e−03
1571_ID.323_P1_PLLQHLIGL KD: 2.06e−08 Kass: 1.07e+05, kdiss: 2.21e−03
1572_ID.3231_A8_SLLQHLIAL KD: 1.02e−08 Kass: 1.06e+05, kdiss: 1.08e−03
1573_ID.3232_R4_SLLRHLIGL KD: 1.09e−08 Kass: 1.94e+05, kdiss: 2.12e−03
1574_ID.3233_Q8_SLLQHLIQL KD: 3.38e−08 Kass: 7.87e+04, kdiss: 2.66e−03
1575_ID.3235_T7_SLLQHLTGL KD: 5.49e−08 Kass: 8.70e+04, kdiss: 4.77e−03
1576_ID.3236_R4_SLLRHLIGL KD: 1.06e−08 Kass: 2.05e+05, kdiss: 2.18e−03
1577_ID.3237_G7_SLLQHLGGL KD: 7.77e−07 Kass: 5.59e+04, kdiss: 4.35e−02
1578_ID.3239_Q7_SLLQHLQGL KD: 4.96e−07 Kass: 7.06e+04, kdiss: 3.50e−02
1579_ID.3240_P5_SLLQPLIGL KD: 3.92e−07 Kass: 5.67e+04, kdiss: 2.22e−02
1580_ID.3241_D7_SLLQHLDGL KD: 6.42e−09 Kass: 9.32e+04, kdiss: 5.99e−04
1581_ID.3244_S4_SLLSHLIGL KD: 1.27e−08 Kass: 1.39e+05, kdiss: 1.77e−03
1582_ID.3245_P8_SLLQHLIPL KD: 1.39e−08 Kass: 1.14e+05, kdiss: 1.58e−03
1583_ID.325_I3_SLIQHLIGL KD: 1.05e−08 Kass: 9.45e+04, kdiss: 9.89e−04
1584_ID.3250_P4_SLLPHLIGL KD: 8.59e−09 Kass: 1.36e+05, kdiss: 1.17e−03
1585_ID.3251_D6_SLLQHDIGL KD: 1.48e−07 Kass: 6.28e+04, kdiss: 9.28e−03
1586_ID.3252_K4_SLLKHLIGL KD: 8.85e−09 Kass: 1.66e+05, kdiss: 1.47e−03
1587_ID.3253_K7_SLLQHLKGL KD: 9.67e−08 Kass: 7.56e+04, kdiss: 7.30e−03
1588_ID.3254_S4_SLLSHLIGL KD: 1.19e−08 Kass: 1.35e+05, kdiss: 1.61e−03
1589_ID.3255_W7_SLLQHLWGL KD:~ Kass:~, , kdiss: 6.03e−02
1590_ID.3256_N3_SLNQHLIGL KD: 1.91e−08 Kass: 1.31e+05, kdiss: 2.50e−03
1591_ID.3257_K1_KLLQHLIGL KD: 1.28e−08 Kass: 1.44e+05, kdiss: 1.85e−03
1592_ID.326_K5_SLLQKLIGL KD: 4.53e−07 Kass: 4.32e+04, kdiss: 1.96e−02
1593_ID.3260_Y4_SLLYHLIGL KD: 1.60e−08 Kass: 1.29e+05, kdiss: 2.06e−03
1594_ID.3261_G7_SLLQHLGGL KD: 6.45e−07 Kass: 5.57e+04, kdiss: 3.60e−02
1595_ID.3267_E7_SLLQHLEGL KD: 1.38e−07 Kass: 3.79e+04, kdiss: 5.24e−03
1596_ID.3269_E7_SLLQHLEGL KD:~ Kass:~, , kdiss: 8.98e−03
1597_ID.3270_E3_SLEQHLIGL KD: 1.02e−08 Kass: 1.21e+05, kdiss: 1.23e−03
1598_ID.3271_M8_SLLQHLIML KD: 1.38e−07 Kass: 4.96e+04, kdiss: 6.82e−03
1599_ID.3273_H8_SLLQHLIHL KD: 3.15e−07 Kass: 4.15e+04, kdiss: 1.31e−02
1600_ID.3275_Q7_SLLQHLQGL KD: 5.65e−07 Kass: 4.45e+04, kdiss: 2.52e−02
value
Time

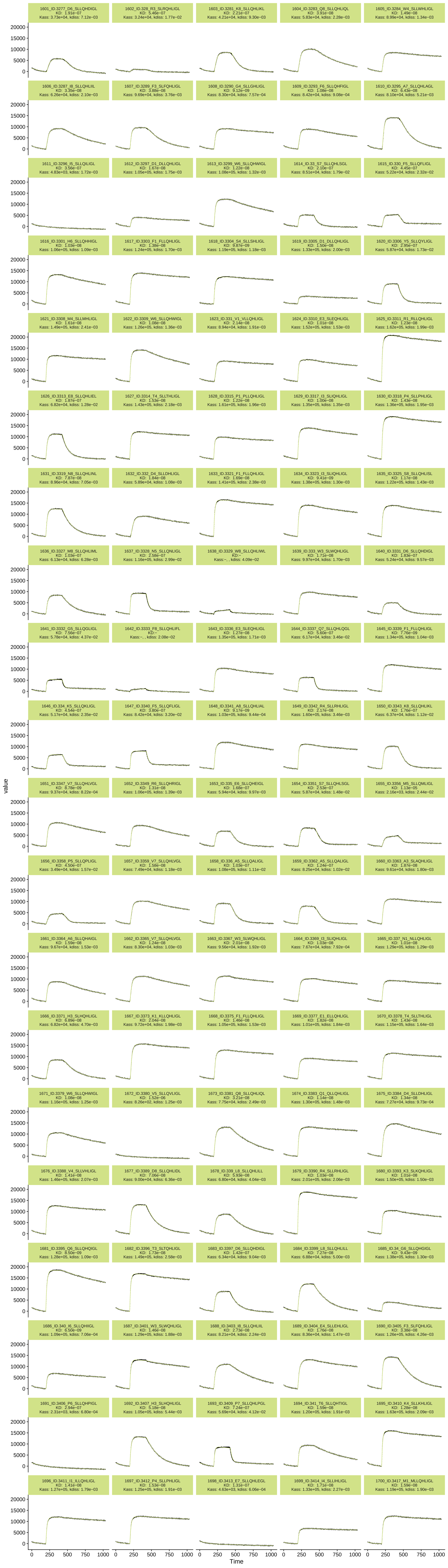

value
Time
0
250
500
750
1000
0
5000
10000
15000
20000

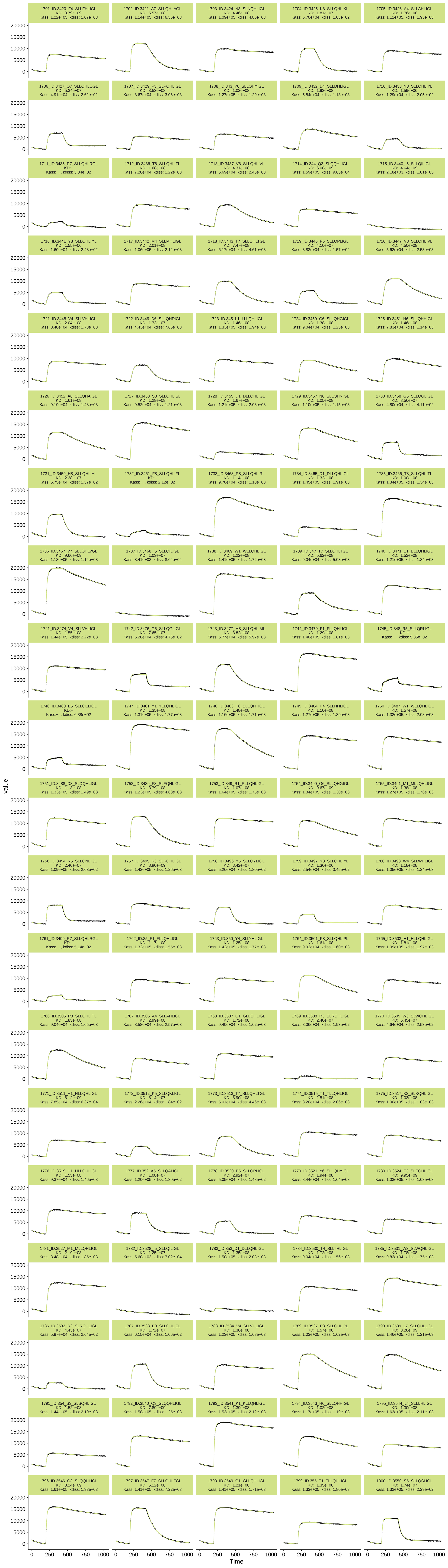
value
Time
0
250
500
750
1000
0
5000
10000
15000
20000

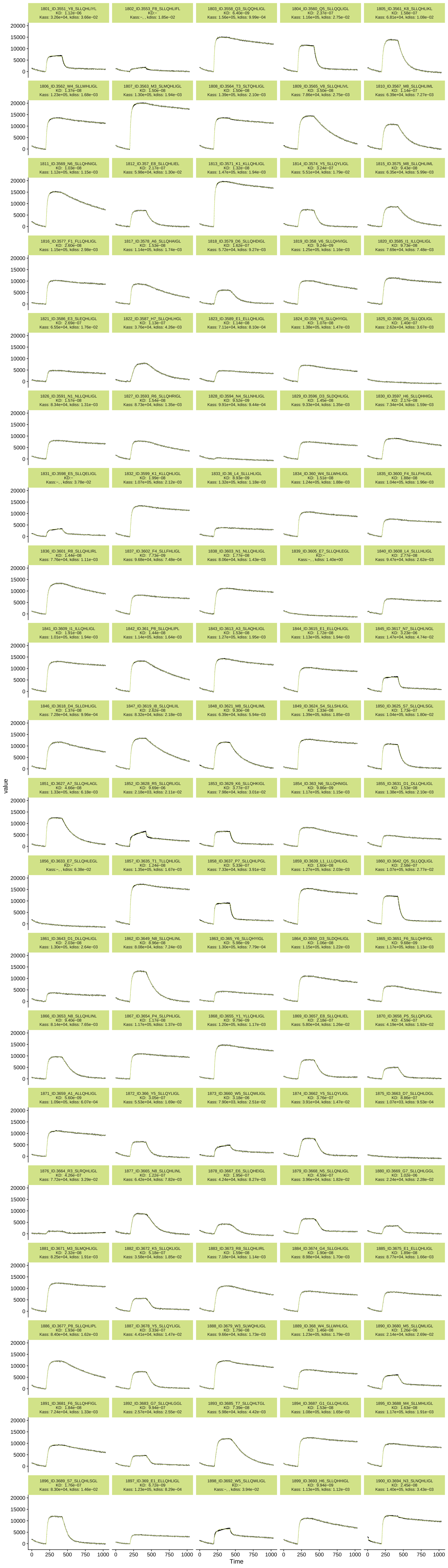

value
Time
0
5000
10000
15000
20000
250
500
750
1000

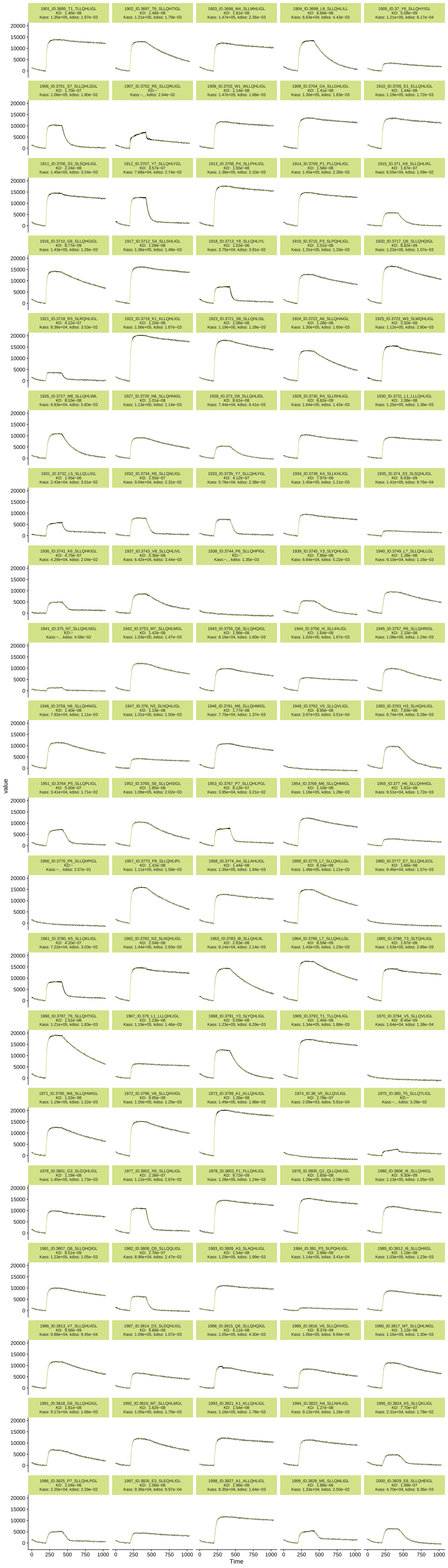

1901_ID.3695_T1_TLLQHLIGL
KD: 1.46e−08
Kass: 1.35e+05, kdiss: 1.97e−03
1902_ID.3697_T6_SLLQHTIGL
KD: 1.48e−08
Kass: 1.21e+05, kdiss: 1.79e−03
1903_ID.3698_M4_SLLMHLIGL
KD: 1.61e−08
Kass: 1.47e+05, kdiss: 2.36e−03
1904_ID.3699_L8_SLLQHLILL
KD: 6.68e−08
Kass: 6.63e+04, kdiss: 4.43e−03
1905_ID.37_Y6_SLLQHYIGL
KD: 5.09e−09
Kass: 1.21e+05, kdiss: 6.17e−04
1906_ID.3701_S7_SLLQHLSGL
KD: 1.70e−07
Kass: 1.06e+05, kdiss: 1.80e−02
1907_ID.3702_R5_SLLQRLIGL
KD:−
Kass:−, , kdiss: 2.64e−02
1908_ID.3703_W1_WLLQHLIGL
KD: 1.14e−08
Kass: 1.47e+05, kdiss: 1.68e−03
1909_ID.3704_G4_SLLGHLIGL
KD: 1.41e−08
Kass: 1.30e+05, kdiss: 1.83e−03
1910_ID.3705_E1_ELLQHLIGL
KD: 1.44e−08
Kass: 1.19e+05, kdiss: 1.72e−03
1911_ID.3706_S3_SLSQHLIGL
KD: 2.24e−08
Kass: 1.45e+05, kdiss: 3.24e−03
1912_ID.3707_Y7_SLLQHLYGL
KD: 3.57e−07
Kass: 7.68e+04, kdiss: 2.74e−02
1913_ID.3708_P4_SLLPHLIGL
KD: 1.55e−08
Kass: 1.36e+05, kdiss: 2.10e−03
1914_ID.3709_P1_PLLQHLIGL
KD: 1.58e−08
Kass: 1.45e+05, kdiss: 2.30e−03
1915_ID.371_K8_SLLQHLIKL
KD: 1.67e−07
Kass: 6.55e+04, kdiss: 1.09e−02
1916_ID.3710_G6_SLLQHGIGL
KD: 8.77e−09
Kass: 1.43e+05, kdiss: 1.26e−03
1917_ID.3712_S4_SLLSHLIGL
KD: 1.09e−08
Kass: 1.36e+05, kdiss: 1.48e−03
1918_ID.3713_Y8_SLLQHLIYL
KD: 1.02e−06
Kass: 3.75e+04, kdiss: 3.81e−02
1919_ID.3715_P3_SLPQHLIGL
KD: 1.01e−08
Kass: 1.31e+05, kdiss: 1.33e−03
1920_ID.3717_Q6_SLLQHQIGL
KD: 8.82e−09
Kass: 1.22e+05, kdiss: 1.07e−03
1921_ID.3718_R3_SLRQHLIGL
KD: 4.22e−07
Kass: 8.36e+04, kdiss: 3.53e−02
1922_ID.3719_K1_KLLQHLIGL
KD: 1.20e−08
Kass: 1.56e+05, kdiss: 1.87e−03
1923_ID.3721_S8_SLLQHLISL
KD: 1.08e−08
Kass: 1.19e+05, kdiss: 1.28e−03
1924_ID.3722_A6_SLLQHAIGL
KD: 1.28e−08
Kass: 1.30e+05, kdiss: 1.65e−03
1925_ID.3723_W3_SLWQHLIGL
KD: 2.50e−08
Kass: 1.12e+05, kdiss: 2.80e−03
1926_ID.3727_M8_SLLQHLIML
KD: 8.53e−08
Kass: 6.83e+04, kdiss: 5.83e−03
1927_ID.3729_N6_SLLQHNIGL
KD: 1.01e−08
Kass: 1.13e+05, kdiss: 1.14e−03
1928_ID.373_D8_SLLQHLIDL
KD: 8.61e−08
Kass: 7.44e+04, kdiss: 6.41e−03
1929_ID.3730_R4_SLLRHLIGL
KD: 8.62e−09
Kass: 1.64e+05, kdiss: 1.42e−03
1930_ID.3731_L1_LLLQHLIGL
KD: 1.08e−08
Kass: 1.25e+05, kdiss: 1.36e−03
1931_ID.3732_L5_SLLQLLIGL
KD: 1.45e−06
Kass: 2.43e+04, kdiss: 3.51e−02
1932_ID.3734_N5_SLLQNLIGL
KD: 2.55e−07
Kass: 9.04e+04, kdiss: 2.31e−02
1933_ID.3735_Y7_SLLQHLYGL
KD: 4.12e−07
Kass: 5.76e+04, kdiss: 2.38e−02
1934_ID.3736_K4_SLLKHLIGL
KD: 7.97e−09
Kass: 1.40e+05, kdiss: 1.11e−03
1935_ID.374_S3_SLSQHLIGL
KD: 6.93e−09
Kass: 1.41e+05, kdiss: 9.76e−04
1936_ID.3741_K6_SLLQHKIGL
KD: 4.75e−07
Kass: 4.29e+04, kdiss: 2.04e−02
1937_ID.3743_V8_SLLQHLIVL
KD: 5.36e−08
Kass: 6.42e+04, kdiss: 3.44e−03
1938_ID.3744_P6_SLLQHPIGL
KD:−
Kass:−, , kdiss: 1.35e−03
1939_ID.3745_Y3_SLYQHLIGL
KD: 7.86e−08
Kass: 6.64e+04, kdiss: 5.22e−03
1940_ID.3749_L7_SLLQHLLGL
KD: 1.28e−08
Kass: 9.10e+04, kdiss: 1.16e−03
1941_ID.375_N7_SLLQHLNGL
KD:−
Kass:−, , kdiss: 4.56e−02
1942_ID.3753_M7_SLLQHLMGL
KD: 1.42e−08
Kass: 1.03e+05, kdiss: 1.47e−03
1943_ID.3755_Q6_SLLQHQIGL
KD: 1.96e−08
Kass: 8.16e+04, kdiss: 1.60e−03
1944_ID.3756_I4_SLLIHLIGL
KD: 1.64e−08
Kass: 1.02e+05, kdiss: 1.67e−03
1945_ID.3757_R6_SLLQHRIGL
KD: 1.15e−08
Kass: 1.08e+05, kdiss: 1.24e−03
1946_ID.3759_N6_SLLQHNIGL
KD: 1.40e−08
Kass: 7.92e+04, kdiss: 1.11e−03
1947_ID.376_N3_SLNQHLIGL
KD: 1.15e−08
Kass: 1.31e+05, kdiss: 1.50e−03
1948_ID.3761_M6_SLLQHMIGL
KD: 1.77e−08
Kass: 7.75e+04, kdiss: 1.37e−03
1949_ID.3762_V5_SLLQVLIGL
KD: 8.85e−08
Kass: 3.97e+03, kdiss: 3.51e−04
1950_ID.3763_H3_SLHQHLIGL
KD: 7.83e−08
Kass: 6.74e+04, kdiss: 5.28e−03
1951_ID.3764_P5_SLLQPLIGL
KD: 5.00e−07
Kass: 3.41e+04, kdiss: 1.71e−02
1952_ID.3765_S6_SLLQHSIGL
KD: 1.85e−08
Kass: 1.09e+05, kdiss: 2.02e−03
1953_ID.3767_P7_SLLQHLPGL
KD: 8.12e−07
Kass: 3.95e+04, kdiss: 3.21e−02
1954_ID.3769_M6_SLLQHMIGL
KD: 1.10e−08
Kass: 1.16e+05, kdiss: 1.28e−03
1955_ID.377_H6_SLLQHHIGL
KD: 1.81e−08
Kass: 9.51e+04, kdiss: 1.72e−03
1956_ID.3770_P6_SLLQHPIGL
KD:−
Kass:−, , kdiss: 2.57e−01
1957_ID.3773_P8_SLLQHLIPL
KD: 1.42e−08
Kass: 1.11e+05, kdiss: 1.58e−03
1958_ID.3774_A4_SLLAHLIGL
KD: 1.44e−08
Kass: 1.35e+05, kdiss: 1.94e−03
1959_ID.3775_L7_SLLQHLLGL
KD: 8.16e−09
Kass: 1.48e+05, kdiss: 1.21e−03
1960_ID.3777_E7_SLLQHLEGL
KD: 1.66e−08
Kass: 9.46e+04, kdiss: 1.57e−03
1961_ID.3780_K5_SLLQKLIGL
KD: 4.20e−07
Kass: 7.22e+04, kdiss: 3.03e−02
1962_ID.3782_N3_SLNQHLIGL
KD: 2.04e−08
Kass: 1.44e+05, kdiss: 2.93e−03
1963_ID.3783_I8_SLLQHLIIL
KD: 2.63e−08
Kass: 8.14e+04, kdiss: 2.14e−03
1964_ID.3785_L7_SLLQHLLGL
KD: 8.59e−09
Kass: 1.43e+05, kdiss: 1.23e−03
1965_ID.3786_T3_SLTQHLIGL
KD: 1.87e−08
Kass: 1.53e+05, kdiss: 2.86e−03
1966_ID.3787_T6_SLLQHTIGL
KD: 1.51e−08
Kass: 1.21e+05, kdiss: 1.83e−03
1967_ID.379_L1_LLLQHLIGL
KD: 1.23e−08
Kass: 1.19e+05, kdiss: 1.46e−03
1968_ID.3791_Y3_SLYQHLIGL
KD: 5.09e−08
Kass: 1.23e+05, kdiss: 6.29e−03
1969_ID.3793_T1_TLLQHLIGL
KD: 1.40e−08
Kass: 1.34e+05, kdiss: 1.88e−03
1970_ID.3794_V5_SLLQVLIGL
KD: 8.45e−09
Kass: 1.64e+04, kdiss: 1.38e−04
1971_ID.3795_W6_SLLQHWIGL
KD: 1.02e−08
Kass: 1.19e+05, kdiss: 1.22e−03
1972_ID.3796_V6_SLLQHVIGL
KD: 9.95e−09
Kass: 1.26e+05, kdiss: 1.25e−03
1973_ID.3799_K1_KLLQHLIGL
KD: 1.26e−08
Kass: 1.49e+05, kdiss: 1.88e−03
1974_ID.38_V5_SLLQVLIGL
KD: 2.78e−07
Kass: 2.09e+03, kdiss: 5.81e−04
1975_ID.380_T5_SLLQTLIGL
KD:−
Kass:−, , kdiss: 3.28e−02
1976_ID.3801_G3_SLGQHLIGL
KD: 1.19e−08
Kass: 1.45e+05, kdiss: 1.73e−03
1977_ID.3802_N5_SLLQNLIGL
KD: 2.38e−07
Kass: 1.12e+05, kdiss: 2.67e−02
1978_ID.3803_F1_FLLQHLIGL
KD: 9.72e−09
Kass: 1.28e+05, kdiss: 1.24e−03
1979_ID.3805_Q1_QLLQHLIGL
KD: 1.65e−08
Kass: 1.26e+05, kdiss: 2.08e−03
1980_ID.3806_I6_SLLQHIIGL
KD: 9.30e−09
Kass: 1.13e+05, kdiss: 1.05e−03
1981_ID.3807_Q6_SLLQHQIGL
KD: 8.51e−09
Kass: 1.23e+05, kdiss: 1.05e−03
1982_ID.3808_Q5_SLLQQLIGL
KD: 2.76e−07
Kass: 8.96e+04, kdiss: 2.47e−02
1983_ID.3809_A3_SLAQHLIGL
KD: 1.54e−08
Kass: 1.29e+05, kdiss: 1.99e−03
1984_ID.381_P3_SLPQHLIGL
KD: 2.99e−09
Kass: 1.14e+05, kdiss: 3.41e−04
1985_ID.3812_I6_SLLQHIIGL
KD: 1.19e−08
Kass: 1.03e+05, kdiss: 1.23e−03
1986_ID.3813_V7_SLLQHLVGL
KD: 9.56e−09
Kass: 9.88e+04, kdiss: 9.45e−04
1987_ID.3814_D3_SLDQHLIGL
KD: 9.88e−09
Kass: 1.09e+05, kdiss: 1.07e−03
1988_ID.3815_Q6_SLLQHQIGL
KD: 4.11e−08
Kass: 1.05e+05, kdiss: 4.30e−03
1989_ID.3816_V6_SLLQHVIGL
KD: 9.37e−09
Kass: 1.06e+05, kdiss: 9.94e−04
1990_ID.3817_M7_SLLQHLMGL
KD: 1.12e−08
Kass: 1.16e+05, kdiss: 1.30e−03
1991_ID.3818_G6_SLLQHGIGL
KD: 1.81e−08
Kass: 9.17e+04, kdiss: 1.66e−03
1992_ID.3819_M7_SLLQHLMGL
KD: 1.62e−08
Kass: 1.05e+05, kdiss: 1.70e−03
1993_ID.3821_K1_KLLQHLIGL
KD: 1.54e−08
Kass: 1.16e+05, kdiss: 1.78e−03
1994_ID.3822_N4_SLLNHLIGL
KD: 1.27e−08
Kass: 9.12e+04, kdiss: 1.16e−03
1995_ID.3824_K5_SLLQKLIGL
KD: 7.70e−07
Kass: 2.31e+04, kdiss: 1.78e−02
1996_ID.3825_P7_SLLQHLPGL
KD: 1.04e−06
Kass: 2.20e+04, kdiss: 2.29e−02
1997_ID.3826_E3_SLEQHLIGL
KD: 1.06e−08
Kass: 9.36e+04, kdiss: 9.97e−04
1998_ID.3827_A1_ALLQHLIGL
KD: 1.96e−08
Kass: 8.35e+04, kdiss: 1.64e−03
1999_ID.3828_M5_SLLQMLIGL
KD: 1.88e−06
Kass: 1.33e+04, kdiss: 2.50e−02
2000_ID.3829_E6_SLLQHEIGL
KD: 1.99e−07
Kass: 4.70e+04, kdiss: 9.36e−03
value
0
5000
10000
15000
20000
0
250
500
750
1000
Time

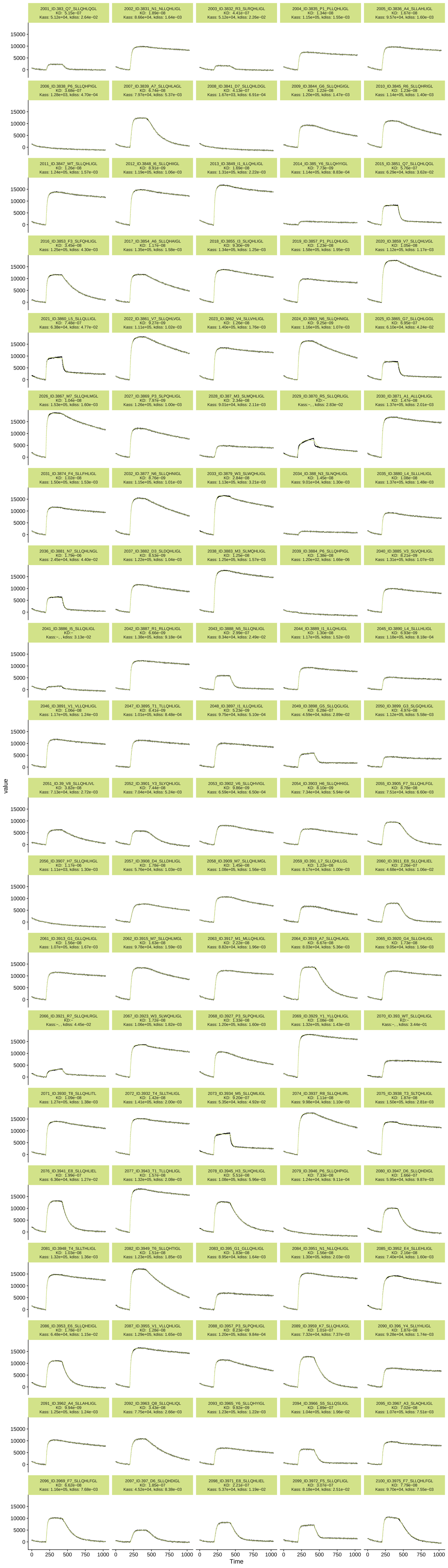

2001_ID.383_Q7_SLLQHLQGL KD: 5.15e-07 Kass: 5.12e+04, kdiss: 2.64e-02
2002_ID.3831_N1_NLLQHLIGL KD: 1.89e-08 Kass: 8.66e+04, kdiss: 1.64e-03
2003_ID.3832_R3_SLRQHLIGL KD: 4.41e-07 Kass: 5.12e+04, kdiss: 2.26e-02
2004_ID.3835_P1_PLLQHLIGL KD: 1.34e-08 Kass: 1.15e+05, kdiss: 1.55e-03
2005_ID.3836_A4_SLLAHLIGL KD: 1.67e-08 Kass: 9.57e+04, kdiss: 1.60e-03
2006_ID.3838_P6_SLLQHPIGL KD: 3.68e-07 Kass: 1.28e+03, kdiss: 4.70e-04
2007_ID.3839_A7_SLLQHLAGL KD: 6.74e-08 Kass: 7.97e+04, kdiss: 5.37e-03
2008_ID.3841_D7_SLLQHLDGL KD: 4.13e-07 Kass: 1.67e+03, kdiss: 6.91e-04
2009_ID.3844_G6_SLLQHGIGL KD: 1.22e-08 Kass: 1.20e+05, kdiss: 1.47e-03
2010_ID.3845_R6_SLLQHRIGL KD: 1.23e-08 Kass: 1.14e+05, kdiss: 1.40e-03
2011_ID.3847_WT_SLLQHLIGL KD: 1.26e-08 Kass: 1.24e+05, kdiss: 1.57e-03
2012_ID.3848_I6_SLLQHIIGL KD: 8.91e-09 Kass: 1.19e+05, kdiss: 1.06e-03
2013_ID.3849_I1_ILLQHLIGL KD: 1.69e-08 Kass: 1.31e+05, kdiss: 2.22e-03
2014_ID.385_Y6_SLLQHYIGL KD: 7.73e-09 Kass: 1.14e+05, kdiss: 8.83e-04
2015_ID.3851_Q7_SLLQHLQGL KD: 5.76e-07 Kass: 6.29e+04, kdiss: 3.62e-02
2016_ID.3853_F3_SLFQHLIGL KD: 3.46e-08 Kass: 1.25e+05, kdiss: 4.30e-03
2017_ID.3854_A6_SLLQHAIGL KD: 1.17e-08 Kass: 1.35e+05, kdiss: 1.58e-03
2018_ID.3855_I3_SLIQHLIGL KD: 9.30e-09 Kass: 1.34e+05, kdiss: 1.25e-03
2019_ID.3857_P1_PLLQHLIGL KD: 1.23e-08 Kass: 1.58e+05, kdiss: 1.95e-03
2020_ID.3859_V7_SLLQHLVGL KD: 1.05e-08 Kass: 1.12e+05, kdiss: 1.17e-03
2021_ID.3860_L5_SLLQLLIGL KD: 7.48e-07 Kass: 6.38e+04, kdiss: 4.77e-02
2022_ID.3861_V7_SLLQHLVGL KD: 9.27e-09 Kass: 1.11e+05, kdiss: 1.02e-03
2023_ID.3862_V4_SLLVHLIGL KD: 1.26e-08 Kass: 1.40e+05, kdiss: 1.76e-03
2024_ID.3863_N6_SLLQHNIGL KD: 9.25e-09 Kass: 1.16e+05, kdiss: 1.07e-03
2025_ID.3865_G7_SLLQHLGGL KD: 6.95e-07 Kass: 6.10e+04, kdiss: 4.24e-02
2026_ID.3867_M7_SLLQHLMGL KD: 1.04e-08 Kass: 1.53e+05, kdiss: 1.60e-03
2027_ID.3869_P3_SLPQHLIGL KD: 7.97e-09 Kass: 1.26e+05, kdiss: 1.00e-03
2028_ID.387_M3_SLMQHLIGL KD: 2.34e-08 Kass: 9.01e+04, kdiss: 2.11e-03
2029_ID.3870_R5_SLLQRLIGL KD: ~ Kass: ~, , kdiss: 2.83e-02
2030_ID.3871_A1_ALLQHLIGL KD: 1.47e-08 Kass: 1.37e+05, kdiss: 2.01e-03
2031_ID.3874_F4_SLLFHLIGL KD: 1.02e-08 Kass: 1.50e+05, kdiss: 1.53e-03
2032_ID.3877_N6_SLLQHNIGL KD: 8.76e-09 Kass: 1.15e+05, kdiss: 1.01e-03
2033_ID.3879_W3_SLWQHLIGL KD: 2.84e-08 Kass: 1.13e+05, kdiss: 3.21e-03
2034_ID.388_N3_SLNQHLIGL KD: 1.45e-08 Kass: 9.01e+04, kdiss: 1.30e-03
2035_ID.3880_L4_SLLLHLIGL KD: 1.08e-08 Kass: 1.37e+05, kdiss: 1.48e-03
2036_ID.3881_N7_SLLQHLNGL KD: 1.79e-06 Kass: 2.45e+04, kdiss: 4.40e-02
2037_ID.3882_D3_SLDQHLIGL KD: 8.53e-09 Kass: 1.22e+05, kdiss: 1.04e-03
2038_ID.3883_M3_SLMQHLIGL KD: 1.25e-08 Kass: 1.25e+05, kdiss: 1.57e-03
2039_ID.3884_P6_SLLQHPIGL KD: 1.38e-08 Kass: 1.20e+02, kdiss: 1.66e-06
2040_ID.3885_V3_SLVQHLIGL KD: 8.21e-09 Kass: 1.31e+05, kdiss: 1.07e-03
2041_ID.3886_I5_SLLQILIGL KD: ~ Kass: ~, , kdiss: 3.13e-02
2042_ID.3887_R1_RLLQHLIGL KD: 6.66e-09 Kass: 1.38e+05, kdiss: 9.18e-04
2043_ID.3888_N5_SLLQNLIGL KD: 2.99e-07 Kass: 8.34e+04, kdiss: 2.49e-02
2044_ID.3889_I1_ILLQHLIGL KD: 1.30e-08 Kass: 1.17e+05, kdiss: 1.52e-03
2045_ID.3890_L4_SLLLHLIGL KD: 6.93e-09 Kass: 1.18e+05, kdiss: 8.18e-04
2046_ID.3891_V1_VLLQHLIGL KD: 1.06e-08 Kass: 1.17e+05, kdiss: 1.24e-03
2047_ID.3895_T1_TLLQHLIGL KD: 8.41e-09 Kass: 1.01e+05, kdiss: 8.48e-04
2048_ID.3897_I1_ILLQHLIGL KD: 5.23e-09 Kass: 9.75e+04, kdiss: 5.10e-04
2049_ID.3898_G5_SLLQGLIGL KD: 6.28e-07 Kass: 4.59e+04, kdiss: 2.89e-02
2050_ID.3899_G3_SLGQHLIGL KD: 4.97e-08 Kass: 1.12e+05, kdiss: 5.58e-03
2051_ID.39_V8_SLLQHLIVL KD: 3.82e-08 Kass: 7.13e+04, kdiss: 2.72e-03
2052_ID.3901_Y3_SLYQHLIGL KD: 7.44e-08 Kass: 7.04e+04, kdiss: 5.24e-03
2053_ID.3902_V6_SLLQHVIGL KD: 9.86e-09 Kass: 6.59e+04, kdiss: 6.50e-04
2054_ID.3903_H6_SLLQHHIGL KD: 8.10e-09 Kass: 7.34e+04, kdiss: 5.94e-04
2055_ID.3905_F7_SLLQHLFGL KD: 8.78e-08 Kass: 7.51e+04, kdiss: 6.60e-03
2056_ID.3907_H7_SLLQHLHGL KD: 1.17e-06 Kass: 1.11e+03, kdiss: 1.30e-03
2057_ID.3908_D4_SLLDHLIGL KD: 1.78e-08 Kass: 5.76e+04, kdiss: 1.03e-03
2058_ID.3909_M7_SLLQHLMGL KD: 1.45e-08 Kass: 1.08e+05, kdiss: 1.56e-03
2059_ID.391_L7_SLLQHLLGL KD: 1.22e-08 Kass: 8.17e+04, kdiss: 1.00e-03
2060_ID.3911_E8_SLLQHLIEL KD: 2.26e-07 Kass: 4.68e+04, kdiss: 1.06e-02
2061_ID.3913_G1_GLLQHLIGL KD: 1.56e-08 Kass: 1.07e+05, kdiss: 1.67e-03
2062_ID.3915_M7_SLLQHLMGL KD: 1.63e-08 Kass: 9.78e+04, kdiss: 1.59e-03
2063_ID.3917_M1_MLLQHLIGL KD: 2.22e-08 Kass: 8.82e+04, kdiss: 1.96e-03
2064_ID.3919_A7_SLLQHLAGL KD: 6.67e-08 Kass: 8.03e+04, kdiss: 5.36e-03
2065_ID.3920_G4_SLLGHLIGL KD: 1.72e-08 Kass: 9.05e+04, kdiss: 1.56e-03
2066_ID.3921_R7_SLLQHLRGL KD: ~ Kass: ~, , kdiss: 4.45e-02
2067_ID.3923_W3_SLWQHLIGL KD: 1.72e-08 Kass: 1.06e+05, kdiss: 1.82e-03
2068_ID.3927_P3_SLPQHLIGL KD: 1.33e-08 Kass: 1.20e+05, kdiss: 1.60e-03
2069_ID.3929_Y1_YLLQHLIGL KD: 1.08e-08 Kass: 1.32e+05, kdiss: 1.43e-03
2070_ID.393_WT_SLLQHLIGL KD: ~ Kass: ~, , kdiss: 3.44e-01
2071_ID.3930_T8_SLLQHLITL KD: 1.09e-08 Kass: 1.27e+05, kdiss: 1.38e-03
2072_ID.3932_T4_SLLTHLIGL KD: 1.42e-08 Kass: 1.41e+05, kdiss: 2.00e-03
2073_ID.3934_M5_SLLQMLIGL KD: 9.20e-07 Kass: 5.35e+04, kdiss: 4.92e-02
2074_ID.3937_R8_SLLQHLIRL KD: 1.11e-08 Kass: 9.98e+04, kdiss: 1.10e-03
2075_ID.3938_T3_SLTQHLIGL KD: 1.87e-08 Kass: 1.50e+05, kdiss: 2.81e-03
2076_ID.3941_E8_SLLQHLIEL KD: 1.99e-07 Kass: 6.36e+04, kdiss: 1.27e-02
2077_ID.3943_T1_TLLQHLIGL KD: 1.57e-08 Kass: 1.32e+05, kdiss: 2.08e-03
2078_ID.3945_H3_SLHQHLIGL KD: 5.51e-08 Kass: 1.08e+05, kdiss: 5.96e-03
2079_ID.3946_P6_SLLQHPIGL KD: 7.33e-08 Kass: 1.24e+04, kdiss: 9.11e-04
2080_ID.3947_D6_SLLQHDIGL KD: 1.66e-07 Kass: 5.95e+04, kdiss: 9.87e-03
2081_ID.3948_T4_SLLTHLIGL KD: 1.03e-08 Kass: 1.32e+05, kdiss: 1.36e-03
2082_ID.3949_T6_SLLQHTIGL KD: 1.51e-08 Kass: 1.23e+05, kdiss: 1.85e-03
2083_ID.395_G1_GLLQHLIGL KD: 1.83e-08 Kass: 8.95e+04, kdiss: 1.64e-03
2084_ID.3951_N1_NLLQHLIGL KD: 1.56e-08 Kass: 1.30e+05, kdiss: 2.03e-03
2085_ID.3952_E4_SLLEHLIGL KD: 2.16e-08 Kass: 7.40e+04, kdiss: 1.60e-03
2086_ID.3953_E6_SLLQHEIGL KD: 1.78e-07 Kass: 6.48e+04, kdiss: 1.15e-02
2087_ID.3955_V1_VLLQHLIGL KD: 1.28e-08 Kass: 1.29e+05, kdiss: 1.65e-03
2088_ID.3957_P3_SLPQHLIGL KD: 8.23e-09 Kass: 1.20e+05, kdiss: 9.84e-04
2089_ID.3959_K7_SLLQHLKGL KD: 1.01e-07 Kass: 7.32e+04, kdiss: 7.37e-03
2090_ID.396_Y4_SLLYHLIGL KD: 1.87e-08 Kass: 9.28e+04, kdiss: 1.74e-03
2091_ID.3962_A4_SLLAHLIGL KD: 9.94e-09 Kass: 1.25e+05, kdiss: 1.24e-03
2092_ID.3963_Q8_SLLQHLIQL KD: 3.43e-08 Kass: 7.75e+04, kdiss: 2.66e-03
2093_ID.3965_Y6_SLLQHYIGL KD: 9.92e-09 Kass: 1.23e+05, kdiss: 1.22e-03
2094_ID.3966_S5_SLLQSLIGL KD: 1.89e-07 Kass: 1.04e+05, kdiss: 1.96e-02
2095_ID.3967_A3_SLAQHLIGL KD: 7.02e-08 Kass: 1.07e+05, kdiss: 7.51e-03
2096_ID.3969_F7_SLLQHLFGL KD: 6.62e-08 Kass: 1.16e+05, kdiss: 7.68e-03
2097_ID.397_D6_SLLQHDIGL KD: 1.85e-07 Kass: 4.52e+04, kdiss: 8.38e-03
2098_ID.3971_E8_SLLQHLIEL KD: 2.21e-07 Kass: 5.37e+04, kdiss: 1.19e-02
2099_ID.3972_F5_SLLQFLIGL KD: 3.07e-07 Kass: 8.18e+04, kdiss: 2.51e-02
2100_ID.3975_F7_SLLQHLFGL KD: 7.79e-08 Kass: 9.70e+04, kdiss: 7.55e-03
value
15000
10000
5000
0
0
250
500
750
1000
Time

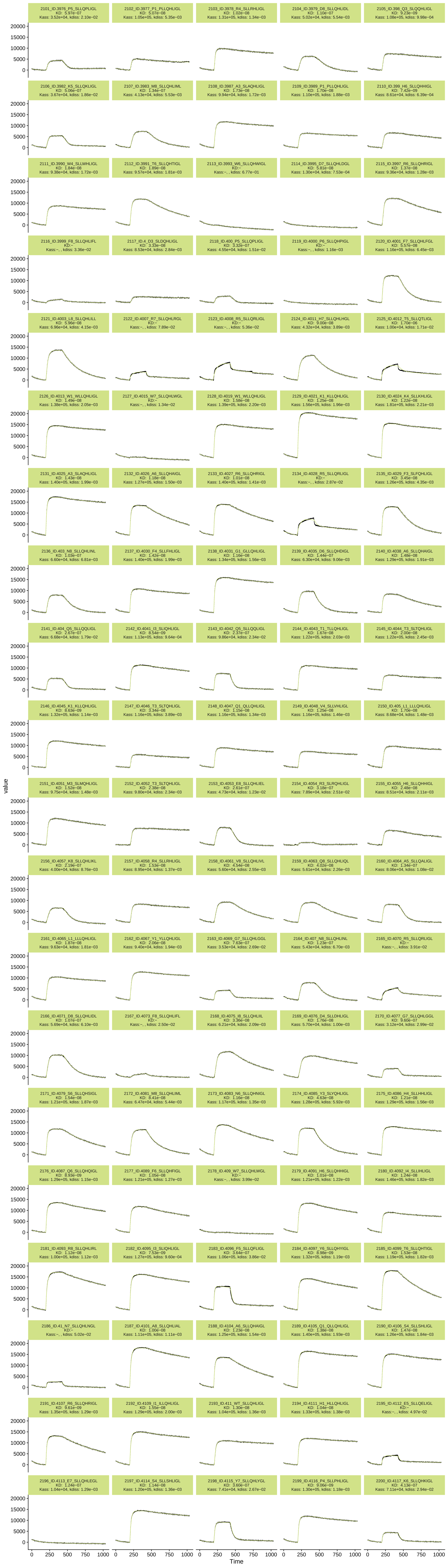
value
Time
0
250
500
750
1000
0
5000
10000
15000
20000

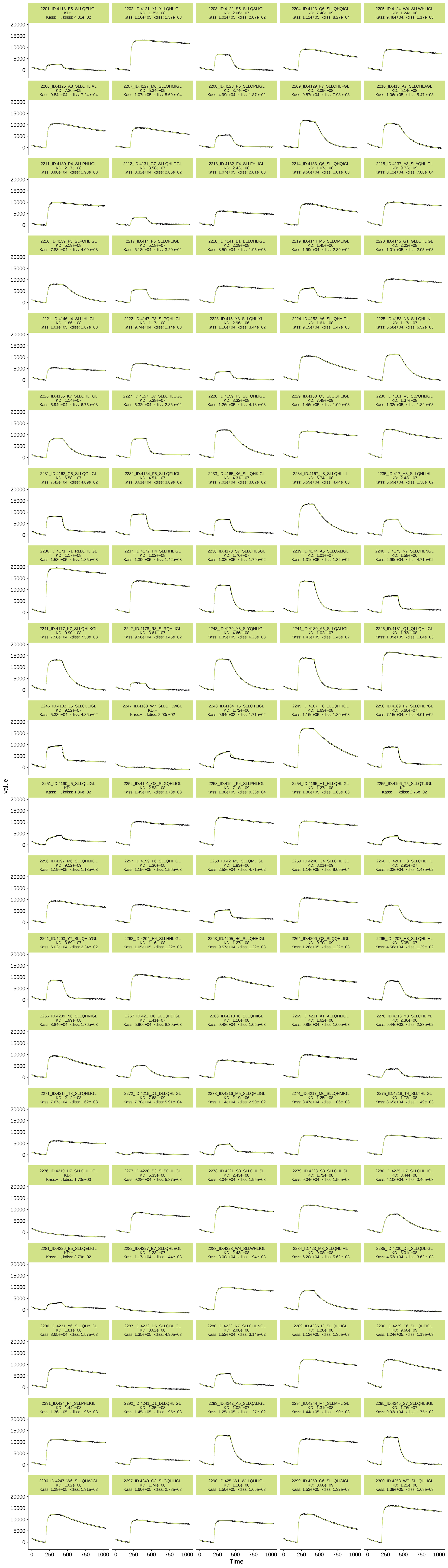
value
Time

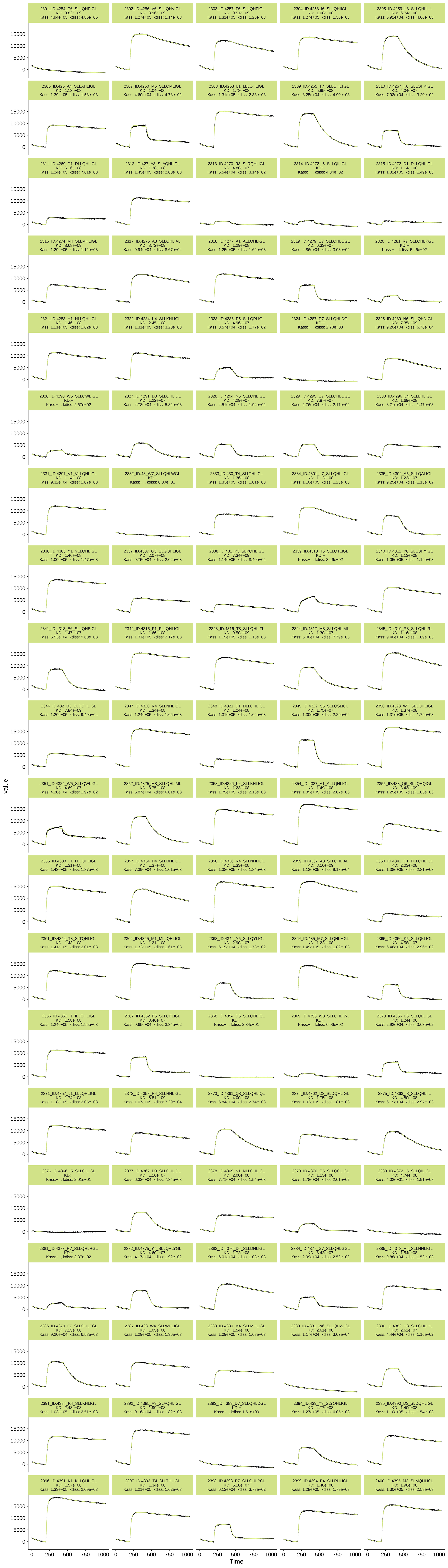

value
Time
0
250
500
750
1000
0
5000
10000
15000

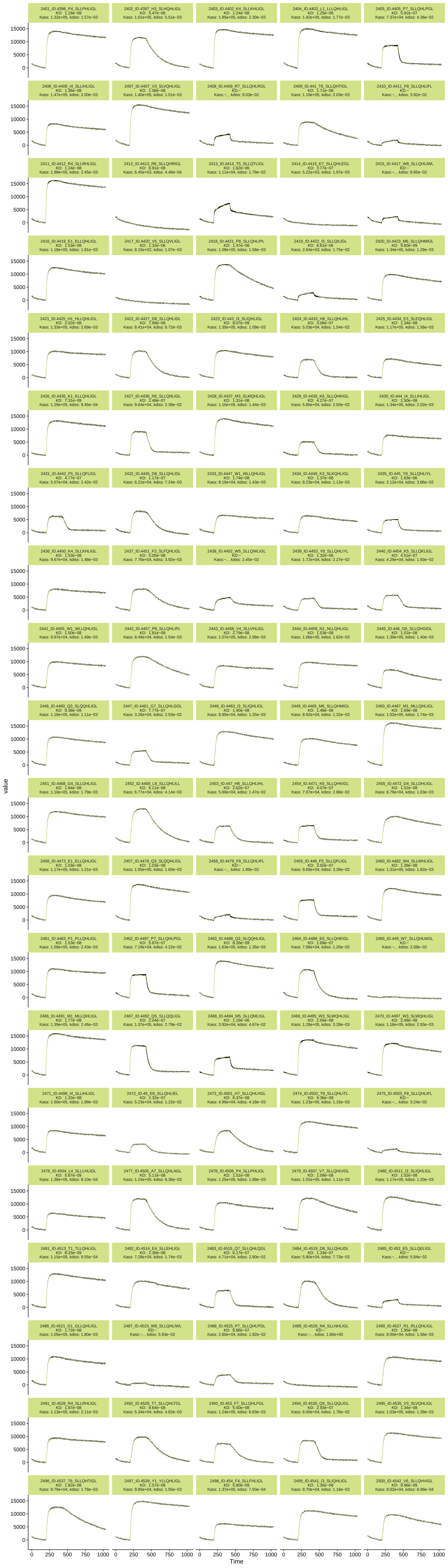
2401_ID.4396_P4_SLLPHLIGL KD: 1.19e−08 Kass: 1.32e+05, kdiss: 1.57e−03
2402_ID.4397_H3_SLLQHLIGL KD: 5.47e−08 Kass: 1.01e+05, kdiss: 5.51e−03
2403_ID.4402_K4_SLLKHLIGL KD: 1.24e−08 Kass: 1.85e+05, kdiss: 2.30e−03
2404_ID.4403_L1_LLLQHLIGL KD: 1.25e−08 Kass: 1.42e+05, kdiss: 1.77e−03
2405_ID.4405_P7_SLLQHLPGL KD: 5.91e−07 Kass: 7.37e+04, kdiss: 4.36e−02
2406_ID.4406_I4_SLLIHLIGL KD: 1.36e−08 Kass: 1.47e+05, kdiss: 2.00e−03
2407_ID.4407_V3_SLVQHLIGL KD: 1.08e−08 Kass: 1.40e+05, kdiss: 1.51e−03
2408_ID.4409_R7_SLLQHLRGL KD:~ Kass:~ , kdiss: 9.03e−02
2409_ID.441_T6_SLLQHTIGL KD: 1.71e−08 Kass: 1.19e+05, kdiss: 2.03e−03
2410_ID.4411_F8_SLLQHLIFL KD:~ Kass:~ , kdiss: 3.92e−02
2411_ID.4412_R4_SLLRHLIGL KD: 1.24e−08 Kass: 1.98e+05, kdiss: 2.45e−03
2412_ID.4413_R6_SLLQHRIGL KD: 6.91e−08 Kass: 6.45e+03, kdiss: 4.46e−04
2413_ID.4414_T5_SLLQTLIGL KD: 1.62e−06 Kass: 1.11e+04, kdiss: 1.79e−02
2414_ID.4415_E7_SLLQHLEGL KD: 3.77e−07 Kass: 5.22e+03, kdiss: 1.97e−03
2415_ID.4417_W8_SLLQHLIWL KD:~ Kass:~ , kdiss: 9.65e−02
2416_ID.4419_E1_ELLQHLIGL KD: 1.53e−08 Kass: 1.18e+05, kdiss: 1.81e−03
2417_ID.4420_V5_SLLQVLIGL KD: 1.31e−06 Kass: 8.15e+02, kdiss: 1.07e−03
2418_ID.4421_P8_SLLQHLIPL KD: 1.47e−08 Kass: 1.08e+05, kdiss: 1.58e−03
2419_ID.4422_I5_SLLQILIGL KD: 6.61e−06 Kass: 2.64e+03, kdiss: 1.75e−02
2420_ID.4423_M6_SLLQHMIGL KD: 9.60e−09 Kass: 1.34e+05, kdiss: 1.29e−03
2421_ID.4425_H1_HLLQHLIGL KD: 2.02e−08 Kass: 1.33e+05, kdiss: 2.69e−03
2422_ID.4427_D8_SLLQHLIDL KD: 7.99e−08 Kass: 8.41e+04, kdiss: 6.72e−03
2423_ID.443_I3_SLIQHLIGL KD: 8.07e−09 Kass: 1.35e+05, kdiss: 1.09e−03
2424_ID.4433_H8_SLLQHLIHL KD: 3.06e−07 Kass: 5.03e+04, kdiss: 1.54e−02
2425_ID.4434_E3_SLEQHLIGL KD: 1.34e−08 Kass: 1.17e+05, kdiss: 1.56e−03
2426_ID.4435_K1_KLLQHLIGL KD: 7.31e−09 Kass: 1.29e+05, kdiss: 9.45e−04
2427_ID.4436_N5_SLLQNLIGL KD: 2.48e−07 Kass: 9.64e+04, kdiss: 2.39e−02
2428_ID.4437_M3_SLMQHLIGL KD: 1.31e−08 Kass: 1.10e+05, kdiss: 1.44e−03
2429_ID.4439_K6_SLLQHKIGL KD: 4.27e−07 Kass: 5.85e+04, kdiss: 2.50e−02
2430_ID.444_I4_SLLIHLIGL KD: 1.50e−08 Kass: 1.34e+05, kdiss: 2.02e−03
2431_ID.4442_F5_SLLQFLIGL KD: 4.77e−07 Kass: 5.07e+04, kdiss: 2.42e−02
2432_ID.4445_D8_SLLQHLIDL KD: 1.17e−07 Kass: 6.21e+04, kdiss: 7.24e−03
2433_ID.4447_W1_WLLQHLIGL KD: 1.74e−08 Kass: 8.19e+04, kdiss: 1.43e−03
2434_ID.4449_K3_SLKQHLIGL KD: 1.37e−08 Kass: 8.23e+04, kdiss: 1.13e−03
2435_ID.445_Y8_SLLQHLIYL KD: 1.83e−06 Kass: 2.12e+04, kdiss: 3.86e−02
2436_ID.4450_K4_SLLKHLIGL KD: 1.53e−08 Kass: 9.67e+04, kdiss: 1.48e−03
2437_ID.4451_F3_SLFQHLIGL KD: 5.05e−08 Kass: 7.76e+04, kdiss: 3.92e−03
2438_ID.4452_W5_SLLQWLIGL KD:~ Kass:~ , kdiss: 2.45e−02
2439_ID.4453_Y8_SLLQHLIYL KD: 1.32e−06 Kass: 1.72e+04, kdiss: 2.27e−02
2440_ID.4454_K5_SLLQKLIGL KD: 4.51e−07 Kass: 4.29e+04, kdiss: 1.93e−02
2441_ID.4455_W1_WLLQHLIGL KD: 1.50e−08 Kass: 9.97e+04, kdiss: 1.49e−03
2442_ID.4457_P8_SLLQHLIPL KD: 1.81e−08 Kass: 8.49e+04, kdiss: 1.54e−03
2443_ID.4458_V4_SLLVHLIGL KD: 2.79e−08 Kass: 1.07e+05, kdiss: 2.98e−03
2444_ID.4459_N1_NLLQHLIGL KD: 1.53e−08 Kass: 1.06e+05, kdiss: 1.62e−03
2445_ID.446_G6_SLLQHGIGL KD: 1.01e−08 Kass: 1.39e+05, kdiss: 1.40e−03
2446_ID.4460_Q3_SLQQHLIGL KD: 9.38e−09 Kass: 1.18e+05, kdiss: 1.11e−03
2447_ID.4461_G7_SLLQHLGGL KD: 7.77e−07 Kass: 3.26e+04, kdiss: 2.53e−02
2448_ID.4463_I3_SLIQHLIGL KD: 1.40e−08 Kass: 8.95e+04, kdiss: 1.25e−03
2449_ID.4465_M6_SLLQHMIGL KD: 1.48e−08 Kass: 8.92e+04, kdiss: 1.32e−03
2450_ID.4467_M1_MLLQHLIGL KD: 1.69e−08 Kass: 1.03e+05, kdiss: 1.74e−03
2451_ID.4468_G4_SLLGHLIGL KD: 1.64e−08 Kass: 1.10e+05, kdiss: 1.79e−03
2452_ID.4469_L8_SLLQHLILL KD: 6.11e−08 Kass: 6.77e+04, kdiss: 4.14e−03
2453_ID.447_H8_SLLQHLIHL KD: 2.62e−07 Kass: 5.60e+04, kdiss: 1.47e−02
2454_ID.4471_K6_SLLQHKIGL KD: 4.07e−07 Kass: 7.07e+04, kdiss: 2.88e−02
2455_ID.4472_D4_SLLDHLIGL KD: 1.52e−08 Kass: 6.79e+04, kdiss: 1.03e−03
2456_ID.4473_E1_ELLQHLIGL KD: 1.03e−08 Kass: 1.17e+05, kdiss: 1.21e−03
2457_ID.4478_Q3_SLQQHLIGL KD: 1.03e−08 Kass: 1.55e+05, kdiss: 1.60e−03
2458_ID.4479_F8_SLLQHLIFL KD:~ Kass:~ , kdiss: 1.89e−02
2459_ID.448_F5_SLLQFLIGL KD: 3.50e−07 Kass: 9.69e+04, kdiss: 3.39e−02
2460_ID.4482_W4_SLLWHLIGL KD: 1.39e−08 Kass: 1.31e+05, kdiss: 1.82e−03
2461_ID.4483_P1_PLLQHLIGL KD: 1.53e−08 Kass: 1.59e+05, kdiss: 2.43e−03
2462_ID.4487_P7_SLLQHLPGL KD: 5.87e−07 Kass: 7.19e+04, kdiss: 4.22e−02
2463_ID.4488_Q3_SLQQHLIGL KD: 8.26e−09 Kass: 1.63e+05, kdiss: 1.35e−03
2464_ID.4489_E6_SLLQHEIGL KD: 1.69e−07 Kass: 7.08e+04, kdiss: 1.20e−02
2465_ID.449_W7_SLLQHLWGL KD:~ Kass:~ , kdiss: 2.08e−02
2466_ID.4491_M1_MLLQHLIGL KD: 1.77e−08 Kass: 1.39e+05, kdiss: 2.46e−03
2467_ID.4492_Q5_SLLQQLIGL KD: 2.04e−07 Kass: 1.37e+05, kdiss: 2.79e−02
2468_ID.4494_M5_SLLQMLIGL KD: 1.19e−06 Kass: 3.92e+04, kdiss: 4.67e−02
2469_ID.4495_W3_SLWQHLIGL KD: 2.56e−08 Kass: 1.28e+05, kdiss: 3.28e−03
2470_ID.4497_W3_SLWQHLIGL KD: 2.48e−08 Kass: 1.18e+05, kdiss: 2.93e−03
2471_ID.4498_I4_SLLIHLIGL KD: 1.33e−08 Kass: 1.50e+05, kdiss: 1.99e−03
2472_ID.45_E8_SLLQHLIEL KD: 2.33e−07 Kass: 5.23e+04, kdiss: 1.22e−02
2473_ID.4501_H7_SLLQHLHGL KD: 8.37e−08 Kass: 4.99e+04, kdiss: 4.18e−03
2474_ID.4502_T8_SLLQHLITL KD: 9.36e−09 Kass: 1.23e+05, kdiss: 1.15e−03
2475_ID.4503_F8_SLLQHLIFL KD:~ Kass:~ , kdiss: 3.24e−02
2476_ID.4504_L4_SLLLHLIGL KD: 5.87e−09 Kass: 1.38e+05, kdiss: 8.10e−04
2477_ID.4505_A7_SLLQHLAGL KD: 5.11e−08 Kass: 1.24e+05, kdiss: 6.36e−03
2478_ID.4506_P4_SLLPHLIGL KD: 1.51e−08 Kass: 1.25e+05, kdiss: 1.88e−03
2479_ID.4507_V7_SLLQHLVGL KD: 1.09e−08 Kass: 1.02e+05, kdiss: 1.11e−03
2480_ID.4511_I3_SLIQHLIGL KD: 1.02e−08 Kass: 1.17e+05, kdiss: 1.20e−03
2481_ID.4513_T1_TLLQHLIGL KD: 8.33e−09 Kass: 1.15e+05, kdiss: 9.55e−04
2482_ID.4514_E4_SLLEHLIGL KD: 2.39e−08 Kass: 7.28e+04, kdiss: 1.74e−03
2483_ID.4515_Q7_SLLQHLQGL KD: 6.17e−07 Kass: 4.71e+04, kdiss: 2.90e−02
2484_ID.4519_D8_SLLQHLIDL KD: 1.33e−07 Kass: 5.80e+04, kdiss: 7.73e−03
2485_ID.452_E5_SLLQELIGL KD:~ Kass:~ , kdiss: 5.84e−02
2486_ID.4521_G1_GLLQHLIGL KD: 1.72e−08 Kass: 1.05e+05, kdiss: 1.80e−03
2487_ID.4523_W8_SLLQHLIWL KD:~ Kass:~ , kdiss: 5.93e−02
2488_ID.4525_P7_SLLQHLPGL KD: 6.86e−07 Kass: 2.80e+04, kdiss: 1.92e−02
2489_ID.4526_N4_SLLNHLIGL KD:~ Kass:~ , kdiss: 1.60e+00
2490_ID.4527_R1_RLLQHLIGL KD: 1.95e−08 Kass: 8.00e+04, kdiss: 1.56e−03
2491_ID.4528_R4_SLLRHLIGL KD: 1.87e−08 Kass: 1.13e+05, kdiss: 2.11e−03
2492_ID.4529_T7_SLLQHLTGL KD: 8.64e−08 Kass: 5.34e+04, kdiss: 4.62e−03
2493_ID.453_F7_SLLQHLFGL KD: 5.50e−08 Kass: 1.24e+05, kdiss: 6.83e−03
2494_ID.4530_Q5_SLLQQLIGL KD: 2.93e−07 Kass: 6.00e+04, kdiss: 1.76e−02
2495_ID.4535_V3_SLVQHLIGL KD: 1.34e−08 Kass: 1.03e+05, kdiss: 1.39e−03
2496_ID.4537_T6_SLLQHTIGL KD: 1.82e−08 Kass: 9.79e+04, kdiss: 1.78e−03
2497_ID.4539_Y1_YLLQHLIGL KD: 1.57e−08 Kass: 9.85e+04, kdiss: 1.55e−03
2498_ID.454_F4_SLLFHLIGL KD: 5.80e−09 Kass: 1.37e+05, kdiss: 7.93e−04
2499_ID.4541_I3_SLIQHLIGL KD: 1.36e−08 Kass: 8.70e+04, kdiss: 1.18e−03
2500_ID.4542_V6_SLLQHVIGL KD: 9.96e−09 Kass: 9.02e+04, kdiss: 8.99e−04
value
Time
0 250 500 750 1000
0 5000 10000 15000

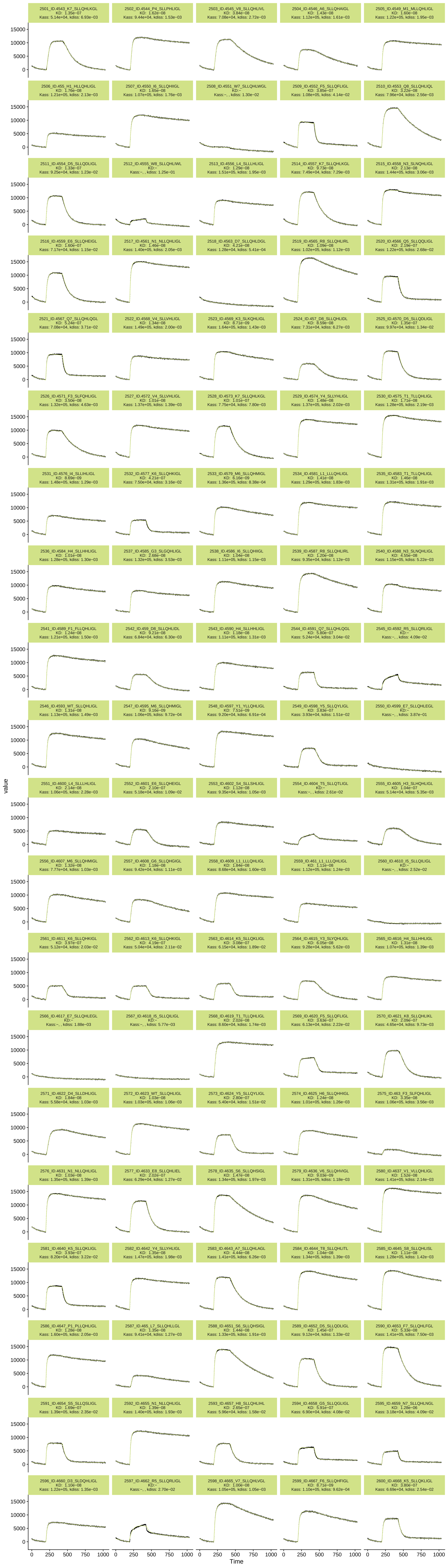

value
Time

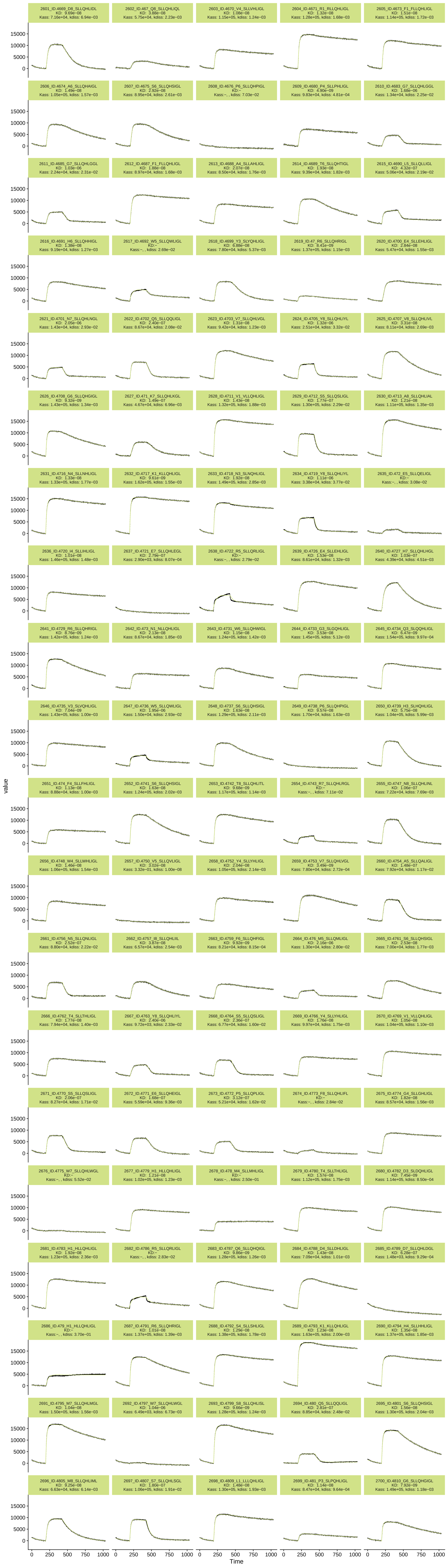
2601_ID.4669_D8_SLLQHLIDL
KD: 9.69e−08
Kass: 7.16e+04, kdiss: 6.94e−03
2602_ID.467_Q8_SLLQHLIQL
KD: 3.88e−08
Kass: 5.75e+04, kdiss: 2.23e−03
2603_ID.4670_V4_SLLVHLIGL
KD: 1.08e−08
Kass: 1.15e+05, kdiss: 1.24e−03
2604_ID.4671_R1_RLLQHLIGL
KD: 1.32e−08
Kass: 1.28e+05, kdiss: 1.68e−03
2605_ID.4673_F1_FLLQHLIGL
KD: 1.51e−08
Kass: 1.14e+05, kdiss: 1.72e−03
2606_ID.4674_A6_SLLQHAIGL
KD: 1.49e−08
Kass: 1.05e+05, kdiss: 1.57e−03
2607_ID.4675_S6_SLLQHSIGL
KD: 2.92e−08
Kass: 8.95e+04, kdiss: 2.61e−03
2608_ID.4676_P6_SLLQHPIGL
KD:−
Kass:−, , kdiss: 7.03e−02
2609_ID.4680_P4_SLLPHLIGL
KD: 4.90e−09
Kass: 9.83e+04, kdiss: 4.81e−04
2610_ID.4683_G7_SLLQHLGGL
KD: 1.68e−06
Kass: 1.34e+04, kdiss: 2.25e−02
2611_ID.4685_G7_SLLQHLGGL
KD: 1.03e−06
Kass: 2.24e+04, kdiss: 2.31e−02
2612_ID.4687_F1_FLLQHLIGL
KD: 1.88e−08
Kass: 8.97e+04, kdiss: 1.68e−03
2613_ID.4688_A4_SLLAHLIGL
KD: 2.07e−08
Kass: 8.50e+04, kdiss: 1.76e−03
2614_ID.4689_T6_SLLQHTIGL
KD: 1.93e−08
Kass: 9.39e+04, kdiss: 1.82e−03
2615_ID.4690_L5_SLLQLLIGL
KD: 4.32e−07
Kass: 5.06e+04, kdiss: 2.19e−02
2616_ID.4691_H6_SLLQHHIGL
KD: 1.38e−08
Kass: 9.19e+04, kdiss: 1.27e−03
2617_ID.4692_W5_SLLQWLIGL
KD:−
Kass:−, , kdiss: 2.69e−02
2618_ID.4699_Y3_SLYQHLIGL
KD: 6.88e−08
Kass: 7.80e+04, kdiss: 5.37e−03
2619_ID.47_R6_SLLQHRIGL
KD: 8.41e−09
Kass: 1.37e+05, kdiss: 1.15e−03
2620_ID.4700_E4_SLLEHLIGL
KD: 2.84e−08
Kass: 5.47e+04, kdiss: 1.55e−03
2621_ID.4701_N7_SLLQHLNGL
KD: 2.05e−06
Kass: 1.43e+04, kdiss: 2.93e−02
2622_ID.4702_Q5_SLLQQLIGL
KD: 2.40e−07
Kass: 8.67e+04, kdiss: 2.08e−02
2623_ID.4703_V7_SLLQHLVGL
KD: 1.31e−08
Kass: 9.42e+04, kdiss: 1.23e−03
2624_ID.4705_Y8_SLLQHLIYL
KD: 1.32e−06
Kass: 2.51e+04, kdiss: 3.32e−02
2625_ID.4707_V8_SLLQHLIVL
KD: 3.31e−08
Kass: 8.11e+04, kdiss: 2.69e−03
2626_ID.4708_G6_SLLQHGIGL
KD: 9.32e−09
Kass: 1.43e+05, kdiss: 1.34e−03
2627_ID.471_K7_SLLQHLKGL
KD: 1.49e−07
Kass: 4.67e+04, kdiss: 6.96e−03
2628_ID.4711_V1_VLLQHLIGL
KD: 1.43e−08
Kass: 1.32e+05, kdiss: 1.88e−03
2629_ID.4712_S5_SLLQSLIGL
KD: 1.77e−07
Kass: 1.30e+05, kdiss: 2.29e−02
2630_ID.4713_A8_SLLQHLIAL
KD: 1.21e−08
Kass: 1.11e+05, kdiss: 1.35e−03
2631_ID.4716_N4_SLLNHLIGL
KD: 1.33e−08
Kass: 1.33e+05, kdiss: 1.77e−03
2632_ID.4717_K1_KLLQHLIGL
KD: 9.61e−09
Kass: 1.62e+05, kdiss: 1.55e−03
2633_ID.4718_N3_SLNQHLIGL
KD: 1.92e−08
Kass: 1.49e+05, kdiss: 2.85e−03
2634_ID.4719_Y8_SLLQHLIYL
KD: 1.11e−06
Kass: 3.38e+04, kdiss: 3.77e−02
2635_ID.472_E5_SLLQELIGL
KD:−
Kass:−, , kdiss: 3.08e−02
2636_ID.4720_I4_SLLIHLIGL
KD: 1.01e−08
Kass: 1.46e+05, kdiss: 1.48e−03
2637_ID.4721_E7_SLLQHLEGL
KD: 2.79e−07
Kass: 2.90e+03, kdiss: 8.07e−04
2638_ID.4722_R5_SLLQRLIGL
KD:−
Kass:−, , kdiss: 2.79e−02
2639_ID.4726_E4_SLLEHLIGL
KD: 1.53e−08
Kass: 8.61e+04, kdiss: 1.32e−03
2640_ID.4727_H7_SLLQHLHGL
KD: 1.03e−07
Kass: 4.39e+04, kdiss: 4.51e−03
2641_ID.4729_R6_SLLQHRIGL
KD: 8.76e−09
Kass: 1.42e+05, kdiss: 1.24e−03
2642_ID.473_N1_NLLQHLIGL
KD: 2.13e−08
Kass: 8.67e+04, kdiss: 1.85e−03
2643_ID.4731_W6_SLLQHWIGL
KD: 1.15e−08
Kass: 1.24e+05, kdiss: 1.42e−03
2644_ID.4733_G3_SLGQHLIGL
KD: 3.53e−08
Kass: 1.45e+05, kdiss: 5.12e−03
2645_ID.4734_Q3_SLQQHLIGL
KD: 6.47e−09
Kass: 1.54e+05, kdiss: 9.97e−04
2646_ID.4735_V3_SLVQHLIGL
KD: 7.04e−09
Kass: 1.43e+05, kdiss: 1.00e−03
2647_ID.4736_W5_SLLQWLIGL
KD: 1.95e−06
Kass: 1.50e+04, kdiss: 2.93e−02
2648_ID.4737_S6_SLLQHSIGL
KD: 1.63e−08
Kass: 1.29e+05, kdiss: 2.11e−03
2649_ID.4738_P6_SLLQHPIGL
KD: 9.57e−08
Kass: 1.70e+04, kdiss: 1.63e−03
2650_ID.4739_H3_SLHQHLIGL
KD: 5.75e−08
Kass: 1.04e+05, kdiss: 5.99e−03
2651_ID.474_F4_SLLFHLIGL
KD: 1.13e−08
Kass: 8.88e+04, kdiss: 1.00e−03
2652_ID.4741_S6_SLLQHSIGL
KD: 1.63e−08
Kass: 1.24e+05, kdiss: 2.02e−03
2653_ID.4742_T8_SLLQHLITL
KD: 9.68e−09
Kass: 1.17e+05, kdiss: 1.14e−03
2654_ID.4743_R7_SLLQHLRGL
KD:−
Kass:−, , kdiss: 7.11e−02
2655_ID.4747_N8_SLLQHLINL
KD: 1.06e−07
Kass: 7.22e+04, kdiss: 7.69e−03
2656_ID.4748_W4_SLLWHLIGL
KD: 1.46e−08
Kass: 1.06e+05, kdiss: 1.54e−03
2657_ID.4750_V5_SLLQVLIGL
KD: 3.02e−08
Kass: 3.32e−01, kdiss: 1.00e−08
2658_ID.4752_Y4_SLLYHLIGL
KD: 2.04e−08
Kass: 1.05e+05, kdiss: 2.14e−03
2659_ID.4753_V7_SLLQHLVGL
KD: 3.49e−09
Kass: 7.80e+04, kdiss: 2.72e−04
2660_ID.4754_A5_SLLQALIGL
KD: 1.48e−07
Kass: 7.92e+04, kdiss: 1.17e−02
2661_ID.4756_N5_SLLQNLIGL
KD: 2.52e−07
Kass: 8.80e+04, kdiss: 2.22e−02
2662_ID.4757_I8_SLLQHLIIL
KD: 3.87e−08
Kass: 6.57e+04, kdiss: 2.54e−03
2663_ID.4759_F6_SLLQHFIGL
KD: 9.92e−09
Kass: 8.21e+04, kdiss: 8.15e−04
2664_ID.476_M5_SLLQMLIGL
KD: 2.16e−06
Kass: 1.30e+04, kdiss: 2.80e−02
2665_ID.4761_S6_SLLQHSIGL
KD: 2.53e−08
Kass: 7.00e+04, kdiss: 1.77e−03
2666_ID.4762_T4_SLLTHLIGL
KD: 1.77e−08
Kass: 7.94e+04, kdiss: 1.40e−03
2667_ID.4763_Y8_SLLQHLIYL
KD: 2.40e−06
Kass: 9.72e+03, kdiss: 2.33e−02
2668_ID.4764_S5_SLLQSLIGL
KD: 2.36e−07
Kass: 6.77e+04, kdiss: 1.60e−02
2669_ID.4766_Y4_SLLYHLIGL
KD: 1.76e−08
Kass: 9.97e+04, kdiss: 1.75e−03
2670_ID.4769_V1_VLLQHLIGL
KD: 1.05e−08
Kass: 1.04e+05, kdiss: 1.10e−03
2671_ID.4770_S5_SLLQSLIGL
KD: 2.06e−07
Kass: 8.27e+04, kdiss: 1.71e−02
2672_ID.4771_E6_SLLQHEIGL
KD: 1.68e−07
Kass: 5.59e+04, kdiss: 9.36e−03
2673_ID.4772_P5_SLLQPLIGL
KD: 3.12e−07
Kass: 5.21e+04, kdiss: 1.62e−02
2674_ID.4773_F8_SLLQHLIFL
KD:−
Kass:−, , kdiss: 2.84e−02
2675_ID.4774_G4_SLLGHLIGL
KD: 1.82e−08
Kass: 8.57e+04, kdiss: 1.56e−03
2676_ID.4775_W7_SLLQHLWGL
KD:−
Kass:−, , kdiss: 5.52e−02
2677_ID.4779_H1_HLLQHLIGL
KD: 1.21e−08
Kass: 1.02e+05, kdiss: 1.23e−03
2678_ID.478_M4_SLLMHLIGL
KD:−
Kass:−, , kdiss: 2.50e−01
2679_ID.4780_T4_SLLTHLIGL
KD: 1.57e−08
Kass: 1.12e+05, kdiss: 1.75e−03
2680_ID.4782_D3_SLDQHLIGL
KD: 7.45e−09
Kass: 1.14e+05, kdiss: 8.50e−04
2681_ID.4783_H1_HLLQHLIGL
KD: 1.92e−08
Kass: 1.23e+05, kdiss: 2.36e−03
2682_ID.4786_R5_SLLQRLIGL
KD:−
Kass:−, , kdiss: 2.83e−02
2683_ID.4787_Q6_SLLQHQIGL
KD: 9.86e−09
Kass: 1.28e+05, kdiss: 1.26e−03
2684_ID.4788_D4_SLLDHLIGL
KD: 1.43e−08
Kass: 7.09e+04, kdiss: 1.01e−03
2685_ID.4789_D7_SLLQHLDGL
KD: 6.28e−07
Kass: 1.48e+03, kdiss: 9.29e−04
2686_ID.479_H1_HLLQHLIGL
KD:−
Kass:−, , kdiss: 3.70e−01
2687_ID.4791_R6_SLLQHRIGL
KD: 1.01e−08
Kass: 1.37e+05, kdiss: 1.39e−03
2688_ID.4792_S4_SLLSHLIGL
KD: 1.29e−08
Kass: 1.38e+05, kdiss: 1.78e−03
2689_ID.4793_K1_KLLQHLIGL
KD: 1.23e−08
Kass: 1.63e+05, kdiss: 2.00e−03
2690_ID.4794_H4_SLLHHLIGL
KD: 1.35e−08
Kass: 1.37e+05, kdiss: 1.85e−03
2691_ID.4795_M7_SLLQHLMGL
KD: 1.04e−08
Kass: 1.50e+05, kdiss: 1.56e−03
2692_ID.4797_W7_SLLQHLWGL
KD: 1.04e−06
Kass: 6.49e+03, kdiss: 6.73e−03
2693_ID.4799_S8_SLLQHLISL
KD: 9.66e−09
Kass: 1.28e+05, kdiss: 1.24e−03
2694_ID.480_Q5_SLLQQLIGL
KD: 2.81e−07
Kass: 8.85e+04, kdiss: 2.48e−02
2695_ID.4801_S6_SLLQHSIGL
KD: 1.56e−08
Kass: 1.30e+05, kdiss: 2.04e−03
2696_ID.4805_M8_SLLQHLIML
KD: 9.25e−08
Kass: 6.63e+04, kdiss: 6.14e−03
2697_ID.4807_S7_SLLQHLSGL
KD: 1.80e−07
Kass: 1.06e+05, kdiss: 1.91e−02
2698_ID.4809_L1_LLLQHLIGL
KD: 1.48e−08
Kass: 1.30e+05, kdiss: 1.93e−03
2699_ID.481_P3_SLPQHLIGL
KD: 1.14e−08
Kass: 8.47e+04, kdiss: 9.64e−04
2700_ID.4810_G6_SLLQHGIGL
KD: 7.92e−09
Kass: 1.49e+05, kdiss: 1.18e−03
value
0
5000
10000
15000
Time
0
250
500
750
1000

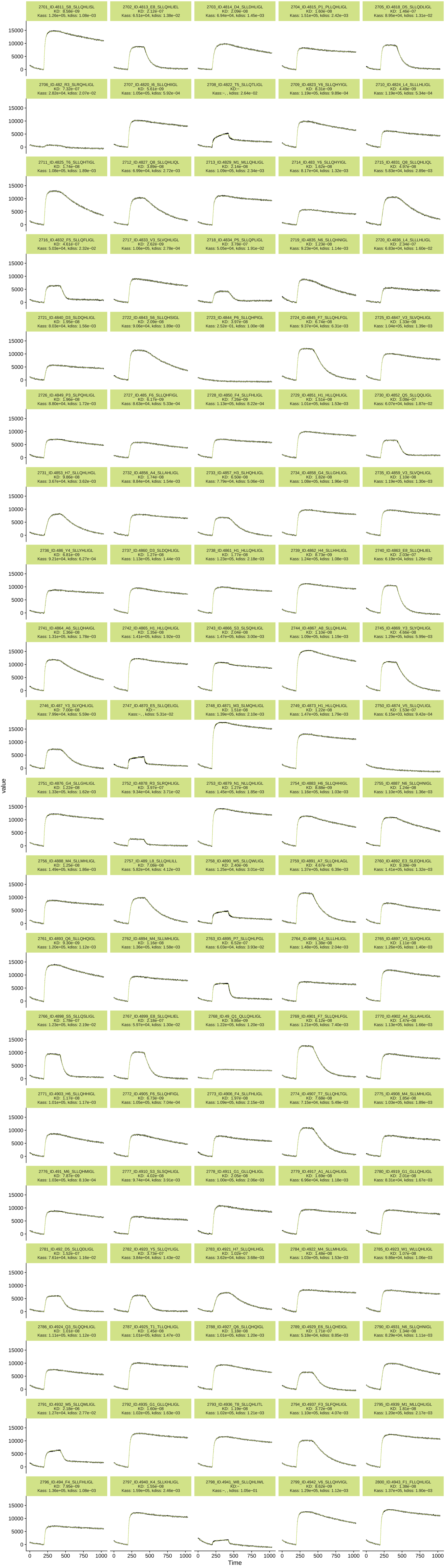
value
Time

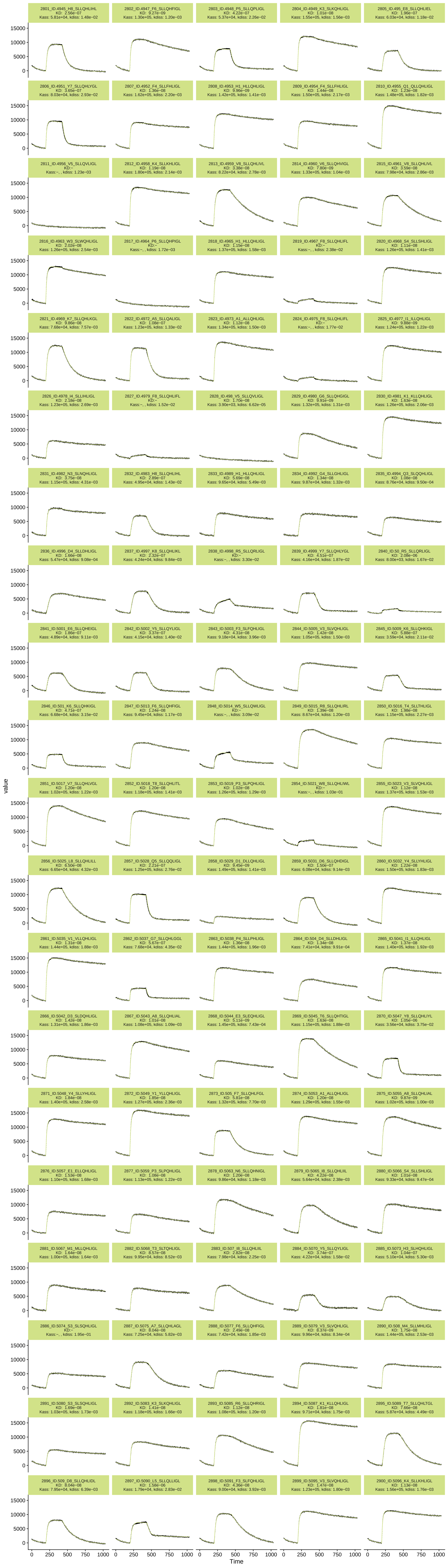
2801_ID.4945_H8_SLLQHLIHL KD: 2.56e−07 Kass: 5.81e+04, kdiss: 1.48e−02
2802_ID.4947_F6_SLLQHFIGL KD: 9.27e−09 Kass: 1.30e+05, kdiss: 1.20e−03
2803_ID.4948_P5_SLLQPLIGL KD: 4.22e−07 Kass: 5.37e+04, kdiss: 2.26e−02
2804_ID.4949_K3_SLKQHLIGL KD: 1.01e−08 Kass: 1.55e+05, kdiss: 1.56e−03
2805_ID.495_E8_SLLQHLIEL KD: 1.96e−07 Kass: 6.03e+04, kdiss: 1.18e−02
2806_ID.4951_Y7_SLLQHLYGL KD: 3.65e−07 Kass: 8.03e+04, kdiss: 2.93e−02
2807_ID.4952_F4_SLLFHLIGL KD: 1.36e−08 Kass: 1.62e+05, kdiss: 2.20e−03
2808_ID.4953_H1_HLLQHLIGL KD: 9.96e−09 Kass: 1.42e+05, kdiss: 1.41e−03
2809_ID.4954_F4_SLLFHLIGL KD: 1.44e−08 Kass: 1.50e+05, kdiss: 2.17e−03
2810_ID.4955_Q1_QLLQHLIGL KD: 1.23e−08 Kass: 1.48e+05, kdiss: 1.82e−03
2811_ID.4956_V5_SLLQVLIGL KD:− Kass:−, , kdiss: 1.23e−03
2812_ID.4958_K4_SLLKHLIGL KD: 1.19e−08 Kass: 1.80e+05, kdiss: 2.14e−03
2813_ID.4959_V8_SLLQHLIVL KD: 3.38e−08 Kass: 8.22e+04, kdiss: 2.78e−03
2814_ID.4960_V6_SLLQHVIGL KD: 7.80e−09 Kass: 1.33e+05, kdiss: 1.04e−03
2815_ID.4961_V8_SLLQHLIVL KD: 3.59e−08 Kass: 7.98e+04, kdiss: 2.86e−03
2816_ID.4963_W3_SLWQHLIGL KD: 2.02e−08 Kass: 1.26e+05, kdiss: 2.54e−03
2817_ID.4964_P6_SLLQHPIGL KD:− Kass:−, , kdiss: 1.72e−03
2818_ID.4965_H1_HLLQHLIGL KD: 1.15e−08 Kass: 1.37e+05, kdiss: 1.58e−03
2819_ID.4967_F8_SLLQHLIFL KD:− Kass:−, , kdiss: 2.38e−02
2820_ID.4968_S4_SLLSHLIGL KD: 1.11e−08 Kass: 1.26e+05, kdiss: 1.41e−03
2821_ID.4969_K7_SLLQHLKGL KD: 9.86e−08 Kass: 7.68e+04, kdiss: 7.57e−03
2822_ID.4972_A5_SLLQALIGL KD: 1.08e−07 Kass: 1.23e+05, kdiss: 1.33e−02
2823_ID.4973_A1_ALLQHLIGL KD: 1.12e−08 Kass: 1.34e+05, kdiss: 1.50e−03
2824_ID.4975_F8_SLLQHLIFL KD:− Kass:−, , kdiss: 1.77e−02
2825_ID.4977_I1_ILLQHLIGL KD: 9.88e−09 Kass: 1.24e+05, kdiss: 1.22e−03
2826_ID.4978_I4_SLLIHLIGL KD: 2.18e−08 Kass: 1.23e+05, kdiss: 2.69e−03
2827_ID.4979_F8_SLLQHLIFL KD:− Kass:−, , kdiss: 1.52e−02
2828_ID.498_V5_SLLQVLIGL KD: 1.70e−08 Kass: 3.90e+03, kdiss: 6.62e−05
2829_ID.4980_G6_SLLQHGIGL KD: 9.91e−09 Kass: 1.32e+05, kdiss: 1.31e−03
2830_ID.4981_K1_KLLQHLIGL KD: 1.63e−08 Kass: 1.26e+05, kdiss: 2.06e−03
2831_ID.4982_N3_SLNQHLIGL KD: 3.75e−08 Kass: 1.15e+05, kdiss: 4.31e−03
2832_ID.4983_H8_SLLQHLIHL KD: 2.89e−07 Kass: 4.95e+04, kdiss: 1.43e−02
2833_ID.4989_H1_HLLQHLIGL KD: 5.69e−08 Kass: 9.65e+04, kdiss: 5.49e−03
2834_ID.4992_G4_SLLGHLIGL KD: 1.34e−08 Kass: 9.87e+04, kdiss: 1.32e−03
2835_ID.4994_Q3_SLQQHLIGL KD: 1.08e−08 Kass: 8.76e+04, kdiss: 9.50e−04
2836_ID.4996_D4_SLLDHLIGL KD: 1.66e−08 Kass: 5.47e+04, kdiss: 9.08e−04
2837_ID.4997_K8_SLLQHLIKL KD: 2.32e−07 Kass: 4.24e+04, kdiss: 9.84e−03
2838_ID.4998_R5_SLLQRLIGL KD:− Kass:−, , kdiss: 3.30e−02
2839_ID.4999_Y7_SLLQHLYGL KD: 4.51e−07 Kass: 4.16e+04, kdiss: 1.87e−02
2840_ID.50_R5_SLLQRLIGL KD: 2.08e−06 Kass: 8.00e+03, kdiss: 1.67e−02
2841_ID.5001_E6_SLLQHEIGL KD: 1.86e−07 Kass: 4.89e+04, kdiss: 9.11e−03
2842_ID.5002_Y5_SLLQYLIGL KD: 3.37e−07 Kass: 4.15e+04, kdiss: 1.40e−02
2843_ID.5003_F3_SLFQHLIGL KD: 4.31e−08 Kass: 9.18e+04, kdiss: 3.96e−03
2844_ID.5005_V3_SLVQHLIGL KD: 1.42e−08 Kass: 1.05e+05, kdiss: 1.50e−03
2845_ID.5009_K6_SLLQHKIGL KD: 5.88e−07 Kass: 3.59e+04, kdiss: 2.11e−02
2846_ID.501_K6_SLLQHKIGL KD: 4.71e−07 Kass: 6.68e+04, kdiss: 3.15e−02
2847_ID.5013_F6_SLLQHFIGL KD: 1.24e−08 Kass: 9.45e+04, kdiss: 1.17e−03
2848_ID.5014_W5_SLLQWLIGL KD:− Kass:−, , kdiss: 3.09e−02
2849_ID.5015_R8_SLLQHLIRL KD: 1.39e−08 Kass: 8.67e+04, kdiss: 1.20e−03
2850_ID.5016_T4_SLLTHLIGL KD: 1.98e−08 Kass: 1.15e+05, kdiss: 2.27e−03
2851_ID.5017_V7_SLLQHLVGL KD: 1.20e−08 Kass: 1.02e+05, kdiss: 1.22e−03
2852_ID.5018_T8_SLLQHLITL KD: 1.20e−08 Kass: 1.18e+05, kdiss: 1.41e−03
2853_ID.5019_P3_SLPQHLIGL KD: 1.02e−08 Kass: 1.26e+05, kdiss: 1.29e−03
2854_ID.5021_W8_SLLQHLIWL KD:− Kass:−, , kdiss: 1.03e−01
2855_ID.5023_V3_SLVQHLIGL KD: 1.12e−08 Kass: 1.37e+05, kdiss: 1.53e−03
2856_ID.5025_L8_SLLQHLILL KD: 6.50e−08 Kass: 6.65e+04, kdiss: 4.32e−03
2857_ID.5028_Q5_SLLQQLIGL KD: 2.21e−07 Kass: 1.25e+05, kdiss: 2.76e−02
2858_ID.5029_D1_DLLQHLIGL KD: 9.45e−09 Kass: 1.49e+05, kdiss: 1.41e−03
2859_ID.5031_D6_SLLQHDIGL KD: 1.50e−07 Kass: 6.08e+04, kdiss: 9.14e−03
2860_ID.5032_Y4_SLLYHLIGL KD: 1.22e−08 Kass: 1.50e+05, kdiss: 1.83e−03
2861_ID.5035_V1_VLLQHLIGL KD: 1.31e−08 Kass: 1.44e+05, kdiss: 1.88e−03
2862_ID.5037_G7_SLLQHLGGL KD: 5.67e−07 Kass: 7.68e+04, kdiss: 4.35e−02
2863_ID.5038_P4_SLLPHLIGL KD: 1.36e−08 Kass: 1.44e+05, kdiss: 1.96e−03
2864_ID.504_D4_SLLDHLIGL KD: 1.34e−08 Kass: 7.41e+04, kdiss: 9.91e−04
2865_ID.5041_I1_ILLQHLIGL KD: 1.37e−08 Kass: 1.40e+05, kdiss: 1.92e−03
2866_ID.5042_D3_SLDQHLIGL KD: 1.42e−08 Kass: 1.31e+05, kdiss: 1.86e−03
2867_ID.5043_A8_SLLQHLIAL KD: 1.01e−08 Kass: 1.08e+05, kdiss: 1.09e−03
2868_ID.5044_E3_SLEQHLIGL KD: 5.11e−09 Kass: 1.45e+05, kdiss: 7.43e−04
2869_ID.5045_T6_SLLQHTIGL KD: 1.63e−08 Kass: 1.15e+05, kdiss: 1.88e−03
2870_ID.5047_Y8_SLLQHLIYL KD: 1.05e−06 Kass: 3.56e+04, kdiss: 3.75e−02
2871_ID.5048_Y4_SLLYHLIGL KD: 1.84e−08 Kass: 1.40e+05, kdiss: 2.58e−03
2872_ID.5049_Y1_YLLQHLIGL KD: 1.85e−08 Kass: 1.27e+05, kdiss: 2.36e−03
2873_ID.505_F7_SLLQHLFGL KD: 5.81e−08 Kass: 1.32e+05, kdiss: 7.70e−03
2874_ID.5053_A1_ALLQHLIGL KD: 1.20e−08 Kass: 1.29e+05, kdiss: 1.55e−03
2875_ID.5055_A8_SLLQHLIAL KD: 9.87e−09 Kass: 1.02e+05, kdiss: 1.00e−03
2876_ID.5057_E1_ELLQHLIGL KD: 1.53e−08 Kass: 1.10e+05, kdiss: 1.68e−03
2877_ID.5059_P3_SLPQHLIGL KD: 1.08e−08 Kass: 1.13e+05, kdiss: 1.22e−03
2878_ID.5063_N6_SLLQHNIGL KD: 1.20e−08 Kass: 9.86e+04, kdiss: 1.18e−03
2879_ID.5065_I8_SLLQHLIIL KD: 4.22e−08 Kass: 5.64e+04, kdiss: 2.38e−03
2880_ID.5066_S4_SLLSHLIGL KD: 1.01e−08 Kass: 9.33e+04, kdiss: 9.47e−04
2881_ID.5067_M1_MLLQHLIGL KD: 1.64e−08 Kass: 1.00e+05, kdiss: 1.64e−03
2882_ID.5068_T3_SLTQHLIGL KD: 8.57e−08 Kass: 9.95e+04, kdiss: 8.52e−03
2883_ID.507_I8_SLLQHLIIL KD: 2.82e−08 Kass: 7.98e+04, kdiss: 2.25e−03
2884_ID.5070_Y5_SLLQYLIGL KD: 3.74e−07 Kass: 4.22e+04, kdiss: 1.58e−02
2885_ID.5073_H3_SLHQHLIGL KD: 1.04e−07 Kass: 5.10e+04, kdiss: 5.30e−03
2886_ID.5074_S3_SLSQHLIGL KD:− Kass:−, , kdiss: 1.95e−01
2887_ID.5075_A7_SLLQHLAGL KD: 8.04e−08 Kass: 7.25e+04, kdiss: 5.82e−03
2888_ID.5077_F6_SLLQHFIGL KD: 2.49e−08 Kass: 7.42e+04, kdiss: 1.85e−03
2889_ID.5079_V3_SLVQHLIGL KD: 8.37e−09 Kass: 9.96e+04, kdiss: 8.34e−04
2890_ID.508_M4_SLLMHLIGL KD: 1.75e−08 Kass: 1.44e+05, kdiss: 2.53e−03
2891_ID.5080_S3_SLSQHLIGL KD: 1.69e−08 Kass: 1.03e+05, kdiss: 1.73e−03
2892_ID.5083_K3_SLKQHLIGL KD: 1.41e−08 Kass: 1.18e+05, kdiss: 1.66e−03
2893_ID.5085_R6_SLLQHRIGL KD: 1.12e−08 Kass: 1.08e+05, kdiss: 1.20e−03
2894_ID.5087_K1_KLLQHLIGL KD: 1.81e−08 Kass: 9.71e+04, kdiss: 1.75e−03
2895_ID.5089_T7_SLLQHLTGL KD: 7.66e−08 Kass: 5.87e+04, kdiss: 4.49e−03
2896_ID.509_D8_SLLQHLIDL KD: 8.04e−08 Kass: 7.95e+04, kdiss: 6.39e−03
2897_ID.5090_L5_SLLQLLIGL KD: 1.58e−06 Kass: 1.79e+04, kdiss: 2.83e−02
2898_ID.5091_F3_SLFQHLIGL KD: 4.36e−08 Kass: 9.00e+04, kdiss: 3.92e−03
2899_ID.5095_V3_SLVQHLIGL KD: 1.47e−08 Kass: 1.23e+05, kdiss: 1.80e−03
2900_ID.5096_K4_SLLKHLIGL KD: 1.13e−08 Kass: 1.56e+05, kdiss: 1.76e−03
value
15000
10000
5000
0
Time
0
250
500
750
1000

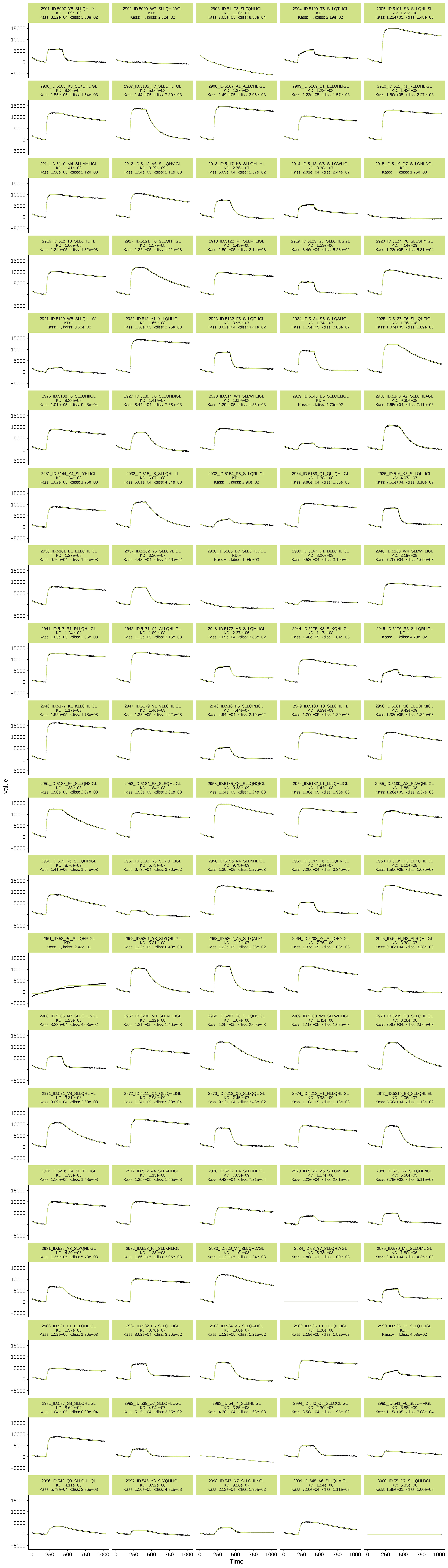
2901_ID.5097_Y8_SLLQHLIYL KD: 1.09e−06 Kass: 3.22e+04, kdiss: 3.50e−02
2902_ID.5099_W7_SLLQHLWGL KD:− Kass:−, , kdiss: 2.72e−02
2903_ID.51_F3_SLFQHLIGL KD: 1.16e−07 Kass: 7.63e+03, kdiss: 8.88e−04
2904_ID.5100_T5_SLLQTLIGL KD:− Kass:−, , kdiss: 2.19e−02
2905_ID.5101_S8_SLLQHLISL KD: 1.21e−08 Kass: 1.22e+05, kdiss: 1.48e−03
2906_ID.5103_K3_SLKQHLIGL KD: 9.89e−09 Kass: 1.55e+05, kdiss: 1.54e−03
2907_ID.5105_F7_SLLQHLFGL KD: 5.06e−08 Kass: 1.44e+05, kdiss: 7.30e−03
2908_ID.5107_A1_ALLQHLIGL KD: 1.37e−08 Kass: 1.49e+05, kdiss: 2.05e−03
2909_ID.5109_E1_ELLQHLIGL KD: 1.28e−08 Kass: 1.23e+05, kdiss: 1.57e−03
2910_ID.511_R1_RLLQHLIGL KD: 1.42e−08 Kass: 1.60e+05, kdiss: 2.27e−03
2911_ID.5110_M4_SLLMHLIGL KD: 1.41e−08 Kass: 1.50e+05, kdiss: 2.12e−03
2912_ID.5112_V6_SLLQHVIGL KD: 8.29e−09 Kass: 1.34e+05, kdiss: 1.11e−03
2913_ID.5117_H8_SLLQHLIHL KD: 2.76e−07 Kass: 5.69e+04, kdiss: 1.57e−02
2914_ID.5118_W5_SLLQWLIGL KD: 8.38e−07 Kass: 2.91e+04, kdiss: 2.44e−02
2915_ID.5119_D7_SLLQHLDGL KD:− Kass:−, , kdiss: 1.75e−03
2916_ID.512_T8_SLLQHLITL KD: 1.06e−08 Kass: 1.24e+05, kdiss: 1.32e−03
2917_ID.5121_T6_SLLQHTIGL KD: 1.57e−08 Kass: 1.22e+05, kdiss: 1.91e−03
2918_ID.5122_F4_SLLFHLIGL KD: 1.43e−08 Kass: 1.50e+05, kdiss: 2.14e−03
2919_ID.5123_G7_SLLQHLGGL KD: 1.53e−06 Kass: 3.46e+04, kdiss: 5.28e−02
2920_ID.5127_Y6_SLLQHYIGL KD: 4.14e−09 Kass: 1.28e+05, kdiss: 5.31e−04
2921_ID.5129_W8_SLLQHLIWL KD:− Kass:−, , kdiss: 8.52e−02
2922_ID.513_Y1_YLLQHLIGL KD: 1.65e−08 Kass: 1.36e+05, kdiss: 2.25e−03
2923_ID.5132_F5_SLLQFLIGL KD: 3.95e−07 Kass: 8.62e+04, kdiss: 3.41e−02
2924_ID.5134_S5_SLLQSLIGL KD: 1.74e−07 Kass: 1.15e+05, kdiss: 2.00e−02
2925_ID.5137_T6_SLLQHTIGL KD: 1.76e−08 Kass: 1.07e+05, kdiss: 1.89e−03
2926_ID.5138_I6_SLLQHIIGL KD: 9.38e−09 Kass: 1.01e+05, kdiss: 9.48e−04
2927_ID.5139_D6_SLLQHDIGL KD: 1.41e−07 Kass: 5.44e+04, kdiss: 7.65e−03
2928_ID.514_W4_SLLWHLIGL KD: 1.05e−08 Kass: 1.29e+05, kdiss: 1.36e−03
2929_ID.5140_E5_SLLQELIGL KD:− Kass:−, , kdiss: 4.70e−02
2930_ID.5143_A7_SLLQHLAGL KD: 9.30e−08 Kass: 7.65e+04, kdiss: 7.11e−03
2931_ID.5144_Y4_SLLYHLIGL KD: 1.24e−08 Kass: 1.02e+05, kdiss: 1.26e−03
2932_ID.515_L8_SLLQHLILL KD: 6.87e−08 Kass: 6.61e+04, kdiss: 4.54e−03
2933_ID.5154_R5_SLLQRLIGL KD:− Kass:−, , kdiss: 2.96e−02
2934_ID.5159_Q1_QLLQHLIGL KD: 1.38e−08 Kass: 9.88e+04, kdiss: 1.36e−03
2935_ID.516_K5_SLLQKLIGL KD: 4.07e−07 Kass: 7.62e+04, kdiss: 3.10e−02
2936_ID.5161_E1_ELLQHLIGL KD: 1.27e−08 Kass: 9.76e+04, kdiss: 1.24e−03
2937_ID.5162_Y5_SLLQYLIGL KD: 3.30e−07 Kass: 4.43e+04, kdiss: 1.46e−02
2938_ID.5165_D7_SLLQHLDGL KD:− Kass:−, , kdiss: 1.04e−03
2939_ID.5167_D1_DLLQHLIGL KD: 3.26e−09 Kass: 9.53e+04, kdiss: 3.10e−04
2940_ID.5168_W4_SLLWHLIGL KD: 2.19e−08 Kass: 7.70e+04, kdiss: 1.69e−03
2941_ID.517_R1_RLLQHLIGL KD: 1.24e−08 Kass: 1.65e+05, kdiss: 2.06e−03
2942_ID.5171_A1_ALLQHLIGL KD: 1.89e−08 Kass: 1.13e+05, kdiss: 2.15e−03
2943_ID.5172_M5_SLLQMLIGL KD: 2.27e−06 Kass: 1.69e+04, kdiss: 3.83e−02
2944_ID.5175_K3_SLKQHLIGL KD: 1.17e−08 Kass: 1.40e+05, kdiss: 1.64e−03
2945_ID.5176_R5_SLLQRLIGL KD:− Kass:−, , kdiss: 4.73e−02
2946_ID.5177_K1_KLLQHLIGL KD: 1.17e−08 Kass: 1.52e+05, kdiss: 1.78e−03
2947_ID.5179_V1_VLLQHLIGL KD: 1.46e−08 Kass: 1.32e+05, kdiss: 1.92e−03
2948_ID.518_P5_SLLQPLIGL KD: 4.44e−07 Kass: 4.94e+04, kdiss: 2.19e−02
2949_ID.5180_T8_SLLQHLITL KD: 9.53e−09 Kass: 1.26e+05, kdiss: 1.20e−03
2950_ID.5181_M6_SLLQHMIGL KD: 9.43e−09 Kass: 1.32e+05, kdiss: 1.24e−03
2951_ID.5183_S6_SLLQHSIGL KD: 1.38e−08 Kass: 1.50e+05, kdiss: 2.07e−03
2952_ID.5184_S3_SLSQHLIGL KD: 1.84e−08 Kass: 1.53e+05, kdiss: 2.81e−03
2953_ID.5185_Q6_SLLQHQIGL KD: 9.23e−09 Kass: 1.34e+05, kdiss: 1.24e−03
2954_ID.5187_L1_LLLQHLIGL KD: 1.42e−08 Kass: 1.38e+05, kdiss: 1.96e−03
2955_ID.5189_W3_SLWQHLIGL KD: 1.88e−08 Kass: 1.26e+05, kdiss: 2.37e−03
2956_ID.519_R6_SLLQHRIGL KD: 8.76e−09 Kass: 1.41e+05, kdiss: 1.24e−03
2957_ID.5192_R3_SLRQHLIGL KD: 5.73e−07 Kass: 6.73e+04, kdiss: 3.86e−02
2958_ID.5196_N4_SLLNHLIGL KD: 9.78e−09 Kass: 1.30e+05, kdiss: 1.27e−03
2959_ID.5197_K6_SLLQHKIGL KD: 4.64e−07 Kass: 7.20e+04, kdiss: 3.34e−02
2960_ID.5199_K3_SLKQHLIGL KD: 1.11e−08 Kass: 1.50e+05, kdiss: 1.67e−03
2961_ID.52_P6_SLLQHPIGL KD:− Kass:−, , kdiss: 2.42e−01
2962_ID.5201_Y3_SLYQHLIGL KD: 5.31e−08 Kass: 1.22e+05, kdiss: 6.48e−03
2963_ID.5202_A5_SLLQALIGL KD: 1.12e−07 Kass: 1.23e+05, kdiss: 1.38e−02
2964_ID.5203_Y6_SLLQHYIGL KD: 7.76e−09 Kass: 1.37e+05, kdiss: 1.06e−03
2965_ID.5204_R3_SLRQHLIGL KD: 3.30e−07 Kass: 9.96e+04, kdiss: 3.28e−02
2966_ID.5205_N7_SLLQHLNGL KD: 1.25e−06 Kass: 3.23e+04, kdiss: 4.03e−02
2967_ID.5206_M4_SLLMHLIGL KD: 1.12e−08 Kass: 1.31e+05, kdiss: 1.46e−03
2968_ID.5207_S6_SLLQHSIGL KD: 1.67e−08 Kass: 1.25e+05, kdiss: 2.09e−03
2969_ID.5208_W4_SLLWHLIGL KD: 1.42e−08 Kass: 1.15e+05, kdiss: 1.62e−03
2970_ID.5209_Q8_SLLQHLIQL KD: 3.28e−08 Kass: 7.80e+04, kdiss: 2.56e−03
2971_ID.521_V8_SLLQHLIVL KD: 3.31e−08 Kass: 8.09e+04, kdiss: 2.68e−03
2972_ID.5211_Q1_QLLQHLIGL KD: 7.98e−09 Kass: 1.24e+05, kdiss: 9.88e−04
2973_ID.5212_Q5_SLLQQLIGL KD: 2.45e−07 Kass: 9.92e+04, kdiss: 2.43e−02
2974_ID.5213_H1_HLLQHLIGL KD: 9.98e−09 Kass: 1.18e+05, kdiss: 1.18e−03
2975_ID.5215_E8_SLLQHLIEL KD: 2.06e−07 Kass: 5.50e+04, kdiss: 1.13e−02
2976_ID.5216_T4_SLLTHLIGL KD: 1.35e−08 Kass: 1.10e+05, kdiss: 1.48e−03
2977_ID.522_A4_SLLAHLIGL KD: 1.15e−08 Kass: 1.35e+05, kdiss: 1.55e−03
2978_ID.5222_H4_SLLHHLIGL KD: 7.65e−09 Kass: 9.42e+04, kdiss: 7.21e−04
2979_ID.5226_M5_SLLQMLIGL KD: 1.17e−06 Kass: 2.23e+04, kdiss: 2.61e−02
2980_ID.523_N7_SLLQHLNGL KD: 6.56e−05 Kass: 7.79e+02, kdiss: 5.11e−02
2981_ID.525_Y3_SLYQHLIGL KD: 4.29e−08 Kass: 1.35e+05, kdiss: 5.78e−03
2982_ID.528_K4_SLLKHLIGL KD: 1.23e−08 Kass: 1.66e+05, kdiss: 2.05e−03
2983_ID.529_V7_SLLQHLVGL KD: 1.10e−08 Kass: 1.12e+05, kdiss: 1.24e−03
2984_ID.53_Y7_SLLQHLYGL KD: 5.33e−08 Kass: 1.88e−01, kdiss: 1.00e−08
2985_ID.530_M5_SLLQMLIGL KD: 1.80e−06 Kass: 2.42e+04, kdiss: 4.35e−02
2986_ID.531_E1_ELLQHLIGL KD: 1.57e−08 Kass: 1.12e+05, kdiss: 1.76e−03
2987_ID.532_F5_SLLQFLIGL KD: 3.78e−07 Kass: 8.62e+04, kdiss: 3.26e−02
2988_ID.534_A5_SLLQALIGL KD: 1.08e−07 Kass: 1.12e+05, kdiss: 1.21e−02
2989_ID.535_F1_FLLQHLIGL KD: 1.28e−08 Kass: 1.18e+05, kdiss: 1.52e−03
2990_ID.536_T5_SLLQTLIGL KD:− Kass:−, , kdiss: 4.58e−02
2991_ID.537_S8_SLLQHLISL KD: 8.62e−09 Kass: 1.04e+05, kdiss: 8.99e−04
2992_ID.539_Q7_SLLQHLQGL KD: 4.94e−07 Kass: 5.15e+04, kdiss: 2.55e−02
2993_ID.54_I4_SLLIHLIGL KD: 3.85e−08 Kass: 4.38e+04, kdiss: 1.68e−03
2994_ID.540_Q5_SLLQQLIGL KD: 2.30e−07 Kass: 8.50e+04, kdiss: 1.95e−02
2995_ID.541_F6_SLLQHFIGL KD: 6.88e−09 Kass: 1.15e+05, kdiss: 7.88e−04
2996_ID.543_Q8_SLLQHLIQL KD: 4.11e−08 Kass: 5.73e+04, kdiss: 2.36e−03
2997_ID.545_Y3_SLYQHLIGL KD: 3.92e−08 Kass: 1.10e+05, kdiss: 4.31e−03
2998_ID.547_N7_SLLQHLNGL KD: 9.16e−07 Kass: 2.13e+04, kdiss: 1.96e−02
2999_ID.548_A6_SLLQHAIGL KD: 1.54e−08 Kass: 7.16e+04, kdiss: 1.11e−03
3000_ID.55_D7_SLLQHLDGL KD: 5.33e−08 Kass: 1.88e−01, kdiss: 1.00e−08
value
15000
10000
5000
0
−5000
0 250 500 750 1000
Time

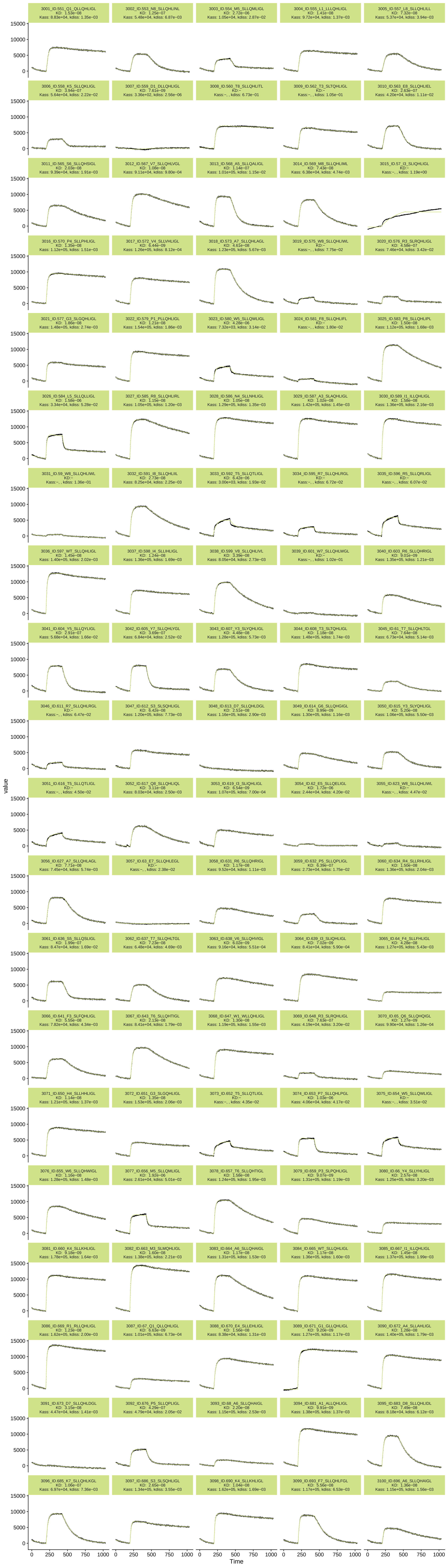

value
Time

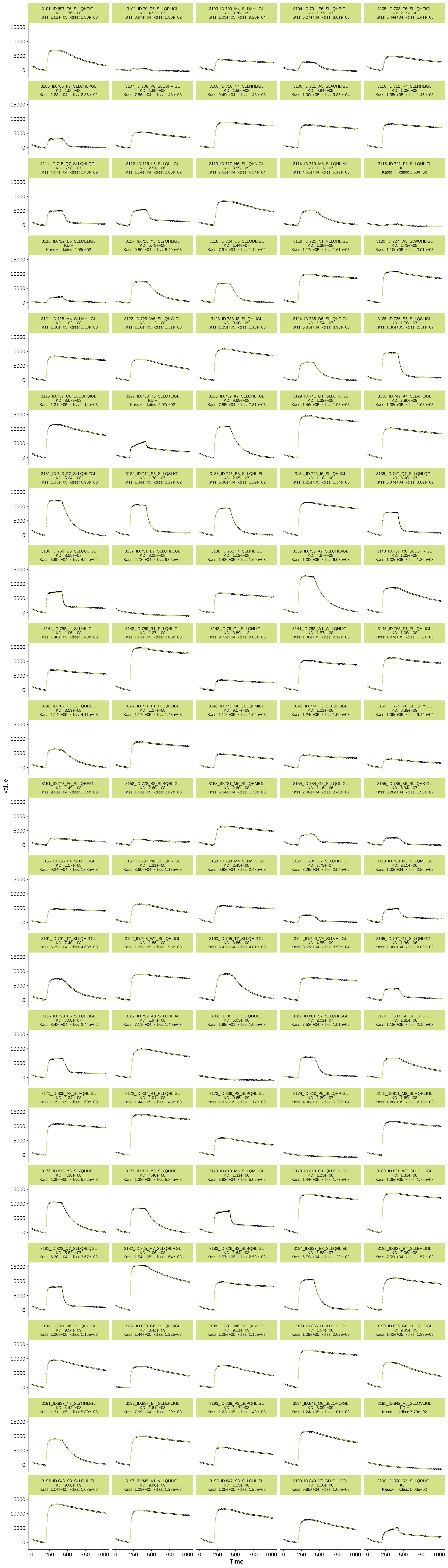
3101_ID.697_T6_SLLQHTIGL KD: 1.76e−08 Kass: 1.02e+05, kdiss: 1.80e−03
3102_ID.70_P5_SLLQPLIGL KD: 4.53e−07 Kass: 3.97e+04, kdiss: 1.80e−02
3103_ID.700_M4_SLLMHLIGL KD: 8.76e−09 Kass: 1.05e+05, kdiss: 9.20e−04
3104_ID.701_E6_SLLQHEIGL KD: 1.37e−07 Kass: 6.27e+04, kdiss: 8.61e−03
3105_ID.703_F6_SLLQHFIGL KD: 2.19e−08 Kass: 6.44e+04, kdiss: 1.41e−03
3106_ID.705_P7_SLLQHLPGL KD: 1.06e−06 Kass: 2.23e+04, kdiss: 2.36e−02
3107_ID.708_V6_SLLQHVIGL KD: 1.89e−08 Kass: 7.56e+04, kdiss: 1.43e−03
3108_ID.710_N4_SLLNHLIGL KD: 1.50e−08 Kass: 9.49e+04, kdiss: 1.43e−03
3109_ID.711_A3_SLAQHLIGL KD: 9.84e−09 Kass: 1.00e+05, kdiss: 9.86e−04
3110_ID.712_R4_SLLRHLIGL KD: 1.08e−08 Kass: 1.35e+05, kdiss: 1.45e−03
3111_ID.715_Q7_SLLQHLQGL KD: 5.98e−07 Kass: 4.07e+04, kdiss: 2.43e−02
3112_ID.716_L5_SLLQLLIGL KD: 2.51e−06 Kass: 1.14e+04, kdiss: 2.85e−02
3113_ID.717_N6_SLLQHNIGL KD: 8.59e−09 Kass: 7.61e+04, kdiss: 6.54e−04
3114_ID.719_M8_SLLQHLIML KD: 1.11e−07 Kass: 4.62e+04, kdiss: 5.12e−03
3115_ID.721_F8_SLLQHLIFL KD:− Kass:−, , kdiss: 2.63e−02
3116_ID.722_E5_SLLQELIGL KD:− Kass:−, , kdiss: 4.08e−02
3117_ID.723_Y3_SLYQHLIGL KD: 5.78e−08 Kass: 9.50e+04, kdiss: 5.49e−03
3118_ID.724_D5_SLLQDLIGL KD: 1.44e−07 Kass: 7.91e+04, kdiss: 1.14e−02
3119_ID.725_N1_NLLQHLIGL KD: 1.38e−08 Kass: 1.17e+05, kdiss: 1.61e−03
3120_ID.727_W3_SLWQHLIGL KD: 2.73e−08 Kass: 1.10e+05, kdiss: 3.01e−03
3121_ID.728_M4_SLLMHLIGL KD: 1.02e−08 Kass: 1.30e+05, kdiss: 1.33e−03
3122_ID.729_W6_SLLQHWIGL KD: 1.12e−08 Kass: 1.16e+05, kdiss: 1.31e−03
3123_ID.733_I3_SLIQHLIGL KD: 9.00e−09 Kass: 1.25e+05, kdiss: 1.13e−03
3124_ID.735_D6_SLLQHDIGL KD: 1.54e−07 Kass: 5.83e+04, kdiss: 8.98e−03
3125_ID.736_S5_SLLQSLIGL KD: 1.78e−07 Kass: 1.30e+05, kdiss: 2.31e−02
3126_ID.737_Q6_SLLQHQIGL KD: 8.67e−09 Kass: 1.31e+05, kdiss: 1.14e−03
3127_ID.738_T5_SLLQTLIGL KD:− Kass:−, , kdiss: 2.07e−02
3128_ID.739_K7_SLLQHLKGL KD: 9.68e−08 Kass: 7.55e+04, kdiss: 7.31e−03
3129_ID.741_G1_GLLQHLIGL KD: 1.32e−08 Kass: 1.46e+05, kdiss: 1.93e−03
3130_ID.742_A4_SLLAHLIGL KD: 7.86e−09 Kass: 1.38e+05, kdiss: 1.08e−03
3131_ID.743_F7_SLLQHLFGL KD: 5.24e−08 Kass: 1.33e+05, kdiss: 6.95e−03
3132_ID.744_S5_SLLQSLIGL KD: 1.70e−07 Kass: 1.34e+05, kdiss: 2.27e−02
3133_ID.745_E8_SLLQHLIEL KD: 2.05e−07 Kass: 6.30e+04, kdiss: 1.29e−02
3134_ID.746_I6_SLLQHIIGL KD: 1.10e−08 Kass: 1.22e+05, kdiss: 1.34e−03
3135_ID.747_Q7_SLLQHLQGL KD: 5.68e−07 Kass: 6.37e+04, kdiss: 3.62e−02
3136_ID.750_G5_SLLQGLIGL KD: 8.25e−07 Kass: 5.99e+04, kdiss: 4.94e−02
3137_ID.751_E7_SLLQHLEGL KD: 3.25e−08 Kass: 2.78e+04, kdiss: 9.05e−04
3138_ID.752_I4_SLLIHLIGL KD: 1.12e−08 Kass: 1.42e+05, kdiss: 1.60e−03
3139_ID.753_A7_SLLQHLAGL KD: 4.67e−08 Kass: 1.30e+05, kdiss: 6.08e−03
3140_ID.757_R6_SLLQHRIGL KD: 1.02e−08 Kass: 1.33e+05, kdiss: 1.36e−03
3141_ID.758_I4_SLLIHLIGL KD: 1.06e−08 Kass: 1.40e+05, kdiss: 1.48e−03
3142_ID.759_R1_RLLQHLIGL KD: 1.27e−08 Kass: 1.61e+05, kdiss: 2.05e−03
3143_ID.76_G4_SLLGHLIGL KD: 8.89e−13 Kass: 9.71e+04, kdiss: 8.63e−08
3144_ID.763_W1_WLLQHLIGL KD: 1.57e−08 Kass: 1.38e+05, kdiss: 2.17e−03
3145_ID.765_F1_FLLQHLIGL KD: 1.08e−08 Kass: 1.27e+05, kdiss: 1.38e−03
3146_ID.767_F3_SLFQHLIGL KD: 3.59e−08 Kass: 1.14e+05, kdiss: 4.11e−03
3147_ID.771_F1_FLLQHLIGL KD: 1.27e−08 Kass: 1.17e+05, kdiss: 1.48e−03
3148_ID.773_M6_SLLQHMIGL KD: 9.17e−09 Kass: 1.11e+05, kdiss: 1.02e−03
3149_ID.774_T3_SLTQHLIGL KD: 1.21e−08 Kass: 1.24e+05, kdiss: 1.50e−03
3150_ID.775_Y6_SLLQHYIGL KD: 8.39e−09 Kass: 1.09e+05, kdiss: 9.14e−04
3151_ID.777_F6_SLLQHFIGL KD: 1.49e−08 Kass: 9.01e+04, kdiss: 1.34e−03
3152_ID.778_S3_SLSQHLIGL KD: 2.60e−08 Kass: 1.01e+05, kdiss: 2.62e−03
3153_ID.781_M6_SLLQHMIGL KD: 2.60e−08 Kass: 6.54e+04, kdiss: 1.70e−03
3154_ID.784_G5_SLLQGLIGL KD: 1.16e−06 Kass: 2.06e+04, kdiss: 2.40e−02
3155_ID.785_K6_SLLQHKIGL KD: 5.94e−07 Kass: 3.28e+04, kdiss: 1.95e−02
3156_ID.786_F4_SLLFHLIGL KD: 1.17e−08 Kass: 9.24e+04, kdiss: 1.08e−03
3157_ID.787_N6_SLLQHNIGL KD: 1.31e−08 Kass: 8.60e+04, kdiss: 1.13e−03
3158_ID.788_M4_SLLMHLIGL KD: 2.45e−08 Kass: 9.93e+04, kdiss: 2.43e−03
3159_ID.789_G7_SLLQHLGGL KD: 7.73e−07 Kass: 3.29e+04, kdiss: 2.54e−02
3160_ID.790_M5_SLLQMLIGL KD: 2.15e−06 Kass: 1.33e+04, kdiss: 2.86e−02
3161_ID.791_T7_SLLQHLTGL KD: 7.40e−08 Kass: 6.25e+04, kdiss: 4.63e−03
3162_ID.793_WT_SLLQHLIGL KD: 1.86e−08 Kass: 1.05e+05, kdiss: 1.95e−03
3163_ID.795_T7_SLLQHLTGL KD: 8.86e−08 Kass: 5.42e+04, kdiss: 4.81e−03
3164_ID.796_V4_SLLVHLIGL KD: 4.55e−09 Kass: 8.57e+04, kdiss: 3.90e−04
3165_ID.797_G7_SLLQHLGGL KD: 1.36e−06 Kass: 2.08e+04, kdiss: 2.82e−02
3166_ID.798_F5_SLLQFLIGL KD: 7.00e−07 Kass: 3.48e+04, kdiss: 2.44e−02
3167_ID.799_A8_SLLQHLIAL KD: 1.97e−08 Kass: 7.11e+04, kdiss: 1.40e−03
3168_ID.80_D5_SLLQDLIGL KD: 5.03e−08 Kass: 1.99e−01, kdiss: 1.00e−08
3169_ID.801_S7_SLLQHLSGL KD: 2.01e−07 Kass: 7.52e+04, kdiss: 1.51e−02
3170_ID.803_S6_SLLQHSIGL KD: 1.82e−08 Kass: 1.18e+05, kdiss: 2.15e−03
3171_ID.805_A3_SLAQHLIGL KD: 1.24e−08 Kass: 1.29e+05, kdiss: 1.60e−03
3172_ID.807_R1_RLLQHLIGL KD: 1.01e−08 Kass: 1.44e+05, kdiss: 1.45e−03
3173_ID.809_P3_SLPQHLIGL KD: 9.65e−09 Kass: 1.21e+05, kdiss: 1.17e−03
3174_ID.810_P6_SLLQHPIGL KD: 1.29e−07 Kass: 4.08e+03, kdiss: 5.28e−04
3175_ID.811_M3_SLMQHLIGL KD: 1.69e−08 Kass: 1.28e+05, kdiss: 2.15e−03
3176_ID.815_Y3_SLYQHLIGL KD: 4.38e−08 Kass: 1.35e+05, kdiss: 5.92e−03
3177_ID.817_Y3_SLYQHLIGL KD: 4.40e−08 Kass: 1.33e+05, kdiss: 5.84e−03
3178_ID.818_M5_SLLQMLIGL KD: 1.31e−06 Kass: 3.83e+04, kdiss: 5.02e−02
3179_ID.819_Q1_QLLQHLIGL KD: 1.23e−08 Kass: 1.44e+05, kdiss: 1.77e−03
3180_ID.821_WT_SLLQHLIGL KD: 1.33e−08 Kass: 1.35e+05, kdiss: 1.79e−03
3181_ID.823_Q7_SLLQHLQGL KD: 5.82e−07 Kass: 6.30e+04, kdiss: 3.67e−02
3182_ID.825_M7_SLLQHLMGL KD: 1.06e−08 Kass: 1.54e+05, kdiss: 1.64e−03
3183_ID.826_S3_SLSQHLIGL KD: 1.64e−08 Kass: 1.57e+05, kdiss: 2.58e−03
3184_ID.827_E8_SLLQHLIEL KD: 1.88e−07 Kass: 6.79e+04, kdiss: 1.28e−02
3185_ID.828_E4_SLLEHLIGL KD: 2.06e−08 Kass: 7.59e+04, kdiss: 1.57e−03
3186_ID.829_H6_SLLQHHIGL KD: 9.54e−09 Kass: 1.20e+05, kdiss: 1.15e−03
3187_ID.830_G6_SLLQHGIGL KD: 8.42e−09 Kass: 1.44e+05, kdiss: 1.22e−03
3188_ID.831_W6_SLLQHWIGL KD: 9.11e−09 Kass: 1.28e+05, kdiss: 1.16e−03
3189_ID.835_I1_ILLQHLIGL KD: 1.57e−08 Kass: 1.29e+05, kdiss: 2.02e−03
3190_ID.836_G6_SLLQHGIGL KD: 9.35e−09 Kass: 1.42e+05, kdiss: 1.33e−03
3191_ID.837_Y3_SLYQHLIGL KD: 4.44e−08 Kass: 1.31e+05, kdiss: 5.80e−03
3192_ID.838_E4_SLLEHLIGL KD: 1.61e−08 Kass: 7.99e+04, kdiss: 1.29e−03
3193_ID.839_P3_SLPQHLIGL KD: 1.17e−08 Kass: 1.22e+05, kdiss: 1.43e−03
3194_ID.841_Q6_SLLQHQIGL KD: 8.09e−09 Kass: 1.24e+05, kdiss: 1.01e−03
3195_ID.842_V5_SLLQVLIGL KD:− Kass:−, , kdiss: 7.70e−02
3196_ID.843_S8_SLLQHLISL KD: 9.08e−09 Kass: 1.14e+05, kdiss: 1.03e−03
3197_ID.845_V1_VLLQHLIGL KD: 9.96e−09 Kass: 1.23e+05, kdiss: 1.23e−03
3198_ID.847_S8_SLLQHLISL KD: 1.15e−08 Kass: 1.08e+05, kdiss: 1.25e−03
3199_ID.849_V7_SLLQHLVGL KD: 1.10e−08 Kass: 9.85e+04, kdiss: 1.08e−03
3200_ID.850_R5_SLLQRLIGL KD:− Kass:−, , kdiss: 5.03e−02
value
0
5000
10000
15000
Time
0
250
500
750
1000

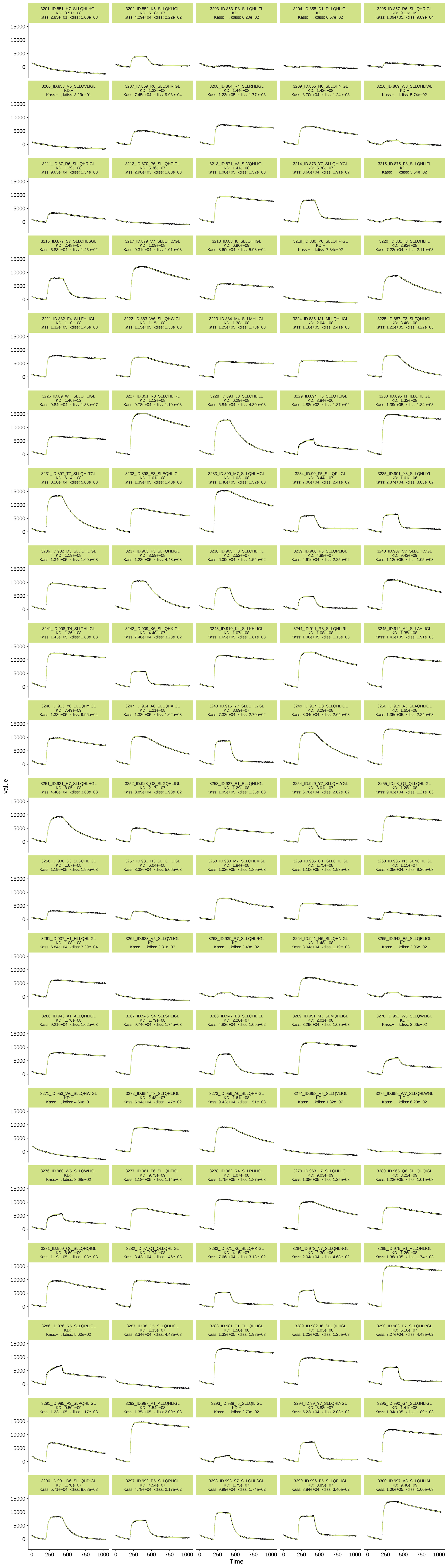
value
Time

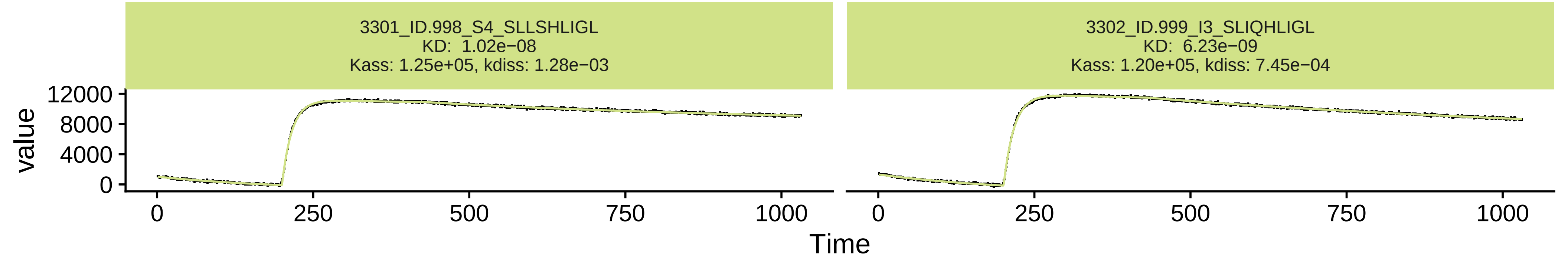
3301_ID.998_S4_SLLSHLIGL
KD: 1.02e−08
Kass: 1.25e+05, kdiss: 1.28e−03
3302_ID.999_I3_SLIQHLIGL
KD: 6.23e−09
Kass: 1.20e+05, kdiss: 7.45e−04
value
12000
8000
4000
0
0
250
500
750
1000
Time
