# Supplementary material for: An ultra-high-throughput screen for the evaluation of peptide HLA-Binder interactions
Source: Sci Rep. 2023 Mar 31;13:5290. doi: 10.1038/s41598-023-32384-z (PMC10066226; doi:10.1038/s41598-023-32384-z)
Supplement: Supplementary file 2 — Supplementary Information 2. [file 41598_2023_32384_MOESM2_ESM.zip › raw_data_package/K02025_240nM/K02025_240nM_binding_curve_plots.pdf]

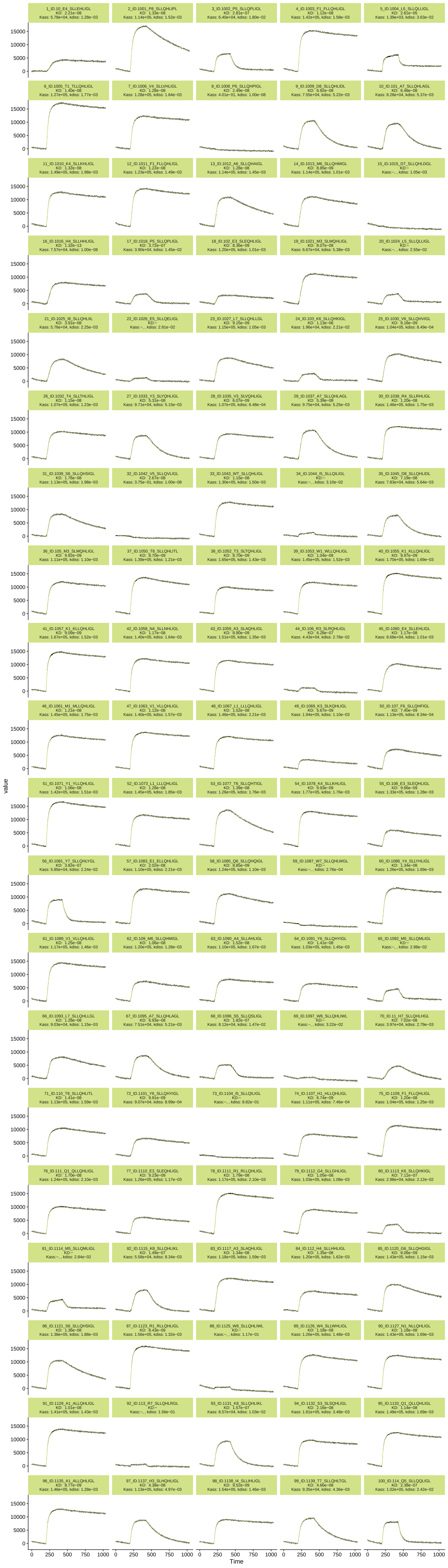

1_ID.10_E4_SLLEHLIGL
KD: 2.21e-08
Kass: 5.78e+04, kdiss: 1.28e-03
2_ID.1001_P8_SLLQHLIPL
KD: 1.33e-08
Kass: 1.14e+05, kdiss: 1.52e-03
3_ID.1002_P5_SLLQPLIGL
KD: 2.81e-07
Kass: 6.40e+04, kdiss: 1.80e-02
4_ID.1003_F1_FLLQHLIGL
KD: 1.12e-08
Kass: 1.42e+05, kdiss: 1.58e-03
5_ID.1004_L5_SLLQLLIGL
KD: 2.61e-05
Kass: 1.39e+03, kdiss: 3.63e-02
6_ID.1005_T1_TLLQHLIGL
KD: 1.40e-08
Kass: 1.27e+05, kdiss: 1.77e-03
7_ID.1006_V4_SLLVHLIGL
KD: 1.28e-08
Kass: 1.28e+05, kdiss: 1.64e-03
8_ID.1008_P6_SLLQHPIGL
KD: 2.49e-08
Kass: 4.01e-01, kdiss: 1.00e-08
9_ID.1009_D8_SLLQHLIDL
KD: 6.92e-08
Kass: 7.55e+04, kdiss: 5.22e-03
10_ID.101_A7_SLLQHLAGL
KD: 6.48e-08
Kass: 8.28e+04, kdiss: 5.37e-03
11_ID.1010_K4_SLLKHLIGL
KD: 1.33e-08
Kass: 1.49e+05, kdiss: 1.98e-03
12_ID.1011_F1_FLLQHLIGL
KD: 1.22e-08
Kass: 1.23e+05, kdiss: 1.49e-03
13_ID.1012_A6_SLLQHAIGL
KD: 1.28e-08
Kass: 1.14e+05, kdiss: 1.45e-03
14_ID.1013_M6_SLLQHMIGL
KD: 8.85e-09
Kass: 1.14e+05, kdiss: 1.01e-03
15_ID.1015_D7_SLLQHLDGL
KD:~
Kass:~, , kdiss: 1.05e-03
16_ID.1016_H4_SLLHHLIGL
KD: 1.15e-13
Kass: 7.57e+04, kdiss: 1.00e-08
17_ID.1018_P5_SLLQPLIGL
KD: 3.72e-07
Kass: 3.90e+04, kdiss: 1.45e-02
18_ID.102_E3_SLEQHLIGL
KD: 8.36e-09
Kass: 1.20e+05, kdiss: 1.01e-03
19_ID.1021_M3_SLMQHLIGL
KD: 8.07e-08
Kass: 6.67e+04, kdiss: 5.38e-03
20_ID.1024_L5_SLLQLLIGL
KD:~
Kass:~, , kdiss: 2.55e-02
21_ID.1025_I8_SLLQHLIIL
KD: 3.91e-08
Kass: 5.76e+04, kdiss: 2.25e-03
22_ID.1026_E5_SLLQELIGL
KD:~
Kass:~, , kdiss: 2.91e-02
23_ID.1027_L7_SLLQHLLGL
KD: 9.15e-09
Kass: 1.15e+05, kdiss: 1.05e-03
24_ID.103_K6_SLLQHKIGL
KD: 1.13e-06
Kass: 1.96e+04, kdiss: 2.21e-02
25_ID.1030_V6_SLLQHVIGL
KD: 8.16e-09
Kass: 1.04e+05, kdiss: 8.49e-04
26_ID.1032_T4_SLLTHLIGL
KD: 1.15e-08
Kass: 1.07e+05, kdiss: 1.23e-03
27_ID.1033_Y3_SLYQHLIGL
KD: 5.31e-08
Kass: 9.71e+04, kdiss: 5.15e-03
28_ID.1035_V3_SLVQHLIGL
KD: 6.07e-09
Kass: 1.07e+05, kdiss: 6.48e-04
29_ID.1037_A7_SLLQHLAGL
KD: 5.39e-08
Kass: 9.75e+04, kdiss: 5.25e-03
30_ID.1038_R4_SLLRHLIGL
KD: 1.20e-08
Kass: 1.46e+05, kdiss: 1.75e-03
31_ID.1039_S6_SLLQHSIGL
KD: 1.76e-08
Kass: 1.13e+05, kdiss: 1.98e-03
32_ID.1042_V5_SLLQVLIGL
KD: 2.67e-08
Kass: 3.75e-01, kdiss: 1.00e-08
33_ID.1043_WT_SLLQHLIGL
KD: 1.15e-08
Kass: 1.30e+05, kdiss: 1.50e-03
34_ID.1044_I5_SLLQILIGL
KD:~
Kass:~, , kdiss: 3.10e-02
35_ID.1045_D8_SLLQHLIDL
KD: 7.19e-08
Kass: 7.83e+04, kdiss: 5.64e-03
36_ID.105_M3_SLMQHLIGL
KD: 9.92e-09
Kass: 1.11e+05, kdiss: 1.10e-03
37_ID.1050_T8_SLLQHLITL
KD: 8.70e-09
Kass: 1.39e+05, kdiss: 1.21e-03
38_ID.1052_T3_SLTQHLIGL
KD: 8.70e-09
Kass: 1.65e+05, kdiss: 1.43e-03
39_ID.1053_W1_WLLQHLIGL
KD: 1.04e-08
Kass: 1.45e+05, kdiss: 1.52e-03
40_ID.1055_K1_KLLQHLIGL
KD: 9.97e-09
Kass: 1.70e+05, kdiss: 1.69e-03
41_ID.1057_K1_KLLQHLIGL
KD: 9.09e-09
Kass: 1.67e+05, kdiss: 1.52e-03
42_ID.1058_N4_SLLNHLIGL
KD: 1.17e-08
Kass: 1.40e+05, kdiss: 1.64e-03
43_ID.1059_A3_SLAQHLIGL
KD: 8.90e-09
Kass: 1.51e+05, kdiss: 1.35e-03
44_ID.106_R3_SLRQHLIGL
KD: 6.28e-07
Kass: 4.43e+04, kdiss: 2.78e-02
45_ID.1060_E4_SLLEHLIGL
KD: 1.17e-08
Kass: 8.68e+04, kdiss: 1.01e-03
46_ID.1061_M1_MLLQHLIGL
KD: 1.21e-08
Kass: 1.45e+05, kdiss: 1.75e-03
47_ID.1063_V1_VLLQHLIGL
KD: 1.12e-08
Kass: 1.40e+05, kdiss: 1.57e-03
48_ID.1067_L1_LLLQHLIGL
KD: 1.52e-08
Kass: 1.46e+05, kdiss: 2.21e-03
49_ID.1069_K3_SLKQHLIGL
KD: 5.67e-09
Kass: 1.94e+05, kdiss: 1.10e-03
50_ID.107_F6_SLLQHFIGL
KD: 7.40e-09
Kass: 1.13e+05, kdiss: 8.34e-04
51_ID.1071_Y1_YLLQHLIGL
KD: 1.06e-08
Kass: 1.42e+05, kdiss: 1.51e-03
52_ID.1073_L1_LLLQHLIGL
KD: 1.28e-08
Kass: 1.45e+05, kdiss: 1.85e-03
53_ID.1077_T6_SLLQHTIGL
KD: 1.39e-08
Kass: 1.26e+05, kdiss: 1.76e-03
54_ID.1078_K4_SLLKHLIGL
KD: 9.93e-09
Kass: 1.77e+05, kdiss: 1.76e-03
55_ID.108_E3_SLEQHLIGL
KD: 9.66e-09
Kass: 1.33e+05, kdiss: 1.28e-03
56_ID.1081_Y7_SLLQHLYGL
KD: 3.82e-07
Kass: 5.85e+04, kdiss: 2.24e-02
57_ID.1083_E1_ELLQHLIGL
KD: 2.02e-08
Kass: 1.10e+05, kdiss: 2.21e-03
58_ID.1085_Q6_SLLQHQIGL
KD: 8.85e-09
Kass: 1.24e+05, kdiss: 1.10e-03
59_ID.1087_W7_SLLQHLWGL
KD:~
Kass:~, , kdiss: 2.76e-04
60_ID.1088_Y4_SLLYHLIGL
KD: 1.34e-08
Kass: 1.26e+05, kdiss: 1.69e-03
61_ID.1089_V1_VLLQHLIGL
KD: 1.25e-08
Kass: 1.17e+05, kdiss: 1.46e-03
62_ID.109_M6_SLLQHMIGL
KD: 1.06e-08
Kass: 1.20e+05, kdiss: 1.28e-03
63_ID.1090_A4_SLLAHLIGL
KD: 1.52e-08
Kass: 1.10e+05, kdiss: 1.67e-03
64_ID.1091_Y6_SLLQHYIGL
KD: 1.41e-08
Kass: 1.03e+05, kdiss: 1.45e-03
65_ID.1092_M5_SLLQMLIGL
KD:~
Kass:~, , kdiss: 2.98e-02
66_ID.1093_L7_SLLQHLLGL
KD: 1.28e-08
Kass: 9.03e+04, kdiss: 1.15e-03
67_ID.1095_A7_SLLQHLAGL
KD: 6.93e-08
Kass: 7.51e+04, kdiss: 5.21e-03
68_ID.1096_S5_SLLQSLIGL
KD: 1.82e-07
Kass: 8.12e+04, kdiss: 1.47e-02
69_ID.1097_W8_SLLQHLIWL
KD:~
Kass:~, , kdiss: 3.22e-02
70_ID.11_H7_SLLQHLHGL
KD: 7.02e-08
Kass: 3.97e+04, kdiss: 2.79e-03
71_ID.110_T8_SLLQHLITL
KD: 1.41e-08
Kass: 1.13e+05, kdiss: 1.59e-03
72_ID.1101_Y6_SLLQHYIGL
KD: 9.91e-09
Kass: 9.07e+04, kdiss: 8.99e-04
73_ID.1104_I5_SLLQILIGL
KD:~
Kass:~, , kdiss: 8.82e-01
74_ID.1107_H1_HLLQHLIGL
KD: 6.74e-09
Kass: 1.11e+05, kdiss: 7.46e-04
75_ID.1109_F1_FLLQHLIGL
KD: 1.20e-08
Kass: 1.04e+05, kdiss: 1.25e-03
76_ID.111_Q1_QLLQHLIGL
KD: 1.70e-08
Kass: 1.24e+05, kdiss: 2.10e-03
77_ID.1110_E3_SLEQHLIGL
KD: 9.23e-09
Kass: 1.26e+05, kdiss: 1.17e-03
78_ID.1111_R1_RLLQHLIGL
KD: 1.79e-08
Kass: 1.17e+05, kdiss: 2.10e-03
79_ID.1112_G4_SLLGHLIGL
KD: 1.05e-08
Kass: 1.03e+05, kdiss: 1.08e-03
80_ID.1113_K6_SLLQHKIGL
KD: 7.11e-07
Kass: 2.98e+04, kdiss: 2.12e-02
81_ID.1114_M5_SLLQMLIGL
KD:~
Kass:~, , kdiss: 2.84e-02
82_ID.1115_K8_SLLQHLIKL
KD: 1.49e-07
Kass: 5.58e+04, kdiss: 8.34e-03
83_ID.1117_A3_SLAQHLIGL
KD: 1.34e-08
Kass: 1.18e+05, kdiss: 1.59e-03
84_ID.112_H4_SLLHHLIGL
KD: 1.35e-08
Kass: 1.20e+05, kdiss: 1.62e-03
85_ID.1120_G6_SLLQHGIGL
KD: 8.06e-09
Kass: 1.43e+05, kdiss: 1.15e-03
86_ID.1121_S6_SLLQHSIGL
KD: 1.36e-08
Kass: 1.38e+05, kdiss: 1.88e-03
87_ID.1123_R1_RLLQHLIGL
KD: 8.43e-09
Kass: 1.56e+05, kdiss: 1.32e-03
88_ID.1125_W8_SLLQHLIWL
KD:~
Kass:~, , kdiss: 1.17e-01
89_ID.1126_W4_SLLWHLIGL
KD: 1.18e-08
Kass: 1.26e+05, kdiss: 1.48e-03
90_ID.1127_N1_NLLQHLIGL
KD: 1.18e-08
Kass: 1.43e+05, kdiss: 1.69e-03
91_ID.1129_A1_ALLQHLIGL
KD: 1.01e-08
Kass: 1.41e+05, kdiss: 1.43e-03
92_ID.113_R7_SLLQHLRGL
KD:~
Kass:~, , kdiss: 1.56e-01
93_ID.1131_K8_SLLQHLIKL
KD: 1.57e-07
Kass: 6.57e+04, kdiss: 1.03e-02
94_ID.1132_S3_SLSQHLIGL
KD: 2.16e-08
Kass: 1.61e+05, kdiss: 3.48e-03
95_ID.1133_Q1_QLLQHLIGL
KD: 1.14e-08
Kass: 1.48e+05, kdiss: 1.69e-03
96_ID.1135_A1_ALLQHLIGL
KD: 8.77e-09
Kass: 1.46e+05, kdiss: 1.28e-03
97_ID.1137_H3_SLHQHLIGL
KD: 4.38e-08
Kass: 1.13e+05, kdiss: 4.97e-03
98_ID.1138_I4_SLLIHLIGL
KD: 9.52e-09
Kass: 1.54e+05, kdiss: 1.46e-03
99_ID.1139_T7_SLLQHLTGL
KD: 4.66e-08
Kass: 9.35e+04, kdiss: 4.36e-03
100_ID.114_Q5_SLLQQLIGL
KD: 2.38e-07
Kass: 1.02e+05, kdiss: 2.42e-02
value
15000
10000
5000
0
0
250
500
750
1000
Time

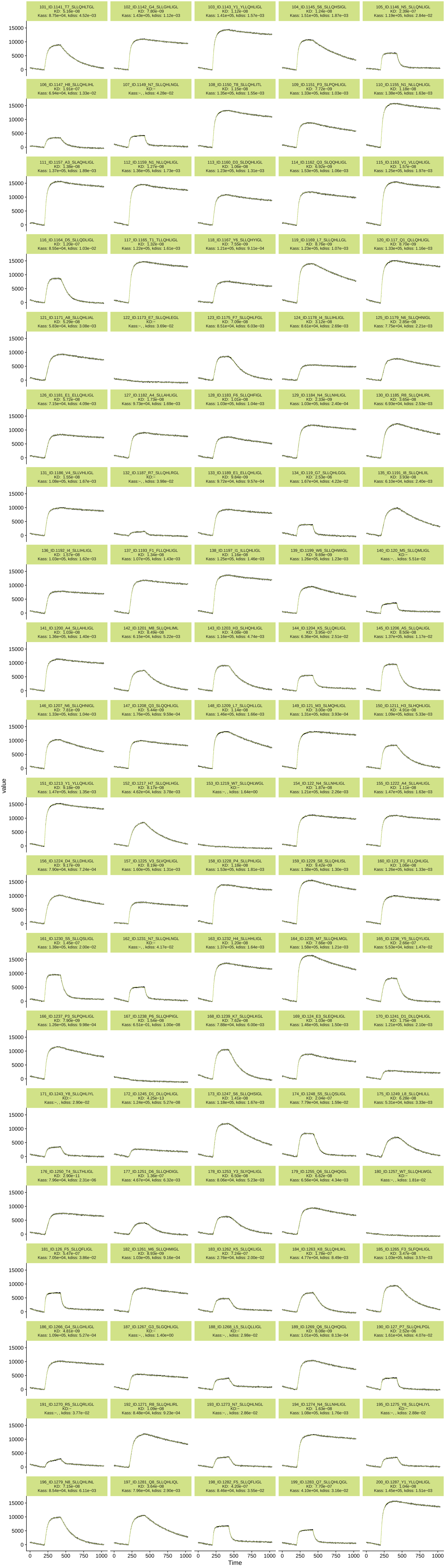
101_ID.1141_T7_SLLQHLTGL KD: 5.16e-08 Kass: 8.75e+04, kdiss: 4.52e-03
102_ID.1142_G4_SLLGHLIGL KD: 7.80e-09 Kass: 1.43e+05, kdiss: 1.12e-03
103_ID.1143_Y1_YLLQHLIGL KD: 1.12e-08 Kass: 1.41e+05, kdiss: 1.57e-03
104_ID.1145_S6_SLLQHSIGL KD: 1.24e-08 Kass: 1.51e+05, kdiss: 1.87e-03
105_ID.1146_N5_SLLQNLIGL KD: 2.39e-07 Kass: 1.19e+05, kdiss: 2.84e-02
106_ID.1147_H8_SLLQHLIHL KD: 1.91e-07 Kass: 6.94e+04, kdiss: 1.33e-02
107_ID.1149_N7_SLLQHLNGL KD:~ Kass:~, , kdiss: 4.28e-02
108_ID.1150_T8_SLLQHLITL KD: 1.15e-08 Kass: 1.35e+05, kdiss: 1.55e-03
109_ID.1151_P3_SLPQHLIGL KD: 7.72e-09 Kass: 1.33e+05, kdiss: 1.03e-03
110_ID.1155_N1_NLLQHLIGL KD: 1.18e-08 Kass: 1.38e+05, kdiss: 1.63e-03
111_ID.1157_A3_SLAQHLIGL KD: 1.38e-08 Kass: 1.37e+05, kdiss: 1.89e-03
112_ID.1159_N1_NLLQHLIGL KD: 1.27e-08 Kass: 1.36e+05, kdiss: 1.73e-03
113_ID.1160_D3_SLDQHLIGL KD: 1.06e-08 Kass: 1.23e+05, kdiss: 1.31e-03
114_ID.1162_Q3_SLQQHLIGL KD: 6.92e-09 Kass: 1.53e+05, kdiss: 1.06e-03
115_ID.1163_V1_VLLQHLIGL KD: 1.57e-08 Kass: 1.25e+05, kdiss: 1.97e-03
116_ID.1164_D5_SLLQDLIGL KD: 1.20e-07 Kass: 8.55e+04, kdiss: 1.03e-02
117_ID.1165_T1_TLLQHLIGL KD: 1.32e-08 Kass: 1.22e+05, kdiss: 1.61e-03
118_ID.1167_Y6_SLLQHYIGL KD: 7.55e-09 Kass: 1.21e+05, kdiss: 9.11e-04
119_ID.1169_L7_SLLQHLLGL KD: 8.76e-09 Kass: 1.23e+05, kdiss: 1.07e-03
120_ID.1170_Q1_QLLQHLIGL KD: 8.70e-09 Kass: 1.33e+05, kdiss: 1.16e-03
121_ID.1171_A8_SLLQHLIAL KD: 5.29e-08 Kass: 5.83e+04, kdiss: 3.08e-03
122_ID.1173_E7_SLLQHLEGL KD:~ Kass:~, , kdiss: 3.69e-02
123_ID.1175_F7_SLLQHLFGL KD: 7.09e-08 Kass: 8.51e+04, kdiss: 6.03e-03
124_ID.1178_I4_SLLIHLIGL KD: 3.12e-08 Kass: 8.61e+04, kdiss: 2.69e-03
125_ID.1179_N6_SLLQHNIGL KD: 2.85e-08 Kass: 7.75e+04, kdiss: 2.21e-03
126_ID.1181_E1_ELLQHLIGL KD: 5.72e-08 Kass: 7.15e+04, kdiss: 4.09e-03
127_ID.1182_A4_SLLAHLIGL KD: 1.73e-08 Kass: 9.73e+04, kdiss: 1.69e-03
128_ID.1183_F6_SLLQHFIGL KD: 1.01e-08 Kass: 1.03e+05, kdiss: 1.04e-03
129_ID.1184_N4_SLLNHLIGL KD: 2.33e-09 Kass: 1.03e+05, kdiss: 2.40e-04
130_ID.1185_R8_SLLQHLIRL KD: 3.65e-08 Kass: 6.93e+04, kdiss: 2.53e-03
131_ID.1186_V4_SLLVHLIGL KD: 1.55e-08 Kass: 1.08e+05, kdiss: 1.67e-03
132_ID.1187_R7_SLLQHLRGL KD:~ Kass:~, , kdiss: 3.98e-02
133_ID.1189_E1_ELLQHLIGL KD: 9.84e-09 Kass: 9.72e+04, kdiss: 9.57e-04
134_ID.119_G7_SLLQHLGGL KD: 2.53e-06 Kass: 1.67e+04, kdiss: 4.22e-02
135_ID.1191_I8_SLLQHLIIL KD: 3.93e-08 Kass: 6.10e+04, kdiss: 2.40e-03
136_ID.1192_I4_SLLIHLIGL KD: 1.57e-08 Kass: 1.03e+05, kdiss: 1.62e-03
137_ID.1193_F1_FLLQHLIGL KD: 1.34e-08 Kass: 1.07e+05, kdiss: 1.43e-03
138_ID.1197_I1_ILLQHLIGL KD: 1.16e-08 Kass: 1.25e+05, kdiss: 1.46e-03
139_ID.1199_W6_SLLQHWIGL KD: 9.69e-09 Kass: 1.26e+05, kdiss: 1.23e-03
140_ID.120_M5_SLLQMLIGL KD:~ Kass:~, , kdiss: 5.51e-02
141_ID.1200_A4_SLLAHLIGL KD: 1.03e-08 Kass: 1.36e+05, kdiss: 1.40e-03
142_ID.1201_M8_SLLQHLIML KD: 8.49e-08 Kass: 6.15e+04, kdiss: 5.22e-03
143_ID.1203_H3_SLHQHLIGL KD: 4.08e-08 Kass: 1.16e+05, kdiss: 4.74e-03
144_ID.1204_K5_SLLQKLIGL KD: 3.95e-07 Kass: 6.36e+04, kdiss: 2.51e-02
145_ID.1206_A5_SLLQALIGL KD: 8.50e-08 Kass: 1.37e+05, kdiss: 1.17e-02
146_ID.1207_N6_SLLQHNIGL KD: 7.81e-09 Kass: 1.33e+05, kdiss: 1.04e-03
147_ID.1208_Q3_SLQQHLIGL KD: 5.44e-09 Kass: 1.76e+05, kdiss: 9.59e-04
148_ID.1209_L7_SLLQHLLGL KD: 1.14e-08 Kass: 1.46e+05, kdiss: 1.66e-03
149_ID.121_M3_SLMQHLIGL KD: 3.00e-09 Kass: 1.31e+05, kdiss: 3.93e-04
150_ID.1211_H3_SLHQHLIGL KD: 4.91e-08 Kass: 1.09e+05, kdiss: 5.33e-03
151_ID.1213_Y1_YLLQHLIGL KD: 9.18e-09 Kass: 1.47e+05, kdiss: 1.35e-03
152_ID.1217_H7_SLLQHLHGL KD: 8.17e-08 Kass: 4.62e+04, kdiss: 3.78e-03
153_ID.1219_W7_SLLQHLWGL KD:~ Kass:~, , kdiss: 1.64e+00
154_ID.122_N4_SLLNHLIGL KD: 1.87e-08 Kass: 1.21e+05, kdiss: 2.26e-03
155_ID.1222_A4_SLLAHLIGL KD: 1.11e-08 Kass: 1.47e+05, kdiss: 1.63e-03
156_ID.1224_D4_SLLDHLIGL KD: 9.17e-09 Kass: 7.90e+04, kdiss: 7.24e-04
157_ID.1225_V3_SLVQHLIGL KD: 8.19e-09 Kass: 1.60e+05, kdiss: 1.31e-03
158_ID.1228_P4_SLLPHLIGL KD: 1.18e-08 Kass: 1.53e+05, kdiss: 1.81e-03
159_ID.1229_S8_SLLQHLISL KD: 9.42e-09 Kass: 1.38e+05, kdiss: 1.30e-03
160_ID.123_F1_FLLQHLIGL KD: 1.06e-08 Kass: 1.26e+05, kdiss: 1.33e-03
161_ID.1230_S5_SLLQSLIGL KD: 1.45e-07 Kass: 1.38e+05, kdiss: 2.00e-02
162_ID.1231_N7_SLLQHLNGL KD:~ Kass:~, , kdiss: 4.17e-02
163_ID.1232_H4_SLLHHLIGL KD: 1.20e-08 Kass: 1.37e+05, kdiss: 1.64e-03
164_ID.1235_M7_SLLQHLMGL KD: 7.66e-09 Kass: 1.58e+05, kdiss: 1.21e-03
165_ID.1236_Y5_SLLQYLIGL KD: 2.66e-07 Kass: 5.53e+04, kdiss: 1.47e-02
166_ID.1237_P3_SLPQHLIGL KD: 7.90e-09 Kass: 1.26e+05, kdiss: 9.98e-04
167_ID.1238_P6_SLLQHPIGL KD: 1.54e-08 Kass: 6.51e-01, kdiss: 1.00e-08
168_ID.1239_K7_SLLQHLKGL KD: 7.62e-08 Kass: 7.88e+04, kdiss: 6.00e-03
169_ID.124_E3_SLEQHLIGL KD: 1.03e-08 Kass: 1.46e+05, kdiss: 1.50e-03
170_ID.1241_D1_DLLQHLIGL KD: 1.75e-08 Kass: 1.21e+05, kdiss: 2.10e-03
171_ID.1243_Y8_SLLQHLIYL KD:~ Kass:~, , kdiss: 2.90e-02
172_ID.1245_D1_DLLQHLIGL KD: 4.25e-13 Kass: 1.24e+05, kdiss: 5.27e-08
173_ID.1247_S6_SLLQHSIGL KD: 1.41e-08 Kass: 1.18e+05, kdiss: 1.67e-03
174_ID.1248_S5_SLLQSLIGL KD: 2.04e-07 Kass: 7.79e+04, kdiss: 1.59e-02
175_ID.1249_L8_SLLQHLILL KD: 6.26e-08 Kass: 5.31e+04, kdiss: 3.33e-03
176_ID.1250_T4_SLLTHLIGL KD: 2.90e-11 Kass: 7.96e+04, kdiss: 2.31e-06
177_ID.1251_D6_SLLQHDIGL KD: 1.36e-07 Kass: 4.67e+04, kdiss: 6.32e-03
178_ID.1253_Y3_SLYQHLIGL KD: 6.50e-08 Kass: 8.06e+04, kdiss: 5.23e-03
179_ID.1255_Q6_SLLQHQIGL KD: 6.62e-08 Kass: 6.56e+04, kdiss: 4.34e-03
180_ID.1257_W7_SLLQHLWGL KD:~ Kass:~, , kdiss: 1.81e-02
181_ID.126_F5_SLLQFLIGL KD: 5.47e-07 Kass: 7.05e+04, kdiss: 3.86e-02
182_ID.1261_M6_SLLQHMIGL KD: 8.93e-09 Kass: 1.03e+05, kdiss: 9.16e-04
183_ID.1262_K5_SLLQKLIGL KD: 7.24e-07 Kass: 2.76e+04, kdiss: 2.00e-02
184_ID.1263_K8_SLLQHLIKL KD: 1.78e-07 Kass: 4.77e+04, kdiss: 8.49e-03
185_ID.1265_F3_SLFQHLIGL KD: 3.47e-08 Kass: 1.03e+05, kdiss: 3.57e-03
186_ID.1266_G4_SLLGHLIGL KD: 4.81e-09 Kass: 1.09e+05, kdiss: 5.27e-04
187_ID.1267_G3_SLGQHLIGL KD:~ Kass:~, , kdiss: 1.40e+00
188_ID.1268_L5_SLLQLLIGL KD:~ Kass:~, , kdiss: 2.98e-02
189_ID.1269_Q6_SLLQHQIGL KD: 8.08e-09 Kass: 1.01e+05, kdiss: 8.13e-04
190_ID.127_P7_SLLQHLPGL KD: 2.52e-06 Kass: 1.61e+04, kdiss: 4.07e-02
191_ID.1270_R5_SLLQRLIGL KD:~ Kass:~, , kdiss: 3.77e-02
192_ID.1271_R8_SLLQHLIRL KD: 1.09e-08 Kass: 8.48e+04, kdiss: 9.23e-04
193_ID.1273_N7_SLLQHLNGL KD:~ Kass:~, , kdiss: 2.86e-02
194_ID.1274_N4_SLLNHLIGL KD: 1.63e-08 Kass: 1.08e+05, kdiss: 1.76e-03
195_ID.1275_Y8_SLLQHLIYL KD:~ Kass:~, , kdiss: 2.88e-02
196_ID.1279_N8_SLLQHLINL KD: 7.15e-08 Kass: 8.54e+04, kdiss: 6.11e-03
197_ID.1281_Q8_SLLQHLIQL KD: 3.64e-08 Kass: 7.96e+04, kdiss: 2.90e-03
198_ID.1282_F5_SLLQFLIGL KD: 4.20e-07 Kass: 8.46e+04, kdiss: 3.55e-02
199_ID.1283_Q7_SLLQHLQGL KD: 7.70e-07 Kass: 4.10e+04, kdiss: 3.16e-02
200_ID.1287_Y1_YLLQHLIGL KD: 1.04e-08 Kass: 1.45e+05, kdiss: 1.51e-03
value
0 5000 10000 15000
Time
0 250 500 750 1000

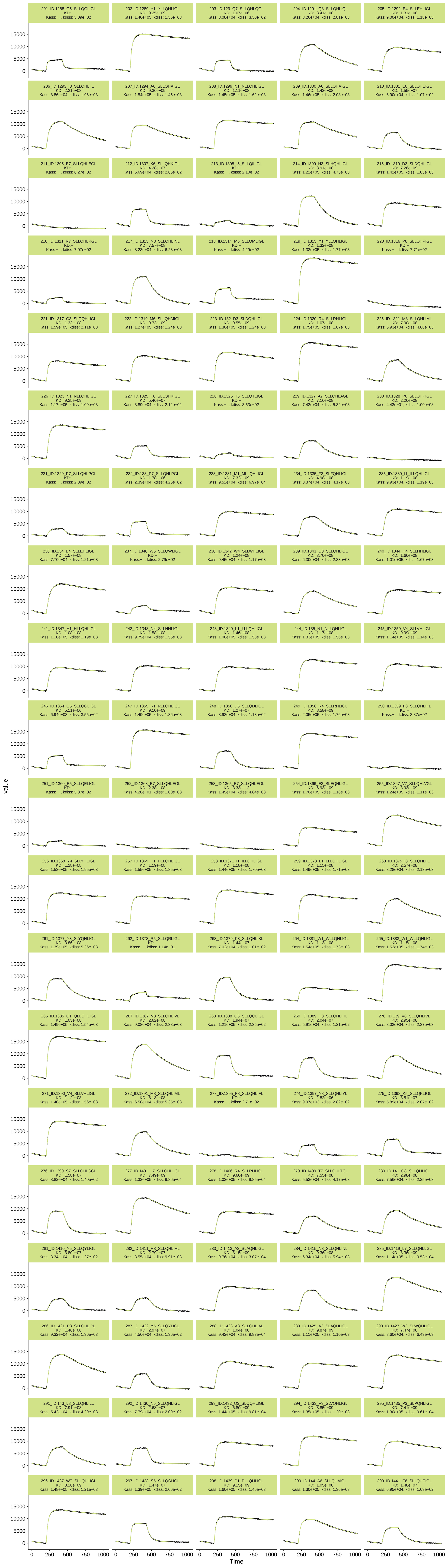
201_ID.1288_G5_SLLQGLIGL KD:− Kass:−, , kdiss: 5.09e−02
202_ID.1289_Y1_YLLQHLIGL KD: 9.25e−09 Kass: 1.46e+05, kdiss: 1.35e−03
203_ID.129_Q7_SLLQHLQGL KD: 1.07e−06 Kass: 3.08e+04, kdiss: 3.30e−02
204_ID.1291_Q8_SLLQHLIQL KD: 3.41e−08 Kass: 8.26e+04, kdiss: 2.81e−03
205_ID.1292_E4_SLLEHLIGL KD: 1.31e−08 Kass: 9.00e+04, kdiss: 1.18e−03
206_ID.1293_I8_SLLQHLIIL KD: 2.21e−08 Kass: 8.86e+04, kdiss: 1.96e−03
207_ID.1294_A6_SLLQHAIGL KD: 9.36e−09 Kass: 1.54e+05, kdiss: 1.45e−03
208_ID.1299_N1_NLLQHLIGL KD: 1.11e−08 Kass: 1.45e+05, kdiss: 1.62e−03
209_ID.1300_A6_SLLQHAIGL KD: 1.42e−08 Kass: 1.46e+05, kdiss: 2.08e−03
210_ID.1301_E6_SLLQHEIGL KD: 1.55e−07 Kass: 6.90e+04, kdiss: 1.07e−02
211_ID.1305_E7_SLLQHLEGL KD:− Kass:−, , kdiss: 6.27e−02
212_ID.1307_K6_SLLQHKIGL KD: 4.28e−07 Kass: 6.69e+04, kdiss: 2.86e−02
213_ID.1308_I5_SLLQILIGL KD:− Kass:−, , kdiss: 2.10e−02
214_ID.1309_H3_SLHQHLIGL KD: 3.91e−08 Kass: 1.22e+05, kdiss: 4.75e−03
215_ID.1310_D3_SLDQHLIGL KD: 7.26e−09 Kass: 1.42e+05, kdiss: 1.03e−03
216_ID.1311_R7_SLLQHLRGL KD:− Kass:−, , kdiss: 7.07e−02
217_ID.1313_N8_SLLQHLINL KD: 7.57e−08 Kass: 8.23e+04, kdiss: 6.23e−03
218_ID.1314_M5_SLLQMLIGL KD:− Kass:−, , kdiss: 4.29e−02
219_ID.1315_Y1_YLLQHLIGL KD: 1.32e−08 Kass: 1.33e+05, kdiss: 1.77e−03
220_ID.1316_P6_SLLQHPIGL KD:− Kass:−, , kdiss: 7.71e−02
221_ID.1317_G3_SLGQHLIGL KD: 1.33e−08 Kass: 1.59e+05, kdiss: 2.11e−03
222_ID.1319_M6_SLLQHMIGL KD: 9.73e−09 Kass: 1.27e+05, kdiss: 1.24e−03
223_ID.132_D3_SLDQHLIGL KD: 9.55e−09 Kass: 1.30e+05, kdiss: 1.24e−03
224_ID.1320_R4_SLLRHLIGL KD: 1.07e−08 Kass: 1.75e+05, kdiss: 1.87e−03
225_ID.1321_M8_SLLQHLIML KD: 7.90e−08 Kass: 5.93e+04, kdiss: 4.68e−03
226_ID.1323_N1_NLLQHLIGL KD: 9.25e−09 Kass: 1.17e+05, kdiss: 1.09e−03
227_ID.1325_K6_SLLQHKIGL KD: 5.46e−07 Kass: 3.89e+04, kdiss: 2.12e−02
228_ID.1326_T5_SLLQTLIGL KD:− Kass:−, , kdiss: 3.53e−02
229_ID.1327_A7_SLLQHLAGL KD: 7.16e−08 Kass: 7.43e+04, kdiss: 5.32e−03
230_ID.1328_P6_SLLQHPIGL KD: 2.26e−08 Kass: 4.43e−01, kdiss: 1.00e−08
231_ID.1329_P7_SLLQHLPGL KD:− Kass:−, , kdiss: 2.39e−02
232_ID.133_P7_SLLQHLPGL KD: 1.78e−06 Kass: 2.39e+04, kdiss: 4.26e−02
233_ID.1331_M1_MLLQHLIGL KD: 7.32e−09 Kass: 9.52e+04, kdiss: 6.97e−04
234_ID.1335_F3_SLFQHLIGL KD: 4.98e−08 Kass: 8.37e+04, kdiss: 4.17e−03
235_ID.1339_I1_ILLQHLIGL KD: 1.19e−08 Kass: 9.93e+04, kdiss: 1.19e−03
236_ID.134_E4_SLLEHLIGL KD: 1.57e−08 Kass: 7.70e+04, kdiss: 1.21e−03
237_ID.1340_W5_SLLQWLIGL KD:− Kass:−, , kdiss: 2.79e−02
238_ID.1342_W4_SLLWHLIGL KD: 1.24e−08 Kass: 9.45e+04, kdiss: 1.17e−03
239_ID.1343_Q8_SLLQHLIQL KD: 3.70e−08 Kass: 6.30e+04, kdiss: 2.33e−03
240_ID.1344_H4_SLLHHLIGL KD: 1.66e−08 Kass: 1.01e+05, kdiss: 1.67e−03
241_ID.1347_H1_HLLQHLIGL KD: 1.08e−08 Kass: 1.10e+05, kdiss: 1.19e−03
242_ID.1348_N4_SLLNHLIGL KD: 1.58e−08 Kass: 9.79e+04, kdiss: 1.55e−03
243_ID.1349_L1_LLLQHLIGL KD: 1.46e−08 Kass: 1.08e+05, kdiss: 1.58e−03
244_ID.135_N1_NLLQHLIGL KD: 1.17e−08 Kass: 1.33e+05, kdiss: 1.56e−03
245_ID.1350_V4_SLLVHLIGL KD: 9.99e−09 Kass: 1.14e+05, kdiss: 1.14e−03
246_ID.1354_G5_SLLQGLIGL KD: 5.11e−06 Kass: 6.94e+03, kdiss: 3.55e−02
247_ID.1355_R1_RLLQHLIGL KD: 9.10e−09 Kass: 1.49e+05, kdiss: 1.36e−03
248_ID.1356_D5_SLLQDLIGL KD: 1.27e−07 Kass: 8.92e+04, kdiss: 1.13e−02
249_ID.1358_R4_SLLRHLIGL KD: 8.58e−09 Kass: 2.05e+05, kdiss: 1.76e−03
250_ID.1359_F8_SLLQHLIFL KD:− Kass:−, , kdiss: 3.87e−02
251_ID.1360_E5_SLLQELIGL KD:− Kass:−, , kdiss: 5.37e−02
252_ID.1363_E7_SLLQHLEGL KD: 2.38e−08 Kass: 4.20e−01, kdiss: 1.00e−08
253_ID.1365_E7_SLLQHLEGL KD: 3.33e−12 Kass: 1.45e+04, kdiss: 4.84e−08
254_ID.1366_E3_SLEQHLIGL KD: 6.93e−09 Kass: 1.70e+05, kdiss: 1.18e−03
255_ID.1367_V7_SLLQHLVGL KD: 8.93e−09 Kass: 1.24e+05, kdiss: 1.11e−03
256_ID.1368_Y4_SLLYHLIGL KD: 1.28e−08 Kass: 1.53e+05, kdiss: 1.95e−03
257_ID.1369_H1_HLLQHLIGL KD: 1.19e−08 Kass: 1.55e+05, kdiss: 1.85e−03
258_ID.1371_I1_ILLQHLIGL KD: 1.18e−08 Kass: 1.44e+05, kdiss: 1.70e−03
259_ID.1373_L1_LLLQHLIGL KD: 1.15e−08 Kass: 1.49e+05, kdiss: 1.71e−03
260_ID.1375_I8_SLLQHLIIL KD: 2.57e−08 Kass: 8.28e+04, kdiss: 2.13e−03
261_ID.1377_Y3_SLYQHLIGL KD: 3.86e−08 Kass: 1.39e+05, kdiss: 5.36e−03
262_ID.1378_R5_SLLQRLIGL KD:− Kass:−, , kdiss: 1.14e−01
263_ID.1379_K8_SLLQHLIKL KD: 1.44e−07 Kass: 7.02e+04, kdiss: 1.01e−02
264_ID.1381_W1_WLLQHLIGL KD: 1.13e−08 Kass: 1.54e+05, kdiss: 1.73e−03
265_ID.1383_W1_WLLQHLIGL KD: 1.15e−08 Kass: 1.52e+05, kdiss: 1.74e−03
266_ID.1385_Q1_QLLQHLIGL KD: 1.03e−08 Kass: 1.49e+05, kdiss: 1.54e−03
267_ID.1387_V8_SLLQHLIVL KD: 2.62e−08 Kass: 9.08e+04, kdiss: 2.38e−03
268_ID.1388_Q5_SLLQQLIGL KD: 1.94e−07 Kass: 1.21e+05, kdiss: 2.35e−02
269_ID.1389_H8_SLLQHLIHL KD: 2.04e−07 Kass: 5.91e+04, kdiss: 1.21e−02
270_ID.139_V8_SLLQHLIVL KD: 2.95e−08 Kass: 8.02e+04, kdiss: 2.37e−03
271_ID.1390_V4_SLLVHLIGL KD: 1.12e−08 Kass: 1.40e+05, kdiss: 1.56e−03
272_ID.1391_M8_SLLQHLIML KD: 8.13e−08 Kass: 6.58e+04, kdiss: 5.35e−03
273_ID.1395_F8_SLLQHLIFL KD:− Kass:−, , kdiss: 2.71e−02
274_ID.1397_Y8_SLLQHLIYL KD: 2.82e−06 Kass: 9.97e+03, kdiss: 2.82e−02
275_ID.1398_K5_SLLQKLIGL KD: 3.51e−07 Kass: 5.89e+04, kdiss: 2.07e−02
276_ID.1399_S7_SLLQHLSGL KD: 1.58e−07 Kass: 8.82e+04, kdiss: 1.40e−02
277_ID.1401_L7_SLLQHLLGL KD: 7.49e−09 Kass: 1.32e+05, kdiss: 9.86e−04
278_ID.1406_R4_SLLRHLIGL KD: 9.60e−09 Kass: 1.03e+05, kdiss: 9.85e−04
279_ID.1409_T7_SLLQHLTGL KD: 7.55e−08 Kass: 5.53e+04, kdiss: 4.17e−03
280_ID.141_Q8_SLLQHLIQL KD: 2.98e−08 Kass: 7.56e+04, kdiss: 2.25e−03
281_ID.1410_Y5_SLLQYLIGL KD: 3.80e−07 Kass: 3.34e+04, kdiss: 1.27e−02
282_ID.1411_H8_SLLQHLIHL KD: 2.79e−07 Kass: 3.55e+04, kdiss: 9.91e−03
283_ID.1413_A3_SLAQHLIGL KD: 3.15e−09 Kass: 9.76e+04, kdiss: 3.07e−04
284_ID.1415_N8_SLLQHLINL KD: 9.36e−08 Kass: 6.34e+04, kdiss: 5.94e−03
285_ID.1419_L7_SLLQHLLGL KD: 8.36e−09 Kass: 1.14e+05, kdiss: 9.53e−04
286_ID.1421_P8_SLLQHLIPL KD: 1.46e−08 Kass: 9.32e+04, kdiss: 1.36e−03
287_ID.1422_Y5_SLLQYLIGL KD: 2.97e−07 Kass: 4.56e+04, kdiss: 1.36e−02
288_ID.1423_A8_SLLQHLIAL KD: 1.04e−08 Kass: 9.42e+04, kdiss: 9.83e−04
289_ID.1425_A3_SLAQHLIGL KD: 9.87e−09 Kass: 1.11e+05, kdiss: 1.10e−03
290_ID.1427_W3_SLWQHLIGL KD: 7.47e−08 Kass: 8.60e+04, kdiss: 6.43e−03
291_ID.143_L8_SLLQHLILL KD: 7.91e−08 Kass: 5.42e+04, kdiss: 4.29e−03
292_ID.1430_N5_SLLQNLIGL KD: 2.68e−07 Kass: 7.79e+04, kdiss: 2.09e−02
293_ID.1432_Q3_SLQQHLIGL KD: 6.80e−09 Kass: 1.44e+05, kdiss: 9.81e−04
294_ID.1433_V3_SLVQHLIGL KD: 8.85e−09 Kass: 1.35e+05, kdiss: 1.20e−03
295_ID.1435_P3_SLPQHLIGL KD: 7.41e−09 Kass: 1.30e+05, kdiss: 9.61e−04
296_ID.1437_WT_SLLQHLIGL KD: 8.18e−09 Kass: 1.48e+05, kdiss: 1.21e−03
297_ID.1438_S5_SLLQSLIGL KD: 1.47e−07 Kass: 1.39e+05, kdiss: 2.06e−02
298_ID.1439_P1_PLLQHLIGL KD: 9.15e−09 Kass: 1.60e+05, kdiss: 1.46e−03
299_ID.144_A6_SLLQHAIGL KD: 1.05e−08 Kass: 1.30e+05, kdiss: 1.36e−03
300_ID.1441_E6_SLLQHEIGL KD: 1.48e−07 Kass: 6.95e+04, kdiss: 1.03e−02
value
15000
10000
5000
0
0 250 500 750 1000
Time

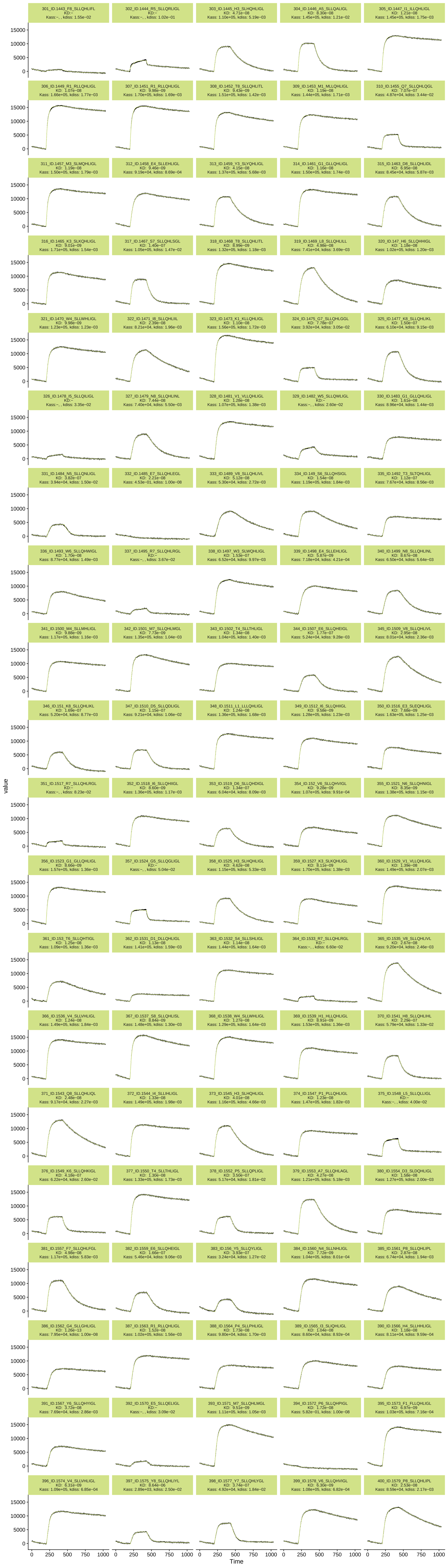
301_ID.1443_F8_SLLQHLIFL
KD:−
Kass:−, , kdiss: 1.55e−02
302_ID.1444_R5_SLLQRLIGL
KD:−
Kass:−, , kdiss: 1.02e−01
303_ID.1445_H3_SLHQHLIGL
KD: 4.71e−08
Kass: 1.10e+05, kdiss: 5.19e−03
304_ID.1446_A5_SLLQALIGL
KD: 8.30e−08
Kass: 1.45e+05, kdiss: 1.21e−02
305_ID.1447_I1_ILLQHLIGL
KD: 1.21e−08
Kass: 1.45e+05, kdiss: 1.75e−03
306_ID.1449_R1_RLLQHLIGL
KD: 1.07e−08
Kass: 1.66e+05, kdiss: 1.77e−03
307_ID.1451_R1_RLLQHLIGL
KD: 9.98e−09
Kass: 1.70e+05, kdiss: 1.69e−03
308_ID.1452_T8_SLLQHLITL
KD: 9.43e−09
Kass: 1.51e+05, kdiss: 1.42e−03
309_ID.1453_M1_MLLQHLIGL
KD: 1.19e−08
Kass: 1.44e+05, kdiss: 1.71e−03
310_ID.1455_Q7_SLLQHLQGL
KD: 7.07e−07
Kass: 4.87e+04, kdiss: 3.44e−02
311_ID.1457_M3_SLMQHLIGL
KD: 1.19e−08
Kass: 1.50e+05, kdiss: 1.79e−03
312_ID.1458_E4_SLLEHLIGL
KD: 9.46e−09
Kass: 9.19e+04, kdiss: 8.69e−04
313_ID.1459_Y3_SLYQHLIGL
KD: 4.15e−08
Kass: 1.37e+05, kdiss: 5.68e−03
314_ID.1461_G1_GLLQHLIGL
KD: 1.16e−08
Kass: 1.50e+05, kdiss: 1.74e−03
315_ID.1463_D8_SLLQHLIDL
KD: 6.95e−08
Kass: 8.45e+04, kdiss: 5.87e−03
316_ID.1465_K3_SLKQHLIGL
KD: 9.01e−09
Kass: 1.71e+05, kdiss: 1.54e−03
317_ID.1467_S7_SLLQHLSGL
KD: 1.40e−07
Kass: 1.05e+05, kdiss: 1.47e−02
318_ID.1468_T8_SLLQHLITL
KD: 8.99e−09
Kass: 1.32e+05, kdiss: 1.18e−03
319_ID.1469_L8_SLLQHLILL
KD: 4.98e−08
Kass: 7.41e+04, kdiss: 3.69e−03
320_ID.147_H6_SLLQHHIGL
KD: 1.18e−08
Kass: 1.02e+05, kdiss: 1.20e−03
321_ID.1470_W4_SLLWHLIGL
KD: 9.98e−09
Kass: 1.23e+05, kdiss: 1.23e−03
322_ID.1471_I8_SLLQHLIIL
KD: 2.39e−08
Kass: 8.21e+04, kdiss: 1.96e−03
323_ID.1473_K1_KLLQHLIGL
KD: 1.10e−08
Kass: 1.56e+05, kdiss: 1.72e−03
324_ID.1475_G7_SLLQHLGGL
KD: 7.78e−07
Kass: 3.92e+04, kdiss: 3.05e−02
325_ID.1477_K8_SLLQHLIKL
KD: 1.50e−07
Kass: 6.10e+04, kdiss: 9.15e−03
326_ID.1478_I5_SLLQILIGL
KD:−
Kass:−, , kdiss: 3.35e−02
327_ID.1479_N8_SLLQHLINL
KD: 7.44e−08
Kass: 7.40e+04, kdiss: 5.50e−03
328_ID.1481_V1_VLLQHLIGL
KD: 1.28e−08
Kass: 1.07e+05, kdiss: 1.38e−03
329_ID.1482_W5_SLLQWLIGL
KD:−
Kass:−, , kdiss: 2.60e−02
330_ID.1483_G1_GLLQHLIGL
KD: 1.61e−08
Kass: 8.96e+04, kdiss: 1.44e−03
331_ID.1484_N5_SLLQNLIGL
KD: 3.82e−07
Kass: 3.94e+04, kdiss: 1.50e−02
332_ID.1485_E7_SLLQHLEGL
KD: 2.21e−08
Kass: 4.53e−01, kdiss: 1.00e−08
333_ID.1489_V8_SLLQHLIVL
KD: 5.12e−08
Kass: 5.30e+04, kdiss: 2.72e−03
334_ID.149_S6_SLLQHSIGL
KD: 1.54e−08
Kass: 1.19e+05, kdiss: 1.84e−03
335_ID.1492_T3_SLTQHLIGL
KD: 1.12e−07
Kass: 7.67e+04, kdiss: 8.56e−03
336_ID.1493_W6_SLLQHWIGL
KD: 1.70e−08
Kass: 8.77e+04, kdiss: 1.49e−03
337_ID.1495_R7_SLLQHLRGL
KD:−
Kass:−, , kdiss: 3.67e−02
338_ID.1497_W3_SLWQHLIGL
KD: 1.53e−07
Kass: 6.52e+04, kdiss: 9.97e−03
339_ID.1498_E4_SLLEHLIGL
KD: 5.87e−09
Kass: 7.18e+04, kdiss: 4.21e−04
340_ID.1499_N8_SLLQHLINL
KD: 8.67e−08
Kass: 6.50e+04, kdiss: 5.64e−03
341_ID.1500_M4_SLLMHLIGL
KD: 9.88e−09
Kass: 1.17e+05, kdiss: 1.16e−03
342_ID.1501_M7_SLLQHLMGL
KD: 7.73e−09
Kass: 1.35e+05, kdiss: 1.04e−03
343_ID.1502_T4_SLLTHLIGL
KD: 1.34e−08
Kass: 1.04e+05, kdiss: 1.40e−03
344_ID.1507_E6_SLLQHEIGL
KD: 1.77e−07
Kass: 5.24e+04, kdiss: 9.28e−03
345_ID.1509_V8_SLLQHLIVL
KD: 2.95e−08
Kass: 8.01e+04, kdiss: 2.36e−03
346_ID.151_K8_SLLQHLIKL
KD: 1.69e−07
Kass: 5.20e+04, kdiss: 8.77e−03
347_ID.1510_D5_SLLQDLIGL
KD: 1.15e−07
Kass: 9.21e+04, kdiss: 1.06e−02
348_ID.1511_L1_LLLQHLIGL
KD: 1.24e−08
Kass: 1.36e+05, kdiss: 1.68e−03
349_ID.1512_I6_SLLQHIIGL
KD: 9.58e−09
Kass: 1.28e+05, kdiss: 1.23e−03
350_ID.1516_E3_SLEQHLIGL
KD: 7.68e−09
Kass: 1.63e+05, kdiss: 1.25e−03
351_ID.1517_R7_SLLQHLRGL
KD:−
Kass:−, , kdiss: 8.23e−02
352_ID.1518_I6_SLLQHIIGL
KD: 8.60e−09
Kass: 1.36e+05, kdiss: 1.17e−03
353_ID.1519_D6_SLLQHDIGL
KD: 1.34e−07
Kass: 6.04e+04, kdiss: 8.09e−03
354_ID.152_V6_SLLQHVIGL
KD: 9.28e−09
Kass: 1.07e+05, kdiss: 9.91e−04
355_ID.1521_N6_SLLQHNIGL
KD: 8.35e−09
Kass: 1.38e+05, kdiss: 1.15e−03
356_ID.1523_G1_GLLQHLIGL
KD: 8.66e−09
Kass: 1.57e+05, kdiss: 1.36e−03
357_ID.1524_G5_SLLQGLIGL
KD:−
Kass:−, , kdiss: 5.04e−02
358_ID.1525_H3_SLHQHLIGL
KD: 4.62e−08
Kass: 1.15e+05, kdiss: 5.33e−03
359_ID.1527_K3_SLKQHLIGL
KD: 8.11e−09
Kass: 1.70e+05, kdiss: 1.38e−03
360_ID.1529_V1_VLLQHLIGL
KD: 1.39e−08
Kass: 1.49e+05, kdiss: 2.07e−03
361_ID.153_T6_SLLQHTIGL
KD: 1.25e−08
Kass: 1.09e+05, kdiss: 1.36e−03
362_ID.1531_D1_DLLQHLIGL
KD: 1.13e−08
Kass: 1.41e+05, kdiss: 1.59e−03
363_ID.1532_S4_SLLSHLIGL
KD: 1.14e−08
Kass: 1.44e+05, kdiss: 1.64e−03
364_ID.1533_R7_SLLQHLRGL
KD:−
Kass:−, , kdiss: 6.60e−02
365_ID.1535_V8_SLLQHLIVL
KD: 2.67e−08
Kass: 9.20e+04, kdiss: 2.46e−03
366_ID.1536_V4_SLLVHLIGL
KD: 1.24e−08
Kass: 1.49e+05, kdiss: 1.84e−03
367_ID.1537_S8_SLLQHLISL
KD: 8.84e−09
Kass: 1.48e+05, kdiss: 1.30e−03
368_ID.1538_W4_SLLWHLIGL
KD: 1.27e−08
Kass: 1.29e+05, kdiss: 1.64e−03
369_ID.1539_H1_HLLQHLIGL
KD: 8.91e−09
Kass: 1.53e+05, kdiss: 1.36e−03
370_ID.1541_H8_SLLQHLIHL
KD: 2.29e−07
Kass: 5.79e+04, kdiss: 1.33e−02
371_ID.1543_Q8_SLLQHLIQL
KD: 2.48e−08
Kass: 9.17e+04, kdiss: 2.27e−03
372_ID.1544_I4_SLLIHLIGL
KD: 1.33e−08
Kass: 1.49e+05, kdiss: 1.98e−03
373_ID.1545_H3_SLHQHLIGL
KD: 4.01e−08
Kass: 1.16e+05, kdiss: 4.66e−03
374_ID.1547_P1_PLLQHLIGL
KD: 1.23e−08
Kass: 1.47e+05, kdiss: 1.82e−03
375_ID.1548_L5_SLLQLLIGL
KD:−
Kass:−, , kdiss: 4.00e−02
376_ID.1549_K6_SLLQHKIGL
KD: 4.18e−07
Kass: 6.22e+04, kdiss: 2.60e−02
377_ID.1550_T4_SLLTHLIGL
KD: 1.30e−08
Kass: 1.33e+05, kdiss: 1.73e−03
378_ID.1552_P5_SLLQPLIGL
KD: 3.50e−07
Kass: 5.17e+04, kdiss: 1.81e−02
379_ID.1553_A7_SLLQHLAGL
KD: 4.27e−08
Kass: 1.21e+05, kdiss: 5.18e−03
380_ID.1554_D3_SLDQHLIGL
KD: 1.58e−08
Kass: 1.27e+05, kdiss: 2.00e−03
381_ID.1557_F7_SLLQHLFGL
KD: 4.98e−08
Kass: 1.17e+05, kdiss: 5.83e−03
382_ID.1559_E6_SLLQHEIGL
KD: 1.66e−07
Kass: 5.46e+04, kdiss: 9.06e−03
383_ID.156_Y5_SLLQYLIGL
KD: 3.93e−07
Kass: 3.24e+04, kdiss: 1.27e−02
384_ID.1560_N4_SLLNHLIGL
KD: 7.72e−09
Kass: 1.04e+05, kdiss: 8.01e−04
385_ID.1561_P8_SLLQHLIPL
KD: 2.87e−08
Kass: 6.74e+04, kdiss: 1.94e−03
386_ID.1562_G4_SLLGHLIGL
KD: 1.26e−13
Kass: 7.95e+04, kdiss: 1.00e−08
387_ID.1563_R1_RLLQHLIGL
KD: 1.52e−08
Kass: 1.02e+05, kdiss: 1.56e−03
388_ID.1564_P4_SLLPHLIGL
KD: 1.73e−08
Kass: 9.80e+04, kdiss: 1.70e−03
389_ID.1565_I3_SLIQHLIGL
KD: 1.04e−08
Kass: 8.60e+04, kdiss: 8.92e−04
390_ID.1566_H4_SLLHHLIGL
KD: 1.18e−08
Kass: 8.11e+04, kdiss: 9.59e−04
391_ID.1567_Y6_SLLQHYIGL
KD: 3.72e−08
Kass: 7.69e+04, kdiss: 2.86e−03
392_ID.1570_E5_SLLQELIGL
KD:−
Kass:−, , kdiss: 3.09e−02
393_ID.1571_M7_SLLQHLMGL
KD: 9.51e−09
Kass: 1.11e+05, kdiss: 1.05e−03
394_ID.1572_P6_SLLQHPIGL
KD: 1.72e−08
Kass: 5.82e−01, kdiss: 1.00e−08
395_ID.1573_F1_FLLQHLIGL
KD: 6.97e−09
Kass: 1.03e+05, kdiss: 7.16e−04
396_ID.1574_V4_SLLVHLIGL
KD: 6.31e−09
Kass: 1.09e+05, kdiss: 6.85e−04
397_ID.1575_Y8_SLLQHLIYL
KD: 8.64e−06
Kass: 2.89e+03, kdiss: 2.50e−02
398_ID.1577_Y7_SLLQHLYGL
KD: 3.74e−07
Kass: 4.92e+04, kdiss: 1.84e−02
399_ID.1578_V6_SLLQHVIGL
KD: 6.30e−09
Kass: 1.08e+05, kdiss: 6.82e−04
400_ID.1579_P8_SLLQHLIPL
KD: 2.53e−08
Kass: 8.59e+04, kdiss: 2.17e−03
value
15000
10000
5000
0
0 250 500 750 1000
Time

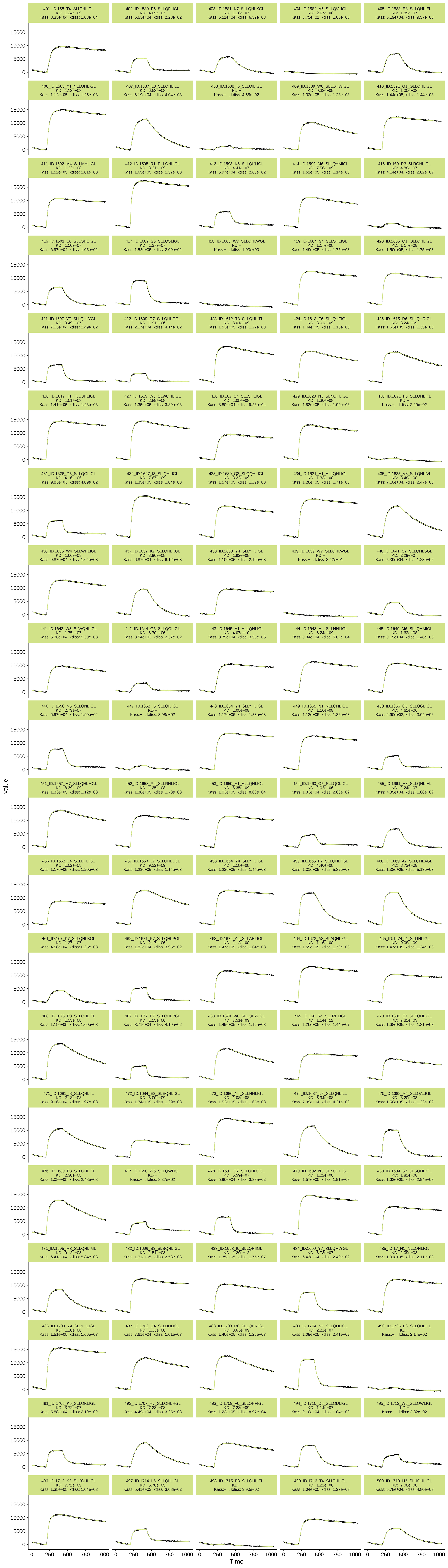

value
Time
0
5000
10000
15000
0
250
500
750
1000

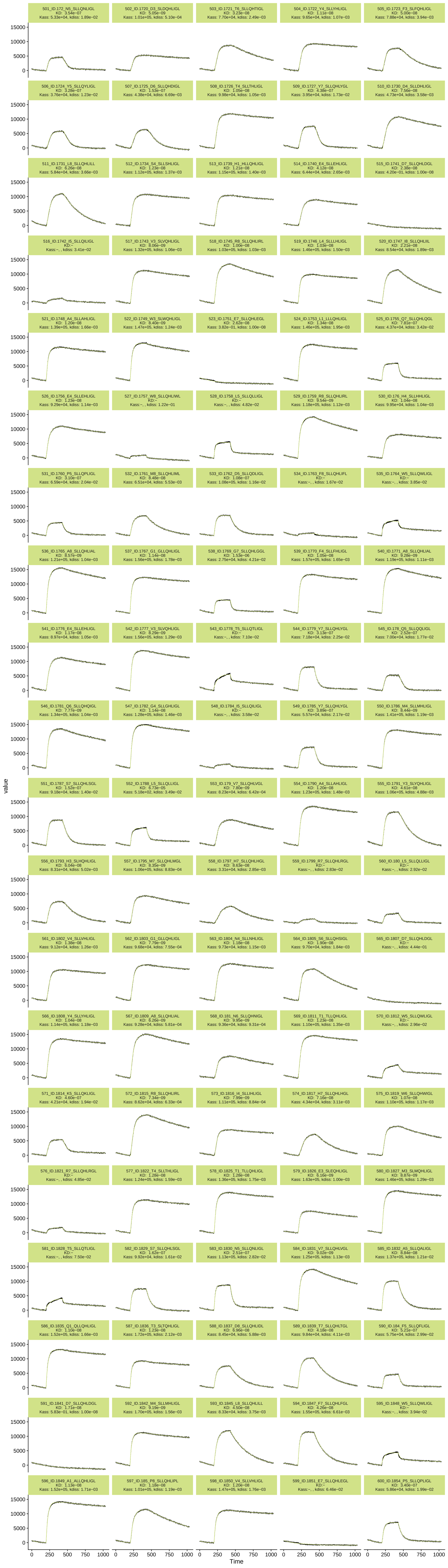

value
Time

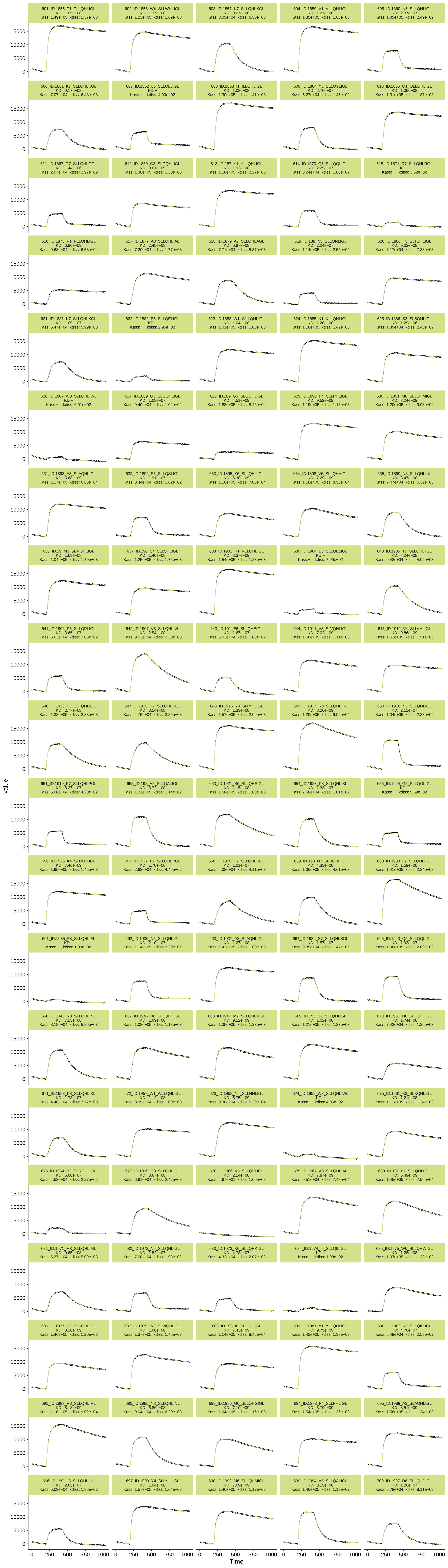
601_ID.1855_T1_TLLQHLIGL
KD: 1.05e−08
Kass: 1.49e+05, kdiss: 1.57e−03
602_ID.1856_W4_SLLWHLIGL
KD: 1.37e−08
Kass: 1.23e+05, kdiss: 1.69e−03
603_ID.1857_K7_SLLQHLKGL
KD: 8.07e−08
Kass: 8.05e+04, kdiss: 6.50e−03
604_ID.1859_V1_VLLQHLIGL
KD: 1.21e−08
Kass: 1.35e+05, kdiss: 1.63e−03
605_ID.1860_N5_SLLQNLIGL
KD: 2.37e−07
Kass: 1.05e+05, kdiss: 2.49e−02
606_ID.1861_K7_SLLQHLKGL
KD: 9.17e−08
Kass: 7.07e+04, kdiss: 6.48e−03
607_ID.1862_L5_SLLQLLIGL
KD:~
Kass:~, , kdiss: 4.00e−02
608_ID.1863_I1_ILLQHLIGL
KD: 1.08e−08
Kass: 1.30e+05, kdiss: 1.41e−03
609_ID.1864_Y5_SLLQYLIGL
KD: 2.76e−07
Kass: 5.27e+04, kdiss: 1.45e−02
610_ID.1865_Q1_QLLQHLIGL
KD: 1.05e−08
Kass: 1.31e+05, kdiss: 1.37e−03
611_ID.1867_G7_SLLQHLGGL
KD: 1.44e−06
Kass: 2.07e+04, kdiss: 2.97e−02
612_ID.1869_G3_SLGQHLIGL
KD: 9.61e−09
Kass: 1.36e+05, kdiss: 1.30e−03
613_ID.187_Y1_YLLQHLIGL
KD: 1.93e−08
Kass: 1.18e+05, kdiss: 2.27e−03
614_ID.1870_Q5_SLLQQLIGL
KD: 2.29e−07
Kass: 8.24e+04, kdiss: 1.88e−02
615_ID.1871_R7_SLLQHLRGL
KD:~
Kass:~, , kdiss: 3.62e−02
616_ID.1873_P1_PLLQHLIGL
KD: 6.80e−09
Kass: 9.68e+04, kdiss: 6.58e−04
617_ID.1877_A8_SLLQHLIAL
KD: 2.45e−08
Kass: 7.20e+04, kdiss: 1.77e−03
618_ID.1879_A7_SLLQHLAGL
KD: 6.97e−08
Kass: 7.71e+04, kdiss: 5.37e−03
619_ID.188_N5_SLLQNLIGL
KD: 2.24e−07
Kass: 1.14e+05, kdiss: 2.56e−02
620_ID.1880_T3_SLTQHLIGL
KD: 9.03e−09
Kass: 8.17e+04, kdiss: 7.38e−04
621_ID.1881_K7_SLLQHLKGL
KD: 1.09e−07
Kass: 5.47e+04, kdiss: 5.99e−03
622_ID.1882_E5_SLLQELIGL
KD:~
Kass:~, , kdiss: 2.95e−02
623_ID.1883_W1_WLLQHLIGL
KD: 1.04e−08
Kass: 1.01e+05, kdiss: 1.05e−03
624_ID.1885_K1_KLLQHLIGL
KD: 1.20e−08
Kass: 1.18e+05, kdiss: 1.42e−03
625_ID.1886_S3_SLSQHLIGL
KD: 1.29e−06
Kass: 1.89e+04, kdiss: 2.45e−02
626_ID.1887_W8_SLLQHLIWL
KD:~
Kass:~, , kdiss: 6.01e−02
627_ID.1889_G3_SLGQHLIGL
KD: 1.08e−07
Kass: 9.40e+04, kdiss: 1.02e−02
628_ID.189_G3_SLGQHLIGL
KD: 4.51e−09
Kass: 1.88e+05, kdiss: 8.46e−04
629_ID.1890_P4_SLLPHLIGL
KD: 9.52e−09
Kass: 1.19e+05, kdiss: 1.13e−03
630_ID.1891_M6_SLLQHMIGL
KD: 8.24e−09
Kass: 1.20e+05, kdiss: 9.93e−04
631_ID.1893_A3_SLAQHLIGL
KD: 5.68e−09
Kass: 1.17e+05, kdiss: 6.66e−04
632_ID.1894_S5_SLLQSLIGL
KD: 1.91e−07
Kass: 8.44e+04, kdiss: 1.62e−02
633_ID.1895_Y6_SLLQHYIGL
KD: 6.38e−09
Kass: 1.10e+05, kdiss: 7.03e−04
634_ID.1898_V6_SLLQHVIGL
KD: 7.29e−09
Kass: 1.18e+05, kdiss: 8.58e−04
635_ID.1899_N8_SLLQHLINL
KD: 8.47e−08
Kass: 7.47e+04, kdiss: 6.32e−03
636_ID.19_M3_SLMQHLIGL
KD: 1.63e−08
Kass: 1.04e+05, kdiss: 1.70e−03
637_ID.190_S4_SLLSHLIGL
KD: 1.40e−08
Kass: 1.25e+05, kdiss: 1.75e−03
638_ID.1901_R1_RLLQHLIGL
KD: 8.37e−09
Kass: 1.54e+05, kdiss: 1.28e−03
639_ID.1904_E5_SLLQELIGL
KD:~
Kass:~, , kdiss: 7.99e−02
640_ID.1905_T7_SLLQHLTGL
KD: 4.24e−08
Kass: 9.48e+04, kdiss: 4.02e−03
641_ID.1906_P5_SLLQPLIGL
KD: 3.65e−07
Kass: 5.63e+04, kdiss: 2.05e−02
642_ID.1907_V8_SLLQHLIVL
KD: 2.54e−08
Kass: 9.03e+04, kdiss: 2.30e−03
643_ID.191_E6_SLLQHEIGL
KD: 1.67e−07
Kass: 6.03e+04, kdiss: 1.00e−02
644_ID.1911_V3_SLVQHLIGL
KD: 7.02e−09
Kass: 1.58e+05, kdiss: 1.11e−03
645_ID.1912_V4_SLLVHLIGL
KD: 9.86e−09
Kass: 1.53e+05, kdiss: 1.51e−03
646_ID.1913_F3_SLFQHLIGL
KD: 2.77e−08
Kass: 1.38e+05, kdiss: 3.83e−03
647_ID.1915_H7_SLLQHLHGL
KD: 8.14e−08
Kass: 4.75e+04, kdiss: 3.86e−03
648_ID.1916_Y4_SLLYHLIGL
KD: 1.33e−08
Kass: 1.57e+05, kdiss: 2.09e−03
649_ID.1917_R8_SLLQHLIRL
KD: 8.26e−09
Kass: 1.20e+05, kdiss: 9.92e−04
650_ID.1918_N5_SLLQNLIGL
KD: 2.11e−07
Kass: 1.34e+05, kdiss: 2.83e−02
651_ID.1919_P7_SLLQHLPGL
KD: 8.27e−07
Kass: 5.08e+04, kdiss: 4.20e−02
652_ID.192_A5_SLLQALIGL
KD: 8.72e−08
Kass: 1.31e+05, kdiss: 1.14e−02
653_ID.1921_S6_SLLQHSIGL
KD: 1.15e−08
Kass: 1.56e+05, kdiss: 1.80e−03
654_ID.1923_K8_SLLQHLIKL
KD: 1.33e−07
Kass: 7.58e+04, kdiss: 1.01e−02
655_ID.1924_G5_SLLQGLIGL
KD:~
Kass:~, , kdiss: 5.59e−02
656_ID.1926_K4_SLLKHLIGL
KD: 7.46e−09
Kass: 1.95e+05, kdiss: 1.45e−03
657_ID.1927_P7_SLLQHLPGL
KD: 1.70e−06
Kass: 2.63e+04, kdiss: 4.46e−02
658_ID.1929_H7_SLLQHLHGL
KD: 1.01e−07
Kass: 4.08e+04, kdiss: 4.11e−03
659_ID.193_H3_SLHQHLIGL
KD: 4.33e−08
Kass: 1.06e+05, kdiss: 4.61e−03
660_ID.1933_L7_SLLQHLLGL
KD: 1.59e−08
Kass: 1.41e+05, kdiss: 2.24e−03
661_ID.1935_F8_SLLQHLIFL
KD:~
Kass:~, , kdiss: 1.99e−02
662_ID.1936_N5_SLLQNLIGL
KD: 2.10e−07
Kass: 1.14e+05, kdiss: 2.39e−02
663_ID.1937_A3_SLAQHLIGL
KD: 1.27e−08
Kass: 1.42e+05, kdiss: 1.80e−03
664_ID.1939_S7_SLLQHLSGL
KD: 1.58e−07
Kass: 9.35e+04, kdiss: 1.47e−02
665_ID.1940_Q5_SLLQQLIGL
KD: 1.93e−07
Kass: 1.08e+05, kdiss: 2.09e−02
666_ID.1943_N8_SLLQHLINL
KD: 7.15e−08
Kass: 8.19e+04, kdiss: 5.86e−03
667_ID.1945_H6_SLLQHHIGL
KD: 1.09e−08
Kass: 1.08e+05, kdiss: 1.18e−03
668_ID.1947_M7_SLLQHLMGL
KD: 9.12e−09
Kass: 1.35e+05, kdiss: 1.23e−03
669_ID.195_S8_SLLQHLISL
KD: 1.02e−08
Kass: 1.21e+05, kdiss: 1.23e−03
670_ID.1951_H6_SLLQHHIGL
KD: 1.74e−08
Kass: 7.42e+04, kdiss: 1.29e−03
671_ID.1953_K8_SLLQHLIKL
KD: 1.73e−07
Kass: 4.48e+04, kdiss: 7.77e−03
672_ID.1957_W1_WLLQHLIGL
KD: 1.12e−08
Kass: 8.95e+04, kdiss: 1.00e−03
673_ID.1958_N4_SLLNHLIGL
KD: 5.75e−09
Kass: 9.38e+04, kdiss: 5.39e−04
674_ID.1959_W8_SLLQHLIWL
KD:~
Kass:~, , kdiss: 4.06e−02
675_ID.1961_K3_SLKQHLIGL
KD: 1.21e−08
Kass: 1.11e+05, kdiss: 1.34e−03
676_ID.1964_R3_SLRQHLIGL
KD: 5.00e−07
Kass: 4.53e+04, kdiss: 2.27e−02
677_ID.1965_Q8_SLLQHLIQL
KD: 3.67e−08
Kass: 6.61e+04, kdiss: 2.42e−03
678_ID.1966_V5_SLLQVLIGL
KD: 2.14e−09
Kass: 4.67e−01, kdiss: 1.00e−08
679_ID.1967_A8_SLLQHLIAL
KD: 7.87e−09
Kass: 9.51e+04, kdiss: 7.48e−04
680_ID.197_L7_SLLQHLLGL
KD: 5.49e−09
Kass: 1.45e+05, kdiss: 7.96e−04
681_ID.1971_M8_SLLQHLIML
KD: 9.65e−08
Kass: 5.27e+04, kdiss: 5.09e−03
682_ID.1972_N5_SLLQNLIGL
KD: 2.82e−07
Kass: 7.00e+04, kdiss: 1.98e−02
683_ID.1973_K6_SLLQHKIGL
KD: 4.78e−07
Kass: 4.32e+04, kdiss: 2.07e−02
684_ID.1974_I5_SLLQILIGL
KD:~
Kass:~, , kdiss: 1.98e−02
685_ID.1975_W6_SLLQHWIGL
KD: 1.28e−08
Kass: 1.07e+05, kdiss: 1.38e−03
686_ID.1977_K3_SLKQHLIGL
KD: 8.20e−09
Kass: 1.46e+05, kdiss: 1.20e−03
687_ID.1979_W3_SLWQHLIGL
KD: 1.06e−08
Kass: 1.37e+05, kdiss: 1.45e−03
688_ID.198_I6_SLLQHIIGL
KD: 7.43e−09
Kass: 1.14e+05, kdiss: 8.45e−04
689_ID.1981_Y1_YLLQHLIGL
KD: 9.75e−09
Kass: 1.42e+05, kdiss: 1.38e−03
690_ID.1982_K5_SLLQKLIGL
KD: 4.70e−07
Kass: 5.49e+04, kdiss: 2.58e−02
691_ID.1983_R8_SLLQHLIRL
KD: 8.18e−09
Kass: 1.10e+05, kdiss: 9.02e−04
692_ID.1985_N8_SLLQHLINL
KD: 6.86e−08
Kass: 9.04e+04, kdiss: 6.20e−03
693_ID.1986_G6_SLLQHGIGL
KD: 7.10e−09
Kass: 1.64e+05, kdiss: 1.16e−03
694_ID.1988_F4_SLLFHLIGL
KD: 8.79e−09
Kass: 1.54e+05, kdiss: 1.36e−03
695_ID.1989_A3_SLAQHLIGL
KD: 8.51e−09
Kass: 1.58e+05, kdiss: 1.34e−03
696_ID.199_H8_SLLQHLIHL
KD: 2.65e−07
Kass: 5.09e+04, kdiss: 1.35e−02
697_ID.1990_Y4_SLLYHLIGL
KD: 1.04e−08
Kass: 1.57e+05, kdiss: 1.64e−03
698_ID.1993_M6_SLLQHMIGL
KD: 7.69e−09
Kass: 1.46e+05, kdiss: 1.12e−03
699_ID.1994_A5_SLLQALIGL
KD: 8.15e−08
Kass: 1.46e+05, kdiss: 1.19e−02
700_ID.1997_D6_SLLQHDIGL
KD: 1.20e−07
Kass: 6.78e+04, kdiss: 8.11e−03
value
Time
0
5000
10000
15000
250
500
750
1000

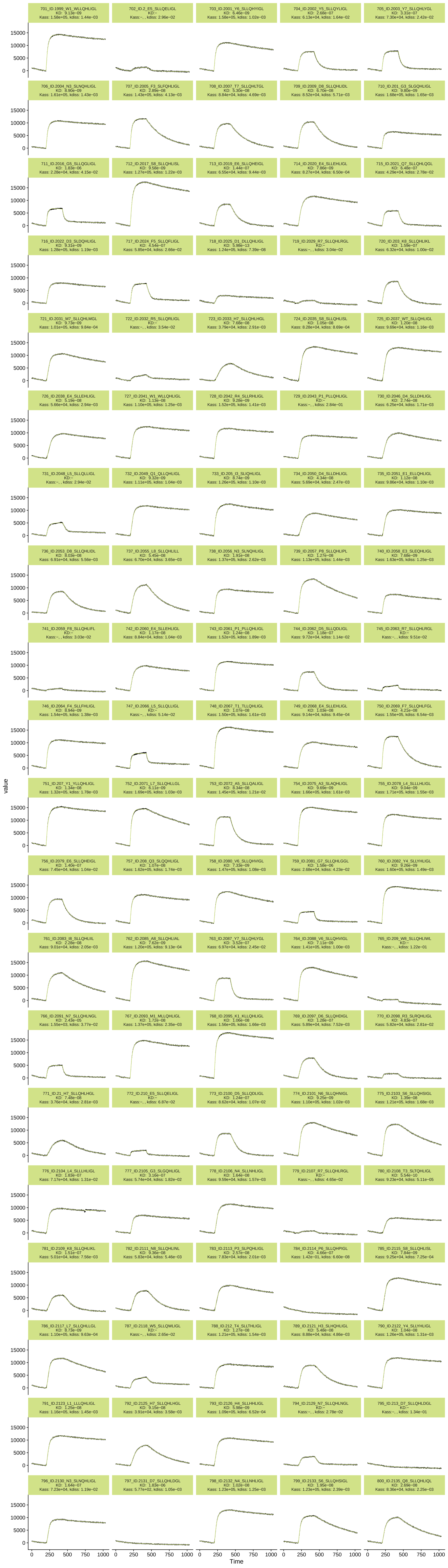
value
Time
0
5000
10000
15000
0
250
500
750
1000

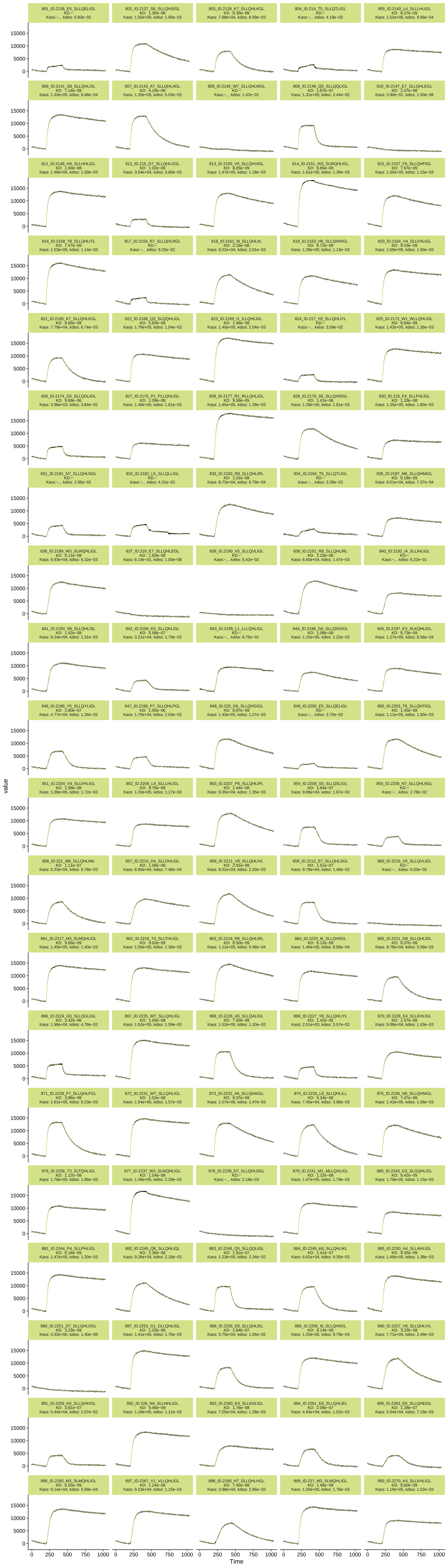
801_ID.2136_E5_SLLQELIGL
KD:−
Kass:−, , kdiss: 5.83e−02
802_ID.2137_S6_SLLQHSIGL
KD: 1.30e−08
Kass: 1.50e+05, kdiss: 1.95e−03
803_ID.2139_K7_SLLQHLKGL
KD: 8.36e−08
Kass: 7.88e+04, kdiss: 6.59e−03
804_ID.214_T5_SLLQTLIGL
KD:−
Kass:−, , kdiss: 4.19e−02
805_ID.2140_L4_SLLLHLIGL
KD: 6.27e−09
Kass: 1.52e+05, kdiss: 9.56e−04
806_ID.2141_S8_SLLQHLISL
KD: 7.14e−09
Kass: 1.33e+05, kdiss: 9.48e−04
807_ID.2143_A7_SLLQHLAGL
KD: 4.10e−08
Kass: 1.35e+05, kdiss: 5.53e−03
808_ID.2145_W7_SLLQHLWGL
KD:−
Kass:−, , kdiss: 1.42e−01
809_ID.2146_Q5_SLLQQLIGL
KD: 1.87e−07
Kass: 1.31e+05, kdiss: 2.44e−02
810_ID.2147_E7_SLLQHLEGL
KD: 2.57e−08
Kass: 3.90e−01, kdiss: 1.00e−08
811_ID.2148_H4_SLLHHLIGL
KD: 1.00e−08
Kass: 1.49e+05, kdiss: 1.50e−03
812_ID.215_G7_SLLQHLGGL
KD: 1.02e−06
Kass: 3.54e+04, kdiss: 3.60e−02
813_ID.2150_V6_SLLQHVIGL
KD: 8.05e−09
Kass: 1.47e+05, kdiss: 1.18e−03
814_ID.2151_W3_SLWQHLIGL
KD: 8.65e−09
Kass: 1.61e+05, kdiss: 1.39e−03
815_ID.2157_F6_SLLQHFIGL
KD: 7.67e−09
Kass: 1.50e+05, kdiss: 1.15e−03
816_ID.2158_T8_SLLQHLITL
KD: 7.47e−09
Kass: 1.53e+05, kdiss: 1.14e−03
817_ID.2159_R7_SLLQHLRGL
KD:−
Kass:−, , kdiss: 9.25e−02
818_ID.2161_I8_SLLQHLIIL
KD: 2.18e−08
Kass: 9.22e+04, kdiss: 2.01e−03
819_ID.2163_H6_SLLQHHIGL
KD: 8.72e−09
Kass: 1.29e+05, kdiss: 1.13e−03
820_ID.2164_V4_SLLVHLIGL
KD: 9.43e−09
Kass: 1.59e+05, kdiss: 1.50e−03
821_ID.2165_K7_SLLQHLKGL
KD: 8.65e−08
Kass: 7.79e+04, kdiss: 6.74e−03
822_ID.2168_Q3_SLQQHLIGL
KD: 5.83e−09
Kass: 1.79e+05, kdiss: 1.04e−03
823_ID.2169_I1_ILLQHLIGL
KD: 1.46e−08
Kass: 1.40e+05, kdiss: 2.04e−03
824_ID.217_Y8_SLLQHLIYL
KD:−
Kass:−, , kdiss: 3.59e−02
825_ID.2173_W1_WLLQHLIGL
KD: 8.84e−09
Kass: 1.42e+05, kdiss: 1.26e−03
826_ID.2174_G5_SLLQGLIGL
KD: 9.68e−06
Kass: 3.96e+03, kdiss: 3.84e−02
827_ID.2175_P1_PLLQHLIGL
KD: 1.09e−08
Kass: 1.48e+05, kdiss: 1.61e−03
828_ID.2177_R1_RLLQHLIGL
KD: 9.58e−09
Kass: 1.45e+05, kdiss: 1.39e−03
829_ID.2179_S6_SLLQHSIGL
KD: 1.41e−08
Kass: 1.28e+05, kdiss: 1.81e−03
830_ID.218_F4_SLLFHLIGL
KD: 1.33e−08
Kass: 1.35e+05, kdiss: 1.80e−03
831_ID.2181_N7_SLLQHLNGL
KD:−
Kass:−, , kdiss: 2.95e−02
832_ID.2182_L5_SLLQLLIGL
KD:−
Kass:−, , kdiss: 4.31e−02
833_ID.2183_R8_SLLQHLIRL
KD: 1.01e−08
Kass: 8.73e+04, kdiss: 8.79e−04
834_ID.2184_T5_SLLQTLIGL
KD:−
Kass:−, , kdiss: 3.39e−02
835_ID.2187_M6_SLLQHMIGL
KD: 8.18e−09
Kass: 9.01e+04, kdiss: 7.37e−04
836_ID.2189_W3_SLWQHLIGL
KD: 9.11e−08
Kass: 6.93e+04, kdiss: 6.32e−03
837_ID.219_E7_SLLQHLEGL
KD: 1.63e−08
Kass: 6.14e−01, kdiss: 1.00e−08
838_ID.2190_V5_SLLQVLIGL
KD:−
Kass:−, , kdiss: 5.42e−02
839_ID.2191_R8_SLLQHLIRL
KD: 2.23e−08
Kass: 6.60e+04, kdiss: 1.47e−03
840_ID.2192_I4_SLLIHLIGL
KD:−
Kass:−, , kdiss: 6.22e−01
841_ID.2193_S8_SLLQHLISL
KD: 1.42e−08
Kass: 9.24e+04, kdiss: 1.31e−03
842_ID.2194_K5_SLLQKLIGL
KD: 5.58e−07
Kass: 3.21e+04, kdiss: 1.79e−02
843_ID.2195_L1_LLLQHLIGL
KD:−
Kass:−, , kdiss: 8.75e−01
844_ID.2196_G6_SLLQHGIGL
KD: 1.06e−08
Kass: 1.15e+05, kdiss: 1.22e−03
845_ID.2197_K3_SLKQHLIGL
KD: 6.73e−09
Kass: 1.27e+05, kdiss: 8.56e−04
846_ID.2198_Y5_SLLQYLIGL
KD: 2.80e−07
Kass: 4.77e+04, kdiss: 1.34e−02
847_ID.2199_P7_SLLQHLPGL
KD: 1.55e−06
Kass: 1.70e+04, kdiss: 2.63e−02
848_ID.220_G6_SLLQHGIGL
KD: 8.87e−09
Kass: 1.43e+05, kdiss: 1.27e−03
849_ID.2200_E5_SLLQELIGL
KD:−
Kass:−, , kdiss: 3.70e−02
850_ID.2203_T6_SLLQHTIGL
KD: 1.45e−08
Kass: 1.11e+05, kdiss: 1.60e−03
851_ID.2204_V4_SLLVHLIGL
KD: 1.58e−08
Kass: 1.09e+05, kdiss: 1.72e−03
852_ID.2206_L4_SLLLHLIGL
KD: 9.76e−09
Kass: 1.20e+05, kdiss: 1.17e−03
853_ID.2207_P8_SLLQHLIPL
KD: 1.44e−08
Kass: 9.35e+04, kdiss: 1.35e−03
854_ID.2208_S5_SLLQSLIGL
KD: 1.84e−07
Kass: 9.08e+04, kdiss: 1.67e−02
855_ID.2209_N7_SLLQHLNGL
KD:−
Kass:−, , kdiss: 2.78e−02
856_ID.221_M8_SLLQHLIML
KD: 1.11e−07
Kass: 5.23e+04, kdiss: 5.78e−03
857_ID.2210_D4_SLLDHLIGL
KD: 1.08e−08
Kass: 6.93e+04, kdiss: 7.48e−04
858_ID.2211_V8_SLLQHLIVL
KD: 2.91e−08
Kass: 8.01e+04, kdiss: 2.33e−03
859_ID.2213_S7_SLLQHLSGL
KD: 1.51e−07
Kass: 9.79e+04, kdiss: 1.48e−02
860_ID.2216_V5_SLLQVLIGL
KD:−
Kass:−, , kdiss: 6.03e−02
861_ID.2217_M3_SLMQHLIGL
KD: 9.66e−09
Kass: 1.45e+05, kdiss: 1.40e−03
862_ID.2218_T4_SLLTHLIGL
KD: 9.02e−09
Kass: 1.50e+05, kdiss: 1.36e−03
863_ID.2219_R8_SLLQHLIRL
KD: 8.50e−09
Kass: 1.11e+05, kdiss: 9.48e−04
864_ID.2220_I6_SLLQHIIGL
KD: 6.12e−09
Kass: 1.40e+05, kdiss: 8.56e−04
865_ID.2221_D8_SLLQHLIDL
KD: 6.37e−08
Kass: 8.76e+04, kdiss: 5.58e−03
866_ID.2224_G5_SLLQGLIGL
KD: 2.42e−06
Kass: 1.98e+04, kdiss: 4.78e−02
867_ID.2225_WT_SLLQHLIGL
KD: 1.05e−08
Kass: 1.52e+05, kdiss: 1.59e−03
868_ID.2226_A5_SLLQALIGL
KD: 7.93e−08
Kass: 1.52e+05, kdiss: 1.20e−02
869_ID.2227_Y8_SLLQHLIYL
KD: 1.42e−05
Kass: 2.51e+03, kdiss: 3.57e−02
870_ID.2228_E4_SLLEHLIGL
KD: 1.57e−08
Kass: 9.09e+04, kdiss: 1.43e−03
871_ID.2229_F7_SLLQHLFGL
KD: 3.86e−08
Kass: 1.61e+05, kdiss: 6.23e−03
872_ID.2231_WT_SLLQHLIGL
KD: 1.02e−08
Kass: 1.54e+05, kdiss: 1.57e−03
873_ID.2232_A6_SLLQHAIGL
KD: 9.37e−09
Kass: 1.57e+05, kdiss: 1.47e−03
874_ID.2233_L8_SLLQHLILL
KD: 5.34e−08
Kass: 7.45e+04, kdiss: 3.98e−03
875_ID.2235_N6_SLLQHNIGL
KD: 7.47e−09
Kass: 1.42e+05, kdiss: 1.06e−03
876_ID.2236_T3_SLTQHLIGL
KD: 1.12e−08
Kass: 1.76e+05, kdiss: 1.96e−03
877_ID.2237_W3_SLWQHLIGL
KD: 1.54e−08
Kass: 1.49e+05, kdiss: 2.29e−03
878_ID.2239_D7_SLLQHLDGL
KD:−
Kass:−, , kdiss: 2.18e−03
879_ID.2241_M1_MLLQHLIGL
KD: 1.22e−08
Kass: 1.47e+05, kdiss: 1.79e−03
880_ID.2243_G3_SLGQHLIGL
KD: 6.42e−09
Kass: 1.79e+05, kdiss: 1.15e−03
881_ID.2244_P4_SLLPHLIGL
KD: 8.18e−09
Kass: 1.47e+05, kdiss: 1.20e−03
882_ID.2245_Q8_SLLQHLIQL
KD: 2.36e−08
Kass: 9.26e+04, kdiss: 2.18e−03
883_ID.2246_Q5_SLLQQLIGL
KD: 1.91e−07
Kass: 1.23e+05, kdiss: 2.34e−02
884_ID.2249_K8_SLLQHLIKL
KD: 1.41e−07
Kass: 6.62e+04, kdiss: 9.35e−03
885_ID.2250_A4_SLLAHLIGL
KD: 9.35e−09
Kass: 1.48e+05, kdiss: 1.38e−03
886_ID.2251_D7_SLLQHLDGL
KD: 3.23e−09
Kass: 4.32e+00, kdiss: 1.40e−08
887_ID.2253_G1_GLLQHLIGL
KD: 1.25e−08
Kass: 1.41e+05, kdiss: 1.76e−03
888_ID.2255_E8_SLLQHLIEL
KD: 1.84e−07
Kass: 5.70e+04, kdiss: 1.05e−02
889_ID.2256_I6_SLLQHIIGL
KD: 8.14e−09
Kass: 1.20e+05, kdiss: 9.79e−04
890_ID.2257_V8_SLLQHLIVL
KD: 3.23e−08
Kass: 7.71e+04, kdiss: 2.49e−03
891_ID.2259_K6_SLLQHKIGL
KD: 3.81e−07
Kass: 5.44e+04, kdiss: 2.07e−02
892_ID.226_N4_SLLNHLIGL
KD: 9.46e−09
Kass: 1.18e+05, kdiss: 1.11e−03
893_ID.2260_E4_SLLEHLIGL
KD: 1.76e−08
Kass: 7.25e+04, kdiss: 1.28e−03
894_ID.2261_E8_SLLQHLIEL
KD: 2.06e−07
Kass: 4.94e+04, kdiss: 1.02e−02
895_ID.2263_E6_SLLQHEIGL
KD: 1.28e−07
Kass: 5.64e+04, kdiss: 7.19e−03
896_ID.2265_M3_SLMQHLIGL
KD: 6.55e−09
Kass: 9.14e+04, kdiss: 5.99e−04
897_ID.2267_V1_VLLQHLIGL
KD: 1.24e−08
Kass: 9.23e+04, kdiss: 1.15e−03
898_ID.2269_H7_SLLQHLHGL
KD: 7.45e−08
Kass: 3.98e+04, kdiss: 2.96e−03
899_ID.227_M3_SLMQHLIGL
KD: 1.48e−08
Kass: 1.20e+05, kdiss: 1.78e−03
900_ID.2270_K4_SLLKHLIGL
KD: 8.60e−09
Kass: 1.19e+05, kdiss: 1.02e−03
value
0
5000
10000
15000
0
250
500
750
1000
Time

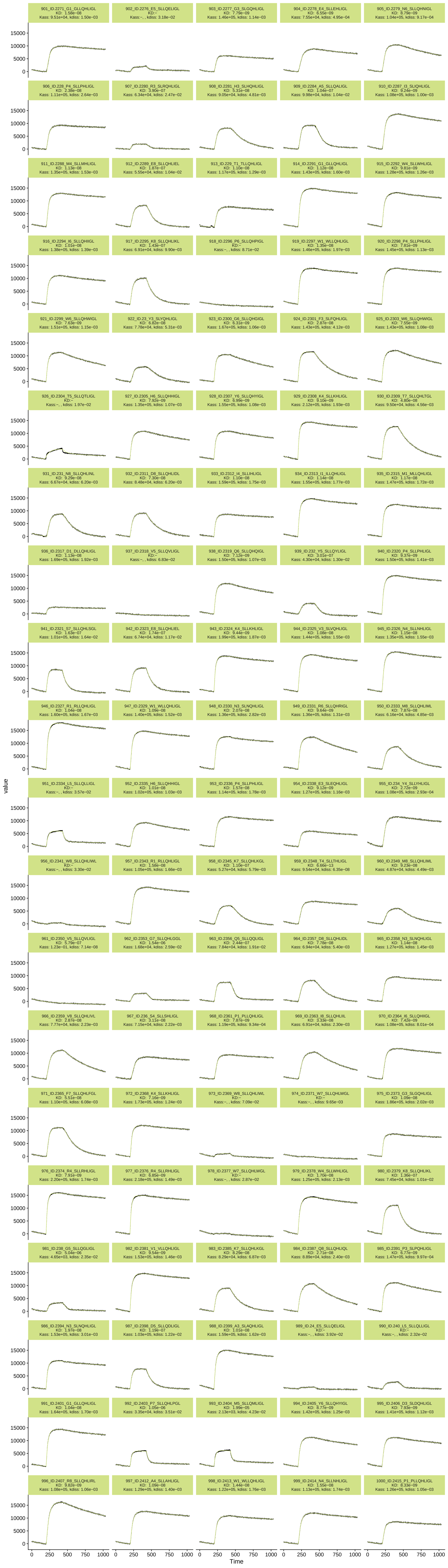
value
Time
0
5000
10000
15000
0
250
500
750
1000

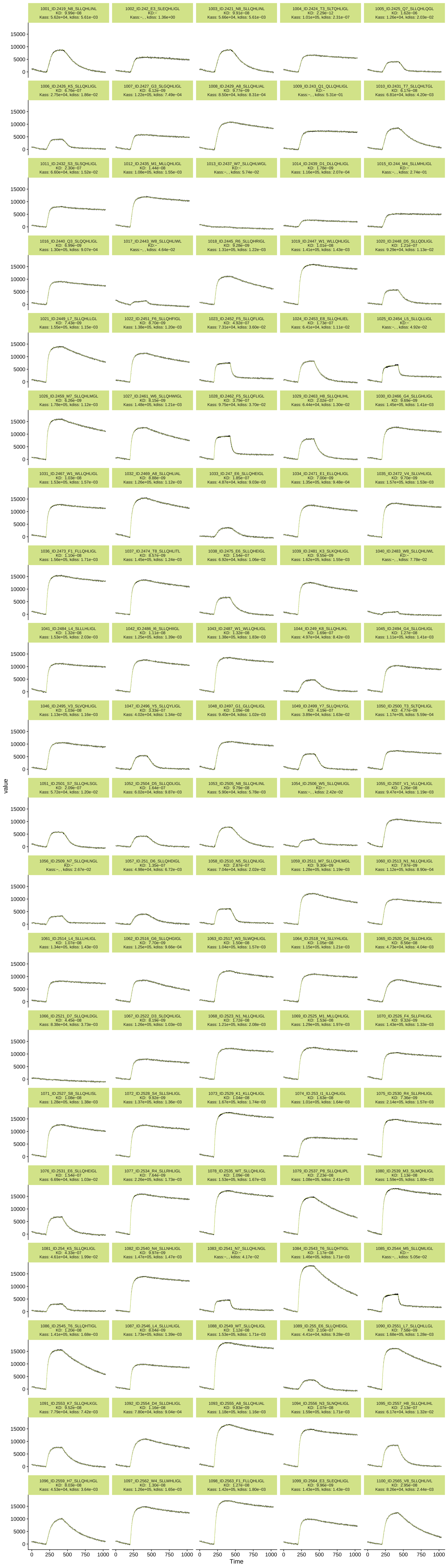
1001_ID.2419_N8_SLLQHLINL KD: 9.99e−08 Kass: 5.62e+04, kdiss: 5.61e−03
1002_ID.242_E3_SLEQHLIGL KD:− Kass:−, , kdiss: 1.36e+00
1003_ID.2421_N8_SLLQHLINL KD: 9.91e−08 Kass: 5.66e+04, kdiss: 5.61e−03
1004_ID.2424_T3_SLTQHLIGL KD: 2.29e−12 Kass: 1.01e+05, kdiss: 2.31e−07
1005_ID.2425_Q7_SLLQHLQGL KD: 1.62e−06 Kass: 1.26e+04, kdiss: 2.03e−02
1006_ID.2426_K5_SLLQKLIGL KD: 6.76e−07 Kass: 2.75e+04, kdiss: 1.86e−02
1007_ID.2427_G3_SLGQHLIGL KD: 6.12e−09 Kass: 1.22e+05, kdiss: 7.49e−04
1008_ID.2429_A8_SLLQHLIAL KD: 9.77e−09 Kass: 8.50e+04, kdiss: 8.31e−04
1009_ID.243_Q1_QLLQHLIGL KD:− Kass:−, , kdiss: 5.31e−01
1010_ID.2431_T7_SLLQHLTGL KD: 6.17e−08 Kass: 6.81e+04, kdiss: 4.20e−03
1011_ID.2432_S3_SLSQHLIGL KD: 2.30e−07 Kass: 6.60e+04, kdiss: 1.52e−02
1012_ID.2435_M1_MLLQHLIGL KD: 1.44e−08 Kass: 1.08e+05, kdiss: 1.55e−03
1013_ID.2437_W7_SLLQHLWGL KD:− Kass:−, , kdiss: 5.74e−02
1014_ID.2439_D1_DLLQHLIGL KD: 1.78e−09 Kass: 1.16e+05, kdiss: 2.07e−04
1015_ID.244_M4_SLLMHLIGL KD:− Kass:−, , kdiss: 2.74e−01
1016_ID.2440_Q3_SLQQHLIGL KD: 6.99e−09 Kass: 1.30e+05, kdiss: 9.07e−04
1017_ID.2443_W8_SLLQHLIWL KD:− Kass:−, , kdiss: 4.64e−02
1018_ID.2445_R6_SLLQHRIGL KD: 9.28e−09 Kass: 1.31e+05, kdiss: 1.22e−03
1019_ID.2447_W1_WLLQHLIGL KD: 1.01e−08 Kass: 1.41e+05, kdiss: 1.43e−03
1020_ID.2448_D5_SLLQDLIGL KD: 1.21e−07 Kass: 9.29e+04, kdiss: 1.13e−02
1021_ID.2449_L7_SLLQHLLGL KD: 7.43e−09 Kass: 1.55e+05, kdiss: 1.15e−03
1022_ID.2451_F6_SLLQHFIGL KD: 8.70e−09 Kass: 1.38e+05, kdiss: 1.20e−03
1023_ID.2452_F5_SLLQFLIGL KD: 4.92e−07 Kass: 7.31e+04, kdiss: 3.60e−02
1024_ID.2453_E8_SLLQHLIEL KD: 1.73e−07 Kass: 6.41e+04, kdiss: 1.11e−02
1025_ID.2454_L5_SLLQLLIGL KD:− Kass:−, , kdiss: 4.92e−02
1026_ID.2459_M7_SLLQHLMGL KD: 6.26e−09 Kass: 1.78e+05, kdiss: 1.12e−03
1027_ID.2461_W6_SLLQHWIGL KD: 8.15e−09 Kass: 1.48e+05, kdiss: 1.21e−03
1028_ID.2462_F5_SLLQFLIGL KD: 3.79e−07 Kass: 9.75e+04, kdiss: 3.70e−02
1029_ID.2463_H8_SLLQHLIHL KD: 2.02e−07 Kass: 6.44e+04, kdiss: 1.30e−02
1030_ID.2466_G4_SLLGHLIGL KD: 9.69e−09 Kass: 1.45e+05, kdiss: 1.41e−03
1031_ID.2467_W1_WLLQHLIGL KD: 1.03e−08 Kass: 1.53e+05, kdiss: 1.57e−03
1032_ID.2469_A8_SLLQHLIAL KD: 8.88e−09 Kass: 1.26e+05, kdiss: 1.12e−03
1033_ID.247_E6_SLLQHEIGL KD: 1.85e−07 Kass: 4.87e+04, kdiss: 9.03e−03
1034_ID.2471_E1_ELLQHLIGL KD: 7.00e−09 Kass: 1.35e+05, kdiss: 9.48e−04
1035_ID.2472_V4_SLLVHLIGL KD: 9.70e−09 Kass: 1.57e+05, kdiss: 1.53e−03
1036_ID.2473_F1_FLLQHLIGL KD: 1.10e−08 Kass: 1.56e+05, kdiss: 1.71e−03
1037_ID.2474_T8_SLLQHLITL KD: 8.57e−09 Kass: 1.45e+05, kdiss: 1.24e−03
1038_ID.2475_E6_SLLQHEIGL KD: 1.54e−07 Kass: 6.92e+04, kdiss: 1.06e−02
1039_ID.2481_K3_SLKQHLIGL KD: 9.55e−09 Kass: 1.62e+05, kdiss: 1.55e−03
1040_ID.2483_W8_SLLQHLIWL KD:− Kass:−, , kdiss: 7.78e−02
1041_ID.2484_L4_SLLLHLIGL KD: 1.32e−08 Kass: 1.53e+05, kdiss: 2.03e−03
1042_ID.2486_I6_SLLQHIIGL KD: 1.11e−08 Kass: 1.25e+05, kdiss: 1.39e−03
1043_ID.2487_W1_WLLQHLIGL KD: 1.32e−08 Kass: 1.38e+05, kdiss: 1.83e−03
1044_ID.249_K8_SLLQHLIKL KD: 1.69e−07 Kass: 4.97e+04, kdiss: 8.42e−03
1045_ID.2494_G4_SLLGHLIGL KD: 1.27e−08 Kass: 1.11e+05, kdiss: 1.41e−03
1046_ID.2495_V3_SLVQHLIGL KD: 1.03e−08 Kass: 1.13e+05, kdiss: 1.16e−03
1047_ID.2496_Y5_SLLQYLIGL KD: 3.33e−07 Kass: 4.02e+04, kdiss: 1.34e−02
1048_ID.2497_G1_GLLQHLIGL KD: 1.09e−08 Kass: 9.40e+04, kdiss: 1.02e−03
1049_ID.2499_Y7_SLLQHLYGL KD: 4.19e−07 Kass: 3.89e+04, kdiss: 1.63e−02
1050_ID.2500_T3_SLTQHLIGL KD: 4.77e−09 Kass: 1.17e+05, kdiss: 5.59e−04
1051_ID.2501_S7_SLLQHLSGL KD: 2.09e−07 Kass: 5.72e+04, kdiss: 1.20e−02
1052_ID.2504_D5_SLLQDLIGL KD: 1.64e−07 Kass: 6.02e+04, kdiss: 9.87e−03
1053_ID.2505_N8_SLLQHLINL KD: 9.79e−08 Kass: 5.90e+04, kdiss: 5.78e−03
1054_ID.2506_W5_SLLQWLIGL KD:− Kass:−, , kdiss: 2.42e−02
1055_ID.2507_V1_VLLQHLIGL KD: 1.26e−08 Kass: 9.47e+04, kdiss: 1.19e−03
1056_ID.2509_N7_SLLQHLNGL KD:− Kass:−, , kdiss: 2.67e−02
1057_ID.251_D6_SLLQHDIGL KD: 1.35e−07 Kass: 4.98e+04, kdiss: 6.72e−03
1058_ID.2510_N5_SLLQNLIGL KD: 2.87e−07 Kass: 7.04e+04, kdiss: 2.02e−02
1059_ID.2511_M7_SLLQHLMGL KD: 9.30e−09 Kass: 1.28e+05, kdiss: 1.19e−03
1060_ID.2513_N1_NLLQHLIGL KD: 7.97e−09 Kass: 1.12e+05, kdiss: 8.90e−04
1061_ID.2514_L4_SLLLHLIGL KD: 1.07e−08 Kass: 1.34e+05, kdiss: 1.43e−03
1062_ID.2516_G6_SLLQHGIGL KD: 7.70e−09 Kass: 1.25e+05, kdiss: 9.66e−04
1063_ID.2517_W3_SLWQHLIGL KD: 1.50e−08 Kass: 1.04e+05, kdiss: 1.57e−03
1064_ID.2518_Y4_SLLYHLIGL KD: 1.05e−08 Kass: 1.15e+05, kdiss: 1.21e−03
1065_ID.2520_D4_SLLDHLIGL KD: 8.56e−08 Kass: 4.73e+04, kdiss: 4.04e−03
1066_ID.2521_D7_SLLQHLDGL KD: 4.45e−08 Kass: 8.38e+04, kdiss: 3.73e−03
1067_ID.2522_D3_SLDQHLIGL KD: 8.19e−09 Kass: 1.26e+05, kdiss: 1.03e−03
1068_ID.2523_N1_NLLQHLIGL KD: 1.72e−08 Kass: 1.21e+05, kdiss: 2.08e−03
1069_ID.2525_M1_MLLQHLIGL KD: 1.53e−08 Kass: 1.29e+05, kdiss: 1.97e−03
1070_ID.2526_F4_SLLFHLIGL KD: 9.32e−09 Kass: 1.43e+05, kdiss: 1.33e−03
1071_ID.2527_S8_SLLQHLISL KD: 1.08e−08 Kass: 1.28e+05, kdiss: 1.38e−03
1072_ID.2528_S4_SLLSHLIGL KD: 9.92e−09 Kass: 1.37e+05, kdiss: 1.36e−03
1073_ID.2529_K1_KLLQHLIGL KD: 1.04e−08 Kass: 1.67e+05, kdiss: 1.74e−03
1074_ID.253_I1_ILLQHLIGL KD: 1.63e−08 Kass: 1.01e+05, kdiss: 1.64e−03
1075_ID.2530_R4_SLLRHLIGL KD: 7.36e−09 Kass: 2.14e+05, kdiss: 1.57e−03
1076_ID.2531_E6_SLLQHEIGL KD: 1.54e−07 Kass: 6.69e+04, kdiss: 1.03e−02
1077_ID.2534_R4_SLLRHLIGL KD: 7.64e−09 Kass: 2.26e+05, kdiss: 1.73e−03
1078_ID.2535_WT_SLLQHLIGL KD: 1.09e−08 Kass: 1.53e+05, kdiss: 1.67e−03
1079_ID.2537_P8_SLLQHLIPL KD: 2.23e−08 Kass: 1.08e+05, kdiss: 2.41e−03
1080_ID.2539_M3_SLMQHLIGL KD: 1.13e−08 Kass: 1.59e+05, kdiss: 1.80e−03
1081_ID.254_K5_SLLQKLIGL KD: 4.33e−07 Kass: 4.61e+04, kdiss: 1.99e−02
1082_ID.2540_N4_SLLNHLIGL KD: 9.97e−09 Kass: 1.47e+05, kdiss: 1.47e−03
1083_ID.2541_N7_SLLQHLNGL KD:− Kass:−, , kdiss: 4.17e−02
1084_ID.2543_T6_SLLQHTIGL KD: 1.17e−08 Kass: 1.46e+05, kdiss: 1.71e−03
1085_ID.2544_M5_SLLQMLIGL KD:− Kass:−, , kdiss: 5.05e−02
1086_ID.2545_T6_SLLQHTIGL KD: 1.20e−08 Kass: 1.41e+05, kdiss: 1.68e−03
1087_ID.2546_L4_SLLLHLIGL KD: 8.04e−09 Kass: 1.73e+05, kdiss: 1.39e−03
1088_ID.2549_WT_SLLQHLIGL KD: 1.12e−08 Kass: 1.53e+05, kdiss: 1.71e−03
1089_ID.255_E6_SLLQHEIGL KD: 2.10e−07 Kass: 4.41e+04, kdiss: 9.28e−03
1090_ID.2551_L7_SLLQHLLGL KD: 7.58e−09 Kass: 1.68e+05, kdiss: 1.28e−03
1091_ID.2553_K7_SLLQHLKGL KD: 9.52e−08 Kass: 7.79e+04, kdiss: 7.42e−03
1092_ID.2554_D4_SLLDHLIGL KD: 1.16e−08 Kass: 7.80e+04, kdiss: 9.04e−04
1093_ID.2555_A8_SLLQHLIAL KD: 9.83e−09 Kass: 1.18e+05, kdiss: 1.16e−03
1094_ID.2556_N3_SLNQHLIGL KD: 1.07e−08 Kass: 1.59e+05, kdiss: 1.71e−03
1095_ID.2557_H8_SLLQHLIHL KD: 2.13e−07 Kass: 6.17e+04, kdiss: 1.32e−02
1096_ID.2559_H7_SLLQHLHGL KD: 8.03e−08 Kass: 4.53e+04, kdiss: 3.64e−03
1097_ID.2562_W4_SLLWHLIGL KD: 1.30e−08 Kass: 1.26e+05, kdiss: 1.65e−03
1098_ID.2563_F1_FLLQHLIGL KD: 1.27e−08 Kass: 1.42e+05, kdiss: 1.80e−03
1099_ID.2564_E3_SLEQHLIGL KD: 9.96e−09 Kass: 1.43e+05, kdiss: 1.43e−03
1100_ID.2565_V8_SLLQHLIVL KD: 2.95e−08 Kass: 8.26e+04, kdiss: 2.44e−03
value
0
5000
10000
15000
Time
0
250
500
750
1000

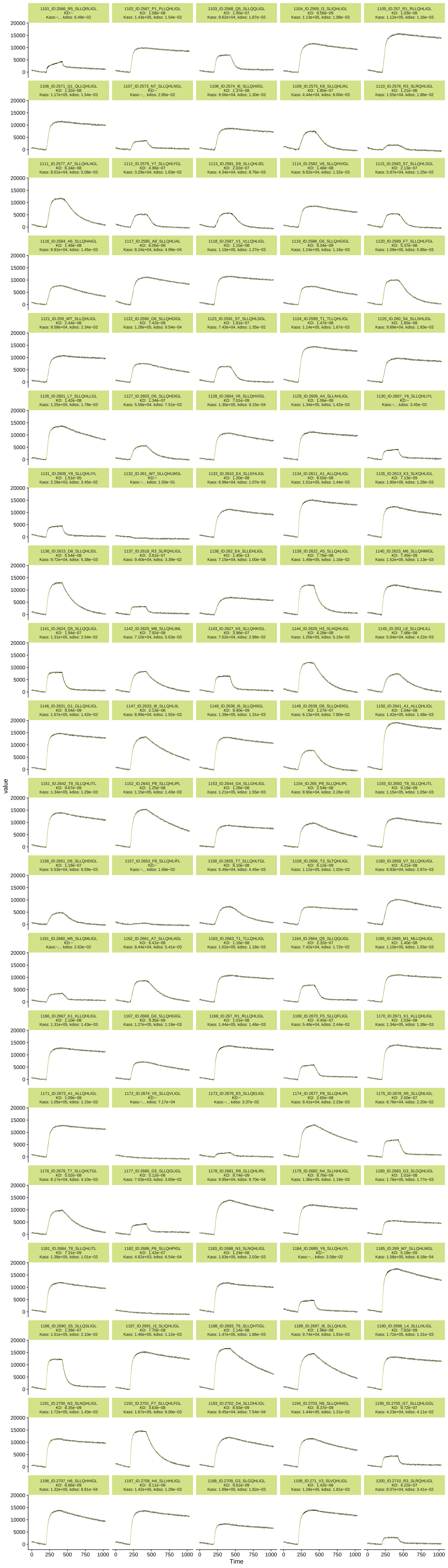

1101_ID.2566_R5_SLLQRLIGL KD:− Kass:−, , kdiss: 6.48e−02
1102_ID.2567_P1_PLLQHLIGL KD: 1.08e−08 Kass: 1.43e+05, kdiss: 1.54e−03
1103_ID.2568_Q5_SLLQQLIGL KD: 1.95e−07 Kass: 9.62e+04, kdiss: 1.87e−02
1104_ID.2569_I3_SLIQHLIGL KD: 9.56e−09 Kass: 1.13e+05, kdiss: 1.08e−03
1105_ID.257_R1_RLLQHLIGL KD: 1.03e−08 Kass: 1.12e+05, kdiss: 1.16e−03
1106_ID.2571_Q1_QLLQHLIGL KD: 1.32e−08 Kass: 1.17e+05, kdiss: 1.54e−03
1107_ID.2573_N7_SLLQHLNGL KD:− Kass:−, , kdiss: 2.85e−02
1108_ID.2574_I6_SLLQHIIGL KD: 1.37e−08 Kass: 9.56e+04, kdiss: 1.30e−03
1109_ID.2575_K8_SLLQHLIKL KD: 1.80e−07 Kass: 4.44e+04, kdiss: 8.00e−03
1110_ID.2576_R3_SLRQHLIGL KD: 1.21e−06 Kass: 1.55e+04, kdiss: 1.88e−02
1111_ID.2577_A7_SLLQHLAGL KD: 6.34e−08 Kass: 8.01e+04, kdiss: 5.08e−03
1112_ID.2579_Y7_SLLQHLYGL KD: 4.96e−07 Kass: 3.29e+04, kdiss: 1.63e−02
1113_ID.2581_E8_SLLQHLIEL KD: 2.02e−07 Kass: 4.34e+04, kdiss: 8.76e−03
1114_ID.2582_V6_SLLQHVIGL KD: 1.48e−08 Kass: 8.92e+04, kdiss: 1.32e−03
1115_ID.2583_S7_SLLQHLSGL KD: 2.13e−07 Kass: 5.87e+04, kdiss: 1.25e−02
1116_ID.2584_A6_SLLQHAIGL KD: 1.46e−08 Kass: 9.91e+04, kdiss: 1.45e−03
1117_ID.2585_A8_SLLQHLIAL KD: 6.05e−09 Kass: 8.24e+04, kdiss: 4.99e−04
1118_ID.2587_V1_VLLQHLIGL KD: 1.15e−08 Kass: 1.10e+05, kdiss: 1.27e−03
1119_ID.2588_G6_SLLQHGIGL KD: 9.34e−09 Kass: 1.24e+05, kdiss: 1.16e−03
1120_ID.2589_F7_SLLQHLFGL KD: 5.37e−08 Kass: 1.09e+05, kdiss: 5.86e−03
1121_ID.259_WT_SLLQHLIGL KD: 2.44e−08 Kass: 9.58e+04, kdiss: 2.34e−03
1122_ID.2590_G6_SLLQHGIGL KD: 7.42e−09 Kass: 1.28e+05, kdiss: 9.54e−04
1123_ID.2591_S7_SLLQHLSGL KD: 1.81e−07 Kass: 7.43e+04, kdiss: 1.35e−02
1124_ID.2599_T1_TLLQHLIGL KD: 1.47e−08 Kass: 1.14e+05, kdiss: 1.67e−03
1125_ID.260_S4_SLLSHLIGL KD: 1.85e−08 Kass: 9.89e+04, kdiss: 1.83e−03
1126_ID.2601_L7_SLLQHLLGL KD: 1.42e−08 Kass: 1.25e+05, kdiss: 1.78e−03
1127_ID.2603_D6_SLLQHDIGL KD: 1.34e−07 Kass: 5.59e+04, kdiss: 7.51e−03
1128_ID.2604_V6_SLLQHVIGL KD: 7.01e−09 Kass: 1.30e+05, kdiss: 9.15e−04
1129_ID.2606_A4_SLLAHLIGL KD: 1.06e−08 Kass: 1.34e+05, kdiss: 1.42e−03
1130_ID.2607_Y8_SLLQHLIYL KD:− Kass:−, , kdiss: 3.45e−02
1131_ID.2609_Y8_SLLQHLIYL KD: 1.51e−05 Kass: 2.28e+03, kdiss: 3.45e−02
1132_ID.261_W7_SLLQHLWGL KD:− Kass:−, , kdiss: 1.50e−01
1133_ID.2610_E4_SLLEHLIGL KD: 1.20e−08 Kass: 8.96e+04, kdiss: 1.07e−03
1134_ID.2611_A1_ALLQHLIGL KD: 9.55e−09 Kass: 1.51e+05, kdiss: 1.44e−03
1135_ID.2613_K3_SLKQHLIGL KD: 7.13e−09 Kass: 1.80e+05, kdiss: 1.28e−03
1136_ID.2615_D8_SLLQHLIDL KD: 5.54e−08 Kass: 9.72e+04, kdiss: 5.38e−03
1137_ID.2618_R3_SLRQHLIGL KD: 3.61e−07 Kass: 9.40e+04, kdiss: 3.39e−02
1138_ID.262_E4_SLLEHLIGL KD: 1.40e−13 Kass: 7.15e+04, kdiss: 1.00e−08
1139_ID.2622_A5_SLLQALIGL KD: 7.76e−08 Kass: 1.49e+05, kdiss: 1.16e−02
1140_ID.2623_M6_SLLQHMIGL KD: 7.45e−09 Kass: 1.52e+05, kdiss: 1.13e−03
1141_ID.2624_Q5_SLLQQLIGL KD: 1.94e−07 Kass: 1.31e+05, kdiss: 2.54e−02
1142_ID.2625_M8_SLLQHLIML KD: 7.92e−08 Kass: 7.10e+04, kdiss: 5.63e−03
1143_ID.2627_K6_SLLQHKIGL KD: 3.96e−07 Kass: 7.52e+04, kdiss: 2.98e−02
1144_ID.2629_H3_SLHQHLIGL KD: 4.28e−08 Kass: 1.20e+05, kdiss: 5.15e−03
1145_ID.263_L8_SLLQHLILL KD: 7.48e−08 Kass: 5.64e+04, kdiss: 4.22e−03
1146_ID.2631_G1_GLLQHLIGL KD: 9.04e−09 Kass: 1.57e+05, kdiss: 1.42e−03
1147_ID.2633_I8_SLLQHLIIL KD: 2.13e−08 Kass: 8.99e+04, kdiss: 1.92e−03
1148_ID.2636_I6_SLLQHIIGL KD: 9.40e−09 Kass: 1.39e+05, kdiss: 1.31e−03
1149_ID.2639_D6_SLLQHDIGL KD: 1.27e−07 Kass: 6.13e+04, kdiss: 7.80e−03
1150_ID.2641_A1_ALLQHLIGL KD: 1.04e−08 Kass: 1.42e+05, kdiss: 1.48e−03
1151_ID.2642_T8_SLLQHLITL KD: 9.67e−09 Kass: 1.34e+05, kdiss: 1.29e−03
1152_ID.2643_P8_SLLQHLIPL KD: 1.25e−08 Kass: 1.15e+05, kdiss: 1.43e−03
1153_ID.2644_G4_SLLGHLIGL KD: 1.28e−08 Kass: 1.21e+05, kdiss: 1.55e−03
1154_ID.265_P8_SLLQHLIPL KD: 2.54e−08 Kass: 8.90e+04, kdiss: 2.26e−03
1155_ID.2650_T8_SLLQHLITL KD: 9.19e−09 Kass: 1.15e+05, kdiss: 1.05e−03
1156_ID.2651_D6_SLLQHDIGL KD: 1.19e−07 Kass: 5.53e+04, kdiss: 6.59e−03
1157_ID.2653_F8_SLLQHLIFL KD:− Kass:−, , kdiss: 1.68e−02
1158_ID.2655_T7_SLLQHLTGL KD: 8.10e−08 Kass: 5.49e+04, kdiss: 4.45e−03
1159_ID.2656_T3_SLTQHLIGL KD: 9.12e−09 Kass: 1.12e+05, kdiss: 1.02e−03
1160_ID.2659_V7_SLLQHLVGL KD: 4.21e−08 Kass: 6.83e+04, kdiss: 2.87e−03
1161_ID.2660_M5_SLLQMLIGL KD:− Kass:−, , kdiss: 2.63e−02
1162_ID.2661_A7_SLLQHLAGL KD: 6.41e−08 Kass: 8.44e+04, kdiss: 5.41e−03
1163_ID.2663_T1_TLLQHLIGL KD: 1.16e−08 Kass: 1.02e+05, kdiss: 1.18e−03
1164_ID.2664_Q5_SLLQQLIGL KD: 2.32e−07 Kass: 7.42e+04, kdiss: 1.72e−02
1165_ID.2665_M1_MLLQHLIGL KD: 1.40e−08 Kass: 1.10e+05, kdiss: 1.55e−03
1166_ID.2667_K1_KLLQHLIGL KD: 1.10e−08 Kass: 1.31e+05, kdiss: 1.43e−03
1167_ID.2668_G6_SLLQHGIGL KD: 9.35e−09 Kass: 1.27e+05, kdiss: 1.19e−03
1168_ID.267_R1_RLLQHLIGL KD: 1.01e−08 Kass: 1.44e+05, kdiss: 1.46e−03
1169_ID.2670_F5_SLLQFLIGL KD: 4.45e−07 Kass: 5.48e+04, kdiss: 2.44e−02
1170_ID.2671_K1_KLLQHLIGL KD: 1.03e−08 Kass: 1.34e+05, kdiss: 1.38e−03
1171_ID.2673_A1_ALLQHLIGL KD: 1.09e−08 Kass: 1.05e+05, kdiss: 1.15e−03
1172_ID.2674_V5_SLLQVLIGL KD:− Kass:−, , kdiss: 7.17e−04
1173_ID.2676_E5_SLLQELIGL KD:− Kass:−, , kdiss: 3.37e−02
1174_ID.2677_P8_SLLQHLIPL KD: 2.65e−08 Kass: 8.41e+04, kdiss: 2.23e−03
1175_ID.2678_N5_SLLQNLIGL KD: 2.50e−07 Kass: 8.78e+04, kdiss: 2.20e−02
1176_ID.2679_T7_SLLQHLTGL KD: 5.02e−08 Kass: 8.17e+04, kdiss: 4.10e−03
1177_ID.2680_G5_SLLQGLIGL KD: 5.12e−06 Kass: 7.03e+03, kdiss: 3.60e−02
1178_ID.2681_R8_SLLQHLIRL KD: 9.74e−09 Kass: 9.95e+04, kdiss: 9.70e−04
1179_ID.2682_N4_SLLNHLIGL KD: 8.76e−09 Kass: 1.36e+05, kdiss: 1.19e−03
1180_ID.2683_G3_SLGQHLIGL KD: 1.01e−08 Kass: 1.76e+05, kdiss: 1.77e−03
1181_ID.2684_T8_SLLQHLITL KD: 7.31e−09 Kass: 1.38e+05, kdiss: 1.01e−03
1182_ID.2686_P6_SLLQHPIGL KD: 1.42e−07 Kass: 4.62e+03, kdiss: 6.54e−04
1183_ID.2688_N3_SLNQHLIGL KD: 1.24e−08 Kass: 1.63e+05, kdiss: 2.03e−03
1184_ID.2689_Y8_SLLQHLIYL KD:− Kass:−, , kdiss: 3.58e−02
1185_ID.269_M7_SLLQHLMGL KD: 5.18e−09 Kass: 1.58e+05, kdiss: 8.18e−04
1186_ID.2690_S5_SLLQSLIGL KD: 1.39e−07 Kass: 1.51e+05, kdiss: 2.10e−02
1187_ID.2691_I3_SLIQHLIGL KD: 7.70e−09 Kass: 1.46e+05, kdiss: 1.12e−03
1188_ID.2693_T6_SLLQHTIGL KD: 1.14e−08 Kass: 1.47e+05, kdiss: 1.66e−03
1189_ID.2697_I8_SLLQHLIIL KD: 1.96e−08 Kass: 9.74e+04, kdiss: 1.91e−03
1190_ID.2698_L4_SLLLHLIGL KD: 7.62e−09 Kass: 1.72e+05, kdiss: 1.31e−03
1191_ID.2700_N3_SLNQHLIGL KD: 8.35e−09 Kass: 1.72e+05, kdiss: 1.43e−03
1192_ID.2701_F7_SLLQHLFGL KD: 3.63e−08 Kass: 1.67e+05, kdiss: 6.06e−03
1193_ID.2702_D4_SLLDHLIGL KD: 8.93e−09 Kass: 8.45e+04, kdiss: 7.54e−04
1194_ID.2703_N6_SLLQHNIGL KD: 8.37e−09 Kass: 1.44e+05, kdiss: 1.21e−03
1195_ID.2705_G7_SLLQHLGGL KD: 9.72e−07 Kass: 4.23e+04, kdiss: 4.11e−02
1196_ID.2707_H6_SLLQHHIGL KD: 6.66e−09 Kass: 1.32e+05, kdiss: 8.81e−04
1197_ID.2708_H4_SLLHHLIGL KD: 9.11e−09 Kass: 1.42e+05, kdiss: 1.29e−03
1198_ID.2709_G3_SLGQHLIGL KD: 9.61e−09 Kass: 1.89e+05, kdiss: 1.82e−03
1199_ID.271_V3_SLVQHLIGL KD: 1.42e−08 Kass: 1.28e+05, kdiss: 1.81e−03
1200_ID.2710_R3_SLRQHLIGL KD: 4.22e−07 Kass: 8.07e+04, kdiss: 3.41e−02
value
0
5000
10000
15000
20000
Time
0
250
500
750
1000

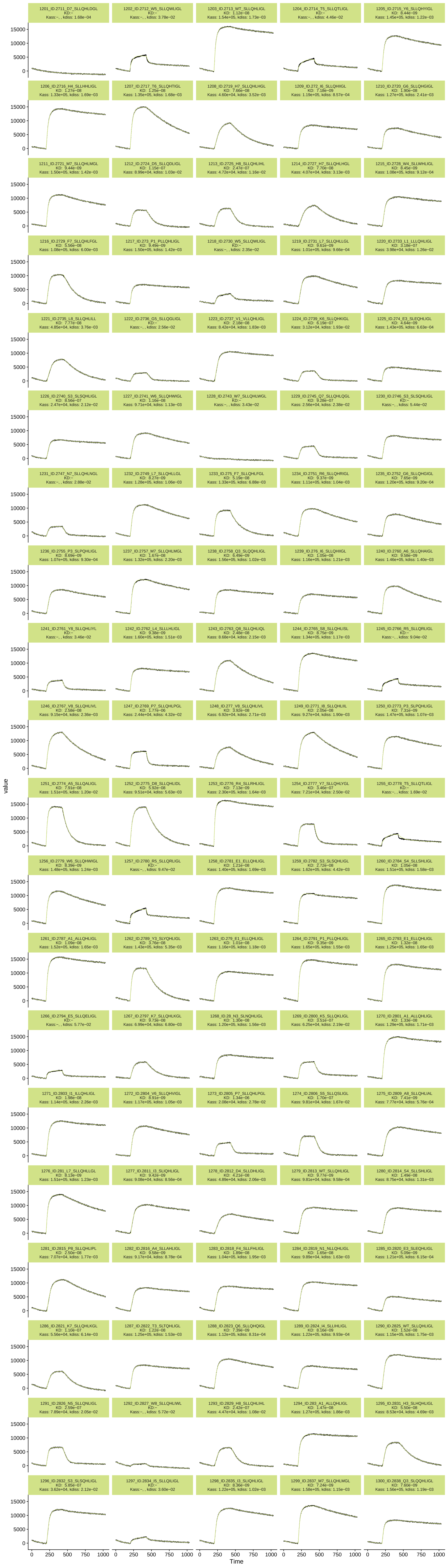
value
Time

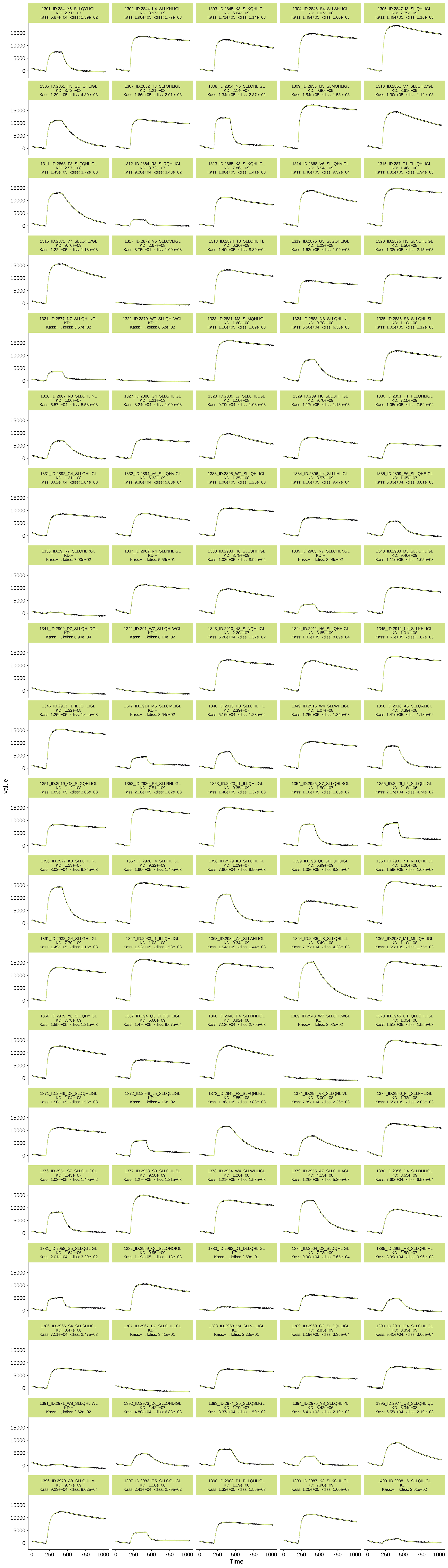
1301_ID.284_Y5_SLLQYLIGL KD: 2.71e−07 Kass: 5.87e+04, kdiss: 1.59e−02
1302_ID.2844_K4_SLLKHLIGL KD: 8.97e−09 Kass: 1.98e+05, kdiss: 1.77e−03
1303_ID.2845_K3_SLKQHLIGL KD: 6.64e−09 Kass: 1.71e+05, kdiss: 1.14e−03
1304_ID.2846_S4_SLLSHLIGL KD: 1.07e−08 Kass: 1.49e+05, kdiss: 1.60e−03
1305_ID.2847_I3_SLIQHLIGL KD: 7.75e−09 Kass: 1.49e+05, kdiss: 1.16e−03
1306_ID.2851_H3_SLHQHLIGL KD: 3.72e−08 Kass: 1.29e+05, kdiss: 4.80e−03
1307_ID.2852_T3_SLTQHLIGL KD: 1.21e−08 Kass: 1.66e+05, kdiss: 2.01e−03
1308_ID.2854_N5_SLLQNLIGL KD: 2.14e−07 Kass: 1.34e+05, kdiss: 2.87e−02
1309_ID.2855_M3_SLMQHLIGL KD: 9.96e−09 Kass: 1.54e+05, kdiss: 1.53e−03
1310_ID.2861_V7_SLLQHLVGL KD: 8.61e−09 Kass: 1.30e+05, kdiss: 1.12e−03
1311_ID.2863_F3_SLFQHLIGL KD: 2.57e−08 Kass: 1.45e+05, kdiss: 3.72e−03
1312_ID.2864_R3_SLRQHLIGL KD: 3.73e−07 Kass: 9.20e+04, kdiss: 3.43e−02
1313_ID.2865_K3_SLKQHLIGL KD: 7.86e−09 Kass: 1.80e+05, kdiss: 1.41e−03
1314_ID.2868_V6_SLLQHVIGL KD: 6.54e−09 Kass: 1.46e+05, kdiss: 9.52e−04
1315_ID.287_T1_TLLQHLIGL KD: 1.46e−08 Kass: 1.32e+05, kdiss: 1.94e−03
1316_ID.2871_V7_SLLQHLVGL KD: 9.70e−09 Kass: 1.22e+05, kdiss: 1.18e−03
1317_ID.2872_V5_SLLQVLIGL KD: 2.67e−08 Kass: 3.75e−01, kdiss: 1.00e−08
1318_ID.2874_T8_SLLQHLITL KD: 6.36e−09 Kass: 1.40e+05, kdiss: 8.89e−04
1319_ID.2875_G3_SLGQHLIGL KD: 1.23e−08 Kass: 1.62e+05, kdiss: 1.99e−03
1320_ID.2876_N3_SLNQHLIGL KD: 1.56e−08 Kass: 1.38e+05, kdiss: 2.15e−03
1321_ID.2877_N7_SLLQHLNGL KD:− Kass:−, , kdiss: 3.57e−02
1322_ID.2879_W7_SLLQHLWGL KD:− Kass:−, , kdiss: 6.62e−02
1323_ID.2881_M3_SLMQHLIGL KD: 1.60e−08 Kass: 1.18e+05, kdiss: 1.89e−03
1324_ID.2883_N8_SLLQHLINL KD: 9.78e−08 Kass: 6.50e+04, kdiss: 6.36e−03
1325_ID.2885_S8_SLLQHLISL KD: 1.10e−08 Kass: 1.02e+05, kdiss: 1.12e−03
1326_ID.2887_N8_SLLQHLINL KD: 1.00e−07 Kass: 5.57e+04, kdiss: 5.58e−03
1327_ID.2888_G4_SLLGHLIGL KD: 1.21e−13 Kass: 8.24e+04, kdiss: 1.00e−08
1328_ID.2889_L7_SLLQHLLGL KD: 1.10e−08 Kass: 9.79e+04, kdiss: 1.08e−03
1329_ID.289_H6_SLLQHHIGL KD: 9.70e−09 Kass: 1.17e+05, kdiss: 1.13e−03
1330_ID.2891_P1_PLLQHLIGL KD: 7.15e−09 Kass: 1.05e+05, kdiss: 7.54e−04
1331_ID.2892_G4_SLLGHLIGL KD: 1.21e−08 Kass: 8.62e+04, kdiss: 1.04e−03
1332_ID.2894_V6_SLLQHVIGL KD: 6.33e−09 Kass: 9.30e+04, kdiss: 5.88e−04
1333_ID.2895_WT_SLLQHLIGL KD: 1.25e−08 Kass: 1.00e+05, kdiss: 1.25e−03
1334_ID.2896_L4_SLLLHLIGL KD: 8.57e−09 Kass: 1.10e+05, kdiss: 9.47e−04
1335_ID.2899_E6_SLLQHEIGL KD: 1.65e−07 Kass: 5.33e+04, kdiss: 8.81e−03
1336_ID.29_R7_SLLQHLRGL KD:− Kass:−, , kdiss: 7.90e−02
1337_ID.2902_N4_SLLNHLIGL KD:− Kass:−, , kdiss: 5.59e−01
1338_ID.2903_H6_SLLQHHIGL KD: 8.78e−09 Kass: 1.02e+05, kdiss: 8.92e−04
1339_ID.2905_N7_SLLQHLNGL KD:− Kass:−, , kdiss: 3.06e−02
1340_ID.2908_D3_SLDQHLIGL KD: 9.46e−09 Kass: 1.11e+05, kdiss: 1.05e−03
1341_ID.2909_D7_SLLQHLDGL KD:− Kass:−, , kdiss: 6.90e−04
1342_ID.291_W7_SLLQHLWGL KD:− Kass:−, , kdiss: 8.10e−02
1343_ID.2910_N3_SLNQHLIGL KD: 2.20e−07 Kass: 6.20e+04, kdiss: 1.37e−02
1344_ID.2911_H6_SLLQHHIGL KD: 8.65e−09 Kass: 1.01e+05, kdiss: 8.69e−04
1345_ID.2912_K4_SLLKHLIGL KD: 1.01e−08 Kass: 1.61e+05, kdiss: 1.62e−03
1346_ID.2913_I1_ILLQHLIGL KD: 1.32e−08 Kass: 1.25e+05, kdiss: 1.64e−03
1347_ID.2914_M5_SLLQMLIGL KD:− Kass:−, , kdiss: 3.64e−02
1348_ID.2915_H8_SLLQHLIHL KD: 2.39e−07 Kass: 5.16e+04, kdiss: 1.23e−02
1349_ID.2916_W4_SLLWHLIGL KD: 1.07e−08 Kass: 1.25e+05, kdiss: 1.34e−03
1350_ID.2918_A5_SLLQALIGL KD: 8.39e−08 Kass: 1.41e+05, kdiss: 1.18e−02
1351_ID.2919_G3_SLGQHLIGL KD: 1.12e−08 Kass: 1.85e+05, kdiss: 2.06e−03
1352_ID.2920_R4_SLLRHLIGL KD: 7.51e−09 Kass: 2.16e+05, kdiss: 1.62e−03
1353_ID.2923_I1_ILLQHLIGL KD: 9.35e−09 Kass: 1.46e+05, kdiss: 1.37e−03
1354_ID.2925_S7_SLLQHLSGL KD: 1.50e−07 Kass: 1.10e+05, kdiss: 1.65e−02
1355_ID.2926_L5_SLLQLLIGL KD: 2.18e−06 Kass: 2.17e+04, kdiss: 4.74e−02
1356_ID.2927_K8_SLLQHLIKL KD: 1.23e−07 Kass: 8.02e+04, kdiss: 9.84e−03
1357_ID.2928_I4_SLLIHLIGL KD: 9.32e−09 Kass: 1.60e+05, kdiss: 1.49e−03
1358_ID.2929_K8_SLLQHLIKL KD: 1.29e−07 Kass: 7.66e+04, kdiss: 9.90e−03
1359_ID.293_Q6_SLLQHQIGL KD: 5.99e−09 Kass: 1.38e+05, kdiss: 8.25e−04
1360_ID.2931_N1_NLLQHLIGL KD: 1.06e−08 Kass: 1.59e+05, kdiss: 1.68e−03
1361_ID.2932_G4_SLLGHLIGL KD: 7.70e−09 Kass: 1.49e+05, kdiss: 1.15e−03
1362_ID.2933_I1_ILLQHLIGL KD: 1.03e−08 Kass: 1.52e+05, kdiss: 1.58e−03
1363_ID.2934_A4_SLLAHLIGL KD: 9.34e−09 Kass: 1.54e+05, kdiss: 1.44e−03
1364_ID.2935_L8_SLLQHLILL KD: 5.49e−08 Kass: 7.79e+04, kdiss: 4.28e−03
1365_ID.2937_M1_MLLQHLIGL KD: 1.10e−08 Kass: 1.59e+05, kdiss: 1.75e−03
1366_ID.2939_Y6_SLLQHYIGL KD: 7.78e−09 Kass: 1.55e+05, kdiss: 1.21e−03
1367_ID.294_Q3_SLQQHLIGL KD: 6.60e−09 Kass: 1.47e+05, kdiss: 9.67e−04
1368_ID.2940_D4_SLLDHLIGL KD: 3.92e−08 Kass: 7.12e+04, kdiss: 2.79e−03
1369_ID.2943_W7_SLLQHLWGL KD:− Kass:−, , kdiss: 2.02e−02
1370_ID.2945_Q1_QLLQHLIGL KD: 1.03e−08 Kass: 1.51e+05, kdiss: 1.55e−03
1371_ID.2946_D3_SLDQHLIGL KD: 1.04e−08 Kass: 1.50e+05, kdiss: 1.55e−03
1372_ID.2948_L5_SLLQLLIGL KD:− Kass:−, , kdiss: 4.15e−02
1373_ID.2949_F3_SLFQHLIGL KD: 2.85e−08 Kass: 1.36e+05, kdiss: 3.88e−03
1374_ID.295_V8_SLLQHLIVL KD: 3.00e−08 Kass: 7.85e+04, kdiss: 2.36e−03
1375_ID.2950_F4_SLLFHLIGL KD: 1.32e−08 Kass: 1.55e+05, kdiss: 2.05e−03
1376_ID.2951_S7_SLLQHLSGL KD: 1.45e−07 Kass: 1.03e+05, kdiss: 1.49e−02
1377_ID.2953_S8_SLLQHLISL KD: 9.58e−09 Kass: 1.27e+05, kdiss: 1.21e−03
1378_ID.2954_W4_SLLWHLIGL KD: 1.26e−08 Kass: 1.21e+05, kdiss: 1.53e−03
1379_ID.2955_A7_SLLQHLAGL KD: 4.13e−08 Kass: 1.26e+05, kdiss: 5.20e−03
1380_ID.2956_D4_SLLDHLIGL KD: 8.65e−09 Kass: 7.60e+04, kdiss: 6.57e−04
1381_ID.2958_G5_SLLQGLIGL KD: 1.64e−06 Kass: 2.01e+04, kdiss: 3.29e−02
1382_ID.2959_Q6_SLLQHQIGL KD: 9.95e−09 Kass: 1.19e+05, kdiss: 1.18e−03
1383_ID.2963_D1_DLLQHLIGL KD:− Kass:−, , kdiss: 2.58e−01
1384_ID.2964_D3_SLDQHLIGL KD: 7.73e−09 Kass: 9.90e+04, kdiss: 7.65e−04
1385_ID.2965_H8_SLLQHLIHL KD: 2.50e−07 Kass: 3.99e+04, kdiss: 9.96e−03
1386_ID.2966_S4_SLLSHLIGL KD: 3.47e−08 Kass: 7.11e+04, kdiss: 2.47e−03
1387_ID.2967_E7_SLLQHLEGL KD:− Kass:−, , kdiss: 3.41e−01
1388_ID.2968_V4_SLLVHLIGL KD:− Kass:−, , kdiss: 2.23e−01
1389_ID.2969_G3_SLGQHLIGL KD: 2.83e−09 Kass: 1.19e+05, kdiss: 3.36e−04
1390_ID.2970_G4_SLLGHLIGL KD: 3.89e−09 Kass: 9.41e+04, kdiss: 3.66e−04
1391_ID.2971_W8_SLLQHLIWL KD:− Kass:−, , kdiss: 2.62e−02
1392_ID.2973_D6_SLLQHDIGL KD: 1.42e−07 Kass: 4.80e+04, kdiss: 6.83e−03
1393_ID.2974_S5_SLLQSLIGL KD: 1.79e−07 Kass: 8.37e+04, kdiss: 1.50e−02
1394_ID.2975_Y8_SLLQHLIYL KD: 3.42e−06 Kass: 6.41e+03, kdiss: 2.19e−02
1395_ID.2977_Q8_SLLQHLIQL KD: 3.34e−08 Kass: 6.55e+04, kdiss: 2.19e−03
1396_ID.2979_A8_SLLQHLIAL KD: 9.77e−09 Kass: 9.23e+04, kdiss: 9.02e−04
1397_ID.2982_G5_SLLQGLIGL KD: 1.16e−06 Kass: 2.41e+04, kdiss: 2.79e−02
1398_ID.2983_P1_PLLQHLIGL KD: 1.19e−08 Kass: 1.32e+05, kdiss: 1.56e−03
1399_ID.2987_K3_SLKQHLIGL KD: 7.98e−09 Kass: 1.25e+05, kdiss: 1.00e−03
1400_ID.2988_I5_SLLQILIGL KD:− Kass:−, , kdiss: 2.61e−02
value
0
5000
10000
15000
Time
0 250 500 750 1000

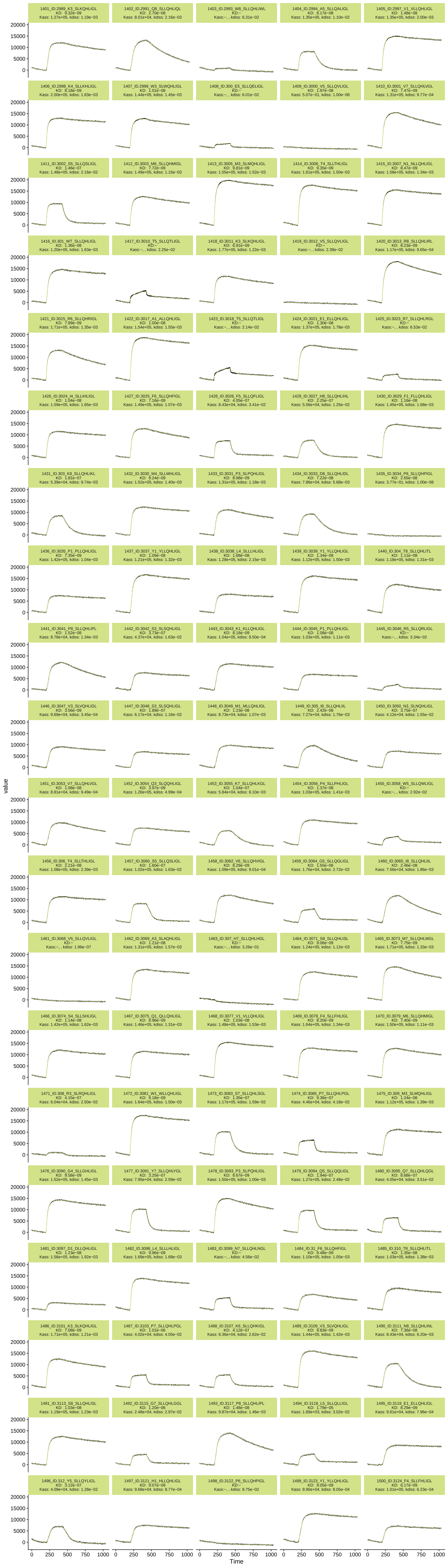
1401_ID.2989_K3_SLKQHLIGL
KD: 9.32e−09
Kass: 1.27e+05, kdiss: 1.19e−03
1402_ID.2991_Q8_SLLQHLIQL
KD: 2.70e−08
Kass: 8.01e+04, kdiss: 2.16e−03
1403_ID.2993_W8_SLLQHLIWL
KD:−
Kass:−, , kdiss: 6.31e−02
1404_ID.2994_A5_SLLQALIGL
KD: 8.17e−08
Kass: 1.35e+05, kdiss: 1.10e−02
1405_ID.2997_V1_VLLQHLIGL
KD: 1.48e−08
Kass: 1.35e+05, kdiss: 2.00e−03
1406_ID.2998_K4_SLLKHLIGL
KD: 8.18e−09
Kass: 2.00e+05, kdiss: 1.63e−03
1407_ID.2999_W3_SLWQHLIGL
KD: 1.01e−08
Kass: 1.44e+05, kdiss: 1.45e−03
1408_ID.300_E5_SLLQELIGL
KD:−
Kass:−, , kdiss: 6.01e−02
1409_ID.3000_V5_SLLQVLIGL
KD: 1.97e−08
Kass: 5.07e−01, kdiss: 1.00e−08
1410_ID.3001_V7_SLLQHLVGL
KD: 7.47e−09
Kass: 1.31e+05, kdiss: 9.77e−04
1411_ID.3002_S5_SLLQSLIGL
KD: 1.46e−07
Kass: 1.48e+05, kdiss: 2.16e−02
1412_ID.3003_M6_SLLQHMIGL
KD: 7.72e−09
Kass: 1.49e+05, kdiss: 1.15e−03
1413_ID.3005_M3_SLMQHLIGL
KD: 9.81e−09
Kass: 1.55e+05, kdiss: 1.52e−03
1414_ID.3006_T4_SLLTHLIGL
KD: 9.35e−09
Kass: 1.61e+05, kdiss: 1.50e−03
1415_ID.3007_N1_NLLQHLIGL
KD: 8.47e−09
Kass: 1.58e+05, kdiss: 1.34e−03
1416_ID.301_WT_SLLQHLIGL
KD: 1.36e−08
Kass: 1.20e+05, kdiss: 1.63e−03
1417_ID.3010_T5_SLLQTLIGL
KD:−
Kass:−, , kdiss: 2.25e−02
1418_ID.3011_K3_SLKQHLIGL
KD: 6.91e−09
Kass: 1.77e+05, kdiss: 1.22e−03
1419_ID.3012_V5_SLLQVLIGL
KD:−
Kass:−, , kdiss: 2.39e−02
1420_ID.3013_R8_SLLQHLIRL
KD: 8.23e−09
Kass: 1.17e+05, kdiss: 9.65e−04
1421_ID.3015_R6_SLLQHRIGL
KD: 7.89e−09
Kass: 1.71e+05, kdiss: 1.35e−03
1422_ID.3017_A1_ALLQHLIGL
KD: 1.00e−08
Kass: 1.54e+05, kdiss: 1.55e−03
1423_ID.3018_T5_SLLQTLIGL
KD:−
Kass:−, , kdiss: 2.14e−02
1424_ID.3021_E1_ELLQHLIGL
KD: 1.30e−08
Kass: 1.37e+05, kdiss: 1.78e−03
1425_ID.3023_R7_SLLQHLRGL
KD:−
Kass:−, , kdiss: 6.53e−02
1426_ID.3024_I4_SLLIHLIGL
KD: 1.04e−08
Kass: 1.59e+05, kdiss: 1.65e−03
1427_ID.3025_F6_SLLQHFIGL
KD: 7.16e−09
Kass: 1.49e+05, kdiss: 1.07e−03
1428_ID.3026_F5_SLLQFLIGL
KD: 4.05e−07
Kass: 8.43e+04, kdiss: 3.41e−02
1429_ID.3027_H8_SLLQHLIHL
KD: 2.25e−07
Kass: 5.56e+04, kdiss: 1.25e−02
1430_ID.3029_F1_FLLQHLIGL
KD: 1.16e−08
Kass: 1.45e+05, kdiss: 1.68e−03
1431_ID.303_K8_SLLQHLIKL
KD: 1.81e−07
Kass: 5.39e+04, kdiss: 9.74e−03
1432_ID.3030_M4_SLLMHLIGL
KD: 9.24e−09
Kass: 1.52e+05, kdiss: 1.40e−03
1433_ID.3031_P3_SLPQHLIGL
KD: 8.98e−09
Kass: 1.31e+05, kdiss: 1.18e−03
1434_ID.3033_D8_SLLQHLIDL
KD: 7.22e−08
Kass: 7.86e+04, kdiss: 5.68e−03
1435_ID.3034_P6_SLLQHPIGL
KD: 2.65e−08
Kass: 3.77e−01, kdiss: 1.00e−08
1436_ID.3035_P1_PLLQHLIGL
KD: 7.35e−09
Kass: 1.42e+05, kdiss: 1.04e−03
1437_ID.3037_Y1_YLLQHLIGL
KD: 1.09e−08
Kass: 1.21e+05, kdiss: 1.32e−03
1438_ID.3038_L4_SLLLHLIGL
KD: 1.68e−08
Kass: 1.28e+05, kdiss: 2.15e−03
1439_ID.3039_Y1_YLLQHLIGL
KD: 1.34e−08
Kass: 1.12e+05, kdiss: 1.50e−03
1440_ID.304_T8_SLLQHLITL
KD: 1.11e−08
Kass: 1.18e+05, kdiss: 1.31e−03
1441_ID.3041_P8_SLLQHLIPL
KD: 1.52e−08
Kass: 8.78e+04, kdiss: 1.34e−03
1442_ID.3042_S3_SLSQHLIGL
KD: 3.73e−07
Kass: 4.37e+04, kdiss: 1.63e−02
1443_ID.3043_K1_KLLQHLIGL
KD: 8.18e−09
Kass: 1.04e+05, kdiss: 8.50e−04
1444_ID.3045_P1_PLLQHLIGL
KD: 1.08e−08
Kass: 1.03e+05, kdiss: 1.11e−03
1445_ID.3046_R5_SLLQRLIGL
KD:−
Kass:−, , kdiss: 3.34e−02
1446_ID.3047_V3_SLVQHLIGL
KD: 3.56e−09
Kass: 9.69e+04, kdiss: 3.45e−04
1447_ID.3048_S3_SLSQHLIGL
KD: 1.89e−07
Kass: 6.17e+04, kdiss: 1.16e−02
1448_ID.3049_M1_MLLQHLIGL
KD: 1.23e−08
Kass: 8.73e+04, kdiss: 1.07e−03
1449_ID.305_I8_SLLQHLIIL
KD: 2.42e−08
Kass: 7.27e+04, kdiss: 1.76e−03
1450_ID.3050_N3_SLNQHLIGL
KD: 3.75e−07
Kass: 4.12e+04, kdiss: 1.55e−02
1451_ID.3053_V7_SLLQHLVGL
KD: 1.08e−08
Kass: 8.81e+04, kdiss: 9.49e−04
1452_ID.3054_Q3_SLQQHLIGL
KD: 3.97e−09
Kass: 1.26e+05, kdiss: 4.99e−04
1453_ID.3055_K7_SLLQHLKGL
KD: 1.04e−07
Kass: 5.84e+04, kdiss: 6.10e−03
1454_ID.3056_P4_SLLPHLIGL
KD: 1.37e−08
Kass: 1.03e+05, kdiss: 1.41e−03
1455_ID.3058_W5_SLLQWLIGL
KD:−
Kass:−, , kdiss: 2.92e−02
1456_ID.306_T4_SLLTHLIGL
KD: 2.21e−08
Kass: 1.08e+05, kdiss: 2.39e−03
1457_ID.3060_S5_SLLQSLIGL
KD: 1.60e−07
Kass: 1.02e+05, kdiss: 1.63e−02
1458_ID.3062_V6_SLLQHVIGL
KD: 8.29e−09
Kass: 1.09e+05, kdiss: 9.01e−04
1459_ID.3064_G5_SLLQGLIGL
KD: 1.55e−06
Kass: 1.76e+04, kdiss: 2.72e−02
1460_ID.3065_I8_SLLQHLIIL
KD: 2.46e−08
Kass: 7.56e+04, kdiss: 1.86e−03
1461_ID.3068_V5_SLLQVLIGL
KD:−
Kass:−, , kdiss: 1.96e−07
1462_ID.3069_A3_SLAQHLIGL
KD: 1.21e−08
Kass: 1.31e+05, kdiss: 1.57e−03
1463_ID.307_H7_SLLQHLHGL
KD:−
Kass:−, , kdiss: 3.26e−01
1464_ID.3071_S8_SLLQHLISL
KD: 9.06e−09
Kass: 1.24e+05, kdiss: 1.12e−03
1465_ID.3073_M7_SLLQHLMGL
KD: 7.75e−09
Kass: 1.71e+05, kdiss: 1.33e−03
1466_ID.3074_S4_SLLSHLIGL
KD: 1.14e−08
Kass: 1.42e+05, kdiss: 1.62e−03
1467_ID.3075_Q1_QLLQHLIGL
KD: 8.96e−09
Kass: 1.46e+05, kdiss: 1.31e−03
1468_ID.3077_V1_VLLQHLIGL
KD: 1.03e−08
Kass: 1.48e+05, kdiss: 1.53e−03
1469_ID.3078_F4_SLLFHLIGL
KD: 8.20e−09
Kass: 1.64e+05, kdiss: 1.34e−03
1470_ID.3079_M6_SLLQHMIGL
KD: 7.40e−09
Kass: 1.50e+05, kdiss: 1.11e−03
1471_ID.308_R3_SLRQHLIGL
KD: 4.15e−07
Kass: 6.04e+04, kdiss: 2.50e−02
1472_ID.3081_W1_WLLQHLIGL
KD: 9.18e−09
Kass: 1.64e+05, kdiss: 1.50e−03
1473_ID.3083_S7_SLLQHLSGL
KD: 1.35e−07
Kass: 1.17e+05, kdiss: 1.59e−02
1474_ID.3085_P7_SLLQHLPGL
KD: 9.36e−07
Kass: 4.46e+04, kdiss: 4.18e−02
1475_ID.309_M3_SLMQHLIGL
KD: 1.24e−08
Kass: 1.12e+05, kdiss: 1.39e−03
1476_ID.3090_G4_SLLGHLIGL
KD: 9.58e−09
Kass: 1.52e+05, kdiss: 1.45e−03
1477_ID.3091_Y7_SLLQHLYGL
KD: 3.25e−07
Kass: 7.95e+04, kdiss: 2.59e−02
1478_ID.3093_P3_SLPQHLIGL
KD: 6.67e−09
Kass: 1.50e+05, kdiss: 1.00e−03
1479_ID.3094_Q5_SLLQQLIGL
KD: 1.94e−07
Kass: 1.27e+05, kdiss: 2.48e−02
1480_ID.3095_Q7_SLLQHLQGL
KD: 8.68e−07
Kass: 4.05e+04, kdiss: 3.51e−02
1481_ID.3097_D1_DLLQHLIGL
KD: 1.23e−08
Kass: 1.56e+05, kdiss: 1.92e−03
1482_ID.3098_L4_SLLLHLIGL
KD: 9.96e−09
Kass: 1.69e+05, kdiss: 1.68e−03
1483_ID.3099_N7_SLLQHLNGL
KD:−
Kass:−, , kdiss: 4.56e−02
1484_ID.31_F6_SLLQHFIGL
KD: 9.48e−09
Kass: 1.10e+05, kdiss: 1.05e−03
1485_ID.310_T8_SLLQHLITL
KD: 1.35e−08
Kass: 1.03e+05, kdiss: 1.38e−03
1486_ID.3101_K3_SLKQHLIGL
KD: 7.08e−09
Kass: 1.71e+05, kdiss: 1.21e−03
1487_ID.3103_P7_SLLQHLPGL
KD: 1.01e−06
Kass: 4.02e+04, kdiss: 4.05e−02
1488_ID.3107_K6_SLLQHKIGL
KD: 4.12e−07
Kass: 6.36e+04, kdiss: 2.62e−02
1489_ID.3109_V3_SLVQHLIGL
KD: 9.83e−09
Kass: 1.44e+05, kdiss: 1.42e−03
1490_ID.3111_N8_SLLQHLINL
KD: 7.36e−08
Kass: 8.43e+04, kdiss: 6.20e−03
1491_ID.3113_S8_SLLQHLISL
KD: 1.03e−08
Kass: 1.19e+05, kdiss: 1.23e−03
1492_ID.3115_G7_SLLQHLGGL
KD: 1.20e−06
Kass: 2.48e+04, kdiss: 2.97e−02
1493_ID.3117_P8_SLLQHLIPL
KD: 1.48e−08
Kass: 9.87e+04, kdiss: 1.46e−03
1494_ID.3118_L5_SLLQLLIGL
KD: 1.79e−05
Kass: 1.69e+03, kdiss: 3.02e−02
1495_ID.3119_E1_ELLQHLIGL
KD: 8.29e−09
Kass: 9.61e+04, kdiss: 7.96e−04
1496_ID.312_Y5_SLLQYLIGL
KD: 3.12e−07
Kass: 4.09e+04, kdiss: 1.28e−02
1497_ID.3121_H1_HLLQHLIGL
KD: 9.07e−09
Kass: 9.68e+04, kdiss: 8.77e−04
1498_ID.3122_P6_SLLQHPIGL
KD:−
Kass:−, , kdiss: 9.75e−02
1499_ID.3123_Y1_YLLQHLIGL
KD: 9.05e−09
Kass: 8.90e+04, kdiss: 8.05e−04
1500_ID.3124_F4_SLLFHLIGL
KD: 6.17e−09
Kass: 1.01e+05, kdiss: 6.23e−04
value
Time
0
250
500
750
1000
0
5000
10000
15000
20000

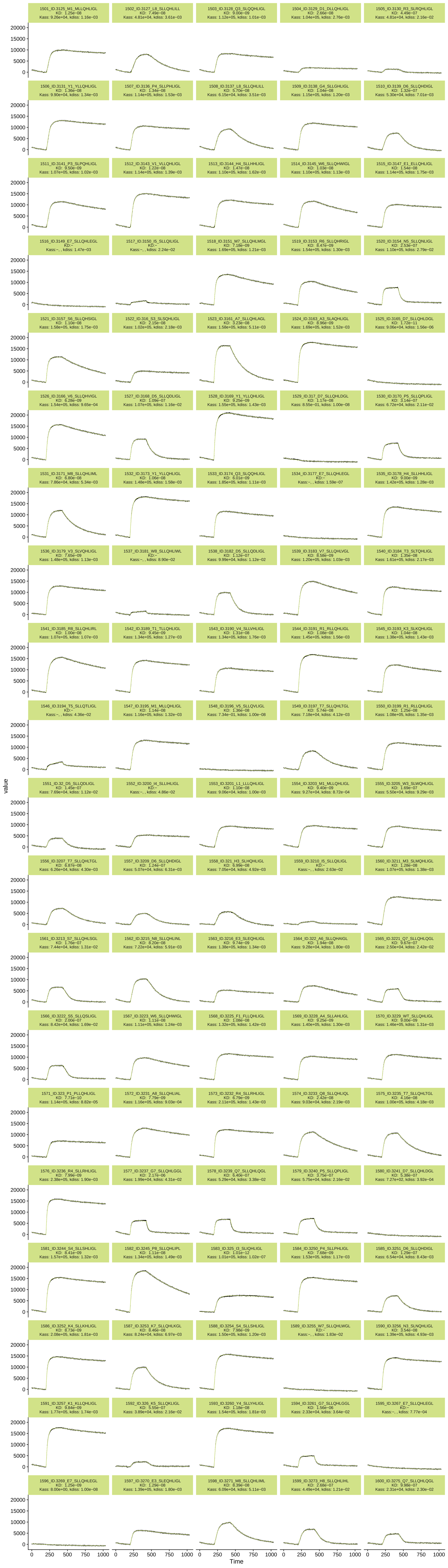

1501_ID.3125_M1_MLLQHLIGL KD: 1.25e−08 Kass: 9.26e+04, kdiss: 1.16e−03
1502_ID.3127_L8_SLLQHLILL KD: 7.49e−08 Kass: 4.81e+04, kdiss: 3.61e−03
1503_ID.3128_Q3_SLQQHLIGL KD: 9.00e−09 Kass: 1.12e+05, kdiss: 1.01e−03
1504_ID.3129_D1_DLLQHLIGL KD: 2.66e−08 Kass: 1.04e+05, kdiss: 2.76e−03
1505_ID.3130_R3_SLRQHLIGL KD: 4.49e−07 Kass: 4.81e+04, kdiss: 2.16e−02
1506_ID.3131_Y1_YLLQHLIGL KD: 1.36e−08 Kass: 9.90e+04, kdiss: 1.34e−03
1507_ID.3136_P4_SLLPHLIGL KD: 1.34e−08 Kass: 1.14e+05, kdiss: 1.53e−03
1508_ID.3137_L8_SLLQHLILL KD: 5.70e−08 Kass: 6.15e+04, kdiss: 3.51e−03
1509_ID.3138_G4_SLLGHLIGL KD: 1.04e−08 Kass: 1.15e+05, kdiss: 1.20e−03
1510_ID.3139_D6_SLLQHDIGL KD: 1.32e−07 Kass: 5.30e+04, kdiss: 7.01e−03
1511_ID.3141_P3_SLPQHLIGL KD: 9.50e−09 Kass: 1.07e+05, kdiss: 1.02e−03
1512_ID.3143_V1_VLLQHLIGL KD: 1.22e−08 Kass: 1.14e+05, kdiss: 1.39e−03
1513_ID.3144_H4_SLLHHLIGL KD: 1.47e−08 Kass: 1.10e+05, kdiss: 1.62e−03
1514_ID.3145_W8_SLLQHWIGL KD: 1.03e−08 Kass: 1.10e+05, kdiss: 1.13e−03
1515_ID.3147_E1_ELLQHLIGL KD: 1.54e−08 Kass: 1.14e+05, kdiss: 1.75e−03
1516_ID.3149_E7_SLLQHLEGL KD:− Kass:−, , kdiss: 1.47e−03
1517_ID.3150_I5_SLLQILIGL KD:− Kass:−, , kdiss: 2.24e−02
1518_ID.3151_M7_SLLQHLMGL KD: 7.18e−09 Kass: 1.69e+05, kdiss: 1.21e−03
1519_ID.3153_R6_SLLQHRIGL KD: 8.44e−09 Kass: 1.54e+05, kdiss: 1.30e−03
1520_ID.3154_N5_SLLQNLIGL KD: 2.53e−07 Kass: 1.10e+05, kdiss: 2.79e−02
1521_ID.3157_S6_SLLQHSIGL KD: 1.10e−08 Kass: 1.58e+05, kdiss: 1.75e−03
1522_ID.316_S3_SLSQHLIGL KD: 2.15e−08 Kass: 1.02e+05, kdiss: 2.18e−03
1523_ID.3161_A7_SLLQHLAGL KD: 3.23e−08 Kass: 1.58e+05, kdiss: 5.11e−03
1524_ID.3163_A3_SLAQHLIGL KD: 8.96e−09 Kass: 1.69e+05, kdiss: 1.52e−03
1525_ID.3165_D7_SLLQHLDGL KD: 1.72e−11 Kass: 9.06e+04, kdiss: 1.56e−06
1526_ID.3166_V6_SLLQHVIGL KD: 6.28e−09 Kass: 1.54e+05, kdiss: 9.65e−04
1527_ID.3168_D5_SLLQDLIGL KD: 1.09e−07 Kass: 1.07e+05, kdiss: 1.16e−02
1528_ID.3169_Y1_YLLQHLIGL KD: 9.25e−09 Kass: 1.55e+05, kdiss: 1.43e−03
1529_ID.317_D7_SLLQHLDGL KD: 1.17e−08 Kass: 8.55e−01, kdiss: 1.00e−08
1530_ID.3170_P5_SLLQPLIGL KD: 3.14e−07 Kass: 6.72e+04, kdiss: 2.11e−02
1531_ID.3171_M8_SLLQHLIML KD: 6.80e−08 Kass: 7.86e+04, kdiss: 5.34e−03
1532_ID.3173_Y1_YLLQHLIGL KD: 1.06e−08 Kass: 1.48e+05, kdiss: 1.58e−03
1533_ID.3174_Q3_SLQQHLIGL KD: 6.01e−09 Kass: 1.85e+05, kdiss: 1.11e−03
1534_ID.3177_E7_SLLQHLEGL KD:− Kass:−, , kdiss: 1.59e−07
1535_ID.3178_H4_SLLHHLIGL KD: 9.00e−09 Kass: 1.42e+05, kdiss: 1.28e−03
1536_ID.3179_V3_SLVQHLIGL KD: 7.65e−09 Kass: 1.48e+05, kdiss: 1.13e−03
1537_ID.3181_W8_SLLQHLIWL KD:− Kass:−, , kdiss: 8.90e−02
1538_ID.3182_D5_SLLQDLIGL KD: 1.12e−07 Kass: 9.99e+04, kdiss: 1.12e−02
1539_ID.3183_V7_SLLQHLVGL KD: 8.58e−09 Kass: 1.20e+05, kdiss: 1.03e−03
1540_ID.3184_T3_SLTQHLIGL KD: 1.35e−08 Kass: 1.61e+05, kdiss: 2.17e−03
1541_ID.3185_R8_SLLQHLIRL KD: 1.00e−08 Kass: 1.07e+05, kdiss: 1.07e−03
1542_ID.3189_T1_TLLQHLIGL KD: 9.45e−09 Kass: 1.34e+05, kdiss: 1.27e−03
1543_ID.3190_V4_SLLVHLIGL KD: 1.31e−08 Kass: 1.34e+05, kdiss: 1.76e−03
1544_ID.3191_R1_RLLQHLIGL KD: 1.08e−08 Kass: 1.45e+05, kdiss: 1.56e−03
1545_ID.3193_K3_SLKQHLIGL KD: 1.04e−08 Kass: 1.39e+05, kdiss: 1.43e−03
1546_ID.3194_T5_SLLQTLIGL KD:− Kass:−, , kdiss: 4.36e−02
1547_ID.3195_M1_MLLQHLIGL KD: 1.14e−08 Kass: 1.16e+05, kdiss: 1.32e−03
1548_ID.3196_V5_SLLQVLIGL KD: 1.36e−08 Kass: 7.34e−01, kdiss: 1.00e−08
1549_ID.3197_T7_SLLQHLTGL KD: 5.74e−08 Kass: 7.18e+04, kdiss: 4.12e−03
1550_ID.3199_R1_RLLQHLIGL KD: 1.25e−08 Kass: 1.08e+05, kdiss: 1.35e−03
1551_ID.32_D5_SLLQDLIGL KD: 1.45e−07 Kass: 7.69e+04, kdiss: 1.12e−02
1552_ID.3200_I4_SLLIHLIGL KD:− Kass:−, , kdiss: 4.86e−02
1553_ID.3201_L1_LLLQHLIGL KD: 1.10e−08 Kass: 9.06e+04, kdiss: 1.00e−03
1554_ID.3203_M1_MLLQHLIGL KD: 9.40e−09 Kass: 9.27e+04, kdiss: 8.72e−04
1555_ID.3205_W3_SLWQHLIGL KD: 1.69e−07 Kass: 5.50e+04, kdiss: 9.29e−03
1556_ID.3207_T7_SLLQHLTGL KD: 6.87e−08 Kass: 6.26e+04, kdiss: 4.30e−03
1557_ID.3209_D6_SLLQHDIGL KD: 1.24e−07 Kass: 5.07e+04, kdiss: 6.31e−03
1558_ID.321_H3_SLHQHLIGL KD: 6.99e−08 Kass: 7.05e+04, kdiss: 4.92e−03
1559_ID.3210_I5_SLLQILIGL KD:− Kass:−, , kdiss: 2.63e−02
1560_ID.3211_M3_SLMQHLIGL KD: 1.28e−08 Kass: 1.07e+05, kdiss: 1.38e−03
1561_ID.3213_S7_SLLQHLSGL KD: 1.76e−07 Kass: 7.44e+04, kdiss: 1.31e−02
1562_ID.3215_N8_SLLQHLINL KD: 8.20e−08 Kass: 7.22e+04, kdiss: 5.91e−03
1563_ID.3216_E3_SLEQHLIGL KD: 9.70e−09 Kass: 1.38e+05, kdiss: 1.34e−03
1564_ID.322_A6_SLLQHAIGL KD: 1.94e−07 Kass: 9.28e+04, kdiss: 1.80e−02
1565_ID.3221_Q7_SLLQHLQGL KD: 9.67e−07 Kass: 2.50e+04, kdiss: 2.42e−02
1566_ID.3222_S5_SLLQSLIGL KD: 2.00e−07 Kass: 8.42e+04, kdiss: 1.69e−02
1567_ID.3223_W6_SLLQHWIGL KD: 1.11e−08 Kass: 1.11e+05, kdiss: 1.24e−03
1568_ID.3225_F1_FLLQHLIGL KD: 1.08e−08 Kass: 1.32e+05, kdiss: 1.42e−03
1569_ID.3228_A4_SLLAHLIGL KD: 9.31e−09 Kass: 1.40e+05, kdiss: 1.30e−03
1570_ID.3229_W1_WLLQHLIGL KD: 9.00e−09 Kass: 1.46e+05, kdiss: 1.31e−03
1571_ID.323_P1_PLLQHLIGL KD: 7.72e−10 Kass: 1.14e+05, kdiss: 8.82e−05
1572_ID.3231_A8_SLLQHLIAL KD: 7.79e−09 Kass: 1.16e+05, kdiss: 9.03e−04
1573_ID.3232_R4_SLLRHLIGL KD: 6.75e−09 Kass: 2.11e+05, kdiss: 1.43e−03
1574_ID.3233_Q8_SLLQHLIQL KD: 2.42e−08 Kass: 9.03e+04, kdiss: 2.19e−03
1575_ID.3235_T7_SLLQHLTGL KD: 4.16e−08 Kass: 1.00e+05, kdiss: 4.18e−03
1576_ID.3236_R4_SLLRHLIGL KD: 7.99e−09 Kass: 2.38e+05, kdiss: 1.90e−03
1577_ID.3237_G7_SLLQHLGGL KD: 2.17e−06 Kass: 1.99e+04, kdiss: 4.31e−02
1578_ID.3239_Q7_SLLQHLQGL KD: 6.40e−07 Kass: 5.29e+04, kdiss: 3.38e−02
1579_ID.3240_P5_SLLQPLIGL KD: 3.75e−07 Kass: 5.75e+04, kdiss: 2.16e−02
1580_ID.3241_D7_SLLQHLDGL KD: 5.38e−07 Kass: 7.27e+02, kdiss: 3.92e−04
1581_ID.3244_S4_SLLSHLIGL KD: 8.41e−09 Kass: 1.57e+05, kdiss: 1.32e−03
1582_ID.3245_P8_SLLQHLIPL KD: 1.11e−08 Kass: 1.34e+05, kdiss: 1.49e−03
1583_ID.325_I3_SLIQHLIGL KD: 1.01e−12 Kass: 1.01e+05, kdiss: 1.02e−07
1584_ID.3250_P4_SLLPHLIGL KD: 7.68e−09 Kass: 1.53e+05, kdiss: 1.17e−03
1585_ID.3251_D6_SLLQHDIGL KD: 1.29e−07 Kass: 6.54e+04, kdiss: 8.43e−03
1586_ID.3252_K4_SLLKHLIGL KD: 8.73e−09 Kass: 2.08e+05, kdiss: 1.81e−03
1587_ID.3253_K7_SLLQHLKGL KD: 8.46e−08 Kass: 8.24e+04, kdiss: 6.97e−03
1588_ID.3254_S4_SLLSHLIGL KD: 7.98e−09 Kass: 1.50e+05, kdiss: 1.20e−03
1589_ID.3255_W7_SLLQHLWGL KD:− Kass:−, , kdiss: 1.83e−02
1590_ID.3256_N3_SLNQHLIGL KD: 3.54e−08 Kass: 1.39e+05, kdiss: 4.93e−03
1591_ID.3257_K1_KLLQHLIGL KD: 9.84e−09 Kass: 1.77e+05, kdiss: 1.74e−03
1592_ID.326_K5_SLLQKLIGL KD: 5.55e−07 Kass: 3.89e+04, kdiss: 2.16e−02
1593_ID.3260_Y4_SLLYHLIGL KD: 1.18e−08 Kass: 1.54e+05, kdiss: 1.81e−03
1594_ID.3261_G7_SLLQHLGGL KD: 1.56e−06 Kass: 2.33e+04, kdiss: 3.64e−02
1595_ID.3267_E7_SLLQHLEGL KD:− Kass:−, , kdiss: 7.77e−04
1596_ID.3269_E7_SLLQHLEGL KD: 1.25e−09 Kass: 8.00e+00, kdiss: 1.00e−08
1597_ID.3270_E3_SLEQHLIGL KD: 1.29e−08 Kass: 1.39e+05, kdiss: 1.80e−03
1598_ID.3271_M8_SLLQHLIML KD: 8.39e−08 Kass: 6.09e+04, kdiss: 5.11e−03
1599_ID.3273_H8_SLLQHLIHL KD: 2.68e−07 Kass: 4.49e+04, kdiss: 1.21e−02
1600_ID.3275_Q7_SLLQHLQGL KD: 9.98e−07 Kass: 2.31e+04, kdiss: 2.30e−02
value
0
5000
10000
15000
20000
Time
0
250
500
750
1000

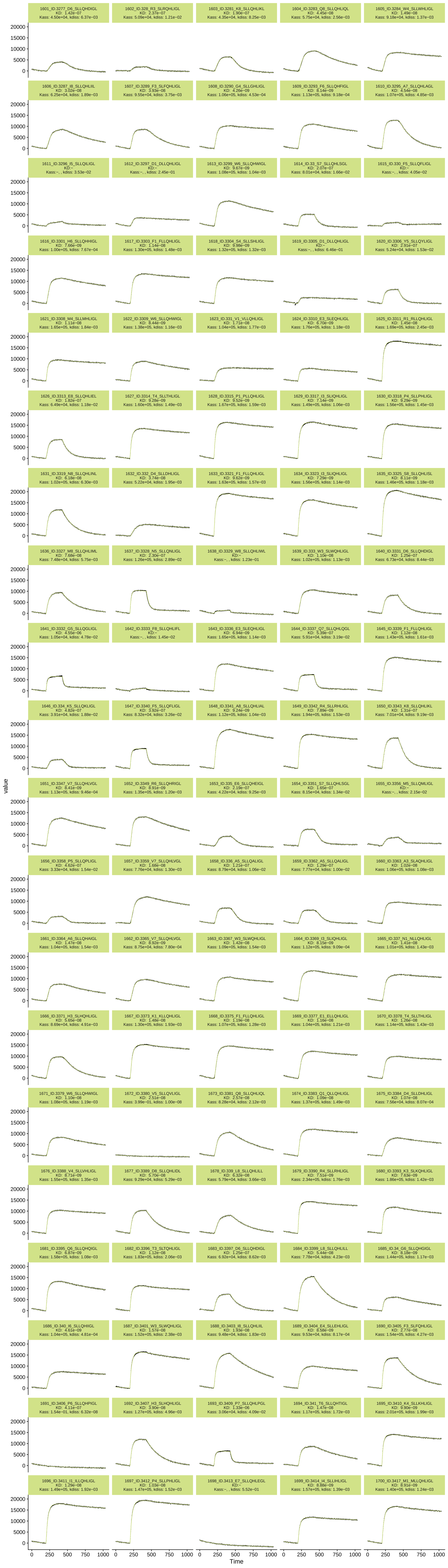

1601_ID.3277_D6_SLLQHDIGL
KD: 1.42e−07
Kass: 4.50e+04, kdiss: 6.37e−03
1602_ID.328_R3_SLRQHLIGL
KD: 2.37e−07
Kass: 5.09e+04, kdiss: 1.21e−02
1603_ID.3281_K8_SLLQHLIKL
KD: 1.90e−07
Kass: 4.35e+04, kdiss: 8.25e−03
1604_ID.3283_Q8_SLLQHLIQL
KD: 4.45e−08
Kass: 5.75e+04, kdiss: 2.56e−03
1605_ID.3284_W4_SLLWHLIGL
KD: 1.49e−08
Kass: 9.18e+04, kdiss: 1.37e−03
1606_ID.3287_I8_SLLQHLIIL
KD: 3.02e−08
Kass: 6.25e+04, kdiss: 1.89e−03
1607_ID.3289_F3_SLFQHLIGL
KD: 3.93e−08
Kass: 9.55e+04, kdiss: 3.75e−03
1608_ID.3290_G4_SLLGHLIGL
KD: 4.26e−09
Kass: 1.06e+05, kdiss: 4.53e−04
1609_ID.3293_F6_SLLQHFIGL
KD: 8.14e−09
Kass: 1.13e+05, kdiss: 9.18e−04
1610_ID.3295_A7_SLLQHLAGL
KD: 4.54e−08
Kass: 1.07e+05, kdiss: 4.85e−03
1611_ID.3296_I5_SLLQILIGL
KD:−
Kass:−, , kdiss: 3.53e−02
1612_ID.3297_D1_DLLQHLIGL
KD:−
Kass:−, , kdiss: 2.45e−01
1613_ID.3299_W6_SLLQHWIGL
KD: 9.67e−09
Kass: 1.08e+05, kdiss: 1.04e−03
1614_ID.33_S7_SLLQHLSGL
KD: 2.07e−07
Kass: 8.01e+04, kdiss: 1.66e−02
1615_ID.330_F5_SLLQFLIGL
KD:−
Kass:−, , kdiss: 4.05e−02
1616_ID.3301_H6_SLLQHHIGL
KD: 7.66e−09
Kass: 1.00e+05, kdiss: 7.67e−04
1617_ID.3303_F1_FLLQHLIGL
KD: 1.14e−08
Kass: 1.30e+05, kdiss: 1.48e−03
1618_ID.3304_S4_SLLSHLIGL
KD: 9.98e−09
Kass: 1.32e+05, kdiss: 1.32e−03
1619_ID.3305_D1_DLLQHLIGL
KD:−
Kass:−, , kdiss: 6.46e−01
1620_ID.3306_Y5_SLLQYLIGL
KD: 2.91e−07
Kass: 5.24e+04, kdiss: 1.53e−02
1621_ID.3308_M4_SLLMHLIGL
KD: 1.11e−08
Kass: 1.65e+05, kdiss: 1.84e−03
1622_ID.3309_W6_SLLQHWIGL
KD: 8.44e−09
Kass: 1.38e+05, kdiss: 1.16e−03
1623_ID.331_V1_VLLQHLIGL
KD: 1.71e−08
Kass: 1.04e+05, kdiss: 1.77e−03
1624_ID.3310_E3_SLEQHLIGL
KD: 6.70e−09
Kass: 1.76e+05, kdiss: 1.18e−03
1625_ID.3311_R1_RLLQHLIGL
KD: 1.45e−08
Kass: 1.69e+05, kdiss: 2.45e−03
1626_ID.3313_E8_SLLQHLIEL
KD: 1.82e−07
Kass: 6.49e+04, kdiss: 1.18e−02
1627_ID.3314_T4_SLLTHLIGL
KD: 9.28e−09
Kass: 1.60e+05, kdiss: 1.49e−03
1628_ID.3315_P1_PLLQHLIGL
KD: 9.52e−09
Kass: 1.67e+05, kdiss: 1.59e−03
1629_ID.3317_I3_SLIQHLIGL
KD: 7.14e−09
Kass: 1.49e+05, kdiss: 1.06e−03
1630_ID.3318_P4_SLLPHLIGL
KD: 9.29e−09
Kass: 1.56e+05, kdiss: 1.45e−03
1631_ID.3319_N8_SLLQHLINL
KD: 6.18e−08
Kass: 1.02e+05, kdiss: 6.30e−03
1632_ID.332_D4_SLLDHLIGL
KD: 3.74e−08
Kass: 5.22e+04, kdiss: 1.95e−03
1633_ID.3321_F1_FLLQHLIGL
KD: 9.62e−09
Kass: 1.63e+05, kdiss: 1.57e−03
1634_ID.3323_I3_SLIQHLIGL
KD: 7.29e−09
Kass: 1.56e+05, kdiss: 1.14e−03
1635_ID.3325_S8_SLLQHLISL
KD: 8.11e−09
Kass: 1.46e+05, kdiss: 1.18e−03
1636_ID.3327_M8_SLLQHLIML
KD: 7.68e−08
Kass: 7.48e+04, kdiss: 5.75e−03
1637_ID.3328_N5_SLLQNLIGL
KD: 2.30e−07
Kass: 1.26e+05, kdiss: 2.89e−02
1638_ID.3329_W8_SLLQHLIWL
KD:−
Kass:−, , kdiss: 1.23e−01
1639_ID.333_W3_SLWQHLIGL
KD: 1.10e−08
Kass: 1.02e+05, kdiss: 1.13e−03
1640_ID.3331_D6_SLLQHDIGL
KD: 1.25e−07
Kass: 6.73e+04, kdiss: 8.44e−03
1641_ID.3332_G5_SLLQGLIGL
KD: 4.55e−06
Kass: 1.05e+04, kdiss: 4.78e−02
1642_ID.3333_F8_SLLQHLIFL
KD:−
Kass:−, , kdiss: 1.45e−02
1643_ID.3336_E3_SLEQHLIGL
KD: 6.94e−09
Kass: 1.65e+05, kdiss: 1.14e−03
1644_ID.3337_Q7_SLLQHLQGL
KD: 5.39e−07
Kass: 5.91e+04, kdiss: 3.19e−02
1645_ID.3339_F1_FLLQHLIGL
KD: 1.12e−08
Kass: 1.43e+05, kdiss: 1.61e−03
1646_ID.334_K5_SLLQKLIGL
KD: 4.82e−07
Kass: 3.91e+04, kdiss: 1.88e−02
1647_ID.3340_F5_SLLQFLIGL
KD: 3.92e−07
Kass: 8.32e+04, kdiss: 3.26e−02
1648_ID.3341_A8_SLLQHLIAL
KD: 9.24e−09
Kass: 1.12e+05, kdiss: 1.04e−03
1649_ID.3342_R4_SLLRHLIGL
KD: 7.89e−09
Kass: 1.94e+05, kdiss: 1.53e−03
1650_ID.3343_K8_SLLQHLIKL
KD: 1.31e−07
Kass: 7.01e+04, kdiss: 9.19e−03
1651_ID.3347_V7_SLLQHLVGL
KD: 8.41e−09
Kass: 1.13e+05, kdiss: 9.46e−04
1652_ID.3349_R6_SLLQHRIGL
KD: 8.91e−09
Kass: 1.35e+05, kdiss: 1.20e−03
1653_ID.335_E6_SLLQHEIGL
KD: 2.19e−07
Kass: 4.22e+04, kdiss: 9.25e−03
1654_ID.3351_S7_SLLQHLSGL
KD: 1.65e−07
Kass: 8.15e+04, kdiss: 1.34e−02
1655_ID.3356_M5_SLLQMLIGL
KD:−
Kass:−, , kdiss: 2.15e−02
1656_ID.3358_P5_SLLQPLIGL
KD: 4.62e−07
Kass: 3.33e+04, kdiss: 1.54e−02
1657_ID.3359_V7_SLLQHLVGL
KD: 1.68e−08
Kass: 7.76e+04, kdiss: 1.30e−03
1658_ID.336_A5_SLLQALIGL
KD: 1.21e−07
Kass: 8.79e+04, kdiss: 1.06e−02
1659_ID.3362_A5_SLLQALIGL
KD: 1.29e−07
Kass: 7.77e+04, kdiss: 1.00e−02
1660_ID.3363_A3_SLAQHLIGL
KD: 1.02e−08
Kass: 1.06e+05, kdiss: 1.08e−03
1661_ID.3364_A6_SLLQHAIGL
KD: 1.47e−08
Kass: 1.04e+05, kdiss: 1.54e−03
1662_ID.3365_V7_SLLQHLVGL
KD: 8.92e−09
Kass: 8.75e+04, kdiss: 7.80e−04
1663_ID.3367_W3_SLWQHLIGL
KD: 1.42e−08
Kass: 1.09e+05, kdiss: 1.54e−03
1664_ID.3369_I3_SLIQHLIGL
KD: 8.15e−09
Kass: 1.12e+05, kdiss: 9.09e−04
1665_ID.337_N1_NLLQHLIGL
KD: 1.41e−08
Kass: 1.01e+05, kdiss: 1.43e−03
1666_ID.3371_H3_SLHQHLIGL
KD: 5.65e−08
Kass: 8.69e+04, kdiss: 4.91e−03
1667_ID.3373_K1_KLLQHLIGL
KD: 1.48e−08
Kass: 1.30e+05, kdiss: 1.93e−03
1668_ID.3375_F1_FLLQHLIGL
KD: 1.19e−08
Kass: 1.07e+05, kdiss: 1.28e−03
1669_ID.3377_E1_ELLQHLIGL
KD: 1.16e−08
Kass: 1.04e+05, kdiss: 1.21e−03
1670_ID.3378_T4_SLLTHLIGL
KD: 1.26e−08
Kass: 1.14e+05, kdiss: 1.43e−03
1671_ID.3379_W6_SLLQHWIGL
KD: 1.10e−08
Kass: 1.08e+05, kdiss: 1.19e−03
1672_ID.3380_V5_SLLQVLIGL
KD: 2.51e−08
Kass: 3.99e−01, kdiss: 1.00e−08
1673_ID.3381_Q8_SLLQHLIQL
KD: 2.57e−08
Kass: 8.28e+04, kdiss: 2.12e−03
1674_ID.3383_Q1_QLLQHLIGL
KD: 1.09e−08
Kass: 1.37e+05, kdiss: 1.49e−03
1675_ID.3384_D4_SLLDHLIGL
KD: 1.07e−08
Kass: 7.56e+04, kdiss: 8.07e−04
1676_ID.3388_V4_SLLVHLIGL
KD: 8.71e−09
Kass: 1.55e+05, kdiss: 1.35e−03
1677_ID.3389_D8_SLLQHLIDL
KD: 5.70e−08
Kass: 9.29e+04, kdiss: 5.29e−03
1678_ID.339_L8_SLLQHLILL
KD: 6.32e−08
Kass: 5.79e+04, kdiss: 3.66e−03
1679_ID.3390_R4_SLLRHLIGL
KD: 7.51e−09
Kass: 2.34e+05, kdiss: 1.76e−03
1680_ID.3393_K3_SLKQHLIGL
KD: 7.63e−09
Kass: 1.86e+05, kdiss: 1.42e−03
1681_ID.3395_Q6_SLLQHQIGL
KD: 6.87e−09
Kass: 1.58e+05, kdiss: 1.08e−03
1682_ID.3396_T3_SLTQHLIGL
KD: 1.12e−08
Kass: 1.83e+05, kdiss: 2.06e−03
1683_ID.3397_D6_SLLQHDIGL
KD: 1.25e−07
Kass: 6.92e+04, kdiss: 8.62e−03
1684_ID.3399_L8_SLLQHLILL
KD: 5.44e−08
Kass: 7.78e+04, kdiss: 4.23e−03
1685_ID.34_G6_SLLQHGIGL
KD: 8.18e−09
Kass: 1.44e+05, kdiss: 1.17e−03
1686_ID.340_I6_SLLQHIIGL
KD: 4.61e−09
Kass: 1.04e+05, kdiss: 4.81e−04
1687_ID.3401_W3_SLWQHLIGL
KD: 1.57e−08
Kass: 1.52e+05, kdiss: 2.38e−03
1688_ID.3403_I8_SLLQHLIIL
KD: 1.93e−08
Kass: 9.48e+04, kdiss: 1.83e−03
1689_ID.3404_E4_SLLEHLIGL
KD: 8.58e−09
Kass: 9.53e+04, kdiss: 8.17e−04
1690_ID.3405_F3_SLFQHLIGL
KD: 2.77e−08
Kass: 1.54e+05, kdiss: 4.27e−03
1691_ID.3406_P6_SLLQHPIGL
KD: 4.11e−07
Kass: 1.54e−01, kdiss: 6.32e−08
1692_ID.3407_H3_SLHQHLIGL
KD: 3.90e−08
Kass: 1.27e+05, kdiss: 4.96e−03
1693_ID.3409_P7_SLLQHLPGL
KD: 1.33e−06
Kass: 3.06e+04, kdiss: 4.09e−02
1694_ID.341_T6_SLLQHTIGL
KD: 1.47e−08
Kass: 1.17e+05, kdiss: 1.72e−03
1695_ID.3410_K4_SLLKHLIGL
KD: 9.90e−09
Kass: 2.01e+05, kdiss: 1.99e−03
1696_ID.3411_I1_ILLQHLIGL
KD: 1.29e−08
Kass: 1.49e+05, kdiss: 1.92e−03
1697_ID.3412_P4_SLLPHLIGL
KD: 1.03e−08
Kass: 1.47e+05, kdiss: 1.52e−03
1698_ID.3413_E7_SLLQHLEGL
KD:−
Kass:−, , kdiss: 5.52e−01
1699_ID.3414_I4_SLLIHLIGL
KD: 8.88e−09
Kass: 1.57e+05, kdiss: 1.39e−03
1700_ID.3417_M1_MLLQHLIGL
KD: 8.91e−09
Kass: 1.40e+05, kdiss: 1.24e−03
value
20000
15000
10000
5000
0
0
250
500
750
1000
Time

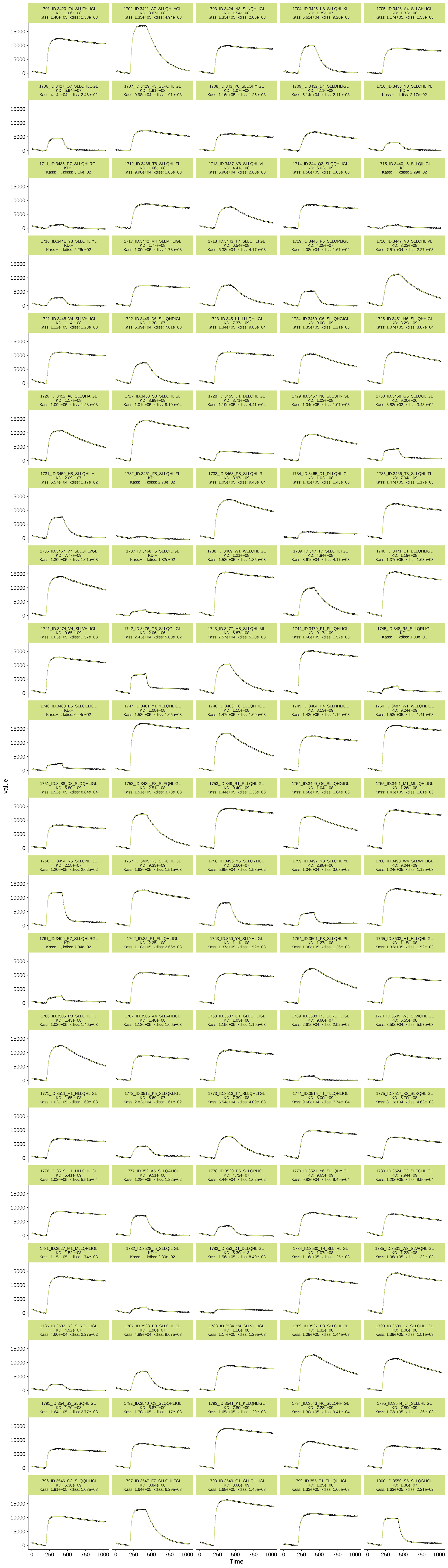
1701_ID.3420_F4_SLLFHLIGL KD: 1.06e−08 Kass: 1.48e+05, kdiss: 1.58e−03
1702_ID.3421_A7_SLLQHLAGL KD: 3.67e−08 Kass: 1.35e+05, kdiss: 4.94e−03
1703_ID.3424_N3_SLNQHLIGL KD: 1.54e−08 Kass: 1.33e+05, kdiss: 2.06e−03
1704_ID.3425_K8_SLLQHLIKL KD: 1.39e−07 Kass: 6.61e+04, kdiss: 9.20e−03
1705_ID.3426_A4_SLLAHLIGL KD: 1.32e−08 Kass: 1.17e+05, kdiss: 1.55e−03
1706_ID.3427_Q7_SLLQHLQGL KD: 5.94e−07 Kass: 4.14e+04, kdiss: 2.46e−02
1707_ID.3429_P3_SLPQHLIGL KD: 1.77e−08 Kass: 9.98e+04, kdiss: 1.91e−03
1708_ID.343_Y6_SLLQHYIGL KD: 1.07e−08 Kass: 1.16e+05, kdiss: 1.25e−03
1709_ID.3432_D4_SLLDHLIGL KD: 4.08e−08 Kass: 5.14e+04, kdiss: 2.11e−03
1710_ID.3433_Y8_SLLQHLIYL KD:~ Kass:~ , kdiss: 2.17e−02
1711_ID.3435_R7_SLLQHLRGL KD:~ Kass:~ , kdiss: 3.16e−02
1712_ID.3436_T8_SLLQHLITL KD: 1.06e−08 Kass: 9.98e+04, kdiss: 1.06e−03
1713_ID.3437_V8_SLLQHLIVL KD: 4.41e−08 Kass: 5.90e+04, kdiss: 2.60e−03
1714_ID.344_Q3_SLQQHLIGL KD: 6.62e−09 Kass: 1.58e+05, kdiss: 1.05e−03
1715_ID.3440_I5_SLLQILIGL KD:~ Kass:~ , kdiss: 2.29e−02
1716_ID.3441_Y8_SLLQHLIYL KD:~ Kass:~ , kdiss: 2.26e−02
1717_ID.3442_M4_SLLMHLIGL KD: 1.78e−08 Kass: 1.00e+05, kdiss: 1.78e−03
1718_ID.3443_T7_SLLQHLTGL KD: 6.54e−08 Kass: 6.38e+04, kdiss: 4.17e−03
1719_ID.3446_P5_SLLQPLIGL KD: 4.11e−07 Kass: 4.08e+04, kdiss: 1.67e−02
1720_ID.3447_V8_SLLQHLIVL KD: 3.03e−08 Kass: 7.51e+04, kdiss: 2.27e−03
1721_ID.3448_V4_SLLVHLIGL KD: 1.14e−08 Kass: 1.12e+05, kdiss: 1.28e−03
1722_ID.3449_D6_SLLQHDIGL KD: 1.30e−07 Kass: 5.39e+04, kdiss: 7.01e−03
1723_ID.345_L1_LLLQHLIGL KD: 7.37e−09 Kass: 1.34e+05, kdiss: 9.86e−04
1724_ID.3450_G6_SLLQHGIGL KD: 9.00e−09 Kass: 1.35e+05, kdiss: 1.21e−03
1725_ID.3451_H6_SLLQHHIGL KD: 8.29e−09 Kass: 1.07e+05, kdiss: 8.87e−04
1726_ID.3452_A6_SLLQHAIGL KD: 1.17e−08 Kass: 1.09e+05, kdiss: 1.28e−03
1727_ID.3453_S8_SLLQHLISL KD: 8.99e−09 Kass: 1.01e+05, kdiss: 9.10e−04
1728_ID.3455_D1_DLLQHLIGL KD: 3.71e−09 Kass: 1.19e+05, kdiss: 4.41e−04
1729_ID.3457_N6_SLLQHNIGL KD: 1.03e−08 Kass: 1.04e+05, kdiss: 1.07e−03
1730_ID.3458_G5_SLLQGLIGL KD: 9.00e−06 Kass: 3.82e+03, kdiss: 3.43e−02
1731_ID.3459_H8_SLLQHLIHL KD: 2.09e−07 Kass: 5.57e+04, kdiss: 1.17e−02
1732_ID.3461_F8_SLLQHLIFL KD:~ Kass:~ , kdiss: 2.73e−02
1733_ID.3463_R8_SLLQHLIRL KD: 8.97e−09 Kass: 1.05e+05, kdiss: 9.43e−04
1734_ID.3465_D1_DLLQHLIGL KD: 1.02e−08 Kass: 1.41e+05, kdiss: 1.43e−03
1735_ID.3466_T8_SLLQHLITL KD: 7.94e−09 Kass: 1.47e+05, kdiss: 1.17e−03
1736_ID.3467_V7_SLLQHLVGL KD: 7.77e−09 Kass: 1.30e+05, kdiss: 1.01e−03
1737_ID.3468_I5_SLLQILIGL KD:~ Kass:~ , kdiss: 1.82e−02
1738_ID.3469_W1_WLLQHLIGL KD: 1.21e−08 Kass: 1.52e+05, kdiss: 1.85e−03
1739_ID.347_T7_SLLQHLTGL KD: 4.84e−08 Kass: 8.61e+04, kdiss: 4.17e−03
1740_ID.3471_E1_ELLQHLIGL KD: 1.19e−08 Kass: 1.37e+05, kdiss: 1.63e−03
1741_ID.3474_V4_SLLVHLIGL KD: 9.65e−09 Kass: 1.63e+05, kdiss: 1.57e−03
1742_ID.3476_G5_SLLQGLIGL KD: 2.06e−06 Kass: 2.43e+04, kdiss: 5.00e−02
1743_ID.3477_M8_SLLQHLIML KD: 6.87e−08 Kass: 7.57e+04, kdiss: 5.20e−03
1744_ID.3479_F1_FLLQHLIGL KD: 9.17e−09 Kass: 1.66e+05, kdiss: 1.52e−03
1745_ID.348_R5_SLLQRLIGL KD:~ Kass:~ , kdiss: 1.08e−01
1746_ID.3480_E5_SLLQELIGL KD:~ Kass:~ , kdiss: 6.44e−02
1747_ID.3481_Y1_YLLQHLIGL KD: 1.08e−08 Kass: 1.53e+05, kdiss: 1.65e−03
1748_ID.3483_T5_SLLQTLIGL KD: 1.15e−08 Kass: 1.47e+05, kdiss: 1.69e−03
1749_ID.3484_H4_SLLHHLIGL KD: 8.13e−09 Kass: 1.43e+05, kdiss: 1.16e−03
1750_ID.3487_W1_WLLQHLIGL KD: 9.24e−09 Kass: 1.53e+05, kdiss: 1.41e−03
1751_ID.3488_D3_SLDQHLIGL KD: 5.80e−09 Kass: 1.52e+05, kdiss: 8.84e−04
1752_ID.3489_F3_SLFQHLIGL KD: 2.51e−08 Kass: 1.51e+05, kdiss: 3.78e−03
1753_ID.349_R1_RLLQHLIGL KD: 9.40e−09 Kass: 1.44e+05, kdiss: 1.36e−03
1754_ID.3490_G6_SLLQHGIGL KD: 1.04e−08 Kass: 1.58e+05, kdiss: 1.64e−03
1755_ID.3491_M1_MLLQHLIGL KD: 1.26e−08 Kass: 1.43e+05, kdiss: 1.81e−03
1756_ID.3494_N5_SLLQNLIGL KD: 2.18e−07 Kass: 1.20e+05, kdiss: 2.62e−02
1757_ID.3495_K3_SLKQHLIGL KD: 9.33e−09 Kass: 1.62e+05, kdiss: 1.51e−03
1758_ID.3496_Y5_SLLQYLIGL KD: 2.66e−07 Kass: 5.95e+04, kdiss: 1.58e−02
1759_ID.3497_Y8_SLLQHLIYL KD: 2.98e−06 Kass: 1.04e+04, kdiss: 3.08e−02
1760_ID.3498_W4_SLLWHLIGL KD: 9.04e−09 Kass: 1.24e+05, kdiss: 1.12e−03
1761_ID.3499_R7_SLLQHLRGL KD:~ Kass:~ , kdiss: 7.04e−02
1762_ID.35_F1_FLLQHLIGL KD: 2.25e−08 Kass: 1.18e+05, kdiss: 2.66e−03
1763_ID.350_Y4_SLLYHLIGL KD: 1.11e−08 Kass: 1.37e+05, kdiss: 1.52e−03
1764_ID.3501_P8_SLLQHLIPL KD: 1.26e−08 Kass: 1.08e+05, kdiss: 1.36e−03
1765_ID.3503_H1_HLLQHLIGL KD: 1.15e−08 Kass: 1.32e+05, kdiss: 1.52e−03
1766_ID.3505_P8_SLLQHLIPL KD: 1.43e−08 Kass: 1.02e+05, kdiss: 1.46e−03
1767_ID.3506_A4_SLLAHLIGL KD: 1.46e−08 Kass: 1.13e+05, kdiss: 1.66e−03
1768_ID.3507_G1_GLLQHLIGL KD: 1.03e−08 Kass: 1.15e+05, kdiss: 1.19e−03
1769_ID.3508_R3_SLRQHLIGL KD: 9.65e−07 Kass: 2.61e+04, kdiss: 2.52e−02
1770_ID.3509_W3_SLWQHLIGL KD: 6.55e−08 Kass: 8.50e+04, kdiss: 5.57e−03
1771_ID.3511_H1_HLLQHLIGL KD: 1.65e−08 Kass: 1.02e+05, kdiss: 1.69e−03
1772_ID.3512_K5_SLLQKLIGL KD: 5.69e−07 Kass: 2.83e+04, kdiss: 1.61e−02
1773_ID.3513_T7_SLLQHLTGL KD: 7.39e−08 Kass: 5.54e+04, kdiss: 4.09e−03
1774_ID.3515_T1_TLLQHLIGL KD: 8.00e−09 Kass: 9.68e+04, kdiss: 7.74e−04
1775_ID.3517_K3_SLKQHLIGL KD: 5.70e−08 Kass: 8.11e+04, kdiss: 4.63e−03
1776_ID.3519_H1_HLLQHLIGL KD: 5.41e−09 Kass: 1.02e+05, kdiss: 5.51e−04
1777_ID.352_A5_SLLQALIGL KD: 9.51e−08 Kass: 1.28e+05, kdiss: 1.22e−02
1778_ID.3520_P5_SLLQPLIGL KD: 4.72e−07 Kass: 3.44e+04, kdiss: 1.62e−02
1779_ID.3521_Y6_SLLQHYIGL KD: 9.65e−09 Kass: 9.82e+04, kdiss: 9.49e−04
1780_ID.3524_E3_SLEQHLIGL KD: 7.94e−09 Kass: 1.20e+05, kdiss: 9.50e−04
1781_ID.3527_M1_MLLQHLIGL KD: 1.52e−08 Kass: 1.15e+05, kdiss: 1.74e−03
1782_ID.3528_I5_SLLQILIGL KD:~ Kass:~ , kdiss: 2.80e−02
1783_ID.353_D1_DLLQHLIGL KD: 5.39e−13 Kass: 1.56e+05, kdiss: 8.40e−08
1784_ID.3530_T4_SLLTHLIGL KD: 1.07e−08 Kass: 1.16e+05, kdiss: 1.25e−03
1785_ID.3531_W3_SLWQHLIGL KD: 1.22e−08 Kass: 1.08e+05, kdiss: 1.32e−03
1786_ID.3532_R3_SLRQHLIGL KD: 4.92e−07 Kass: 4.60e+04, kdiss: 2.27e−02
1787_ID.3533_E8_SLLQHLIEL KD: 1.98e−07 Kass: 4.89e+04, kdiss: 9.67e−03
1788_ID.3534_V4_SLLVHLIGL KD: 1.10e−08 Kass: 1.17e+05, kdiss: 1.29e−03
1789_ID.3537_P8_SLLQHLIPL KD: 1.32e−08 Kass: 1.09e+05, kdiss: 1.44e−03
1790_ID.3539_L7_SLLQHLLGL KD: 1.08e−08 Kass: 1.39e+05, kdiss: 1.51e−03
1791_ID.354_S3_SLSQHLIGL KD: 1.70e−08 Kass: 1.64e+05, kdiss: 2.77e−03
1792_ID.3540_Q3_SLQQHLIGL KD: 6.87e−09 Kass: 1.70e+05, kdiss: 1.17e−03
1793_ID.3541_K1_KLLQHLIGL KD: 7.80e−09 Kass: 1.65e+05, kdiss: 1.29e−03
1794_ID.3543_H6_SLLQHHIGL KD: 7.23e−09 Kass: 1.30e+05, kdiss: 9.41e−04
1795_ID.3544_L4_SLLLHLIGL KD: 7.89e−09 Kass: 1.72e+05, kdiss: 1.36e−03
1796_ID.3546_Q3_SLQQHLIGL KD: 5.38e−09 Kass: 1.91e+05, kdiss: 1.03e−03
1797_ID.3547_F7_SLLQHLFGL KD: 3.84e−08 Kass: 1.64e+05, kdiss: 6.29e−03
1798_ID.3549_G1_GLLQHLIGL KD: 8.66e−09 Kass: 1.68e+05, kdiss: 1.45e−03
1799_ID.355_T1_TLLQHLIGL KD: 1.25e−08 Kass: 1.32e+05, kdiss: 1.66e−03
1800_ID.3550_S5_SLLQSLIGL KD: 1.36e−07 Kass: 1.63e+05, kdiss: 2.21e−02
value
Time
0
5000
10000
15000
0 250 500 750 1000

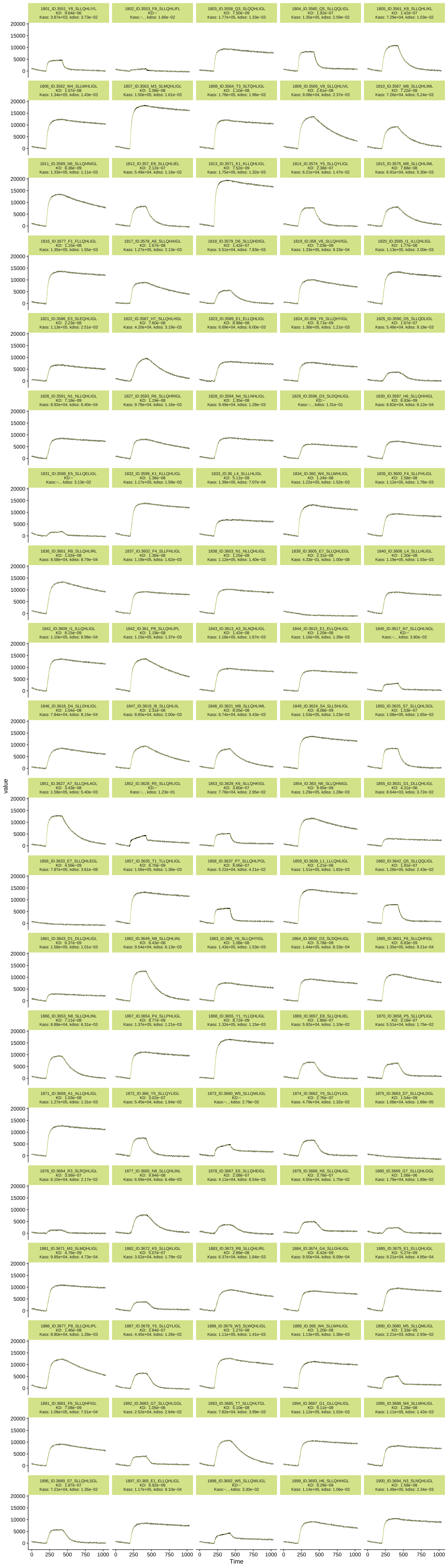
value
Time
20000
15000
10000
5000
0
250
500
750
1000

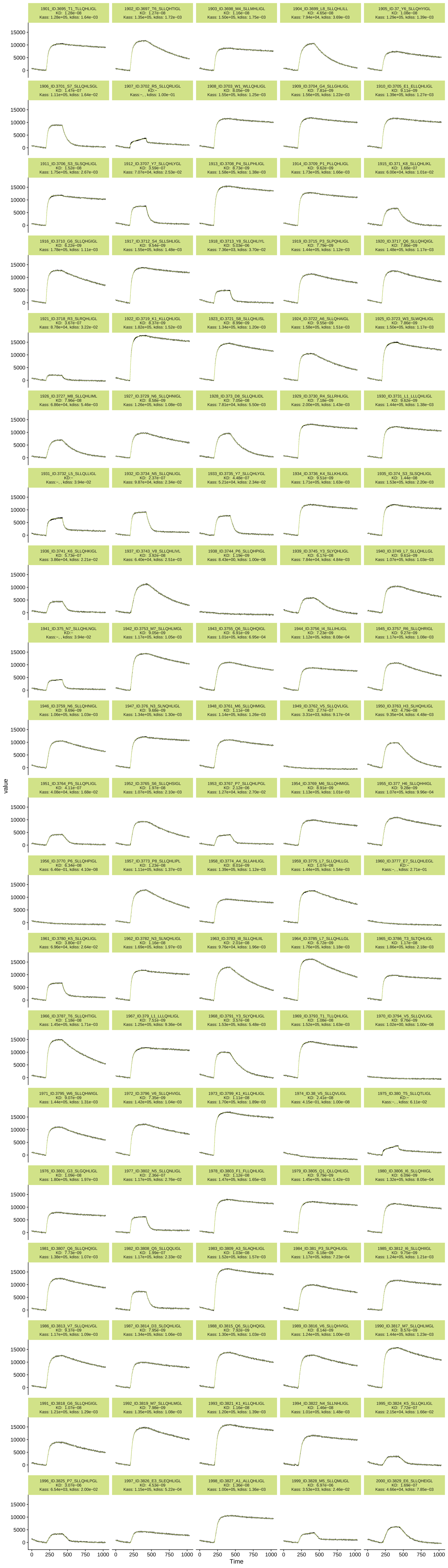
value
Time
0
5000
10000
15000
0
250
500
750
1000
1901_ID.3695_T1_TLLQHLIGL
2000_ID.3829_E6_SLLQHEIGL

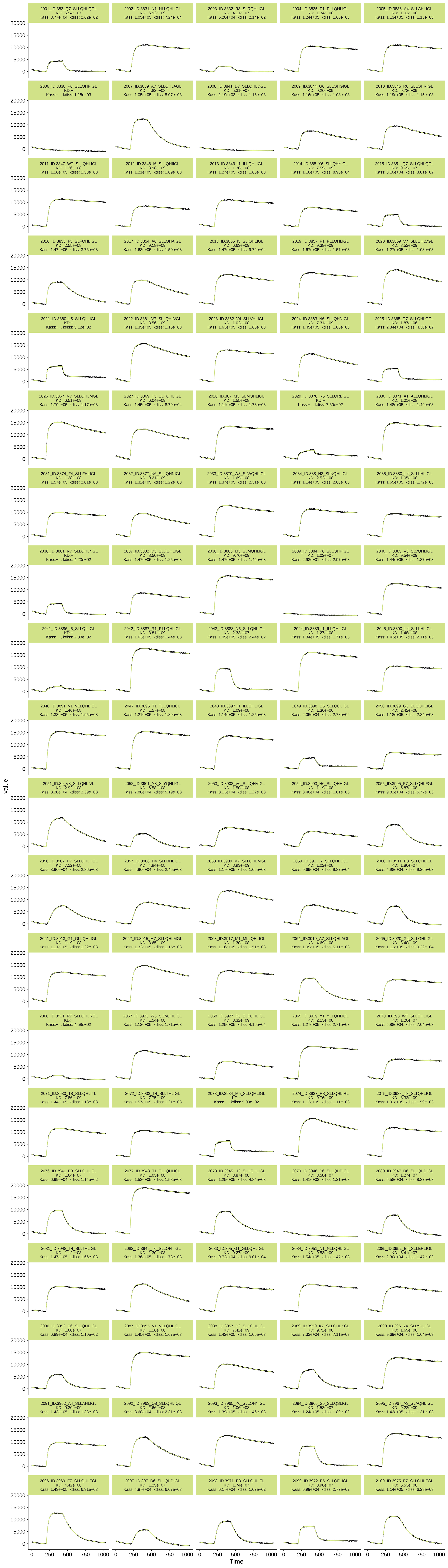

2001_ID.383_Q7_SLLQHLQGL
KD: 6.94e−07
Kass: 3.77e+04, kdiss: 2.62e−02
2002_ID.3831_N1_NLLQHLIGL
KD: 6.92e−09
Kass: 1.05e+05, kdiss: 7.24e−04
2003_ID.3832_R3_SLRQHLIGL
KD: 4.11e−07
Kass: 5.20e+04, kdiss: 2.14e−02
2004_ID.3835_P1_PLLQHLIGL
KD: 1.34e−08
Kass: 1.24e+05, kdiss: 1.66e−03
2005_ID.3836_A4_SLLAHLIGL
KD: 1.01e−08
Kass: 1.13e+05, kdiss: 1.15e−03
2006_ID.3838_P6_SLLQHPIGL
KD:−
Kass:−, , kdiss: 1.18e−03
2007_ID.3839_A7_SLLQHLAGL
KD: 4.82e−08
Kass: 1.05e+05, kdiss: 5.07e−03
2008_ID.3841_D7_SLLQHLDGL
KD: 5.31e−07
Kass: 2.19e+03, kdiss: 1.16e−03
2009_ID.3844_G6_SLLQHGIGL
KD: 9.26e−09
Kass: 1.16e+05, kdiss: 1.08e−03
2010_ID.3845_R6_SLLQHRIGL
KD: 9.72e−09
Kass: 1.19e+05, kdiss: 1.15e−03
2011_ID.3847_WT_SLLQHLIGL
KD: 1.36e−08
Kass: 1.16e+05, kdiss: 1.58e−03
2012_ID.3848_I6_SLLQHIIGL
KD: 8.98e−09
Kass: 1.21e+05, kdiss: 1.09e−03
2013_ID.3849_I1_ILLQHLIGL
KD: 1.30e−08
Kass: 1.27e+05, kdiss: 1.65e−03
2014_ID.385_Y6_SLLQHYIGL
KD: 7.59e−09
Kass: 1.18e+05, kdiss: 8.95e−04
2015_ID.3851_Q7_SLLQHLQGL
KD: 9.69e−07
Kass: 3.10e+04, kdiss: 3.01e−02
2016_ID.3853_F3_SLFQHLIGL
KD: 2.55e−08
Kass: 1.47e+05, kdiss: 3.76e−03
2017_ID.3854_A6_SLLQHAIGL
KD: 9.18e−09
Kass: 1.63e+05, kdiss: 1.50e−03
2018_ID.3855_I3_SLIQHLIGL
KD: 6.63e−09
Kass: 1.47e+05, kdiss: 9.72e−04
2019_ID.3857_P1_PLLQHLIGL
KD: 9.38e−09
Kass: 1.67e+05, kdiss: 1.57e−03
2020_ID.3859_V7_SLLQHLVGL
KD: 8.52e−09
Kass: 1.27e+05, kdiss: 1.08e−03
2021_ID.3860_L5_SLLQLLIGL
KD:−
Kass:−, , kdiss: 5.12e−02
2022_ID.3861_V7_SLLQHLVGL
KD: 8.56e−09
Kass: 1.35e+05, kdiss: 1.15e−03
2023_ID.3862_V4_SLLVHLIGL
KD: 1.02e−08
Kass: 1.63e+05, kdiss: 1.66e−03
2024_ID.3863_N6_SLLQHNIGL
KD: 7.31e−09
Kass: 1.45e+05, kdiss: 1.06e−03
2025_ID.3865_G7_SLLQHLGGL
KD: 1.87e−06
Kass: 2.34e+04, kdiss: 4.38e−02
2026_ID.3867_M7_SLLQHLMGL
KD: 6.51e−09
Kass: 1.79e+05, kdiss: 1.17e−03
2027_ID.3869_P3_SLPQHLIGL
KD: 6.04e−09
Kass: 1.45e+05, kdiss: 8.79e−04
2028_ID.387_M3_SLMQHLIGL
KD: 1.55e−08
Kass: 1.11e+05, kdiss: 1.73e−03
2029_ID.3870_R5_SLLQRLIGL
KD:−
Kass:−, , kdiss: 7.60e−02
2030_ID.3871_A1_ALLQHLIGL
KD: 1.01e−08
Kass: 1.48e+05, kdiss: 1.49e−03
2031_ID.3874_F4_SLLFHLIGL
KD: 1.28e−08
Kass: 1.57e+05, kdiss: 2.01e−03
2032_ID.3877_N6_SLLQHNIGL
KD: 9.21e−09
Kass: 1.32e+05, kdiss: 1.22e−03
2033_ID.3879_W3_SLWQHLIGL
KD: 1.69e−08
Kass: 1.37e+05, kdiss: 2.31e−03
2034_ID.388_N3_SLNQHLIGL
KD: 2.52e−08
Kass: 1.14e+05, kdiss: 2.88e−03
2035_ID.3880_L4_SLLLHLIGL
KD: 1.05e−08
Kass: 1.65e+05, kdiss: 1.72e−03
2036_ID.3881_N7_SLLQHLNGL
KD:−
Kass:−, , kdiss: 4.23e−02
2037_ID.3882_D3_SLDQHLIGL
KD: 8.50e−09
Kass: 1.47e+05, kdiss: 1.25e−03
2038_ID.3883_M3_SLMQHLIGL
KD: 9.76e−09
Kass: 1.47e+05, kdiss: 1.44e−03
2039_ID.3884_P6_SLLQHPIGL
KD: 1.02e−07
Kass: 2.93e−01, kdiss: 2.97e−08
2040_ID.3885_V3_SLVQHLIGL
KD: 9.54e−09
Kass: 1.44e+05, kdiss: 1.37e−03
2041_ID.3886_I5_SLLQILIGL
KD:−
Kass:−, , kdiss: 2.83e−02
2042_ID.3887_R1_RLLQHLIGL
KD: 8.81e−09
Kass: 1.63e+05, kdiss: 1.44e−03
2043_ID.3888_N5_SLLQNLIGL
KD: 2.33e−07
Kass: 1.05e+05, kdiss: 2.44e−02
2044_ID.3889_I1_ILLQHLIGL
KD: 1.27e−08
Kass: 1.34e+05, kdiss: 1.71e−03
2045_ID.3890_L4_SLLLHLIGL
KD: 1.48e−08
Kass: 1.43e+05, kdiss: 2.11e−03
2046_ID.3891_V1_VLLQHLIGL
KD: 1.46e−08
Kass: 1.33e+05, kdiss: 1.95e−03
2047_ID.3895_T1_TLLQHLIGL
KD: 1.57e−08
Kass: 1.21e+05, kdiss: 1.89e−03
2048_ID.3897_I1_ILLQHLIGL
KD: 1.09e−08
Kass: 1.14e+05, kdiss: 1.25e−03
2049_ID.3898_G5_SLLQGLIGL
KD: 1.36e−06
Kass: 2.05e+04, kdiss: 2.78e−02
2050_ID.3899_G3_SLGQHLIGL
KD: 2.42e−08
Kass: 1.18e+05, kdiss: 2.84e−03
2051_ID.39_V8_SLLQHLIVL
KD: 2.92e−08
Kass: 8.20e+04, kdiss: 2.39e−03
2052_ID.3901_Y3_SLYQHLIGL
KD: 6.58e−08
Kass: 7.88e+04, kdiss: 5.19e−03
2053_ID.3902_V6_SLLQHVIGL
KD: 1.50e−08
Kass: 8.13e+04, kdiss: 1.22e−03
2054_ID.3903_H6_SLLQHHIGL
KD: 1.19e−08
Kass: 8.48e+04, kdiss: 1.01e−03
2055_ID.3905_F7_SLLQHLFGL
KD: 5.87e−08
Kass: 9.82e+04, kdiss: 5.77e−03
2056_ID.3907_H7_SLLQHLHGL
KD: 7.22e−08
Kass: 3.96e+04, kdiss: 2.86e−03
2057_ID.3908_D4_SLLDHLIGL
KD: 4.94e−08
Kass: 4.96e+04, kdiss: 2.45e−03
2058_ID.3909_M7_SLLQHLMGL
KD: 8.93e−09
Kass: 1.17e+05, kdiss: 1.05e−03
2059_ID.391_L7_SLLQHLLGL
KD: 1.02e−08
Kass: 9.69e+04, kdiss: 9.87e−04
2060_ID.3911_E8_SLLQHLIEL
KD: 1.86e−07
Kass: 4.98e+04, kdiss: 9.26e−03
2061_ID.3913_G1_GLLQHLIGL
KD: 1.19e−08
Kass: 1.11e+05, kdiss: 1.32e−03
2062_ID.3915_M7_SLLQHLMGL
KD: 8.65e−09
Kass: 1.33e+05, kdiss: 1.15e−03
2063_ID.3917_M1_MLLQHLIGL
KD: 1.30e−08
Kass: 1.16e+05, kdiss: 1.51e−03
2064_ID.3919_A7_SLLQHLAGL
KD: 4.69e−08
Kass: 1.09e+05, kdiss: 5.11e−03
2065_ID.3920_G4_SLLGHLIGL
KD: 8.40e−09
Kass: 1.11e+05, kdiss: 9.32e−04
2066_ID.3921_R7_SLLQHLRGL
KD:−
Kass:−, , kdiss: 4.58e−02
2067_ID.3923_W3_SLWQHLIGL
KD: 1.54e−08
Kass: 1.12e+05, kdiss: 1.71e−03
2068_ID.3927_P3_SLPQHLIGL
KD: 3.32e−09
Kass: 1.25e+05, kdiss: 4.16e−04
2069_ID.3929_Y1_YLLQHLIGL
KD: 2.13e−08
Kass: 1.27e+05, kdiss: 2.71e−03
2070_ID.393_WT_SLLQHLIGL
KD: 1.20e−07
Kass: 5.88e+04, kdiss: 7.04e−03
2071_ID.3930_T8_SLLQHLITL
KD: 7.86e−09
Kass: 1.44e+05, kdiss: 1.13e−03
2072_ID.3932_T4_SLLTHLIGL
KD: 7.75e−09
Kass: 1.57e+05, kdiss: 1.21e−03
2073_ID.3934_M5_SLLQMLIGL
KD:−
Kass:−, , kdiss: 5.09e−02
2074_ID.3937_R8_SLLQHLIRL
KD: 9.76e−09
Kass: 1.13e+05, kdiss: 1.11e−03
2075_ID.3938_T3_SLTQHLIGL
KD: 8.32e−09
Kass: 1.91e+05, kdiss: 1.59e−03
2076_ID.3941_E8_SLLQHLIEL
KD: 1.64e−07
Kass: 6.99e+04, kdiss: 1.14e−02
2077_ID.3943_T1_TLLQHLIGL
KD: 1.03e−08
Kass: 1.53e+05, kdiss: 1.58e−03
2078_ID.3945_H3_SLHQHLIGL
KD: 3.87e−08
Kass: 1.25e+05, kdiss: 4.84e−03
2079_ID.3946_P6_SLLQHPIGL
KD: 8.58e−07
Kass: 1.41e+03, kdiss: 1.21e−03
2080_ID.3947_D6_SLLQHDIGL
KD: 1.27e−07
Kass: 6.58e+04, kdiss: 8.37e−03
2081_ID.3948_T4_SLLTHLIGL
KD: 1.12e−08
Kass: 1.47e+05, kdiss: 1.66e−03
2082_ID.3949_T6_SLLQHTIGL
KD: 1.30e−08
Kass: 1.36e+05, kdiss: 1.78e−03
2083_ID.395_G1_GLLQHLIGL
KD: 9.27e−09
Kass: 9.72e+04, kdiss: 9.01e−04
2084_ID.3951_N1_NLLQHLIGL
KD: 9.53e−09
Kass: 1.54e+05, kdiss: 1.47e−03
2085_ID.3952_E4_SLLEHLIGL
KD: 6.41e−07
Kass: 2.30e+04, kdiss: 1.47e−02
2086_ID.3953_E6_SLLQHEIGL
KD: 1.60e−07
Kass: 6.89e+04, kdiss: 1.10e−02
2087_ID.3955_V1_VLLQHLIGL
KD: 1.16e−08
Kass: 1.45e+05, kdiss: 1.67e−03
2088_ID.3957_P3_SLPQHLIGL
KD: 7.42e−09
Kass: 1.42e+05, kdiss: 1.05e−03
2089_ID.3959_K7_SLLQHLKGL
KD: 9.72e−08
Kass: 7.32e+04, kdiss: 7.11e−03
2090_ID.396_Y4_SLLYHLIGL
KD: 1.69e−08
Kass: 9.69e+04, kdiss: 1.64e−03
2091_ID.3962_A4_SLLAHLIGL
KD: 9.30e−09
Kass: 1.43e+05, kdiss: 1.33e−03
2092_ID.3963_Q8_SLLQHLIQL
KD: 2.66e−08
Kass: 8.68e+04, kdiss: 2.31e−03
2093_ID.3965_Y6_SLLQHYIGL
KD: 1.06e−08
Kass: 1.39e+05, kdiss: 1.46e−03
2094_ID.3966_S5_SLLQSLIGL
KD: 1.53e−07
Kass: 1.24e+05, kdiss: 1.89e−02
2095_ID.3967_A3_SLAQHLIGL
KD: 9.22e−09
Kass: 1.42e+05, kdiss: 1.31e−03
2096_ID.3969_F7_SLLQHLFGL
KD: 4.42e−08
Kass: 1.43e+05, kdiss: 6.31e−03
2097_ID.397_D6_SLLQHDIGL
KD: 1.25e−07
Kass: 4.87e+04, kdiss: 6.07e−03
2098_ID.3971_E8_SLLQHLIEL
KD: 1.74e−07
Kass: 6.17e+04, kdiss: 1.07e−02
2099_ID.3972_F5_SLLQFLIGL
KD: 3.96e−07
Kass: 6.99e+04, kdiss: 2.77e−02
2100_ID.3975_F7_SLLQHLFGL
KD: 5.53e−08
Kass: 1.14e+05, kdiss: 6.28e−03
value
20000
15000
10000
5000
0
0
250
500
750
1000
Time

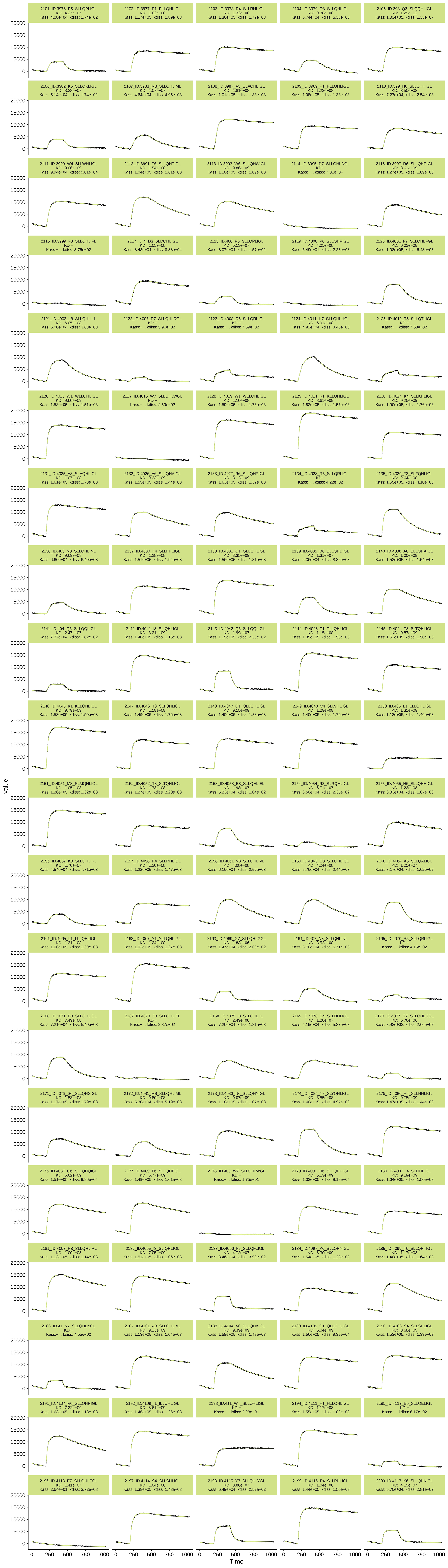
value
Time
0
250
500
750
1000
20000
15000
10000
5000
0

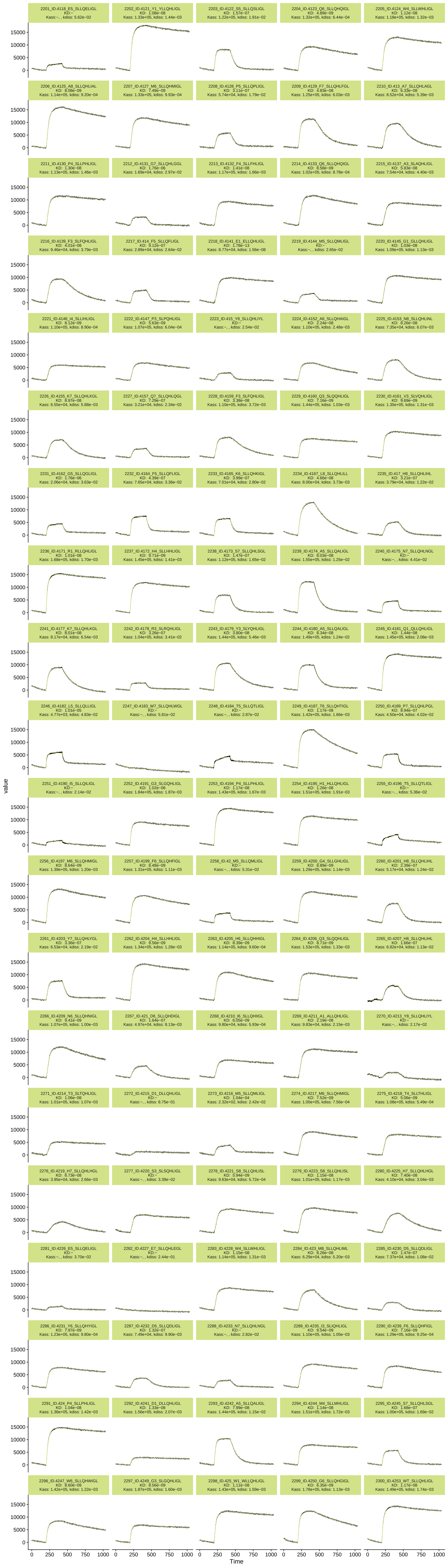

2201_ID.4118_E5_SLLQELIGL
KD:−
Kass:−, , kdiss: 5.62e−02
2202_ID.4121_Y1_YLLQHLIGL
KD: 1.08e−08
Kass: 1.33e+05, kdiss: 1.44e−03
2203_ID.4122_S5_SLLQSLIGL
KD: 1.57e−07
Kass: 1.22e+05, kdiss: 1.91e−02
2204_ID.4123_Q6_SLLQHQIGL
KD: 4.89e−09
Kass: 1.32e+05, kdiss: 6.44e−04
2205_ID.4124_W4_SLLWHLIGL
KD: 1.12e−08
Kass: 1.18e+05, kdiss: 1.32e−03
2206_ID.4125_A8_SLLQHLIAL
KD: 8.08e−09
Kass: 1.14e+05, kdiss: 9.20e−04
2207_ID.4127_M6_SLLQHMIGL
KD: 7.49e−09
Kass: 1.33e+05, kdiss: 9.93e−04
2208_ID.4128_P5_SLLQPLIGL
KD: 3.11e−07
Kass: 5.74e+04, kdiss: 1.79e−02
2209_ID.4129_F7_SLLQHLFGL
KD: 4.83e−08
Kass: 1.25e+05, kdiss: 6.03e−03
2210_ID.413_A7_SLLQHLAGL
KD: 6.33e−08
Kass: 8.52e+04, kdiss: 5.39e−03
2211_ID.4130_P4_SLLPHLIGL
KD: 1.30e−08
Kass: 1.13e+05, kdiss: 1.46e−03
2212_ID.4131_G7_SLLQHLGGL
KD: 1.76e−06
Kass: 1.69e+04, kdiss: 2.97e−02
2213_ID.4132_F4_SLLFHLIGL
KD: 1.41e−08
Kass: 1.17e+05, kdiss: 1.66e−03
2214_ID.4133_Q6_SLLQHQIGL
KD: 8.58e−09
Kass: 1.02e+05, kdiss: 8.78e−04
2215_ID.4137_A3_SLAQHLIGL
KD: 5.83e−08
Kass: 7.54e+04, kdiss: 4.40e−03
2216_ID.4139_F3_SLFQHLIGL
KD: 4.01e−08
Kass: 9.46e+04, kdiss: 3.79e−03
2217_ID.414_F5_SLLQFLIGL
KD: 9.12e−07
Kass: 2.89e+04, kdiss: 2.64e−02
2218_ID.4141_E1_ELLQHLIGL
KD: 1.78e−13
Kass: 8.77e+04, kdiss: 1.56e−08
2219_ID.4144_M5_SLLQMLIGL
KD:−
Kass:−, , kdiss: 2.65e−02
2220_ID.4145_G1_GLLQHLIGL
KD: 1.03e−08
Kass: 1.09e+05, kdiss: 1.13e−03
2221_ID.4146_I4_SLLIHLIGL
KD: 8.12e−09
Kass: 1.10e+05, kdiss: 8.90e−04
2222_ID.4147_P3_SLPQHLIGL
KD: 5.63e−09
Kass: 1.07e+05, kdiss: 6.04e−04
2223_ID.415_Y8_SLLQHLIYL
KD:−
Kass:−, , kdiss: 2.54e−02
2224_ID.4152_A6_SLLQHAIGL
KD: 2.24e−08
Kass: 1.10e+05, kdiss: 2.48e−03
2225_ID.4153_N8_SLLQHLINL
KD: 8.26e−08
Kass: 7.35e+04, kdiss: 6.07e−03
2226_ID.4155_K7_SLLQHLKGL
KD: 8.97e−08
Kass: 6.55e+04, kdiss: 5.88e−03
2227_ID.4157_Q7_SLLQHLQGL
KD: 7.29e−07
Kass: 3.21e+04, kdiss: 2.34e−02
2228_ID.4159_F3_SLFQHLIGL
KD: 3.38e−08
Kass: 1.10e+05, kdiss: 3.72e−03
2229_ID.4160_Q3_SLQQHLIGL
KD: 7.16e−09
Kass: 1.44e+05, kdiss: 1.03e−03
2230_ID.4161_V3_SLVQHLIGL
KD: 9.69e−09
Kass: 1.35e+05, kdiss: 1.31e−03
2231_ID.4162_G5_SLLQGLIGL
KD: 1.76e−06
Kass: 2.06e+04, kdiss: 3.63e−02
2232_ID.4164_F5_SLLQFLIGL
KD: 4.39e−07
Kass: 7.65e+04, kdiss: 3.36e−02
2233_ID.4165_K6_SLLQHKIGL
KD: 3.99e−07
Kass: 7.01e+04, kdiss: 2.80e−02
2234_ID.4167_L8_SLLQHLILL
KD: 4.66e−08
Kass: 8.00e+04, kdiss: 3.73e−03
2235_ID.417_H8_SLLQHLIHL
KD: 3.21e−07
Kass: 3.79e+04, kdiss: 1.22e−02
2236_ID.4171_R1_RLLQHLIGL
KD: 1.01e−08
Kass: 1.68e+05, kdiss: 1.70e−03
2237_ID.4172_H4_SLLHHLIGL
KD: 9.71e−09
Kass: 1.45e+05, kdiss: 1.41e−03
2238_ID.4173_S7_SLLQHLSGL
KD: 1.47e−07
Kass: 1.12e+05, kdiss: 1.65e−02
2239_ID.4174_A5_SLLQALIGL
KD: 8.03e−08
Kass: 1.55e+05, kdiss: 1.25e−02
2240_ID.4175_N7_SLLQHLNGL
KD:−
Kass:−, , kdiss: 4.41e−02
2241_ID.4177_K7_SLLQHLKGL
KD: 8.01e−08
Kass: 8.17e+04, kdiss: 6.54e−03
2242_ID.4178_R3_SLRQHLIGL
KD: 3.26e−07
Kass: 1.04e+05, kdiss: 3.41e−02
2243_ID.4179_Y3_SLYQHLIGL
KD: 3.80e−08
Kass: 1.44e+05, kdiss: 5.46e−03
2244_ID.4180_A5_SLLQALIGL
KD: 8.34e−08
Kass: 1.49e+05, kdiss: 1.24e−02
2245_ID.4181_Q1_QLLQHLIGL
KD: 1.44e−08
Kass: 1.45e+05, kdiss: 2.08e−03
2246_ID.4182_L5_SLLQLLIGL
KD: 1.01e−05
Kass: 4.77e+03, kdiss: 4.83e−02
2247_ID.4183_W7_SLLQHLWGL
KD:−
Kass:−, , kdiss: 5.81e−02
2248_ID.4184_T5_SLLQTLIGL
KD:−
Kass:−, , kdiss: 2.87e−02
2249_ID.4187_T6_SLLQHTIGL
KD: 1.17e−08
Kass: 1.42e+05, kdiss: 1.66e−03
2250_ID.4189_P7_SLLQHLPGL
KD: 8.94e−07
Kass: 4.50e+04, kdiss: 4.02e−02
2251_ID.4190_I5_SLLQILIGL
KD:−
Kass:−, , kdiss: 2.14e−02
2252_ID.4191_G3_SLGQHLIGL
KD: 1.02e−08
Kass: 1.84e+05, kdiss: 1.87e−03
2253_ID.4194_P4_SLLPHLIGL
KD: 1.17e−08
Kass: 1.43e+05, kdiss: 1.67e−03
2254_ID.4195_H1_HLLQHLIGL
KD: 1.26e−08
Kass: 1.51e+05, kdiss: 1.91e−03
2255_ID.4196_T5_SLLQTLIGL
KD:−
Kass:−, , kdiss: 5.36e−02
2256_ID.4197_M6_SLLQHMIGL
KD: 8.64e−09
Kass: 1.39e+05, kdiss: 1.20e−03
2257_ID.4199_F6_SLLQHFIGL
KD: 8.48e−09
Kass: 1.31e+05, kdiss: 1.11e−03
2258_ID.42_M5_SLLQMLIGL
KD:−
Kass:−, , kdiss: 5.31e−02
2259_ID.4200_G4_SLLGHLIGL
KD: 8.89e−09
Kass: 1.29e+05, kdiss: 1.14e−03
2260_ID.4201_H8_SLLQHLIHL
KD: 2.39e−07
Kass: 5.17e+04, kdiss: 1.24e−02
2261_ID.4203_Y7_SLLQHLYGL
KD: 3.36e−07
Kass: 6.53e+04, kdiss: 2.19e−02
2262_ID.4204_H4_SLLHHLIGL
KD: 9.56e−09
Kass: 1.34e+05, kdiss: 1.28e−03
2263_ID.4205_H6_SLLQHHIGL
KD: 8.39e−09
Kass: 1.14e+05, kdiss: 9.60e−04
2264_ID.4206_Q3_SLQQHLIGL
KD: 8.71e−09
Kass: 1.53e+05, kdiss: 1.33e−03
2265_ID.4207_H8_SLLQHLIHL
KD: 1.66e−07
Kass: 6.82e+04, kdiss: 1.13e−02
2266_ID.4209_N6_SLLQHNIGL
KD: 9.41e−09
Kass: 1.07e+05, kdiss: 1.00e−03
2267_ID.421_D6_SLLQHDIGL
KD: 1.64e−07
Kass: 4.97e+04, kdiss: 8.13e−03
2268_ID.4210_I6_SLLQHIIGL
KD: 6.05e−09
Kass: 9.80e+04, kdiss: 5.93e−04
2269_ID.4211_A1_ALLQHLIGL
KD: 2.19e−08
Kass: 9.83e+04, kdiss: 2.15e−03
2270_ID.4213_Y8_SLLQHLIYL
KD:−
Kass:−, , kdiss: 2.17e−02
2271_ID.4214_T3_SLTQHLIGL
KD: 1.06e−08
Kass: 1.01e+05, kdiss: 1.07e−03
2272_ID.4215_D1_DLLQHLIGL
KD:−
Kass:−, , kdiss: 8.75e−01
2273_ID.4216_M5_SLLQMLIGL
KD: 1.04e−04
Kass: 2.32e+02, kdiss: 2.42e−02
2274_ID.4217_M6_SLLQHMIGL
KD: 7.52e−09
Kass: 1.00e+05, kdiss: 7.56e−04
2275_ID.4218_T4_SLLTHLIGL
KD: 5.06e−09
Kass: 1.08e+05, kdiss: 5.49e−04
2276_ID.4219_H7_SLLQHLHGL
KD: 6.73e−08
Kass: 3.95e+04, kdiss: 2.66e−03
2277_ID.4220_S3_SLSQHLIGL
KD:−
Kass:−, , kdiss: 3.39e−02
2278_ID.4221_S8_SLLQHLISL
KD: 5.94e−09
Kass: 9.63e+04, kdiss: 5.72e−04
2279_ID.4223_S8_SLLQHLISL
KD: 1.15e−08
Kass: 1.01e+05, kdiss: 1.17e−03
2280_ID.4225_H7_SLLQHLHGL
KD: 7.40e−08
Kass: 4.10e+04, kdiss: 3.04e−03
2281_ID.4226_E5_SLLQELIGL
KD:−
Kass:−, , kdiss: 3.70e−02
2282_ID.4227_E7_SLLQHLEGL
KD:−
Kass:−, , kdiss: 2.44e−01
2283_ID.4228_W4_SLLWHLIGL
KD: 1.15e−08
Kass: 1.14e+05, kdiss: 1.31e−03
2284_ID.423_M8_SLLQHLIML
KD: 8.26e−08
Kass: 6.29e+04, kdiss: 5.20e−03
2285_ID.4230_D5_SLLQDLIGL
KD: 1.47e−07
Kass: 7.37e+04, kdiss: 1.08e−02
2286_ID.4231_Y6_SLLQHYIGL
KD: 7.97e−09
Kass: 1.23e+05, kdiss: 9.80e−04
2287_ID.4232_D5_SLLQDLIGL
KD: 1.32e−07
Kass: 7.49e+04, kdiss: 9.90e−03
2288_ID.4233_N7_SLLQHLNGL
KD:−
Kass:−, , kdiss: 2.82e−02
2289_ID.4235_I3_SLIQHLIGL
KD: 9.54e−09
Kass: 1.10e+05, kdiss: 1.05e−03
2290_ID.4239_F6_SLLQHFIGL
KD: 7.16e−09
Kass: 1.29e+05, kdiss: 9.25e−04
2291_ID.424_P4_SLLPHLIGL
KD: 1.04e−08
Kass: 1.36e+05, kdiss: 1.42e−03
2292_ID.4241_D1_DLLQHLIGL
KD: 1.33e−08
Kass: 1.56e+05, kdiss: 2.07e−03
2293_ID.4242_A5_SLLQALIGL
KD: 7.99e−08
Kass: 1.44e+05, kdiss: 1.15e−02
2294_ID.4244_M4_SLLMHLIGL
KD: 1.14e−08
Kass: 1.51e+05, kdiss: 1.72e−03
2295_ID.4245_S7_SLLQHLSGL
KD: 1.68e−07
Kass: 1.00e+05, kdiss: 1.69e−02
2296_ID.4247_W6_SLLQHWIGL
KD: 8.60e−09
Kass: 1.42e+05, kdiss: 1.22e−03
2297_ID.4249_G3_SLGQHLIGL
KD: 8.56e−09
Kass: 1.87e+05, kdiss: 1.60e−03
2298_ID.425_W1_WLLQHLIGL
KD: 1.11e−08
Kass: 1.43e+05, kdiss: 1.59e−03
2299_ID.4250_G6_SLLQHGIGL
KD: 6.35e−09
Kass: 1.78e+05, kdiss: 1.13e−03
2300_ID.4253_WT_SLLQHLIGL
KD: 1.17e−08
Kass: 1.49e+05, kdiss: 1.74e−03
value
15000
10000
5000
0
0
250
500
750
1000
Time

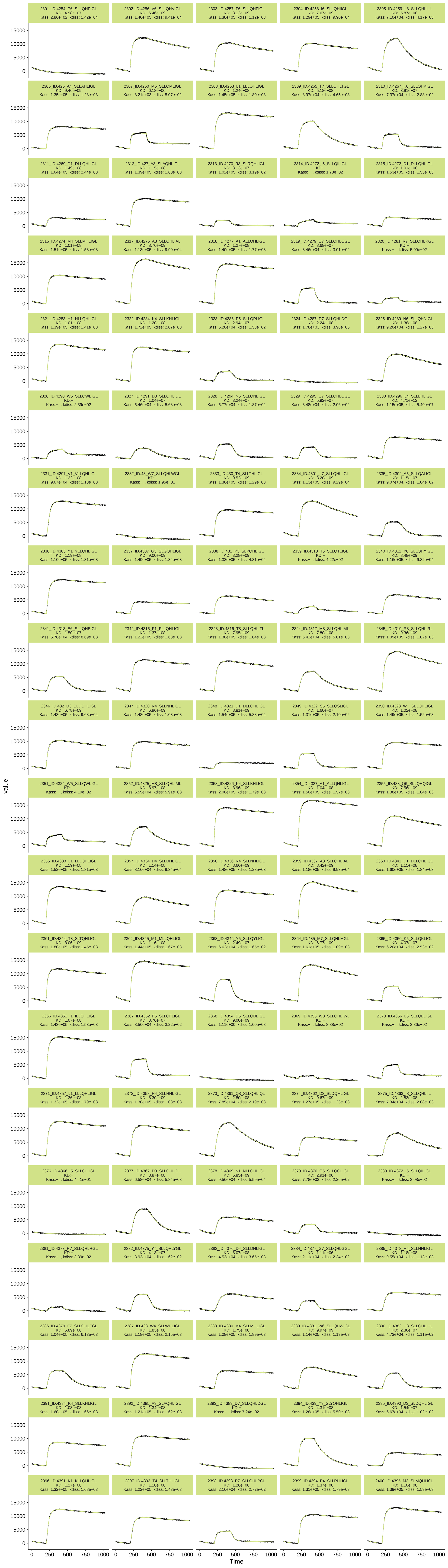
2301_ID.4254_P6_SLLQHPIGL
KD: 4.98e−07
Kass: 2.86e+02, kdiss: 1.42e−04
2302_ID.4256_V6_SLLQHVIGL
KD: 6.46e−09
Kass: 1.46e+05, kdiss: 9.41e−04
2303_ID.4257_F6_SLLQHFIGL
KD: 8.13e−09
Kass: 1.38e+05, kdiss: 1.12e−03
2304_ID.4258_I6_SLLQHIIGL
KD: 7.67e−09
Kass: 1.29e+05, kdiss: 9.90e−04
2305_ID.4259_L8_SLLQHLILL
KD: 5.87e−08
Kass: 7.10e+04, kdiss: 4.17e−03
2306_ID.426_A4_SLLAHLIGL
KD: 9.46e−09
Kass: 1.35e+05, kdiss: 1.28e−03
2307_ID.4260_M5_SLLQMLIGL
KD: 6.18e−06
Kass: 8.21e+03, kdiss: 5.07e−02
2308_ID.4263_L1_LLLQHLIGL
KD: 1.24e−08
Kass: 1.45e+05, kdiss: 1.80e−03
2309_ID.4265_T7_SLLQHLTGL
KD: 5.18e−08
Kass: 8.97e+04, kdiss: 4.65e−03
2310_ID.4267_K6_SLLQHKIGL
KD: 3.91e−07
Kass: 7.37e+04, kdiss: 2.88e−02
2311_ID.4269_D1_DLLQHLIGL
KD: 1.49e−08
Kass: 1.64e+05, kdiss: 2.44e−03
2312_ID.427_A3_SLAQHLIGL
KD: 1.15e−08
Kass: 1.39e+05, kdiss: 1.60e−03
2313_ID.4270_R3_SLRQHLIGL
KD: 3.13e−07
Kass: 1.02e+05, kdiss: 3.19e−02
2314_ID.4272_I5_SLLQILIGL
KD:−
Kass:−, , kdiss: 1.78e−02
2315_ID.4273_D1_DLLQHLIGL
KD: 1.01e−08
Kass: 1.53e+05, kdiss: 1.55e−03
2316_ID.4274_M4_SLLMHLIGL
KD: 1.01e−08
Kass: 1.51e+05, kdiss: 1.53e−03
2317_ID.4275_A8_SLLQHLIAL
KD: 8.76e−09
Kass: 1.13e+05, kdiss: 9.90e−04
2318_ID.4277_A1_ALLQHLIGL
KD: 1.27e−08
Kass: 1.40e+05, kdiss: 1.77e−03
2319_ID.4279_Q7_SLLQHLQGL
KD: 8.68e−07
Kass: 3.46e+04, kdiss: 3.01e−02
2320_ID.4281_R7_SLLQHLRGL
KD:−
Kass:−, , kdiss: 5.09e−02
2321_ID.4283_H1_HLLQHLIGL
KD: 1.01e−08
Kass: 1.39e+05, kdiss: 1.41e−03
2322_ID.4284_K4_SLLKHLIGL
KD: 1.20e−08
Kass: 1.72e+05, kdiss: 2.07e−03
2323_ID.4286_P5_SLLQPLIGL
KD: 2.94e−07
Kass: 5.20e+04, kdiss: 1.53e−02
2324_ID.4287_D7_SLLQHLDGL
KD: 2.24e−08
Kass: 1.78e+03, kdiss: 3.98e−05
2325_ID.4289_N6_SLLQHNIGL
KD: 1.38e−08
Kass: 9.20e+04, kdiss: 1.27e−03
2326_ID.4290_W5_SLLQWLIGL
KD:−
Kass:−, , kdiss: 2.39e−02
2327_ID.4291_D8_SLLQHLIDL
KD: 1.04e−07
Kass: 5.46e+04, kdiss: 5.68e−03
2328_ID.4294_N5_SLLQNLIGL
KD: 3.24e−07
Kass: 5.77e+04, kdiss: 1.87e−02
2329_ID.4295_Q7_SLLQHLQGL
KD: 5.92e−07
Kass: 3.48e+04, kdiss: 2.06e−02
2330_ID.4296_L4_SLLLHLIGL
KD: 4.71e−12
Kass: 1.15e+05, kdiss: 5.40e−07
2331_ID.4297_V1_VLLQHLIGL
KD: 1.22e−08
Kass: 9.67e+04, kdiss: 1.18e−03
2332_ID.43_W7_SLLQHLWGL
KD:−
Kass:−, , kdiss: 1.95e−01
2333_ID.430_T4_SLLTHLIGL
KD: 9.52e−09
Kass: 1.36e+05, kdiss: 1.29e−03
2334_ID.4301_L7_SLLQHLLGL
KD: 8.20e−09
Kass: 1.13e+05, kdiss: 9.29e−04
2335_ID.4302_A5_SLLQALIGL
KD: 1.15e−07
Kass: 9.07e+04, kdiss: 1.04e−02
2336_ID.4303_Y1_YLLQHLIGL
KD: 1.19e−08
Kass: 1.10e+05, kdiss: 1.31e−03
2337_ID.4307_G3_SLGQHLIGL
KD: 9.00e−09
Kass: 1.49e+05, kdiss: 1.34e−03
2338_ID.431_P3_SLPQHLIGL
KD: 3.28e−09
Kass: 1.32e+05, kdiss: 4.31e−04
2339_ID.4310_T5_SLLQTLIGL
KD:−
Kass:−, , kdiss: 4.22e−02
2340_ID.4311_Y6_SLLQHYIGL
KD: 8.48e−09
Kass: 1.16e+05, kdiss: 9.82e−04
2341_ID.4313_E6_SLLQHEIGL
KD: 1.50e−07
Kass: 5.78e+04, kdiss: 8.69e−03
2342_ID.4315_F1_FLLQHLIGL
KD: 1.37e−08
Kass: 1.22e+05, kdiss: 1.68e−03
2343_ID.4316_T8_SLLQHLITL
KD: 7.95e−09
Kass: 1.30e+05, kdiss: 1.04e−03
2344_ID.4317_M8_SLLQHLIML
KD: 7.80e−08
Kass: 6.42e+04, kdiss: 5.01e−03
2345_ID.4319_R8_SLLQHLIRL
KD: 9.36e−09
Kass: 1.09e+05, kdiss: 1.02e−03
2346_ID.432_D3_SLDQHLIGL
KD: 6.78e−09
Kass: 1.43e+05, kdiss: 9.68e−04
2347_ID.4320_N4_SLLNHLIGL
KD: 6.96e−09
Kass: 1.48e+05, kdiss: 1.03e−03
2348_ID.4321_D1_DLLQHLIGL
KD: 3.81e−09
Kass: 1.54e+05, kdiss: 5.88e−04
2349_ID.4322_S5_SLLQSLIGL
KD: 1.60e−07
Kass: 1.31e+05, kdiss: 2.10e−02
2350_ID.4323_WT_SLLQHLIGL
KD: 1.02e−08
Kass: 1.49e+05, kdiss: 1.52e−03
2351_ID.4324_W5_SLLQWLIGL
KD:−
Kass:−, , kdiss: 4.10e−02
2352_ID.4325_M8_SLLQHLIML
KD: 8.97e−08
Kass: 6.59e+04, kdiss: 5.91e−03
2353_ID.4326_K4_SLLKHLIGL
KD: 8.96e−09
Kass: 2.00e+05, kdiss: 1.79e−03
2354_ID.4327_A1_ALLQHLIGL
KD: 1.04e−08
Kass: 1.50e+05, kdiss: 1.57e−03
2355_ID.433_Q6_SLLQHQIGL
KD: 7.56e−09
Kass: 1.38e+05, kdiss: 1.04e−03
2356_ID.4333_L1_LLLQHLIGL
KD: 1.19e−08
Kass: 1.52e+05, kdiss: 1.81e−03
2357_ID.4334_D4_SLLDHLIGL
KD: 1.14e−08
Kass: 8.16e+04, kdiss: 9.34e−04
2358_ID.4336_N4_SLLNHLIGL
KD: 8.66e−09
Kass: 1.48e+05, kdiss: 1.28e−03
2359_ID.4337_A8_SLLQHLIAL
KD: 8.42e−09
Kass: 1.18e+05, kdiss: 9.93e−04
2360_ID.4341_D1_DLLQHLIGL
KD: 1.15e−08
Kass: 1.60e+05, kdiss: 1.84e−03
2361_ID.4344_T3_SLTQHLIGL
KD: 8.06e−09
Kass: 1.80e+05, kdiss: 1.45e−03
2362_ID.4345_M1_MLLQHLIGL
KD: 1.16e−08
Kass: 1.44e+05, kdiss: 1.67e−03
2363_ID.4346_Y5_SLLQYLIGL
KD: 2.49e−07
Kass: 6.63e+04, kdiss: 1.65e−02
2364_ID.435_M7_SLLQHLMGL
KD: 6.77e−09
Kass: 1.61e+05, kdiss: 1.09e−03
2365_ID.4350_K5_SLLQKLIGL
KD: 4.07e−07
Kass: 6.20e+04, kdiss: 2.53e−02
2366_ID.4351_I1_ILLQHLIGL
KD: 1.07e−08
Kass: 1.43e+05, kdiss: 1.53e−03
2367_ID.4352_F5_SLLQFLIGL
KD: 3.76e−07
Kass: 8.56e+04, kdiss: 3.22e−02
2368_ID.4354_D5_SLLQDLIGL
KD: 9.00e−09
Kass: 1.11e+00, kdiss: 1.00e−08
2369_ID.4355_W8_SLLQHLIWL
KD:−
Kass:−, , kdiss: 8.88e−02
2370_ID.4356_L5_SLLQLLIGL
KD:−
Kass:−, , kdiss: 3.86e−02
2371_ID.4357_L1_LLLQHLIGL
KD: 1.36e−08
Kass: 1.32e+05, kdiss: 1.79e−03
2372_ID.4358_H4_SLLHHLIGL
KD: 8.30e−09
Kass: 1.30e+05, kdiss: 1.08e−03
2373_ID.4361_Q8_SLLQHLIQL
KD: 2.80e−08
Kass: 7.85e+04, kdiss: 2.19e−03
2374_ID.4362_D3_SLDQHLIGL
KD: 9.67e−09
Kass: 1.27e+05, kdiss: 1.23e−03
2375_ID.4363_I8_SLLQHLIIL
KD: 2.83e−08
Kass: 7.34e+04, kdiss: 2.08e−03
2376_ID.4366_I5_SLLQILIGL
KD:−
Kass:−, , kdiss: 4.41e−01
2377_ID.4367_D8_SLLQHLIDL
KD: 8.87e−08
Kass: 6.58e+04, kdiss: 5.84e−03
2378_ID.4369_N1_NLLQHLIGL
KD: 5.85e−09
Kass: 9.56e+04, kdiss: 5.59e−04
2379_ID.4370_G5_SLLQGLIGL
KD: 2.91e−06
Kass: 7.78e+03, kdiss: 2.26e−02
2380_ID.4372_I5_SLLQILIGL
KD:−
Kass:−, , kdiss: 3.08e−02
2381_ID.4373_R7_SLLQHLRGL
KD:−
Kass:−, , kdiss: 3.39e−02
2382_ID.4375_Y7_SLLQHLYGL
KD: 4.13e−07
Kass: 3.93e+04, kdiss: 1.62e−02
2383_ID.4376_D4_SLLDHLIGL
KD: 8.07e−08
Kass: 4.53e+04, kdiss: 3.65e−03
2384_ID.4377_G7_SLLQHLGGL
KD: 1.11e−06
Kass: 2.11e+04, kdiss: 2.34e−02
2385_ID.4378_H4_SLLHHLIGL
KD: 1.18e−08
Kass: 9.55e+04, kdiss: 1.13e−03
2386_ID.4379_F7_SLLQHLFGL
KD: 5.89e−08
Kass: 1.04e+05, kdiss: 6.13e−03
2387_ID.438_W4_SLLWHLIGL
KD: 1.83e−08
Kass: 1.18e+05, kdiss: 2.15e−03
2388_ID.4380_M4_SLLMHLIGL
KD: 1.75e−08
Kass: 1.08e+05, kdiss: 1.89e−03
2389_ID.4381_W6_SLLQHWIGL
KD: 9.97e−09
Kass: 1.14e+05, kdiss: 1.13e−03
2390_ID.4383_H8_SLLQHLIHL
KD: 2.36e−07
Kass: 4.73e+04, kdiss: 1.11e−02
2391_ID.4384_K4_SLLKHLIGL
KD: 1.03e−08
Kass: 1.60e+05, kdiss: 1.66e−03
2392_ID.4385_A3_SLAQHLIGL
KD: 1.34e−08
Kass: 1.21e+05, kdiss: 1.62e−03
2393_ID.4389_D7_SLLQHLDGL
KD:−
Kass:−, , kdiss: 7.24e−02
2394_ID.439_Y3_SLYQHLIGL
KD: 4.31e−08
Kass: 1.28e+05, kdiss: 5.50e−03
2395_ID.4390_D3_SLDQHLIGL
KD: 1.54e−07
Kass: 6.67e+04, kdiss: 1.02e−02
2396_ID.4391_K1_KLLQHLIGL
KD: 1.27e−08
Kass: 1.32e+05, kdiss: 1.68e−03
2397_ID.4392_T4_SLLTHLIGL
KD: 1.18e−08
Kass: 1.22e+05, kdiss: 1.43e−03
2398_ID.4393_P7_SLLQHLPGL
KD: 1.26e−06
Kass: 2.16e+04, kdiss: 2.72e−02
2399_ID.4394_P4_SLLPHLIGL
KD: 1.37e−08
Kass: 1.31e+05, kdiss: 1.79e−03
2400_ID.4395_M3_SLMQHLIGL
KD: 1.10e−08
Kass: 1.39e+05, kdiss: 1.53e−03
value
0
5000
10000
15000
Time
0
250
500
750
1000

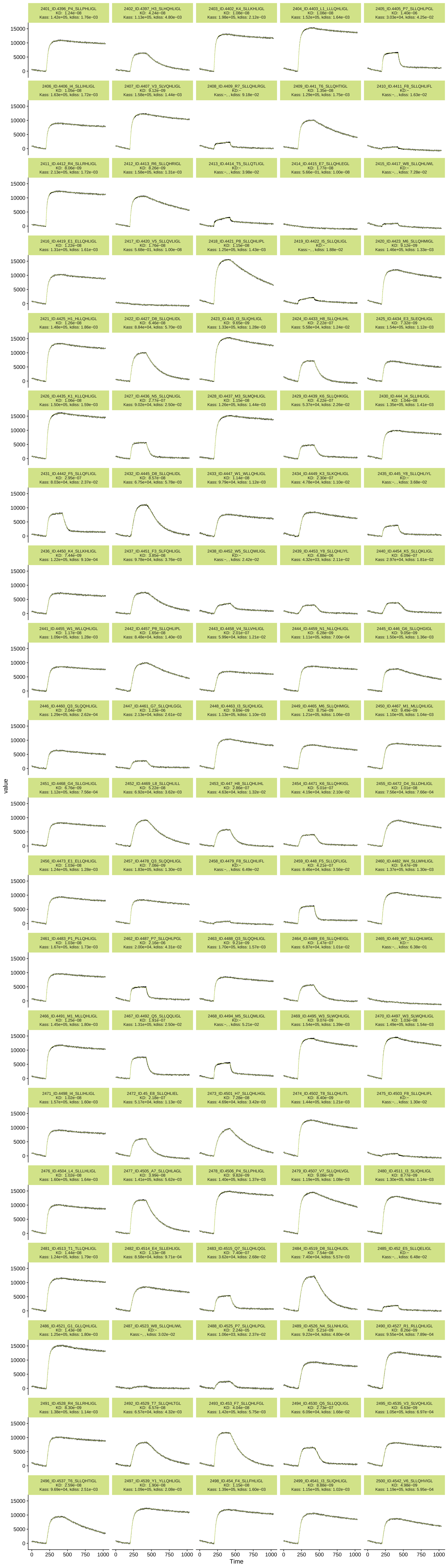

value
Time
0
5000
10000
15000
0
250
500
750
1000

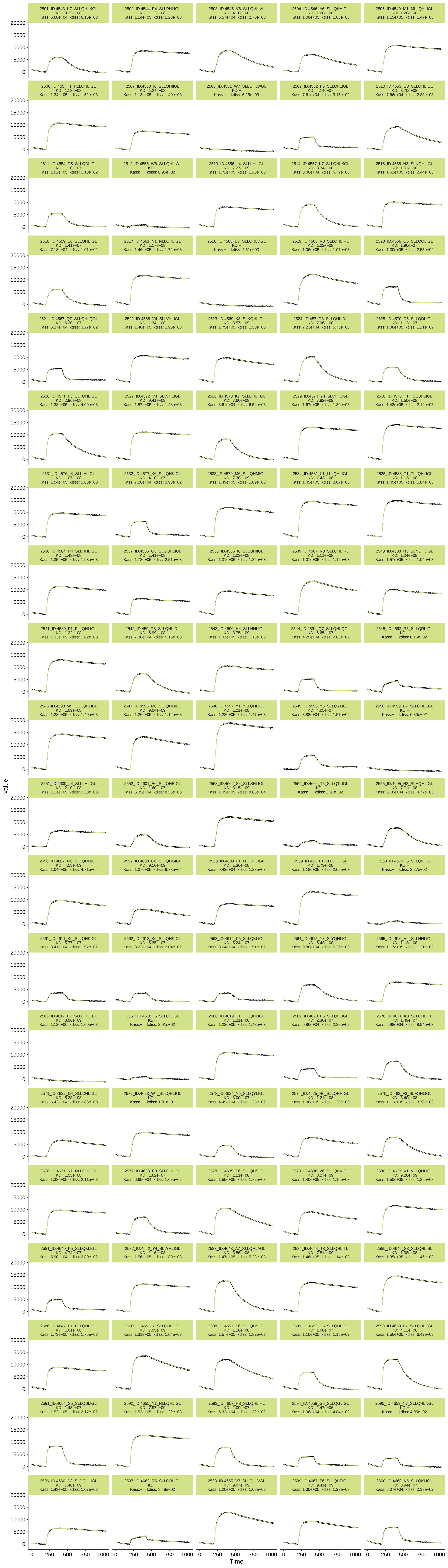
2501_ID.4543_K7_SLLQHLKGL KD: 9.22e−08 Kass: 6.68e+04, kdiss: 6.16e−03
2502_ID.4544_P4_SLLPHLIGL KD: 1.12e−08 Kass: 1.14e+05, kdiss: 1.28e−03
2503_ID.4545_V8_SLLQHLIVL KD: 4.10e−08 Kass: 6.57e+04, kdiss: 2.70e−03
2504_ID.4546_A6_SLLQHAIGL KD: 1.68e−08 Kass: 1.09e+05, kdiss: 1.83e−03
2505_ID.4549_M1_MLLQHLIGL KD: 1.28e−08 Kass: 1.15e+05, kdiss: 1.47e−03
2506_ID.455_H1_HLLQHLIGL KD: 1.13e−08 Kass: 1.34e+05, kdiss: 1.52e−03
2507_ID.4550_I6_SLLQHIIGL KD: 1.24e−08 Kass: 1.13e+05, kdiss: 1.40e−03
2508_ID.4551_W7_SLLQHLWGL KD:− Kass:−, kdiss: 6.25e−03
2509_ID.4552_F5_SLLQFLIGL KD: 4.14e−07 Kass: 7.61e+04, kdiss: 3.15e−02
2510_ID.4553_Q8_SLLQHLIQL KD: 3.70e−08 Kass: 7.66e+04, kdiss: 2.83e−03
2511_ID.4554_D5_SLLQDLIGL KD: 1.10e−07 Kass: 1.02e+05, kdiss: 1.13e−02
2512_ID.4555_W8_SLLQHLIWL KD:− Kass:−, kdiss: 8.95e−02
2513_ID.4556_L4_SLLLHLIGL KD: 7.27e−09 Kass: 1.72e+05, kdiss: 1.25e−03
2514_ID.4557_K7_SLLQHLKGL KD: 8.34e−08 Kass: 8.06e+04, kdiss: 6.72e−03
2515_ID.4558_N3_SLNQHLIGL KD: 1.51e−08 Kass: 1.62e+05, kdiss: 2.44e−03
2516_ID.4559_E6_SLLQHEIGL KD: 1.41e−07 Kass: 7.18e+04, kdiss: 1.01e−02
2517_ID.4561_N1_NLLQHLIGL KD: 1.17e−08 Kass: 1.46e+05, kdiss: 1.72e−03
2518_ID.4563_D7_SLLQHLDGL KD:− Kass:−, kdiss: 4.51e−03
2519_ID.4565_R8_SLLQHLIRL KD: 1.02e−08 Kass: 1.06e+05, kdiss: 1.07e−03
2520_ID.4566_Q5_SLLQQLIGL KD: 1.96e−07 Kass: 1.30e+05, kdiss: 2.55e−02
2521_ID.4567_Q7_SLLQHLQGL KD: 6.20e−07 Kass: 5.27e+04, kdiss: 3.27e−02
2522_ID.4568_V4_SLLVHLIGL KD: 1.34e−08 Kass: 1.46e+05, kdiss: 1.95e−03
2523_ID.4569_K3_SLKQHLIGL KD: 8.57e−09 Kass: 1.75e+05, kdiss: 1.50e−03
2524_ID.457_D8_SLLQHLIDL KD: 7.96e−08 Kass: 7.23e+04, kdiss: 5.75e−03
2525_ID.4570_D5_SLLQDLIGL KD: 1.12e−07 Kass: 1.08e+05, kdiss: 1.21e−02
2526_ID.4571_F3_SLFQHLIGL KD: 2.96e−08 Kass: 1.38e+05, kdiss: 4.09e−03
2527_ID.4572_V4_SLLVHLIGL KD: 9.41e−09 Kass: 1.57e+05, kdiss: 1.48e−03
2528_ID.4573_K7_SLLQHLKGL KD: 7.60e−08 Kass: 8.61e+04, kdiss: 6.54e−03
2529_ID.4574_Y4_SLLYHLIGL KD: 7.82e−09 Kass: 1.67e+05, kdiss: 1.30e−03
2530_ID.4575_T1_TLLQHLIGL KD: 1.50e−08 Kass: 1.42e+05, kdiss: 2.14e−03
2531_ID.4576_I4_SLLIHLIGL KD: 1.07e−08 Kass: 1.54e+05, kdiss: 1.65e−03
2532_ID.4577_K6_SLLQHKIGL KD: 4.10e−07 Kass: 7.28e+04, kdiss: 2.98e−02
2533_ID.4579_M6_SLLQHMIGL KD: 7.30e−09 Kass: 1.49e+05, kdiss: 1.09e−03
2534_ID.4581_L1_LLLQHLIGL KD: 1.43e−08 Kass: 1.45e+05, kdiss: 2.07e−03
2535_ID.4583_T1_TLLQHLIGL KD: 1.13e−08 Kass: 1.45e+05, kdiss: 1.64e−03
2536_ID.4584_H4_SLLHHLIGL KD: 1.43e−08 Kass: 1.35e+05, kdiss: 1.93e−03
2537_ID.4585_G3_SLGQHLIGL KD: 1.41e−08 Kass: 1.78e+05, kdiss: 2.51e−03
2538_ID.4586_I6_SLLQHIIGL KD: 1.03e−08 Kass: 1.31e+05, kdiss: 1.34e−03
2539_ID.4587_R8_SLLQHLIRL KD: 1.11e−08 Kass: 1.01e+05, kdiss: 1.12e−03
2540_ID.4588_N3_SLNQHLIGL KD: 1.24e−08 Kass: 1.57e+05, kdiss: 1.94e−03
2541_ID.4589_F1_FLLQHLIGL KD: 1.22e−08 Kass: 1.33e+05, kdiss: 1.62e−03
2542_ID.459_D8_SLLQHLIDL KD: 6.99e−08 Kass: 7.38e+04, kdiss: 5.15e−03
2543_ID.4590_H4_SLLHHLIGL KD: 8.75e−09 Kass: 1.31e+05, kdiss: 1.15e−03
2544_ID.4591_Q7_SLLQHLQGL KD: 6.65e−07 Kass: 4.25e+04, kdiss: 2.83e−02
2545_ID.4592_R5_SLLQRLIGL KD:− Kass:−, kdiss: 6.16e−02
2546_ID.4593_WT_SLLQHLIGL KD: 1.09e−08 Kass: 1.28e+05, kdiss: 1.40e−03
2547_ID.4595_M6_SLLQHMIGL KD: 9.04e−09 Kass: 1.26e+05, kdiss: 1.14e−03
2548_ID.4597_Y1_YLLQHLIGL KD: 1.21e−08 Kass: 1.22e+05, kdiss: 1.47e−03
2549_ID.4598_Y5_SLLQYLIGL KD: 4.05e−07 Kass: 3.88e+04, kdiss: 1.57e−02
2550_ID.4599_E7_SLLQHLEGL KD:− Kass:−, kdiss: 6.80e−02
2551_ID.4600_L4_SLLLHLIGL KD: 2.10e−08 Kass: 1.11e+05, kdiss: 2.33e−03
2552_ID.4601_E6_SLLQHEIGL KD: 1.60e−07 Kass: 5.35e+04, kdiss: 8.56e−03
2553_ID.4602_S4_SLLSHLIGL KD: 8.15e−09 Kass: 1.09e+05, kdiss: 8.85e−04
2554_ID.4604_T5_SLLQTLIGL KD:− Kass:−, kdiss: 2.81e−02
2555_ID.4605_H3_SLHQHLIGL KD: 7.71e−08 Kass: 6.18e+04, kdiss: 4.77e−03
2556_ID.4607_M6_SLLQHMIGL KD: 4.53e−09 Kass: 1.04e+05, kdiss: 4.71e−04
2557_ID.4608_G6_SLLQHGIGL KD: 9.16e−09 Kass: 1.07e+05, kdiss: 9.78e−04
2558_ID.4609_L1_LLLQHLIGL KD: 1.36e−08 Kass: 9.42e+04, kdiss: 1.28e−03
2559_ID.461_L1_LLLQHLIGL KD: 1.73e−08 Kass: 1.18e+05, kdiss: 2.05e−03
2560_ID.4610_I5_SLLQILIGL KD:− Kass:−, kdiss: 2.27e−02
2561_ID.4611_K6_SLLQHKIGL KD: 5.77e−07 Kass: 3.41e+04, kdiss: 1.97e−02
2562_ID.4613_K6_SLLQHKIGL KD: 6.35e−07 Kass: 3.22e+04, kdiss: 2.04e−02
2563_ID.4614_K5_SLLQKLIGL KD: 5.24e−07 Kass: 3.64e+04, kdiss: 1.91e−02
2564_ID.4615_Y3_SLYQHLIGL KD: 5.43e−08 Kass: 9.88e+04, kdiss: 5.36e−03
2565_ID.4616_H4_SLLHHLIGL KD: 1.12e−08 Kass: 1.17e+05, kdiss: 1.31e−03
2566_ID.4617_E7_SLLQHLEGL KD: 8.96e−09 Kass: 1.12e+00, kdiss: 1.00e−08
2567_ID.4618_I5_SLLQILIGL KD:− Kass:−, kdiss: 2.91e−02
2568_ID.4619_T1_TLLQHLIGL KD: 1.21e−08 Kass: 1.23e+05, kdiss: 1.48e−03
2569_ID.4620_F5_SLLQFLIGL KD: 2.36e−07 Kass: 9.86e+04, kdiss: 2.32e−02
2570_ID.4621_K8_SLLQHLIKL KD: 1.49e−07 Kass: 5.98e+04, kdiss: 8.94e−03
2571_ID.4622_D4_SLLDHLIGL KD: 5.28e−08 Kass: 5.42e+04, kdiss: 2.86e−03
2572_ID.4623_WT_SLLQHLIGL KD:− Kass:−, kdiss: 1.91e−01
2573_ID.4624_Y5_SLLQYLIGL KD: 3.00e−07 Kass: 4.49e+04, kdiss: 1.35e−02
2574_ID.4625_H6_SLLQHHIGL KD: 1.21e−08 Kass: 1.06e+05, kdiss: 1.28e−03
2575_ID.463_F3_SLFQHLIGL KD: 3.40e−08 Kass: 1.11e+05, kdiss: 3.78e−03
2576_ID.4631_N1_NLLQHLIGL KD: 1.53e−08 Kass: 1.38e+05, kdiss: 2.11e−03
2577_ID.4633_E8_SLLQHLIEL KD: 1.62e−07 Kass: 6.65e+04, kdiss: 1.08e−02
2578_ID.4635_S6_SLLQHSIGL KD: 1.11e−08 Kass: 1.55e+05, kdiss: 1.72e−03
2579_ID.4636_V6_SLLQHVIGL KD: 8.27e−09 Kass: 1.40e+05, kdiss: 1.16e−03
2580_ID.4637_V1_VLLQHLIGL KD: 9.26e−09 Kass: 1.50e+05, kdiss: 1.39e−03
2581_ID.4640_K5_SLLQKLIGL KD: 4.74e−07 Kass: 5.90e+04, kdiss: 2.80e−02
2582_ID.4642_Y4_SLLYHLIGL KD: 1.16e−08 Kass: 1.56e+05, kdiss: 1.80e−03
2583_ID.4643_A7_SLLQHLAGL KD: 3.56e−08 Kass: 1.47e+05, kdiss: 5.23e−03
2584_ID.4644_T8_SLLQHLITL KD: 7.81e−09 Kass: 1.46e+05, kdiss: 1.14e−03
2585_ID.4645_S8_SLLQHLISL KD: 1.08e−08 Kass: 1.35e+05, kdiss: 1.46e−03
2586_ID.4647_P1_PLLQHLIGL KD: 1.01e−08 Kass: 1.73e+05, kdiss: 1.75e−03
2587_ID.465_L7_SLLQHLLGL KD: 7.95e−09 Kass: 1.31e+05, kdiss: 1.04e−03
2588_ID.4651_S6_SLLQHSIGL KD: 1.16e−08 Kass: 1.57e+05, kdiss: 1.82e−03
2589_ID.4652_D5_SLLQDLIGL KD: 1.06e−07 Kass: 1.10e+05, kdiss: 1.16e−02
2590_ID.4653_F7_SLLQHLFGL KD: 4.12e−08 Kass: 1.56e+05, kdiss: 6.42e−03
2591_ID.4654_S5_SLLQSLIGL KD: 1.43e−07 Kass: 1.52e+05, kdiss: 2.17e−02
2592_ID.4655_N1_NLLQHLIGL KD: 7.97e−09 Kass: 1.53e+05, kdiss: 1.22e−03
2593_ID.4657_H8_SLLQHLIHL KD: 2.09e−07 Kass: 6.32e+04, kdiss: 1.32e−02
2594_ID.4658_G5_SLLQGLIGL KD: 2.47e−06 Kass: 1.96e+04, kdiss: 4.84e−02
2595_ID.4659_N7_SLLQHLNGL KD:− Kass:−, kdiss: 4.05e−02
2596_ID.4660_D3_SLDQHLIGL KD: 7.48e−09 Kass: 1.43e+05, kdiss: 1.07e−03
2597_ID.4662_R5_SLLQRLIGL KD:− Kass:−, kdiss: 8.48e−02
2598_ID.4665_V7_SLLQHLVGL KD: 9.07e−09 Kass: 1.20e+05, kdiss: 1.08e−03
2599_ID.4667_F6_SLLQHFIGL KD: 9.41e−09 Kass: 1.30e+05, kdiss: 1.23e−03
2600_ID.4668_K5_SLLQKLIGL KD: 3.94e−07 Kass: 6.07e+04, kdiss: 2.39e−02
value
Time
0 5000 10000 15000 20000
0 250 500 750 1000

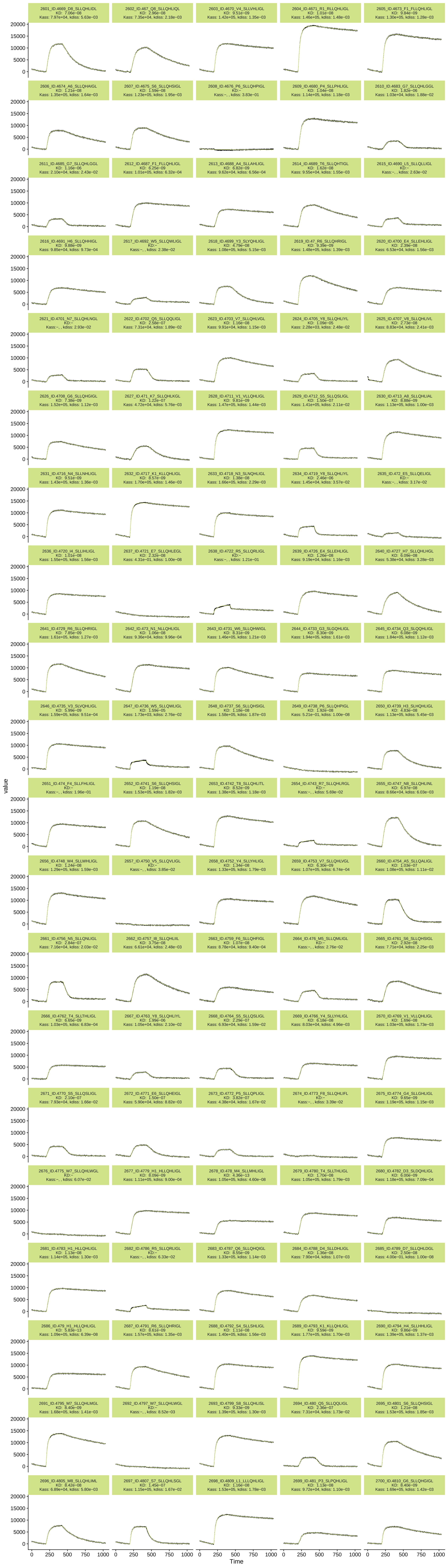

2601_ID.4669_D8_SLLQHLIDL KD: 7.06e−08 Kass: 7.97e+04, kdiss: 5.63e−03
2602_ID.467_Q8_SLLQHLIQL KD: 2.96e−08 Kass: 7.35e+04, kdiss: 2.18e−03
2603_ID.4670_V4_SLLVHLIGL KD: 9.51e−09 Kass: 1.42e+05, kdiss: 1.35e−03
2604_ID.4671_R1_RLLQHLIGL KD: 1.01e−08 Kass: 1.46e+05, kdiss: 1.48e−03
2605_ID.4673_F1_FLLQHLIGL KD: 9.84e−09 Kass: 1.30e+05, kdiss: 1.28e−03
2606_ID.4674_A6_SLLQHAIGL KD: 1.21e−08 Kass: 1.35e+05, kdiss: 1.64e−03
2607_ID.4675_S6_SLLQHSIGL KD: 1.59e−08 Kass: 1.23e+05, kdiss: 1.95e−03
2608_ID.4676_P6_SLLQHPIGL KD:~ Kass:~, , kdiss: 3.83e−01
2609_ID.4680_P4_SLLPHLIGL KD: 1.04e−08 Kass: 1.14e+05, kdiss: 1.18e−03
2610_ID.4683_G7_SLLQHLGGL KD: 1.82e−06 Kass: 1.03e+04, kdiss: 1.88e−02
2611_ID.4685_G7_SLLQHLGGL KD: 1.16e−06 Kass: 2.10e+04, kdiss: 2.43e−02
2612_ID.4687_F1_FLLQHLIGL KD: 6.25e−09 Kass: 1.01e+05, kdiss: 6.32e−04
2613_ID.4688_A4_SLLAHLIGL KD: 6.82e−09 Kass: 9.62e+04, kdiss: 6.56e−04
2614_ID.4689_T6_SLLQHTIGL KD: 1.62e−08 Kass: 9.55e+04, kdiss: 1.55e−03
2615_ID.4690_L5_SLLQLLIGL KD:~ Kass:~, , kdiss: 2.63e−02
2616_ID.4691_H6_SLLQHHIGL KD: 9.88e−09 Kass: 9.85e+04, kdiss: 9.73e−04
2617_ID.4692_W5_SLLQWLIGL KD:~ Kass:~, , kdiss: 2.38e−02
2618_ID.4699_Y3_SLYQHLIGL KD: 4.79e−08 Kass: 1.08e+05, kdiss: 5.15e−03
2619_ID.47_R6_SLLQHRIGL KD: 9.39e−09 Kass: 1.48e+05, kdiss: 1.39e−03
2620_ID.4700_E4_SLLEHLIGL KD: 2.39e−08 Kass: 6.53e+04, kdiss: 1.56e−03
2621_ID.4701_N7_SLLQHLNGL KD:~ Kass:~, , kdiss: 2.93e−02
2622_ID.4702_Q5_SLLQQLIGL KD: 2.58e−07 Kass: 7.31e+04, kdiss: 1.89e−02
2623_ID.4703_V7_SLLQHLVGL KD: 1.16e−08 Kass: 9.91e+04, kdiss: 1.15e−03
2624_ID.4705_Y8_SLLQHLIYL KD: 1.09e−05 Kass: 2.28e+03, kdiss: 2.48e−02
2625_ID.4707_V8_SLLQHLIVL KD: 2.73e−08 Kass: 8.83e+04, kdiss: 2.41e−03
2626_ID.4708_G6_SLLQHGIGL KD: 7.38e−09 Kass: 1.52e+05, kdiss: 1.12e−03
2627_ID.471_K7_SLLQHLKGL KD: 1.22e−07 Kass: 4.72e+04, kdiss: 5.76e−03
2628_ID.4711_V1_VLLQHLIGL KD: 9.81e−09 Kass: 1.47e+05, kdiss: 1.44e−03
2629_ID.4712_S5_SLLQSLIGL KD: 1.50e−07 Kass: 1.41e+05, kdiss: 2.11e−02
2630_ID.4713_A8_SLLQHLIAL KD: 8.88e−09 Kass: 1.13e+05, kdiss: 1.00e−03
2631_ID.4716_N4_SLLNHLIGL KD: 9.51e−09 Kass: 1.43e+05, kdiss: 1.36e−03
2632_ID.4717_K1_KLLQHLIGL KD: 8.57e−09 Kass: 1.70e+05, kdiss: 1.46e−03
2633_ID.4718_N3_SLNQHLIGL KD: 1.38e−08 Kass: 1.66e+05, kdiss: 2.29e−03
2634_ID.4719_Y8_SLLQHLIYL KD: 2.46e−06 Kass: 1.45e+04, kdiss: 3.57e−02
2635_ID.472_E5_SLLQELIGL KD:~ Kass:~, , kdiss: 3.17e−02
2636_ID.4720_I4_SLLIHLIGL KD: 1.01e−08 Kass: 1.55e+05, kdiss: 1.56e−03
2637_ID.4721_E7_SLLQHLEGL KD: 2.32e−08 Kass: 4.31e−01, kdiss: 1.00e−08
2638_ID.4722_R5_SLLQRLIGL KD:~ Kass:~, , kdiss: 1.21e−01
2639_ID.4726_E4_SLLELHIGL KD: 1.26e−08 Kass: 9.19e+04, kdiss: 1.16e−03
2640_ID.4727_H7_SLLQHLHGL KD: 6.09e−08 Kass: 5.38e+04, kdiss: 3.28e−03
2641_ID.4729_R6_SLLQHRIGL KD: 7.85e−09 Kass: 1.61e+05, kdiss: 1.27e−03
2642_ID.473_N1_NLLQHLIGL KD: 1.06e−08 Kass: 9.36e+04, kdiss: 9.96e−04
2643_ID.4731_W6_SLLQHWIGL KD: 8.31e−09 Kass: 1.46e+05, kdiss: 1.21e−03
2644_ID.4733_G3_SLGQHLIGL KD: 8.30e−09 Kass: 1.94e+05, kdiss: 1.61e−03
2645_ID.4734_Q3_SLQQHLIGL KD: 6.08e−09 Kass: 1.84e+05, kdiss: 1.12e−03
2646_ID.4735_V3_SLVQHLIGL KD: 5.99e−09 Kass: 1.59e+05, kdiss: 9.51e−04
2647_ID.4736_W5_SLLQWLIGL KD: 1.59e−05 Kass: 1.73e+03, kdiss: 2.76e−02
2648_ID.4737_S6_SLLQHSIGL KD: 1.18e−08 Kass: 1.58e+05, kdiss: 1.87e−03
2649_ID.4738_P6_SLLQHPIGL KD: 1.92e−08 Kass: 5.21e−01, kdiss: 1.00e−08
2650_ID.4739_H3_SLHQHLIGL KD: 4.83e−08 Kass: 1.13e+05, kdiss: 5.45e−03
2651_ID.474_F4_SLLFHLIGL KD:~ Kass:~, , kdiss: 1.96e−01
2652_ID.4741_S6_SLLQHSIGL KD: 1.19e−08 Kass: 1.53e+05, kdiss: 1.82e−03
2653_ID.4742_T8_SLLQHLITL KD: 8.52e−09 Kass: 1.38e+05, kdiss: 1.18e−03
2654_ID.4743_R7_SLLQHLRGL KD:~ Kass:~, , kdiss: 5.69e−02
2655_ID.4747_N8_SLLQHLINL KD: 6.97e−08 Kass: 8.66e+04, kdiss: 6.03e−03
2656_ID.4748_W4_SLLWHLIGL KD: 1.24e−08 Kass: 1.29e+05, kdiss: 1.59e−03
2657_ID.4750_V5_SLLQVLIGL KD:~ Kass:~, , kdiss: 3.85e−02
2658_ID.4752_Y4_SLLYHLIGL KD: 1.34e−08 Kass: 1.33e+05, kdiss: 1.79e−03
2659_ID.4753_V7_SLLQHLVGL KD: 6.30e−09 Kass: 1.07e+05, kdiss: 6.74e−04
2660_ID.4754_A5_SLLQALIGL KD: 1.03e−07 Kass: 1.08e+05, kdiss: 1.11e−02
2661_ID.4756_N5_SLLQNLIGL KD: 2.84e−07 Kass: 7.16e+04, kdiss: 2.03e−02
2662_ID.4757_I8_SLLQHLIIL KD: 3.75e−08 Kass: 6.61e+04, kdiss: 2.48e−03
2663_ID.4759_F6_SLLQHFIGL KD: 1.07e−08 Kass: 8.78e+04, kdiss: 9.40e−04
2664_ID.476_M5_SLLQMLIGL KD:~ Kass:~, , kdiss: 2.76e−02
2665_ID.4761_S6_SLLQHSIGL KD: 2.92e−07 Kass: 7.71e+04, kdiss: 2.25e−03
2666_ID.4762_T4_SLLTHLIGL KD: 6.65e−09 Kass: 1.03e+05, kdiss: 6.83e−04
2667_ID.4763_Y8_SLLQHLIYL KD: 1.99e−06 Kass: 1.05e+04, kdiss: 2.10e−02
2668_ID.4764_S5_SLLQSLIGL KD: 2.29e−07 Kass: 6.93e+04, kdiss: 1.59e−02
2669_ID.4766_Y4_SLLYHLIGL KD: 6.18e−08 Kass: 8.03e+04, kdiss: 4.96e−03
2670_ID.4769_V1_VLLQHLIGL KD: 1.68e−08 Kass: 1.03e+05, kdiss: 1.73e−03
2671_ID.4770_S5_SLLQSLIGL KD: 2.10e−07 Kass: 7.93e+04, kdiss: 1.66e−02
2672_ID.4771_E6_SLLQHEIGL KD: 1.50e−07 Kass: 5.90e+04, kdiss: 8.82e−03
2673_ID.4772_P5_SLLQPLIGL KD: 3.82e−07 Kass: 4.38e+04, kdiss: 1.67e−02
2674_ID.4773_F8_SLLQHLIFL KD:~ Kass:~, , kdiss: 3.39e−02
2675_ID.4774_G4_SLLGHLIGL KD: 9.65e−09 Kass: 1.19e+05, kdiss: 1.15e−03
2676_ID.4775_W7_SLLQHLWGL KD:~ Kass:~, , kdiss: 6.07e−02
2677_ID.4779_H1_HLLQHLIGL KD: 8.09e−09 Kass: 1.11e+05, kdiss: 9.00e−04
2678_ID.478_M4_SLLMHLIGL KD: 4.36e−13 Kass: 1.05e+05, kdiss: 4.60e−08
2679_ID.4780_T4_SLLTHLIGL KD: 1.70e−08 Kass: 1.05e+05, kdiss: 1.79e−03
2680_ID.4782_D3_SLDQHLIGL KD: 6.00e−09 Kass: 1.18e+05, kdiss: 7.09e−04
2681_ID.4783_H1_HLLQHLIGL KD: 1.13e−08 Kass: 1.14e+05, kdiss: 1.30e−03
2682_ID.4786_R5_SLLQRLIGL KD:~ Kass:~, , kdiss: 6.33e−02
2683_ID.4787_Q6_SLLQHQIGL KD: 8.55e−09 Kass: 1.33e+05, kdiss: 1.14e−03
2684_ID.4788_D4_SLLDHLIGL KD: 1.36e−08 Kass: 7.90e+04, kdiss: 1.07e−03
2685_ID.4789_D7_SLLQHLDGL KD: 2.50e−08 Kass: 4.00e−01, kdiss: 1.00e−08
2686_ID.479_H1_HLLQHLIGL KD: 5.83e−13 Kass: 1.09e+05, kdiss: 6.39e−08
2687_ID.4791_R6_SLLQHRIGL KD: 8.61e−09 Kass: 1.57e+05, kdiss: 1.35e−03
2688_ID.4792_S4_SLLSHLIGL KD: 1.11e−08 Kass: 1.40e+05, kdiss: 1.56e−03
2689_ID.4793_K1_KLLQHLIGL KD: 9.59e−09 Kass: 1.77e+05, kdiss: 1.70e−03
2690_ID.4794_H4_SLLHHLIGL KD: 9.86e−09 Kass: 1.39e+05, kdiss: 1.37e−03
2691_ID.4795_M7_SLLQHLMGL KD: 8.40e−09 Kass: 1.68e+05, kdiss: 1.41e−03
2692_ID.4797_W7_SLLQHLWGL KD:~ Kass:~, , kdiss: 8.52e−03
2693_ID.4799_S8_SLLQHLISL KD: 9.33e−09 Kass: 1.39e+05, kdiss: 1.30e−03
2694_ID.480_Q5_SLLQQLIGL KD: 2.36e−07 Kass: 7.31e+04, kdiss: 1.73e−02
2695_ID.4801_S6_SLLQHSIGL KD: 1.21e−08 Kass: 1.53e+05, kdiss: 1.85e−03
2696_ID.4805_M8_SLLQHLIML KD: 8.42e−08 Kass: 6.89e+04, kdiss: 5.80e−03
2697_ID.4807_S7_SLLQHLSGL KD: 1.45e−07 Kass: 1.15e+05, kdiss: 1.67e−02
2698_ID.4809_L1_LLLQHLIGL KD: 1.16e−08 Kass: 1.53e+05, kdiss: 1.78e−03
2699_ID.481_P3_SLPQHLIGL KD: 1.13e−08 Kass: 9.72e+04, kdiss: 1.10e−03
2700_ID.4810_G6_SLLQHGIGL KD: 8.40e−09 Kass: 1.69e+05, kdiss: 1.42e−03
value
Time
0 250 500 750 1000
0 5000 10000 15000 20000

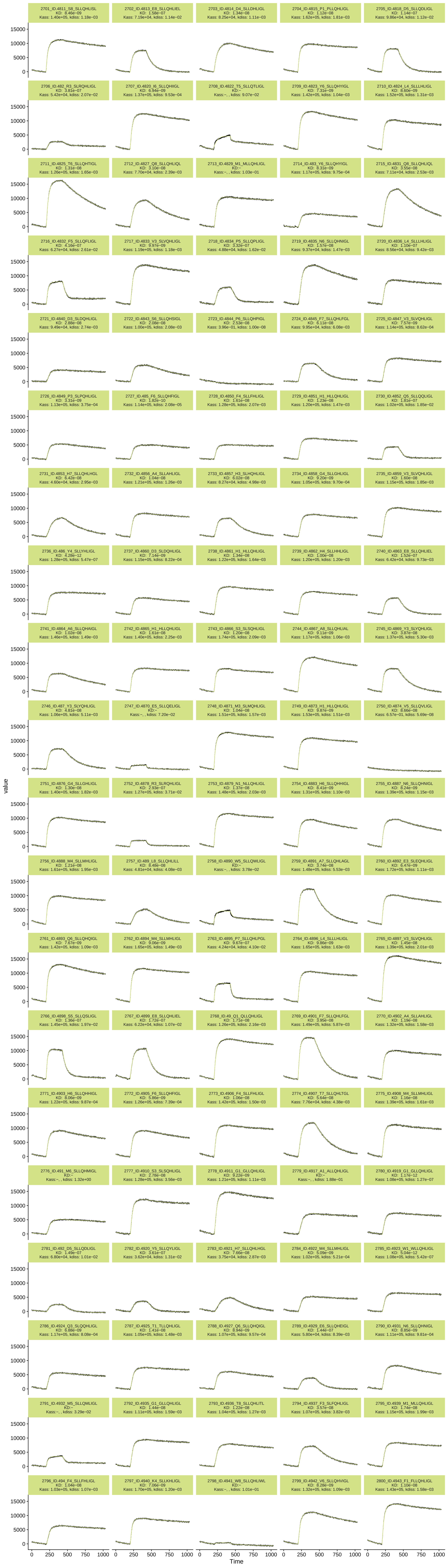
2701_ID.4811_S8_SLLQHLISL
KD: 8.46e−09
Kass: 1.40e+05, kdiss: 1.18e−03
2702_ID.4813_E8_SLLQHLIEL
KD: 1.58e−07
Kass: 7.19e+04, kdiss: 1.14e−02
2703_ID.4814_D4_SLLDHLIGL
KD: 1.34e−08
Kass: 8.25e+04, kdiss: 1.11e−03
2704_ID.4815_P1_PLLQHLIGL
KD: 1.12e−08
Kass: 1.62e+05, kdiss: 1.81e−03
2705_ID.4818_D5_SLLQDLIGL
KD: 1.14e−07
Kass: 9.86e+04, kdiss: 1.12e−02
2706_ID.482_R3_SLRQHLIGL
KD: 3.81e−07
Kass: 5.42e+04, kdiss: 2.07e−02
2707_ID.4820_I6_SLLQHIIGL
KD: 6.94e−09
Kass: 1.37e+05, kdiss: 9.53e−04
2708_ID.4822_T5_SLLQTLIGL
KD:−
Kass:−, , kdiss: 9.07e−02
2709_ID.4823_Y6_SLLQHYIGL
KD: 7.31e−09
Kass: 1.42e+05, kdiss: 1.04e−03
2710_ID.4824_L4_SLLLHLIGL
KD: 8.60e−09
Kass: 1.52e+05, kdiss: 1.31e−03
2711_ID.4825_T6_SLLQHTIGL
KD: 1.31e−08
Kass: 1.26e+05, kdiss: 1.65e−03
2712_ID.4827_Q8_SLLQHLIQL
KD: 3.10e−08
Kass: 7.70e+04, kdiss: 2.39e−03
2713_ID.4829_M1_MLLQHLIGL
KD:−
Kass:−, , kdiss: 1.03e−01
2714_ID.483_Y6_SLLQHYIGL
KD: 8.31e−09
Kass: 1.17e+05, kdiss: 9.75e−04
2715_ID.4831_Q8_SLLQHLIQL
KD: 3.55e−08
Kass: 7.11e+04, kdiss: 2.53e−03
2716_ID.4832_F5_SLLQFLIGL
KD: 4.16e−07
Kass: 6.27e+04, kdiss: 2.61e−02
2717_ID.4833_V3_SLVQHLIGL
KD: 9.97e−09
Kass: 1.19e+05, kdiss: 1.18e−03
2718_ID.4834_P5_SLLQPLIGL
KD: 3.32e−07
Kass: 4.88e+04, kdiss: 1.62e−02
2719_ID.4835_N6_SLLQHNIGL
KD: 1.57e−08
Kass: 9.37e+04, kdiss: 1.47e−03
2720_ID.4836_L4_SLLLHLIGL
KD: 1.10e−07
Kass: 8.56e+04, kdiss: 9.42e−03
2721_ID.4840_D3_SLDQHLIGL
KD: 2.88e−08
Kass: 9.49e+04, kdiss: 2.74e−03
2722_ID.4843_S6_SLLQHSIGL
KD: 2.08e−08
Kass: 1.00e+05, kdiss: 2.08e−03
2723_ID.4844_P6_SLLQHPIGL
KD: 2.53e−08
Kass: 3.96e−01, kdiss: 1.00e−08
2724_ID.4845_F7_SLLQHLFGL
KD: 6.11e−08
Kass: 9.95e+04, kdiss: 6.08e−03
2725_ID.4847_V3_SLVQHLIGL
KD: 7.57e−09
Kass: 1.14e+05, kdiss: 8.62e−04
2726_ID.4849_P3_SLPQHLIGL
KD: 3.31e−09
Kass: 1.13e+05, kdiss: 3.75e−04
2727_ID.485_F6_SLLQHFIGL
KD: 1.82e−10
Kass: 1.14e+05, kdiss: 2.08e−05
2728_ID.4850_F4_SLLFHLIGL
KD: 1.61e−08
Kass: 1.28e+05, kdiss: 2.07e−03
2729_ID.4851_H1_HLLQHLIGL
KD: 1.23e−08
Kass: 1.20e+05, kdiss: 1.47e−03
2730_ID.4852_Q5_SLLQQLIGL
KD: 1.81e−07
Kass: 1.02e+05, kdiss: 1.85e−02
2731_ID.4853_H7_SLLQHLHGL
KD: 6.42e−08
Kass: 4.60e+04, kdiss: 2.95e−03
2732_ID.4856_A4_SLLAHLIGL
KD: 1.04e−08
Kass: 1.21e+05, kdiss: 1.26e−03
2733_ID.4857_H3_SLHQHLIGL
KD: 6.02e−08
Kass: 8.27e+04, kdiss: 4.98e−03
2734_ID.4858_G4_SLLGHLIGL
KD: 9.20e−09
Kass: 1.05e+05, kdiss: 9.70e−04
2735_ID.4859_V3_SLVQHLIGL
KD: 1.60e−08
Kass: 1.15e+05, kdiss: 1.85e−03
2736_ID.486_Y4_SLLYHLIGL
KD: 4.28e−12
Kass: 1.28e+05, kdiss: 5.47e−07
2737_ID.4860_D3_SLDQHLIGL
KD: 7.14e−09
Kass: 1.15e+05, kdiss: 8.22e−04
2738_ID.4861_H1_HLLQHLIGL
KD: 1.34e−08
Kass: 1.22e+05, kdiss: 1.64e−03
2739_ID.4862_H4_SLLHHLIGL
KD: 1.00e−08
Kass: 1.20e+05, kdiss: 1.20e−03
2740_ID.4863_E8_SLLQHLIEL
KD: 1.52e−07
Kass: 6.42e+04, kdiss: 9.73e−03
2741_ID.4864_A6_SLLQHAIGL
KD: 1.02e−08
Kass: 1.46e+05, kdiss: 1.49e−03
2742_ID.4865_H1_HLLQHLIGL
KD: 1.61e−08
Kass: 1.40e+05, kdiss: 2.25e−03
2743_ID.4866_S3_SLSQHLIGL
KD: 1.20e−08
Kass: 1.74e+05, kdiss: 2.09e−03
2744_ID.4867_A8_SLLQHLIAL
KD: 9.11e−09
Kass: 1.17e+05, kdiss: 1.06e−03
2745_ID.4869_Y3_SLYQHLIGL
KD: 3.87e−08
Kass: 1.37e+05, kdiss: 5.30e−03
2746_ID.487_Y3_SLYQHLIGL
KD: 4.81e−08
Kass: 1.06e+05, kdiss: 5.11e−03
2747_ID.4870_E5_SLLQELIGL
KD:−
Kass:−, , kdiss: 7.20e−02
2748_ID.4871_M3_SLMQHLIGL
KD: 1.04e−08
Kass: 1.51e+05, kdiss: 1.57e−03
2749_ID.4873_H1_HLLQHLIGL
KD: 9.87e−09
Kass: 1.53e+05, kdiss: 1.51e−03
2750_ID.4874_V5_SLLQVLIGL
KD: 8.66e−08
Kass: 6.57e−01, kdiss: 5.69e−08
2751_ID.4876_G4_SLLGHLIGL
KD: 1.30e−08
Kass: 1.40e+05, kdiss: 1.82e−03
2752_ID.4878_R3_SLRQHLIGL
KD: 2.93e−07
Kass: 1.27e+05, kdiss: 3.71e−02
2753_ID.4879_N1_NLLQHLIGL
KD: 1.37e−08
Kass: 1.48e+05, kdiss: 2.03e−03
2754_ID.4883_H6_SLLQHHIGL
KD: 8.41e−09
Kass: 1.31e+05, kdiss: 1.10e−03
2755_ID.4887_N6_SLLQHNIGL
KD: 8.24e−09
Kass: 1.39e+05, kdiss: 1.15e−03
2756_ID.4888_M4_SLLMHLIGL
KD: 1.21e−08
Kass: 1.61e+05, kdiss: 1.95e−03
2757_ID.489_L8_SLLQHLILL
KD: 8.48e−08
Kass: 4.81e+04, kdiss: 4.08e−03
2758_ID.4890_W5_SLLQWLIGL
KD:−
Kass:−, , kdiss: 3.78e−02
2759_ID.4891_A7_SLLQHLAGL
KD: 3.74e−08
Kass: 1.48e+05, kdiss: 5.53e−03
2760_ID.4892_E3_SLEQHLIGL
KD: 6.47e−09
Kass: 1.72e+05, kdiss: 1.11e−03
2761_ID.4893_Q6_SLLQHQIGL
KD: 7.67e−09
Kass: 1.42e+05, kdiss: 1.09e−03
2762_ID.4894_M4_SLLMHLIGL
KD: 9.06e−09
Kass: 1.65e+05, kdiss: 1.49e−03
2763_ID.4895_P7_SLLQHLPGL
KD: 9.67e−07
Kass: 4.24e+04, kdiss: 4.10e−02
2764_ID.4896_L4_SLLLHLIGL
KD: 9.86e−09
Kass: 1.65e+05, kdiss: 1.63e−03
2765_ID.4897_V3_SLVQHLIGL
KD: 1.45e−08
Kass: 1.39e+05, kdiss: 2.01e−03
2766_ID.4898_S5_SLLQSLIGL
KD: 1.36e−07
Kass: 1.45e+05, kdiss: 1.97e−02
2767_ID.4899_E8_SLLQHLIEL
KD: 1.72e−07
Kass: 6.22e+04, kdiss: 1.07e−02
2768_ID.49_Q1_QLLQHLIGL
KD: 1.71e−08
Kass: 1.26e+05, kdiss: 2.16e−03
2769_ID.4901_F7_SLLQHLFGL
KD: 3.95e−08
Kass: 1.49e+05, kdiss: 5.87e−03
2770_ID.4902_A4_SLLAHLIGL
KD: 1.19e−08
Kass: 1.32e+05, kdiss: 1.58e−03
2771_ID.4903_H6_SLLQHHIGL
KD: 8.06e−09
Kass: 1.22e+05, kdiss: 9.87e−04
2772_ID.4905_F6_SLLQHFIGL
KD: 5.86e−09
Kass: 1.26e+05, kdiss: 7.39e−04
2773_ID.4906_F4_SLLFHLIGL
KD: 1.06e−08
Kass: 1.42e+05, kdiss: 1.50e−03
2774_ID.4907_T7_SLLQHLTGL
KD: 5.64e−08
Kass: 7.76e+04, kdiss: 4.38e−03
2775_ID.4908_M4_SLLMHLIGL
KD: 1.16e−08
Kass: 1.39e+05, kdiss: 1.61e−03
2776_ID.491_M6_SLLQHMIGL
KD:−
Kass:−, , kdiss: 1.32e+00
2777_ID.4910_S3_SLSQHLIGL
KD: 2.78e−08
Kass: 1.28e+05, kdiss: 3.56e−03
2778_ID.4911_G1_GLLQHLIGL
KD: 9.22e−09
Kass: 1.21e+05, kdiss: 1.11e−03
2779_ID.4917_A1_ALLQHLIGL
KD:−
Kass:−, , kdiss: 1.88e−01
2780_ID.4919_G1_GLLQHLIGL
KD: 1.17e−12
Kass: 1.08e+05, kdiss: 1.27e−07
2781_ID.492_D5_SLLQDLIGL
KD: 1.49e−07
Kass: 6.80e+04, kdiss: 1.01e−02
2782_ID.4920_Y5_SLLQYLIGL
KD: 3.61e−07
Kass: 3.62e+04, kdiss: 1.31e−02
2783_ID.4921_H7_SLLQHLHGL
KD: 7.66e−08
Kass: 3.75e+04, kdiss: 2.87e−03
2784_ID.4922_M4_SLLMHLIGL
KD: 5.09e−09
Kass: 1.02e+05, kdiss: 5.21e−04
2785_ID.4923_W1_WLLQHLIGL
KD: 5.04e−12
Kass: 1.08e+05, kdiss: 5.42e−07
2786_ID.4924_Q3_SLQQHLIGL
KD: 6.88e−09
Kass: 1.17e+05, kdiss: 8.08e−04
2787_ID.4925_T1_TLLQHLIGL
KD: 1.41e−08
Kass: 1.05e+05, kdiss: 1.48e−03
2788_ID.4927_Q6_SLLQHQIGL
KD: 8.94e−09
Kass: 1.07e+05, kdiss: 9.57e−04
2789_ID.4929_E6_SLLQHEIGL
KD: 1.44e−07
Kass: 5.80e+04, kdiss: 8.39e−03
2790_ID.4931_N6_SLLQHNIGL
KD: 8.85e−09
Kass: 1.11e+05, kdiss: 9.81e−04
2791_ID.4932_M5_SLLQMLIGL
KD:−
Kass:−, , kdiss: 3.29e−02
2792_ID.4935_G1_GLLQHLIGL
KD: 1.44e−08
Kass: 1.11e+05, kdiss: 1.59e−03
2793_ID.4936_T8_SLLQHLITL
KD: 1.22e−08
Kass: 1.04e+05, kdiss: 1.27e−03
2794_ID.4937_F3_SLFQHLIGL
KD: 3.57e−08
Kass: 1.07e+05, kdiss: 3.82e−03
2795_ID.4939_M1_MLLQHLIGL
KD: 1.74e−08
Kass: 1.15e+05, kdiss: 1.99e−03
2796_ID.494_F4_SLLFHLIGL
KD: 1.04e−08
Kass: 1.03e+05, kdiss: 1.07e−03
2797_ID.4940_K4_SLLKHLIGL
KD: 7.06e−09
Kass: 1.70e+05, kdiss: 1.20e−03
2798_ID.4941_W8_SLLQHLIWL
KD:−
Kass:−, , kdiss: 1.01e−01
2799_ID.4942_V6_SLLQHVIGL
KD: 8.28e−09
Kass: 1.32e+05, kdiss: 1.09e−03
2800_ID.4943_F1_FLLQHLIGL
KD: 1.10e−08
Kass: 1.43e+05, kdiss: 1.58e−03
value
0
5000
10000
15000
Time
0
250
500
750
1000

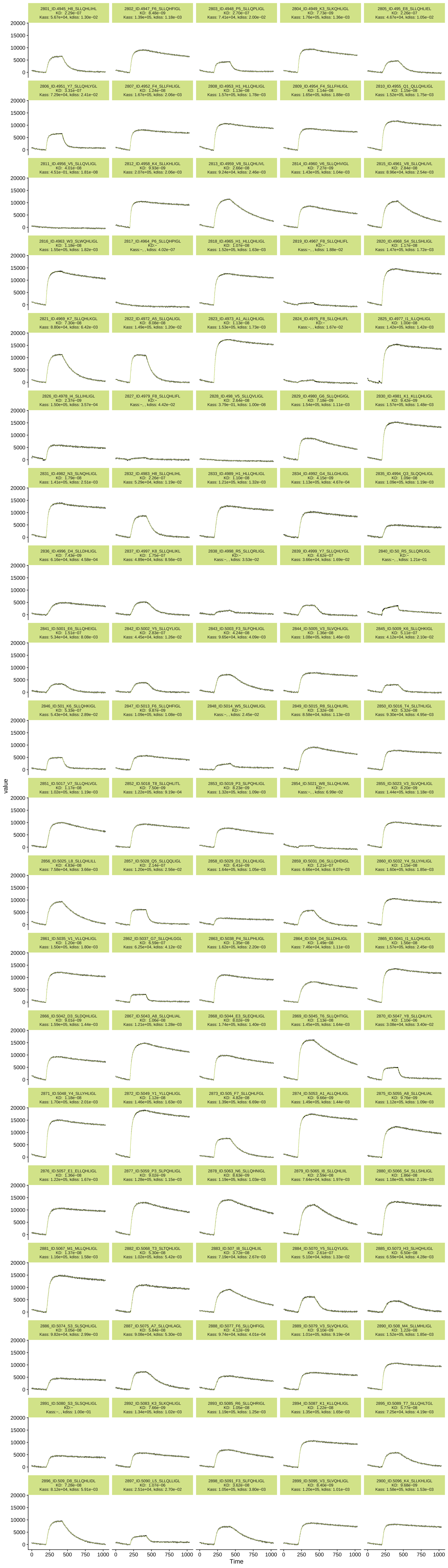
2801_ID.4945_H8_SLLQHLIHL
KD: 2.29e−07
Kass: 5.67e+04, kdiss: 1.30e−02
2802_ID.4947_F6_SLLQHFIGL
KD: 8.48e−09
Kass: 1.39e+05, kdiss: 1.18e−03
2803_ID.4948_P5_SLLQPLIGL
KD: 2.70e−07
Kass: 7.41e+04, kdiss: 2.00e−02
2804_ID.4949_K3_SLKQHLIGL
KD: 7.73e−09
Kass: 1.76e+05, kdiss: 1.36e−03
2805_ID.495_E8_SLLQHLIEL
KD: 2.26e−07
Kass: 4.67e+04, kdiss: 1.05e−02
2806_ID.4951_Y7_SLLQHLYGL
KD: 3.31e−07
Kass: 7.29e+04, kdiss: 2.41e−02
2807_ID.4952_F4_SLLFHLIGL
KD: 1.24e−08
Kass: 1.67e+05, kdiss: 2.06e−03
2808_ID.4953_H1_HLLQHLIGL
KD: 1.13e−08
Kass: 1.57e+05, kdiss: 1.78e−03
2809_ID.4954_F4_SLLFHLIGL
KD: 1.14e−08
Kass: 1.65e+05, kdiss: 1.88e−03
2810_ID.4955_Q1_QLLQHLIGL
KD: 1.15e−08
Kass: 1.52e+05, kdiss: 1.75e−03
2811_ID.4956_V5_SLLQVLIGL
KD: 4.01e−08
Kass: 4.51e−01, kdiss: 1.81e−08
2812_ID.4958_K4_SLLKHLIGL
KD: 9.93e−09
Kass: 2.07e+05, kdiss: 2.06e−03
2813_ID.4959_V8_SLLQHLIVL
KD: 2.66e−08
Kass: 9.24e+04, kdiss: 2.46e−03
2814_ID.4960_V6_SLLQHVIGL
KD: 7.27e−09
Kass: 1.43e+05, kdiss: 1.04e−03
2815_ID.4961_V8_SLLQHLIVL
KD: 2.84e−08
Kass: 8.96e+04, kdiss: 2.54e−03
2816_ID.4963_W3_SLWQHLIGL
KD: 1.18e−08
Kass: 1.55e+05, kdiss: 1.82e−03
2817_ID.4964_P6_SLLQHPIGL
KD:−
Kass:−, , kdiss: 4.02e−07
2818_ID.4965_H1_HLLQHLIGL
KD: 1.07e−08
Kass: 1.52e+05, kdiss: 1.63e−03
2819_ID.4967_F8_SLLQHLIFL
KD:−
Kass:−, , kdiss: 1.88e−02
2820_ID.4968_S4_SLLSHLIGL
KD: 1.17e−08
Kass: 1.47e+05, kdiss: 1.72e−03
2821_ID.4969_K7_SLLQHLKGL
KD: 7.30e−08
Kass: 8.80e+04, kdiss: 6.42e−03
2822_ID.4972_A5_SLLQALIGL
KD: 8.08e−08
Kass: 1.49e+05, kdiss: 1.20e−02
2823_ID.4973_A1_ALLQHLIGL
KD: 1.13e−08
Kass: 1.53e+05, kdiss: 1.73e−03
2824_ID.4975_F8_SLLQHLIFL
KD:−
Kass:−, , kdiss: 1.67e−02
2825_ID.4977_I1_ILLQHLIGL
KD: 1.00e−08
Kass: 1.42e+05, kdiss: 1.42e−03
2826_ID.4978_I4_SLLIHLIGL
KD: 2.37e−09
Kass: 1.50e+05, kdiss: 3.57e−04
2827_ID.4979_F8_SLLQHLIFL
KD:−
Kass:−, , kdiss: 4.42e−02
2828_ID.498_V5_SLLQVLIGL
KD: 2.64e−08
Kass: 3.79e−01, kdiss: 1.00e−08
2829_ID.4980_G6_SLLQHGIGL
KD: 7.18e−09
Kass: 1.54e+05, kdiss: 1.11e−03
2830_ID.4981_K1_KLLQHLIGL
KD: 9.42e−09
Kass: 1.57e+05, kdiss: 1.48e−03
2831_ID.4982_N3_SLNQHLIGL
KD: 1.79e−08
Kass: 1.41e+05, kdiss: 2.51e−03
2832_ID.4983_H8_SLLQHLIHL
KD: 2.26e−07
Kass: 5.29e+04, kdiss: 1.19e−02
2833_ID.4989_H1_HLLQHLIGL
KD: 1.10e−08
Kass: 1.21e+05, kdiss: 1.32e−03
2834_ID.4992_G4_SLLGHLIGL
KD: 4.15e−09
Kass: 1.13e+05, kdiss: 4.67e−04
2835_ID.4994_Q3_SLQQHLIGL
KD: 1.09e−08
Kass: 1.09e+05, kdiss: 1.19e−03
2836_ID.4996_D4_SLLDHLIGL
KD: 7.43e−09
Kass: 6.16e+04, kdiss: 4.58e−04
2837_ID.4997_K8_SLLQHLIKL
KD: 1.75e−07
Kass: 4.89e+04, kdiss: 8.56e−03
2838_ID.4998_R5_SLLQRLIGL
KD:−
Kass:−, , kdiss: 3.53e−02
2839_ID.4999_Y7_SLLQHLYGL
KD: 4.62e−07
Kass: 3.66e+04, kdiss: 1.69e−02
2840_ID.50_R5_SLLQRLIGL
KD:−
Kass:−, , kdiss: 1.21e−01
2841_ID.5001_E6_SLLQHEIGL
KD: 1.51e−07
Kass: 5.34e+04, kdiss: 8.08e−03
2842_ID.5002_Y5_SLLQYLIGL
KD: 2.83e−07
Kass: 4.45e+04, kdiss: 1.26e−02
2843_ID.5003_F3_SLFQHLIGL
KD: 4.24e−08
Kass: 9.65e+04, kdiss: 4.09e−03
2844_ID.5005_V3_SLVQHLIGL
KD: 1.36e−08
Kass: 1.08e+05, kdiss: 1.46e−03
2845_ID.5009_K6_SLLQHKIGL
KD: 5.11e−07
Kass: 4.12e+04, kdiss: 2.10e−02
2846_ID.501_K6_SLLQHKIGL
KD: 5.33e−07
Kass: 5.43e+04, kdiss: 2.89e−02
2847_ID.5013_F6_SLLQHFIGL
KD: 9.87e−09
Kass: 1.09e+05, kdiss: 1.08e−03
2848_ID.5014_W5_SLLQWLIGL
KD:−
Kass:−, , kdiss: 2.45e−02
2849_ID.5015_R8_SLLQHLIRL
KD: 1.32e−08
Kass: 8.58e+04, kdiss: 1.13e−03
2850_ID.5016_T4_SLLTHLIGL
KD: 5.32e−08
Kass: 9.30e+04, kdiss: 4.95e−03
2851_ID.5017_V7_SLLQHLVGL
KD: 1.17e−08
Kass: 1.02e+05, kdiss: 1.19e−03
2852_ID.5018_T8_SLLQHLITL
KD: 7.50e−09
Kass: 1.22e+05, kdiss: 9.19e−04
2853_ID.5019_P3_SLPQHLIGL
KD: 8.23e−09
Kass: 1.32e+05, kdiss: 1.09e−03
2854_ID.5021_W8_SLLQHLIWL
KD:−
Kass:−, , kdiss: 6.99e−02
2855_ID.5023_V3_SLVQHLIGL
KD: 8.20e−09
Kass: 1.44e+05, kdiss: 1.18e−03
2856_ID.5025_L8_SLLQHLILL
KD: 4.83e−08
Kass: 7.58e+04, kdiss: 3.66e−03
2857_ID.5028_Q5_SLLQQLIGL
KD: 2.14e−07
Kass: 1.20e+05, kdiss: 2.56e−02
2858_ID.5029_D1_DLLQHLIGL
KD: 6.41e−09
Kass: 1.64e+05, kdiss: 1.05e−03
2859_ID.5031_D6_SLLQHDIGL
KD: 1.21e−07
Kass: 6.66e+04, kdiss: 8.07e−03
2860_ID.5032_Y4_SLLYHLIGL
KD: 1.15e−08
Kass: 1.60e+05, kdiss: 1.85e−03
2861_ID.5035_V1_VLLQHLIGL
KD: 1.20e−08
Kass: 1.50e+05, kdiss: 1.80e−03
2862_ID.5037_G7_SLLQHLGGL
KD: 6.59e−07
Kass: 6.25e+04, kdiss: 4.12e−02
2863_ID.5038_P4_SLLPHLIGL
KD: 1.35e−08
Kass: 1.62e+05, kdiss: 2.20e−03
2864_ID.504_D4_SLLDHLIGL
KD: 1.49e−08
Kass: 7.46e+04, kdiss: 1.11e−03
2865_ID.5041_I1_ILLQHLIGL
KD: 1.56e−08
Kass: 1.57e+05, kdiss: 2.45e−03
2866_ID.5042_D3_SLDQHLIGL
KD: 9.01e−09
Kass: 1.59e+05, kdiss: 1.44e−03
2867_ID.5043_A8_SLLQHLIAL
KD: 1.06e−08
Kass: 1.21e+05, kdiss: 1.28e−03
2868_ID.5044_E3_SLEQHLIGL
KD: 8.02e−09
Kass: 1.74e+05, kdiss: 1.40e−03
2869_ID.5045_T6_SLLQHTIGL
KD: 1.13e−08
Kass: 1.45e+05, kdiss: 1.64e−03
2870_ID.5047_Y8_SLLQHLIYL
KD: 1.10e−06
Kass: 3.08e+04, kdiss: 3.40e−02
2871_ID.5048_Y4_SLLYHLIGL
KD: 1.18e−08
Kass: 1.70e+05, kdiss: 2.01e−03
2872_ID.5049_Y1_YLLQHLIGL
KD: 1.12e−08
Kass: 1.46e+05, kdiss: 1.63e−03
2873_ID.505_F7_SLLQHLFGL
KD: 4.82e−08
Kass: 1.39e+05, kdiss: 6.69e−03
2874_ID.5053_A1_ALLQHLIGL
KD: 9.66e−09
Kass: 1.49e+05, kdiss: 1.44e−03
2875_ID.5055_A8_SLLQHLIAL
KD: 9.76e−09
Kass: 1.12e+05, kdiss: 1.09e−03
2876_ID.5057_E1_ELLQHLIGL
KD: 1.36e−08
Kass: 1.22e+05, kdiss: 1.67e−03
2877_ID.5059_P3_SLPQHLIGL
KD: 9.02e−09
Kass: 1.28e+05, kdiss: 1.15e−03
2878_ID.5063_N6_SLLQHNIGL
KD: 8.63e−09
Kass: 1.19e+05, kdiss: 1.03e−03
2879_ID.5065_I8_SLLQHLIIL
KD: 2.59e−08
Kass: 7.64e+04, kdiss: 1.97e−03
2880_ID.5066_S4_SLLSHLIGL
KD: 1.86e−08
Kass: 1.18e+05, kdiss: 2.19e−03
2881_ID.5067_M1_MLLQHLIGL
KD: 1.37e−08
Kass: 1.16e+05, kdiss: 1.58e−03
2882_ID.5068_T3_SLTQHLIGL
KD: 5.30e−08
Kass: 1.02e+05, kdiss: 5.42e−03
2883_ID.507_I8_SLLQHLIIL
KD: 3.72e−08
Kass: 7.19e+04, kdiss: 2.67e−03
2884_ID.5070_Y5_SLLQYLIGL
KD: 2.61e−07
Kass: 5.10e+04, kdiss: 1.33e−02
2885_ID.5073_H3_SLHQHLIGL
KD: 6.50e−08
Kass: 6.59e+04, kdiss: 4.28e−03
2886_ID.5074_S3_SLSQHLIGL
KD: 3.05e−08
Kass: 9.82e+04, kdiss: 2.99e−03
2887_ID.5075_A7_SLLQHLAGL
KD: 5.84e−08
Kass: 9.08e+04, kdiss: 5.30e−03
2888_ID.5077_F6_SLLQHFIGL
KD: 4.12e−09
Kass: 9.74e+04, kdiss: 4.01e−04
2889_ID.5079_V3_SLVQHLIGL
KD: 9.10e−09
Kass: 1.01e+05, kdiss: 9.19e−04
2890_ID.508_M4_SLLMHLIGL
KD: 1.22e−08
Kass: 1.52e+05, kdiss: 1.85e−03
2891_ID.5080_S3_SLSQHLIGL
KD:−
Kass:−, , kdiss: 1.00e−01
2892_ID.5083_K3_SLKQHLIGL
KD: 7.66e−09
Kass: 1.34e+05, kdiss: 1.02e−03
2893_ID.5085_R6_SLLQHRIGL
KD: 1.05e−08
Kass: 1.19e+05, kdiss: 1.25e−03
2894_ID.5087_K1_KLLQHLIGL
KD: 1.22e−08
Kass: 1.35e+05, kdiss: 1.65e−03
2895_ID.5089_T7_SLLQHLTGL
KD: 5.77e−08
Kass: 7.25e+04, kdiss: 4.19e−03
2896_ID.509_D8_SLLQHLIDL
KD: 7.28e−08
Kass: 8.12e+04, kdiss: 5.91e−03
2897_ID.5090_L5_SLLQLLIGL
KD: 1.07e−06
Kass: 2.51e+04, kdiss: 2.70e−02
2898_ID.5091_F3_SLFQHLIGL
KD: 3.62e−08
Kass: 1.05e+05, kdiss: 3.80e−03
2899_ID.5095_V3_SLVQHLIGL
KD: 8.40e−09
Kass: 1.20e+05, kdiss: 1.01e−03
2900_ID.5096_K4_SLLKHLIGL
KD: 9.68e−09
Kass: 1.58e+05, kdiss: 1.53e−03
value
0
5000
10000
15000
20000
Time
0
250
500
750
1000

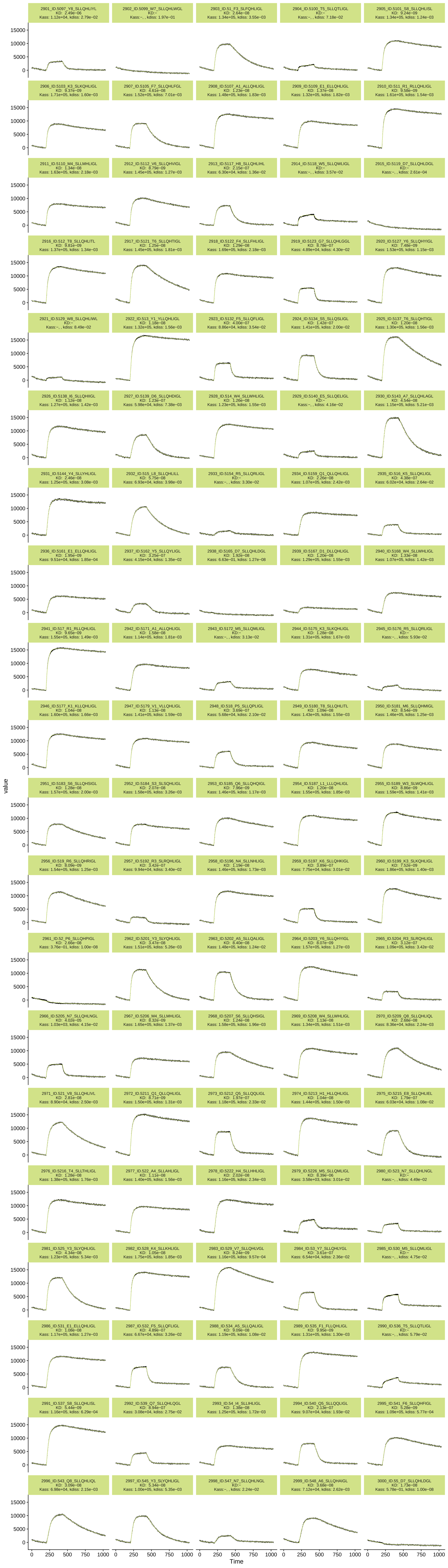

2901_ID.5097_Y8_SLLQHLIYL
KD: 2.49e−06
Kass: 1.12e+04, kdiss: 2.79e−02
2902_ID.5099_W7_SLLQHLWGL
KD:~
Kass:~ , kdiss: 1.97e−01
2903_ID.51_F3_SLFQHLIGL
KD: 2.64e−08
Kass: 1.34e+05, kdiss: 3.55e−03
2904_ID.5100_T5_SLLQTLIGL
KD:~
Kass:~ , kdiss: 7.18e−02
2905_ID.5101_S8_SLLQHLISL
KD: 9.24e−09
Kass: 1.34e+05, kdiss: 1.24e−03
2906_ID.5103_K3_SLKQHLIGL
KD: 9.37e−09
Kass: 1.71e+05, kdiss: 1.60e−03
2907_ID.5105_F7_SLLQHLFGL
KD: 4.61e−08
Kass: 1.52e+05, kdiss: 7.01e−03
2908_ID.5107_A1_ALLQHLIGL
KD: 1.23e−08
Kass: 1.48e+05, kdiss: 1.83e−03
2909_ID.5109_E1_ELLQHLIGL
KD: 1.37e−08
Kass: 1.32e+05, kdiss: 1.82e−03
2910_ID.511_R1_RLLQHLIGL
KD: 9.58e−09
Kass: 1.61e+05, kdiss: 1.54e−03
2911_ID.5110_M4_SLLMHLIGL
KD: 1.34e−08
Kass: 1.63e+05, kdiss: 2.18e−03
2912_ID.5112_V6_SLLQHVIGL
KD: 8.79e−09
Kass: 1.45e+05, kdiss: 1.27e−03
2913_ID.5117_H8_SLLQHLIHL
KD: 2.15e−07
Kass: 6.30e+04, kdiss: 1.36e−02
2914_ID.5118_W5_SLLQWLIGL
KD:~
Kass:~ , kdiss: 3.57e−02
2915_ID.5119_D7_SLLQHLDGL
KD:~
Kass:~ , kdiss: 2.61e−04
2916_ID.512_T8_SLLQHLITL
KD: 9.81e−09
Kass: 1.37e+05, kdiss: 1.34e−03
2917_ID.5121_T6_SLLQHTIGL
KD: 1.25e−08
Kass: 1.45e+05, kdiss: 1.81e−03
2918_ID.5122_F4_SLLFHLIGL
KD: 1.29e−08
Kass: 1.69e+05, kdiss: 2.18e−03
2919_ID.5123_G7_SLLQHLGGL
KD: 8.78e−07
Kass: 4.89e+04, kdiss: 4.30e−02
2920_ID.5127_Y6_SLLQHYIGL
KD: 7.48e−09
Kass: 1.53e+05, kdiss: 1.15e−03
2921_ID.5129_W8_SLLQHLIWL
KD:~
Kass:~ , kdiss: 8.49e−02
2922_ID.513_Y1_YLLQHLIGL
KD: 1.18e−08
Kass: 1.32e+05, kdiss: 1.56e−03
2923_ID.5132_F5_SLLQFLIGL
KD: 4.00e−07
Kass: 8.86e+04, kdiss: 3.54e−02
2924_ID.5134_S5_SLLQSLIGL
KD: 1.42e−07
Kass: 1.41e+05, kdiss: 2.00e−02
2925_ID.5137_T6_SLLQHTIGL
KD: 1.20e−08
Kass: 1.30e+05, kdiss: 1.56e−03
2926_ID.5138_I6_SLLQHIIGL
KD: 1.12e−08
Kass: 1.27e+05, kdiss: 1.42e−03
2927_ID.5139_D6_SLLQHDIGL
KD: 1.23e−07
Kass: 5.98e+04, kdiss: 7.38e−03
2928_ID.514_W4_SLLWHLIGL
KD: 1.26e−08
Kass: 1.23e+05, kdiss: 1.55e−03
2929_ID.5140_E5_SLLQELIGL
KD:~
Kass:~ , kdiss: 4.16e−02
2930_ID.5143_A7_SLLQHLAGL
KD: 4.54e−08
Kass: 1.15e+05, kdiss: 5.21e−03
2931_ID.5144_Y4_SLLYHLIGL
KD: 2.46e−08
Kass: 1.25e+05, kdiss: 3.08e−03
2932_ID.515_L8_SLLQHLILL
KD: 5.75e−08
Kass: 6.93e+04, kdiss: 3.98e−03
2933_ID.5154_R5_SLLQRLIGL
KD:~
Kass:~ , kdiss: 3.30e−02
2934_ID.5159_Q1_QLLQHLIGL
KD: 2.26e−08
Kass: 1.07e+05, kdiss: 2.42e−03
2935_ID.516_K5_SLLQKLIGL
KD: 4.38e−07
Kass: 6.02e+04, kdiss: 2.64e−02
2936_ID.5161_E1_ELLQHLIGL
KD: 1.95e−09
Kass: 9.51e+04, kdiss: 1.85e−04
2937_ID.5162_Y5_SLLQYLIGL
KD: 3.25e−07
Kass: 4.15e+04, kdiss: 1.35e−02
2938_ID.5165_D7_SLLQHLDGL
KD: 1.92e−08
Kass: 6.63e−01, kdiss: 1.27e−08
2939_ID.5167_D1_DLLQHLIGL
KD: 1.20e−08
Kass: 1.29e+05, kdiss: 1.55e−03
2940_ID.5168_W4_SLLWHLIGL
KD: 1.33e−08
Kass: 1.07e+05, kdiss: 1.42e−03
2941_ID.517_R1_RLLQHLIGL
KD: 9.65e−09
Kass: 1.55e+05, kdiss: 1.49e−03
2942_ID.5171_A1_ALLQHLIGL
KD: 1.58e−08
Kass: 1.14e+05, kdiss: 1.81e−03
2943_ID.5172_M5_SLLQMLIGL
KD:~
Kass:~ , kdiss: 3.13e−02
2944_ID.5175_K3_SLKQHLIGL
KD: 1.28e−08
Kass: 1.31e+05, kdiss: 1.67e−03
2945_ID.5176_R5_SLLQRLIGL
KD:~
Kass:~ , kdiss: 5.93e−02
2946_ID.5177_K1_KLLQHLIGL
KD: 1.04e−08
Kass: 1.60e+05, kdiss: 1.66e−03
2947_ID.5179_V1_VLLQHLIGL
KD: 1.13e−08
Kass: 1.41e+05, kdiss: 1.59e−03
2948_ID.518_P5_SLLQPLIGL
KD: 3.69e−07
Kass: 5.68e+04, kdiss: 2.10e−02
2949_ID.5180_T8_SLLQHLITL
KD: 1.09e−08
Kass: 1.43e+05, kdiss: 1.55e−03
2950_ID.5181_M6_SLLQHMIGL
KD: 8.54e−09
Kass: 1.46e+05, kdiss: 1.25e−03
2951_ID.5183_S6_SLLQHSIGL
KD: 1.28e−08
Kass: 1.57e+05, kdiss: 2.00e−03
2952_ID.5184_S3_SLSQHLIGL
KD: 2.07e−08
Kass: 1.58e+05, kdiss: 3.26e−03
2953_ID.5185_Q6_SLLQHQIGL
KD: 7.96e−09
Kass: 1.46e+05, kdiss: 1.17e−03
2954_ID.5187_L1_LLLQHLIGL
KD: 1.20e−08
Kass: 1.55e+05, kdiss: 1.85e−03
2955_ID.5189_W3_SLWQHLIGL
KD: 8.86e−09
Kass: 1.59e+05, kdiss: 1.41e−03
2956_ID.519_R6_SLLQHRIGL
KD: 8.09e−09
Kass: 1.54e+05, kdiss: 1.25e−03
2957_ID.5192_R3_SLRQHLIGL
KD: 3.42e−07
Kass: 9.94e+04, kdiss: 3.40e−02
2958_ID.5196_N4_SLLNHLIGL
KD: 1.19e−08
Kass: 1.46e+05, kdiss: 1.73e−03
2959_ID.5197_K6_SLLQHKIGL
KD: 3.89e−07
Kass: 7.75e+04, kdiss: 3.01e−02
2960_ID.5199_K3_SLKQHLIGL
KD: 7.52e−09
Kass: 1.86e+05, kdiss: 1.40e−03
2961_ID.52_P6_SLLQHPIGL
KD: 2.66e−09
Kass: 3.76e−01, kdiss: 1.00e−08
2962_ID.5201_Y3_SLYQHLIGL
KD: 3.48e−08
Kass: 1.51e+05, kdiss: 5.26e−03
2963_ID.5202_A5_SLLQALIGL
KD: 8.40e−08
Kass: 1.48e+05, kdiss: 1.24e−02
2964_ID.5203_Y6_SLLQHYIGL
KD: 8.07e−09
Kass: 1.57e+05, kdiss: 1.27e−03
2965_ID.5204_R3_SLRQHLIGL
KD: 3.12e−07
Kass: 1.09e+05, kdiss: 3.42e−02
2966_ID.5205_N7_SLLQHLNGL
KD: 4.02e−05
Kass: 1.03e+03, kdiss: 4.15e−02
2967_ID.5206_M4_SLLMHLIGL
KD: 8.32e−09
Kass: 1.65e+05, kdiss: 1.37e−03
2968_ID.5207_S6_SLLQHSIGL
KD: 1.24e−08
Kass: 1.58e+05, kdiss: 1.96e−03
2969_ID.5208_W4_SLLWHLIGL
KD: 1.13e−08
Kass: 1.34e+05, kdiss: 1.51e−03
2970_ID.5209_Q8_SLLQHLIQL
KD: 2.68e−08
Kass: 8.36e+04, kdiss: 2.24e−03
2971_ID.521_V8_SLLQHLIVL
KD: 2.81e−08
Kass: 8.90e+04, kdiss: 2.50e−03
2972_ID.5211_Q1_QLLQHLIGL
KD: 8.71e−09
Kass: 1.50e+05, kdiss: 1.31e−03
2973_ID.5212_Q5_SLLQQLIGL
KD: 1.97e−07
Kass: 1.18e+05, kdiss: 2.33e−02
2974_ID.5213_H1_HLLQHLIGL
KD: 1.04e−08
Kass: 1.44e+05, kdiss: 1.50e−03
2975_ID.5215_E8_SLLQHLIEL
KD: 1.79e−07
Kass: 6.03e+04, kdiss: 1.08e−02
2976_ID.5216_T4_SLLTHLIGL
KD: 1.28e−08
Kass: 1.38e+05, kdiss: 1.76e−03
2977_ID.522_A4_SLLAHLIGL
KD: 1.11e−08
Kass: 1.40e+05, kdiss: 1.56e−03
2978_ID.5222_H4_SLLHHLIGL
KD: 2.02e−08
Kass: 1.16e+05, kdiss: 2.34e−03
2979_ID.5226_M5_SLLQMLIGL
KD: 8.39e−06
Kass: 3.58e+03, kdiss: 3.01e−02
2980_ID.523_N7_SLLQHLNGL
KD:~
Kass:~ , kdiss: 4.49e−02
2981_ID.525_Y3_SLYQHLIGL
KD: 4.34e−08
Kass: 1.23e+05, kdiss: 5.34e−03
2982_ID.528_K4_SLLKHLIGL
KD: 1.05e−08
Kass: 1.75e+05, kdiss: 1.85e−03
2983_ID.529_V7_SLLQHLVGL
KD: 8.24e−09
Kass: 1.16e+05, kdiss: 9.57e−04
2984_ID.53_Y7_SLLQHLYGL
KD: 3.61e−07
Kass: 6.54e+04, kdiss: 2.36e−02
2985_ID.530_M5_SLLQMLIGL
KD:~
Kass:~ , kdiss: 4.75e−02
2986_ID.531_E1_ELLQHLIGL
KD: 1.08e−08
Kass: 1.17e+05, kdiss: 1.27e−03
2987_ID.532_F5_SLLQFLIGL
KD: 4.89e−07
Kass: 6.67e+04, kdiss: 3.26e−02
2988_ID.534_A5_SLLQALIGL
KD: 9.09e−08
Kass: 1.19e+05, kdiss: 1.08e−02
2989_ID.535_F1_FLLQHLIGL
KD: 9.95e−09
Kass: 1.31e+05, kdiss: 1.30e−03
2990_ID.536_T5_SLLQTLIGL
KD:~
Kass:~ , kdiss: 5.79e−02
2991_ID.537_S8_SLLQHLISL
KD: 5.44e−09
Kass: 1.16e+05, kdiss: 6.29e−04
2992_ID.539_Q7_SLLQHLQGL
KD: 8.94e−07
Kass: 3.08e+04, kdiss: 2.75e−02
2993_ID.54_I4_SLLIHLIGL
KD: 1.38e−08
Kass: 1.25e+05, kdiss: 1.72e−03
2994_ID.540_Q5_SLLQQLIGL
KD: 2.13e−07
Kass: 9.07e+04, kdiss: 1.93e−02
2995_ID.541_F6_SLLQHFIGL
KD: 5.28e−09
Kass: 1.09e+05, kdiss: 5.77e−04
2996_ID.543_Q8_SLLQHLIQL
KD: 3.09e−08
Kass: 6.98e+04, kdiss: 2.15e−03
2997_ID.545_Y3_SLYQHLIGL
KD: 5.34e−08
Kass: 1.00e+05, kdiss: 5.35e−03
2998_ID.547_N7_SLLQHLNGL
KD:~
Kass:~ , kdiss: 2.24e−02
2999_ID.548_A6_SLLQHAIGL
KD: 3.68e−08
Kass: 7.12e+04, kdiss: 2.62e−03
3000_ID.55_D7_SLLQHLDGL
KD: 1.73e−08
Kass: 5.78e−01, kdiss: 1.00e−08
value
Time
0
5000
10000
15000
0
250
500
750
1000

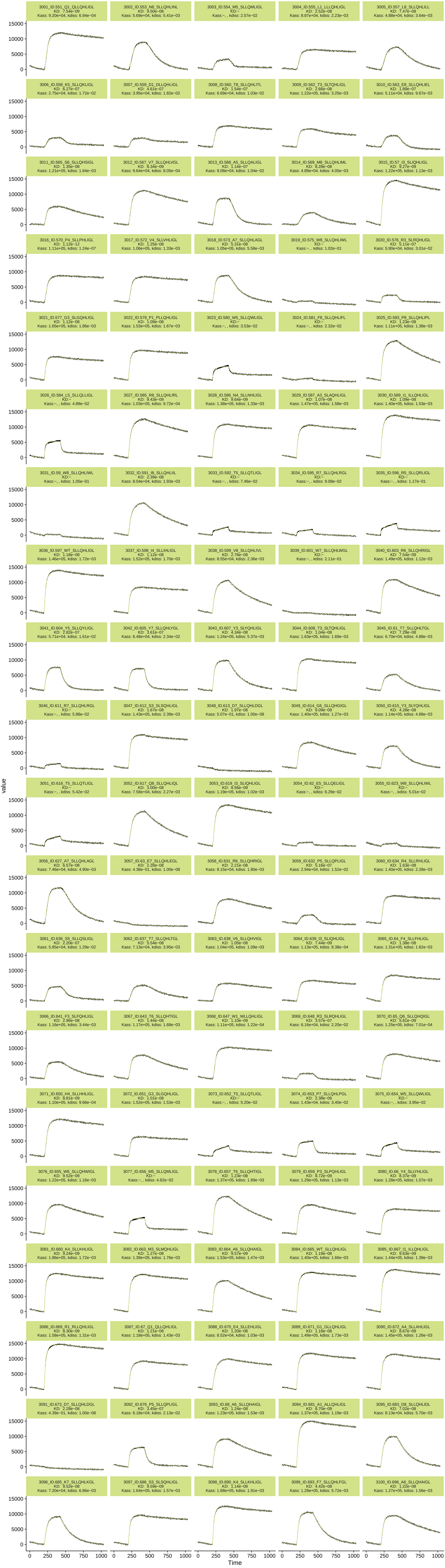

3001_ID.551_Q1_QLLQHLIGL KD: 7.54e-09 Kass: 9.20e+04, kdiss: 6.94e-04
3002_ID.553_N8_SLLQHLINL KD: 9.50e-08 Kass: 5.69e+04, kdiss: 5.41e-03
3003_ID.554_M5_SLLQMLIGL KD:~ Kass:~ , kdiss: 2.57e-02
3004_ID.555_L1_LLLQHLIGL KD: 2.52e-08 Kass: 8.87e+04, kdiss: 2.23e-03
3005_ID.557_L8_SLLQHLILL KD: 7.47e-08 Kass: 4.88e+04, kdiss: 3.64e-03
3006_ID.558_K5_SLLQKLIGL KD: 6.22e-08 Kass: 2.75e+04, kdiss: 1.72e-02
3007_ID.559_D1_DLLQHLIGL KD: 4.61e-07 Kass: 3.95e+04, kdiss: 1.82e-02
3008_ID.560_T8_SLLQHLITL KD: 1.54e-07 Kass: 6.69e+04, kdiss: 1.03e-02
3009_ID.562_T3_SLTQHLIGL KD: 2.66e-08 Kass: 1.22e+05, kdiss: 3.25e-03
3010_ID.563_E8_SLLQHLIEL KD: 1.89e-07 Kass: 5.11e+04, kdiss: 9.67e-03
3011_ID.565_S6_SLLQHSIGL KD: 1.35e-08 Kass: 1.21e+05, kdiss: 1.64e-03
3012_ID.567_V7_SLLQHLVGL KD: 8.34e-09 Kass: 9.64e+04, kdiss: 8.05e-04
3013_ID.568_A5_SLLQALIGL KD: 1.14e-07 Kass: 9.08e+04, kdiss: 1.04e-02
3014_ID.569_M8_SLLQHLIML KD: 8.28e-08 Kass: 4.89e+04, kdiss: 4.05e-03
3015_ID.57_I3_SLIQHLIGL KD: 9.27e-09 Kass: 1.22e+05, kdiss: 1.13e-03
3016_ID.570_P4_SLLPHLIGL KD: 1.12e-12 Kass: 1.11e+05, kdiss: 1.24e-07
3017_ID.572_V4_SLLVHLIGL KD: 1.26e-08 Kass: 1.06e+05, kdiss: 1.33e-03
3018_ID.573_A7_SLLQHLAGL KD: 5.30e-08 Kass: 1.05e+05, kdiss: 5.58e-03
3019_ID.575_W8_SLLQHLIWL KD:~ Kass:~ , kdiss: 1.02e-01
3020_ID.576_R3_SLRQHLIGL KD: 5.11e-07 Kass: 5.90e+04, kdiss: 3.01e-02
3021_ID.577_G3_SLGQHLIGL KD: 1.12e-08 Kass: 1.65e+05, kdiss: 1.86e-03
3022_ID.579_P1_PLLQHLIGL KD: 1.09e-08 Kass: 1.53e+05, kdiss: 1.67e-03
3023_ID.580_W5_SLLQWLIGL KD:~ Kass:~ , kdiss: 3.53e-02
3024_ID.581_F8_SLLQHLIFL KD:~ Kass:~ , kdiss: 2.32e-02
3025_ID.583_P8_SLLQHLIPL KD: 1.23e-08 Kass: 1.11e+05, kdiss: 1.38e-03
3026_ID.584_L5_SLLQLLIGL KD:~ Kass:~ , kdiss: 4.89e-02
3027_ID.585_R8_SLLQHLIRL KD: 9.43e-09 Kass: 1.03e+05, kdiss: 9.72e-04
3028_ID.586_N4_SLLNHLIGL KD: 9.64e-09 Kass: 1.38e+05, kdiss: 1.33e-03
3029_ID.587_A3_SLAQHLIGL KD: 1.07e-08 Kass: 1.47e+05, kdiss: 1.58e-03
3030_ID.589_I1_ILLQHLIGL KD: 1.09e-08 Kass: 1.40e+05, kdiss: 1.53e-03
3031_ID.59_W8_SLLQHLIWL KD:~ Kass:~ , kdiss: 1.05e-01
3032_ID.591_I8_SLLQHLIIL KD: 2.39e-08 Kass: 8.04e+04, kdiss: 1.93e-03
3033_ID.592_T5_SLLQTLIGL KD:~ Kass:~ , kdiss: 7.46e-02
3034_ID.595_R7_SLLQHLRGL KD:~ Kass:~ , kdiss: 9.08e-02
3035_ID.596_R5_SLLQRLIGL KD:~ Kass:~ , kdiss: 1.17e-01
3036_ID.597_W7_SLLQHLWGL KD: 1.18e-08 Kass: 1.46e+05, kdiss: 1.72e-03
3037_ID.598_I4_SLLIHLIGL KD: 1.12e-08 Kass: 1.52e+05, kdiss: 1.70e-03
3038_ID.599_V8_SLLQHLIVL KD: 2.76e-08 Kass: 8.55e+04, kdiss: 2.36e-03
3039_ID.601_W7_SLLQHLWGL KD:~ Kass:~ , kdiss: 2.11e-01
3040_ID.603_R6_SLLQHRIGL KD: 7.54e-09 Kass: 1.49e+05, kdiss: 1.12e-03
3041_ID.604_Y5_SLLQYLIGL KD: 2.82e-07 Kass: 5.71e+04, kdiss: 1.61e-02
3042_ID.605_Y7_SLLQHLYGL KD: 3.61e-07 Kass: 6.48e+04, kdiss: 2.34e-02
3043_ID.607_Y3_SLYQHLIGL KD: 4.34e-08 Kass: 1.24e+05, kdiss: 5.37e-03
3044_ID.608_T3_SLTQHLIGL KD: 1.04e-08 Kass: 1.63e+05, kdiss: 1.69e-03
3045_ID.61_T7_SLLQHLTGL KD: 7.29e-08 Kass: 6.70e+04, kdiss: 4.88e-03
3046_ID.611_R7_SLLQHLRGL KD:~ Kass:~ , kdiss: 5.88e-02
3047_ID.612_S3_SLSQHLIGL KD: 1.67e-08 Kass: 1.43e+05, kdiss: 2.39e-03
3048_ID.613_D7_SLLQHLDGL KD: 1.97e-08 Kass: 5.07e-01, kdiss: 1.00e-08
3049_ID.614_G6_SLLQHGIGL KD: 9.09e-09 Kass: 1.40e+05, kdiss: 1.27e-03
3050_ID.615_Y3_SLYQHLIGL KD: 4.28e-08 Kass: 1.14e+05, kdiss: 4.88e-03
3051_ID.616_T5_SLLQTLIGL KD:~ Kass:~ , kdiss: 5.42e-02
3052_ID.617_Q8_SLLQHLIQL KD: 3.00e-08 Kass: 7.58e+04, kdiss: 2.27e-03
3053_ID.619_I3_SLIQHLIGL KD: 8.56e-09 Kass: 1.19e+05, kdiss: 1.02e-03
3054_ID.62_E5_SLLQELIGL KD:~ Kass:~ , kdiss: 6.29e-02
3055_ID.623_W8_SLLQHLIWL KD:~ Kass:~ , kdiss: 5.01e-02
3056_ID.627_A7_SLLQHLAGL KD: 6.57e-08 Kass: 7.46e+04, kdiss: 4.90e-03
3057_ID.63_E7_SLLQHLEGL KD: 2.28e-08 Kass: 4.38e-01, kdiss: 1.00e-08
3058_ID.631_R6_SLLQHRIGL KD: 2.21e-08 Kass: 8.15e+04, kdiss: 1.80e-03
3059_ID.632_P5_SLLQPLIGL KD: 5.16e-07 Kass: 2.94e+04, kdiss: 1.52e-02
3060_ID.634_R4_SLLRHLIGL KD: 1.63e-08 Kass: 1.40e+05, kdiss: 2.28e-03
3061_ID.636_S5_SLLQSLIGL KD: 2.21e-07 Kass: 5.85e+04, kdiss: 1.29e-02
3062_ID.637_T7_SLLQHLTGL KD: 5.54e-08 Kass: 7.13e+04, kdiss: 3.95e-03
3063_ID.638_V6_SLLQHVIGL KD: 1.05e-08 Kass: 1.04e+05, kdiss: 1.09e-03
3064_ID.639_I3_SLIQHLIGL KD: 7.44e-09 Kass: 1.13e+05, kdiss: 8.38e-04
3065_ID.64_F4_SLLFHLIGL KD: 1.39e-08 Kass: 1.31e+05, kdiss: 1.82e-03
3066_ID.641_F3_SLFQHLIGL KD: 2.96e-08 Kass: 1.16e+05, kdiss: 3.44e-03
3067_ID.643_T6_SLLQHTIGL KD: 1.43e-08 Kass: 1.17e+05, kdiss: 1.68e-03
3068_ID.647_W1_WLLQHLIGL KD: 1.10e-09 Kass: 1.11e+05, kdiss: 1.22e-04
3069_ID.648_R3_SLRQHLIGL KD: 3.57e-07 Kass: 6.16e+04, kdiss: 2.20e-02
3070_ID.65_Q6_SLLQHQIGL KD: 5.61e-09 Kass: 1.25e+05, kdiss: 7.01e-04
3071_ID.650_H4_SLLHHLIGL KD: 8.81e-09 Kass: 1.10e+05, kdiss: 9.66e-04
3072_ID.651_G3_SLGQHLIGL KD: 1.01e-08 Kass: 1.52e+05, kdiss: 1.53e-03
3073_ID.652_T5_SLLQTLIGL KD:~ Kass:~ , kdiss: 5.20e-02
3074_ID.653_P7_SLLQHLPGL KD: 2.38e-07 Kass: 1.43e+04, kdiss: 3.40e-02
3075_ID.654_W5_SLLQWLIGL KD:~ Kass:~ , kdiss: 3.95e-02
3076_ID.655_W6_SLLQHWIGL KD: 9.52e-09 Kass: 1.22e+05, kdiss: 1.16e-03
3077_ID.656_M5_SLLQMLIGL KD:~ Kass:~ , kdiss: 4.62e-02
3078_ID.657_T6_SLLQHTIGL KD: 1.23e-08 Kass: 1.37e+05, kdiss: 1.69e-03
3079_ID.659_P3_SLPQHLIGL KD: 8.72e-09 Kass: 1.29e+05, kdiss: 1.13e-03
3080_ID.66_Y4_SLLYHLIGL KD: 8.37e-09 Kass: 1.28e+05, kdiss: 1.07e-03
3081_ID.660_K4_SLLKHLIGL KD: 9.24e-09 Kass: 1.86e+05, kdiss: 1.72e-03
3082_ID.663_M3_SLMQHLIGL KD: 1.27e-08 Kass: 1.39e+05, kdiss: 1.76e-03
3083_ID.664_A6_SLLQHAIGL KD: 9.57e-09 Kass: 1.53e+05, kdiss: 1.47e-03
3084_ID.665_W7_SLLQHLWGL KD: 1.19e-08 Kass: 1.40e+05, kdiss: 1.66e-03
3085_ID.667_I1_ILLQHLIGL KD: 9.63e-09 Kass: 1.44e+05, kdiss: 1.39e-03
3086_ID.669_R1_RLLQHLIGL KD: 8.30e-09 Kass: 1.58e+05, kdiss: 1.31e-03
3087_ID.67_Q1_QLLQHLIGL KD: 1.21e-08 Kass: 1.18e+05, kdiss: 1.43e-03
3088_ID.670_E4_SLLEHLIGL KD: 1.20e-08 Kass: 8.52e+04, kdiss: 1.03e-03
3089_ID.671_G1_GLLQHLIGL KD: 1.16e-08 Kass: 1.49e+05, kdiss: 1.73e-03
3090_ID.672_A4_SLLAHLIGL KD: 8.67e-09 Kass: 1.45e+05, kdiss: 1.26e-03
3091_ID.673_D7_SLLQHLDGL KD: 2.28e-08 Kass: 4.39e-01, kdiss: 1.00e-08
3092_ID.676_P5_SLLQPLIGL KD: 3.45e-07 Kass: 6.18e+04, kdiss: 2.13e-02
3093_ID.68_A6_SLLQHAIGL KD: 1.24e-08 Kass: 1.23e+05, kdiss: 1.53e-03
3094_ID.681_A1_ALLQHLIGL KD: 8.70e-09 Kass: 1.37e+05, kdiss: 1.19e-03
3095_ID.683_D8_SLLQHLIDL KD: 7.02e-08 Kass: 8.13e+04, kdiss: 5.70e-03
3096_ID.685_K7_SLLQHLKGL KD: 9.52e-08 Kass: 7.20e+04, kdiss: 6.86e-03
3097_ID.686_S3_SLSQHLIGL KD: 9.59e-09 Kass: 1.64e+05, kdiss: 1.57e-03
3098_ID.690_K4_SLLKHLIGL KD: 1.14e-08 Kass: 1.68e+05, kdiss: 1.91e-03
3099_ID.693_F7_SLLQHLFGL KD: 4.42e-08 Kass: 1.29e+05, kdiss: 5.72e-03
3100_ID.696_A6_SLLQHAIGL KD: 1.22e-08 Kass: 1.27e+05, kdiss: 1.56e-03
value
Time
0 250 500 750 1000
0 5000 10000 15000

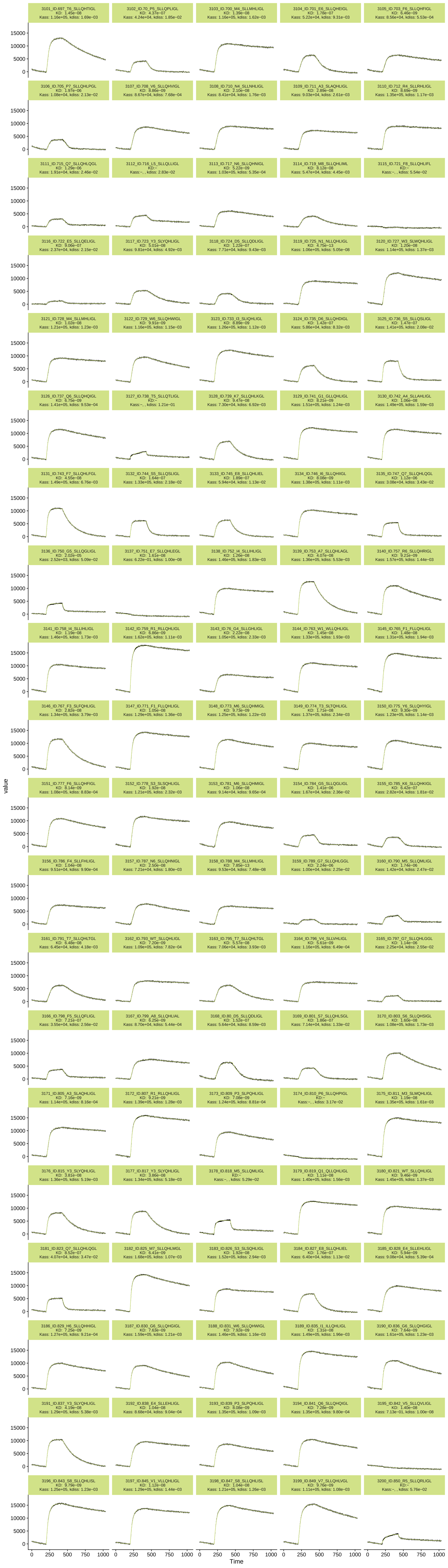
value
Time
0
5000
10000
15000
0
250
500
750
1000

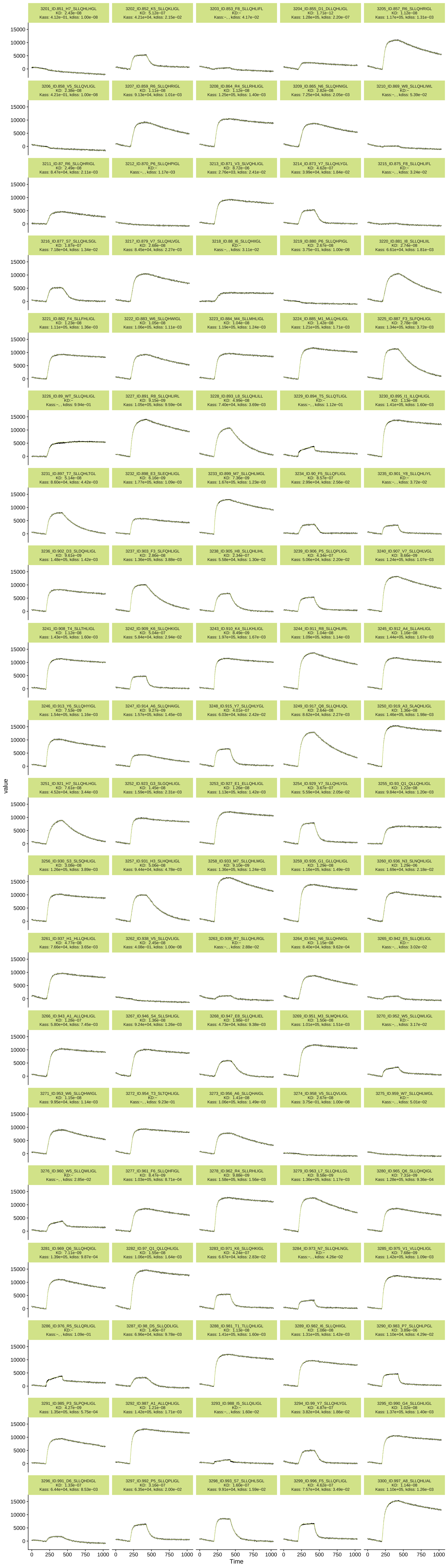
value
Time
0
5000
10000
15000
0
250
500
750
1000

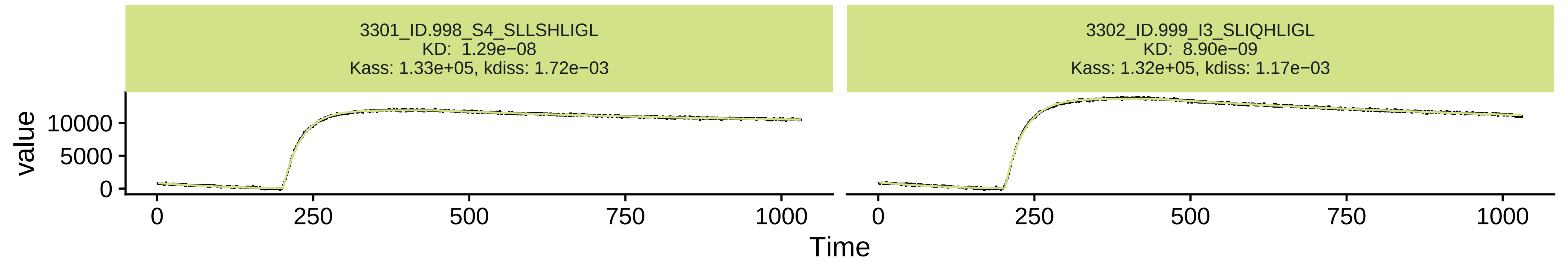
3301_ID.998_S4_SLLSHLIGL
KD: 1.29e−08
Kass: 1.33e+05, kdiss: 1.72e−03
3302_ID.999_I3_SLIQHLIGL
KD: 8.90e−09
Kass: 1.32e+05, kdiss: 1.17e−03
value
10000
5000
0
0
250
500
750
1000
Time
