# Supplementary material for: An ultra-high-throughput screen for the evaluation of peptide HLA-Binder interactions
Source: Sci Rep. 2023 Mar 31;13:5290. doi: 10.1038/s41598-023-32384-z (PMC10066226; doi:10.1038/s41598-023-32384-z)
Supplement: Supplementary file 2 — Supplementary Information 2. [file 41598_2023_32384_MOESM2_ESM.zip › raw_data_package/K01693_60nM/K01693_60nM_binding_curve_plots.pdf]

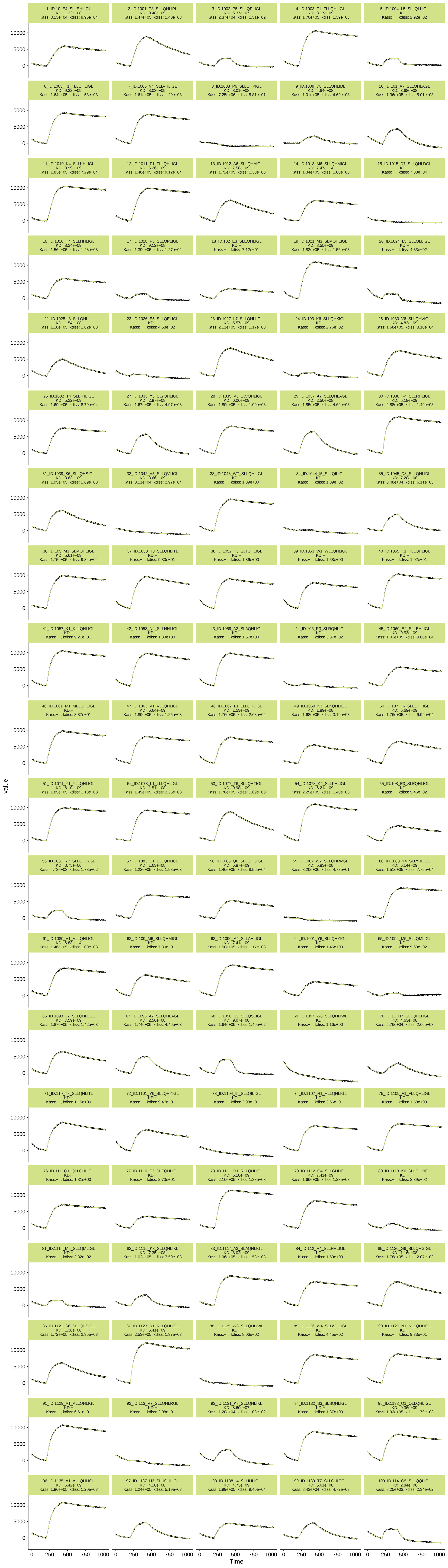

1_ID.10_E4_SLLEHLIGL
KD: 1.23e−08
Kass: 8.13e+04, kdiss: 9.96e−04
2_ID.1001_P8_SLLQHLIPL
KD: 9.48e−09
Kass: 1.47e+05, kdiss: 1.40e−03
3_ID.1002_P5_SLLQPLIGL
KD: 6.37e−07
Kass: 2.37e+04, kdiss: 1.51e−02
4_ID.1003_F1_FLLQHLIGL
KD: 8.17e−09
Kass: 1.70e+05, kdiss: 1.39e−03
5_ID.1004_L5_SLLQLLIGL
KD:−
Kass:−, , kdiss: 2.92e−02
6_ID.1005_T1_TLLQHLIGL
KD: 9.32e−09
Kass: 1.64e+05, kdiss: 1.53e−03
7_ID.1006_V4_SLLVHLIGL
KD: 8.03e−09
Kass: 1.61e+05, kdiss: 1.29e−03
8_ID.1008_P6_SLLQHPIGL
KD: 8.01e−08
Kass: 7.25e+06, kdiss: 5.81e−01
9_ID.1009_D8_SLLQHLIDL
KD: 4.64e−08
Kass: 1.01e+05, kdiss: 4.69e−03
10_ID.101_A7_SLLQHLAGL
KD: 3.68e−08
Kass: 1.36e+05, kdiss: 5.01e−03
11_ID.1010_K4_SLLKHLIGL
KD: 3.98e−09
Kass: 1.83e+05, kdiss: 7.29e−04
12_ID.1011_F1_FLLQHLIGL
KD: 6.26e−09
Kass: 1.46e+05, kdiss: 9.12e−04
13_ID.1012_A6_SLLQHAIGL
KD: 7.58e−09
Kass: 1.72e+05, kdiss: 1.30e−03
14_ID.1013_M6_SLLQHMIGL
KD: 7.47e−14
Kass: 1.34e+05, kdiss: 1.00e−08
15_ID.1015_D7_SLLQHLDGL
KD:−
Kass:−, , kdiss: 7.88e−04
16_ID.1016_H4_SLLHHLIGL
KD: 8.22e−09
Kass: 1.56e+05, kdiss: 1.28e−03
17_ID.1018_P5_SLLQPLIGL
KD: 9.12e−09
Kass: 1.39e+05, kdiss: 1.27e−02
18_ID.102_E3_SLEQHLIGL
KD:−
Kass:−, , kdiss: 7.12e−01
19_ID.1021_M3_SLMQHLIGL
KD: 8.55e−09
Kass: 1.83e+05, kdiss: 1.56e−03
20_ID.1024_L5_SLLQLLIGL
KD:−
Kass:−, , kdiss: 4.33e−02
21_ID.1025_I8_SLLQHLIIL
KD: 1.54e−08
Kass: 1.18e+05, kdiss: 1.82e−03
22_ID.1026_E5_SLLQELIGL
KD:−
Kass:−, , kdiss: 4.58e−02
23_ID.1027_L7_SLLQHLLGL
KD: 5.57e−09
Kass: 2.11e+05, kdiss: 1.17e−03
24_ID.103_K6_SLLQHKIGL
KD:−
Kass:−, , kdiss: 2.76e−02
25_ID.1030_V6_SLLQHVIGL
KD: 4.83e−09
Kass: 1.68e+05, kdiss: 8.10e−04
26_ID.1032_T4_SLLTHLIGL
KD: 5.22e−09
Kass: 1.69e+05, kdiss: 8.79e−04
27_ID.1033_Y3_SLYQHLIGL
KD: 2.97e−08
Kass: 1.67e+05, kdiss: 4.97e−03
28_ID.1035_V3_SLVQHLIGL
KD: 6.06e−09
Kass: 1.80e+05, kdiss: 1.09e−03
29_ID.1037_A7_SLLQHLAGL
KD: 2.50e−08
Kass: 1.85e+05, kdiss: 4.62e−03
30_ID.1038_R4_SLLRHLIGL
KD: 5.18e−09
Kass: 2.88e+05, kdiss: 1.49e−03
31_ID.1039_S6_SLLQHSIGL
KD: 8.63e−09
Kass: 1.95e+05, kdiss: 1.69e−03
32_ID.1042_V5_SLLQVLIGL
KD: 3.66e−06
Kass: 8.11e+04, kdiss: 2.97e−04
33_ID.1043_W7_SLLQHLWGL
KD:−
Kass:−, , kdiss: 1.39e+00
34_ID.1044_I5_SLLQILIGL
KD:−
Kass:−, , kdiss: 1.89e−02
35_ID.1045_D8_SLLQHLIDL
KD: 7.20e−08
Kass: 8.48e+04, kdiss: 6.11e−03
36_ID.105_M3_SLMQHLIGL
KD: 5.61e−09
Kass: 1.75e+05, kdiss: 9.84e−04
37_ID.1050_T8_SLLQHLITL
KD:−
Kass:−, , kdiss: 9.30e−01
38_ID.1052_T3_SLTQHLIGL
KD:−
Kass:−, , kdiss: 1.36e+00
39_ID.1053_W1_WLLQHLIGL
KD:−
Kass:−, , kdiss: 1.58e+00
40_ID.1055_K1_KLLQHLIGL
KD:−
Kass:−, , kdiss: 1.02e−01
41_ID.1057_K1_KLLQHLIGL
KD:−
Kass:−, , kdiss: 9.21e−01
42_ID.1058_N4_SLLNHLIGL
KD:−
Kass:−, , kdiss: 1.33e+00
43_ID.1059_A3_SLAQHLIGL
KD:−
Kass:−, , kdiss: 1.57e+00
44_ID.106_R3_SLRQHLIGL
KD:−
Kass:−, , kdiss: 3.37e−02
45_ID.1060_E4_SLLEHLIGL
KD: 9.53e−09
Kass: 1.01e+05, kdiss: 9.66e−04
46_ID.1061_M1_MLLQHLIGL
KD:−
Kass:−, , kdiss: 3.87e−01
47_ID.1063_V1_VLLQHLIGL
KD: 6.64e−09
Kass: 1.89e+05, kdiss: 1.25e−03
48_ID.1067_L1_LLLQHLIGL
KD: 1.53e−09
Kass: 1.76e+05, kdiss: 2.68e−04
49_ID.1069_K3_SLKQHLIGL
KD: 1.89e−08
Kass: 1.68e+05, kdiss: 3.19e−03
50_ID.107_F6_SLLQHFIGL
KD: 5.69e−09
Kass: 1.76e+05, kdiss: 9.99e−04
51_ID.1071_Y1_YLLQHLIGL
KD: 6.10e−09
Kass: 1.85e+05, kdiss: 1.13e−03
52_ID.1073_L1_LLLQHLIGL
KD: 1.51e−08
Kass: 1.49e+05, kdiss: 2.25e−03
53_ID.1077_T6_SLLQHTIGL
KD: 9.98e−09
Kass: 1.70e+05, kdiss: 1.69e−03
54_ID.1078_K4_SLLKHLIGL
KD: 6.21e−09
Kass: 2.25e+05, kdiss: 1.40e−03
55_ID.108_E3_SLEQHLIGL
KD:−
Kass:−, , kdiss: 5.46e−02
56_ID.1081_Y7_SLLQHLYGL
KD: 3.75e−06
Kass: 4.73e+03, kdiss: 1.78e−02
57_ID.1083_E1_ELLQHLIGL
KD: 1.63e−08
Kass: 1.22e+05, kdiss: 1.98e−03
58_ID.1085_Q6_SLLQHQIGL
KD: 5.87e−09
Kass: 1.46e+05, kdiss: 8.56e−04
59_ID.1087_W7_SLLQHLWGL
KD: 5.83e−08
Kass: 8.20e+06, kdiss: 4.78e−01
60_ID.1088_Y4_SLLYHLIGL
KD: 5.14e−09
Kass: 1.51e+05, kdiss: 7.75e−04
61_ID.1089_V1_VLLQHLIGL
KD: 6.83e−14
Kass: 1.46e+05, kdiss: 1.00e−08
62_ID.109_M6_SLLQHMIGL
KD:−
Kass:−, , kdiss: 7.80e−01
63_ID.1090_A4_SLLAHLIGL
KD: 7.41e−09
Kass: 1.58e+05, kdiss: 1.17e−03
64_ID.1091_Y6_SLLQHYIGL
KD:−
Kass:−, , kdiss: 1.45e+00
65_ID.1092_M5_SLLQMLIGL
KD:−
Kass:−, , kdiss: 5.63e−02
66_ID.1093_L7_SLLQHLLGL
KD: 7.59e−09
Kass: 1.87e+05, kdiss: 1.42e−03
67_ID.1095_A7_SLLQHLAGL
KD: 2.56e−08
Kass: 1.74e+05, kdiss: 4.46e−03
68_ID.1096_S5_SLLQSLIGL
KD: 9.07e−08
Kass: 1.64e+05, kdiss: 1.49e−02
69_ID.1097_W8_SLLQHLIWL
KD:−
Kass:−, , kdiss: 1.16e+00
70_ID.11_H7_SLLQHLHGL
KD: 4.63e−08
Kass: 5.76e+04, kdiss: 2.66e−03
71_ID.110_T8_SLLQHLITL
KD:−
Kass:−, , kdiss: 1.15e+00
72_ID.1101_Y6_SLLQHYIGL
KD:−
Kass:−, , kdiss: 9.47e−01
73_ID.1104_I5_SLLQILIGL
KD:−
Kass:−, , kdiss: 2.98e−01
74_ID.1107_H1_HLLQHLIGL
KD:−
Kass:−, , kdiss: 3.66e−01
75_ID.1109_F1_FLLQHLIGL
KD:−
Kass:−, , kdiss: 1.58e+00
76_ID.111_Q1_QLLQHLIGL
KD:−
Kass:−, , kdiss: 1.31e+00
77_ID.1110_E3_SLEQHLIGL
KD:−
Kass:−, , kdiss: 2.73e−01
78_ID.1111_R1_RLLQHLIGL
KD: 6.18e−09
Kass: 2.16e+05, kdiss: 1.33e−03
79_ID.1112_G4_SLLGHLIGL
KD: 7.41e−09
Kass: 1.66e+05, kdiss: 1.23e−03
80_ID.1113_K6_SLLQHKIGL
KD:−
Kass:−, , kdiss: 2.39e−02
81_ID.1114_M5_SLLQMLIGL
KD:−
Kass:−, , kdiss: 3.82e−02
82_ID.1115_K8_SLLQHLIKL
KD: 7.35e−08
Kass: 1.02e+05, kdiss: 7.50e−03
83_ID.1117_A3_SLAQHLIGL
KD: 8.02e−09
Kass: 1.96e+05, kdiss: 1.58e−03
84_ID.112_H4_SLLHHLIGL
KD:−
Kass:−, , kdiss: 1.59e+00
85_ID.1120_G6_SLLQHGIGL
KD: 1.16e−08
Kass: 1.78e+05, kdiss: 2.07e−03
86_ID.1121_S6_SLLQHSIGL
KD: 1.36e−08
Kass: 1.72e+05, kdiss: 2.35e−03
87_ID.1123_R1_RLLQHLIGL
KD: 5.41e−09
Kass: 2.53e+05, kdiss: 1.37e−03
88_ID.1125_W8_SLLQHLIWL
KD:−
Kass:−, , kdiss: 8.06e−02
89_ID.1126_W4_SLLWHLIGL
KD:−
Kass:−, , kdiss: 4.45e−02
90_ID.1127_N1_NLLQHLIGL
KD:−
Kass:−, , kdiss: 9.33e−01
91_ID.1129_A1_ALLQHLIGL
KD:−
Kass:−, , kdiss: 6.61e−01
92_ID.113_R7_SLLQHLRGL
KD:−
Kass:−, , kdiss: 2.08e−01
93_ID.1131_K8_SLLQHLIKL
KD: 8.60e−07
Kass: 1.20e+04, kdiss: 1.03e−02
94_ID.1132_S3_SLSQHLIGL
KD:−
Kass:−, , kdiss: 1.37e+00
95_ID.1133_Q1_QLLQHLIGL
KD: 9.36e−09
Kass: 1.92e+05, kdiss: 1.79e−03
96_ID.1135_A1_ALLQHLIGL
KD: 6.42e−09
Kass: 1.86e+05, kdiss: 1.20e−03
97_ID.1137_H3_SLHQHLIGL
KD: 4.18e−08
Kass: 1.24e+05, kdiss: 5.19e−03
98_ID.1138_I4_SLLIHLIGL
KD: 4.73e−09
Kass: 1.99e+05, kdiss: 9.40e−04
99_ID.1139_T7_SLLQHLTGL
KD: 5.61e−08
Kass: 8.42e+04, kdiss: 4.72e−03
100_ID.114_Q5_SLLQQLIGL
KD: 2.84e−06
Kass: 8.25e+03, kdiss: 2.34e−02
value
Time
0
5000
10000
250
500
750
1000

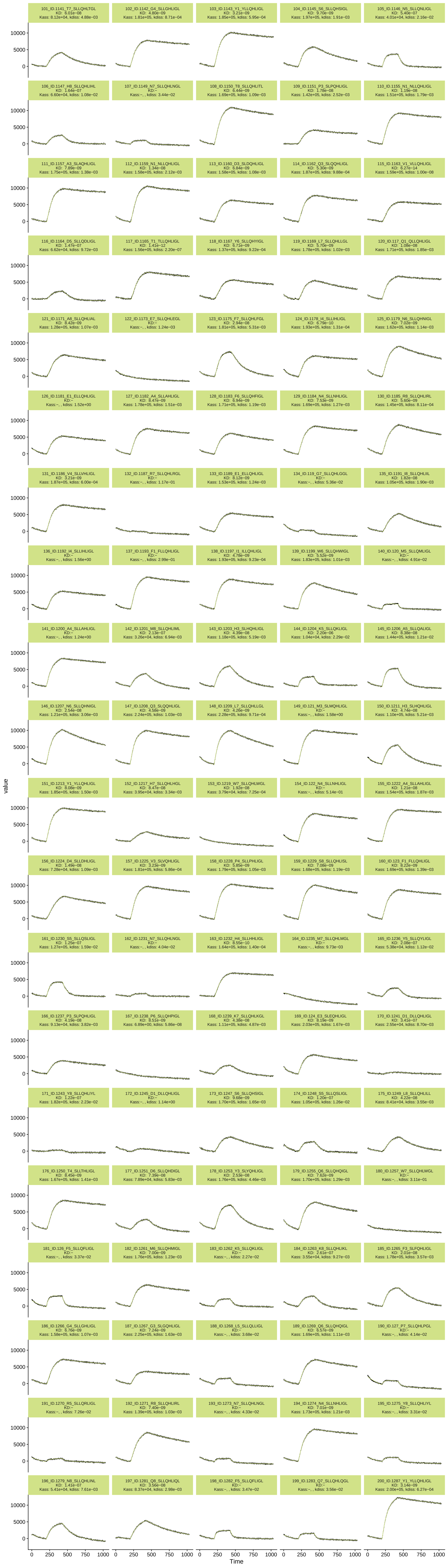
101_ID.1141_T7_SLLQHLTGL
KD: 6.01e−08
Kass: 8.12e+04, kdiss: 4.88e−03
102_ID.1142_G4_SLLGHLIGL
KD: 4.80e−09
Kass: 1.81e+05, kdiss: 8.71e−04
103_ID.1143_Y1_YLLQHLIGL
KD: 3.21e−09
Kass: 1.85e+05, kdiss: 5.95e−04
104_ID.1145_S6_SLLQHSIGL
KD: 9.70e−09
Kass: 1.97e+05, kdiss: 1.91e−03
105_ID.1146_N5_SLLQNLIGL
KD: 5.40e−07
Kass: 4.01e+04, kdiss: 2.16e−02
106_ID.1147_H8_SLLQHLIHL
KD: 1.64e−07
Kass: 6.60e+04, kdiss: 1.08e−02
107_ID.1149_N7_SLLQHLNGL
KD:−
Kass:−, , kdiss: 3.44e−02
108_ID.1150_T8_SLLQHLITL
KD: 6.44e−09
Kass: 1.69e+05, kdiss: 1.09e−03
109_ID.1151_P3_SLPQHLIGL
KD: 1.78e−08
Kass: 1.42e+05, kdiss: 2.52e−03
110_ID.1155_N1_NLLQHLIGL
KD: 1.19e−08
Kass: 1.51e+05, kdiss: 1.79e−03
111_ID.1157_A3_SLAQHLIGL
KD: 7.89e−09
Kass: 1.75e+05, kdiss: 1.38e−03
112_ID.1159_N1_NLLQHLIGL
KD: 1.34e−08
Kass: 1.58e+05, kdiss: 2.12e−03
113_ID.1160_D3_SLDQHLIGL
KD: 6.84e−09
Kass: 1.58e+05, kdiss: 1.08e−03
114_ID.1162_Q3_SLQQHLIGL
KD: 5.30e−09
Kass: 1.87e+05, kdiss: 9.88e−04
115_ID.1163_V1_VLLQHLIGL
KD: 6.27e−14
Kass: 1.59e+05, kdiss: 1.00e−08
116_ID.1164_D5_SLLQDLIGL
KD: 1.47e−07
Kass: 6.62e+04, kdiss: 9.72e−03
117_ID.1165_T1_TLLQHLIGL
KD: 1.41e−12
Kass: 1.56e+05, kdiss: 2.20e−07
118_ID.1167_Y6_SLLQHYIGL
KD: 6.71e−09
Kass: 1.37e+05, kdiss: 9.22e−04
119_ID.1169_L7_SLLQHLLGL
KD: 5.70e−09
Kass: 1.78e+05, kdiss: 1.02e−03
120_ID.117_Q1_QLLQHLIGL
KD: 1.08e−08
Kass: 1.71e+05, kdiss: 1.85e−03
121_ID.1171_A8_SLLQHLIAL
KD: 8.42e−09
Kass: 1.28e+05, kdiss: 1.07e−03
122_ID.1173_E7_SLLQHLEGL
KD:−
Kass:−, , kdiss: 1.24e−03
123_ID.1175_F7_SLLQHLFGL
KD: 2.94e−08
Kass: 1.81e+05, kdiss: 5.31e−03
124_ID.1178_I4_SLLIHLIGL
KD: 6.79e−10
Kass: 1.93e+05, kdiss: 1.31e−04
125_ID.1179_N6_SLLQHNIGL
KD: 7.02e−09
Kass: 1.62e+05, kdiss: 1.14e−03
126_ID.1181_E1_ELLQHLIGL
KD:−
Kass:−, , kdiss: 1.52e+00
127_ID.1182_A4_SLLAHLIGL
KD: 8.47e−09
Kass: 1.78e+05, kdiss: 1.51e−03
128_ID.1183_F6_SLLQHFIGL
KD: 6.94e−09
Kass: 1.71e+05, kdiss: 1.19e−03
129_ID.1184_N4_SLLNHLIGL
KD: 7.53e−09
Kass: 1.69e+05, kdiss: 1.27e−03
130_ID.1185_R8_SLLQHLIRL
KD: 5.60e−09
Kass: 1.45e+05, kdiss: 8.11e−04
131_ID.1186_V4_SLLVHLIGL
KD: 3.21e−09
Kass: 1.87e+05, kdiss: 6.00e−04
132_ID.1187_R7_SLLQHLRGL
KD:−
Kass:−, , kdiss: 1.17e−01
133_ID.1189_E1_ELLQHLIGL
KD: 8.12e−09
Kass: 1.53e+05, kdiss: 1.24e−03
134_ID.119_G7_SLLQHLGGL
KD:−
Kass:−, , kdiss: 5.36e−02
135_ID.1191_I8_SLLQHLIIL
KD: 1.82e−08
Kass: 1.05e+05, kdiss: 1.90e−03
136_ID.1192_I4_SLLIHLIGL
KD:−
Kass:−, , kdiss: 1.56e+00
137_ID.1193_F1_FLLQHLIGL
KD:−
Kass:−, , kdiss: 2.99e−01
138_ID.1197_I1_ILLQHLIGL
KD: 4.78e−09
Kass: 1.93e+05, kdiss: 9.23e−04
139_ID.1199_W6_SLLQHWIGL
KD: 5.52e−09
Kass: 1.83e+05, kdiss: 1.01e−03
140_ID.120_M5_SLLQMLIGL
KD:−
Kass:−, , kdiss: 4.91e−02
141_ID.1200_A4_SLLAHLIGL
KD:−
Kass:−, , kdiss: 1.24e+00
142_ID.1201_M8_SLLQHLIML
KD: 2.13e−07
Kass: 3.26e+04, kdiss: 6.94e−03
143_ID.1203_H3_SLHQHLIGL
KD: 4.39e−08
Kass: 1.18e+05, kdiss: 5.19e−03
144_ID.1204_K5_SLLQKLIGL
KD: 2.20e−06
Kass: 1.04e+04, kdiss: 2.29e−02
145_ID.1206_A5_SLLQALIGL
KD: 8.38e−08
Kass: 1.44e+05, kdiss: 1.21e−02
146_ID.1207_N6_SLLQHNIGL
KD: 2.54e−08
Kass: 1.21e+05, kdiss: 3.06e−03
147_ID.1208_Q3_SLQQHLIGL
KD: 4.58e−09
Kass: 2.24e+05, kdiss: 1.03e−03
148_ID.1209_L7_SLLQHLLGL
KD: 4.26e−09
Kass: 2.28e+05, kdiss: 9.71e−04
149_ID.121_M3_SLMQHLIGL
KD:−
Kass:−, , kdiss: 1.58e+00
150_ID.1211_H3_SLHQHLIGL
KD: 4.74e−08
Kass: 1.10e+05, kdiss: 5.21e−03
151_ID.1213_Y1_YLLQHLIGL
KD: 8.08e−09
Kass: 1.85e+05, kdiss: 1.50e−03
152_ID.1217_H7_SLLQHLHGL
KD: 8.47e−08
Kass: 3.95e+04, kdiss: 3.34e−03
153_ID.1219_W7_SLLQHLWGL
KD: 1.92e−08
Kass: 3.79e+04, kdiss: 7.25e−04
154_ID.122_N4_SLLNHLIGL
KD:−
Kass:−, , kdiss: 5.14e−01
155_ID.1222_A4_SLLAHLIGL
KD: 1.21e−08
Kass: 1.54e+05, kdiss: 1.87e−03
156_ID.1224_D4_SLLDHLIGL
KD: 1.49e−08
Kass: 7.28e+04, kdiss: 1.09e−03
157_ID.1225_V3_SLVQHLIGL
KD: 3.23e−09
Kass: 1.81e+05, kdiss: 5.86e−04
158_ID.1228_P4_SLLPHLIGL
KD: 5.85e−09
Kass: 1.79e+05, kdiss: 1.05e−03
159_ID.1229_S8_SLLQHLISL
KD: 7.08e−09
Kass: 1.68e+05, kdiss: 1.19e−03
160_ID.123_F1_FLLQHLIGL
KD: 8.22e−09
Kass: 1.69e+05, kdiss: 1.39e−03
161_ID.1230_S5_SLLQSLIGL
KD: 1.25e−07
Kass: 1.27e+05, kdiss: 1.59e−02
162_ID.1231_N7_SLLQHLNGL
KD:−
Kass:−, , kdiss: 4.04e−02
163_ID.1232_H4_SLLHHLIGL
KD: 8.55e−10
Kass: 1.64e+05, kdiss: 1.40e−04
164_ID.1235_M7_SLLQHLMGL
KD:−
Kass:−, , kdiss: 9.73e−03
165_ID.1236_Y5_SLLQYLIGL
KD: 2.08e−07
Kass: 5.38e+04, kdiss: 1.12e−02
166_ID.1237_P3_SLPQHLIGL
KD: 4.19e−08
Kass: 9.13e+04, kdiss: 3.82e−03
167_ID.1238_P6_SLLQHPIGL
KD: 8.51e−09
Kass: 6.89e+00, kdiss: 5.86e−08
168_ID.1239_K7_SLLQHLKGL
KD: 4.38e−08
Kass: 1.11e+05, kdiss: 4.87e−03
169_ID.124_E3_SLEQHLIGL
KD: 8.19e−09
Kass: 2.03e+05, kdiss: 1.67e−03
170_ID.1241_D1_DLLQHLIGL
KD: 3.41e−07
Kass: 2.55e+04, kdiss: 8.70e−03
171_ID.1243_Y8_SLLQHLIYL
KD: 1.22e−07
Kass: 1.82e+05, kdiss: 2.23e−02
172_ID.1245_D1_DLLQHLIGL
KD:−
Kass:−, , kdiss: 1.14e+00
173_ID.1247_S6_SLLQHSIGL
KD: 9.68e−09
Kass: 1.70e+05, kdiss: 1.65e−03
174_ID.1248_S5_SLLQSLIGL
KD: 1.20e−07
Kass: 1.05e+05, kdiss: 1.26e−02
175_ID.1249_L8_SLLQHLILL
KD: 4.22e−08
Kass: 8.41e+04, kdiss: 3.55e−03
176_ID.1250_T4_SLLTHLIGL
KD: 8.45e−09
Kass: 1.67e+05, kdiss: 1.41e−03
177_ID.1251_D6_SLLQHDIGL
KD: 7.39e−08
Kass: 7.89e+04, kdiss: 5.83e−03
178_ID.1253_Y3_SLYQHLIGL
KD: 2.53e−08
Kass: 1.76e+05, kdiss: 4.46e−03
179_ID.1255_Q6_SLLQHQIGL
KD: 7.62e−09
Kass: 1.70e+05, kdiss: 1.29e−03
180_ID.1257_W7_SLLQHLWGL
KD:−
Kass:−, , kdiss: 3.11e−01
181_ID.126_F5_SLLQFLIGL
KD:−
Kass:−, , kdiss: 3.37e−02
182_ID.1261_M6_SLLQHMIGL
KD: 7.00e−09
Kass: 1.76e+05, kdiss: 1.23e−03
183_ID.1262_K5_SLLQKLIGL
KD:−
Kass:−, , kdiss: 2.27e−02
184_ID.1263_K8_SLLQHLIKL
KD: 2.61e−07
Kass: 3.55e+04, kdiss: 9.27e−03
185_ID.1265_F3_SLFQHLIGL
KD: 2.01e−08
Kass: 1.78e+05, kdiss: 3.57e−03
186_ID.1266_G4_SLLGHLIGL
KD: 6.76e−09
Kass: 1.58e+05, kdiss: 1.07e−03
187_ID.1267_G3_SLGQHLIGL
KD: 7.24e−09
Kass: 2.25e+05, kdiss: 1.63e−03
188_ID.1268_L5_SLLQLLIGL
KD:−
Kass:−, , kdiss: 3.68e−02
189_ID.1269_Q6_SLLQHQIGL
KD: 6.57e−09
Kass: 1.69e+05, kdiss: 1.11e−03
190_ID.127_P7_SLLQHLPGL
KD:−
Kass:−, , kdiss: 4.14e−02
191_ID.1270_R5_SLLQRLIGL
KD:−
Kass:−, , kdiss: 7.26e−02
192_ID.1271_R8_SLLQHLIRL
KD: 7.40e−09
Kass: 1.39e+05, kdiss: 1.03e−03
193_ID.1273_N7_SLLQHLNGL
KD:−
Kass:−, , kdiss: 4.33e−02
194_ID.1274_N4_SLLNHLIGL
KD: 7.01e−09
Kass: 1.73e+05, kdiss: 1.21e−03
195_ID.1275_Y8_SLLQHLIYL
KD:−
Kass:−, , kdiss: 3.31e−02
196_ID.1279_N8_SLLQHLINL
KD: 1.41e−07
Kass: 5.41e+04, kdiss: 7.61e−03
197_ID.1281_Q8_SLLQHLIQL
KD: 3.56e−08
Kass: 8.37e+04, kdiss: 2.98e−03
198_ID.1282_F5_SLLQFLIGL
KD:−
Kass:−, , kdiss: 3.47e−02
199_ID.1283_Q7_SLLQHLQGL
KD:−
Kass:−, , kdiss: 3.56e−02
200_ID.1287_Y1_YLLQHLIGL
KD: 3.14e−09
Kass: 2.00e+05, kdiss: 6.27e−04
value
0
5000
10000
Time
0
250
500
750
1000

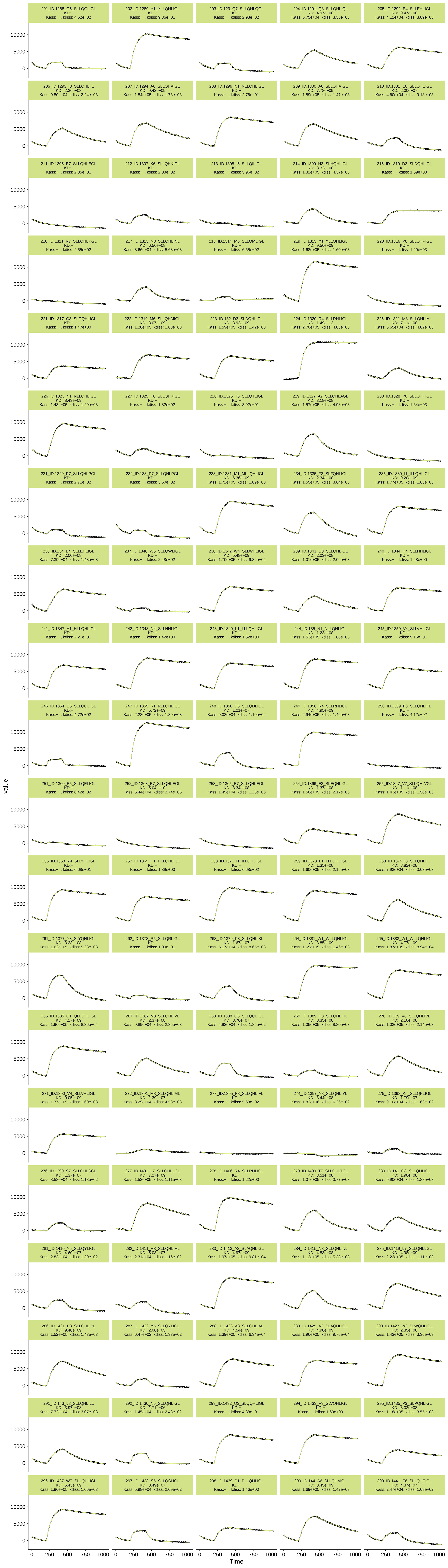
201_ID.1288_G5_SLLQGLIGL KD:– Kass:–, , kdiss: 4.62e–02
202_ID.1289_Y1_YLLQHLIGL KD:– Kass:–, , kdiss: 9.36e–01
203_ID.129_Q7_SLLQHLQGL KD:– Kass:–, , kdiss: 2.93e–02
204_ID.1291_Q8_SLLQHLIQL KD: 4.97e–08 Kass: 6.75e+04, kdiss: 3.35e–03
205_ID.1292_E4_SLLEHLIGL KD: 9.47e–08 Kass: 4.11e+04, kdiss: 3.89e–03
206_ID.1293_I8_SLLQHLIIL KD: 2.36e–08 Kass: 9.50e+04, kdiss: 2.24e–03
207_ID.1294_A6_SLLQHAIGL KD: 9.42e–09 Kass: 1.84e+05, kdiss: 1.73e–03
208_ID.1299_N1_NLLQHLIGL KD:– Kass:–, , kdiss: 2.76e–01
209_ID.1300_A6_SLLQHAIGL KD: 7.78e–09 Kass: 1.89e+05, kdiss: 1.47e–03
210_ID.1301_E6_SLLQHEIGL KD: 2.00e–07 Kass: 4.60e+04, kdiss: 9.18e–03
211_ID.1305_E7_SLLQHLEGL KD:– Kass:–, , kdiss: 2.85e–01
212_ID.1307_K6_SLLQHKIGL KD:– Kass:–, , kdiss: 2.08e–02
213_ID.1308_I5_SLLQILIGL KD:– Kass:–, , kdiss: 5.96e–02
214_ID.1309_H3_SLHQHLIGL KD: 3.32e–08 Kass: 1.31e+05, kdiss: 4.37e–03
215_ID.1310_D3_SLDQHLIGL KD:– Kass:–, , kdiss: 1.59e+00
216_ID.1311_R7_SLLQHLRGL KD:– Kass:–, , kdiss: 2.55e–02
217_ID.1313_N8_SLLQHLINL KD: 6.56e–08 Kass: 8.66e+04, kdiss: 5.68e–03
218_ID.1314_M5_SLLQMLIGL KD:– Kass:–, , kdiss: 6.65e–02
219_ID.1315_Y1_YLLQHLIGL KD: 9.56e–09 Kass: 1.68e+05, kdiss: 1.60e–03
220_ID.1316_P6_SLLQHPIGL KD:– Kass:–, , kdiss: 1.29e–03
221_ID.1317_G3_SLGQHLIGL KD:– Kass:–, , kdiss: 1.47e+00
222_ID.1319_M6_SLLQHMIGL KD: 8.07e–09 Kass: 1.28e+05, kdiss: 1.03e–03
223_ID.132_D3_SLDQHLIGL KD: 8.93e–09 Kass: 1.59e+05, kdiss: 1.42e–03
224_ID.1320_R4_SLLRHLIGL KD: 1.49e–13 Kass: 2.70e+05, kdiss: 4.03e–08
225_ID.1321_M8_SLLQHLIML KD: 7.11e–08 Kass: 5.65e+04, kdiss: 4.02e–03
226_ID.1323_N1_NLLQHLIGL KD: 8.43e–09 Kass: 1.43e+05, kdiss: 1.20e–03
227_ID.1325_K6_SLLQHKIGL KD:– Kass:–, , kdiss: 1.82e–02
228_ID.1326_T5_SLLQTLIGL KD:– Kass:–, , kdiss: 3.92e–01
229_ID.1327_A7_SLLQHLAGL KD: 3.18e–08 Kass: 1.57e+05, kdiss: 4.98e–03
230_ID.1328_P6_SLLQHPIGL KD:– Kass:–, , kdiss: 1.64e–03
231_ID.1329_P7_SLLQHLPGL KD:– Kass:–, , kdiss: 2.71e–02
232_ID.133_P7_SLLQHLPGL KD:– Kass:–, , kdiss: 3.60e–02
233_ID.1331_M1_MLLQHLIGL KD: 6.36e–09 Kass: 1.72e+05, kdiss: 1.09e–03
234_ID.1335_F3_SLFQHLIGL KD: 2.34e–08 Kass: 1.55e+05, kdiss: 3.64e–03
235_ID.1339_I1_ILLQHLIGL KD: 9.20e–09 Kass: 1.77e+05, kdiss: 1.63e–03
236_ID.134_E4_SLLEHLIGL KD: 2.00e–08 Kass: 7.39e+04, kdiss: 1.48e–03
237_ID.1340_W5_SLLQWLIGL KD:– Kass:–, , kdiss: 2.48e–02
238_ID.1342_W4_SLLWHLIGL KD: 5.48e–09 Kass: 1.70e+05, kdiss: 9.32e–04
239_ID.1343_Q8_SLLQHLIQL KD: 2.03e–08 Kass: 1.01e+05, kdiss: 2.06e–03
240_ID.1344_H4_SLLHHLIGL KD:– Kass:–, , kdiss: 1.48e+00
241_ID.1347_H1_HLLQHLIGL KD:– Kass:–, , kdiss: 2.21e–01
242_ID.1348_N4_SLLNHLIGL KD:– Kass:–, , kdiss: 1.42e+00
243_ID.1349_L1_LLLQHLIGL KD:– Kass:–, , kdiss: 1.52e+00
244_ID.135_N1_NLLQHLIGL KD: 1.23e–08 Kass: 1.53e+05, kdiss: 1.88e–03
245_ID.1350_V4_SLLVHLIGL KD:– Kass:–, , kdiss: 9.16e–01
246_ID.1354_G5_SLLQGLIGL KD:– Kass:–, , kdiss: 4.72e–02
247_ID.1355_R1_RLLQHLIGL KD: 5.72e–09 Kass: 2.28e+05, kdiss: 1.30e–03
248_ID.1356_D5_SLLQDLIGL KD: 1.21e–07 Kass: 9.02e+04, kdiss: 1.10e–02
249_ID.1358_R4_SLLRHLIGL KD: 4.95e–09 Kass: 2.94e+05, kdiss: 1.46e–03
250_ID.1359_F8_SLLQHLIFL KD:– Kass:–, , kdiss: 4.12e–02
251_ID.1360_E5_SLLQELIGL KD:– Kass:–, , kdiss: 8.42e–02
252_ID.1363_E7_SLLQHLEGL KD: 5.04e–10 Kass: 5.44e+04, kdiss: 2.74e–05
253_ID.1365_E7_SLLQHLEGL KD: 8.34e–08 Kass: 1.49e+04, kdiss: 1.25e–03
254_ID.1366_E3_SLEQHLIGL KD: 1.37e–08 Kass: 1.58e+05, kdiss: 2.17e–03
255_ID.1367_V7_SLLQHLVGL KD: 1.11e–08 Kass: 1.43e+05, kdiss: 1.58e–03
256_ID.1368_Y4_SLLYHLIGL KD:– Kass:–, , kdiss: 6.68e–01
257_ID.1369_H1_HLLQHLIGL KD:– Kass:–, , kdiss: 1.39e+00
258_ID.1371_I1_ILLQHLIGL KD:– Kass:–, , kdiss: 6.68e–02
259_ID.1373_L1_LLLQHLIGL KD: 1.35e–08 Kass: 1.60e+05, kdiss: 2.15e–03
260_ID.1375_I8_SLLQHLIIL KD: 3.82e–08 Kass: 7.93e+04, kdiss: 3.03e–03
261_ID.1377_Y3_SLYQHLIGL KD: 3.23e–08 Kass: 1.62e+05, kdiss: 5.23e–03
262_ID.1378_R5_SLLQRLIGL KD:– Kass:–, , kdiss: 1.09e–01
263_ID.1379_K8_SLLQHLIKL KD: 1.67e–07 Kass: 5.17e+04, kdiss: 8.65e–03
264_ID.1381_W1_WLLQHLIGL KD: 8.85e–09 Kass: 1.65e+05, kdiss: 1.46e–03
265_ID.1383_W1_WLLQHLIGL KD: 4.77e–09 Kass: 1.87e+05, kdiss: 8.94e–04
266_ID.1385_Q1_QLLQHLIGL KD: 4.27e–09 Kass: 1.96e+05, kdiss: 8.36e–04
267_ID.1387_V8_SLLQHLIVL KD: 2.37e–08 Kass: 9.89e+04, kdiss: 2.35e–03
268_ID.1388_Q5_SLLQQLIGL KD: 3.76e–07 Kass: 4.92e+04, kdiss: 1.85e–02
269_ID.1389_H8_SLLQHLIHL KD: 8.35e–08 Kass: 1.05e+05, kdiss: 8.80e–03
270_ID.139_V8_SLLQHLIVL KD: 2.10e–08 Kass: 1.02e+05, kdiss: 2.14e–03
271_ID.1390_V4_SLLVHLIGL KD: 9.05e–09 Kass: 1.77e+05, kdiss: 1.60e–03
272_ID.1391_M8_SLLQHLIML KD: 1.39e–07 Kass: 3.29e+04, kdiss: 4.58e–03
273_ID.1395_F8_SLLQHLIFL KD:– Kass:–, , kdiss: 5.63e–02
274_ID.1397_Y8_SLLQHLIYL KD: 3.44e–08 Kass: 1.82e+06, kdiss: 6.26e–02
275_ID.1398_K5_SLLQKLIGL KD: 1.79e–07 Kass: 9.10e+04, kdiss: 1.63e–02
276_ID.1399_S7_SLLQHLSGL KD: 1.37e–07 Kass: 8.58e+04, kdiss: 1.18e–02
277_ID.1401_L7_SLLQHLLGL KD: 7.27e–09 Kass: 1.53e+05, kdiss: 1.11e–03
278_ID.1406_R4_SLLRHLIGL KD:– Kass:–, , kdiss: 1.22e+00
279_ID.1409_T7_SLLQHLTGL KD: 3.51e–08 Kass: 1.07e+05, kdiss: 3.77e–03
280_ID.141_Q8_SLLQHLIQL KD: 1.90e–08 Kass: 9.90e+04, kdiss: 1.88e–03
281_ID.1410_Y5_SLLQYLIGL KD: 4.60e–07 Kass: 2.83e+04, kdiss: 1.30e–02
282_ID.1411_H8_SLLQHLIHL KD: 5.03e–07 Kass: 2.31e+04, kdiss: 1.16e–02
283_ID.1413_A3_SLAQHLIGL KD: 4.97e–09 Kass: 1.97e+05, kdiss: 9.81e–04
284_ID.1415_N8_SLLQHLINL KD: 4.83e–08 Kass: 1.12e+05, kdiss: 5.38e–03
285_ID.1419_L7_SLLQHLLGL KD: 4.98e–09 Kass: 2.22e+05, kdiss: 1.11e–03
286_ID.1421_P8_SLLQHLIPL KD: 9.40e–09 Kass: 1.52e+05, kdiss: 1.43e–03
287_ID.1422_Y5_SLLQYLIGL KD: 2.06e–05 Kass: 6.47e+02, kdiss: 1.33e–02
288_ID.1423_A8_SLLQHLIAL KD: 4.54e–09 Kass: 1.39e+05, kdiss: 6.34e–04
289_ID.1425_A3_SLAQHLIGL KD: 4.98e–09 Kass: 1.96e+05, kdiss: 9.76e–04
290_ID.1427_W3_SLWQHLIGL KD: 2.35e–08 Kass: 1.43e+05, kdiss: 3.36e–03
291_ID.143_L8_SLLQHLILL KD: 3.97e–08 Kass: 7.72e+04, kdiss: 3.07e–03
292_ID.1430_N5_SLLQNLIGL KD: 1.71e–06 Kass: 1.45e+04, kdiss: 2.48e–02
293_ID.1432_Q3_SLQQHLIGL KD:– Kass:–, , kdiss: 4.88e–01
294_ID.1433_V3_SLVQHLIGL KD:– Kass:–, , kdiss: 1.60e+00
295_ID.1435_P3_SLPQHLIGL KD: 3.02e–08 Kass: 1.18e+05, kdiss: 3.55e–03
296_ID.1437_WT_SLLQHLIGL KD: 5.43e–09 Kass: 1.96e+05, kdiss: 1.06e–03
297_ID.1438_S5_SLLQSLIGL KD: 3.49e–07 Kass: 5.98e+04, kdiss: 2.09e–02
298_ID.1439_P1_PLLQHLIGL KD:– Kass:–, , kdiss: 1.46e+00
299_ID.144_A6_SLLQHAIGL KD: 8.45e–09 Kass: 1.69e+05, kdiss: 1.42e–03
300_ID.1441_E6_SLLQHEIGL KD: 4.37e–07 Kass: 2.47e+04, kdiss: 1.08e–02
value
0
5000
10000
Time
0 250 500 750 1000

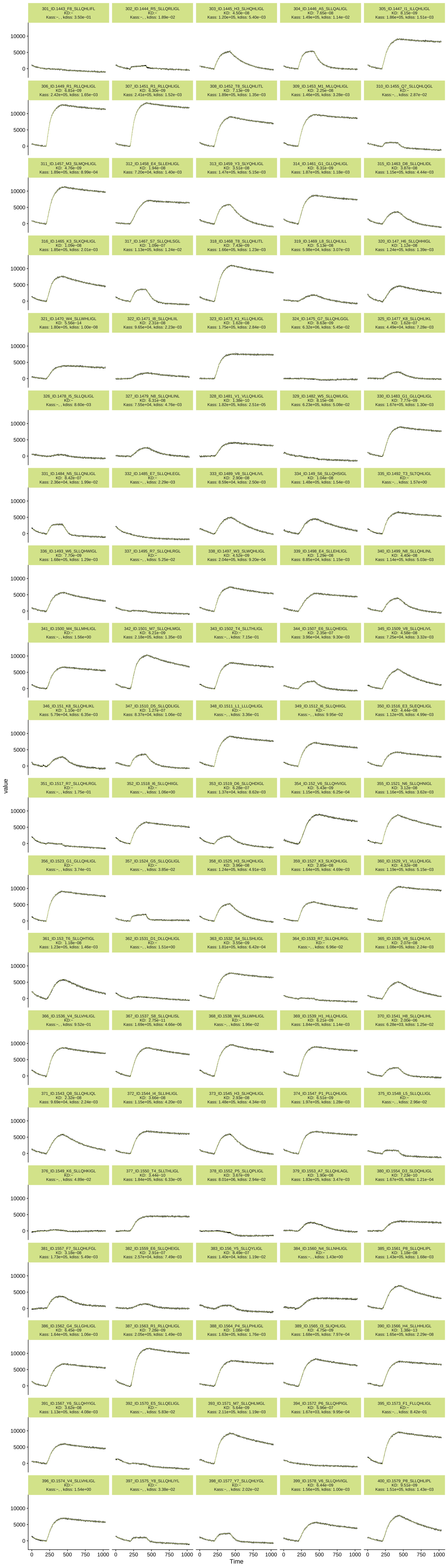

301_ID.1443_F8_SLLQHLIFL
KD:−
Kass:−, , kdiss: 3.50e−01
302_ID.1444_R5_SLLQRLIGL
KD:−
Kass:−, , kdiss: 1.89e−02
303_ID.1445_H3_SLHQHLIGL
KD: 4.50e−08
Kass: 1.20e+05, kdiss: 5.40e−03
304_ID.1446_A5_SLLQALIGL
KD: 7.65e−08
Kass: 1.49e+05, kdiss: 1.14e−02
305_ID.1447_I1_ILLQHLIGL
KD: 8.15e−09
Kass: 1.86e+05, kdiss: 1.51e−03
306_ID.1449_R1_RLLQHLIGL
KD: 6.81e−09
Kass: 2.42e+05, kdiss: 1.65e−03
307_ID.1451_R1_RLLQHLIGL
KD: 6.30e−09
Kass: 2.41e+05, kdiss: 1.52e−03
308_ID.1452_T8_SLLQHLITL
KD: 7.13e−09
Kass: 1.89e+05, kdiss: 1.35e−03
309_ID.1453_M1_MLLQHLIGL
KD: 2.25e−08
Kass: 1.46e+05, kdiss: 3.28e−03
310_ID.1455_Q7_SLLQHLQGL
KD:−
Kass:−, , kdiss: 2.87e−02
311_ID.1457_M3_SLMQHLIGL
KD: 4.76e−09
Kass: 1.89e+05, kdiss: 8.99e−04
312_ID.1458_E4_SLLEHLIGL
KD: 1.94e−08
Kass: 7.20e+04, kdiss: 1.40e−03
313_ID.1459_Y3_SLYQHLIGL
KD: 3.51e−08
Kass: 1.47e+05, kdiss: 5.15e−03
314_ID.1461_G1_GLLQHLIGL
KD: 6.31e−09
Kass: 1.87e+05, kdiss: 1.18e−03
315_ID.1463_D8_SLLQHLIDL
KD: 3.87e−08
Kass: 1.15e+05, kdiss: 4.44e−03
316_ID.1465_K3_SLKQHLIGL
KD: 1.09e−08
Kass: 1.85e+05, kdiss: 2.01e−03
317_ID.1467_S7_SLLQHLSGL
KD: 1.09e−07
Kass: 1.13e+05, kdiss: 1.24e−02
318_ID.1468_T8_SLLQHLITL
KD: 7.43e−09
Kass: 1.66e+05, kdiss: 1.23e−03
319_ID.1469_L8_SLLQHLILL
KD: 5.13e−08
Kass: 5.98e+04, kdiss: 3.07e−03
320_ID.147_H6_SLLQHHIGL
KD: 1.12e−08
Kass: 1.24e+05, kdiss: 1.39e−03
321_ID.1470_W4_SLLWHLIGL
KD: 5.56e−14
Kass: 1.80e+05, kdiss: 1.00e−08
322_ID.1471_I8_SLLQHLIIL
KD: 2.31e−08
Kass: 9.65e+04, kdiss: 2.23e−03
323_ID.1473_K1_KLLQHLIGL
KD: 1.62e−08
Kass: 1.75e+05, kdiss: 2.84e−03
324_ID.1475_G7_SLLQHLGGL
KD: 8.63e−09
Kass: 6.32e+06, kdiss: 5.45e−02
325_ID.1477_K8_SLLQHLIKL
KD: 1.62e−07
Kass: 4.49e+04, kdiss: 7.28e−03
326_ID.1478_I5_SLLQILIGL
KD:−
Kass:−, , kdiss: 8.60e−03
327_ID.1479_N8_SLLQHLINL
KD: 6.31e−08
Kass: 7.55e+04, kdiss: 4.76e−03
328_ID.1481_V1_VLLQHLIGL
KD: 1.38e−10
Kass: 1.82e+05, kdiss: 2.51e−05
329_ID.1482_W5_SLLQWLIGL
KD: 8.15e−08
Kass: 6.23e+05, kdiss: 5.08e−02
330_ID.1483_G1_GLLQHLIGL
KD: 7.77e−09
Kass: 1.67e+05, kdiss: 1.30e−03
331_ID.1484_N5_SLLQNLIGL
KD: 8.42e−07
Kass: 2.36e+04, kdiss: 1.99e−02
332_ID.1485_E7_SLLQHLEGL
KD:−
Kass:−, , kdiss: 2.29e−03
333_ID.1489_V8_SLLQHLIVL
KD: 2.90e−08
Kass: 8.59e+04, kdiss: 2.50e−03
334_ID.149_S6_SLLQHSIGL
KD: 1.04e−08
Kass: 1.48e+05, kdiss: 1.54e−03
335_ID.1492_T3_SLTQHLIGL
KD:−
Kass:−, , kdiss: 1.57e+00
336_ID.1493_W6_SLLQHWIGL
KD: 7.70e−09
Kass: 1.68e+05, kdiss: 1.29e−03
337_ID.1495_R7_SLLQHLRGL
KD:−
Kass:−, , kdiss: 5.25e−02
338_ID.1497_W3_SLWQHLIGL
KD: 4.52e−09
Kass: 2.04e+05, kdiss: 9.20e−04
339_ID.1498_E4_SLLEHLIGL
KD: 1.29e−08
Kass: 8.85e+04, kdiss: 1.15e−03
340_ID.1499_N8_SLLQHLINL
KD: 4.40e−08
Kass: 1.14e+05, kdiss: 5.03e−03
341_ID.1500_M4_SLLMHLIGL
KD:−
Kass:−, , kdiss: 1.56e+00
342_ID.1501_M7_SLLQHLMGL
KD: 6.21e−09
Kass: 2.18e+05, kdiss: 1.35e−03
343_ID.1502_T4_SLLTHLIGL
KD:−
Kass:−, , kdiss: 7.15e−01
344_ID.1507_E6_SLLQHEIGL
KD: 2.35e−07
Kass: 3.96e+04, kdiss: 9.30e−03
345_ID.1509_V8_SLLQHLIVL
KD: 4.58e−08
Kass: 7.25e+04, kdiss: 3.32e−03
346_ID.151_K8_SLLQHLIKL
KD: 1.10e−07
Kass: 5.79e+04, kdiss: 6.35e−03
347_ID.1510_D5_SLLQDLIGL
KD: 1.27e−07
Kass: 8.37e+04, kdiss: 1.06e−02
348_ID.1511_L1_LLLQHLIGL
KD:−
Kass:−, , kdiss: 3.36e−01
349_ID.1512_I6_SLLQHIIGL
KD:−
Kass:−, , kdiss: 9.95e−02
350_ID.1516_E3_SLEQHLIGL
KD: 4.44e−08
Kass: 1.12e+05, kdiss: 4.99e−03
351_ID.1517_R7_SLLQHLRGL
KD:−
Kass:−, , kdiss: 1.75e−01
352_ID.1518_I6_SLLQHIIGL
KD:−
Kass:−, , kdiss: 1.06e+00
353_ID.1519_D6_SLLQHDIGL
KD: 6.28e−07
Kass: 1.37e+04, kdiss: 8.62e−03
354_ID.152_V6_SLLQHVIGL
KD: 5.43e−09
Kass: 1.15e+05, kdiss: 6.25e−04
355_ID.1521_N6_SLLQHNIGL
KD: 3.12e−08
Kass: 1.16e+05, kdiss: 3.62e−03
356_ID.1523_G1_GLLQHLIGL
KD:−
Kass:−, , kdiss: 3.74e−01
357_ID.1524_G5_SLLQGLIGL
KD:−
Kass:−, , kdiss: 3.85e−02
358_ID.1525_H3_SLHQHLIGL
KD: 3.96e−08
Kass: 1.24e+05, kdiss: 4.91e−03
359_ID.1527_K3_SLKQHLIGL
KD: 2.85e−08
Kass: 1.64e+05, kdiss: 4.69e−03
360_ID.1529_V1_VLLQHLIGL
KD: 4.32e−08
Kass: 1.19e+05, kdiss: 5.15e−03
361_ID.153_T6_SLLQHTIGL
KD: 1.18e−08
Kass: 1.23e+05, kdiss: 1.46e−03
362_ID.1531_D1_DLLQHLIGL
KD:−
Kass:−, , kdiss: 1.51e+00
363_ID.1532_S4_SLLSHLIGL
KD: 3.55e−09
Kass: 1.81e+05, kdiss: 6.42e−04
364_ID.1533_R7_SLLQHLRGL
KD:−
Kass:−, , kdiss: 6.96e−02
365_ID.1535_V8_SLLQHLIVL
KD: 2.07e−08
Kass: 1.08e+05, kdiss: 2.24e−03
366_ID.1536_V4_SLLVHLIGL
KD:−
Kass:−, , kdiss: 9.52e−01
367_ID.1537_S8_SLLQHLISL
KD: 2.75e−11
Kass: 1.69e+05, kdiss: 4.66e−06
368_ID.1538_W4_SLLWHLIGL
KD:−
Kass:−, , kdiss: 1.96e−02
369_ID.1539_H1_HLLQHLIGL
KD: 6.21e−09
Kass: 1.84e+05, kdiss: 1.14e−03
370_ID.1541_H8_SLLQHLIHL
KD: 2.00e−06
Kass: 6.28e+03, kdiss: 1.25e−02
371_ID.1543_Q8_SLLQHLIQL
KD: 2.32e−08
Kass: 9.69e+04, kdiss: 2.24e−03
372_ID.1544_I4_SLLIHLIGL
KD: 3.66e−08
Kass: 1.15e+05, kdiss: 4.20e−03
373_ID.1545_H3_SLHQHLIGL
KD: 2.93e−08
Kass: 1.48e+05, kdiss: 4.34e−03
374_ID.1547_P1_PLLQHLIGL
KD: 6.51e−09
Kass: 1.97e+05, kdiss: 1.28e−03
375_ID.1548_L5_SLLQLLIGL
KD:−
Kass:−, , kdiss: 2.96e−02
376_ID.1549_K6_SLLQHKIGL
KD:−
Kass:−, , kdiss: 4.89e−02
377_ID.1550_T4_SLLTHLIGL
KD: 3.44e−10
Kass: 1.84e+05, kdiss: 6.33e−05
378_ID.1552_P5_SLLQPLIGL
KD: 3.67e−09
Kass: 8.01e+06, kdiss: 2.94e−02
379_ID.1553_A7_SLLQHLAGL
KD: 1.90e−08
Kass: 1.83e+05, kdiss: 3.47e−03
380_ID.1554_D3_SLDQHLIGL
KD: 7.23e−10
Kass: 1.67e+05, kdiss: 1.21e−04
381_ID.1557_F7_SLLQHLFGL
KD: 3.18e−08
Kass: 1.73e+05, kdiss: 5.49e−03
382_ID.1559_E6_SLLQHEIGL
KD: 2.91e−07
Kass: 2.57e+04, kdiss: 7.49e−03
383_ID.156_Y5_SLLQYLIGL
KD: 8.49e−07
Kass: 1.40e+04, kdiss: 1.19e−02
384_ID.1560_N4_SLLNHLIGL
KD:−
Kass:−, , kdiss: 1.43e+00
385_ID.1561_P8_SLLQHLIPL
KD: 1.18e−08
Kass: 1.43e+05, kdiss: 1.68e−03
386_ID.1562_G4_SLLGHLIGL
KD: 6.45e−09
Kass: 1.64e+05, kdiss: 1.06e−03
387_ID.1563_R1_RLLQHLIGL
KD: 7.28e−09
Kass: 2.05e+05, kdiss: 1.49e−03
388_ID.1564_P4_SLLPHLIGL
KD: 1.08e−08
Kass: 1.63e+05, kdiss: 1.76e−03
389_ID.1565_I3_SLIQHLIGL
KD: 4.75e−09
Kass: 1.68e+05, kdiss: 7.97e−04
390_ID.1566_H4_SLLHHLIGL
KD: 1.38e−13
Kass: 1.65e+05, kdiss: 2.29e−08
391_ID.1567_Y6_SLLQHYIGL
KD: 3.62e−08
Kass: 1.13e+05, kdiss: 4.08e−03
392_ID.1570_E5_SLLQELIGL
KD:−
Kass:−, , kdiss: 5.83e−02
393_ID.1571_M7_SLLQHLMGL
KD: 5.64e−09
Kass: 2.11e+05, kdiss: 1.19e−03
394_ID.1572_P6_SLLQHPIGL
KD: 5.96e−07
Kass: 1.67e+03, kdiss: 9.95e−04
395_ID.1573_F1_FLLQHLIGL
KD:−
Kass:−, , kdiss: 8.42e−01
396_ID.1574_V4_SLLVHLIGL
KD:−
Kass:−, , kdiss: 1.54e+00
397_ID.1575_Y8_SLLQHLIYL
KD:−
Kass:−, , kdiss: 3.38e−02
398_ID.1577_Y7_SLLQHLYGL
KD:−
Kass:−, , kdiss: 2.02e−02
399_ID.1578_V6_SLLQHVIGL
KD: 6.44e−09
Kass: 1.56e+05, kdiss: 1.00e−03
400_ID.1579_P8_SLLQHLIPL
KD: 9.51e−09
Kass: 1.51e+05, kdiss: 1.43e−03
value
10000
5000
0
0
250
500
750
1000
Time

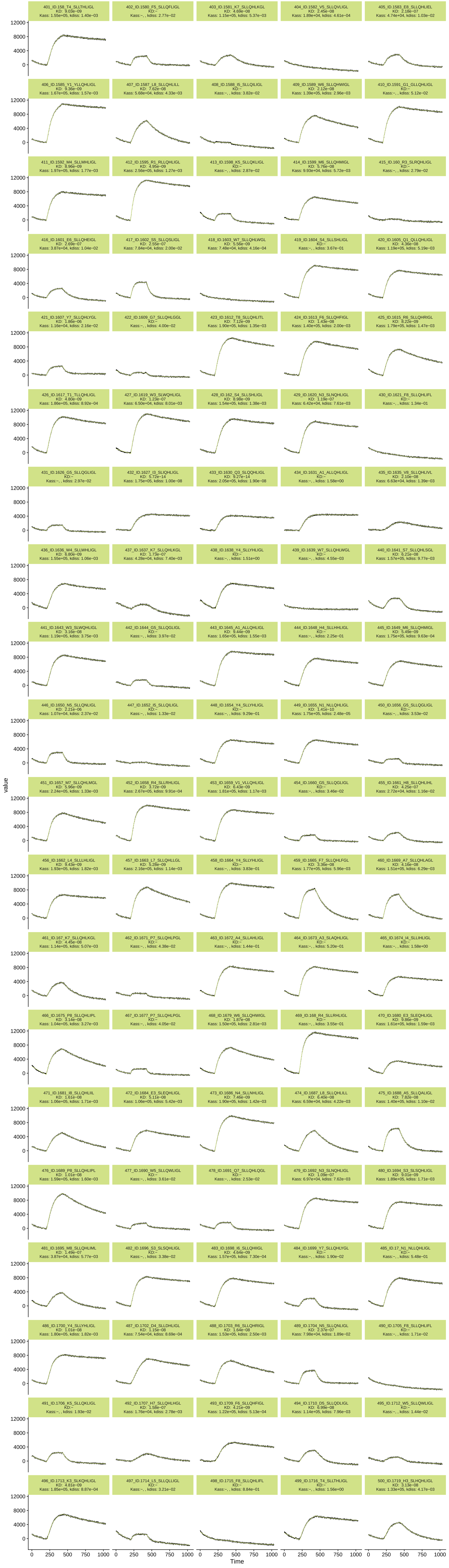

401_ID.158_T4_SLLTHLIGL KD: 9.03e−09 Kass: 1.55e+05, kdiss: 1.40e−03
402_ID.1580_F5_SLLQFLIGL KD:− Kass:−, , kdiss: 2.77e−02
403_ID.1581_K7_SLLQHLKGL KD: 4.69e−08 Kass: 1.15e+05, kdiss: 5.37e−03
404_ID.1582_V5_SLLQVLIGL KD: 2.45e−08 Kass: 1.89e+04, kdiss: 4.61e−04
405_ID.1583_E8_SLLQHLIEL KD: 2.18e−07 Kass: 4.74e+04, kdiss: 1.03e−02
406_ID.1585_Y1_YLLQHLIGL KD: 9.36e−09 Kass: 1.67e+05, kdiss: 1.57e−03
407_ID.1587_L8_SLLQHLILL KD: 7.62e−08 Kass: 5.68e+04, kdiss: 4.33e−03
408_ID.1588_I5_SLLQILIGL KD:− Kass:−, , kdiss: 3.82e−02
409_ID.1589_W6_SLLQHWIGL KD: 2.12e−08 Kass: 1.39e+05, kdiss: 2.96e−03
410_ID.1591_G1_GLLQHLIGL KD:− Kass:−, , kdiss: 5.12e−02
411_ID.1592_M4_SLLMHLIGL KD: 8.96e−09 Kass: 1.97e+05, kdiss: 1.77e−03
412_ID.1595_R1_RLLQHLIGL KD: 4.95e−09 Kass: 2.56e+05, kdiss: 1.27e−03
413_ID.1598_K5_SLLQKLIGL KD:− Kass:−, , kdiss: 2.87e−02
414_ID.1599_M6_SLLQHMIGL KD: 5.76e−08 Kass: 9.93e+04, kdiss: 5.72e−03
415_ID.160_R3_SLRQHLIGL KD:− Kass:−, , kdiss: 2.79e−02
416_ID.1601_E6_SLLQHEIGL KD: 2.69e−07 Kass: 3.87e+04, kdiss: 1.04e−02
417_ID.1602_S5_SLLQSLIGL KD: 2.55e−07 Kass: 7.84e+04, kdiss: 2.00e−02
418_ID.1603_W7_SLLQHLWGL KD: 5.56e−09 Kass: 7.48e+04, kdiss: 4.16e−04
419_ID.1604_S4_SLLSHLIGL KD:− Kass:−, , kdiss: 3.67e−01
420_ID.1605_Q1_QLLQHLIGL KD: 4.36e−08 Kass: 1.19e+05, kdiss: 5.19e−03
421_ID.1607_Y7_SLLQHLYGL KD: 1.86e−06 Kass: 1.16e+04, kdiss: 2.16e−02
422_ID.1609_G7_SLLQHLGGL KD:− Kass:−, , kdiss: 4.00e−02
423_ID.1612_T8_SLLQHLITL KD: 7.12e−09 Kass: 1.90e+05, kdiss: 1.35e−03
424_ID.1613_F6_SLLQHFIGL KD: 1.43e−08 Kass: 1.40e+05, kdiss: 2.00e−03
425_ID.1615_R6_SLLQHRIGL KD: 8.22e−09 Kass: 1.79e+05, kdiss: 1.47e−03
426_ID.1617_T1_TLLQHLIGL KD: 4.80e−09 Kass: 1.86e+05, kdiss: 8.92e−04
427_ID.1619_W3_SLWQHLIGL KD: 1.23e−07 Kass: 6.50e+04, kdiss: 8.01e−03
428_ID.162_S4_SLLSHLIGL KD: 8.98e−09 Kass: 1.54e+05, kdiss: 1.38e−03
429_ID.1620_N3_SLNQHLIGL KD: 1.19e−07 Kass: 6.42e+04, kdiss: 7.61e−03
430_ID.1621_F8_SLLQHLIFL KD:− Kass:−, , kdiss: 1.34e−01
431_ID.1626_G5_SLLQGLIGL KD:− Kass:−, , kdiss: 2.97e−02
432_ID.1627_I3_SLIQHLIGL KD: 5.72e−14 Kass: 1.75e+05, kdiss: 1.00e−08
433_ID.1630_Q3_SLQQHLIGL KD: 9.27e−14 Kass: 2.05e+05, kdiss: 1.90e−08
434_ID.1631_A1_ALLQHLIGL KD:− Kass:−, , kdiss: 1.58e+00
435_ID.1635_V8_SLLQHLIVL KD: 2.10e−08 Kass: 6.63e+04, kdiss: 1.39e−03
436_ID.1636_W4_SLLWHLIGL KD: 6.80e−09 Kass: 1.55e+05, kdiss: 1.06e−03
437_ID.1637_K7_SLLQHLKGL KD: 1.73e−07 Kass: 4.28e+04, kdiss: 7.40e−03
438_ID.1638_Y4_SLLYHLIGL KD:− Kass:−, , kdiss: 1.51e+00
439_ID.1639_W7_SLLQHLWGL KD:− Kass:−, , kdiss: 4.55e−03
440_ID.1641_S7_SLLQHLSGL KD: 6.21e−08 Kass: 1.57e+05, kdiss: 9.77e−03
441_ID.1643_W3_SLWQHLIGL KD: 3.16e−08 Kass: 1.19e+05, kdiss: 3.75e−03
442_ID.1644_G5_SLLQGLIGL KD:− Kass:−, , kdiss: 3.97e−02
443_ID.1645_A1_ALLQHLIGL KD: 9.44e−09 Kass: 1.65e+05, kdiss: 1.55e−03
444_ID.1648_H4_SLLHHLIGL KD:− Kass:−, , kdiss: 2.25e−01
445_ID.1649_M6_SLLQHMIGL KD: 5.49e−09 Kass: 1.75e+05, kdiss: 9.63e−04
446_ID.1650_N5_SLLQNLIGL KD: 2.21e−06 Kass: 1.07e+04, kdiss: 2.37e−02
447_ID.1652_I5_SLLQILIGL KD:− Kass:−, , kdiss: 1.33e−02
448_ID.1654_Y4_SLLYHLIGL KD:− Kass:−, , kdiss: 9.29e−01
449_ID.1655_N1_NLLQHLIGL KD: 1.41e−10 Kass: 1.75e+05, kdiss: 2.48e−05
450_ID.1656_G5_SLLQGLIGL KD:− Kass:−, , kdiss: 3.53e−02
451_ID.1657_M7_SLLQHLMGL KD: 5.96e−09 Kass: 2.24e+05, kdiss: 1.33e−03
452_ID.1658_R4_SLLRHLIGL KD: 3.72e−09 Kass: 2.67e+05, kdiss: 9.91e−04
453_ID.1659_V1_VLLQHLIGL KD: 6.43e−09 Kass: 1.81e+05, kdiss: 1.17e−03
454_ID.1660_G5_SLLQGLIGL KD:− Kass:−, , kdiss: 3.46e−02
455_ID.1661_H8_SLLQHLIHL KD: 4.25e−07 Kass: 2.72e+04, kdiss: 1.16e−02
456_ID.1662_L4_SLLLHLIGL KD: 9.43e−09 Kass: 1.93e+05, kdiss: 1.82e−03
457_ID.1663_L7_SLLQHLLGL KD: 5.28e−09 Kass: 2.16e+05, kdiss: 1.14e−03
458_ID.1664_Y4_SLLYHLIGL KD:− Kass:−, , kdiss: 3.83e−01
459_ID.1665_F7_SLLQHLFGL KD: 3.36e−08 Kass: 1.77e+05, kdiss: 5.96e−03
460_ID.1669_A7_SLLQHLAGL KD: 4.16e−08 Kass: 1.51e+05, kdiss: 6.29e−03
461_ID.167_K7_SLLQHLKGL KD: 4.45e−08 Kass: 1.14e+05, kdiss: 5.07e−03
462_ID.1671_P7_SLLQHLPGL KD:− Kass:−, , kdiss: 4.38e−02
463_ID.1672_A4_SLLAHLIGL KD:− Kass:−, , kdiss: 1.44e−01
464_ID.1673_A3_SLAQHLIGL KD:− Kass:−, , kdiss: 5.20e−01
465_ID.1674_I4_SLLIHLIGL KD:− Kass:−, , kdiss: 1.58e+00
466_ID.1675_P8_SLLQHLIPL KD: 3.14e−08 Kass: 1.04e+05, kdiss: 3.27e−03
467_ID.1677_P7_SLLQHLPGL KD:− Kass:−, , kdiss: 4.05e−02
468_ID.1679_W6_SLLQHWIGL KD: 1.87e−08 Kass: 1.50e+05, kdiss: 2.81e−03
469_ID.168_R4_SLLRHLIGL KD:− Kass:−, , kdiss: 3.55e−01
470_ID.1680_E3_SLEQHLIGL KD: 9.86e−09 Kass: 1.61e+05, kdiss: 1.59e−03
471_ID.1681_I8_SLLQHLIIL KD: 1.61e−08 Kass: 1.06e+05, kdiss: 1.71e−03
472_ID.1684_E3_SLEQHLIGL KD: 5.11e−08 Kass: 1.06e+05, kdiss: 5.42e−03
473_ID.1686_N4_SLLNHLIGL KD: 7.46e−09 Kass: 1.90e+05, kdiss: 1.42e−03
474_ID.1687_L8_SLLQHLILL KD: 6.40e−08 Kass: 6.59e+04, kdiss: 4.22e−03
475_ID.1688_A5_SLLQALIGL KD: 7.82e−08 Kass: 1.40e+05, kdiss: 1.10e−02
476_ID.1689_P8_SLLQHLIPL KD: 1.01e−08 Kass: 1.59e+05, kdiss: 1.60e−03
477_ID.1690_W5_SLLQWLIGL KD:− Kass:−, , kdiss: 3.61e−02
478_ID.1691_Q7_SLLQHLQGL KD:− Kass:−, , kdiss: 2.53e−02
479_ID.1692_N3_SLNQHLIGL KD: 1.09e−07 Kass: 6.97e+04, kdiss: 7.62e−03
480_ID.1694_S3_SLSQHLIGL KD: 9.01e−09 Kass: 1.89e+05, kdiss: 1.71e−03
481_ID.1695_M8_SLLQHLIML KD: 1.49e−07 Kass: 3.87e+04, kdiss: 5.77e−03
482_ID.1696_S3_SLSQHLIGL KD:− Kass:−, , kdiss: 3.38e−02
483_ID.1698_I6_SLLQHIIGL KD: 4.64e−09 Kass: 1.57e+05, kdiss: 7.30e−04
484_ID.1699_Y7_SLLQHLYGL KD:− Kass:−, , kdiss: 1.90e−02
485_ID.17_N1_NLLQHLIGL KD:− Kass:−, , kdiss: 5.48e−01
486_ID.1700_Y4_SLLYHLIGL KD: 1.01e−08 Kass: 1.80e+05, kdiss: 1.82e−03
487_ID.1702_D4_SLLDHLIGL KD: 1.15e−08 Kass: 7.54e+04, kdiss: 8.69e−04
488_ID.1703_R6_SLLQHRIGL KD: 1.64e−08 Kass: 1.53e+05, kdiss: 2.50e−03
489_ID.1704_N5_SLLQNLIGL KD: 2.37e−07 Kass: 7.98e+04, kdiss: 1.89e−02
490_ID.1705_F8_SLLQHLIFL KD:− Kass:−, , kdiss: 1.71e−02
491_ID.1706_K5_SLLQKLIGL KD:− Kass:−, , kdiss: 1.93e−02
492_ID.1707_H7_SLLQHLHGL KD: 1.58e−07 Kass: 1.76e+04, kdiss: 2.78e−03
493_ID.1709_F6_SLLQHFIGL KD: 4.21e−09 Kass: 1.22e+05, kdiss: 5.13e−04
494_ID.1710_D5_SLLQDLIGL KD: 6.99e−08 Kass: 1.14e+05, kdiss: 7.96e−03
495_ID.1712_W5_SLLQWLIGL KD:− Kass:−, , kdiss: 1.44e−02
496_ID.1713_K3_SLKQHLIGL KD: 4.81e−09 Kass: 1.85e+05, kdiss: 8.87e−04
497_ID.1714_L5_SLLQLLIGL KD:− Kass:−, , kdiss: 3.21e−02
498_ID.1715_F8_SLLQHLIFL KD:− Kass:−, , kdiss: 8.84e−01
499_ID.1716_T4_SLLTHLIGL KD:− Kass:−, , kdiss: 1.56e+00
500_ID.1719_H3_SLHQHLIGL KD: 3.13e−08 Kass: 1.33e+05, kdiss: 4.17e−03
value
12000
8000
4000
0
0 250 500 750 1000
Time

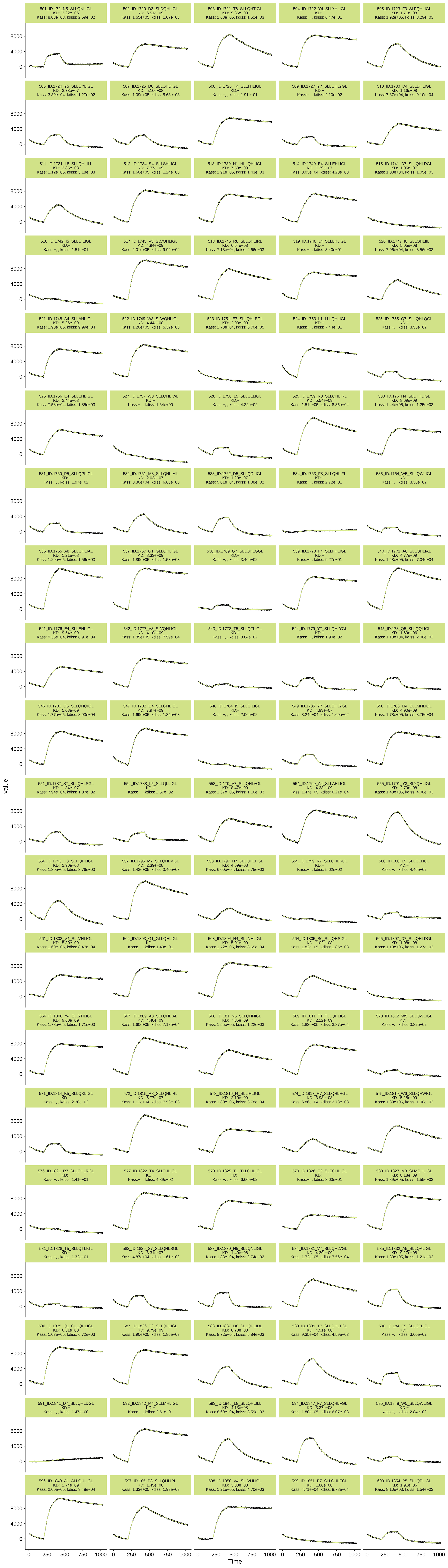

501_ID.172_N5_SLLQNLIGL
KD: 3.22e−06
Kass: 8.03e+03, kdiss: 2.59e−02
502_ID.1720_D3_SLDQHLIGL
KD: 6.51e−09
Kass: 1.65e+05, kdiss: 1.07e−03
503_ID.1721_T6_SLLQHTIGL
KD: 9.36e−09
Kass: 1.63e+05, kdiss: 1.52e−03
504_ID.1722_Y4_SLLYHLIGL
KD:−
Kass:−, , kdiss: 6.47e−01
505_ID.1723_F3_SLFQHLIGL
KD: 1.71e−08
Kass: 1.92e+05, kdiss: 3.29e−03
506_ID.1724_Y5_SLLQYLIGL
KD: 3.73e−07
Kass: 3.39e+04, kdiss: 1.27e−02
507_ID.1725_D6_SLLQHDIGL
KD: 5.16e−08
Kass: 1.09e+05, kdiss: 5.63e−03
508_ID.1726_T4_SLLTHLIGL
KD:−
Kass:−, , kdiss: 1.91e−01
509_ID.1727_Y7_SLLQHLYGL
KD:−
Kass:−, , kdiss: 2.10e−02
510_ID.1730_D4_SLLDHLIGL
KD: 1.16e−08
Kass: 7.87e+04, kdiss: 9.10e−04
511_ID.1731_L8_SLLQHLILL
KD: 2.85e−08
Kass: 1.12e+05, kdiss: 3.18e−03
512_ID.1734_S4_SLLSHLIGL
KD: 7.77e−09
Kass: 1.60e+05, kdiss: 1.24e−03
513_ID.1739_H1_HLLQHLIGL
KD: 7.50e−09
Kass: 1.91e+05, kdiss: 1.43e−03
514_ID.1740_E4_SLLEHLIGL
KD: 1.39e−07
Kass: 3.03e+04, kdiss: 4.20e−03
515_ID.1741_D7_SLLQHLDGL
KD: 1.05e−07
Kass: 1.00e+04, kdiss: 1.05e−03
516_ID.1742_I5_SLLQILIGL
KD:−
Kass:−, , kdiss: 1.51e−01
517_ID.1743_V3_SLVQHLIGL
KD: 4.94e−09
Kass: 2.01e+05, kdiss: 9.92e−04
518_ID.1745_R8_SLLQHLIRL
KD: 6.54e−08
Kass: 7.13e+04, kdiss: 4.66e−03
519_ID.1746_L4_SLLLHLIGL
KD:−
Kass:−, , kdiss: 3.40e−01
520_ID.1747_I8_SLLQHLIIL
KD: 5.05e−08
Kass: 7.06e+04, kdiss: 3.56e−03
521_ID.1748_A4_SLLAHLIGL
KD: 5.26e−09
Kass: 1.90e+05, kdiss: 9.99e−04
522_ID.1749_W3_SLWQHLIGL
KD: 4.44e−08
Kass: 1.20e+05, kdiss: 5.32e−03
523_ID.1751_E7_SLLQHLEGL
KD: 2.08e−09
Kass: 2.73e+04, kdiss: 5.70e−05
524_ID.1753_L1_LLLQHLIGL
KD:−
Kass:−, , kdiss: 7.44e−01
525_ID.1755_Q7_SLLQHLQGL
KD:−
Kass:−, , kdiss: 3.55e−02
526_ID.1756_E4_SLLEHLIGL
KD: 2.44e−08
Kass: 7.58e+04, kdiss: 1.85e−03
527_ID.1757_W8_SLLQHLIWL
KD:−
Kass:−, , kdiss: 1.64e+00
528_ID.1758_L5_SLLQLLIGL
KD:−
Kass:−, , kdiss: 4.22e−02
529_ID.1759_R8_SLLQHLIRL
KD: 5.54e−09
Kass: 1.51e+05, kdiss: 8.35e−04
530_ID.176_H4_SLLHHLIGL
KD: 8.69e−09
Kass: 1.44e+05, kdiss: 1.25e−03
531_ID.1760_P5_SLLQPLIGL
KD:−
Kass:−, , kdiss: 1.97e−02
532_ID.1761_M8_SLLQHLIML
KD: 2.03e−07
Kass: 3.30e+04, kdiss: 6.68e−03
533_ID.1762_D5_SLLQDLIGL
KD: 1.20e−07
Kass: 9.01e+04, kdiss: 1.08e−02
534_ID.1763_F8_SLLQHLIFL
KD:−
Kass:−, , kdiss: 2.72e−01
535_ID.1764_W5_SLLQWLIGL
KD:−
Kass:−, , kdiss: 3.36e−02
536_ID.1765_A8_SLLQHLIAL
KD: 1.21e−08
Kass: 1.29e+05, kdiss: 1.56e−03
537_ID.1767_G1_GLLQHLIGL
KD: 8.33e−09
Kass: 1.89e+05, kdiss: 1.58e−03
538_ID.1769_G7_SLLQHLGGL
KD:−
Kass:−, , kdiss: 3.46e−02
539_ID.1770_F4_SLLFHLIGL
KD:−
Kass:−, , kdiss: 9.27e−01
540_ID.1771_A8_SLLQHLIAL
KD: 4.77e−09
Kass: 1.48e+05, kdiss: 7.04e−04
541_ID.1776_E4_SLLEHLIGL
KD: 9.54e−09
Kass: 9.35e+04, kdiss: 8.91e−04
542_ID.1777_V3_SLVQHLIGL
KD: 4.10e−09
Kass: 1.85e+05, kdiss: 7.59e−04
543_ID.1778_T5_SLLQTLIGL
KD:−
Kass:−, , kdiss: 3.84e−02
544_ID.1779_Y7_SLLQHLYGL
KD:−
Kass:−, , kdiss: 1.90e−02
545_ID.178_Q5_SLLQQLIGL
KD: 1.69e−06
Kass: 1.18e+04, kdiss: 2.00e−02
546_ID.1781_Q6_SLLQHQIGL
KD: 5.03e−09
Kass: 1.77e+05, kdiss: 8.93e−04
547_ID.1782_G4_SLLGHLIGL
KD: 7.97e−09
Kass: 1.69e+05, kdiss: 1.34e−03
548_ID.1784_I5_SLLQILIGL
KD:−
Kass:−, , kdiss: 2.06e−02
549_ID.1785_Y7_SLLQHLYGL
KD: 4.93e−07
Kass: 3.24e+04, kdiss: 1.60e−02
550_ID.1786_M4_SLLMHLIGL
KD: 4.90e−09
Kass: 1.78e+05, kdiss: 8.75e−04
551_ID.1787_S7_SLLQHLSGL
KD: 1.34e−07
Kass: 7.94e+04, kdiss: 1.07e−02
552_ID.1788_L5_SLLQLLIGL
KD:−
Kass:−, , kdiss: 2.57e−02
553_ID.179_V7_SLLQHLVGL
KD: 8.47e−09
Kass: 1.37e+05, kdiss: 1.16e−03
554_ID.1790_A4_SLLAHLIGL
KD: 4.23e−09
Kass: 1.47e+05, kdiss: 6.21e−04
555_ID.1791_Y3_SLYQHLIGL
KD: 2.79e−08
Kass: 1.43e+05, kdiss: 4.00e−03
556_ID.1793_H3_SLHQHLIGL
KD: 2.90e−08
Kass: 1.30e+05, kdiss: 3.76e−03
557_ID.1795_M7_SLLQHLMGL
KD: 2.39e−08
Kass: 1.43e+05, kdiss: 3.40e−03
558_ID.1797_H7_SLLQHLHGL
KD: 4.59e−08
Kass: 6.00e+04, kdiss: 2.75e−03
559_ID.1799_R7_SLLQHLRGL
KD:−
Kass:−, , kdiss: 5.62e−02
560_ID.180_L5_SLLQLLIGL
KD:−
Kass:−, , kdiss: 4.46e−02
561_ID.1802_V4_SLLVHLIGL
KD: 5.30e−09
Kass: 1.60e+05, kdiss: 8.47e−04
562_ID.1803_G1_GLLQHLIGL
KD:−
Kass:−, , kdiss: 1.40e−01
563_ID.1804_N4_SLLNHLIGL
KD: 5.01e−09
Kass: 1.72e+05, kdiss: 8.65e−04
564_ID.1805_S6_SLLQHSIGL
KD: 1.02e−08
Kass: 1.82e+05, kdiss: 1.85e−03
565_ID.1807_D7_SLLQHLDGL
KD: 1.08e−08
Kass: 1.18e+05, kdiss: 1.27e−03
566_ID.1808_Y4_SLLYHLIGL
KD: 9.60e−09
Kass: 1.78e+05, kdiss: 1.71e−03
567_ID.1809_A8_SLLQHLIAL
KD: 4.48e−09
Kass: 1.60e+05, kdiss: 7.18e−04
568_ID.181_N6_SLLQHNIGL
KD: 7.86e−09
Kass: 1.55e+05, kdiss: 1.22e−03
569_ID.1811_T1_TLLQHLIGL
KD: 2.12e−09
Kass: 1.83e+05, kdiss: 3.87e−04
570_ID.1812_W5_SLLQWLIGL
KD:−
Kass:−, , kdiss: 3.82e−02
571_ID.1814_K5_SLLQKLIGL
KD:−
Kass:−, , kdiss: 2.30e−02
572_ID.1815_R8_SLLQHLIRL
KD: 6.77e−07
Kass: 1.11e+04, kdiss: 7.53e−03
573_ID.1816_I4_SLLIHLIGL
KD: 2.10e−09
Kass: 1.80e+05, kdiss: 3.78e−04
574_ID.1817_H7_SLLQHLHGL
KD: 3.98e−08
Kass: 6.86e+04, kdiss: 2.73e−03
575_ID.1819_W6_SLLQHWIGL
KD: 5.28e−09
Kass: 1.89e+05, kdiss: 1.00e−03
576_ID.1821_R7_SLLQHLRGL
KD:−
Kass:−, , kdiss: 1.41e−01
577_ID.1822_T4_SLLTHLIGL
KD:−
Kass:−, , kdiss: 4.89e−02
578_ID.1825_T1_TLLQHLIGL
KD:−
Kass:−, , kdiss: 6.60e−02
579_ID.1826_E3_SLEQHLIGL
KD:−
Kass:−, , kdiss: 3.63e−01
580_ID.1827_M3_SLMQHLIGL
KD: 8.18e−09
Kass: 1.89e+05, kdiss: 1.55e−03
581_ID.1828_T5_SLLQTLIGL
KD:−
Kass:−, , kdiss: 1.32e−01
582_ID.1829_S7_SLLQHLSGL
KD: 3.31e−07
Kass: 4.87e+04, kdiss: 1.61e−02
583_ID.1830_N5_SLLQNLIGL
KD: 1.49e−06
Kass: 1.83e+04, kdiss: 2.74e−02
584_ID.1831_V7_SLLQHLVGL
KD: 4.39e−09
Kass: 1.72e+05, kdiss: 7.56e−04
585_ID.1832_A5_SLLQALIGL
KD: 9.27e−08
Kass: 1.30e+05, kdiss: 1.21e−02
586_ID.1835_Q1_QLLQHLIGL
KD: 6.51e−08
Kass: 1.03e+05, kdiss: 6.72e−03
587_ID.1836_T3_SLTQHLIGL
KD: 9.79e−09
Kass: 1.90e+05, kdiss: 1.86e−03
588_ID.1837_D8_SLLQHLIDL
KD: 6.70e−08
Kass: 8.72e+04, kdiss: 5.84e−03
589_ID.1839_T7_SLLQHLTGL
KD: 4.91e−08
Kass: 9.35e+04, kdiss: 4.59e−03
590_ID.184_F5_SLLQFLIGL
KD:−
Kass:−, , kdiss: 3.60e−02
591_ID.1841_D7_SLLQHLDGL
KD:−
Kass:−, , kdiss: 1.47e+00
592_ID.1842_M4_SLLMHLIGL
KD:−
Kass:−, , kdiss: 2.51e−01
593_ID.1845_L8_SLLQHLILL
KD: 4.13e−08
Kass: 8.69e+04, kdiss: 3.59e−03
594_ID.1847_F7_SLLQHLFGL
KD: 3.37e−08
Kass: 1.80e+05, kdiss: 6.07e−03
595_ID.1848_W5_SLLQWLIGL
KD:−
Kass:−, , kdiss: 2.84e−02
596_ID.1849_A1_ALLQHLIGL
KD: 1.74e−09
Kass: 2.00e+05, kdiss: 3.48e−04
597_ID.185_P8_SLLQHLIPL
KD: 1.45e−08
Kass: 1.33e+05, kdiss: 1.93e−03
598_ID.1850_V4_SLLVHLIGL
KD: 3.88e−08
Kass: 1.21e+05, kdiss: 4.70e−03
599_ID.1851_E7_SLLQHLEGL
KD: 1.86e−08
Kass: 4.71e+04, kdiss: 8.78e−04
600_ID.1854_P5_SLLQPLIGL
KD: 1.91e−06
Kass: 8.10e+03, kdiss: 1.54e−02
value
8000
4000
0
0 250 500 750 1000
Time

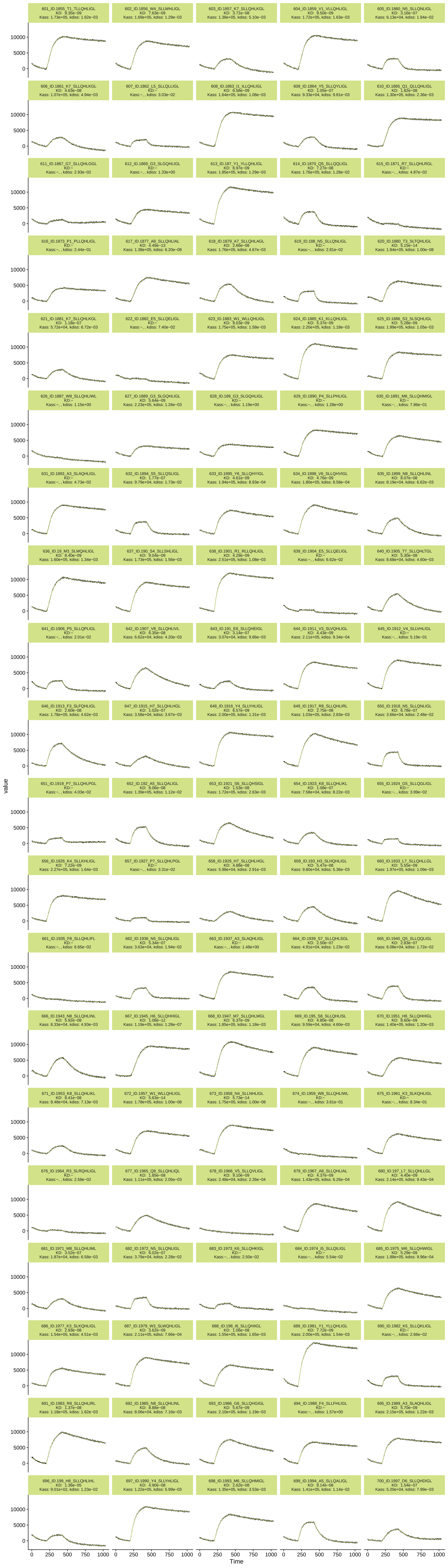
601_ID.1855_T1_TLLQHLIGL KD: 9.35e−09 Kass: 1.73e+05, kdiss: 1.62e−03
602_ID.1856_W4_SLLWHLIGL KD: 7.63e−09 Kass: 1.69e+05, kdiss: 1.29e−03
603_ID.1857_K7_SLLQHLKGL KD: 3.71e−08 Kass: 1.38e+05, kdiss: 5.10e−03
604_ID.1859_V1_VLLQHLIGL KD: 9.50e−09 Kass: 1.72e+05, kdiss: 1.63e−03
605_ID.1860_N5_SLLQNLIGL KD: 3.16e−07 Kass: 6.13e+04, kdiss: 1.94e−02
606_ID.1861_K7_SLLQHLKGL KD: 4.63e−08 Kass: 1.07e+05, kdiss: 4.94e−03
607_ID.1862_L5_SLLQLLIGL KD:− Kass:−, , kdiss: 3.03e−02
608_ID.1863_I1_ILLQHLIGL KD: 6.58e−09 Kass: 1.64e+05, kdiss: 1.08e−03
609_ID.1864_Y5_SLLQYLIGL KD: 1.05e−07 Kass: 9.33e+04, kdiss: 9.81e−03
610_ID.1865_Q1_QLLQHLIGL KD: 1.82e−08 Kass: 1.30e+05, kdiss: 2.36e−03
611_ID.1867_G7_SLLQHLGGL KD:− Kass:−, , kdiss: 2.93e−02
612_ID.1869_G3_SLGQHLIGL KD:− Kass:−, , kdiss: 1.33e+00
613_ID.187_Y1_YLLQHLIGL KD: 6.97e−09 Kass: 1.85e+05, kdiss: 1.29e−03
614_ID.1870_Q5_SLLQQLIGL KD: 7.27e−08 Kass: 1.76e+05, kdiss: 1.28e−02
615_ID.1871_R7_SLLQHLRGL KD:− Kass:−, , kdiss: 4.87e−02
616_ID.1873_P1_PLLQHLIGL KD:− Kass:−, , kdiss: 2.44e−01
617_ID.1877_A8_SLLQHLIAL KD: 4.49e−13 Kass: 1.38e+05, kdiss: 6.20e−08
618_ID.1879_A7_SLLQHLAGL KD: 2.66e−08 Kass: 1.76e+05, kdiss: 4.67e−03
619_ID.188_N5_SLLQNLIGL KD: 5.37e−07 Kass:−, , kdiss: 2.81e−02
620_ID.1880_T3_SLTQHLIGL KD: 5.15e−14 Kass: 1.94e+05, kdiss: 1.00e−08
621_ID.1881_K7_SLLQHLKGL KD: 1.18e−07 Kass: 5.72e+04, kdiss: 6.72e−03
622_ID.1882_E5_SLLQELIGL KD:− Kass:−, , kdiss: 7.40e−02
623_ID.1883_W1_WLLQHLIGL KD: 9.03e−09 Kass: 1.75e+05, kdiss: 1.58e−03
624_ID.1885_K1_KLLQHLIGL KD: 5.37e−09 Kass: 2.20e+05, kdiss: 1.18e−03
625_ID.1886_S3_SLSQHLIGL KD: 5.28e−09 Kass: 1.99e+05, kdiss: 1.05e−03
626_ID.1887_W8_SLLQHLIWL KD:− Kass:−, , kdiss: 1.15e+00
627_ID.1889_G3_SLGQHLIGL KD: 5.64e−09 Kass: 2.23e+05, kdiss: 1.26e−03
628_ID.189_G3_SLGQHLIGL KD:− Kass:−, , kdiss: 1.19e+00
629_ID.1890_P4_SLLPHLIGL KD:− Kass:−, , kdiss: 1.28e+00
630_ID.1891_M6_SLLQHMIGL KD:− Kass:−, , kdiss: 7.86e−01
631_ID.1893_A3_SLAQHLIGL KD:− Kass:−, , kdiss: 4.73e−02
632_ID.1894_S5_SLLQSLIGL KD: 1.77e−07 Kass: 9.79e+04, kdiss: 1.73e−02
633_ID.1895_Y6_SLLQHYIGL KD: 4.61e−09 Kass: 1.94e+05, kdiss: 8.93e−04
634_ID.1898_V6_SLLQHVIGL KD: 4.76e−09 Kass: 1.80e+05, kdiss: 8.58e−04
635_ID.1899_N8_SLLQHLINL KD: 8.07e−08 Kass: 8.19e+04, kdiss: 6.62e−03
636_ID.19_M3_SLMQHLIGL KD: 8.40e−09 Kass: 1.60e+05, kdiss: 1.34e−03
637_ID.190_S4_SLLSHLIGL KD: 9.04e−09 Kass: 1.73e+05, kdiss: 1.56e−03
638_ID.1901_R1_RLLQHLIGL KD: 4.29e−09 Kass: 2.51e+05, kdiss: 1.08e−03
639_ID.1904_E5_SLLQELIGL KD:− Kass:−, , kdiss: 6.62e−02
640_ID.1905_T7_SLLQHLTGL KD: 5.30e−08 Kass: 8.68e+04, kdiss: 4.60e−03
641_ID.1906_P5_SLLQPLIGL KD:− Kass:−, , kdiss: 2.01e−02
642_ID.1907_V8_SLLQHLIVL KD: 6.35e−08 Kass: 6.62e+04, kdiss: 4.20e−03
643_ID.191_E6_SLLQHEIGL KD: 3.14e−07 Kass: 3.07e+04, kdiss: 9.66e−03
644_ID.1911_V3_SLVQHLIGL KD: 4.43e−09 Kass: 2.11e+05, kdiss: 9.34e−04
645_ID.1912_V4_SLLVHLIGL KD:− Kass:−, , kdiss: 5.19e−01
646_ID.1913_F3_SLFQHLIGL KD: 2.60e−08 Kass: 1.78e+05, kdiss: 4.62e−03
647_ID.1915_H7_SLLQHLHGL KD: 1.02e−07 Kass: 3.58e+04, kdiss: 3.67e−03
648_ID.1916_Y4_SLLYHLIGL KD: 6.57e−09 Kass: 2.00e+05, kdiss: 1.31e−03
649_ID.1917_R8_SLLQHLIRL KD: 2.75e−08 Kass: 1.03e+05, kdiss: 2.83e−03
650_ID.1918_N5_SLLQNLIGL KD: 6.78e−07 Kass: 3.66e+04, kdiss: 2.48e−02
651_ID.1919_P7_SLLQHLPGL KD:− Kass:−, , kdiss: 4.03e−02
652_ID.192_A5_SLLQALIGL KD: 8.06e−08 Kass: 1.39e+05, kdiss: 1.12e−02
653_ID.1921_S6_SLLQHSIGL KD: 1.53e−08 Kass: 1.72e+05, kdiss: 2.63e−03
654_ID.1923_K8_SLLQHLIKL KD: 1.08e−07 Kass: 7.58e+04, kdiss: 8.22e−03
655_ID.1924_G5_SLLQGLIGL KD:− Kass:−, , kdiss: 3.99e−02
656_ID.1926_K4_SLLKHLIGL KD: 7.22e−09 Kass: 2.27e+05, kdiss: 1.64e−03
657_ID.1927_P7_SLLQHLPGL KD:− Kass:−, , kdiss: 3.31e−02
658_ID.1929_H7_SLLQHLHGL KD: 4.88e−08 Kass: 5.98e+04, kdiss: 2.91e−03
659_ID.193_H3_SLHQHLIGL KD: 5.47e−08 Kass: 9.80e+04, kdiss: 5.36e−03
660_ID.1933_L7_SLLQHLLGL KD: 5.55e−09 Kass: 1.97e+05, kdiss: 1.09e−03
661_ID.1935_F8_SLLQHLIFL KD:− Kass:−, , kdiss: 6.65e−02
662_ID.1936_N5_SLLQNLIGL KD: 5.34e−07 Kass: 3.63e+04, kdiss: 1.94e−02
663_ID.1937_A3_SLAQHLIGL KD:− Kass:−, , kdiss: 1.48e+00
664_ID.1939_S7_SLLQHLSGL KD: 2.50e−07 Kass: 4.91e+04, kdiss: 1.23e−02
665_ID.1940_Q5_SLLQQLIGL KD: 2.83e−07 Kass: 6.08e+04, kdiss: 1.72e−02
666_ID.1943_N8_SLLQHLINL KD: 5.92e−08 Kass: 8.33e+04, kdiss: 4.93e−03
667_ID.1945_H6_SLLQHHIGL KD: 1.08e−12 Kass: 1.19e+05, kdiss: 1.28e−07
668_ID.1947_M7_SLLQHLMGL KD: 6.37e−09 Kass: 1.85e+05, kdiss: 1.18e−03
669_ID.195_S8_SLLQHLISL KD: 4.80e−08 Kass: 9.59e+04, kdiss: 4.60e−03
670_ID.1951_H6_SLLQHHIGL KD: 8.60e−09 Kass: 1.40e+05, kdiss: 1.20e−03
671_ID.1953_K8_SLLQHLIKL KD: 8.41e−08 Kass: 8.48e+04, kdiss: 7.13e−03
672_ID.1957_W1_WLLQHLIGL KD: 5.63e−14 Kass: 1.78e+05, kdiss: 1.00e−08
673_ID.1958_N4_SLLNHLIGL KD: 5.73e−14 Kass: 1.75e+05, kdiss: 1.00e−08
674_ID.1959_W8_SLLQHLIWL KD:− Kass:−, , kdiss: 3.61e−01
675_ID.1961_K3_SLKQHLIGL KD:− Kass:−, , kdiss: 8.34e−01
676_ID.1964_R3_SLRQHLIGL KD:− Kass:−, , kdiss: 2.58e−02
677_ID.1965_Q8_SLLQHLIQL KD: 1.85e−08 Kass: 1.11e+05, kdiss: 2.05e−03
678_ID.1966_V5_SLLQVLIGL KD: 9.10e−09 Kass: 2.48e+04, kdiss: 2.26e−04
679_ID.1967_A8_SLLQHLIAL KD: 4.37e−09 Kass: 1.43e+05, kdiss: 6.26e−04
680_ID.197_L7_SLLQHLLGL KD: 4.40e−09 Kass: 2.14e+05, kdiss: 9.43e−04
681_ID.1971_M8_SLLQHLIML KD: 3.52e−07 Kass: 1.87e+04, kdiss: 6.58e−03
682_ID.1972_N5_SLLQNLIGL KD: 6.02e−07 Kass: 3.79e+04, kdiss: 2.28e−02
683_ID.1973_K6_SLLQHKIGL KD:− Kass:−, , kdiss: 2.50e−02
684_ID.1974_I5_SLLQILIGL KD:− Kass:−, , kdiss: 5.54e−02
685_ID.1975_W6_SLLQHWIGL KD: 5.29e−09 Kass: 1.88e+05, kdiss: 9.96e−04
686_ID.1977_K3_SLKQHLIGL KD: 2.93e−08 Kass: 1.54e+05, kdiss: 4.51e−03
687_ID.1979_W3_SLWQHLIGL KD: 3.62e−09 Kass: 2.11e+05, kdiss: 7.66e−04
688_ID.198_I6_SLLQHIIGL KD: 1.06e−08 Kass: 1.55e+05, kdiss: 1.65e−03
689_ID.1981_Y1_YLLQHLIGL KD: 7.72e−09 Kass: 2.00e+05, kdiss: 1.54e−03
690_ID.1982_K5_SLLQKLIGL KD:− Kass:−, , kdiss: 2.66e−02
691_ID.1983_R8_SLLQHLIRL KD: 1.37e−08 Kass: 1.18e+05, kdiss: 1.62e−03
692_ID.1985_N8_SLLQHLINL KD: 8.88e−08 Kass: 8.06e+04, kdiss: 7.16e−03
693_ID.1986_G6_SLLQHGIGL KD: 5.67e−09 Kass: 2.10e+05, kdiss: 1.19e−03
694_ID.1988_F4_SLLFHLIGL KD:− Kass:−, , kdiss: 1.57e+00
695_ID.1989_A3_SLAQHLIGL KD: 5.70e−09 Kass: 2.15e+05, kdiss: 1.22e−03
696_ID.199_H8_SLLQHLIHL KD: 1.36e−05 Kass: 9.01e+02, kdiss: 1.23e−02
697_ID.1990_Y4_SLLYHLIGL KD: 4.90e−08 Kass: 1.22e+05, kdiss: 5.99e−03
698_ID.1993_M6_SLLQHMIGL KD: 2.62e−08 Kass: 1.35e+05, kdiss: 3.53e−03
699_ID.1994_A5_SLLQALIGL KD: 8.14e−08 Kass: 1.41e+05, kdiss: 1.14e−02
700_ID.1997_D6_SLLQHDIGL KD: 1.54e−07 Kass: 5.20e+04, kdiss: 7.99e−03
value
0
5000
10000
Time
0
250
500
750
1000

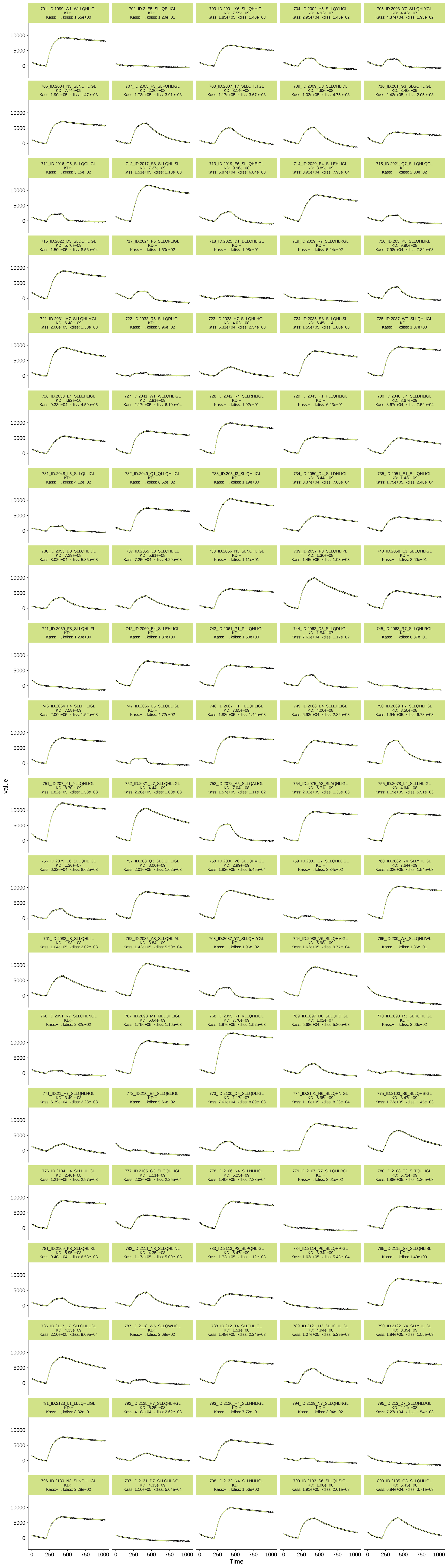
701_ID.1999_W1_WLLQHLIGL
KD:−
Kass:−, , kdiss: 1.55e+00
702_ID.2_E5_SLLQELIGL
KD:−
Kass:−, , kdiss: 1.20e−01
703_ID.2001_Y6_SLLQHYIGL
KD: 7.55e−09
Kass: 1.85e+05, kdiss: 1.40e−03
704_ID.2002_Y5_SLLQYLIGL
KD: 4.92e−07
Kass: 2.95e+04, kdiss: 1.45e−02
705_ID.2003_Y7_SLLQHLYGL
KD: 4.42e−07
Kass: 4.37e+04, kdiss: 1.93e−02
706_ID.2004_N3_SLNQHLIGL
KD: 7.74e−09
Kass: 1.90e+05, kdiss: 1.47e−03
707_ID.2005_F3_SLFQHLIGL
KD: 2.26e−08
Kass: 1.73e+05, kdiss: 3.91e−03
708_ID.2007_T7_SLLQHLTGL
KD: 3.14e−08
Kass: 1.17e+05, kdiss: 3.67e−03
709_ID.2009_D8_SLLQHLIDL
KD: 4.62e−08
Kass: 1.03e+05, kdiss: 4.75e−03
710_ID.201_G3_SLGQHLIGL
KD: 8.46e−09
Kass: 2.42e+05, kdiss: 2.05e−03
711_ID.2016_G5_SLLQGLIGL
KD:−
Kass:−, , kdiss: 3.15e−02
712_ID.2017_S8_SLLQHLISL
KD: 7.27e−09
Kass: 1.51e+05, kdiss: 1.10e−03
713_ID.2019_E6_SLLQHEIGL
KD: 9.96e−08
Kass: 6.87e+04, kdiss: 6.84e−03
714_ID.2020_E4_SLLEHLIGL
KD: 8.89e−09
Kass: 8.92e+04, kdiss: 7.93e−04
715_ID.2021_Q7_SLLQHLQGL
KD:−
Kass:−, , kdiss: 2.00e−02
716_ID.2022_D3_SLDQHLIGL
KD: 5.70e−09
Kass: 1.50e+05, kdiss: 8.56e−04
717_ID.2024_F5_SLLQFLIGL
KD:−
Kass:−, , kdiss: 1.63e−02
718_ID.2025_D1_DLLQHLIGL
KD:−
Kass:−, , kdiss: 1.98e−01
719_ID.2029_R7_SLLQHLRGL
KD:−
Kass:−, , kdiss: 5.24e−02
720_ID.203_K8_SLLQHLIKL
KD: 9.80e−08
Kass: 7.98e+04, kdiss: 7.82e−03
721_ID.2031_M7_SLLQHLMGL
KD: 6.48e−09
Kass: 2.00e+05, kdiss: 1.30e−03
722_ID.2032_R5_SLLQRLIGL
KD:−
Kass:−, , kdiss: 5.96e−02
723_ID.2033_H7_SLLQHLHGL
KD: 4.02e−08
Kass: 6.31e+04, kdiss: 2.54e−03
724_ID.2035_S8_SLLQHLISL
KD: 6.45e−14
Kass: 1.55e+05, kdiss: 1.00e−08
725_ID.2037_WT_SLLQHLIGL
KD:−
Kass:−, , kdiss: 1.07e+00
726_ID.2038_E4_SLLEHLIGL
KD: 4.92e−10
Kass: 9.33e+04, kdiss: 4.59e−05
727_ID.2041_W1_WLLQHLIGL
KD: 2.81e−09
Kass: 2.17e+05, kdiss: 6.10e−04
728_ID.2042_R4_SLLRHLIGL
KD:−
Kass:−, , kdiss: 1.92e−01
729_ID.2043_P1_PLLQHLIGL
KD:−
Kass:−, , kdiss: 6.23e−01
730_ID.2046_D4_SLLDHLIGL
KD: 8.67e−09
Kass: 8.67e+04, kdiss: 7.52e−04
731_ID.2048_L5_SLLQLLIGL
KD:−
Kass:−, , kdiss: 4.12e−02
732_ID.2049_Q1_QLLQHLIGL
KD:−
Kass:−, , kdiss: 6.52e−02
733_ID.205_I3_SLIQHLIGL
KD:−
Kass:−, , kdiss: 1.19e+00
734_ID.2050_D4_SLLDHLIGL
KD: 8.44e−09
Kass: 8.37e+04, kdiss: 7.06e−04
735_ID.2051_E1_ELLQHLIGL
KD: 1.42e−09
Kass: 1.75e+05, kdiss: 2.48e−04
736_ID.2053_D8_SLLQHLIDL
KD: 7.29e−08
Kass: 8.02e+04, kdiss: 5.85e−03
737_ID.2055_L8_SLLQHLILL
KD: 5.91e−08
Kass: 7.25e+04, kdiss: 4.29e−03
738_ID.2056_N3_SLNQHLIGL
KD:−
Kass:−, , kdiss: 1.11e−01
739_ID.2057_P8_SLLQHLIPL
KD: 1.36e−08
Kass: 1.45e+05, kdiss: 1.98e−03
740_ID.2058_E3_SLEQHLIGL
KD:−
Kass:−, , kdiss: 3.60e−01
741_ID.2059_F8_SLLQHLIFL
KD:−
Kass:−, , kdiss: 1.23e+00
742_ID.2060_E4_SLLEHLIGL
KD:−
Kass:−, , kdiss: 1.37e+00
743_ID.2061_P1_PLLQHLIGL
KD:−
Kass:−, , kdiss: 1.60e+00
744_ID.2062_D5_SLLQDLIGL
KD: 1.54e−07
Kass: 7.61e+04, kdiss: 1.17e−02
745_ID.2063_R7_SLLQHLRGL
KD:−
Kass:−, , kdiss: 6.87e−01
746_ID.2064_F4_SLLFHLIGL
KD: 7.58e−09
Kass: 2.00e+05, kdiss: 1.52e−03
747_ID.2066_L5_SLLQLLIGL
KD:−
Kass:−, , kdiss: 4.72e−02
748_ID.2067_T1_TLLQHLIGL
KD: 7.65e−09
Kass: 1.88e+05, kdiss: 1.44e−03
749_ID.2068_E4_SLLEHLIGL
KD: 4.06e−08
Kass: 6.93e+04, kdiss: 2.82e−03
750_ID.2069_F7_SLLQHLFGL
KD: 3.50e−08
Kass: 1.94e+05, kdiss: 6.78e−03
751_ID.207_Y1_YLLQHLIGL
KD: 8.70e−09
Kass: 1.82e+05, kdiss: 1.58e−03
752_ID.2071_L7_SLLQHLLGL
KD: 4.44e−09
Kass: 2.26e+05, kdiss: 1.00e−03
753_ID.2072_A5_SLLQALIGL
KD: 7.04e−08
Kass: 1.57e+05, kdiss: 1.11e−02
754_ID.2075_A3_SLAQHLIGL
KD: 6.71e−09
Kass: 2.02e+05, kdiss: 1.35e−03
755_ID.2078_L4_SLLLHLIGL
KD: 4.64e−08
Kass: 1.19e+05, kdiss: 5.51e−03
756_ID.2079_E6_SLLQHEIGL
KD: 1.36e−07
Kass: 6.32e+04, kdiss: 8.62e−03
757_ID.208_Q3_SLQQHLIGL
KD: 8.06e−09
Kass: 2.01e+05, kdiss: 1.62e−03
758_ID.2080_V6_SLLQHVIGL
KD: 2.99e−09
Kass: 1.82e+05, kdiss: 5.45e−04
759_ID.2081_G7_SLLQHLGGL
KD:−
Kass:−, , kdiss: 3.34e−02
760_ID.2082_Y4_SLLYHLIGL
KD: 7.64e−09
Kass: 2.02e+05, kdiss: 1.54e−03
761_ID.2083_I8_SLLQHLIIL
KD: 1.93e−08
Kass: 1.04e+05, kdiss: 2.02e−03
762_ID.2085_A8_SLLQHLIAL
KD: 3.84e−09
Kass: 1.43e+05, kdiss: 5.50e−04
763_ID.2087_Y7_SLLQHLYGL
KD:−
Kass:−, , kdiss: 1.96e−02
764_ID.2088_V6_SLLQHVIGL
KD: 5.98e−09
Kass: 1.63e+05, kdiss: 9.77e−04
765_ID.209_W8_SLLQHLIWL
KD:−
Kass:−, , kdiss: 1.86e−01
766_ID.2091_N7_SLLQHLNGL
KD:−
Kass:−, , kdiss: 2.82e−02
767_ID.2093_M1_MLLQHLIGL
KD: 6.64e−09
Kass: 1.75e+05, kdiss: 1.16e−03
768_ID.2095_K1_KLLQHLIGL
KD: 7.76e−09
Kass: 1.97e+05, kdiss: 1.52e−03
769_ID.2097_D6_SLLQHDIGL
KD: 1.02e−07
Kass: 5.68e+04, kdiss: 5.80e−03
770_ID.2098_R3_SLRQHLIGL
KD:−
Kass:−, , kdiss: 2.66e−02
771_ID.21_H7_SLLQHLHGL
KD: 3.49e−08
Kass: 6.39e+04, kdiss: 2.23e−03
772_ID.210_E5_SLLQELIGL
KD:−
Kass:−, , kdiss: 5.66e−02
773_ID.2100_D5_SLLQDLIGL
KD: 1.17e−07
Kass: 7.61e+04, kdiss: 8.89e−03
774_ID.2101_N6_SLLQHNIGL
KD: 6.95e−09
Kass: 1.18e+05, kdiss: 8.23e−04
775_ID.2103_S6_SLLQHSIGL
KD: 8.47e−09
Kass: 1.72e+05, kdiss: 1.45e−03
776_ID.2104_L4_SLLLHLIGL
KD: 2.46e−08
Kass: 1.21e+05, kdiss: 2.97e−03
777_ID.2105_G3_SLGQHLIGL
KD: 1.11e−09
Kass: 2.02e+05, kdiss: 2.25e−04
778_ID.2106_N4_SLLNHLIGL
KD: 5.25e−09
Kass: 1.40e+05, kdiss: 7.33e−04
779_ID.2107_R7_SLLQHLRGL
KD:−
Kass:−, , kdiss: 3.61e−02
780_ID.2108_T3_SLTQHLIGL
KD: 6.71e−09
Kass: 1.88e+05, kdiss: 1.26e−03
781_ID.2109_K8_SLLQHLIKL
KD: 6.95e−08
Kass: 9.40e+04, kdiss: 6.53e−03
782_ID.2111_N8_SLLQHLINL
KD: 4.35e−08
Kass: 1.17e+05, kdiss: 5.09e−03
783_ID.2113_P3_SLPQHLIGL
KD: 6.47e−09
Kass: 1.72e+05, kdiss: 1.12e−03
784_ID.2114_P6_SLLQHPIGL
KD: 3.34e−09
Kass: 1.63e+05, kdiss: 5.43e−04
785_ID.2115_S8_SLLQHLISL
KD:−
Kass:−, , kdiss: 1.49e+00
786_ID.2117_L7_SLLQHLLGL
KD: 4.33e−09
Kass: 2.10e+05, kdiss: 9.09e−04
787_ID.2118_W5_SLLQWLIGL
KD:−
Kass:−, , kdiss: 2.68e−02
788_ID.212_T4_SLLTHLIGL
KD: 1.51e−08
Kass: 1.48e+05, kdiss: 2.24e−03
789_ID.2121_H3_SLHQHLIGL
KD: 4.94e−08
Kass: 1.07e+05, kdiss: 5.29e−03
790_ID.2122_Y4_SLLYHLIGL
KD: 8.39e−09
Kass: 1.84e+05, kdiss: 1.55e−03
791_ID.2123_L1_LLLQHLIGL
KD:−
Kass:−, , kdiss: 8.32e−01
792_ID.2125_H7_SLLQHLHGL
KD: 6.25e−08
Kass: 4.18e+04, kdiss: 2.62e−03
793_ID.2126_H4_SLLHHLIGL
KD:−
Kass:−, , kdiss: 7.72e−01
794_ID.2129_N7_SLLQHLNGL
KD:−
Kass:−, , kdiss: 3.94e−02
795_ID.213_D7_SLLQHLDGL
KD: 2.11e−08
Kass: 7.27e+04, kdiss: 1.54e−03
796_ID.2130_N3_SLNQHLIGL
KD:−
Kass:−, , kdiss: 2.28e−02
797_ID.2131_D7_SLLQHLDGL
KD: 4.33e−09
Kass: 1.16e+05, kdiss: 5.04e−04
798_ID.2132_N4_SLLNHLIGL
KD:−
Kass:−, , kdiss: 1.56e+00
799_ID.2133_S6_SLLQHSIGL
KD: 1.06e−08
Kass: 1.91e+05, kdiss: 2.01e−03
800_ID.2135_Q8_SLLQHLIQL
KD: 5.43e−08
Kass: 6.84e+04, kdiss: 3.71e−03
value
Time
0
250
500
750
1000
10000
5000
0

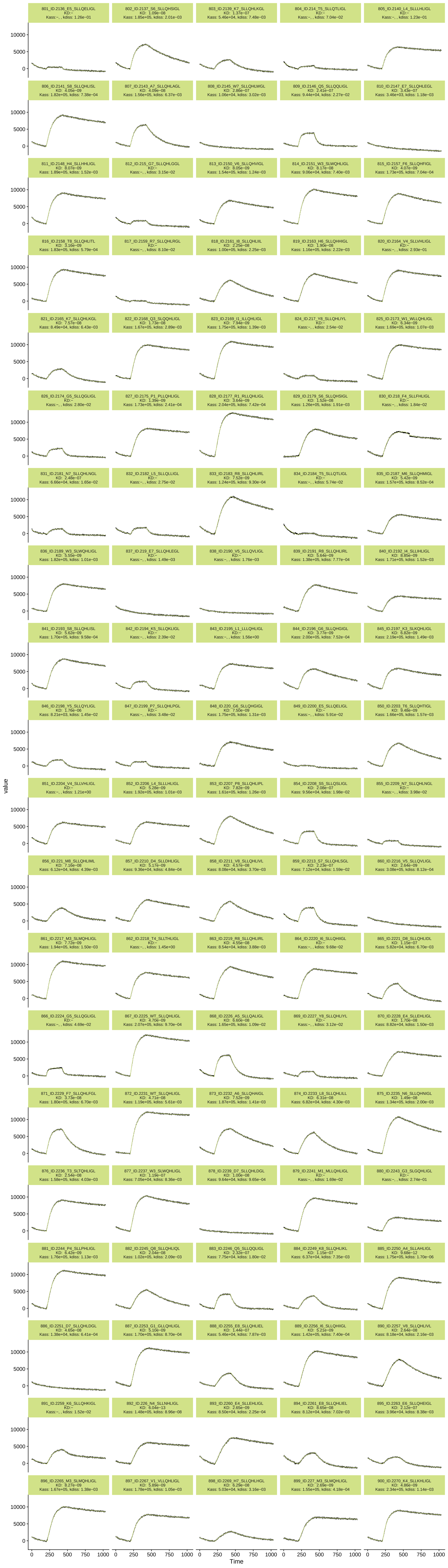

value
Time
0
250
500
750
1000
5000
10000

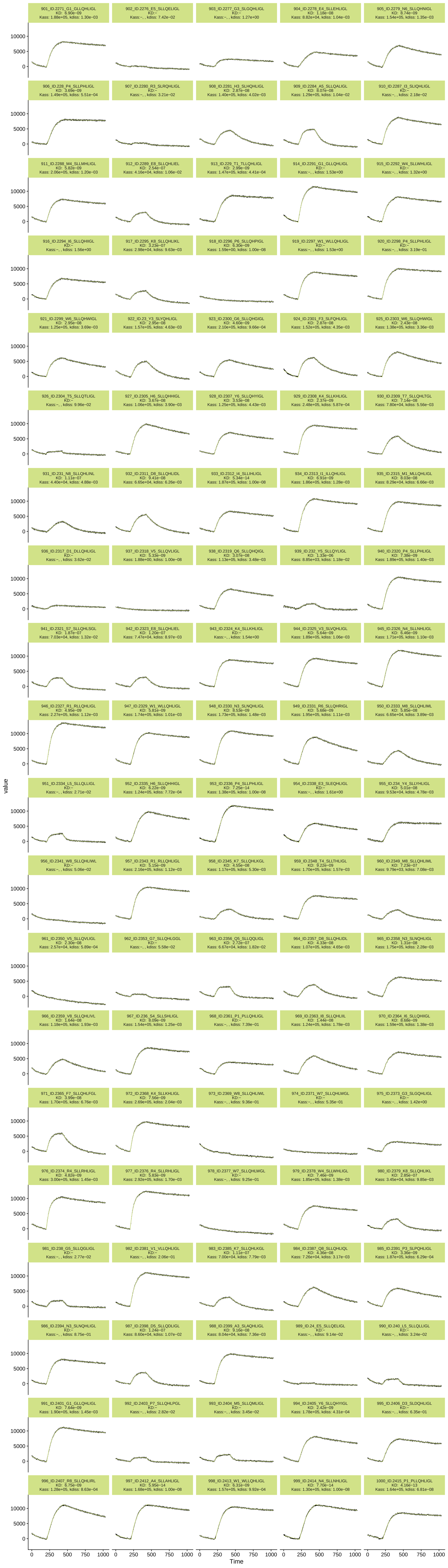

901_ID.2271_G1_GLLQHLIGL KD: 6.90e−09 Kass: 1.88e+05, kdiss: 1.30e−03
902_ID.2276_E5_SLLQELIGL KD:~ Kass:~ , kdiss: 7.42e−02
903_ID.2277_G3_SLGQHLIGL KD:~ Kass:~ , kdiss: 1.27e+00
904_ID.2278_E4_SLLEHLIGL KD: 1.18e−08 Kass: 8.82e+04, kdiss: 1.04e−03
905_ID.2279_N6_SLLQNIGL KD: 8.74e−09 Kass: 1.54e+05, kdiss: 1.35e−03
906_ID.228_P4_SLLPHLIGL KD: 3.69e−09 Kass: 1.49e+05, kdiss: 5.51e−04
907_ID.2280_R3_SLRQHLIGL KD: 3.23e−07 Kass:~ , kdiss: 3.21e−02
908_ID.2281_H3_SLHQHLIGL KD: 2.87e−08 Kass: 1.40e+05, kdiss: 4.02e−03
909_ID.2284_A5_SLLQALIGL KD: 8.07e−08 Kass: 1.29e+05, kdiss: 1.04e−02
910_ID.2287_I3_SLIQHLIGL KD:~ Kass:~ , kdiss: 2.18e−02
911_ID.2288_M4_SLLMHLIGL KD: 5.82e−09 Kass: 2.06e+05, kdiss: 1.20e−03
912_ID.2289_E8_SLLQHLIEL KD: 2.55e−07 Kass: 4.16e+04, kdiss: 1.06e−02
913_ID.229_T1_TLLQHLIGL KD: 2.99e−09 Kass: 1.47e+05, kdiss: 4.41e−04
914_ID.2291_G1_GLLQHLIGL KD:~ Kass:~ , kdiss: 1.53e+00
915_ID.2292_W4_SLLWHLIGL KD:~ Kass:~ , kdiss: 1.32e+00
916_ID.2294_I6_SLLQHIIGL KD:~ Kass:~ , kdiss: 1.56e+00
917_ID.2295_K8_SLLQHLIKL KD: 3.23e−08 Kass: 2.98e+04, kdiss: 9.63e−03
918_ID.2296_P6_SLLQHPIGL KD: 6.30e−09 Kass: 1.59e+00, kdiss: 1.00e−08
919_ID.2297_W1_WLLQHLIGL KD:~ Kass:~ , kdiss: 1.53e+00
920_ID.2298_P4_SLLPHLIGL KD:~ Kass:~ , kdiss: 3.19e−01
921_ID.2299_W6_SLLQHWIGL KD: 2.95e−08 Kass: 1.25e+05, kdiss: 3.69e−03
922_ID.23_Y3_SLYQHLIGL KD: 2.95e−08 Kass: 1.57e+05, kdiss: 4.63e−03
923_ID.2300_G6_SLLQHGIGL KD: 4.60e−09 Kass: 2.10e+05, kdiss: 9.66e−04
924_ID.2301_F3_SLFQHLIGL KD: 2.87e−08 Kass: 1.52e+05, kdiss: 4.35e−03
925_ID.2303_W6_SLLQHWIGL KD: 2.43e−08 Kass: 1.38e+05, kdiss: 3.36e−03
926_ID.2304_T5_SLLQTLIGL KD:~ Kass:~ , kdiss: 9.96e−02
927_ID.2305_H6_SLLQHHIGL KD: 3.67e−08 Kass: 1.06e+05, kdiss: 3.90e−03
928_ID.2307_Y6_SLLQHYIGL KD: 3.53e−08 Kass: 1.25e+05, kdiss: 4.43e−03
929_ID.2308_K4_SLLKHLIGL KD: 2.37e−09 Kass: 2.48e+05, kdiss: 5.87e−04
930_ID.2309_T7_SLLQHLTGL KD: 7.14e−08 Kass: 7.80e+04, kdiss: 5.56e−03
931_ID.231_N8_SLLQHLINL KD: 1.11e−07 Kass: 4.40e+04, kdiss: 4.88e−03
932_ID.2311_D8_SLLQHLIDL KD: 9.41e−08 Kass: 6.65e+04, kdiss: 6.26e−03
933_ID.2312_I4_SLLIHLIGL KD: 5.34e−14 Kass: 1.87e+05, kdiss: 1.00e−08
934_ID.2313_I1_ILLQHLIGL KD: 6.91e−09 Kass: 1.86e+05, kdiss: 1.28e−03
935_ID.2315_M1_MLLQHLIGL KD: 8.03e−08 Kass: 8.29e+04, kdiss: 6.66e−03
936_ID.2317_D1_DLLQHLIGL KD:~ Kass:~ , kdiss: 3.62e−02
937_ID.2318_V5_SLLQVLIGL KD: 5.33e−09 Kass: 1.88e+00, kdiss: 1.00e−08
938_ID.2319_Q6_SLLQHQIGL KD: 3.07e−08 Kass: 1.13e+05, kdiss: 3.48e−03
939_ID.232_Y5_SLLQYLIGL KD: 1.33e−06 Kass: 8.85e+03, kdiss: 1.18e−02
940_ID.2320_P4_SLLPHLIGL KD: 7.38e−09 Kass: 1.89e+05, kdiss: 1.40e−03
941_ID.2321_S7_SLLQHLSGL KD: 1.87e−07 Kass: 7.03e+04, kdiss: 1.32e−02
942_ID.2323_E8_SLLQHLIEL KD: 1.20e−07 Kass: 7.47e+04, kdiss: 8.97e−03
943_ID.2324_K4_SLLKHLIGL KD:~ Kass:~ , kdiss: 1.54e+00
944_ID.2325_V3_SLVQHLIGL KD: 5.64e−09 Kass: 1.89e+05, kdiss: 1.06e−03
945_ID.2326_N4_SLLNHLIGL KD: 6.46e−09 Kass: 1.71e+05, kdiss: 1.10e−03
946_ID.2327_R1_RLLQHLIGL KD: 4.95e−09 Kass: 2.27e+05, kdiss: 1.12e−03
947_ID.2329_W1_WLLQHLIGL KD: 5.81e−09 Kass: 1.74e+05, kdiss: 1.01e−03
948_ID.2330_N3_SLNQHLIGL KD: 8.53e−09 Kass: 1.73e+05, kdiss: 1.48e−03
949_ID.2331_R6_SLLQHRIGL KD: 5.68e−09 Kass: 1.95e+05, kdiss: 1.11e−03
950_ID.2333_M8_SLLQHLIML KD: 5.85e−08 Kass: 6.65e+04, kdiss: 3.89e−03
951_ID.2334_L5_SLLQLLIGL KD:~ Kass:~ , kdiss: 2.71e−02
952_ID.2335_H6_SLLQHHIGL KD: 6.22e−09 Kass: 1.24e+05, kdiss: 7.72e−04
953_ID.2336_P4_SLLPHLIGL KD: 7.25e−14 Kass: 1.38e+05, kdiss: 1.00e−08
954_ID.2338_E3_SLEQHLIGL KD:~ Kass:~ , kdiss: 1.61e+00
955_ID.234_Y4_SLLYHLIGL KD: 5.01e−08 Kass: 9.53e+04, kdiss: 4.78e−03
956_ID.2341_W8_SLLQHLIWL KD:~ Kass:~ , kdiss: 5.06e−02
957_ID.2343_R1_RLLQHLIGL KD: 5.15e−09 Kass: 2.16e+05, kdiss: 1.12e−03
958_ID.2345_K7_SLLQHLKGL KD: 4.55e−08 Kass: 1.17e+05, kdiss: 5.30e−03
959_ID.2348_T4_SLLTHLIGL KD: 9.22e−09 Kass: 1.70e+05, kdiss: 1.57e−03
960_ID.2349_M8_SLLQHLIML KD: 7.23e−07 Kass: 9.79e+03, kdiss: 7.08e−03
961_ID.2350_V5_SLLQVLIGL KD: 2.29e−09 Kass: 2.57e+04, kdiss: 5.89e−04
962_ID.2353_G7_SLLQHLGGL KD:~ Kass:~ , kdiss: 5.58e−02
963_ID.2356_Q5_SLLQQLIGL KD: 2.73e−07 Kass: 6.67e+04, kdiss: 1.82e−02
964_ID.2357_D8_SLLQHLIDL KD: 4.33e−08 Kass: 1.07e+05, kdiss: 4.65e−03
965_ID.2358_N3_SLNQHLIGL KD: 1.31e−08 Kass: 1.75e+05, kdiss: 2.28e−03
966_ID.2359_V8_SLLQHLIVL KD: 1.64e−08 Kass: 1.18e+05, kdiss: 1.93e−03
967_ID.236_S4_SLLSHLIGL KD: 8.09e−09 Kass: 1.54e+05, kdiss: 1.25e−03
968_ID.2361_P1_PLLQHLIGL KD:~ Kass:~ , kdiss: 7.39e−01
969_ID.2363_I8_SLLQHLIIL KD: 1.44e−08 Kass: 1.24e+05, kdiss: 1.78e−03
970_ID.2364_I6_SLLQHIIGL KD: 8.68e−09 Kass: 1.59e+05, kdiss: 1.38e−03
971_ID.2365_F7_SLLQHLFGL KD: 3.99e−08 Kass: 1.70e+05, kdiss: 6.76e−03
972_ID.2368_K4_SLLKHLIGL KD: 7.56e−09 Kass: 2.69e+05, kdiss: 2.04e−03
973_ID.2369_W8_SLLQHLIWL KD:~ Kass:~ , kdiss: 9.36e−01
974_ID.2371_W7_SLLQHLWGL KD:~ Kass:~ , kdiss: 5.35e−01
975_ID.2373_G3_SLGQHLIGL KD:~ Kass:~ , kdiss: 1.42e+00
976_ID.2374_R4_SLLRHLIGL KD: 4.82e−09 Kass: 3.00e+05, kdiss: 1.45e−03
977_ID.2376_R4_SLLRHLIGL KD: 5.83e−09 Kass: 2.92e+05, kdiss: 1.70e−03
978_ID.2377_W7_SLLQHLWGL KD:~ Kass:~ , kdiss: 9.25e−01
979_ID.2378_W4_SLLWHLIGL KD: 7.46e−09 Kass: 1.85e+05, kdiss: 1.38e−03
980_ID.2379_K8_SLLQHLIKL KD: 2.85e−07 Kass: 3.45e+04, kdiss: 9.85e−03
981_ID.238_G5_SLLQGLIGL KD:~ Kass:~ , kdiss: 2.77e−02
982_ID.2381_V1_VLLQHLIGL KD:~ Kass:~ , kdiss: 2.06e−01
983_ID.2385_K7_SLLQHLKGL KD: 1.11e−07 Kass: 7.00e+04, kdiss: 7.79e−03
984_ID.2387_Q8_SLLQHLIQL KD: 4.36e−08 Kass: 7.26e+04, kdiss: 3.17e−03
985_ID.2391_P3_SLPQHLIGL KD: 3.36e−09 Kass: 1.87e+05, kdiss: 6.29e−04
986_ID.2394_N3_SLNQHLIGL KD:~ Kass:~ , kdiss: 8.75e−01
987_ID.2398_D5_SLLQDLIGL KD: 1.24e−07 Kass: 8.60e+04, kdiss: 1.07e−02
988_ID.2399_A3_SLAQHLIGL KD: 9.16e−08 Kass: 8.04e+04, kdiss: 7.36e−03
989_ID.24_E5_SLLQELIGL KD:~ Kass:~ , kdiss: 9.14e−02
990_ID.240_L5_SLLQLLIGL KD:~ Kass:~ , kdiss: 3.24e−02
991_ID.2401_G1_GLLQHLIGL KD: 7.64e−09 Kass: 1.90e+05, kdiss: 1.45e−03
992_ID.2403_P7_SLLQHLPGL KD:~ Kass:~ , kdiss: 2.82e−02
993_ID.2404_M5_SLLQMLIGL KD:~ Kass:~ , kdiss: 3.45e−02
994_ID.2405_Y6_SLLQHYIGL KD: 2.42e−09 Kass: 1.78e+05, kdiss: 4.31e−04
995_ID.2406_D3_SLDQHLIGL KD:~ Kass:~ , kdiss: 6.35e−01
996_ID.2407_R8_SLLQHLIRL KD: 6.75e−09 Kass: 1.28e+05, kdiss: 8.63e−04
997_ID.2412_A4_SLLAHLIGL KD: 5.95e−14 Kass: 1.68e+05, kdiss: 1.00e−08
998_ID.2413_W1_WLLQHLIGL KD: 6.31e−09 Kass: 1.57e+05, kdiss: 9.92e−04
999_ID.2414_N4_SLLNHLIGL KD: 7.70e−14 Kass: 1.30e+05, kdiss: 1.00e−08
1000_ID.2415_P1_PLLQHLIGL KD: 4.16e−13 Kass: 1.64e+05, kdiss: 6.81e−08
value
0
5000
10000
0
250
500
750
1000
Time

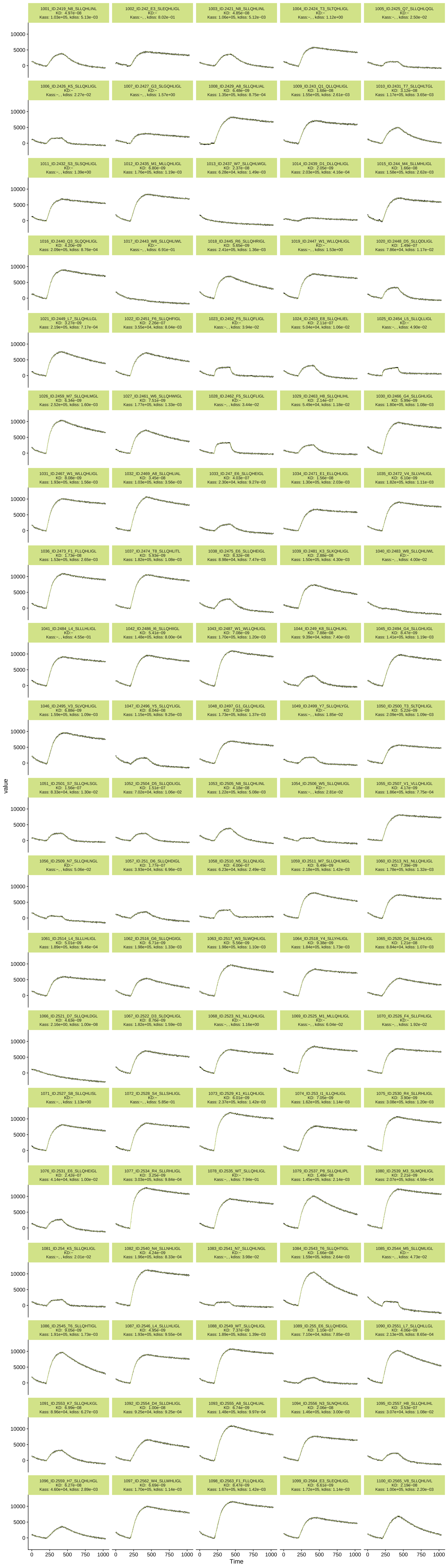
1001_ID.2419_N8_SLLQHLINL KD: 4.97e−08 Kass: 1.03e+05, kdiss: 5.13e−03
1002_ID.242_E3_SLEQHLIGL KD:− Kass:−, , kdiss: 8.02e−01
1003_ID.2421_N8_SLLQHLINL KD: 4.85e−08 Kass: 1.06e+05, kdiss: 5.12e−03
1004_ID.2424_T3_SLTQHLIGL KD:− Kass:−, , kdiss: 1.12e+00
1005_ID.2425_Q7_SLLQHLQGL KD:− Kass:−, , kdiss: 2.50e−02
1006_ID.2426_K5_SLLQKLIGL KD:− Kass:−, , kdiss: 2.27e−02
1007_ID.2427_G3_SLGQHLIGL KD:− Kass:−, , kdiss: 1.57e+00
1008_ID.2429_A8_SLLQHLIAL KD: 6.48e−09 Kass: 1.35e+05, kdiss: 8.75e−04
1009_ID.243_Q1_QLLQHLIGL KD: 1.68e−08 Kass: 1.55e+05, kdiss: 2.61e−03
1010_ID.2431_T7_SLLQHLTGL KD: 3.12e−08 Kass: 1.17e+05, kdiss: 3.65e−03
1011_ID.2432_S3_SLSQHLIGL KD:− Kass:−, , kdiss: 1.39e+00
1012_ID.2435_M1_MLLQHLIGL KD: 6.80e−09 Kass: 1.76e+05, kdiss: 1.19e−03
1013_ID.2437_W7_SLLQHLWGL KD: 2.37e−08 Kass: 6.28e+04, kdiss: 1.49e−03
1014_ID.2439_D1_DLLQHLIGL KD: 2.05e−09 Kass: 2.03e+05, kdiss: 4.16e−04
1015_ID.244_M4_SLLMHLIGL KD: 1.66e−08 Kass: 1.58e+05, kdiss: 2.62e−03
1016_ID.2440_Q3_SLQQHLIGL KD: 4.20e−09 Kass: 2.09e+05, kdiss: 8.76e−04
1017_ID.2443_W8_SLLQHLIWL KD:− Kass:−, , kdiss: 6.91e−01
1018_ID.2445_R6_SLLQHRIGL KD: 5.65e−09 Kass: 2.41e+05, kdiss: 1.36e−03
1019_ID.2447_W1_WLLQHLIGL KD:− Kass:−, , kdiss: 1.53e+00
1020_ID.2448_D5_SLLQDLIGL KD: 1.49e−07 Kass: 7.86e+04, kdiss: 1.17e−02
1021_ID.2449_L7_SLLQHLLGL KD: 3.27e−09 Kass: 2.19e+05, kdiss: 7.17e−04
1022_ID.2451_F6_SLLQHFIGL KD: 2.26e−07 Kass: 3.55e+04, kdiss: 8.04e−03
1023_ID.2452_F5_SLLQFLIGL KD:− Kass:−, , kdiss: 3.94e−02
1024_ID.2453_E8_SLLQHLIEL KD: 2.11e−07 Kass: 5.04e+04, kdiss: 1.06e−02
1025_ID.2454_L5_SLLQLLIGL KD:− Kass:−, , kdiss: 4.90e−02
1026_ID.2459_M7_SLLQHLMGL KD: 6.34e−09 Kass: 2.52e+05, kdiss: 1.60e−03
1027_ID.2461_W6_SLLQHWIGL KD: 7.51e−09 Kass: 1.77e+05, kdiss: 1.33e−03
1028_ID.2462_F5_SLLQFLIGL KD:− Kass:−, , kdiss: 3.44e−02
1029_ID.2463_H8_SLLQHLIHL KD: 2.14e−07 Kass: 5.49e+04, kdiss: 1.18e−02
1030_ID.2466_G4_SLLGHLIGL KD: 5.99e−09 Kass: 1.80e+05, kdiss: 1.08e−03
1031_ID.2467_W1_WLLQHLIGL KD: 8.08e−09 Kass: 1.93e+05, kdiss: 1.56e−03
1032_ID.2469_A8_SLLQHLIAL KD: 3.45e−08 Kass: 1.03e+05, kdiss: 3.56e−03
1033_ID.247_E6_SLLQHEIGL KD: 4.03e−07 Kass: 2.30e+04, kdiss: 9.27e−03
1034_ID.2471_E1_ELLQHLIGL KD: 1.56e−08 Kass: 1.30e+05, kdiss: 2.03e−03
1035_ID.2472_V4_SLLVHLIGL KD: 6.10e−09 Kass: 1.82e+05, kdiss: 1.11e−03
1036_ID.2473_F1_FLLQHLIGL KD: 1.73e−08 Kass: 1.53e+05, kdiss: 2.65e−03
1037_ID.2474_T8_SLLQHLITL KD: 5.93e−09 Kass: 1.82e+05, kdiss: 1.08e−03
1038_ID.2475_E6_SLLQHEIGL KD: 8.32e−08 Kass: 8.98e+04, kdiss: 7.47e−03
1039_ID.2481_K3_SLKQHLIGL KD: 2.88e−08 Kass: 1.50e+05, kdiss: 4.30e−03
1040_ID.2483_W8_SLLQHLIWL KD:− Kass:−, , kdiss: 4.00e−02
1041_ID.2484_L4_SLLLHLIGL KD:− Kass:−, , kdiss: 4.55e−01
1042_ID.2486_I6_SLLQHIIGL KD: 5.41e−09 Kass: 1.48e+05, kdiss: 8.00e−04
1043_ID.2487_W1_WLLQHLIGL KD: 7.08e−09 Kass: 1.70e+05, kdiss: 1.20e−03
1044_ID.249_K8_SLLQHLIKL KD: 7.88e−08 Kass: 9.39e+04, kdiss: 7.40e−03
1045_ID.2494_G4_SLLGHLIGL KD: 8.47e−09 Kass: 1.41e+05, kdiss: 1.19e−03
1046_ID.2495_V3_SLVQHLIGL KD: 6.88e−09 Kass: 1.59e+05, kdiss: 1.09e−03
1047_ID.2496_Y5_SLLQYLIGL KD: 8.04e−08 Kass: 1.15e+05, kdiss: 9.25e−03
1048_ID.2497_G1_GLLQHLIGL KD: 7.92e−09 Kass: 1.73e+05, kdiss: 1.37e−03
1049_ID.2499_Y7_SLLQHLYGL KD:− Kass:−, , kdiss: 1.85e−02
1050_ID.2500_T3_SLTQHLIGL KD: 5.22e−09 Kass: 2.09e+05, kdiss: 1.09e−03
1051_ID.2501_S7_SLLQHLSGL KD: 1.56e−07 Kass: 8.33e+04, kdiss: 1.30e−02
1052_ID.2504_D5_SLLQDLIGL KD: 1.51e−07 Kass: 7.02e+04, kdiss: 1.06e−02
1053_ID.2505_N8_SLLQHLINL KD: 4.18e−08 Kass: 1.22e+05, kdiss: 5.08e−03
1054_ID.2506_W5_SLLQWLIGL KD:− Kass:−, , kdiss: 2.81e−02
1055_ID.2507_V1_VLLQHLIGL KD: 4.17e−09 Kass: 1.86e+05, kdiss: 7.75e−04
1056_ID.2509_N7_SLLQHLNGL KD:− Kass:−, , kdiss: 5.06e−02
1057_ID.251_D6_SLLQHDIGL KD: 1.77e−07 Kass: 3.93e+04, kdiss: 6.96e−03
1058_ID.2510_N5_SLLQNLIGL KD: 4.00e−07 Kass: 6.23e+04, kdiss: 2.49e−02
1059_ID.2511_M7_SLLQHLMGL KD: 6.49e−09 Kass: 2.18e+05, kdiss: 1.42e−03
1060_ID.2513_N1_NLLQHLIGL KD: 7.39e−09 Kass: 1.78e+05, kdiss: 1.32e−03
1061_ID.2514_L4_SLLLHLIGL KD: 5.01e−09 Kass: 1.89e+05, kdiss: 9.46e−04
1062_ID.2516_G6_SLLQHGIGL KD: 6.71e−09 Kass: 1.98e+05, kdiss: 1.33e−03
1063_ID.2517_W3_SLWQHLIGL KD: 5.56e−09 Kass: 1.98e+05, kdiss: 1.10e−03
1064_ID.2518_Y4_SLLYHLIGL KD: 9.38e−09 Kass: 1.84e+05, kdiss: 1.73e−03
1065_ID.2520_D4_SLLDHLIGL KD: 1.21e−08 Kass: 8.84e+04, kdiss: 1.07e−03
1066_ID.2521_D7_SLLQHLDGL KD: 4.63e−09 Kass: 2.16e+00, kdiss: 1.00e−08
1067_ID.2522_D3_SLDQHLIGL KD: 8.76e−09 Kass: 1.82e+05, kdiss: 1.59e−03
1068_ID.2523_N1_NLLQHLIGL KD:− Kass:−, , kdiss: 1.16e+00
1069_ID.2525_M1_MLLQHLIGL KD:− Kass:−, , kdiss: 6.04e−02
1070_ID.2526_F4_SLLFHLIGL KD:− Kass:−, , kdiss: 1.92e−02
1071_ID.2527_S8_SLLQHLISL KD:− Kass:−, , kdiss: 1.13e+00
1072_ID.2528_S4_SLLSHLIGL KD:− Kass:−, , kdiss: 5.85e−01
1073_ID.2529_K1_KLLQHLIGL KD: 6.01e−09 Kass: 2.37e+05, kdiss: 1.42e−03
1074_ID.253_I1_ILLQHLIGL KD: 7.05e−09 Kass: 1.62e+05, kdiss: 1.14e−03
1075_ID.2530_R4_SLLRHLIGL KD: 3.90e−09 Kass: 3.08e+05, kdiss: 1.20e−03
1076_ID.2531_E6_SLLQHEIGL KD: 2.42e−07 Kass: 4.14e+04, kdiss: 1.00e−02
1077_ID.2534_R4_SLLRHLIGL KD: 3.25e−09 Kass: 3.03e+05, kdiss: 9.84e−04
1078_ID.2535_WT_SLLQHLIGL KD:− Kass:−, , kdiss: 7.94e−01
1079_ID.2537_P8_SLLQHLIPL KD: 1.48e−08 Kass: 1.45e+05, kdiss: 2.14e−03
1080_ID.2539_M3_SLMQHLIGL KD: 2.21e−09 Kass: 2.07e+05, kdiss: 4.56e−04
1081_ID.254_K5_SLLQKLIGL KD:− Kass:−, , kdiss: 2.01e−02
1082_ID.2540_N4_SLLNHLIGL KD: 4.24e−09 Kass: 1.96e+05, kdiss: 8.33e−04
1083_ID.2541_N7_SLLQHLNGL KD:− Kass:−, , kdiss: 3.98e−02
1084_ID.2543_T6_SLLQHTIGL KD: 1.66e−08 Kass: 1.59e+05, kdiss: 2.64e−03
1085_ID.2544_M5_SLLQMLIGL KD:− Kass:−, , kdiss: 4.73e−02
1086_ID.2545_T6_SLLQHTIGL KD: 9.05e−09 Kass: 1.91e+05, kdiss: 1.73e−03
1087_ID.2546_L4_SLLLHLIGL KD: 4.95e−09 Kass: 1.93e+05, kdiss: 9.55e−04
1088_ID.2549_WT_SLLQHLIGL KD: 7.37e−09 Kass: 1.89e+05, kdiss: 1.39e−03
1089_ID.255_E6_SLLQHEIGL KD: 1.10e−07 Kass: 7.10e+04, kdiss: 7.85e−03
1090_ID.2551_L7_SLLQHLLGL KD: 4.06e−09 Kass: 2.13e+05, kdiss: 8.65e−04
1091_ID.2553_K7_SLLQHLKGL KD: 6.99e−08 Kass: 8.96e+04, kdiss: 6.27e−03
1092_ID.2554_D4_SLLDHLIGL KD: 1.00e−08 Kass: 9.25e+04, kdiss: 9.25e−04
1093_ID.2555_A8_SLLQHLIAL KD: 6.74e−09 Kass: 1.48e+05, kdiss: 9.97e−04
1094_ID.2556_N3_SLNQHLIGL KD: 2.06e−08 Kass: 1.46e+05, kdiss: 3.00e−03
1095_ID.2557_H8_SLLQHLIHL KD: 3.53e−07 Kass: 3.07e+04, kdiss: 1.08e−02
1096_ID.2559_H7_SLLQHLHGL KD: 6.27e−08 Kass: 4.60e+04, kdiss: 2.89e−03
1097_ID.2562_W4_SLLWHLIGL KD: 6.69e−09 Kass: 1.70e+05, kdiss: 1.14e−03
1098_ID.2563_F1_FLLQHLIGL KD: 8.47e−09 Kass: 1.67e+05, kdiss: 1.42e−03
1099_ID.2564_E3_SLEQHLIGL KD: 6.61e−09 Kass: 1.72e+05, kdiss: 1.14e−03
1100_ID.2565_V8_SLLQHLIVL KD: 2.19e−08 Kass: 1.00e+05, kdiss: 2.20e−03
value
10000
5000
0
0 250 500 750 1000
Time

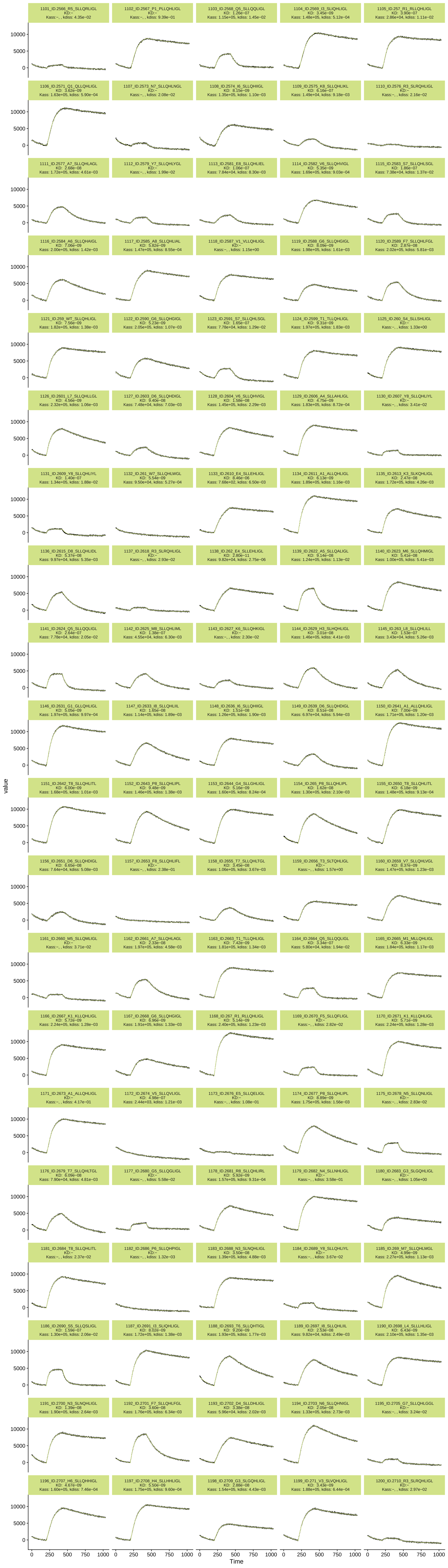

value
Time
0
250
500
750
1000
5000
10000

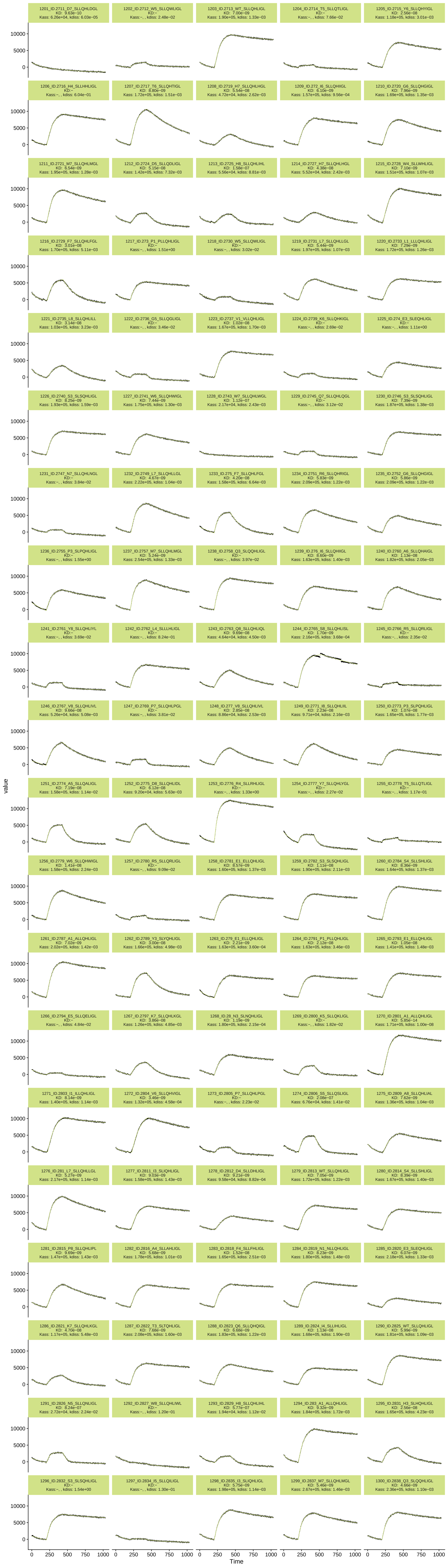
1201_ID.2711_D7_SLLQHLDGL KD: 9.63e−10 Kass: 6.26e+04, kdiss: 6.03e−05
1202_ID.2712_W5_SLLQWLIGL KD:~ Kass:~, , kdiss: 2.48e−02
1203_ID.2713_WT_SLLQHLIGL KD: 7.00e−09 Kass: 1.90e+05, kdiss: 1.33e−03
1204_ID.2714_T5_SLLQTLIGL KD:~ Kass:~, , kdiss: 7.66e−02
1205_ID.2715_Y6_SLLQHYIGL KD: 2.56e−08 Kass: 1.18e+05, kdiss: 3.01e−03
1206_ID.2716_H4_SLLHHLIGL KD:~ Kass:~, , kdiss: 6.04e−01
1207_ID.2717_T6_SLLQHTIGL KD: 8.80e−09 Kass: 1.72e+05, kdiss: 1.51e−03
1208_ID.2719_H7_SLLQHLHGL KD: 5.54e−08 Kass: 4.72e+04, kdiss: 2.62e−03
1209_ID.272_I6_SLLQHIIGL KD: 6.10e−09 Kass: 1.57e+05, kdiss: 9.56e−04
1210_ID.2720_G6_SLLQHGIGL KD: 7.96e−09 Kass: 1.69e+05, kdiss: 1.35e−03
1211_ID.2721_M7_SLLQHLMGL KD: 6.14e−09 Kass: 1.95e+05, kdiss: 1.28e−03
1212_ID.2724_D5_SLLQDLIGL KD: 5.15e−08 Kass: 1.42e+05, kdiss: 7.32e−03
1213_ID.2725_H8_SLLQHLIHL KD: 1.58e−07 Kass: 5.56e+04, kdiss: 8.81e−03
1214_ID.2727_H7_SLLQHLHGL KD: 4.38e−08 Kass: 5.52e+04, kdiss: 2.42e−03
1215_ID.2728_W4_SLLWHLIGL KD: 7.10e−09 Kass: 1.51e+05, kdiss: 1.07e−03
1216_ID.2729_F7_SLLQHLFGL KD: 3.01e−08 Kass: 1.70e+05, kdiss: 5.11e−03
1217_ID.273_P1_PLLQHLIGL KD:~ Kass:~, , kdiss: 1.51e+00
1218_ID.2730_W5_SLLQWLIGL KD:~ Kass:~, , kdiss: 3.02e−02
1219_ID.2731_L7_SLLQHLLGL KD: 5.44e−09 Kass: 1.97e+05, kdiss: 1.07e−03
1220_ID.2733_L1_LLLQHLIGL KD: 7.29e−09 Kass: 1.72e+05, kdiss: 1.26e−03
1221_ID.2735_L8_SLLQHLILL KD: 3.14e−08 Kass: 1.03e+05, kdiss: 3.23e−03
1222_ID.2736_G5_SLLQGLIGL KD:~ Kass:~, , kdiss: 3.46e−02
1223_ID.2737_V1_VLLQHLIGL KD: 1.02e−08 Kass: 1.67e+05, kdiss: 1.70e−03
1224_ID.2739_K6_SLLQHKIGL KD:~ Kass:~, , kdiss: 2.69e−02
1225_ID.274_E3_SLEQHLIGL KD:~ Kass:~, , kdiss: 1.11e+00
1226_ID.2740_S3_SLSQHLIGL KD: 8.25e−09 Kass: 1.93e+05, kdiss: 1.59e−03
1227_ID.2741_W6_SLLQHWIGL KD: 7.44e−09 Kass: 1.75e+05, kdiss: 1.30e−03
1228_ID.2743_W7_SLLQHLWGL KD: 1.12e−07 Kass: 2.17e+04, kdiss: 2.43e−03
1229_ID.2745_Q7_SLLQHLQGL KD:~ Kass:~, , kdiss: 3.12e−02
1230_ID.2746_S3_SLSQHLIGL KD: 7.40e−09 Kass: 1.87e+05, kdiss: 1.38e−03
1231_ID.2747_N7_SLLQHLNGL KD:~ Kass:~, , kdiss: 3.84e−02
1232_ID.2749_L7_SLLQHLLGL KD: 4.67e−09 Kass: 2.22e+05, kdiss: 1.04e−03
1233_ID.275_F7_SLLQHLFGL KD: 4.20e−08 Kass: 1.58e+05, kdiss: 6.64e−03
1234_ID.2751_R6_SLLQHRIGL KD: 5.83e−09 Kass: 2.09e+05, kdiss: 1.22e−03
1235_ID.2752_G6_SLLQHGIGL KD: 5.86e−09 Kass: 2.09e+05, kdiss: 1.22e−03
1236_ID.2755_P3_SLPQHLIGL KD:~ Kass:~, , kdiss: 1.55e+00
1237_ID.2757_M7_SLLQHLMGL KD: 5.24e−09 Kass: 2.54e+05, kdiss: 1.33e−03
1238_ID.2758_Q3_SLQQHLIGL KD:~ Kass:~, , kdiss: 3.97e−02
1239_ID.276_I6_SLLQHIIGL KD: 8.60e−09 Kass: 1.63e+05, kdiss: 1.40e−03
1240_ID.2760_A6_SLLQHAIGL KD: 1.13e−08 Kass: 1.82e+05, kdiss: 2.05e−03
1241_ID.2761_Y8_SLLQHLIYL KD:~ Kass:~, , kdiss: 3.69e−02
1242_ID.2762_L4_SLLLHLIGL KD:~ Kass:~, , kdiss: 8.24e−01
1243_ID.2763_Q8_SLLQHLIQL KD: 9.69e−08 Kass: 4.64e+04, kdiss: 4.50e−03
1244_ID.2765_S8_SLLQHLISL KD: 1.70e−09 Kass: 2.16e+05, kdiss: 3.68e−04
1245_ID.2766_R5_SLLQRLIGL KD:~ Kass:~, , kdiss: 2.35e−02
1246_ID.2767_V8_SLLQHLIVL KD: 9.66e−08 Kass: 5.26e+04, kdiss: 5.08e−03
1247_ID.2769_P7_SLLQHLPGL KD:~ Kass:~, , kdiss: 3.81e−02
1248_ID.277_V8_SLLQHLIVL KD: 2.85e−08 Kass: 8.86e+04, kdiss: 2.53e−03
1249_ID.2771_I8_SLLQHLIIL KD: 2.23e−08 Kass: 9.71e+04, kdiss: 2.16e−03
1250_ID.2773_P3_SLPQHLIGL KD: 1.07e−08 Kass: 1.65e+05, kdiss: 1.77e−03
1251_ID.2774_A5_SLLQALIGL KD: 7.19e−08 Kass: 1.58e+05, kdiss: 1.14e−02
1252_ID.2775_D8_SLLQHLIDL KD: 6.12e−08 Kass: 9.20e+04, kdiss: 5.63e−03
1253_ID.2776_R4_SLLRHLIGL KD:~ Kass:~, , kdiss: 1.33e+00
1254_ID.2777_Y7_SLLQHLYGL KD:~ Kass:~, , kdiss: 2.27e−02
1255_ID.2778_T5_SLLQTLIGL KD:~ Kass:~, , kdiss: 1.17e−01
1256_ID.2779_W6_SLLQHWIGL KD: 1.41e−08 Kass: 1.58e+05, kdiss: 2.24e−03
1257_ID.2780_R5_SLLQRLIGL KD:~ Kass:~, , kdiss: 9.09e−02
1258_ID.2781_E1_ELLQHLIGL KD: 8.57e−09 Kass: 1.60e+05, kdiss: 1.37e−03
1259_ID.2782_S3_SLSQHLIGL KD: 1.11e−08 Kass: 1.90e+05, kdiss: 2.11e−03
1260_ID.2784_S4_SLLSHLIGL KD: 8.36e−09 Kass: 1.64e+05, kdiss: 1.37e−03
1261_ID.2787_A1_ALLQHLIGL KD: 7.02e−09 Kass: 2.02e+05, kdiss: 1.42e−03
1262_ID.2789_Y3_SLYQHLIGL KD: 3.00e−08 Kass: 1.66e+05, kdiss: 4.98e−03
1263_ID.279_E1_ELLQHLIGL KD: 2.21e−09 Kass: 1.63e+05, kdiss: 3.60e−04
1264_ID.2791_P1_PLLQHLIGL KD: 2.12e−08 Kass: 1.63e+05, kdiss: 3.46e−03
1265_ID.2793_E1_ELLQHLIGL KD: 1.05e−08 Kass: 1.41e+05, kdiss: 1.48e−03
1266_ID.2794_E5_SLLQELIGL KD:~ Kass:~, , kdiss: 4.84e−02
1267_ID.2797_K7_SLLQHLKGL KD: 3.86e−08 Kass: 1.26e+05, kdiss: 4.85e−03
1268_ID.28_N3_SLNQHLIGL KD: 1.19e−09 Kass: 1.80e+05, kdiss: 2.15e−04
1269_ID.2800_K5_SLLQKLIGL KD:~ Kass:~, , kdiss: 1.82e−02
1270_ID.2801_A1_ALLQHLIGL KD: 5.85e−14 Kass: 1.71e+05, kdiss: 1.00e−08
1271_ID.2803_I1_ILLQHLIGL KD: 8.14e−09 Kass: 1.40e+05, kdiss: 1.14e−03
1272_ID.2804_V6_SLLQHVIGL KD: 3.46e−09 Kass: 1.32e+05, kdiss: 4.58e−04
1273_ID.2805_P7_SLLQHLPGL KD:~ Kass:~, , kdiss: 2.23e−02
1274_ID.2806_S5_SLLQSLIGL KD: 2.08e−07 Kass: 6.76e+04, kdiss: 1.41e−02
1275_ID.2809_A8_SLLQHLIAL KD: 7.62e−09 Kass: 1.36e+05, kdiss: 1.04e−03
1276_ID.281_L7_SLLQHLLGL KD: 5.27e−09 Kass: 2.17e+05, kdiss: 1.14e−03
1277_ID.2811_I3_SLIQHLIGL KD: 9.03e−09 Kass: 1.58e+05, kdiss: 1.43e−03
1278_ID.2812_D4_SLLDHLIGL KD: 9.21e−09 Kass: 9.58e+04, kdiss: 8.82e−04
1279_ID.2813_WT_SLLQHLIGL KD: 7.05e−09 Kass: 1.72e+05, kdiss: 1.22e−03
1280_ID.2814_S4_SLLSHLIGL KD: 8.39e−09 Kass: 1.67e+05, kdiss: 1.40e−03
1281_ID.2815_P8_SLLQHLIPL KD: 9.69e−09 Kass: 1.47e+05, kdiss: 1.43e−03
1282_ID.2816_A4_SLLAHLIGL KD: 5.68e−09 Kass: 1.78e+05, kdiss: 1.01e−03
1283_ID.2818_F4_SLLFHLIGL KD: 1.52e−08 Kass: 1.65e+05, kdiss: 2.51e−03
1284_ID.2819_N1_NLLQHLIGL KD: 8.23e−09 Kass: 1.80e+05, kdiss: 1.48e−03
1285_ID.2820_E3_SLEQHLIGL KD: 6.07e−09 Kass: 2.18e+05, kdiss: 1.33e−03
1286_ID.2821_K7_SLLQHLKGL KD: 4.70e−08 Kass: 1.17e+05, kdiss: 5.48e−03
1287_ID.2822_T3_SLTQHLIGL KD: 7.68e−09 Kass: 2.08e+05, kdiss: 1.60e−03
1288_ID.2823_Q6_SLLQHQIGL KD: 6.68e−09 Kass: 1.83e+05, kdiss: 1.22e−03
1289_ID.2824_I4_SLLIHLIGL KD: 1.13e−08 Kass: 1.68e+05, kdiss: 1.90e−03
1290_ID.2825_WT_SLLQHLIGL KD: 5.99e−09 Kass: 1.81e+05, kdiss: 1.09e−03
1291_ID.2826_N5_SLLQNLIGL KD: 8.24e−07 Kass: 2.72e+04, kdiss: 2.24e−02
1292_ID.2827_W8_SLLQHLIWL KD:~ Kass:~, , kdiss: 1.20e−01
1293_ID.2829_H8_SLLQHLIHL KD: 5.77e−07 Kass: 1.94e+04, kdiss: 1.12e−02
1294_ID.283_A1_ALLQHLIGL KD: 9.32e−09 Kass: 1.84e+05, kdiss: 1.72e−03
1295_ID.2831_H3_SLHQHLIGL KD: 2.56e−08 Kass: 1.65e+05, kdiss: 4.23e−03
1296_ID.2832_S3_SLSQHLIGL KD:~ Kass:~, , kdiss: 1.54e+00
1297_ID.2834_I5_SLLQILIGL KD:~ Kass:~, , kdiss: 1.30e−01
1298_ID.2835_I3_SLIQHLIGL KD: 5.75e−09 Kass: 1.98e+05, kdiss: 1.14e−03
1299_ID.2837_M7_SLLQHLMGL KD: 5.46e−09 Kass: 2.67e+05, kdiss: 1.46e−03
1300_ID.2838_Q3_SLQQHLIGL KD: 4.66e−09 Kass: 2.36e+05, kdiss: 1.10e−03
value
Time
0
5000
10000
0 250 500 750 1000

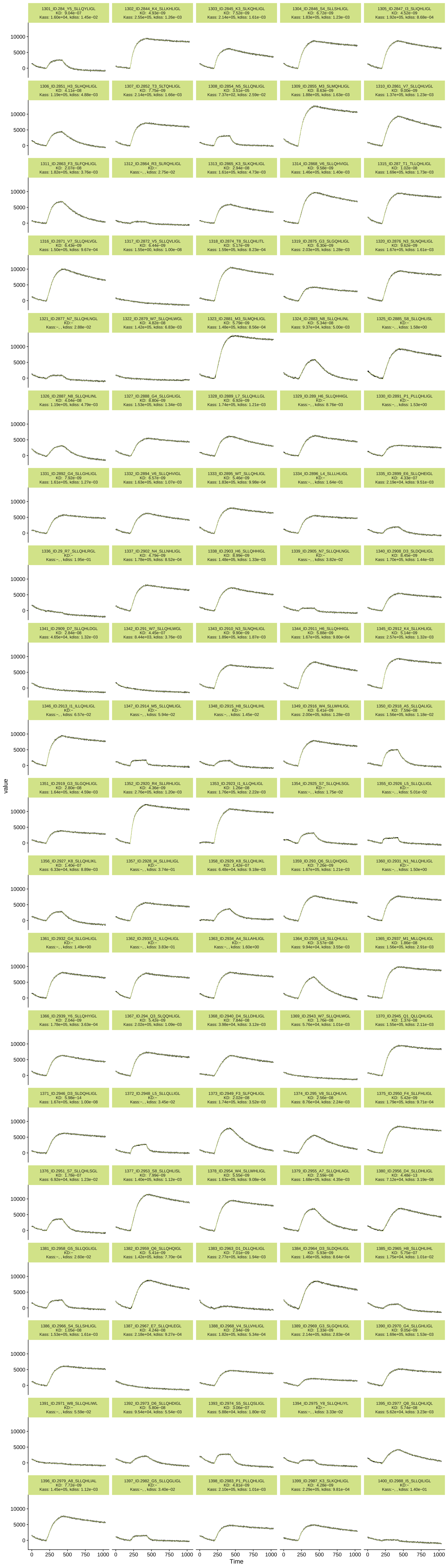
1301_ID.284_Y5_SLLQYLIGL
KD: 9.04e−07
Kass: 1.60e+04, kdiss: 1.45e−02
1302_ID.2844_K4_SLLKHLIGL
KD: 4.93e−09
Kass: 2.55e+05, kdiss: 1.26e−03
1303_ID.2845_K3_SLKQHLIGL
KD: 7.52e−09
Kass: 2.14e+05, kdiss: 1.61e−03
1304_ID.2846_S4_SLLSHLIGL
KD: 6.72e−09
Kass: 1.83e+05, kdiss: 1.23e−03
1305_ID.2847_I3_SLIQHLIGL
KD: 4.52e−09
Kass: 1.92e+05, kdiss: 8.68e−04
1306_ID.2851_H3_SLHQHLIGL
KD: 4.11e−08
Kass: 1.19e+05, kdiss: 4.88e−03
1307_ID.2852_T3_SLTQHLIGL
KD: 7.75e−09
Kass: 2.14e+05, kdiss: 1.66e−03
1308_ID.2854_N5_SLLQNLIGL
KD: 3.51e−05
Kass: 7.37e+02, kdiss: 2.59e−02
1309_ID.2855_M3_SLMQHLIGL
KD: 8.63e−09
Kass: 1.88e+05, kdiss: 1.63e−03
1310_ID.2861_V7_SLLQHLVGL
KD: 9.00e−09
Kass: 1.37e+05, kdiss: 1.23e−03
1311_ID.2863_F3_SLFQHLIGL
KD: 2.07e−08
Kass: 1.82e+05, kdiss: 3.76e−03
1312_ID.2864_R3_SLRQHLIGL
KD:−
Kass:−, , kdiss: 2.75e−02
1313_ID.2865_K3_SLKQHLIGL
KD: 2.94e−08
Kass: 1.61e+05, kdiss: 4.73e−03
1314_ID.2868_V6_SLLQHVIGL
KD: 9.58e−09
Kass: 1.46e+05, kdiss: 1.40e−03
1315_ID.287_T1_TLLQHLIGL
KD: 1.02e−08
Kass: 1.69e+05, kdiss: 1.73e−03
1316_ID.2871_V7_SLLQHLVGL
KD: 6.43e−09
Kass: 1.50e+05, kdiss: 9.67e−04
1317_ID.2872_V5_SLLQVLIGL
KD: 6.44e−09
Kass: 1.55e+00, kdiss: 1.00e−08
1318_ID.2874_T8_SLLQHLITL
KD: 5.17e−09
Kass: 1.59e+05, kdiss: 8.23e−04
1319_ID.2875_G3_SLGQHLIGL
KD: 6.30e−09
Kass: 2.03e+05, kdiss: 1.28e−03
1320_ID.2876_N3_SLNQHLIGL
KD: 9.62e−09
Kass: 1.67e+05, kdiss: 1.61e−03
1321_ID.2877_N7_SLLQHLNGL
KD:−
Kass:−, , kdiss: 2.88e−02
1322_ID.2879_W7_SLLQHLWGL
KD: 4.82e−08
Kass: 1.42e+05, kdiss: 6.83e−03
1323_ID.2881_M3_SLMQHLIGL
KD: 5.79e−09
Kass: 1.48e+05, kdiss: 8.56e−04
1324_ID.2883_N8_SLLQHLINL
KD: 5.34e−08
Kass: 9.37e+04, kdiss: 5.00e−03
1325_ID.2885_S8_SLLQHLISL
KD:−
Kass:−, , kdiss: 1.58e+00
1326_ID.2887_N8_SLLQHLINL
KD: 4.04e−08
Kass: 1.19e+05, kdiss: 4.79e−03
1327_ID.2888_G4_SLLGHLIGL
KD: 8.80e−09
Kass: 1.53e+05, kdiss: 1.34e−03
1328_ID.2889_L7_SLLQHLLGL
KD: 6.92e−09
Kass: 1.74e+05, kdiss: 1.21e−03
1329_ID.289_H6_SLLQHHIGL
KD:−
Kass:−, , kdiss: 8.76e−03
1330_ID.2891_P1_PLLQHLIGL
KD:−
Kass:−, , kdiss: 1.53e+00
1331_ID.2892_G4_SLLGHLIGL
KD: 7.92e−09
Kass: 1.61e+05, kdiss: 1.27e−03
1332_ID.2894_V6_SLLQHVIGL
KD: 6.57e−09
Kass: 1.63e+05, kdiss: 1.07e−03
1333_ID.2895_WT_SLLQHLIGL
KD: 5.46e−09
Kass: 1.83e+05, kdiss: 9.98e−04
1334_ID.2896_L4_SLLLHLIGL
KD:−
Kass:−, , kdiss: 1.64e−01
1335_ID.2899_E6_SLLQHEIGL
KD: 4.33e−07
Kass: 2.19e+04, kdiss: 9.51e−03
1336_ID.29_R7_SLLQHLRGL
KD:−
Kass:−, , kdiss: 1.95e−01
1337_ID.2902_N4_SLLNHLIGL
KD: 4.79e−09
Kass: 1.78e+05, kdiss: 8.52e−04
1338_ID.2903_H6_SLLQHHIGL
KD: 8.99e−09
Kass: 1.48e+05, kdiss: 1.33e−03
1339_ID.2905_N7_SLLQHLNGL
KD:−
Kass:−, , kdiss: 3.82e−02
1340_ID.2908_D3_SLDQHLIGL
KD: 8.45e−09
Kass: 1.70e+05, kdiss: 1.44e−03
1341_ID.2909_D7_SLLQHLDGL
KD: 2.84e−08
Kass: 4.65e+04, kdiss: 1.32e−03
1342_ID.291_W7_SLLQHLWGL
KD: 4.45e−07
Kass: 8.44e+03, kdiss: 3.76e−03
1343_ID.2910_N3_SLNQHLIGL
KD: 9.90e−09
Kass: 1.89e+05, kdiss: 1.87e−03
1344_ID.2911_H6_SLLQHHIGL
KD: 5.88e−09
Kass: 1.67e+05, kdiss: 9.80e−04
1345_ID.2912_K4_SLLKHLIGL
KD: 5.14e−09
Kass: 2.57e+05, kdiss: 1.32e−03
1346_ID.2913_I1_ILLQHLIGL
KD:−
Kass:−, , kdiss: 6.57e−02
1347_ID.2914_M5_SLLQMLIGL
KD:−
Kass:−, , kdiss: 5.94e−02
1348_ID.2915_H8_SLLQHLIHL
KD:−
Kass:−, , kdiss: 1.45e−02
1349_ID.2916_W4_SLLWHLIGL
KD: 6.41e−09
Kass: 2.00e+05, kdiss: 1.28e−03
1350_ID.2918_A5_SLLQALIGL
KD: 7.59e−08
Kass: 1.56e+05, kdiss: 1.18e−02
1351_ID.2919_G3_SLGQHLIGL
KD: 2.80e−08
Kass: 1.64e+05, kdiss: 4.59e−03
1352_ID.2920_R4_SLLRHLIGL
KD: 4.36e−09
Kass: 2.76e+05, kdiss: 1.20e−03
1353_ID.2923_I1_ILLQHLIGL
KD: 1.26e−08
Kass: 1.76e+05, kdiss: 2.22e−03
1354_ID.2925_S7_SLLQHLSGL
KD:−
Kass:−, , kdiss: 1.75e−02
1355_ID.2926_L5_SLLQLLIGL
KD:−
Kass:−, , kdiss: 5.01e−02
1356_ID.2927_K8_SLLQHLIKL
KD: 1.40e−07
Kass: 6.33e+04, kdiss: 8.89e−03
1357_ID.2928_I4_SLLIHLIGL
KD:−
Kass:−, , kdiss: 3.74e−01
1358_ID.2929_K8_SLLQHLIKL
KD: 1.42e−07
Kass: 6.48e+04, kdiss: 9.18e−03
1359_ID.293_Q6_SLLQHQIGL
KD: 7.26e−09
Kass: 1.67e+05, kdiss: 1.21e−03
1360_ID.2931_N1_NLLQHLIGL
KD:−
Kass:−, , kdiss: 1.50e+00
1361_ID.2932_G4_SLLGHLIGL
KD:−
Kass:−, , kdiss: 1.49e+00
1362_ID.2933_I1_ILLQHLIGL
KD:−
Kass:−, , kdiss: 3.83e−01
1363_ID.2934_A4_SLLAHLIGL
KD:−
Kass:−, , kdiss: 1.60e+00
1364_ID.2935_L8_SLLQHLILL
KD: 3.57e−08
Kass: 9.94e+04, kdiss: 3.55e−03
1365_ID.2937_M1_MLLQHLIGL
KD: 1.86e−08
Kass: 1.56e+05, kdiss: 2.91e−03
1366_ID.2939_Y6_SLLQHYIGL
KD: 2.04e−09
Kass: 1.78e+05, kdiss: 3.63e−04
1367_ID.294_Q3_SLQQHLIGL
KD: 5.42e−09
Kass: 2.02e+05, kdiss: 1.09e−03
1368_ID.2940_D4_SLLDHLIGL
KD: 7.84e−08
Kass: 3.98e+04, kdiss: 3.12e−03
1369_ID.2943_W7_SLLQHLWGL
KD: 1.76e−08
Kass: 5.76e+04, kdiss: 1.01e−03
1370_ID.2945_Q1_QLLQHLIGL
KD: 1.37e−08
Kass: 1.55e+05, kdiss: 2.11e−03
1371_ID.2946_D3_SLDQHLIGL
KD: 5.98e−14
Kass: 1.67e+05, kdiss: 1.00e−08
1372_ID.2948_L5_SLLQLLIGL
KD:−
Kass:−, , kdiss: 3.45e−02
1373_ID.2949_F3_SLFQHLIGL
KD: 2.02e−08
Kass: 1.74e+05, kdiss: 3.52e−03
1374_ID.295_V8_SLLQHLIVL
KD: 2.56e−08
Kass: 8.76e+04, kdiss: 2.24e−03
1375_ID.2950_F4_SLLFHLIGL
KD: 5.42e−09
Kass: 1.79e+05, kdiss: 9.71e−04
1376_ID.2951_S7_SLLQHLSGL
KD: 1.78e−07
Kass: 6.92e+04, kdiss: 1.23e−02
1377_ID.2953_S8_SLLQHLISL
KD: 7.99e−09
Kass: 1.40e+05, kdiss: 1.12e−03
1378_ID.2954_W4_SLLWHLIGL
KD: 5.55e−09
Kass: 1.63e+05, kdiss: 9.08e−04
1379_ID.2955_A7_SLLQHLAGL
KD: 2.59e−08
Kass: 1.68e+05, kdiss: 4.35e−03
1380_ID.2956_D4_SLLDHLIGL
KD: 4.48e−13
Kass: 7.12e+04, kdiss: 3.19e−08
1381_ID.2958_G5_SLLQGLIGL
KD:−
Kass:−, , kdiss: 2.60e−02
1382_ID.2959_Q6_SLLQHQIGL
KD: 5.41e−09
Kass: 1.42e+05, kdiss: 7.70e−04
1383_ID.2963_D1_DLLQHLIGL
KD: 7.01e−09
Kass: 2.77e+05, kdiss: 1.94e−03
1384_ID.2964_D3_SLDQHLIGL
KD: 5.93e−09
Kass: 1.46e+05, kdiss: 8.64e−04
1385_ID.2965_H8_SLLQHLIHL
KD: 5.75e−07
Kass: 1.75e+04, kdiss: 1.01e−02
1386_ID.2966_S4_SLLSHLIGL
KD: 1.05e−08
Kass: 1.53e+05, kdiss: 1.61e−03
1387_ID.2967_E7_SLLQHLEGL
KD: 4.24e−08
Kass: 2.18e+04, kdiss: 9.27e−04
1388_ID.2968_V4_SLLVHLIGL
KD: 2.94e−09
Kass: 1.82e+05, kdiss: 5.34e−04
1389_ID.2969_G3_SLGQHLIGL
KD: 1.33e−09
Kass: 2.14e+05, kdiss: 2.83e−04
1390_ID.2970_G4_SLLGHLIGL
KD: 9.05e−09
Kass: 1.69e+05, kdiss: 1.53e−03
1391_ID.2971_W8_SLLQHLIWL
KD:−
Kass:−, , kdiss: 5.59e−02
1392_ID.2973_D6_SLLQHDIGL
KD: 5.80e−08
Kass: 9.54e+04, kdiss: 5.54e−03
1393_ID.2974_S5_SLLQSLIGL
KD: 3.06e−07
Kass: 5.88e+04, kdiss: 1.80e−02
1394_ID.2975_Y8_SLLQHLIYL
KD:−
Kass:−, , kdiss: 3.33e−02
1395_ID.2977_Q8_SLLQHLIQL
KD: 5.74e−08
Kass: 5.62e+04, kdiss: 3.23e−03
1396_ID.2979_A8_SLLQHLIAL
KD: 7.72e−09
Kass: 1.45e+05, kdiss: 1.12e−03
1397_ID.2982_G5_SLLQGLIGL
KD:−
Kass:−, , kdiss: 3.40e−02
1398_ID.2983_P1_PLLQHLIGL
KD: 4.81e−09
Kass: 2.10e+05, kdiss: 1.01e−03
1399_ID.2987_K3_SLKQHLIGL
KD: 4.28e−09
Kass: 2.29e+05, kdiss: 9.81e−04
1400_ID.2988_I5_SLLQILIGL
KD:−
Kass:−, , kdiss: 1.40e−01
value
10000
5000
0
0
250
500
750
1000
Time

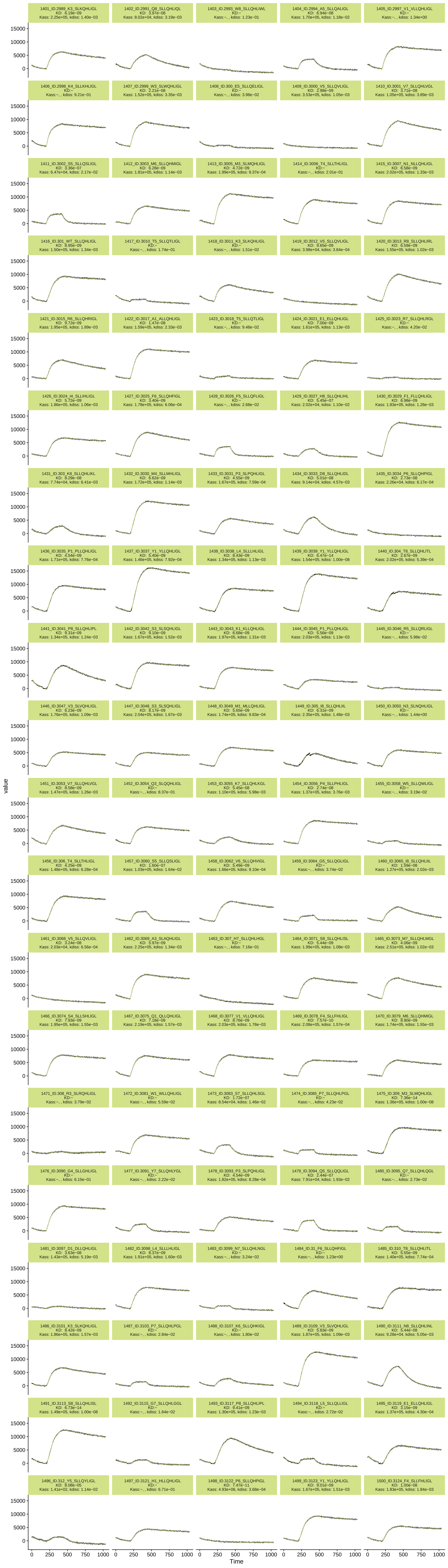
1401_ID.2989_K3_SLKQHLIGL
KD: 6.19e−09
Kass: 2.25e+05, kdiss: 1.40e−03
1402_ID.2991_Q8_SLLQHLIQL
KD: 3.97e−08
Kass: 8.02e+04, kdiss: 3.19e−03
1403_ID.2993_W8_SLLQHLIWL
KD:−
Kass:−, , kdiss: 1.23e−01
1404_ID.2994_A5_SLLQALIGL
KD: 6.94e−08
Kass: 1.70e+05, kdiss: 1.18e−02
1405_ID.2997_V1_VLLQHLIGL
KD:−
Kass:−, , kdiss: 1.34e+00
1406_ID.2998_K4_SLLKHLIGL
KD:−
Kass:−, , kdiss: 9.21e−01
1407_ID.2999_W3_SLWQHLIGL
KD: 2.21e−08
Kass: 1.52e+05, kdiss: 3.35e−03
1408_ID.300_E5_SLLQELIGL
KD:−
Kass:−, , kdiss: 3.98e−02
1409_ID.3000_V5_SLLQVLIGL
KD: 2.98e−09
Kass: 3.53e+05, kdiss: 1.05e−03
1410_ID.3001_V7_SLLQHLVGL
KD: 3.71e−08
Kass: 1.05e+05, kdiss: 3.89e−03
1411_ID.3002_S5_SLLQSLIGL
KD: 3.36e−07
Kass: 6.47e+04, kdiss: 2.17e−02
1412_ID.3003_M6_SLLQHMIGL
KD: 6.28e−09
Kass: 1.81e+05, kdiss: 1.14e−03
1413_ID.3005_M3_SLMQHLIGL
KD: 4.72e−09
Kass: 1.99e+05, kdiss: 9.37e−04
1414_ID.3006_T4_SLLTHLIGL
KD:−
Kass:−, , kdiss: 2.01e−01
1415_ID.3007_N1_NLLQHLIGL
KD: 6.58e−09
Kass: 2.02e+05, kdiss: 1.33e−03
1416_ID.301_WT_SLLQHLIGL
KD: 8.95e−09
Kass: 1.50e+05, kdiss: 1.34e−03
1417_ID.3010_T5_SLLQTLIGL
KD:−
Kass:−, , kdiss: 1.74e−01
1418_ID.3011_K3_SLKQHLIGL
KD:−
Kass:−, , kdiss: 1.51e−02
1419_ID.3012_V5_SLLQVLIGL
KD: 9.65e−09
Kass: 3.98e+04, kdiss: 3.84e−04
1420_ID.3013_R8_SLLQHLIRL
KD: 6.59e−09
Kass: 1.55e+05, kdiss: 1.02e−03
1421_ID.3015_R6_SLLQHRIGL
KD: 9.72e−09
Kass: 1.95e+05, kdiss: 1.89e−03
1422_ID.3017_A1_ALLQHLIGL
KD: 1.47e−08
Kass: 1.59e+05, kdiss: 2.33e−03
1423_ID.3018_T5_SLLQTLIGL
KD:−
Kass:−, , kdiss: 9.48e−02
1424_ID.3021_E1_ELLQHLIGL
KD: 7.00e−09
Kass: 1.61e+05, kdiss: 1.13e−03
1425_ID.3023_R7_SLLQHLRGL
KD:−
Kass:−, , kdiss: 4.20e−02
1426_ID.3024_I4_SLLIHLIGL
KD: 5.72e−09
Kass: 1.86e+05, kdiss: 1.06e−03
1427_ID.3025_F6_SLLQHFIGL
KD: 3.40e−09
Kass: 1.78e+05, kdiss: 6.06e−04
1428_ID.3026_F5_SLLQFLIGL
KD:−
Kass:−, , kdiss: 2.68e−02
1429_ID.3027_H8_SLLQHLIHL
KD: 5.45e−07
Kass: 2.02e+04, kdiss: 1.10e−02
1430_ID.3029_F1_FLLQHLIGL
KD: 6.98e−09
Kass: 1.83e+05, kdiss: 1.28e−03
1431_ID.303_K8_SLLQHLIKL
KD: 8.29e−08
Kass: 7.74e+04, kdiss: 6.41e−03
1432_ID.3030_M4_SLLMHLIGL
KD: 6.62e−09
Kass: 1.72e+05, kdiss: 1.14e−03
1433_ID.3031_P3_SLPQHLIGL
KD: 4.55e−09
Kass: 1.67e+05, kdiss: 7.59e−04
1434_ID.3033_D8_SLLQHLIDL
KD: 5.01e−08
Kass: 9.14e+04, kdiss: 4.57e−03
1435_ID.3034_P6_SLLQHPIGL
KD: 2.73e−08
Kass: 2.26e+04, kdiss: 6.17e−04
1436_ID.3035_P1_PLLQHLIGL
KD: 4.54e−09
Kass: 1.71e+05, kdiss: 7.76e−04
1437_ID.3037_Y1_YLLQHLIGL
KD: 5.40e−09
Kass: 1.46e+05, kdiss: 7.92e−04
1438_ID.3038_L4_SLLLHLIGL
KD: 8.43e−09
Kass: 1.34e+05, kdiss: 1.13e−03
1439_ID.3039_Y1_YLLQHLIGL
KD: 6.47e−14
Kass: 1.54e+05, kdiss: 1.00e−08
1440_ID.304_T8_SLLQHLITL
KD: 2.67e−09
Kass: 2.02e+05, kdiss: 5.39e−04
1441_ID.3041_P8_SLLQHLIPL
KD: 9.31e−09
Kass: 1.34e+05, kdiss: 1.24e−03
1442_ID.3042_S3_SLSQHLIGL
KD: 9.10e−09
Kass: 1.67e+05, kdiss: 1.52e−03
1443_ID.3043_K1_KLLQHLIGL
KD: 6.68e−09
Kass: 1.97e+05, kdiss: 1.31e−03
1444_ID.3045_P1_PLLQHLIGL
KD: 5.56e−09
Kass: 2.03e+05, kdiss: 1.13e−03
1445_ID.3046_R5_SLLQRLIGL
KD:−
Kass:−, , kdiss: 5.98e−02
1446_ID.3047_V3_SLVQHLIGL
KD: 6.23e−09
Kass: 1.76e+05, kdiss: 1.09e−03
1447_ID.3048_S3_SLSQHLIGL
KD: 8.17e−09
Kass: 2.04e+05, kdiss: 1.67e−03
1448_ID.3049_M1_MLLQHLIGL
KD: 5.65e−09
Kass: 1.74e+05, kdiss: 9.83e−04
1449_ID.305_I8_SLLQHLIIL
KD: 6.31e−09
Kass: 2.35e+05, kdiss: 1.48e−03
1450_ID.3050_N3_SLNQHLIGL
KD:−
Kass:−, , kdiss: 1.44e+00
1451_ID.3053_V7_SLLQHLVGL
KD: 8.58e−09
Kass: 1.47e+05, kdiss: 1.26e−03
1452_ID.3054_Q3_SLQQHLIGL
KD:−
Kass:−, , kdiss: 8.37e−01
1453_ID.3055_K7_SLLQHLKGL
KD: 5.45e−08
Kass: 1.10e+05, kdiss: 5.98e−03
1454_ID.3056_P4_SLLPHLIGL
KD: 2.74e−08
Kass: 1.37e+05, kdiss: 3.76e−03
1455_ID.3058_W5_SLLQWLIGL
KD:−
Kass:−, , kdiss: 3.19e−02
1456_ID.306_T4_SLLTHLIGL
KD: 4.25e−09
Kass: 1.48e+05, kdiss: 6.28e−04
1457_ID.3060_S5_SLLQSLIGL
KD: 1.60e−07
Kass: 1.03e+05, kdiss: 1.64e−02
1458_ID.3062_V6_SLLQHVIGL
KD: 5.49e−09
Kass: 1.66e+05, kdiss: 9.10e−04
1459_ID.3064_G5_SLLQGLIGL
KD:−
Kass:−, , kdiss: 3.74e−02
1460_ID.3065_I8_SLLQHLIIL
KD: 1.59e−08
Kass: 1.27e+05, kdiss: 2.02e−03
1461_ID.3068_V5_SLLQVLIGL
KD: 3.24e−08
Kass: 2.03e+04, kdiss: 6.56e−04
1462_ID.3069_A3_SLAQHLIGL
KD: 5.97e−09
Kass: 2.25e+05, kdiss: 1.34e−03
1463_ID.307_H7_SLLQHLHGL
KD:−
Kass:−, , kdiss: 7.16e−01
1464_ID.3071_S8_SLLQHLISL
KD: 5.44e−09
Kass: 1.99e+05, kdiss: 1.08e−03
1465_ID.3073_M7_SLLQHLMGL
KD: 4.06e−09
Kass: 2.51e+05, kdiss: 1.02e−03
1466_ID.3074_S4_SLLSHLIGL
KD: 7.93e−09
Kass: 1.95e+05, kdiss: 1.55e−03
1467_ID.3075_Q1_QLLQHLIGL
KD: 7.18e−09
Kass: 2.19e+05, kdiss: 1.57e−03
1468_ID.3077_V1_VLLQHLIGL
KD: 8.76e−09
Kass: 2.03e+05, kdiss: 1.78e−03
1469_ID.3078_F4_SLLFHLIGL
KD: 7.57e−10
Kass: 2.08e+05, kdiss: 1.57e−04
1470_ID.3079_M6_SLLQHMIGL
KD: 8.90e−09
Kass: 1.74e+05, kdiss: 1.55e−03
1471_ID.308_R3_SLRQHLIGL
KD:−
Kass:−, , kdiss: 3.79e−02
1472_ID.3081_W1_WLLQHLIGL
KD:−
Kass:−, , kdiss: 5.59e−02
1473_ID.3083_S7_SLLQHLSGL
KD: 1.72e−07
Kass: 8.54e+04, kdiss: 1.46e−02
1474_ID.3085_P7_SLLQHLPGL
KD:−
Kass:−, , kdiss: 4.23e−02
1475_ID.309_M3_SLMQHLIGL
KD: 7.36e−14
Kass: 1.36e+05, kdiss: 1.00e−08
1476_ID.3090_G4_SLLGHLIGL
KD:−
Kass:−, , kdiss: 6.15e−01
1477_ID.3091_Y7_SLLQHLYGL
KD:−
Kass:−, , kdiss: 2.22e−02
1478_ID.3093_P3_SLPQHLIGL
KD: 4.54e−09
Kass: 1.82e+05, kdiss: 8.28e−04
1479_ID.3094_Q5_SLLQQLIGL
KD: 2.44e−07
Kass: 7.91e+04, kdiss: 1.93e−02
1480_ID.3095_Q7_SLLQHLQGL
KD:−
Kass:−, , kdiss: 2.73e−02
1481_ID.3097_D1_DLLQHLIGL
KD: 3.63e−08
Kass: 1.43e+05, kdiss: 5.19e−03
1482_ID.3098_L4_SLLLHLIGL
KD: 8.37e−09
Kass: 1.91e+05, kdiss: 1.60e−03
1483_ID.3099_N7_SLLQHLNGL
KD:−
Kass:−, , kdiss: 3.24e−02
1484_ID.31_F6_SLLQHFIGL
KD:−
Kass:−, , kdiss: 1.23e+00
1485_ID.310_T8_SLLQHLITL
KD: 5.55e−09
Kass: 1.40e+05, kdiss: 7.74e−04
1486_ID.3101_K3_SLKQHLIGL
KD: 8.42e−09
Kass: 1.86e+05, kdiss: 1.57e−03
1487_ID.3103_P7_SLLQHLPGL
KD:−
Kass:−, , kdiss: 2.84e−02
1488_ID.3107_K6_SLLQHKIGL
KD:−
Kass:−, , kdiss: 1.80e−02
1489_ID.3109_V3_SLVQHLIGL
KD: 5.83e−09
Kass: 1.87e+05, kdiss: 1.09e−03
1490_ID.3111_N8_SLLQHLINL
KD: 5.44e−08
Kass: 9.28e+04, kdiss: 5.05e−03
1491_ID.3113_S8_SLLQHLISL
KD: 6.73e−14
Kass: 1.49e+05, kdiss: 1.00e−08
1492_ID.3115_G7_SLLQHLGGL
KD:−
Kass:−, , kdiss: 1.64e−02
1493_ID.3117_P8_SLLQHLIPL
KD: 9.41e−09
Kass: 1.30e+05, kdiss: 1.23e−03
1494_ID.3118_L5_SLLQLLIGL
KD:−
Kass:−, , kdiss: 2.72e−02
1495_ID.3119_E1_ELLQHLIGL
KD: 3.15e−09
Kass: 1.37e+05, kdiss: 4.30e−04
1496_ID.312_Y5_SLLQYLIGL
KD: 8.08e−05
Kass: 1.41e+02, kdiss: 1.14e−02
1497_ID.3121_H1_HLLQHLIGL
KD:−
Kass:−, , kdiss: 6.71e−01
1498_ID.3122_P6_SLLQHPIGL
KD: 7.47e−11
Kass: 4.93e+06, kdiss: 3.68e−04
1499_ID.3123_Y1_YLLQHLIGL
KD: 9.01e−09
Kass: 1.67e+05, kdiss: 1.51e−03
1500_ID.3124_F4_SLLFHLIGL
KD: 1.00e−08
Kass: 1.83e+05, kdiss: 1.84e−03
value
Time
0
5000
10000
15000
0
250
500
750
1000

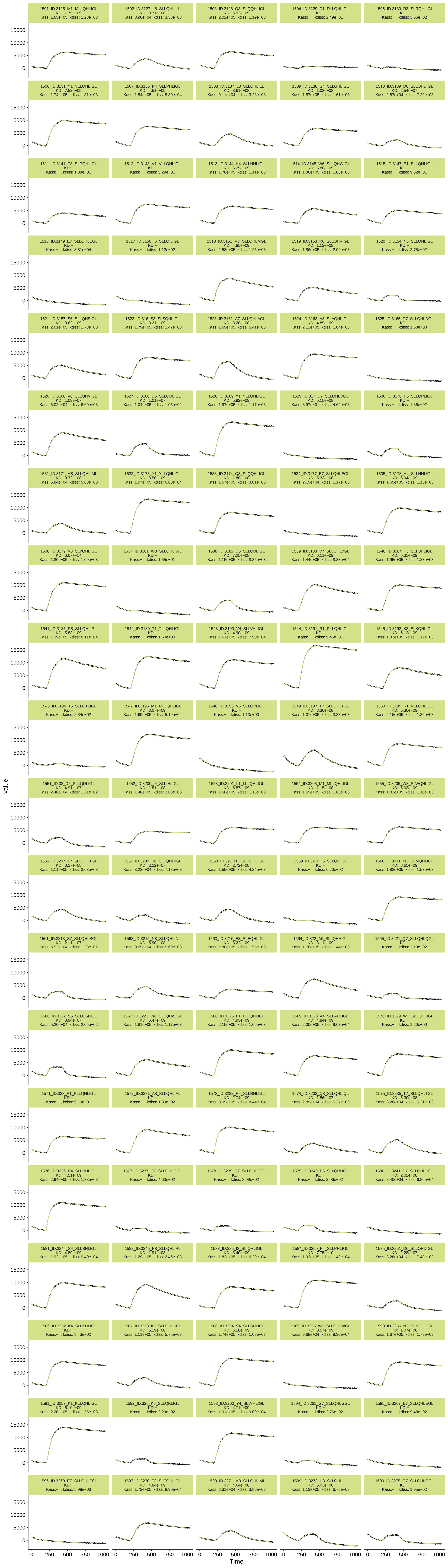

1501_ID.3125_M1_MLLQHLIGL KD: 7.75e−09 Kass: 1.65e+05, kdiss: 1.28e−03
1502_ID.3127_L8_SLLQHLILL KD: 3.71e−08 Kass: 8.98e+04, kdiss: 3.33e−03
1503_ID.3128_Q3_SLQQHLIGL KD: 5.92e−09 Kass: 2.02e+05, kdiss: 1.19e−03
1504_ID.3129_D1_DLLQHLIGL KD:− Kass:− , kdiss: 2.49e−01
1505_ID.3130_R3_SLRQHLIGL KD:− Kass:− , kdiss: 3.56e−02
1506_ID.3131_Y1_YLLQHLIGL KD: 7.52e−09 Kass: 1.74e+05, kdiss: 1.31e−03
1507_ID.3136_P4_SLLPHLIGL KD: 4.51e−09 Kass: 1.84e+05, kdiss: 8.30e−04
1508_ID.3137_L8_SLLQHLILL KD: 3.61e−08 Kass: 9.11e+04, kdiss: 3.28e−03
1509_ID.3138_G4_SLLGHLIGL KD: 1.03e−08 Kass: 1.57e+05, kdiss: 1.61e−03
1510_ID.3139_D6_SLLQHDIGL KD: 2.54e−07 Kass: 2.87e+04, kdiss: 7.29e−03
1511_ID.3141_P3_SLPQHLIGL KD:− Kass:− , kdiss: 1.38e−01
1512_ID.3143_V1_VLLQHLIGL KD:− Kass:− , kdiss: 5.29e−01
1513_ID.3144_H4_SLLHHLIGL KD: 6.25e−09 Kass: 1.78e+05, kdiss: 1.11e−03
1514_ID.3145_W6_SLLQHWIGL KD: 5.80e−09 Kass: 1.86e+05, kdiss: 1.08e−03
1515_ID.3147_E1_ELLQHLIGL KD:− Kass:− , kdiss: 9.62e−01
1516_ID.3149_E7_SLLQHLEGL KD:− Kass:− , kdiss: 8.81e−04
1517_ID.3150_I5_SLLQILIGL KD:− Kass:− , kdiss: 1.12e−02
1518_ID.3151_M7_SLLQHLMGL KD: 4.85e−09 Kass: 2.58e+05, kdiss: 1.25e−03
1519_ID.3153_R6_SLLQHRIGL KD: 1.12e−08 Kass: 1.86e+05, kdiss: 2.09e−03
1520_ID.3154_N5_SLLQNLIGL KD:− Kass:− , kdiss: 2.78e−02
1521_ID.3157_S6_SLLQHSIGL KD: 8.62e−09 Kass: 2.01e+05, kdiss: 1.73e−03
1522_ID.316_S3_SLSQHLIGL KD: 8.21e−09 Kass: 1.79e+05, kdiss: 1.47e−03
1523_ID.3161_A7_SLLQHLAGL KD: 3.20e−08 Kass: 1.69e+05, kdiss: 5.41e−03
1524_ID.3163_A3_SLAQHLIGL KD: 4.89e−09 Kass: 2.12e+05, kdiss: 1.04e−03
1525_ID.3165_D7_SLLQHLDGL KD:− Kass:− , kdiss: 1.50e+00
1526_ID.3166_V6_SLLQHVIGL KD: 1.09e−07 Kass: 6.02e+04, kdiss: 6.60e−03
1527_ID.3168_D5_SLLQDLIGL KD: 1.01e−07 Kass: 1.04e+05, kdiss: 1.05e−02
1528_ID.3169_Y1_YLLQHLIGL KD: 5.92e−09 Kass: 1.97e+05, kdiss: 1.17e−03
1529_ID.317_D7_SLLQHLDGL KD: 5.15e−08 Kass: 8.97e−01, kdiss: 4.62e−08
1530_ID.3170_P5_SLLQPLIGL KD:− Kass:− , kdiss: 1.86e−02
1531_ID.3171_M8_SLLQHLIML KD: 9.72e−08 Kass: 5.84e+04, kdiss: 5.68e−03
1532_ID.3173_Y1_YLLQHLIGL KD: 3.50e−09 Kass: 1.97e+05, kdiss: 6.88e−04
1533_ID.3174_Q3_SLQQHLIGL KD: 1.80e−08 Kass: 1.67e+05, kdiss: 3.01e−03
1534_ID.3177_E7_SLLQHLEGL KD: 5.33e−08 Kass: 2.19e+04, kdiss: 1.17e−03
1535_ID.3178_H4_SLLHHLIGL KD: 6.94e−09 Kass: 1.65e+05, kdiss: 1.15e−03
1536_ID.3179_V3_SLVQHLIGL KD: 6.07e−14 Kass: 1.80e+05, kdiss: 1.09e−08
1537_ID.3181_W8_SLLQHLIWL KD:− Kass:− , kdiss: 1.55e−01
1538_ID.3182_D5_SLLQDLIGL KD: 7.25e−08 Kass: 1.15e+05, kdiss: 8.35e−03
1539_ID.3183_V7_SLLQHLVGL KD: 6.11e−09 Kass: 1.44e+05, kdiss: 8.83e−04
1540_ID.3184_T3_SLTQHLIGL KD: 6.31e−09 Kass: 1.95e+05, kdiss: 1.23e−03
1541_ID.3185_R8_SLLQHLIRL KD: 5.83e−09 Kass: 1.39e+05, kdiss: 8.11e−04
1542_ID.3189_T1_TLLQHLIGL KD:− Kass:− , kdiss: 1.60e+00
1543_ID.3190_V4_SLLVHLIGL KD: 4.90e−09 Kass: 1.61e+05, kdiss: 7.90e−04
1544_ID.3191_R1_RLLQHLIGL KD:− Kass:− , kdiss: 9.45e−01
1545_ID.3193_K3_SLKQHLIGL KD: 6.12e−09 Kass: 1.83e+05, kdiss: 1.12e−03
1546_ID.3194_T5_SLLQTLIGL KD:− Kass:− , kdiss: 2.34e−02
1547_ID.3195_M1_MLLQHLIGL KD: 3.07e−09 Kass: 1.69e+05, kdiss: 5.19e−04
1548_ID.3196_V5_SLLQVLIGL KD:− Kass:− , kdiss: 1.13e+00
1549_ID.3197_T7_SLLQHLTGL KD: 3.30e−08 Kass: 1.01e+05, kdiss: 3.33e−03
1550_ID.3199_R1_RLLQHLIGL KD: 6.30e−09 Kass: 2.16e+05, kdiss: 1.36e−03
1551_ID.32_D5_SLLQDLIGL KD: 4.91e−07 Kass: 2.46e+04, kdiss: 1.21e−02
1552_ID.3200_I4_SLLIHLIGL KD: 1.81e−08 Kass: 1.48e+05, kdiss: 2.68e−03
1553_ID.3201_L1_LLLQHLIGL KD: 6.87e−09 Kass: 1.68e+05, kdiss: 1.15e−03
1554_ID.3203_M1_MLLQHLIGL KD: 1.15e−08 Kass: 1.59e+05, kdiss: 1.83e−03
1555_ID.3205_W3_SLWQHLIGL KD: 6.03e−09 Kass: 1.82e+05, kdiss: 1.10e−03
1556_ID.3207_T7_SLLQHLTGL KD: 3.27e−08 Kass: 1.11e+05, kdiss: 3.63e−03
1557_ID.3209_D6_SLLQHDIGL KD: 2.22e−07 Kass: 3.23e+04, kdiss: 7.18e−03
1558_ID.321_H3_SLHQHLIGL KD: 2.72e−08 Kass: 1.55e+05, kdiss: 4.24e−03
1559_ID.3210_I5_SLLQILIGL KD:− Kass:− , kdiss: 6.20e−02
1560_ID.3211_M3_SLMQHLIGL KD: 8.65e−09 Kass: 1.82e+05, kdiss: 1.57e−03
1561_ID.3213_S7_SLLQHLSGL KD: 2.11e−07 Kass: 6.52e+04, kdiss: 1.38e−02
1562_ID.3215_N8_SLLQHLINL KD: 5.90e−08 Kass: 9.65e+04, kdiss: 5.69e−03
1563_ID.3216_E3_SLEQHLIGL KD: 8.22e−09 Kass: 1.88e+05, kdiss: 1.55e−03
1564_ID.322_A6_SLLQHAIGL KD: 8.12e−09 Kass: 1.78e+05, kdiss: 1.44e−03
1565_ID.3221_Q7_SLLQHLQGL KD:− Kass:− , kdiss: 3.13e−02
1566_ID.3222_S5_SLLQSLIGL KD: 3.94e−07 Kass: 5.20e+04, kdiss: 2.05e−02
1567_ID.3223_W6_SLLQHWIGL KD: 6.47e−09 Kass: 1.81e+05, kdiss: 1.17e−03
1568_ID.3225_F1_FLLQHLIGL KD: 4.93e−09 Kass: 2.15e+05, kdiss: 1.06e−03
1569_ID.3228_A4_SLLAHLIGL KD: 4.84e−09 Kass: 2.00e+05, kdiss: 9.67e−04
1570_ID.3229_WT_SLLQHLIGL KD:− Kass:− , kdiss: 1.20e+00
1571_ID.323_P1_PLLQHLIGL KD:− Kass:− , kdiss: 9.19e−01
1572_ID.3231_A8_SLLQHLIAL KD:− Kass:− , kdiss: 1.39e−02
1573_ID.3232_R4_SLLRHLIGL KD: 2.73e−09 Kass: 3.09e+05, kdiss: 8.44e−04
1574_ID.3233_Q8_SLLQHLIQL KD: 1.86e−07 Kass: 2.89e+04, kdiss: 5.37e−03
1575_ID.3235_T7_SLLQHLTGL KD: 6.30e−08 Kass: 8.28e+04, kdiss: 5.21e−03
1576_ID.3236_R4_SLLRHLIGL KD: 4.51e−09 Kass: 2.94e+05, kdiss: 1.33e−03
1577_ID.3237_G7_SLLQHLGGL KD:− Kass:− , kdiss: 4.63e−02
1578_ID.3239_Q7_SLLQHLQGL KD:− Kass:− , kdiss: 3.09e−02
1579_ID.3240_P5_SLLQPLIGL KD:− Kass:− , kdiss: 2.06e−02
1580_ID.3241_D7_SLLQHLDGL KD: 2.02e−08 Kass: 3.40e+04, kdiss: 6.86e−04
1581_ID.3244_S4_SLLSHLIGL KD: 4.89e−09 Kass: 1.92e+05, kdiss: 9.40e−04
1582_ID.3245_P8_SLLQHLIPL KD: 1.91e−08 Kass: 1.29e+05, kdiss: 2.46e−03
1583_ID.325_I3_SLIQHLIGL KD: 3.40e−09 Kass: 1.82e+05, kdiss: 6.20e−04
1584_ID.3250_P4_SLLPHLIGL KD: 7.75e−10 Kass: 1.92e+05, kdiss: 1.49e−04
1585_ID.3251_D6_SLLQHDIGL KD: 2.28e−07 Kass: 3.28e+04, kdiss: 7.48e−03
1586_ID.3252_K4_SLLKHLIGL KD:− Kass:− , kdiss: 8.43e−02
1587_ID.3253_K7_SLLQHLKGL KD: 5.19e−08 Kass: 1.11e+05, kdiss: 5.75e−03
1588_ID.3254_S4_SLLSHLIGL KD: 6.18e−09 Kass: 1.74e+05, kdiss: 1.08e−03
1589_ID.3255_W7_SLLQHLWGL KD: 8.57e−09 Kass: 9.68e+04, kdiss: 8.30e−04
1590_ID.3256_N3_SLNQHLIGL KD: 1.07e−08 Kass: 1.67e+05, kdiss: 1.79e−03
1591_ID.3257_K1_KLLQHLIGL KD: 6.10e−09 Kass: 2.20e+05, kdiss: 1.35e−03
1592_ID.326_K5_SLLQKLIGL KD:− Kass:− , kdiss: 2.29e−02
1593_ID.3260_Y4_SLLYHLIGL KD: 4.71e−09 Kass: 1.91e+05, kdiss: 9.00e−04
1594_ID.3261_G7_SLLQHLGGL KD:− Kass:− , kdiss: 2.76e−02
1595_ID.3267_E7_SLLQHLEGL KD:− Kass:− , kdiss: 9.48e−02
1596_ID.3269_E7_SLLQHLEGL KD:− Kass:− , kdiss: 5.98e−03
1597_ID.3270_E3_SLEQHLIGL KD: 4.84e−09 Kass: 1.72e+05, kdiss: 8.30e−04
1598_ID.3271_M8_SLLQHLIML KD: 4.64e−08 Kass: 8.31e+04, kdiss: 3.86e−03
1599_ID.3273_H8_SLLQHLIHL KD: 6.03e−08 Kass: 1.12e+05, kdiss: 6.78e−03
1600_ID.3275_Q7_SLLQHLQGL KD:− Kass:− , kdiss: 1.95e−02
value
15000
10000
5000
0
Time
0 250 500 750 1000

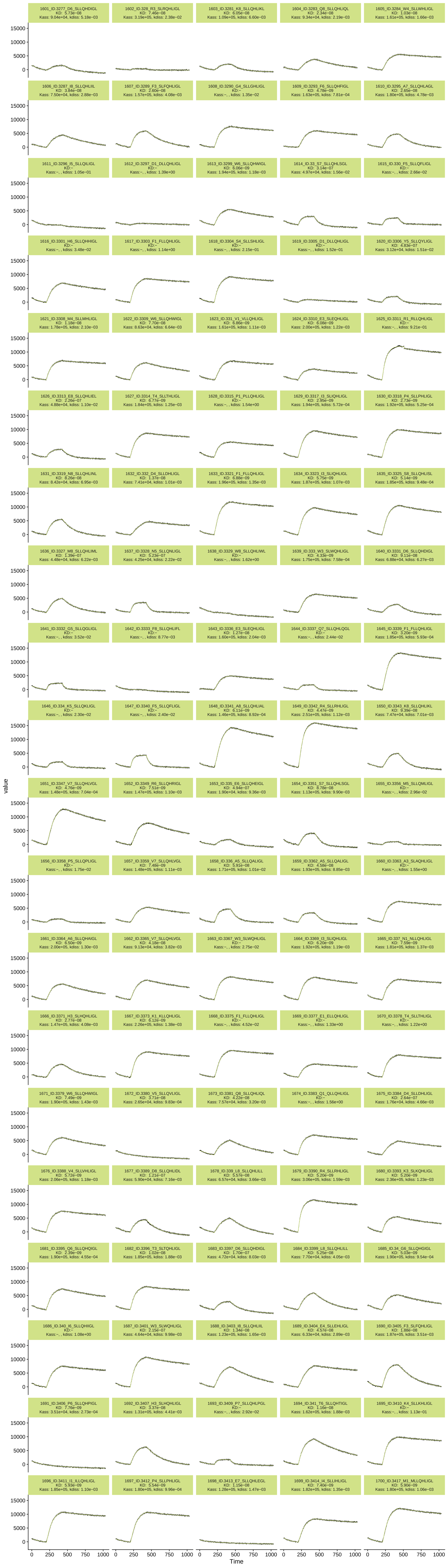

1601_ID.3277_D6_SLLQHDIGL
KD: 5.73e−08
Kass: 9.04e+04, kdiss: 5.18e−03
1602_ID.328_R3_SLRQHLIGL
KD: 7.46e−08
Kass: 3.19e+05, kdiss: 2.38e−02
1603_ID.3281_K8_SLLQHLIKL
KD: 6.05e−08
Kass: 1.09e+05, kdiss: 6.60e−03
1604_ID.3283_Q8_SLLQHLIQL
KD: 2.34e−08
Kass: 9.34e+04, kdiss: 2.19e−03
1605_ID.3284_W4_SLLWHLIGL
KD: 1.03e−08
Kass: 1.61e+05, kdiss: 1.66e−03
1606_ID.3287_I8_SLLQHLIIL
KD: 3.84e−08
Kass: 7.50e+04, kdiss: 2.88e−03
1607_ID.3289_F3_SLFQHLIGL
KD: 2.60e−08
Kass: 1.57e+05, kdiss: 4.08e−03
1608_ID.3290_G4_SLLGHLIGL
KD:−
Kass:−, , kdiss: 1.35e−02
1609_ID.3293_F6_SLLQHFIGL
KD: 4.78e−09
Kass: 1.63e+05, kdiss: 7.81e−04
1610_ID.3295_A7_SLLQHLAGL
KD: 2.65e−08
Kass: 1.80e+05, kdiss: 4.78e−03
1611_ID.3296_I5_SLLQILIGL
KD:−
Kass:−, , kdiss: 1.05e−01
1612_ID.3297_D1_DLLQHLIGL
KD:−
Kass:−, , kdiss: 1.39e+00
1613_ID.3299_W6_SLLQHWIGL
KD: 6.06e−09
Kass: 1.94e+05, kdiss: 1.18e−03
1614_ID.33_S7_SLLQHLSGL
KD: 3.14e−07
Kass: 4.97e+04, kdiss: 1.56e−02
1615_ID.330_F5_SLLQFLIGL
KD:−
Kass:−, , kdiss: 2.66e−02
1616_ID.3301_H6_SLLQHHIGL
KD:−
Kass:−, , kdiss: 3.48e−02
1617_ID.3303_F1_FLLQHLIGL
KD:−
Kass:−, , kdiss: 1.14e+00
1618_ID.3304_S4_SLLSHLIGL
KD:−
Kass:−, , kdiss: 2.15e−01
1619_ID.3305_D1_DLLQHLIGL
KD:−
Kass:−, , kdiss: 1.52e−01
1620_ID.3306_Y5_SLLQYLIGL
KD: 4.83e−07
Kass: 3.12e+04, kdiss: 1.51e−02
1621_ID.3308_M4_SLLMHLIGL
KD: 1.18e−08
Kass: 1.78e+05, kdiss: 2.10e−03
1622_ID.3309_W6_SLLQHWIGL
KD: 7.70e−08
Kass: 8.63e+04, kdiss: 6.64e−03
1623_ID.331_V1_VLLQHLIGL
KD: 6.86e−09
Kass: 1.61e+05, kdiss: 1.11e−03
1624_ID.3310_E3_SLEQHLIGL
KD: 6.08e−09
Kass: 2.00e+05, kdiss: 1.22e−03
1625_ID.3311_R1_RLLQHLIGL
KD:−
Kass:−, , kdiss: 9.21e−01
1626_ID.3313_E8_SLLQHLIEL
KD: 2.26e−07
Kass: 4.88e+04, kdiss: 1.10e−02
1627_ID.3314_T4_SLLTHLIGL
KD: 6.77e−09
Kass: 1.84e+05, kdiss: 1.25e−03
1628_ID.3315_P1_PLLQHLIGL
KD:−
Kass:−, , kdiss: 1.54e+00
1629_ID.3317_I3_SLIQHLIGL
KD: 2.95e−09
Kass: 1.94e+05, kdiss: 5.72e−04
1630_ID.3318_P4_SLLPHLIGL
KD: 2.73e−09
Kass: 1.92e+05, kdiss: 5.25e−04
1631_ID.3319_N8_SLLQHLINL
KD: 8.26e−08
Kass: 8.42e+04, kdiss: 6.95e−03
1632_ID.332_D4_SLLDHLIGL
KD: 1.37e−08
Kass: 7.41e+04, kdiss: 1.01e−03
1633_ID.3321_F1_FLLQHLIGL
KD: 6.88e−09
Kass: 1.96e+05, kdiss: 1.35e−03
1634_ID.3323_I3_SLIQHLIGL
KD: 5.75e−09
Kass: 1.87e+05, kdiss: 1.07e−03
1635_ID.3325_S8_SLLQHLISL
KD: 5.14e−09
Kass: 1.85e+05, kdiss: 9.48e−04
1636_ID.3327_M8_SLLQHLIML
KD: 1.39e−07
Kass: 4.48e+04, kdiss: 6.22e−03
1637_ID.3328_N5_SLLQNLIGL
KD: 5.23e−07
Kass: 4.25e+04, kdiss: 2.22e−02
1638_ID.3329_W8_SLLQHLIWL
KD:−
Kass:−, , kdiss: 1.62e+00
1639_ID.333_W3_SLWQHLIGL
KD: 4.33e−09
Kass: 1.75e+05, kdiss: 7.58e−04
1640_ID.3331_D6_SLLQHDIGL
KD: 9.11e−08
Kass: 6.88e+04, kdiss: 6.27e−03
1641_ID.3332_G5_SLLQGLIGL
KD:−
Kass:−, , kdiss: 3.52e−02
1642_ID.3333_F8_SLLQHLIFL
KD:−
Kass:−, , kdiss: 8.77e−03
1643_ID.3336_E3_SLEQHLIGL
KD: 1.27e−08
Kass: 1.60e+05, kdiss: 2.04e−03
1644_ID.3337_Q7_SLLQHLQGL
KD:−
Kass:−, , kdiss: 2.44e−02
1645_ID.3339_F1_FLLQHLIGL
KD: 3.20e−09
Kass: 1.85e+05, kdiss: 5.93e−04
1646_ID.334_K5_SLLQKLIGL
KD:−
Kass:−, , kdiss: 2.30e−02
1647_ID.3340_F5_SLLQFLIGL
KD:−
Kass:−, , kdiss: 2.40e−02
1648_ID.3341_A8_SLLQHLIAL
KD: 6.11e−09
Kass: 1.46e+05, kdiss: 8.92e−04
1649_ID.3342_R4_SLLRHLIGL
KD: 4.47e−09
Kass: 2.51e+05, kdiss: 1.12e−03
1650_ID.3343_K8_SLLQHLIKL
KD: 9.39e−08
Kass: 7.47e+04, kdiss: 7.01e−03
1651_ID.3347_V7_SLLQHLVGL
KD: 4.76e−09
Kass: 1.48e+05, kdiss: 7.04e−04
1652_ID.3349_R6_SLLQHRIGL
KD: 7.51e−09
Kass: 1.47e+05, kdiss: 1.10e−03
1653_ID.335_E6_SLLQHEIGL
KD: 4.94e−07
Kass: 1.90e+04, kdiss: 9.36e−03
1654_ID.3351_S7_SLLQHLSGL
KD: 8.78e−08
Kass: 1.13e+05, kdiss: 9.90e−03
1655_ID.3356_M5_SLLQMLIGL
KD:−
Kass:−, , kdiss: 2.96e−02
1656_ID.3358_P5_SLLQPLIGL
KD:−
Kass:−, , kdiss: 1.75e−02
1657_ID.3359_V7_SLLQHLVGL
KD: 7.48e−09
Kass: 1.48e+05, kdiss: 1.11e−03
1658_ID.336_A5_SLLQALIGL
KD: 5.91e−08
Kass: 1.71e+05, kdiss: 1.01e−02
1659_ID.3362_A5_SLLQALIGL
KD: 4.58e−08
Kass: 1.93e+05, kdiss: 8.85e−03
1660_ID.3363_A3_SLAQHLIGL
KD:−
Kass:−, , kdiss: 1.55e+00
1661_ID.3364_A6_SLLQHAIGL
KD: 6.50e−09
Kass: 2.00e+05, kdiss: 1.30e−03
1662_ID.3365_V7_SLLQHLVGL
KD: 4.18e−08
Kass: 9.13e+04, kdiss: 3.82e−03
1663_ID.3367_W3_SLWQHLIGL
KD:−
Kass:−, , kdiss: 2.75e−02
1664_ID.3369_I3_SLIQHLIGL
KD: 6.20e−09
Kass: 1.92e+05, kdiss: 1.19e−03
1665_ID.337_N1_NLLQHLIGL
KD: 7.59e−09
Kass: 1.81e+05, kdiss: 1.37e−03
1666_ID.3371_H3_SLHQHLIGL
KD: 2.77e−08
Kass: 1.47e+05, kdiss: 4.08e−03
1667_ID.3373_K1_KLLQHLIGL
KD: 6.12e−09
Kass: 2.26e+05, kdiss: 1.38e−03
1668_ID.3375_F1_FLLQHLIGL
KD:−
Kass:−, , kdiss: 4.52e−02
1669_ID.3377_E1_ELLQHLIGL
KD:−
Kass:−, , kdiss: 1.33e+00
1670_ID.3378_T4_SLLTHLIGL
KD:−
Kass:−, , kdiss: 1.22e+00
1671_ID.3379_W6_SLLQHWIGL
KD: 7.49e−09
Kass: 1.90e+05, kdiss: 1.43e−03
1672_ID.3380_V5_SLLQVLIGL
KD: 3.71e−08
Kass: 2.65e+04, kdiss: 9.83e−04
1673_ID.3381_Q8_SLLQHLIQL
KD: 4.22e−08
Kass: 7.57e+04, kdiss: 3.20e−03
1674_ID.3383_Q1_QLLQHLIGL
KD:−
Kass:−, , kdiss: 1.56e+00
1675_ID.3384_D4_SLLDHLIGL
KD: 2.64e−07
Kass: 1.76e+04, kdiss: 4.66e−03
1676_ID.3388_V4_SLLVHLIGL
KD: 5.72e−09
Kass: 2.06e+05, kdiss: 1.18e−03
1677_ID.3389_D8_SLLQHLIDL
KD: 1.21e−07
Kass: 5.90e+04, kdiss: 7.16e−03
1678_ID.339_L8_SLLQHLILL
KD: 5.57e−08
Kass: 6.57e+04, kdiss: 3.66e−03
1679_ID.3390_R4_SLLRHLIGL
KD: 5.20e−09
Kass: 3.06e+05, kdiss: 1.59e−03
1680_ID.3393_K3_SLKQHLIGL
KD: 5.20e−09
Kass: 2.36e+05, kdiss: 1.23e−03
1681_ID.3395_Q6_SLLQHQIGL
KD: 2.39e−09
Kass: 1.90e+05, kdiss: 4.55e−04
1682_ID.3396_T3_SLTQHLIGL
KD: 1.02e−08
Kass: 1.85e+05, kdiss: 1.88e−03
1683_ID.3397_D6_SLLQHDIGL
KD: 1.70e−07
Kass: 4.72e+04, kdiss: 8.03e−03
1684_ID.3399_L8_SLLQHLILL
KD: 5.25e−08
Kass: 7.70e+04, kdiss: 4.05e−03
1685_ID.34_G6_SLLQHGIGL
KD: 5.03e−09
Kass: 1.90e+05, kdiss: 9.54e−04
1686_ID.340_I6_SLLQHIIGL
KD:−
Kass:−, , kdiss: 1.08e+00
1687_ID.3401_W3_SLWQHLIGL
KD: 2.15e−07
Kass: 4.64e+04, kdiss: 9.98e−03
1688_ID.3403_I8_SLLQHLIIL
KD: 1.34e−08
Kass: 1.23e+05, kdiss: 1.65e−03
1689_ID.3404_E4_SLLEHLIGL
KD: 4.57e−08
Kass: 6.33e+04, kdiss: 2.89e−03
1690_ID.3405_F3_SLFQHLIGL
KD: 1.88e−08
Kass: 1.87e+05, kdiss: 3.51e−03
1691_ID.3406_P6_SLLQHPIGL
KD: 7.76e−09
Kass: 3.51e+04, kdiss: 2.73e−04
1692_ID.3407_H3_SLHQHLIGL
KD: 3.37e−08
Kass: 1.31e+05, kdiss: 4.41e−03
1693_ID.3409_P7_SLLQHLPGL
KD:−
Kass:−, , kdiss: 2.92e−02
1694_ID.341_T6_SLLQHTIGL
KD: 1.16e−08
Kass: 1.62e+05, kdiss: 1.88e−03
1695_ID.3410_K4_SLLKHLIGL
KD:−
Kass:−, , kdiss: 1.13e−01
1696_ID.3411_I1_ILLQHLIGL
KD: 5.93e−09
Kass: 1.85e+05, kdiss: 1.10e−03
1697_ID.3412_P4_SLLPHLIGL
KD: 5.54e−09
Kass: 1.80e+05, kdiss: 9.96e−04
1698_ID.3413_E7_SLLQHLEGL
KD: 1.15e−08
Kass: 1.28e+05, kdiss: 1.47e−03
1699_ID.3414_I4_SLLIHLIGL
KD: 7.40e−09
Kass: 1.82e+05, kdiss: 1.35e−03
1700_ID.3417_M1_MLLQHLIGL
KD: 5.90e−09
Kass: 1.80e+05, kdiss: 1.06e−03
value
15000
10000
5000
0
0
250
500
750
1000
Time

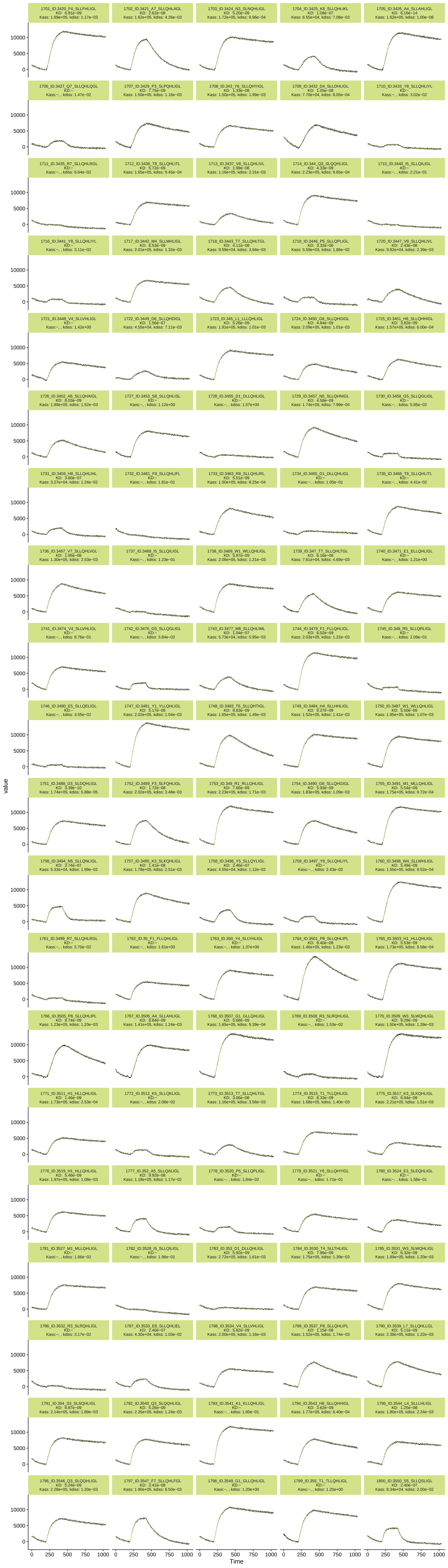

1701_ID.3420_F4_SLLFHLIGL KD: 6.91e−09 Kass: 1.69e+05, kdiss: 1.17e−03
1702_ID.3421_A7_SLLQHLAGL KD: 2.62e−08 Kass: 1.62e+05, kdiss: 4.26e−03
1703_ID.3424_N3_SLNQHLIGL KD: 5.20e−09 Kass: 1.72e+05, kdiss: 8.96e−04
1704_ID.3425_K8_SLLQHLIKL KD: 1.08e−07 Kass: 6.55e+04, kdiss: 7.08e−03
1705_ID.3426_A4_SLLAHLIGL KD: 6.16e−14 Kass: 1.62e+05, kdiss: 1.00e−08
1706_ID.3427_Q7_SLLQHLQGL KD:− Kass:−, , kdiss: 1.47e−02
1707_ID.3429_P3_SLPQHLIGL KD: 7.73e−09 Kass: 1.50e+05, kdiss: 1.16e−03
1708_ID.343_Y6_SLLQHYIGL KD: 1.33e−08 Kass: 1.50e+05, kdiss: 1.99e−03
1709_ID.3432_D4_SLLDHLIGL KD: 1.05e−08 Kass: 7.70e+04, kdiss: 8.05e−04
1710_ID.3433_Y8_SLLQHLIYL KD:− Kass:−, , kdiss: 3.02e−02
1711_ID.3435_R7_SLLQHLRGL KD:− Kass:−, , kdiss: 6.64e−02
1712_ID.3436_T8_SLLQHLITL KD: 5.72e−09 Kass: 1.65e+05, kdiss: 9.45e−04
1713_ID.3437_V8_SLLQHLIVL KD: 1.99e−08 Kass: 1.16e+05, kdiss: 2.31e−03
1714_ID.344_Q3_SLQQHLIGL KD: 4.33e−09 Kass: 2.23e+05, kdiss: 9.65e−04
1715_ID.3440_I5_SLLQIIGL KD:− Kass:−, , kdiss: 2.21e−01
1716_ID.3441_Y8_SLLQHLIYL KD:− Kass:−, , kdiss: 3.11e−02
1717_ID.3442_M4_SLLMHLIGL KD: 6.53e−09 Kass: 2.01e+05, kdiss: 1.32e−03
1718_ID.3443_T7_SLLQHLTGL KD: 4.11e−08 Kass: 9.59e+04, kdiss: 3.94e−03
1719_ID.3446_P5_SLLQPLIGL KD:− Kass: 5.59e+03, kdiss: 1.86e−02
1720_ID.3447_V8_SLLQHLIVL KD: 2.43e−08 Kass: 9.82e+04, kdiss: 2.39e−03
1721_ID.3448_V4_SLLVHLIGL KD:− Kass:−, , kdiss: 1.42e+00
1722_ID.3449_D6_SLLQHDIGL KD: 1.56e−07 Kass: 4.55e+04, kdiss: 7.11e−03
1723_ID.345_L1_LLLQHLIGL KD: 5.26e−09 Kass: 1.91e+05, kdiss: 1.01e−03
1724_ID.3450_G6_SLLQHGIGL KD: 4.84e−09 Kass: 2.09e+05, kdiss: 1.01e−03
1725_ID.3451_H6_SLLQHHIGL KD: 3.82e−09 Kass: 1.57e+05, kdiss: 6.00e−04
1726_ID.3452_A6_SLLQHAIGL KD: 8.03e−09 Kass: 1.89e+05, kdiss: 1.52e−03
1727_ID.3453_S8_SLLQHLISL KD:− Kass:−, , kdiss: 1.12e+00
1728_ID.3455_D1_DLLQHLIGL KD:− Kass:−, , kdiss: 1.57e+00
1729_ID.3457_N6_SLLQHNIGL KD: 4.58e−09 Kass: 1.74e+05, kdiss: 7.99e−04
1730_ID.3458_G5_SLLQGLIGL KD:− Kass:−, , kdiss: 5.85e−02
1731_ID.3459_H8_SLLQHLIHL KD: 3.80e−07 Kass: 3.27e+04, kdiss: 1.24e−02
1732_ID.3461_F8_SLLQHLIFL KD:− Kass:−, , kdiss: 1.81e−01
1733_ID.3463_R8_SLLQHLIRL KD: 5.51e−09 Kass: 1.50e+05, kdiss: 8.25e−04
1734_ID.3465_D1_DLLQHLIGL KD:− Kass:−, , kdiss: 1.05e−01
1735_ID.3466_T8_SLLQHLITL KD:− Kass:−, , kdiss: 4.41e−02
1736_ID.3467_V7_SLLQHLVGL KD: 1.95e−08 Kass: 1.30e+05, kdiss: 2.53e−03
1737_ID.3468_I5_SLLQILIGL KD:− Kass:−, , kdiss: 1.23e−01
1738_ID.3469_W1_WLLQHLIGL KD: 5.87e−09 Kass: 2.06e+05, kdiss: 1.21e−03
1739_ID.347_T7_SLLQHLTGL KD: 6.16e−08 Kass: 7.61e+04, kdiss: 4.69e−03
1740_ID.3471_E1_ELLQHLIGL KD:− Kass:−, , kdiss: 1.21e+00
1741_ID.3474_V4_SLLVHLIGL KD:− Kass:−, , kdiss: 8.76e−01
1742_ID.3476_G5_SLLQGLIGL KD:− Kass:−, , kdiss: 3.84e−02
1743_ID.3477_M8_SLLQHLIML KD: 1.04e−07 Kass: 5.73e+04, kdiss: 5.95e−03
1744_ID.3479_F1_FLLQHLIGL KD: 6.02e−09 Kass: 2.03e+05, kdiss: 1.22e−03
1745_ID.348_R5_SLLQRLIGL KD:− Kass:−, , kdiss: 2.09e−01
1746_ID.3480_E5_SLLQELIGL KD:− Kass:−, , kdiss: 3.55e−02
1747_ID.3481_Y1_YLLQHLIGL KD: 5.17e−09 Kass: 2.02e+05, kdiss: 1.04e−03
1748_ID.3483_T5_SLLQTLIGL KD: 8.83e−09 Kass: 1.65e+05, kdiss: 1.46e−03
1749_ID.3484_H4_SLLHHLIGL KD: 9.27e−09 Kass: 1.52e+05, kdiss: 1.41e−03
1750_ID.3487_W1_WLLQHLIGL KD: 5.50e−09 Kass: 1.95e+05, kdiss: 1.07e−03
1751_ID.3488_D3_SLDQHLIGL KD: 3.39e−10 Kass: 1.74e+05, kdiss: 5.88e−05
1752_ID.3489_F3_SLFQHLIGL KD: 1.72e−08 Kass: 2.02e+05, kdiss: 3.48e−03
1753_ID.349_R1_RLLQHLIGL KD: 7.66e−09 Kass: 2.23e+05, kdiss: 1.71e−03
1754_ID.3490_G6_SLLQHGIGL KD: 5.93e−09 Kass: 1.83e+05, kdiss: 1.09e−03
1755_ID.3491_M1_MLLQHLIGL KD: 5.54e−09 Kass: 1.75e+05, kdiss: 9.72e−04
1756_ID.3494_N5_SLLQNLIGL KD: 3.74e−07 Kass: 5.33e+04, kdiss: 1.99e−02
1757_ID.3495_K3_SLKQHLIGL KD: 1.41e−08 Kass: 1.78e+05, kdiss: 2.51e−03
1758_ID.3496_Y5_SLLQYLIGL KD: 2.46e−07 Kass: 4.55e+04, kdiss: 1.12e−02
1759_ID.3497_Y8_SLLQHLIYL KD:− Kass:−, , kdiss: 2.43e−02
1760_ID.3498_W4_SLLWHLIGL KD: 5.49e−09 Kass: 1.55e+05, kdiss: 8.52e−04
1761_ID.3499_R7_SLLQHLRGL KD:− Kass:−, , kdiss: 5.70e−02
1762_ID.35_F1_FLLQHLIGL KD:− Kass:−, , kdiss: 1.61e+00
1763_ID.350_Y4_SLLYHLIGL KD:− Kass:−, , kdiss: 1.37e+00
1764_ID.3501_P8_SLLQHLIPL KD: 8.40e−09 Kass: 1.46e+05, kdiss: 1.23e−03
1765_ID.3503_H1_HLLQHLIGL KD: 5.53e−09 Kass: 1.73e+05, kdiss: 9.58e−04
1766_ID.3505_P8_SLLQHLIPL KD: 9.74e−09 Kass: 1.23e+05, kdiss: 1.20e−03
1767_ID.3506_A4_SLLAHLIGL KD: 8.84e−09 Kass: 1.41e+05, kdiss: 1.24e−03
1768_ID.3507_G1_GLLQHLIGL KD: 5.68e−09 Kass: 1.65e+05, kdiss: 9.39e−04
1769_ID.3508_R3_SLRQHLIGL KD:− Kass:−, , kdiss: 1.53e−02
1770_ID.3509_W3_SLWQHLIGL KD: 9.29e−09 Kass: 1.50e+05, kdiss: 1.39e−03
1771_ID.3511_H1_HLLQHLIGL KD: 1.46e−09 Kass: 1.73e+05, kdiss: 2.53e−04
1772_ID.3512_K5_SLLQKLIGL KD:− Kass:−, , kdiss: 2.08e−02
1773_ID.3513_T7_SLLQHLTGL KD: 3.06e−08 Kass: 1.16e+05, kdiss: 3.56e−03
1774_ID.3515_T1_TLLQHLIGL KD: 8.33e−09 Kass: 1.68e+05, kdiss: 1.40e−03
1775_ID.3517_K3_SLKQHLIGL KD: 6.84e−09 Kass: 2.21e+05, kdiss: 1.51e−03
1776_ID.3519_H1_HLLQHLIGL KD: 5.46e−09 Kass: 1.97e+05, kdiss: 1.08e−03
1777_ID.352_A5_SLLQALIGL KD: 9.92e−08 Kass: 1.18e+05, kdiss: 1.17e−02
1778_ID.3520_P5_SLLQPLIGL KD:− Kass:−, , kdiss: 1.84e−02
1779_ID.3521_Y6_SLLQHYIGL KD:− Kass:−, , kdiss: 1.71e−01
1780_ID.3524_E3_SLEQHLIGL KD:− Kass:−, , kdiss: 1.58e−01
1781_ID.3527_M1_MLLQHLIGL KD:− Kass:−, , kdiss: 1.66e−02
1782_ID.3528_I5_SLLQILIGL KD:− Kass:−, , kdiss: 1.96e−01
1783_ID.353_D1_DLLQHLIGL KD: 5.92e−09 Kass: 2.72e+05, kdiss: 1.61e−03
1784_ID.3530_T4_SLLTHLIGL KD: 7.96e−09 Kass: 1.75e+05, kdiss: 1.39e−03
1785_ID.3531_W3_SLWQHLIGL KD: 6.32e−09 Kass: 1.89e+05, kdiss: 1.20e−03
1786_ID.3532_R3_SLRQHLIGL KD:− Kass:−, , kdiss: 3.17e−02
1787_ID.3533_E8_SLLQHLIEL KD: 2.40e−07 Kass: 4.30e+04, kdiss: 1.03e−02
1788_ID.3534_V4_SLLVHLIGL KD: 5.82e−09 Kass: 2.00e+05, kdiss: 1.16e−03
1789_ID.3537_P8_SLLQHLIPL KD: 1.15e−08 Kass: 1.52e+05, kdiss: 1.74e−03
1790_ID.3539_L7_SLLQHLLGL KD: 5.11e−09 Kass: 2.38e+05, kdiss: 1.22e−03
1791_ID.354_S3_SLSQHLIGL KD: 8.87e−09 Kass: 2.14e+05, kdiss: 1.89e−03
1792_ID.3540_Q3_SLQQHLIGL KD: 5.26e−09 Kass: 2.35e+05, kdiss: 1.24e−03
1793_ID.3541_K1_KLLQHLIGL KD:− Kass:−, , kdiss: 1.60e−01
1794_ID.3543_H6_SLLQHHIGL KD: 3.62e−09 Kass: 1.77e+05, kdiss: 6.40e−04
1795_ID.3544_L4_SLLLHLIGL KD: 1.25e−08 Kass: 1.80e+05, kdiss: 2.24e−03
1796_ID.3546_Q3_SLQQHLIGL KD: 5.24e−09 Kass: 2.28e+05, kdiss: 1.20e−03
1797_ID.3547_F7_SLLQHLFGL KD: 3.41e−08 Kass: 1.90e+05, kdiss: 6.50e−03
1798_ID.3549_G1_GLLQHLIGL KD:− Kass:−, , kdiss: 1.20e+00
1799_ID.355_T1_TLLQHLIGL KD:− Kass:−, , kdiss: 1.20e+00
1800_ID.3550_S5_SLLQSLIGL KD: 2.40e−07 Kass: 8.34e+04, kdiss: 2.00e−02
value
Time
0
5000
10000
0 250 500 750 1000

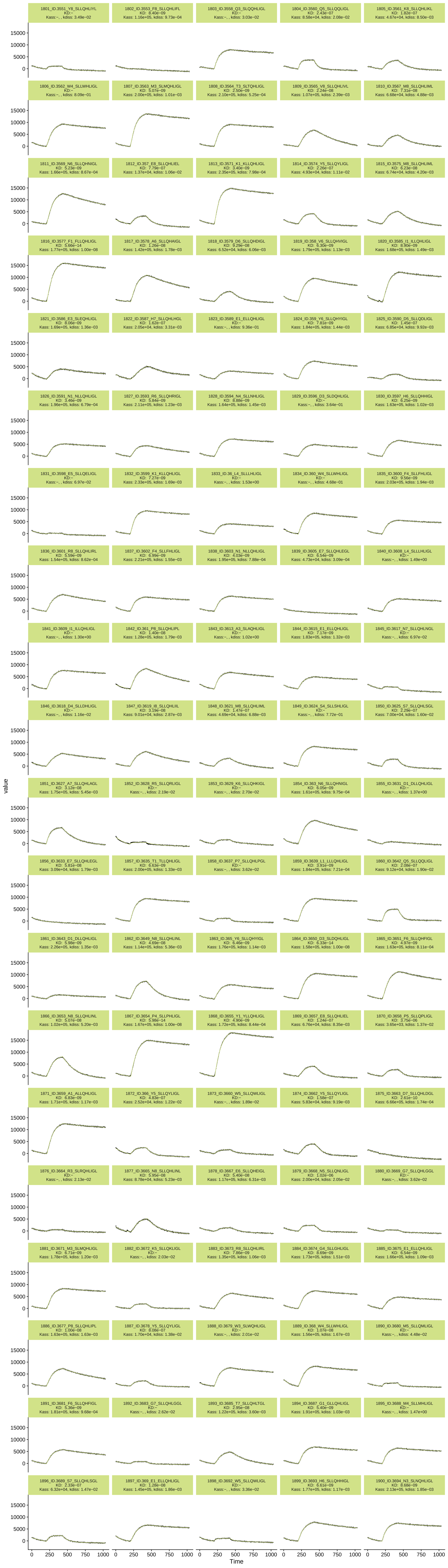
1801_ID.3551_Y8_SLLQHLIYL
KD:−
Kass:−, , kdiss: 3.49e−02
1802_ID.3553_F8_SLLQHLIFL
KD: 8.40e−09
Kass: 1.16e+05, kdiss: 9.73e−04
1803_ID.3558_Q3_SLQQHLIGL
KD:−
Kass:−, , kdiss: 3.03e−02
1804_ID.3560_Q5_SLLQQLIGL
KD: 2.43e−07
Kass: 8.58e+04, kdiss: 2.08e−02
1805_ID.3561_K8_SLLQHLIKL
KD: 1.82e−07
Kass: 4.67e+04, kdiss: 8.50e−03
1806_ID.3562_W4_SLLWHLIGL
KD:−
Kass:−, , kdiss: 8.09e−01
1807_ID.3563_M3_SLMQHLIGL
KD: 5.07e−09
Kass: 2.00e+05, kdiss: 1.01e−03
1808_ID.3564_T3_SLTQHLIGL
KD: 2.50e−09
Kass: 2.10e+05, kdiss: 5.25e−04
1809_ID.3565_V8_SLLQHLIVL
KD: 2.24e−08
Kass: 1.07e+05, kdiss: 2.39e−03
1810_ID.3567_M8_SLLQHLIML
KD: 7.31e−08
Kass: 6.68e+04, kdiss: 4.88e−03
1811_ID.3569_N6_SLLQHNIGL
KD: 5.23e−09
Kass: 1.66e+05, kdiss: 8.67e−04
1812_ID.357_E8_SLLQHLIEL
KD: 7.79e−07
Kass: 1.37e+04, kdiss: 1.06e−02
1813_ID.3571_K1_KLLQHLIGL
KD: 3.40e−09
Kass: 2.35e+05, kdiss: 7.98e−04
1814_ID.3574_Y5_SLLQYLIGL
KD: 2.26e−07
Kass: 4.93e+04, kdiss: 1.11e−02
1815_ID.3575_M8_SLLQHLIML
KD: 6.23e−08
Kass: 6.74e+04, kdiss: 4.20e−03
1816_ID.3577_F1_FLLQHLIGL
KD: 5.66e−14
Kass: 1.77e+05, kdiss: 1.00e−08
1817_ID.3578_A6_SLLQHAIGL
KD: 1.26e−08
Kass: 1.42e+05, kdiss: 1.78e−03
1818_ID.3579_D6_SLLQHDIGL
KD: 9.29e−08
Kass: 6.52e+04, kdiss: 6.06e−03
1819_ID.358_V6_SLLQHVIGL
KD: 6.30e−09
Kass: 1.79e+05, kdiss: 1.13e−03
1820_ID.3585_I1_ILLQHLIGL
KD: 8.90e−09
Kass: 1.68e+05, kdiss: 1.49e−03
1821_ID.3586_E3_SLEQHLIGL
KD: 8.06e−09
Kass: 1.69e+05, kdiss: 1.36e−03
1822_ID.3587_H7_SLLQHLHGL
KD: 1.62e−07
Kass: 2.05e+04, kdiss: 3.31e−03
1823_ID.3589_E1_ELLQHLIGL
KD:−
Kass:−, , kdiss: 9.36e−01
1824_ID.359_Y6_SLLQHYIGL
KD: 7.81e−09
Kass: 1.84e+05, kdiss: 1.44e−03
1825_ID.3590_D5_SLLQDLIGL
KD: 1.45e−07
Kass: 6.85e+04, kdiss: 9.92e−03
1826_ID.3591_N1_NLLQHLIGL
KD: 3.46e−09
Kass: 1.96e+05, kdiss: 6.79e−04
1827_ID.3593_R6_SLLQHRIGL
KD: 5.84e−09
Kass: 2.11e+05, kdiss: 1.23e−03
1828_ID.3594_N4_SLLNHLIGL
KD: 8.88e−09
Kass: 1.64e+05, kdiss: 1.45e−03
1829_ID.3596_D3_SLDQHLIGL
KD:−
Kass:−, , kdiss: 3.64e−01
1830_ID.3597_H6_SLLQHHIGL
KD: 6.25e−09
Kass: 1.63e+05, kdiss: 1.02e−03
1831_ID.3598_E5_SLLQELIGL
KD:−
Kass:−, , kdiss: 6.97e−02
1832_ID.3599_K1_KLLQHLIGL
KD: 7.27e−09
Kass: 2.33e+05, kdiss: 1.69e−03
1833_ID.36_L4_SLLLHLIGL
KD:−
Kass:−, , kdiss: 1.53e+00
1834_ID.360_W4_SLLWHLIGL
KD:−
Kass:−, , kdiss: 4.68e−01
1835_ID.3600_F4_SLLFHLIGL
KD: 9.56e−09
Kass: 2.03e+05, kdiss: 1.94e−03
1836_ID.3601_R8_SLLQHLIRL
KD: 5.59e−09
Kass: 1.54e+05, kdiss: 8.62e−04
1837_ID.3602_F4_SLLFHLIGL
KD: 6.99e−09
Kass: 2.21e+05, kdiss: 1.55e−03
1838_ID.3603_N1_NLLQHLIGL
KD: 4.03e−09
Kass: 1.95e+05, kdiss: 7.88e−04
1839_ID.3605_E7_SLLQHLEGL
KD: 6.54e−09
Kass: 4.73e+04, kdiss: 3.09e−04
1840_ID.3608_L4_SLLLHLIGL
KD:−
Kass:−, , kdiss: 1.49e+00
1841_ID.3609_I1_ILLQHLIGL
KD:−
Kass:−, , kdiss: 1.30e+00
1842_ID.361_P8_SLLQHLIPL
KD: 1.40e−08
Kass: 1.28e+05, kdiss: 1.79e−03
1843_ID.3613_A3_SLAQHLIGL
KD:−
Kass:−, , kdiss: 1.02e+00
1844_ID.3615_E1_ELLQHLIGL
KD: 7.17e−09
Kass: 1.83e+05, kdiss: 1.32e−03
1845_ID.3617_N7_SLLQHLNGL
KD:−
Kass:−, , kdiss: 6.97e−02
1846_ID.3618_D4_SLLDHLIGL
KD:−
Kass:−, , kdiss: 1.16e−02
1847_ID.3619_I8_SLLQHLIIL
KD: 3.19e−08
Kass: 9.01e+04, kdiss: 2.87e−03
1848_ID.3621_M8_SLLQHLIML
KD: 1.47e−07
Kass: 4.69e+04, kdiss: 6.88e−03
1849_ID.3624_S4_SLLSHLIGL
KD:−
Kass:−, , kdiss: 7.72e−01
1850_ID.3625_S7_SLLQHLSGL
KD: 2.29e−07
Kass: 7.00e+04, kdiss: 1.60e−02
1851_ID.3627_A7_SLLQHLAGL
KD: 3.12e−08
Kass: 1.75e+05, kdiss: 5.45e−03
1852_ID.3628_R5_SLLQRLIGL
KD:−
Kass:−, , kdiss: 2.19e−02
1853_ID.3629_K6_SLLQHKIGL
KD:−
Kass:−, , kdiss: 2.70e−02
1854_ID.363_N6_SLLQHNIGL
KD: 6.05e−09
Kass: 1.61e+05, kdiss: 9.75e−04
1855_ID.3631_D1_DLLQHLIGL
KD:−
Kass:−, , kdiss: 1.37e+00
1856_ID.3633_E7_SLLQHLEGL
KD: 5.81e−08
Kass: 3.09e+04, kdiss: 1.79e−03
1857_ID.3635_T1_TLLQHLIGL
KD: 6.63e−09
Kass: 2.00e+05, kdiss: 1.33e−03
1858_ID.3637_P7_SLLQHLPGL
KD:−
Kass:−, , kdiss: 3.62e−02
1859_ID.3639_L1_LLLQHLIGL
KD: 3.91e−09
Kass: 1.84e+05, kdiss: 7.21e−04
1860_ID.3642_Q5_SLLQQLIGL
KD: 2.08e−07
Kass: 9.12e+04, kdiss: 1.90e−02
1861_ID.3643_D1_DLLQHLIGL
KD: 5.98e−09
Kass: 2.26e+05, kdiss: 1.35e−03
1862_ID.3649_N8_SLLQHLINL
KD: 4.69e−08
Kass: 1.14e+05, kdiss: 5.36e−03
1863_ID.365_Y6_SLLQHYIGL
KD: 6.46e−09
Kass: 1.76e+05, kdiss: 1.14e−03
1864_ID.3650_D3_SLDQHLIGL
KD: 6.33e−14
Kass: 1.58e+05, kdiss: 1.00e−08
1865_ID.3651_F6_SLLQHFIGL
KD: 4.97e−09
Kass: 1.63e+05, kdiss: 8.11e−04
1866_ID.3653_N8_SLLQHLINL
KD: 5.07e−08
Kass: 1.02e+05, kdiss: 5.20e−03
1867_ID.3654_P4_SLLPHLIGL
KD: 5.98e−14
Kass: 1.67e+05, kdiss: 1.00e−08
1868_ID.3655_Y1_YLLQHLIGL
KD: 4.90e−09
Kass: 1.72e+05, kdiss: 8.44e−04
1869_ID.3657_E8_SLLQHLIEL
KD: 1.24e−07
Kass: 6.76e+04, kdiss: 8.35e−03
1870_ID.3658_P5_SLLQPLIGL
KD: 3.75e−06
Kass: 3.65e+03, kdiss: 1.37e−02
1871_ID.3659_A1_ALLQHLIGL
KD: 6.83e−09
Kass: 1.71e+05, kdiss: 1.17e−03
1872_ID.366_Y5_SLLQYLIGL
KD: 4.83e−07
Kass: 2.52e+04, kdiss: 1.22e−02
1873_ID.3660_W5_SLLQWLIGL
KD:−
Kass:−, , kdiss: 1.89e−02
1874_ID.3662_Y5_SLLQYLIGL
KD: 1.58e−07
Kass: 5.83e+04, kdiss: 9.19e−03
1875_ID.3663_D7_SLLQHLDGL
KD: 2.61e−10
Kass: 6.66e+05, kdiss: 1.74e−04
1876_ID.3664_R3_SLRQHLIGL
KD:−
Kass:−, , kdiss: 2.13e−02
1877_ID.3665_N8_SLLQHLINL
KD: 5.95e−08
Kass: 8.78e+04, kdiss: 5.23e−03
1878_ID.3667_E6_SLLQHEIGL
KD: 5.40e−08
Kass: 1.17e+05, kdiss: 6.31e−03
1879_ID.3668_N5_SLLQNLIGL
KD: 1.02e−06
Kass: 2.00e+04, kdiss: 2.05e−02
1880_ID.3669_G7_SLLQHLGGL
KD:−
Kass:−, , kdiss: 3.62e−02
1881_ID.3671_M3_SLMQHLIGL
KD: 6.71e−09
Kass: 1.78e+05, kdiss: 1.20e−03
1882_ID.3672_K5_SLLQKLIGL
KD:−
Kass:−, , kdiss: 2.03e−02
1883_ID.3673_R8_SLLQHLIRL
KD: 7.86e−09
Kass: 1.35e+05, kdiss: 1.06e−03
1884_ID.3674_G4_SLLGHLIGL
KD: 8.69e−09
Kass: 1.73e+05, kdiss: 1.51e−03
1885_ID.3675_E1_ELLQHLIGL
KD: 6.54e−09
Kass: 1.66e+05, kdiss: 1.09e−03
1886_ID.3677_P8_SLLQHLIPL
KD: 1.00e−08
Kass: 1.63e+05, kdiss: 1.63e−03
1887_ID.3678_Y5_SLLQYLIGL
KD: 8.08e−07
Kass: 1.70e+04, kdiss: 1.38e−02
1888_ID.3679_W3_SLWQHLIGL
KD:−
Kass:−, , kdiss: 2.01e−02
1889_ID.368_W4_SLLWHLIGL
KD: 1.07e−08
Kass: 1.56e+05, kdiss: 1.67e−03
1890_ID.3680_M5_SLLQMLIGL
KD:−
Kass:−, , kdiss: 4.48e−02
1891_ID.3681_F6_SLLQHFIGL
KD: 5.36e−09
Kass: 1.81e+05, kdiss: 9.68e−04
1892_ID.3683_G7_SLLQHLGGL
KD:−
Kass:−, , kdiss: 2.62e−02
1893_ID.3685_T7_SLLQHLTGL
KD: 2.95e−08
Kass: 1.22e+05, kdiss: 3.60e−03
1894_ID.3687_G1_GLLQHLIGL
KD: 5.40e−09
Kass: 1.91e+05, kdiss: 1.03e−03
1895_ID.3688_M4_SLLMHLIGL
KD:−
Kass:−, , kdiss: 1.47e+00
1896_ID.3689_S7_SLLQHLSGL
KD: 2.33e−07
Kass: 6.32e+04, kdiss: 1.47e−02
1897_ID.369_E1_ELLQHLIGL
KD: 1.28e−08
Kass: 1.45e+05, kdiss: 1.86e−03
1898_ID.3692_W5_SLLQWLIGL
KD:−
Kass:−, , kdiss: 3.36e−02
1899_ID.3693_H6_SLLQHHIGL
KD: 6.61e−09
Kass: 1.77e+05, kdiss: 1.17e−03
1900_ID.3694_N3_SLNQHLIGL
KD: 8.68e−09
Kass: 2.13e+05, kdiss: 1.85e−03
value
15000
10000
5000
0
0
250
500
750
1000
Time

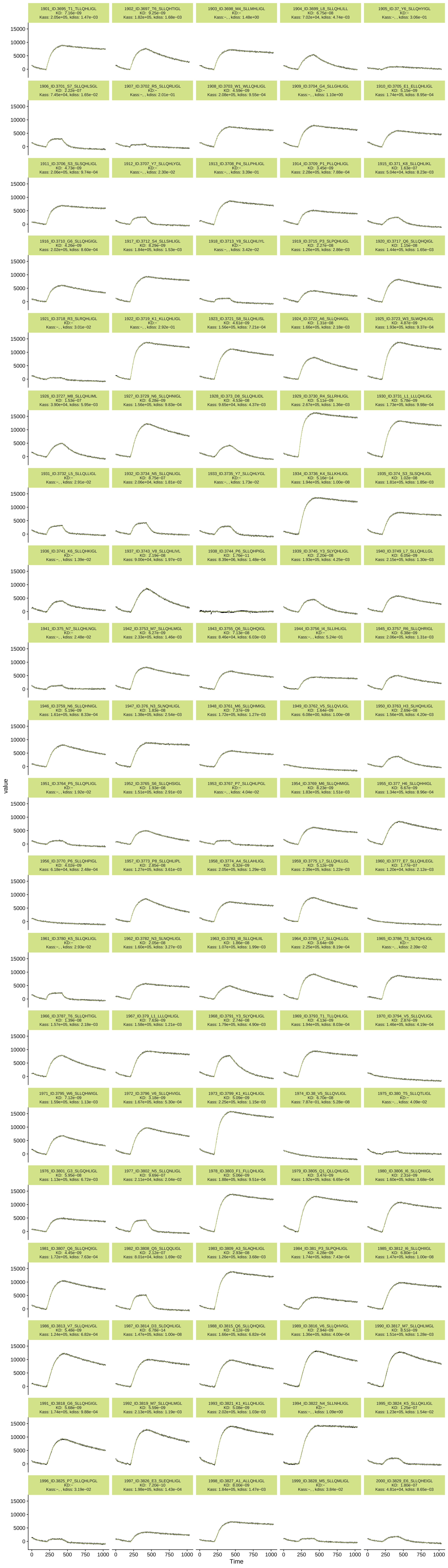

1901_ID.3695_T1_TLLQHLIGL KD: 7.16e−09 Kass: 2.05e+05, kdiss: 1.47e−03
1902_ID.3697_T6_SLLQHTIGL KD: 9.25e−09 Kass: 1.82e+05, kdiss: 1.68e−03
1903_ID.3698_M4_SLLMHLIGL KD:~ Kass:~, , kdiss: 1.48e+00
1904_ID.3699_L8_SLLQHLILL KD: 6.75e−08 Kass: 7.02e+04, kdiss: 4.74e−03
1905_ID.37_Y6_SLLQHYIGL KD:~ Kass:~, , kdiss: 3.06e−01
1906_ID.3701_S7_SLLQHLSGL KD: 2.26e−07 Kass: 7.45e+04, kdiss: 1.65e−02
1907_ID.3702_R5_SLLQRLIGL KD:~ Kass:~, , kdiss: 2.01e−01
1908_ID.3703_W1_WLLQHLIGL KD: 4.59e−09 Kass: 2.08e+05, kdiss: 9.55e−04
1909_ID.3704_G4_SLLGHLIGL KD:~ Kass:~, , kdiss: 1.10e+00
1910_ID.3705_E1_ELLQHLIGL KD: 5.15e−09 Kass: 1.74e+05, kdiss: 8.95e−04
1911_ID.3706_S3_SLSQHLIGL KD: 4.73e−09 Kass: 2.06e+05, kdiss: 9.74e−04
1912_ID.3707_Y7_SLLQHLYGL KD:~ Kass:~, , kdiss: 2.30e−02
1913_ID.3708_P4_SLLPHLIGL KD:~ Kass:~, , kdiss: 3.39e−01
1914_ID.3709_P1_PLLQHLIGL KD: 3.45e−09 Kass: 2.28e+05, kdiss: 7.88e−04
1915_ID.371_K8_SLLQHLIKL KD: 1.63e−07 Kass: 5.04e+04, kdiss: 8.23e−03
1916_ID.3710_G6_SLLQHGIGL KD: 4.26e−09 Kass: 2.02e+05, kdiss: 8.60e−04
1917_ID.3712_S4_SLLSHLIGL KD: 8.29e−09 Kass: 1.84e+05, kdiss: 1.53e−03
1918_ID.3713_Y8_SLLQHLIYL KD:~ Kass:~, , kdiss: 3.42e−02
1919_ID.3715_P3_SLPQHLIGL KD: 2.27e−08 Kass: 1.26e+05, kdiss: 2.86e−03
1920_ID.3717_Q6_SLLQHQIGL KD: 1.15e−08 Kass: 1.44e+05, kdiss: 1.65e−03
1921_ID.3718_R3_SLRQHLIGL KD:~ Kass:~, , kdiss: 3.01e−02
1922_ID.3719_K1_KLLQHLIGL KD:~ Kass:~, , kdiss: 2.92e−01
1923_ID.3721_S8_SLLQHLISL KD: 4.61e−09 Kass: 1.56e+05, kdiss: 7.21e−04
1924_ID.3722_A6_SLLQHAIGL KD: 1.31e−08 Kass: 1.66e+05, kdiss: 2.18e−03
1925_ID.3723_W3_SLWQHLIGL KD: 4.87e−09 Kass: 1.93e+05, kdiss: 9.37e−04
1926_ID.3727_M8_SLLQHLIML KD: 1.53e−07 Kass: 3.90e+04, kdiss: 5.95e−03
1927_ID.3729_N6_SLLQHNIGL KD: 6.28e−09 Kass: 1.56e+05, kdiss: 9.83e−04
1928_ID.373_D8_SLLQHLIDL KD: 4.53e−08 Kass: 9.65e+04, kdiss: 4.37e−03
1929_ID.3730_R4_SLLRHLIGL KD: 5.11e−09 Kass: 2.67e+05, kdiss: 1.36e−03
1930_ID.3731_L1_LLLQHLIGL KD: 5.76e−09 Kass: 1.73e+05, kdiss: 9.98e−04
1931_ID.3732_L5_SLLQLLIGL KD:~ Kass:~, , kdiss: 2.91e−02
1932_ID.3734_N5_SLLQNLIGL KD: 8.75e−07 Kass: 2.06e+04, kdiss: 1.81e−02
1933_ID.3735_Y7_SLLQHLYGL KD:~ Kass:~, , kdiss: 1.73e−02
1934_ID.3736_K4_SLLKHLIGL KD: 5.16e−14 Kass: 1.94e+05, kdiss: 1.00e−08
1935_ID.374_S3_SLSQHLIGL KD: 1.02e−08 Kass: 1.81e+05, kdiss: 1.85e−03
1936_ID.3741_K6_SLLQHKIGL KD:~ Kass:~, , kdiss: 1.39e−02
1937_ID.3743_V8_SLLQHLIVL KD: 2.19e−08 Kass: 9.00e+04, kdiss: 1.97e−03
1938_ID.3744_P6_SLLQHPIGL KD: 1.76e−11 Kass: 8.39e+06, kdiss: 1.48e−04
1939_ID.3745_Y3_SLYQHLIGL KD: 2.20e−08 Kass: 1.93e+05, kdiss: 4.25e−03
1940_ID.3749_L7_SLLQHLLGL KD: 6.05e−09 Kass: 2.15e+05, kdiss: 1.30e−03
1941_ID.375_N7_SLLQHLNGL KD:~ Kass:~, , kdiss: 2.48e−02
1942_ID.3753_M7_SLLQHLMGL KD: 6.27e−09 Kass: 2.33e+05, kdiss: 1.46e−03
1943_ID.3755_Q6_SLLQHQIGL KD: 7.13e−08 Kass: 8.46e+04, kdiss: 6.03e−03
1944_ID.3756_I4_SLLIHLIGL KD:~ Kass:~, , kdiss: 5.24e−01
1945_ID.3757_R6_SLLQHRIGL KD: 6.38e−09 Kass: 2.06e+05, kdiss: 1.31e−03
1946_ID.3759_N6_SLLQHNIGL KD: 5.19e−09 Kass: 1.61e+05, kdiss: 8.33e−04
1947_ID.376_N3_SLNQHLIGL KD: 1.83e−08 Kass: 1.38e+05, kdiss: 2.54e−03
1948_ID.3761_M6_SLLQHMIGL KD: 7.37e−09 Kass: 1.72e+05, kdiss: 1.27e−03
1949_ID.3762_V5_SLLQVLIGL KD: 1.64e−09 Kass: 6.08e+00, kdiss: 1.00e−08
1950_ID.3763_H3_SLHQHLIGL KD: 2.69e−08 Kass: 1.56e+05, kdiss: 4.20e−03
1951_ID.3764_P5_SLLQPLIGL KD:~ Kass:~, , kdiss: 1.92e−02
1952_ID.3765_S6_SLLQHSIGL KD: 1.93e−08 Kass: 1.51e+05, kdiss: 2.91e−03
1953_ID.3767_P7_SLLQHLPGL KD:~ Kass:~, , kdiss: 4.04e−02
1954_ID.3769_M6_SLLQHMIGL KD: 8.23e−09 Kass: 1.83e+05, kdiss: 1.51e−03
1955_ID.377_H6_SLLQHHIGL KD: 6.67e−09 Kass: 1.34e+05, kdiss: 8.96e−04
1956_ID.3770_P6_SLLQHPIGL KD: 4.02e−09 Kass: 6.18e+04, kdiss: 2.48e−04
1957_ID.3773_P8_SLLQHLIPL KD: 2.85e−08 Kass: 1.27e+05, kdiss: 3.61e−03
1958_ID.3774_A4_SLLAHLIGL KD: 6.32e−09 Kass: 2.05e+05, kdiss: 1.29e−03
1959_ID.3775_L7_SLLQHLLGL KD: 5.12e−09 Kass: 2.39e+05, kdiss: 1.22e−03
1960_ID.3777_E7_SLLQHLEGL KD: 1.77e−07 Kass: 1.20e+04, kdiss: 2.12e−03
1961_ID.3780_K5_SLLQKLIGL KD:~ Kass:~, , kdiss: 2.93e−02
1962_ID.3782_N3_SLNQHLIGL KD: 2.05e−08 Kass: 1.60e+05, kdiss: 3.27e−03
1963_ID.3783_I8_SLLQHLIIL KD: 1.86e−08 Kass: 1.07e+05, kdiss: 1.99e−03
1964_ID.3785_L7_SLLQHLLGL KD: 3.64e−09 Kass: 2.25e+05, kdiss: 8.19e−04
1965_ID.3786_T3_SLTQHLIGL KD:~ Kass:~, , kdiss: 2.39e−02
1966_ID.3787_T6_SLLQHTIGL KD: 1.39e−08 Kass: 1.57e+05, kdiss: 2.18e−03
1967_ID.379_L1_LLLQHLIGL KD: 7.63e−09 Kass: 1.58e+05, kdiss: 1.21e−03
1968_ID.3791_Y3_SLYQHLIGL KD: 2.74e−08 Kass: 1.79e+05, kdiss: 4.90e−03
1969_ID.3793_T1_TLLQHLIGL KD: 4.13e−09 Kass: 1.94e+05, kdiss: 8.03e−04
1970_ID.3794_V5_SLLQVLIGL KD: 2.87e−09 Kass: 1.46e+05, kdiss: 4.19e−04
1971_ID.3795_W6_SLLQHWIGL KD: 7.12e−09 Kass: 1.59e+05, kdiss: 1.13e−03
1972_ID.3796_V6_SLLQHVIGL KD: 3.18e−09 Kass: 1.67e+05, kdiss: 5.30e−04
1973_ID.3799_K1_KLLQHLIGL KD: 5.09e−09 Kass: 2.25e+05, kdiss: 1.15e−03
1974_ID.38_V5_SLLQVLIGL KD: 6.70e−09 Kass: 7.87e−01, kdiss: 5.28e−08
1975_ID.380_T5_SLLQTLIGL KD:~ Kass:~, , kdiss: 4.09e−02
1976_ID.3801_G3_SLGQHLIGL KD: 5.95e−08 Kass: 1.13e+05, kdiss: 6.72e−03
1977_ID.3802_N5_SLLQNLIGL KD: 9.69e−07 Kass: 2.11e+04, kdiss: 2.04e−02
1978_ID.3803_F1_FLLQHLIGL KD: 5.06e−09 Kass: 1.88e+05, kdiss: 9.51e−04
1979_ID.3805_Q1_QLLQHLIGL KD: 3.47e−09 Kass: 1.92e+05, kdiss: 6.65e−04
1980_ID.3806_I6_SLLQHIIGL KD: 2.31e−09 Kass: 1.60e+05, kdiss: 3.68e−04
1981_ID.3807_Q6_SLLQHQIGL KD: 4.45e−09 Kass: 1.72e+05, kdiss: 7.63e−04
1982_ID.3808_Q5_SLLQQLIGL KD: 2.12e−07 Kass: 8.01e+04, kdiss: 1.69e−02
1983_ID.3809_A3_SLAQHLIGL KD: 2.93e−08 Kass: 1.26e+05, kdiss: 3.68e−03
1984_ID.381_P3_SLPQHLIGL KD: 4.28e−09 Kass: 1.74e+05, kdiss: 7.43e−04
1985_ID.3812_I6_SLLQHIIGL KD: 6.80e−14 Kass: 1.47e+05, kdiss: 1.00e−08
1986_ID.3813_V7_SLLQHLVGL KD: 5.48e−09 Kass: 1.24e+05, kdiss: 6.82e−04
1987_ID.3814_D3_SLDQHLIGL KD: 6.78e−14 Kass: 1.47e+05, kdiss: 1.00e−08
1988_ID.3815_Q6_SLLQHQIGL KD: 4.12e−09 Kass: 1.66e+05, kdiss: 6.82e−04
1989_ID.3816_V6_SLLQHVIGL KD: 2.94e−09 Kass: 1.36e+05, kdiss: 4.00e−04
1990_ID.3817_M7_SLLQHLMGL KD: 8.51e−09 Kass: 1.51e+05, kdiss: 1.28e−03
1991_ID.3818_G6_SLLQHGIGL KD: 5.68e−09 Kass: 1.74e+05, kdiss: 9.88e−04
1992_ID.3819_M7_SLLQHLMGL KD: 5.59e−09 Kass: 2.13e+05, kdiss: 1.19e−03
1993_ID.3821_K1_KLLQHLIGL KD: 5.08e−09 Kass: 2.02e+05, kdiss: 1.03e−03
1994_ID.3822_N4_SLLNHLIGL KD:~ Kass:~, , kdiss: 1.09e+00
1995_ID.3824_K5_SLLQKLIGL KD: 1.25e−07 Kass: 1.23e+05, kdiss: 1.54e−02
1996_ID.3825_P7_SLLQHLPGL KD:~ Kass:~, , kdiss: 3.19e−02
1997_ID.3826_E3_SLEQHLIGL KD: 7.20e−10 Kass: 1.98e+05, kdiss: 1.43e−04
1998_ID.3827_A1_ALLQHLIGL KD: 8.00e−09 Kass: 1.84e+05, kdiss: 1.47e−03
1999_ID.3828_M5_SLLQMLIGL KD:~ Kass:~, , kdiss: 3.84e−02
2000_ID.3829_E6_SLLQHEIGL KD: 1.80e−07 Kass: 4.81e+04, kdiss: 8.65e−03
value
Time
0 250 500 750 1000
0 5000 10000 15000

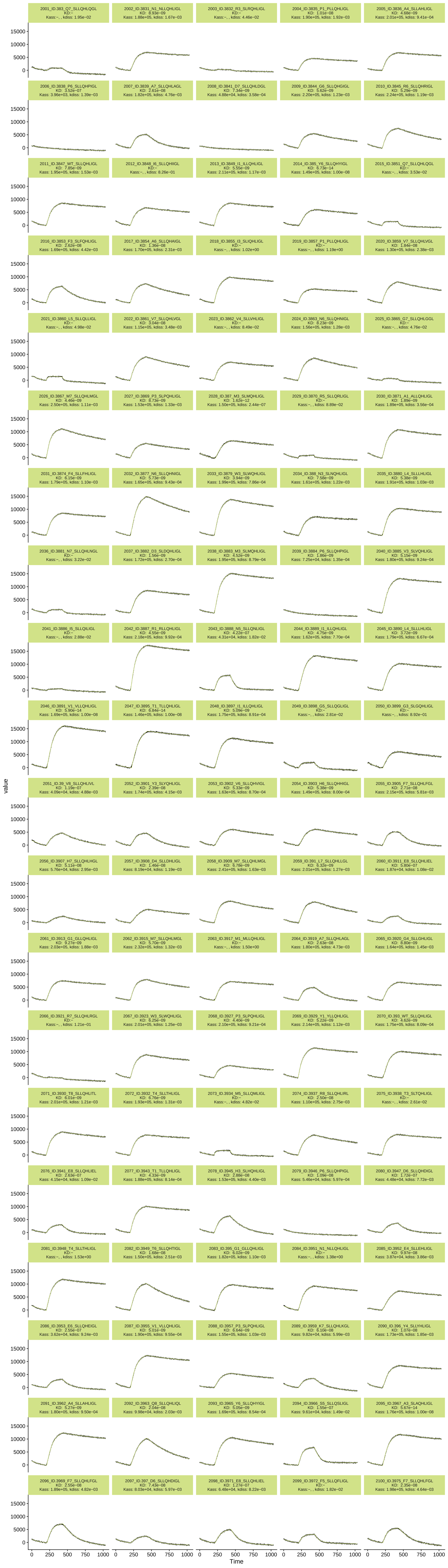
2001_ID.383_Q7_SLLQHLQGL
KD:−
Kass:−, , kdiss: 1.95e−02
2002_ID.3831_N1_NLLQHLIGL
KD: 8.93e−09
Kass: 1.88e+05, kdiss: 1.67e−03
2003_ID.3832_R3_SLRQHLIGL
KD:−
Kass:−, , kdiss: 4.46e−02
2004_ID.3835_P1_PLLQHLIGL
KD: 1.01e−08
Kass: 1.90e+05, kdiss: 1.92e−03
2005_ID.3836_A4_SLLAHLIGL
KD: 4.68e−09
Kass: 2.01e+05, kdiss: 9.41e−04
2006_ID.3838_P6_SLLQHPIGL
KD: 3.52e−07
Kass: 3.96e+03, kdiss: 1.39e−03
2007_ID.3839_A7_SLLQHLAGL
KD: 2.61e−08
Kass: 1.82e+05, kdiss: 4.76e−03
2008_ID.3841_D7_SLLQHLDGL
KD: 7.34e−09
Kass: 4.88e+04, kdiss: 3.58e−04
2009_ID.3844_G6_SLLQHGIGL
KD: 5.62e−09
Kass: 2.20e+05, kdiss: 1.23e−03
2010_ID.3845_R6_SLLQHRIGL
KD: 5.29e−09
Kass: 2.24e+05, kdiss: 1.19e−03
2011_ID.3847_WT_SLLQHLIGL
KD: 7.85e−09
Kass: 1.95e+05, kdiss: 1.53e−03
2012_ID.3848_I6_SLLQHIIGL
KD:−
Kass:−, , kdiss: 8.26e−01
2013_ID.3849_I1_ILLQHLIGL
KD: 5.55e−09
Kass: 2.11e+05, kdiss: 1.17e−03
2014_ID.385_Y6_SLLQHYIGL
KD: 6.73e−14
Kass: 1.49e+05, kdiss: 1.00e−08
2015_ID.3851_Q7_SLLQHLQGL
KD:−
Kass:−, , kdiss: 3.53e−02
2016_ID.3853_F3_SLFQHLIGL
KD: 2.62e−08
Kass: 1.69e+05, kdiss: 4.42e−03
2017_ID.3854_A6_SLLQHAIGL
KD: 1.36e−08
Kass: 1.70e+05, kdiss: 2.31e−03
2018_ID.3855_I3_SLIQHLIGL
KD:−
Kass:−, , kdiss: 1.02e+00
2019_ID.3857_P1_PLLQHLIGL
KD:−
Kass:−, , kdiss: 1.19e+00
2020_ID.3859_V7_SLLQHLVGL
KD: 1.84e−08
Kass: 1.30e+05, kdiss: 2.38e−03
2021_ID.3860_L5_SLLQLLIGL
KD:−
Kass:−, , kdiss: 4.98e−02
2022_ID.3861_V7_SLLQHLVGL
KD: 3.04e−08
Kass: 1.15e+05, kdiss: 3.48e−03
2023_ID.3862_V4_SLLVHLIGL
KD:−
Kass:−, , kdiss: 8.49e−02
2024_ID.3863_N6_SLLQHNIGL
KD: 8.23e−09
Kass: 1.56e+05, kdiss: 1.28e−03
2025_ID.3865_G7_SLLQHLGGL
KD:−
Kass:−, , kdiss: 4.76e−02
2026_ID.3867_M7_SLLQHLMGL
KD: 4.46e−09
Kass: 2.50e+05, kdiss: 1.11e−03
2027_ID.3869_P3_SLPQHLIGL
KD: 8.73e−09
Kass: 1.53e+05, kdiss: 1.33e−03
2028_ID.387_M3_SLMQHLIGL
KD: 1.62e−12
Kass: 1.50e+05, kdiss: 2.44e−07
2029_ID.3870_R5_SLLQRLIGL
KD:−
Kass:−, , kdiss: 8.89e−02
2030_ID.3871_A1_ALLQHLIGL
KD: 1.89e−09
Kass: 1.89e+05, kdiss: 3.56e−04
2031_ID.3874_F4_SLLFHLIGL
KD: 6.15e−09
Kass: 1.79e+05, kdiss: 1.10e−03
2032_ID.3877_N6_SLLQHNIGL
KD: 5.73e−09
Kass: 1.65e+05, kdiss: 9.43e−04
2033_ID.3879_W3_SLWQHLIGL
KD: 3.94e−09
Kass: 1.99e+05, kdiss: 7.86e−04
2034_ID.388_N3_SLNQHLIGL
KD: 7.58e−09
Kass: 1.61e+05, kdiss: 1.22e−03
2035_ID.3880_L4_SLLLHLIGL
KD: 5.38e−09
Kass: 1.91e+05, kdiss: 1.03e−03
2036_ID.3881_N7_SLLQHLNGL
KD:−
Kass:−, , kdiss: 3.22e−02
2037_ID.3882_D3_SLDQHLIGL
KD: 1.56e−09
Kass: 1.72e+05, kdiss: 2.70e−04
2038_ID.3883_M3_SLMQHLIGL
KD: 4.52e−09
Kass: 1.95e+05, kdiss: 8.79e−04
2039_ID.3884_P6_SLLQHPIGL
KD: 1.86e−09
Kass: 7.25e+04, kdiss: 1.35e−04
2040_ID.3885_V3_SLVQHLIGL
KD: 5.15e−09
Kass: 1.80e+05, kdiss: 9.24e−04
2041_ID.3886_I5_SLLQILIGL
KD:−
Kass:−, , kdiss: 2.88e−02
2042_ID.3887_R1_RLLQHLIGL
KD: 4.55e−09
Kass: 2.18e+05, kdiss: 9.92e−04
2043_ID.3888_N5_SLLQNLIGL
KD: 4.22e−07
Kass: 4.31e+04, kdiss: 1.82e−02
2044_ID.3889_I1_ILLQHLIGL
KD: 4.75e−09
Kass: 1.62e+05, kdiss: 7.70e−04
2045_ID.3890_L4_SLLLHLIGL
KD: 3.72e−09
Kass: 1.79e+05, kdiss: 6.67e−04
2046_ID.3891_V1_VLLQHLIGL
KD: 5.90e−14
Kass: 1.69e+05, kdiss: 1.00e−08
2047_ID.3895_T1_TLLQHLIGL
KD: 6.84e−14
Kass: 1.46e+05, kdiss: 1.00e−08
2048_ID.3897_I1_ILLQHLIGL
KD: 5.09e−09
Kass: 1.75e+05, kdiss: 8.91e−04
2049_ID.3898_G5_SLLQGLIGL
KD:−
Kass:−, , kdiss: 2.81e−02
2050_ID.3899_G3_SLGQHLIGL
KD:−
Kass:−, , kdiss: 8.92e−01
2051_ID.39_V8_SLLQHLIVL
KD: 1.19e−07
Kass: 4.09e+04, kdiss: 4.88e−03
2052_ID.3901_Y3_SLYQHLIGL
KD: 2.39e−08
Kass: 1.74e+05, kdiss: 4.15e−03
2053_ID.3902_V6_SLLQHVIGL
KD: 5.33e−09
Kass: 1.63e+05, kdiss: 8.70e−04
2054_ID.3903_H6_SLLQHHIGL
KD: 5.38e−09
Kass: 1.49e+05, kdiss: 8.00e−04
2055_ID.3905_F7_SLLQHLFGL
KD: 2.71e−08
Kass: 2.15e+05, kdiss: 5.81e−03
2056_ID.3907_H7_SLLQHLHGL
KD: 5.11e−08
Kass: 5.76e+04, kdiss: 2.95e−03
2057_ID.3908_D4_SLLDHLIGL
KD: 1.46e−08
Kass: 8.19e+04, kdiss: 1.19e−03
2058_ID.3909_M7_SLLQHLMGL
KD: 6.78e−09
Kass: 2.41e+05, kdiss: 1.63e−03
2059_ID.391_L7_SLLQHLLGL
KD: 6.32e−09
Kass: 2.01e+05, kdiss: 1.27e−03
2060_ID.3911_E8_SLLQHLIEL
KD: 5.80e−07
Kass: 1.87e+04, kdiss: 1.08e−02
2061_ID.3913_G1_GLLQHLIGL
KD: 9.27e−09
Kass: 2.03e+05, kdiss: 1.88e−03
2062_ID.3915_M7_SLLQHLMGL
KD: 5.70e−09
Kass: 2.32e+05, kdiss: 1.32e−03
2063_ID.3917_M1_MLLQHLIGL
KD:−
Kass:−, , kdiss: 1.50e+00
2064_ID.3919_A7_SLLQHLAGL
KD: 2.63e−08
Kass: 1.80e+05, kdiss: 4.73e−03
2065_ID.3920_G4_SLLGHLIGL
KD: 8.80e−09
Kass: 1.64e+05, kdiss: 1.45e−03
2066_ID.3921_R7_SLLQHLRGL
KD:−
Kass:−, , kdiss: 1.21e−01
2067_ID.3923_W3_SLWQHLIGL
KD: 6.25e−09
Kass: 2.01e+05, kdiss: 1.25e−03
2068_ID.3927_P3_SLPQHLIGL
KD: 4.40e−09
Kass: 2.10e+05, kdiss: 9.21e−04
2069_ID.3929_Y1_YLLQHLIGL
KD: 5.22e−09
Kass: 2.14e+05, kdiss: 1.12e−03
2070_ID.393_WT_SLLQHLIGL
KD: 4.62e−09
Kass: 1.75e+05, kdiss: 8.09e−04
2071_ID.3930_T8_SLLQHLITL
KD: 6.01e−09
Kass: 2.01e+05, kdiss: 1.21e−03
2072_ID.3932_T4_SLLTHLIGL
KD: 6.76e−09
Kass: 1.93e+05, kdiss: 1.31e−03
2073_ID.3934_M5_SLLQMLIGL
KD:−
Kass:−, , kdiss: 4.82e−02
2074_ID.3937_R8_SLLQHLIRL
KD: 2.50e−08
Kass: 1.10e+05, kdiss: 2.75e−03
2075_ID.3938_T3_SLTQHLIGL
KD:−
Kass:−, , kdiss: 2.61e−02
2076_ID.3941_E8_SLLQHLIEL
KD: 2.63e−07
Kass: 4.15e+04, kdiss: 1.09e−02
2077_ID.3943_T1_TLLQHLIGL
KD: 4.33e−09
Kass: 1.88e+05, kdiss: 8.14e−04
2078_ID.3945_H3_SLHQHLIGL
KD: 2.88e−08
Kass: 1.53e+05, kdiss: 4.40e−03
2079_ID.3946_P6_SLLQHPIGL
KD: 1.09e−08
Kass: 5.46e+04, kdiss: 5.97e−04
2080_ID.3947_D6_SLLQHDIGL
KD: 1.72e−07
Kass: 4.48e+04, kdiss: 7.72e−03
2081_ID.3948_T4_SLLTHLIGL
KD:−
Kass:−, , kdiss: 1.53e+00
2082_ID.3949_T6_SLLQHTIGL
KD: 1.68e−08
Kass: 1.50e+05, kdiss: 2.51e−03
2083_ID.395_G1_GLLQHLIGL
KD: 6.02e−09
Kass: 1.82e+05, kdiss: 1.10e−03
2084_ID.3951_N1_NLLQHLIGL
KD:−
Kass:−, , kdiss: 1.38e+00
2085_ID.3952_E4_SLLEHLIGL
KD: 9.97e−08
Kass: 3.87e+04, kdiss: 3.86e−03
2086_ID.3953_E6_SLLQHEIGL
KD: 2.55e−07
Kass: 3.62e+04, kdiss: 9.24e−03
2087_ID.3955_V1_VLLQHLIGL
KD: 5.01e−09
Kass: 1.90e+05, kdiss: 9.55e−04
2088_ID.3957_P3_SLPQHLIGL
KD: 6.64e−09
Kass: 1.55e+05, kdiss: 1.03e−03
2089_ID.3959_K7_SLLQHLKGL
KD: 6.10e−08
Kass: 9.82e+04, kdiss: 5.99e−03
2090_ID.396_Y4_SLLYHLIGL
KD: 1.07e−08
Kass: 1.73e+05, kdiss: 1.85e−03
2091_ID.3962_A4_SLLAHLIGL
KD: 5.27e−09
Kass: 1.80e+05, kdiss: 9.50e−04
2092_ID.3963_Q8_SLLQHLIQL
KD: 2.04e−08
Kass: 9.98e+04, kdiss: 2.03e−03
2093_ID.3965_Y6_SLLQHYIGL
KD: 5.05e−09
Kass: 1.69e+05, kdiss: 8.54e−04
2094_ID.3966_S5_SLLQSLIGL
KD: 1.55e−07
Kass: 9.61e+04, kdiss: 1.49e−02
2095_ID.3967_A3_SLAQHLIGL
KD: 5.67e−14
Kass: 1.76e+05, kdiss: 1.00e−08
2096_ID.3969_F7_SLLQHLFGL
KD: 2.55e−08
Kass: 1.89e+05, kdiss: 4.82e−03
2097_ID.397_D6_SLLQHDIGL
KD: 7.43e−08
Kass: 8.03e+04, kdiss: 5.97e−03
2098_ID.3971_E8_SLLQHLIEL
KD: 1.27e−07
Kass: 6.48e+04, kdiss: 8.22e−03
2099_ID.3972_F5_SLLQFLIGL
KD:−
Kass:−, , kdiss: 1.82e−02
2100_ID.3975_F7_SLLQHLFGL
KD: 2.35e−08
Kass: 1.98e+05, kdiss: 4.64e−03
value
15000
10000
5000
0
0
250
500
750
1000
Time

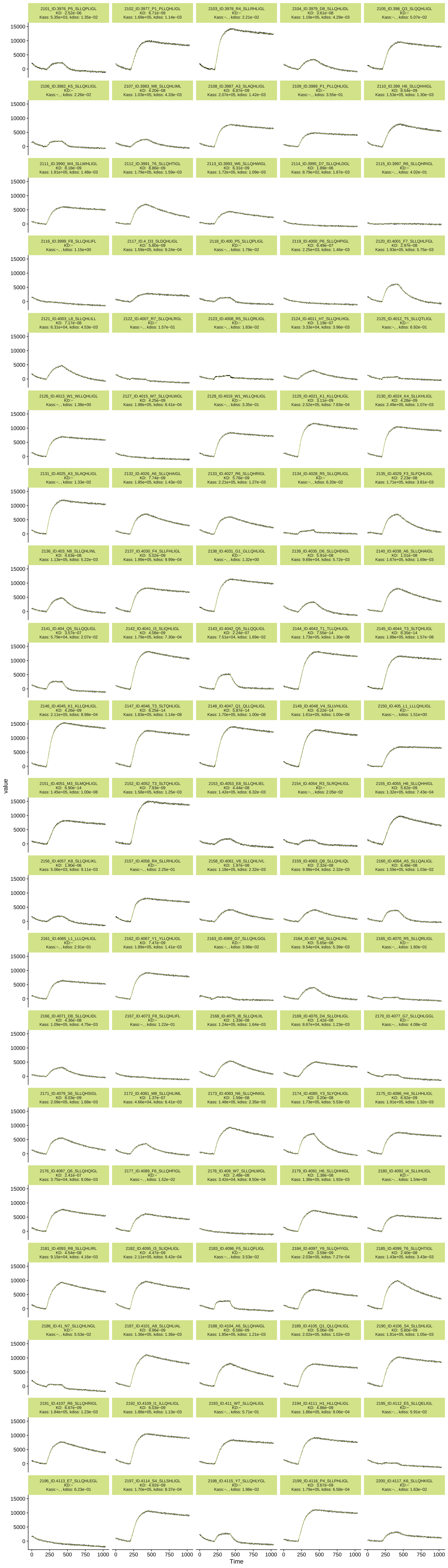

value
Time

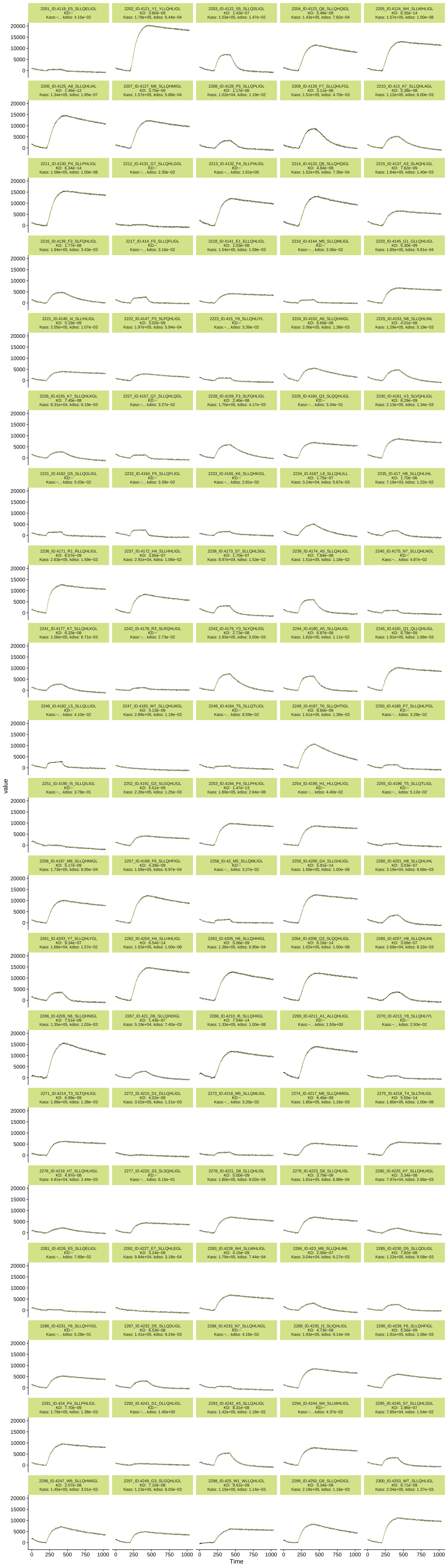

2201_ID.4118_E5_SLLQELIGL
KD: -
Kass:-, , kdiss: 4.15e-02
2202_ID.4121_Y1_YLLQHLIGL
KD: 3.60e-09
Kass: 1.79e+05, kdiss: 6.44e-04
2203_ID.4122_S5_SLLQSLIGL
KD: 1.43e-07
Kass: 1.03e+05, kdiss: 1.47e-02
2204_ID.4123_Q6_SLLQHQIGL
KD: 5.48e-09
Kass: 1.43e+05, kdiss: 7.82e-04
2205_ID.4124_W4_SLLWHLIGL
KD: 6.35e-14
Kass: 1.57e+05, kdiss: 1.00e-08
2206_ID.4125_A8_SLLQHLIAL
KD: 1.46e-12
Kass: 1.34e+05, kdiss: 1.95e-07
2207_ID.4126_M6_SLLQHMIGL
KD: 3.75e-09
Kass: 1.57e+05, kdiss: 5.88e-04
2208_ID.4128_P5_SLLQPLIGL
KD: 1.17e-06
Kass: 1.02e+04, kdiss: 1.19e-02
2209_ID.4129_F7_SLLQHLFGL
KD: 3.11e-08
Kass: 1.51e+05, kdiss: 4.70e-03
2210_ID.413_A7_SLLQHLAGL
KD: 5.36e-08
Kass: 1.12e+05, kdiss: 6.00e-03
2211_ID.4130_P4_SLLPHLIGL
KD: 6.34e-14
Kass: 1.58e+05, kdiss: 1.00e-08
2212_ID.4131_G7_SLLQHLGGL
KD: -
Kass:-, , kdiss: 2.30e-02
2213_ID.4132_F4_SLLFHLIGL
KD: -
Kass:-, , kdiss: 1.61e+00
2214_ID.4133_Q6_SLLQHQIGL
KD: 4.84e-09
Kass: 1.52e+05, kdiss: 7.36e-04
2215_ID.4137_A3_SLAQHLIGL
KD: 7.62e-09
Kass: 1.84e+05, kdiss: 1.40e-03
2216_ID.4139_F3_SLFQHLIGL
KD: 1.77e-08
Kass: 1.94e+05, kdiss: 3.43e-03
2217_ID.414_F5_SLLQFLIGL
KD: -
Kass:-, , kdiss: 3.16e-02
2218_ID.4141_E1_ELLQHLIGL
KD: 1.03e-08
Kass: 1.54e+05, kdiss: 1.59e-03
2219_ID.4144_M5_SLLQMLIGL
KD: -
Kass:-, , kdiss: 3.36e-02
2220_ID.4145_G1_GLLQHLIGL
KD: 5.30e-09
Kass: 1.85e+05, kdiss: 9.81e-04
2221_ID.4146_I4_SLLIHLIGL
KD: 5.19e-09
Kass: 2.05e+05, kdiss: 1.07e-03
2222_ID.4147_P3_SLPQHLIGL
KD: 3.02e-08
Kass: 1.97e+05, kdiss: 5.94e-03
2223_ID.415_Y8_SLLQHLIYL
KD: -
Kass:-, , kdiss: 3.39e-02
2224_ID.4152_A6_SLLQHAIGL
KD: 6.69e-09
Kass: 2.06e+05, kdiss: 1.38e-03
2225_ID.4153_N8_SLLQHLINL
KD: 4.01e-08
Kass: 1.29e+05, kdiss: 5.19e-03
2226_ID.4155_K7_SLLQHLKGL
KD: 7.45e-08
Kass: 8.31e+04, kdiss: 6.19e-03
2227_ID.4157_Q7_SLLQHLQGL
KD: -
Kass:-, , kdiss: 3.27e-02
2228_ID.4159_F3_SLFQHLIGL
KD: 2.46e-08
Kass: 1.70e+05, kdiss: 4.17e-03
2229_ID.4160_Q3_SLQQHLIGL
KD: -
Kass:-, , kdiss: 3.34e-01
2230_ID.4161_V3_SLVQHLIGL
KD: 6.29e-09
Kass: 2.13e+05, kdiss: 1.34e-03
2231_ID.4162_G5_SLLQGLIGL
KD: -
Kass:-, , kdiss: 5.03e-02
2232_ID.4164_F5_SLLQFLIGL
KD: -
Kass:-, , kdiss: 3.39e-02
2233_ID.4165_K6_SLLQHKIGL
KD: -
Kass:-, , kdiss: 2.81e-02
2234_ID.4167_L8_SLLQHLILL
KD: 1.75e-07
Kass: 3.24e+04, kdiss: 5.67e-03
2235_ID.417_H8_SLLQHLIHL
KD: 1.70e-06
Kass: 7.16e+03, kdiss: 1.22e-02
2236_ID.4171_R1_RLLQHLIGL
KD: 6.07e-09
Kass: 2.63e+05, kdiss: 1.59e-03
2237_ID.4172_H4_SLLHHLIGL
KD: 3.65e-07
Kass: 2.91e+04, kdiss: 1.06e-02
2238_ID.4173_S7_SLLQHLSGL
KD: 1.70e-07
Kass: 8.97e+04, kdiss: 1.53e-02
2239_ID.4174_A5_SLLQALIGL
KD: 7.84e-08
Kass: 1.51e+05, kdiss: 1.18e-02
2240_ID.4175_N7_SLLQHLNGL
KD: -
Kass:-, , kdiss: 4.87e-02
2241_ID.4177_K7_SLLQHLKGL
KD: 6.33e-08
Kass: 1.06e+05, kdiss: 6.71e-03
2242_ID.4178_R3_SLRQHLIGL
KD: -
Kass:-, , kdiss: 2.73e-02
2243_ID.4179_Y3_SLYQHLIGL
KD: 2.73e-08
Kass: 1.83e+05, kdiss: 5.00e-03
2244_ID.4180_A5_SLLQALIGL
KD: 6.87e-08
Kass: 1.62e+05, kdiss: 1.11e-02
2245_ID.4181_Q1_QLLQHLIGL
KD: 8.78e-09
Kass: 1.91e+05, kdiss: 1.68e-03
2246_ID.4182_L5_SLLQLLIGL
KD: -
Kass:-, , kdiss: 4.10e-02
2247_ID.4183_W7_SLLQHLWGL
KD: 4.13e-09
Kass: 2.89e+05, kdiss: 1.19e-03
2248_ID.4184_T5_SLLQTLIGL
KD: -
Kass:-, , kdiss: 8.59e-02
2249_ID.4187_T6_SLLQHTIGL
KD: 8.60e-09
Kass: 1.61e+05, kdiss: 1.39e-03
2250_ID.4189_P7_SLLQHLPGL
KD: -
Kass:-, , kdiss: 3.28e-02
2251_ID.4190_I5_SLLQILIGL
KD: -
Kass:-, , kdiss: 3.78e-01
2252_ID.4191_G3_SLGQHLIGL
KD: 5.51e-09
Kass: 2.26e+05, kdiss: 1.25e-03
2253_ID.4194_P4_SLLPHLIGL
KD: 1.47e-13
Kass: 1.80e+05, kdiss: 2.64e-08
2254_ID.4195_H1_HLLQHLIGL
KD: -
Kass:-, , kdiss: 4.40e-02
2255_ID.4196_T5_SLLQTLIGL
KD: -
Kass:-, , kdiss: 5.12e-02
2256_ID.4197_M6_SLLQHMIGL
KD: 5.17e-09
Kass: 1.73e+05, kdiss: 8.95e-04
2257_ID.4199_F6_SLLQHFIGL
KD: 4.39e-09
Kass: 1.59e+05, kdiss: 6.97e-04
2258_ID.42_M5_SLLQMLIGL
KD: -
Kass:-, , kdiss: 3.27e-02
2259_ID.4200_G4_SLLGHLIGL
KD: 5.91e-14
Kass: 1.69e+05, kdiss: 1.00e-08
2260_ID.4201_H8_SLLQHLIHL
KD: 3.03e-07
Kass: 3.19e+04, kdiss: 9.68e-03
2261_ID.4203_Y7_SLLQHLYGL
KD: 9.34e-08
Kass: 1.68e+05, kdiss: 1.57e-02
2262_ID.4204_H4_SLLHHLIGL
KD: 6.54e-14
Kass: 1.53e+05, kdiss: 1.00e-08
2263_ID.4205_H6_SLLQHHIGL
KD: 5.07e-09
Kass: 1.36e+05, kdiss: 6.90e-04
2264_ID.4206_Q3_SLQQHLIGL
KD: 6.16e-14
Kass: 1.62e+05, kdiss: 1.00e-08
2265_ID.4207_H8_SLLQHLIHL
KD: 3.09e-07
Kass: 2.69e+04, kdiss: 8.32e-03
2266_ID.4209_N6_SLLQHNIGL
KD: 7.51e-09
Kass: 1.35e+05, kdiss: 1.02e-03
2267_ID.421_D6_SLLQHDIGL
KD: 1.43e-07
Kass: 5.19e+04, kdiss: 7.40e-03
2268_ID.4210_I6_SLLQHIIGL
KD: 7.54e-14
Kass: 1.33e+05, kdiss: 1.00e-08
2269_ID.4211_A1_ALLQHLIGL
KD: -
Kass:-, , kdiss: 1.50e+00
2270_ID.4213_Y8_SLLQHLIYL
KD: -
Kass:-, , kdiss: 2.50e-02
2271_ID.4214_T3_SLTQHLIGL
KD: 6.99e-09
Kass: 1.98e+05, kdiss: 1.38e-03
2272_ID.4215_D1_DLLQHLIGL
KD: 4.02e-09
Kass: 3.02e+05, kdiss: 1.21e-03
2273_ID.4216_M5_SLLQMLIGL
KD: -
Kass:-, , kdiss: 3.20e-02
2274_ID.4217_M6_SLLQHMIGL
KD: 6.46e-09
Kass: 1.80e+05, kdiss: 1.16e-03
2275_ID.4218_T4_SLLTHLIGL
KD: 5.55e-14
Kass: 1.80e+05, kdiss: 1.00e-08
2276_ID.4219_H7_SLLQHLHGL
KD: 4.97e-08
Kass: 4.91e+04, kdiss: 2.44e-03
2277_ID.4220_S3_SLSQHLIGL
KD: -
Kass:-, , kdiss: 6.15e-01
2278_ID.4221_S8_SLLQHLISL
KD: 5.00e-09
Kass: 1.80e+05, kdiss: 9.02e-04
2279_ID.4223_S8_SLLQHLISL
KD: 3.79e-09
Kass: 1.81e+05, kdiss: 6.88e-04
2280_ID.4225_H7_SLLQHLHGL
KD: 3.34e-08
Kass: 7.97e+04, kdiss: 2.66e-03
2281_ID.4226_E5_SLLQELIGL
KD: -
Kass:-, , kdiss: 7.89e-02
2282_ID.4227_E7_SLLQHLEGL
KD: 3.24e-09
Kass: 9.84e+04, kdiss: 3.18e-04
2283_ID.4228_W4_SLLWHLIGL
KD: 4.15e-09
Kass: 1.79e+05, kdiss: 7.44e-04
2284_ID.423_M8_SLLQHLIML
KD: 2.06e-07
Kass: 3.04e+04, kdiss: 6.27e-03
2285_ID.4230_D5_SLLQDLIGL
KD: 7.82e-08
Kass: 1.22e+05, kdiss: 9.58e-03
2286_ID.4231_Y6_SLLQHYIGL
KD: -
Kass:-, , kdiss: 5.28e-01
2287_ID.4232_D5_SLLQDLIGL
KD: 6.53e-08
Kass: 1.41e+05, kdiss: 9.24e-03
2288_ID.4233_N7_SLLQHLNGL
KD: -
Kass:-, , kdiss: 4.16e-02
2289_ID.4235_I3_SLIQHLIGL
KD: 4.73e-09
Kass: 1.93e+05, kdiss: 9.14e-04
2290_ID.4239_F6_SLLQHFIGL
KD: 5.56e-09
Kass: 1.91e+05, kdiss: 1.06e-03
2291_ID.424_P4_SLLPHLIGL
KD: 7.70e-09
Kass: 1.79e+05, kdiss: 1.38e-03
2292_ID.4241_D1_DLLQHLIGL
KD: -
Kass:-, , kdiss: 1.40e+00
2293_ID.4242_A5_SLLQALIGL
KD: 8.31e-08
Kass: 1.42e+05, kdiss: 1.18e-02
2294_ID.4244_M4_SLLMHLIGL
KD: -
Kass:-, , kdiss: 4.37e-02
2295_ID.4245_S7_SLLQHLSGL
KD: 1.96e-07
Kass: 7.85e+04, kdiss: 1.54e-02
2296_ID.4247_W6_SLLQHWIGL
KD: 2.07e-08
Kass: 1.45e+05, kdiss: 3.01e-03
2297_ID.4249_G3_SLGQHLIGL
KD: 7.10e-08
Kass: 1.13e+05, kdiss: 8.03e-03
2298_ID.425_W1_WLLQHLIGL
KD: 9.91e-09
Kass: 1.15e+05, kdiss: 1.14e-03
2299_ID.4250_G6_SLLQHGIGL
KD: 5.34e-09
Kass: 2.18e+05, kdiss: 1.16e-03
2300_ID.4253_W7_SLLQHLWGL
KD: 6.71e-09
Kass: 2.04e+05, kdiss: 1.37e-03
value
20000
15000
10000
5000
0
0
250
500
750
1000
Time

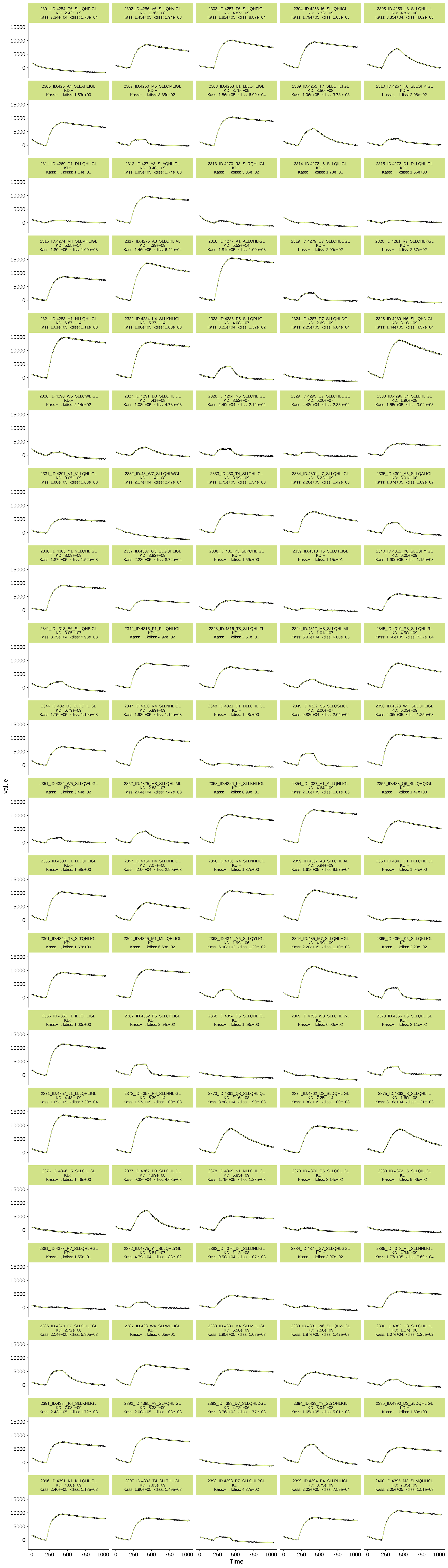

2301_ID.4254_P6_SLLQHPIGL KD: 2.43e−09 Kass: 7.34e+04, kdiss: 1.78e−04
2302_ID.4256_V6_SLLQHVIGL KD: 1.36e−08 Kass: 1.43e+05, kdiss: 1.94e−03
2303_ID.4257_F6_SLLQHFIGL KD: 4.87e−09 Kass: 1.82e+05, kdiss: 8.87e−04
2304_ID.4258_I6_SLLQHIIGL KD: 5.72e−09 Kass: 1.79e+05, kdiss: 1.03e−03
2305_ID.4259_L8_SLLQHLILL KD: 4.81e−08 Kass: 8.35e+04, kdiss: 4.02e−03
2306_ID.426_A4_SLLAHLIGL KD:− Kass:−, , kdiss: 1.53e+00
2307_ID.4260_M5_SLLQMLIGL KD:− Kass:−, , kdiss: 3.85e−02
2308_ID.4263_L1_LLLQHLIGL KD: 3.75e−09 Kass: 1.86e+05, kdiss: 6.99e−04
2309_ID.4265_T7_SLLQHLTGL KD: 3.56e−08 Kass: 1.06e+05, kdiss: 3.78e−03
2310_ID.4267_K6_SLLQHKIGL KD:− Kass:−, , kdiss: 2.08e−02
2311_ID.4269_D1_DLLQHLIGL KD:− Kass:−, , kdiss: 1.14e−01
2312_ID.427_A3_SLAQHLIGL KD: 9.40e−09 Kass: 1.85e+05, kdiss: 1.74e−03
2313_ID.4270_R3_SLRQHLIGL KD:− Kass:−, , kdiss: 3.35e−02
2314_ID.4272_I5_SLLQILIGL KD:− Kass:−, , kdiss: 1.73e−01
2315_ID.4273_D1_DLLQHLIGL KD:− Kass:−, , kdiss: 1.56e+00
2316_ID.4274_M4_SLLMHLIGL KD: 5.55e−14 Kass: 1.80e+05, kdiss: 1.00e−08
2317_ID.4275_A8_SLLQHLIAL KD: 4.39e−09 Kass: 1.46e+05, kdiss: 6.42e−04
2318_ID.4277_A1_ALLQHLIGL KD: 5.52e−14 Kass: 1.81e+05, kdiss: 1.00e−08
2319_ID.4279_Q7_SLLQHLQGL KD:− Kass:−, , kdiss: 2.09e−02
2320_ID.4281_R7_SLLQHLRGL KD:− Kass:−, , kdiss: 2.57e−02
2321_ID.4283_H1_HLLQHLIGL KD: 6.87e−14 Kass: 1.61e+05, kdiss: 1.11e−08
2322_ID.4284_K4_SLLKHLIGL KD: 5.37e−14 Kass: 1.86e+05, kdiss: 1.00e−08
2323_ID.4286_P5_SLLQPLIGL KD: 4.08e−07 Kass: 3.22e+04, kdiss: 1.32e−02
2324_ID.4287_D7_SLLQHLDGL KD: 2.69e−09 Kass: 2.25e+05, kdiss: 6.04e−04
2325_ID.4289_N6_SLLQHNIGL KD: 3.18e−09 Kass: 1.44e+05, kdiss: 4.57e−04
2326_ID.4290_W5_SLLQWLIGL KD:− Kass:−, , kdiss: 2.14e−02
2327_ID.4291_D8_SLLQHLIDL KD: 4.41e−08 Kass: 1.08e+05, kdiss: 4.78e−03
2328_ID.4294_N5_SLLQNLIGL KD: 8.52e−07 Kass: 2.49e+04, kdiss: 2.12e−02
2329_ID.4295_Q7_SLLQHLQGL KD: 5.20e−07 Kass: 4.48e+04, kdiss: 2.33e−02
2330_ID.4296_L4_SLLLHLIGL KD: 1.96e−08 Kass: 1.55e+05, kdiss: 3.04e−03
2331_ID.4297_V1_VLLQHLIGL KD: 9.05e−09 Kass: 1.80e+05, kdiss: 1.63e−03
2332_ID.43_W7_SLLQHLWGL KD: 1.14e−08 Kass: 2.17e+04, kdiss: 2.47e−04
2333_ID.430_T4_SLLTHLIGL KD: 8.99e−09 Kass: 1.72e+05, kdiss: 1.54e−03
2334_ID.4301_L7_SLLQHLLGL KD: 6.22e−09 Kass: 2.28e+05, kdiss: 1.42e−03
2335_ID.4302_A5_SLLQALIGL KD: 8.01e−08 Kass: 1.37e+05, kdiss: 1.09e−02
2336_ID.4303_Y1_YLLQHLIGL KD: 8.09e−09 Kass: 1.87e+05, kdiss: 1.52e−03
2337_ID.4307_G3_SLGQHLIGL KD: 3.82e−09 Kass: 2.28e+05, kdiss: 8.72e−04
2338_ID.431_P3_SLPQHLIGL KD:− Kass:−, , kdiss: 1.59e+00
2339_ID.4310_T5_SLLQTLIGL KD:− Kass:−, , kdiss: 1.15e−01
2340_ID.4311_Y6_SLLQHYIGL KD: 6.05e−09 Kass: 1.90e+05, kdiss: 1.15e−03
2341_ID.4313_E6_SLLQHEIGL KD: 3.05e−07 Kass: 3.25e+04, kdiss: 9.93e−03
2342_ID.4315_F1_FLLQHLIGL KD:− Kass:−, , kdiss: 4.92e−02
2343_ID.4316_T8_SLLQHLITL KD:− Kass:−, , kdiss: 2.61e−01
2344_ID.4317_M8_SLLQHLIML KD: 1.01e−07 Kass: 5.91e+04, kdiss: 6.00e−03
2345_ID.4319_R8_SLLQHLIRL KD: 4.50e−09 Kass: 1.60e+05, kdiss: 7.22e−04
2346_ID.432_D3_SLDQHLIGL KD: 6.79e−09 Kass: 1.75e+05, kdiss: 1.19e−03
2347_ID.4320_N4_SLLNHLIGL KD: 5.89e−09 Kass: 1.93e+05, kdiss: 1.14e−03
2348_ID.4321_D1_DLLQHLIGL KD:− Kass:−, , kdiss: 1.48e+00
2349_ID.4322_S5_SLLQSLIGL KD: 2.06e−07 Kass: 9.88e+04, kdiss: 2.04e−02
2350_ID.4323_WT_SLLQHLIGL KD: 6.03e−09 Kass: 2.06e+05, kdiss: 1.25e−03
2351_ID.4324_W5_SLLQWLIGL KD:− Kass:−, , kdiss: 3.44e−02
2352_ID.4325_M8_SLLQHLIML KD: 2.83e−07 Kass: 2.64e+04, kdiss: 7.47e−03
2353_ID.4326_K4_SLLKHLIGL KD:− Kass:−, , kdiss: 6.99e−01
2354_ID.4327_A1_ALLQHLIGL KD: 4.64e−09 Kass: 2.18e+05, kdiss: 1.01e−03
2355_ID.433_Q6_SLLQHQIGL KD:− Kass:−, , kdiss: 1.47e+00
2356_ID.4333_L1_LLLQHLIGL KD:− Kass:−, , kdiss: 1.58e+00
2357_ID.4334_D4_SLLDHLIGL KD: 7.07e−08 Kass: 4.10e+04, kdiss: 2.90e−03
2358_ID.4336_N4_SLLNHLIGL KD:− Kass:−, , kdiss: 1.37e+00
2359_ID.4337_A8_SLLQHLIAL KD: 5.94e−09 Kass: 1.61e+05, kdiss: 9.57e−04
2360_ID.4341_D1_DLLQHLIGL KD:− Kass:−, , kdiss: 1.04e+00
2361_ID.4344_T3_SLTQHLIGL KD:− Kass:−, , kdiss: 1.57e+00
2362_ID.4345_M1_MLLQHLIGL KD:− Kass:−, , kdiss: 6.68e−02
2363_ID.4346_Y5_SLLQYLIGL KD: 1.99e−06 Kass: 6.98e+03, kdiss: 1.39e−02
2364_ID.435_M7_SLLQHLMGL KD: 4.99e−09 Kass: 2.20e+05, kdiss: 1.10e−03
2365_ID.4350_K5_SLLQKLIGL KD:− Kass:−, , kdiss: 2.20e−02
2366_ID.4351_I1_ILLQHLIGL KD:− Kass:−, , kdiss: 1.60e+00
2367_ID.4352_F5_SLLQFLIGL KD:− Kass:−, , kdiss: 2.54e−02
2368_ID.4354_D5_SLLQDLIGL KD:− Kass:−, , kdiss: 1.58e−03
2369_ID.4355_W8_SLLQHLIWL KD:− Kass:−, , kdiss: 6.00e−02
2370_ID.4356_L5_SLLQLLIGL KD:− Kass:−, , kdiss: 3.11e−02
2371_ID.4357_L1_LLLQHLIGL KD: 4.43e−09 Kass: 1.65e+05, kdiss: 7.30e−04
2372_ID.4358_H4_SLLHHLIGL KD: 6.39e−14 Kass: 1.57e+05, kdiss: 1.00e−08
2373_ID.4361_Q8_SLLQHLIQL KD: 2.16e−08 Kass: 8.80e+04, kdiss: 1.90e−03
2374_ID.4362_D3_SLDQHLIGL KD: 7.25e−14 Kass: 1.38e+05, kdiss: 1.00e−08
2375_ID.4363_I8_SLLQHLIIL KD: 1.60e−08 Kass: 8.18e+04, kdiss: 1.31e−03
2376_ID.4366_I5_SLLQILIGL KD:− Kass:−, , kdiss: 1.46e+00
2377_ID.4367_D8_SLLQHLIDL KD: 4.99e−08 Kass: 9.38e+04, kdiss: 4.68e−03
2378_ID.4369_N1_NLLQHLIGL KD: 6.85e−09 Kass: 1.79e+05, kdiss: 1.23e−03
2379_ID.4370_G5_SLLQGLIGL KD:− Kass:−, , kdiss: 3.14e−02
2380_ID.4372_I5_SLLQILIGL KD:− Kass:−, , kdiss: 9.06e−02
2381_ID.4373_R7_SLLQHLRGL KD:− Kass:−, , kdiss: 1.55e−01
2382_ID.4375_Y7_SLLQHLYGL KD: 3.81e−07 Kass: 4.79e+04, kdiss: 1.83e−02
2383_ID.4376_D4_SLLDHLIGL KD: 1.12e−08 Kass: 9.58e+04, kdiss: 1.07e−03
2384_ID.4377_G7_SLLQHLGGL KD:− Kass:−, , kdiss: 3.97e−02
2385_ID.4378_H4_SLLHHLIGL KD: 4.34e−09 Kass: 1.77e+05, kdiss: 7.69e−04
2386_ID.4379_F7_SLLQHLFGL KD: 2.72e−08 Kass: 2.14e+05, kdiss: 5.80e−03
2387_ID.438_W4_SLLWHLIGL KD:− Kass:−, , kdiss: 6.65e−01
2388_ID.4380_M4_SLLMHLIGL KD: 5.56e−09 Kass: 1.95e+05, kdiss: 1.08e−03
2389_ID.4381_W6_SLLQHWIGL KD: 7.58e−09 Kass: 1.87e+05, kdiss: 1.42e−03
2390_ID.4383_H8_SLLQHLIHL KD: 1.17e−06 Kass: 1.07e+04, kdiss: 1.25e−02
2391_ID.4384_K4_SLLKHLIGL KD: 7.08e−09 Kass: 2.43e+05, kdiss: 1.72e−03
2392_ID.4385_A3_SLAQHLIGL KD: 5.38e−09 Kass: 2.00e+05, kdiss: 1.08e−03
2393_ID.4389_D7_SLLQHLDGL KD: 4.72e−06 Kass: 3.76e+02, kdiss: 1.77e−03
2394_ID.439_Y3_SLYQHLIGL KD: 3.04e−08 Kass: 1.65e+05, kdiss: 5.01e−03
2395_ID.4390_D3_SLDQHLIGL KD:− Kass:−, , kdiss: 1.53e+00
2396_ID.4391_K1_KLLQHLIGL KD: 4.80e−09 Kass: 2.46e+05, kdiss: 1.18e−03
2397_ID.4392_T4_SLLTHLIGL KD: 7.83e−09 Kass: 1.90e+05, kdiss: 1.49e−03
2398_ID.4393_P7_SLLQHLPGL KD:− Kass:−, , kdiss: 4.37e−02
2399_ID.4394_P4_SLLPHLIGL KD: 3.75e−09 Kass: 2.02e+05, kdiss: 7.59e−04
2400_ID.4395_M3_SLMQHLIGL KD: 7.35e−09 Kass: 2.05e+05, kdiss: 1.51e−03
value
15000
10000
5000
0
0 250 500 750 1000
Time

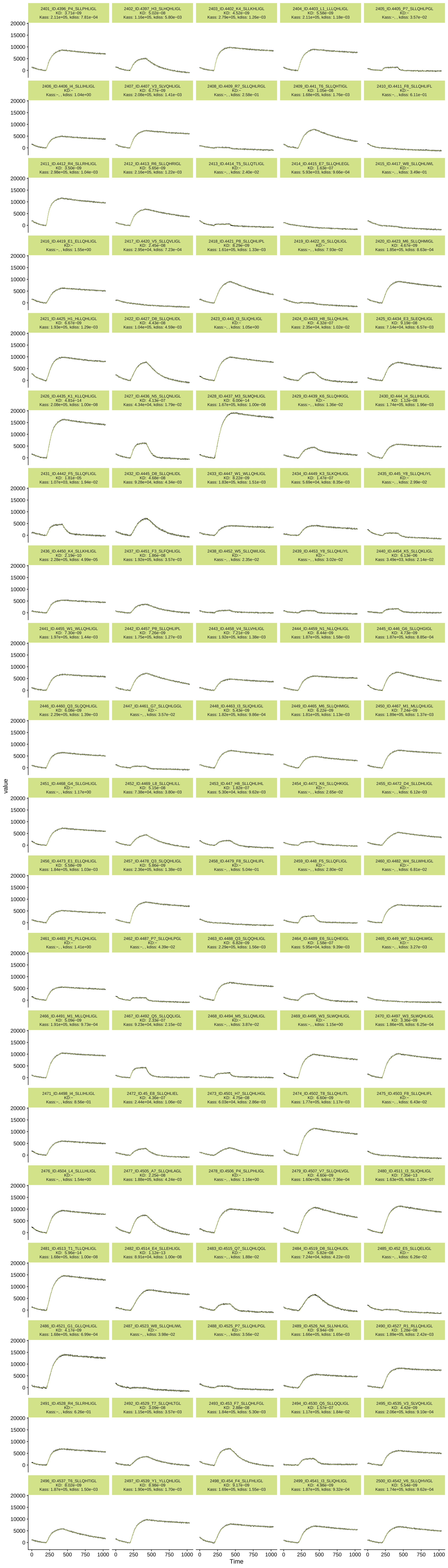
2401_ID.4396_P4_SLLPHLIGL
KD: 3.71e−09
Kass: 2.11e+05, kdiss: 7.81e−04
2402_ID.4397_H3_SLHQHLIGL
KD: 5.02e−08
Kass: 1.16e+05, kdiss: 5.80e−03
2403_ID.4402_K4_SLLKHLIGL
KD: 4.52e−09
Kass: 2.79e+05, kdiss: 1.26e−03
2404_ID.4403_L1_LLLQHLIGL
KD: 5.58e−09
Kass: 2.11e+05, kdiss: 1.18e−03
2405_ID.4405_P7_SLLQHLPGL
KD:−
Kass:−, , kdiss: 3.57e−02
2406_ID.4406_I4_SLLIHLIGL
KD:−
Kass:−, , kdiss: 1.04e+00
2407_ID.4407_V3_SLVQHLIGL
KD: 6.77e−09
Kass: 2.08e+05, kdiss: 1.41e−03
2408_ID.4409_R7_SLLQHLRGL
KD:−
Kass:−, , kdiss: 2.58e−01
2409_ID.441_T6_SLLQHTIGL
KD: 1.05e−08
Kass: 1.68e+05, kdiss: 1.76e−03
2410_ID.4411_F8_SLLQHLIFL
KD:−
Kass:−, , kdiss: 6.11e−01
2411_ID.4412_R4_SLLRHLIGL
KD: 3.50e−09
Kass: 2.98e+05, kdiss: 1.04e−03
2412_ID.4413_R6_SLLQHRIGL
KD: 5.65e−09
Kass: 2.16e+05, kdiss: 1.22e−03
2413_ID.4414_T5_SLLQTLIGL
KD:−
Kass:−, , kdiss: 2.40e−02
2414_ID.4415_E7_SLLQHLEGL
KD: 1.63e−07
Kass: 5.93e+03, kdiss: 9.66e−04
2415_ID.4417_W8_SLLQHLIWL
KD:−
Kass:−, , kdiss: 3.49e−01
2416_ID.4419_E1_ELLQHLIGL
KD:−
Kass:−, , kdiss: 1.55e+00
2417_ID.4420_V5_SLLQVLIGL
KD: 2.45e−08
Kass: 2.95e+04, kdiss: 7.23e−04
2418_ID.4421_P8_SLLQHLIPL
KD: 8.29e−09
Kass: 1.61e+05, kdiss: 1.33e−03
2419_ID.4422_I5_SLLQILIGL
KD:−
Kass:−, , kdiss: 7.93e−02
2420_ID.4423_M6_SLLQHMIGL
KD: 4.67e−09
Kass: 1.85e+05, kdiss: 8.63e−04
2421_ID.4425_H1_HLLQHLIGL
KD: 6.67e−09
Kass: 1.93e+05, kdiss: 1.29e−03
2422_ID.4427_D8_SLLQHLIDL
KD: 4.43e−08
Kass: 1.04e+05, kdiss: 4.59e−03
2423_ID.443_I3_SLIQHLIGL
KD:−
Kass:−, , kdiss: 1.05e+00
2424_ID.4433_H8_SLLQHLIHL
KD: 4.32e−07
Kass: 2.35e+04, kdiss: 1.02e−02
2425_ID.4434_E3_SLEQHLIGL
KD: 9.19e−08
Kass: 7.14e+04, kdiss: 6.57e−03
2426_ID.4435_K1_KLLQHLIGL
KD: 4.81e−14
Kass: 2.08e+05, kdiss: 1.00e−08
2427_ID.4436_N5_SLLQNLIGL
KD: 4.13e−07
Kass: 4.34e+04, kdiss: 1.79e−02
2428_ID.4437_M3_SLMQHLIGL
KD: 6.00e−14
Kass: 1.67e+05, kdiss: 1.00e−08
2429_ID.4439_K6_SLLQHKIGL
KD:−
Kass:−, , kdiss: 1.36e−02
2430_ID.444_I4_SLLIHLIGL
KD: 1.12e−08
Kass: 1.74e+05, kdiss: 1.96e−03
2431_ID.4442_F5_SLLQFLIGL
KD: 1.81e−05
Kass: 1.07e+03, kdiss: 1.94e−02
2432_ID.4445_D8_SLLQHLIDL
KD: 4.68e−08
Kass: 9.28e+04, kdiss: 4.34e−03
2433_ID.4447_W1_WLLQHLIGL
KD: 8.22e−09
Kass: 1.83e+05, kdiss: 1.51e−03
2434_ID.4449_K3_SLKQHLIGL
KD: 1.47e−07
Kass: 5.69e+04, kdiss: 8.35e−03
2435_ID.445_Y8_SLLQHLIYL
KD:−
Kass:−, , kdiss: 2.99e−02
2436_ID.4450_K4_SLLKHLIGL
KD: 2.19e−10
Kass: 2.28e+05, kdiss: 4.99e−05
2437_ID.4451_F3_SLFQHLIGL
KD: 1.86e−08
Kass: 1.92e+05, kdiss: 3.57e−03
2438_ID.4452_W5_SLLQWLIGL
KD:−
Kass:−, , kdiss: 2.35e−02
2439_ID.4453_Y8_SLLQHLIYL
KD:−
Kass:−, , kdiss: 3.02e−02
2440_ID.4454_K5_SLLQKLIGL
KD: 6.13e−06
Kass: 3.49e+03, kdiss: 2.14e−02
2441_ID.4455_W1_WLLQHLIGL
KD: 7.30e−09
Kass: 1.97e+05, kdiss: 1.44e−03
2442_ID.4457_P8_SLLQHLIPL
KD: 7.26e−09
Kass: 1.75e+05, kdiss: 1.27e−03
2443_ID.4458_V4_SLLVHLIGL
KD: 7.21e−09
Kass: 1.92e+05, kdiss: 1.38e−03
2444_ID.4459_N1_NLLQHLIGL
KD: 8.44e−09
Kass: 1.87e+05, kdiss: 1.58e−03
2445_ID.446_G6_SLLQHGIGL
KD: 4.73e−09
Kass: 1.87e+05, kdiss: 8.85e−04
2446_ID.4460_Q3_SLQQHLIGL
KD: 6.08e−09
Kass: 2.29e+05, kdiss: 1.39e−03
2447_ID.4461_G7_SLLQHLGGL
KD:−
Kass:−, , kdiss: 3.57e−02
2448_ID.4463_I3_SLIQHLIGL
KD: 5.43e−09
Kass: 1.82e+05, kdiss: 9.86e−04
2449_ID.4465_M6_SLLQHMIGL
KD: 6.22e−09
Kass: 1.81e+05, kdiss: 1.13e−03
2450_ID.4467_M1_MLLQHLIGL
KD: 7.24e−09
Kass: 1.89e+05, kdiss: 1.37e−03
2451_ID.4468_G4_SLLGHLIGL
KD:−
Kass:−, , kdiss: 1.17e+00
2452_ID.4469_L8_SLLQHLILL
KD: 5.15e−08
Kass: 7.38e+04, kdiss: 3.80e−03
2453_ID.447_H8_SLLQHLIHL
KD: 1.82e−07
Kass: 5.30e+04, kdiss: 9.62e−03
2454_ID.4471_K6_SLLQHKIGL
KD:−
Kass:−, , kdiss: 2.65e−02
2455_ID.4472_D4_SLLDHLIGL
KD:−
Kass:−, , kdiss: 6.12e−03
2456_ID.4473_E1_ELLQHLIGL
KD: 5.58e−09
Kass: 1.84e+05, kdiss: 1.03e−03
2457_ID.4478_Q3_SLQQHLIGL
KD: 5.86e−09
Kass: 2.36e+05, kdiss: 1.38e−03
2458_ID.4479_F8_SLLQHLIFL
KD:−
Kass:−, , kdiss: 5.04e−01
2459_ID.448_F5_SLLQFLIGL
KD:−
Kass:−, , kdiss: 2.80e−02
2460_ID.4482_W4_SLLWHLIGL
KD:−
Kass:−, , kdiss: 6.81e−02
2461_ID.4483_P1_PLLQHLIGL
KD:−
Kass:−, , kdiss: 1.41e+00
2462_ID.4487_P7_SLLQHLPGL
KD:−
Kass:−, , kdiss: 4.39e−02
2463_ID.4488_Q3_SLQQHLIGL
KD: 6.82e−09
Kass: 2.29e+05, kdiss: 1.56e−03
2464_ID.4489_E6_SLLQHEIGL
KD: 1.58e−07
Kass: 5.95e+04, kdiss: 9.39e−03
2465_ID.449_W7_SLLQHLWGL
KD:−
Kass:−, , kdiss: 3.27e−03
2466_ID.4491_M1_MLLQHLIGL
KD: 5.09e−09
Kass: 1.91e+05, kdiss: 9.73e−04
2467_ID.4492_Q5_SLLQQLIGL
KD: 2.33e−07
Kass: 9.23e+04, kdiss: 2.15e−02
2468_ID.4494_M5_SLLQMLIGL
KD:−
Kass:−, , kdiss: 3.87e−02
2469_ID.4495_W3_SLWQHLIGL
KD:−
Kass:−, , kdiss: 1.15e+00
2470_ID.4497_W3_SLWQHLIGL
KD: 3.36e−09
Kass: 1.86e+05, kdiss: 6.25e−04
2471_ID.4498_I4_SLLIHLIGL
KD:−
Kass:−, , kdiss: 8.56e−01
2472_ID.45_E8_SLLQHLIEL
KD: 4.36e−07
Kass: 2.44e+04, kdiss: 1.06e−02
2473_ID.4501_H7_SLLQHLHGL
KD: 4.75e−08
Kass: 6.03e+04, kdiss: 2.86e−03
2474_ID.4502_T8_SLLQHLITL
KD: 6.60e−09
Kass: 1.77e+05, kdiss: 1.17e−03
2475_ID.4503_F8_SLLQHLIFL
KD:−
Kass:−, , kdiss: 6.43e−02
2476_ID.4504_L4_SLLLHLIGL
KD:−
Kass:−, , kdiss: 1.54e+00
2477_ID.4505_A7_SLLQHLAGL
KD: 2.25e−08
Kass: 1.88e+05, kdiss: 4.24e−03
2478_ID.4506_P4_SLLPHLIGL
KD:−
Kass:−, , kdiss: 1.16e+00
2479_ID.4507_V7_SLLQHLVGL
KD: 4.60e−09
Kass: 1.60e+05, kdiss: 7.36e−04
2480_ID.4511_I3_SLIQHLIGL
KD: 7.35e−13
Kass: 1.63e+05, kdiss: 1.20e−07
2481_ID.4513_T1_TLLQHLIGL
KD: 5.96e−14
Kass: 1.68e+05, kdiss: 1.00e−08
2482_ID.4514_E4_SLLEHLIGL
KD: 1.12e−13
Kass: 8.91e+04, kdiss: 1.00e−08
2483_ID.4515_Q7_SLLQHLQGL
KD:−
Kass:−, , kdiss: 1.88e−02
2484_ID.4519_D8_SLLQHLIDL
KD: 5.82e−08
Kass: 7.24e+04, kdiss: 4.22e−03
2485_ID.452_E5_SLLQELIGL
KD:−
Kass:−, , kdiss: 6.26e−02
2486_ID.4521_G1_GLLQHLIGL
KD: 4.17e−09
Kass: 1.68e+05, kdiss: 6.99e−04
2487_ID.4523_W8_SLLQHLIWL
KD:−
Kass:−, , kdiss: 3.98e−02
2488_ID.4525_P7_SLLQHLPGL
KD:−
Kass:−, , kdiss: 3.56e−02
2489_ID.4526_N4_SLLNHLIGL
KD: 9.94e−09
Kass: 1.66e+05, kdiss: 1.65e−03
2490_ID.4527_R1_RLLQHLIGL
KD: 1.28e−08
Kass: 1.89e+05, kdiss: 2.42e−03
2491_ID.4528_R4_SLLRHLIGL
KD:−
Kass:−, , kdiss: 6.26e−01
2492_ID.4529_T7_SLLQHLTGL
KD: 3.09e−08
Kass: 1.15e+05, kdiss: 3.57e−03
2493_ID.453_F7_SLLQHLFGL
KD: 2.88e−08
Kass: 1.84e+05, kdiss: 5.30e−03
2494_ID.4530_Q5_SLLQQLIGL
KD: 1.57e−07
Kass: 1.17e+05, kdiss: 1.84e−02
2495_ID.4535_V3_SLVQHLIGL
KD: 4.42e−09
Kass: 2.06e+05, kdiss: 9.10e−04
2496_ID.4537_T6_SLLQHTIGL
KD: 8.02e−09
Kass: 1.87e+05, kdiss: 1.50e−03
2497_ID.4539_Y1_YLLQHLIGL
KD: 8.98e−09
Kass: 1.90e+05, kdiss: 1.70e−03
2498_ID.454_F4_SLLFHLIGL
KD: 9.17e−09
Kass: 1.69e+05, kdiss: 1.55e−03
2499_ID.4541_I3_SLIQHLIGL
KD: 4.98e−09
Kass: 1.87e+05, kdiss: 9.32e−04
2500_ID.4542_V6_SLLQHVIGL
KD: 5.54e−09
Kass: 1.74e+05, kdiss: 9.62e−04
value
20000
15000
10000
5000
0
0
250
500
750
1000
Time

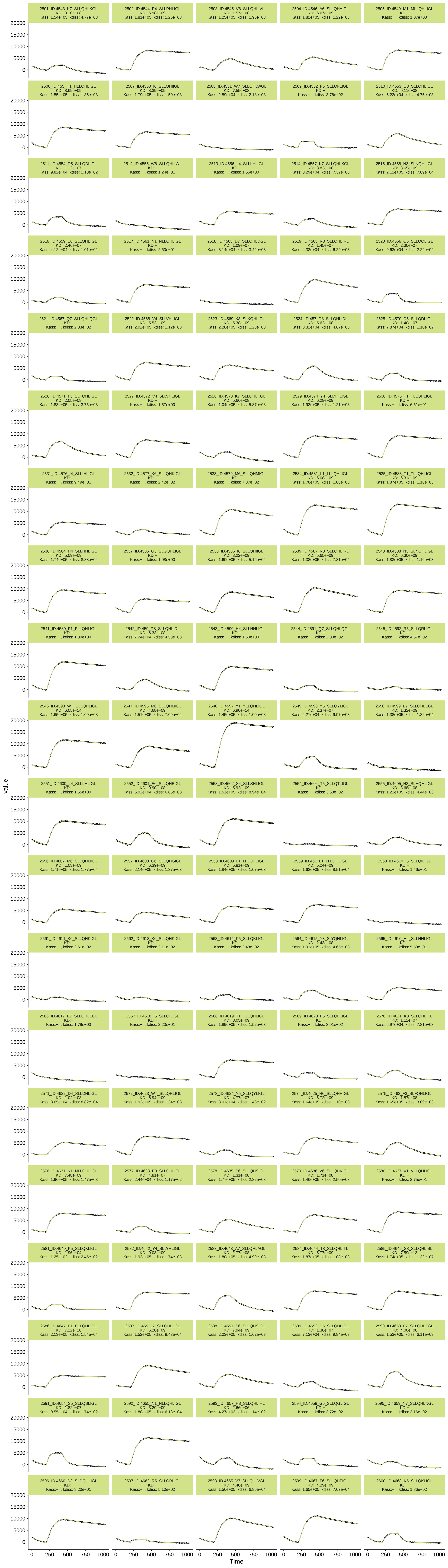
2501_ID.4543_K7_SLLQHLKGL
KD: 3.10e−08
Kass: 1.54e+05, kdiss: 4.77e−03
2502_ID.4544_P4_SLLPHLIGL
KD: 6.98e−09
Kass: 1.81e+05, kdiss: 1.26e−03
2503_ID.4545_V8_SLLQHLIVL
KD: 1.57e−08
Kass: 1.25e+05, kdiss: 1.96e−03
2504_ID.4546_A6_SLLQHAIGL
KD: 6.67e−09
Kass: 1.82e+05, kdiss: 1.22e−03
2505_ID.4549_M1_MLLQHLIGL
KD:−
Kass:−, , kdiss: 1.07e+00
2506_ID.455_H1_HLLQHLIGL
KD: 8.69e−09
Kass: 1.55e+05, kdiss: 1.35e−03
2507_ID.4550_I6_SLLQHIIGL
KD: 8.39e−09
Kass: 1.79e+05, kdiss: 1.50e−03
2508_ID.4551_W7_SLLQHLWGL
KD: 7.55e−08
Kass: 2.89e+04, kdiss: 2.18e−03
2509_ID.4552_F5_SLLQFLIGL
KD:−
Kass:−, , kdiss: 3.76e−02
2510_ID.4553_Q8_SLLQHLIQL
KD: 9.11e−08
Kass: 5.22e+04, kdiss: 4.75e−03
2511_ID.4554_D5_SLLQDLIGL
KD: 1.12e−07
Kass: 9.82e+04, kdiss: 1.10e−02
2512_ID.4555_W8_SLLQHLIWL
KD:−
Kass:−, , kdiss: 1.24e−01
2513_ID.4556_L4_SLLLHLIGL
KD:−
Kass:−, , kdiss: 1.55e+00
2514_ID.4557_K7_SLLQHLKGL
KD: 8.83e−08
Kass: 8.29e+04, kdiss: 7.32e−03
2515_ID.4558_N3_SLNQHLIGL
KD: 3.65e−09
Kass: 2.11e+05, kdiss: 7.69e−04
2516_ID.4559_E6_SLLQHEIGL
KD: 2.46e−07
Kass: 4.12e+04, kdiss: 1.01e−02
2517_ID.4561_N1_NLLQHLIGL
KD:−
Kass:−, , kdiss: 2.60e−01
2518_ID.4563_D7_SLLQHLDGL
KD: 1.09e−07
Kass: 3.14e+04, kdiss: 3.42e−03
2519_ID.4565_R8_SLLQHLIRL
KD: 1.45e−07
Kass: 4.33e+04, kdiss: 6.29e−03
2520_ID.4566_Q5_SLLQQLIGL
KD: 2.30e−07
Kass: 9.63e+04, kdiss: 2.22e−02
2521_ID.4567_Q7_SLLQHLQGL
KD:−
Kass:−, , kdiss: 2.83e−02
2522_ID.4568_V4_SLLVHLIGL
KD: 5.53e−09
Kass: 2.02e+05, kdiss: 1.12e−03
2523_ID.4569_K3_SLKQHLIGL
KD: 5.38e−09
Kass: 2.28e+05, kdiss: 1.23e−03
2524_ID.457_D8_SLLQHLIDL
KD: 5.62e−08
Kass: 8.32e+04, kdiss: 4.67e−03
2525_ID.4570_D5_SLLQDLIGL
KD: 1.40e−07
Kass: 7.87e+04, kdiss: 1.10e−02
2526_ID.4571_F3_SLFQHLIGL
KD: 2.05e−08
Kass: 1.83e+05, kdiss: 3.75e−03
2527_ID.4572_V4_SLLVHLIGL
KD:−
Kass:−, , kdiss: 1.57e+00
2528_ID.4573_K7_SLLQHLKGL
KD: 5.66e−08
Kass: 1.04e+05, kdiss: 5.87e−03
2529_ID.4574_Y4_SLLYHLIGL
KD: 6.28e−09
Kass: 1.92e+05, kdiss: 1.21e−03
2530_ID.4575_T1_TLLQHLIGL
KD:−
Kass:−, , kdiss: 6.51e−01
2531_ID.4576_I4_SLLIHLIGL
KD:−
Kass:−, , kdiss: 9.49e−01
2532_ID.4577_K6_SLLQHKIGL
KD:−
Kass:−, , kdiss: 2.42e−02
2533_ID.4579_M6_SLLQHMIGL
KD:−
Kass:−, , kdiss: 7.87e−02
2534_ID.4581_L1_LLLQHLIGL
KD: 6.08e−09
Kass: 1.78e+05, kdiss: 1.08e−03
2535_ID.4583_T1_TLLQHLIGL
KD: 6.31e−09
Kass: 1.87e+05, kdiss: 1.18e−03
2536_ID.4584_H4_SLLHHLIGL
KD: 5.09e−09
Kass: 1.74e+05, kdiss: 8.88e−04
2537_ID.4585_G3_SLGQHLIGL
KD:−
Kass:−, , kdiss: 1.08e+00
2538_ID.4586_I6_SLLQHIIGL
KD: 3.22e−09
Kass: 1.60e+05, kdiss: 5.16e−04
2539_ID.4587_R8_SLLQHLIRL
KD: 5.65e−09
Kass: 1.38e+05, kdiss: 7.81e−04
2540_ID.4588_N3_SLNQHLIGL
KD: 6.30e−09
Kass: 1.83e+05, kdiss: 1.16e−03
2541_ID.4589_F1_FLLQHLIGL
KD:−
Kass:−, , kdiss: 1.30e+00
2542_ID.459_D8_SLLQHLIDL
KD: 6.33e−08
Kass: 7.24e+04, kdiss: 4.58e−03
2543_ID.4590_H4_SLLHHLIGL
KD:−
Kass:−, , kdiss: 1.60e+00
2544_ID.4591_Q7_SLLQHLQGL
KD:−
Kass:−, , kdiss: 2.00e−02
2545_ID.4592_R5_SLLQRLIGL
KD:−
Kass:−, , kdiss: 4.57e−02
2546_ID.4593_WT_SLLQHLIGL
KD: 6.05e−14
Kass: 1.65e+05, kdiss: 1.00e−08
2547_ID.4595_M6_SLLQHMIGL
KD: 4.68e−09
Kass: 1.51e+05, kdiss: 7.09e−04
2548_ID.4597_Y1_YLLQHLIGL
KD: 6.90e−14
Kass: 1.45e+05, kdiss: 1.00e−08
2549_ID.4598_Y5_SLLQYLIGL
KD: 2.37e−07
Kass: 4.21e+04, kdiss: 9.97e−03
2550_ID.4599_E7_SLLQHLEGL
KD: 1.32e−09
Kass: 1.38e+05, kdiss: 1.82e−04
2551_ID.4600_L4_SLLLHLIGL
KD:−
Kass:−, , kdiss: 1.55e+00
2552_ID.4601_E6_SLLQHEIGL
KD: 9.90e−08
Kass: 6.92e+04, kdiss: 6.85e−03
2553_ID.4602_S4_SLLSHLIGL
KD: 5.92e−09
Kass: 1.51e+05, kdiss: 8.94e−04
2554_ID.4604_T5_SLLQTLIGL
KD:−
Kass:−, , kdiss: 3.68e−02
2555_ID.4605_H3_SLHQHLIGL
KD: 3.68e−08
Kass: 1.21e+05, kdiss: 4.44e−03
2556_ID.4607_M6_SLLQHMIGL
KD: 1.03e−09
Kass: 1.71e+05, kdiss: 1.77e−04
2557_ID.4608_G6_SLLQHGIGL
KD: 6.39e−09
Kass: 2.14e+05, kdiss: 1.37e−03
2558_ID.4609_L1_LLLQHLIGL
KD: 5.81e−09
Kass: 1.84e+05, kdiss: 1.07e−03
2559_ID.461_L1_LLLQHLIGL
KD: 5.24e−09
Kass: 1.62e+05, kdiss: 8.51e−04
2560_ID.4610_I5_SLLQILIGL
KD:−
Kass:−, , kdiss: 1.46e−01
2561_ID.4611_K6_SLLQHKIGL
KD:−
Kass:−, , kdiss: 2.61e−02
2562_ID.4613_K6_SLLQHKIGL
KD:−
Kass:−, , kdiss: 3.11e−02
2563_ID.4614_K5_SLLQKLIGL
KD:−
Kass:−, , kdiss: 2.48e−02
2564_ID.4615_Y3_SLYQHLIGL
KD: 2.43e−08
Kass: 1.91e+05, kdiss: 4.65e−03
2565_ID.4616_H4_SLLHHLIGL
KD:−
Kass:−, , kdiss: 5.58e−01
2566_ID.4617_E7_SLLQHLEGL
KD:−
Kass:−, , kdiss: 1.79e−03
2567_ID.4618_I5_SLLQILIGL
KD:−
Kass:−, , kdiss: 2.23e−01
2568_ID.4619_T1_TLLQHLIGL
KD: 8.05e−09
Kass: 1.89e+05, kdiss: 1.52e−03
2569_ID.4620_F5_SLLQFLIGL
KD:−
Kass:−, , kdiss: 3.01e−02
2570_ID.4621_K8_SLLQHLIKL
KD: 1.12e−07
Kass: 6.97e+04, kdiss: 7.81e−03
2571_ID.4622_D4_SLLDHLIGL
KD: 1.02e−08
Kass: 8.65e+04, kdiss: 8.82e−04
2572_ID.4623_WT_SLLQHLIGL
KD: 6.94e−09
Kass: 1.93e+05, kdiss: 1.34e−03
2573_ID.4624_Y5_SLLQYLIGL
KD: 4.77e−07
Kass: 3.01e+04, kdiss: 1.43e−02
2574_ID.4625_H6_SLLQHHIGL
KD: 6.72e−09
Kass: 1.64e+05, kdiss: 1.10e−03
2575_ID.463_F3_SLFQHLIGL
KD: 1.87e−08
Kass: 1.65e+05, kdiss: 3.09e−03
2576_ID.4631_N1_NLLQHLIGL
KD: 7.48e−09
Kass: 1.96e+05, kdiss: 1.47e−03
2577_ID.4633_E8_SLLQHLIEL
KD: 4.81e−07
Kass: 2.44e+04, kdiss: 1.17e−02
2578_ID.4635_S6_SLLQHSIGL
KD: 1.31e−08
Kass: 1.77e+05, kdiss: 2.32e−03
2579_ID.4636_V6_SLLQHVIGL
KD: 1.71e−08
Kass: 1.46e+05, kdiss: 2.50e−03
2580_ID.4637_V1_VLLQHLIGL
KD:−
Kass:−, , kdiss: 2.75e−01
2581_ID.4640_K5_SLLQKLIGL
KD: 1.96e−04
Kass: 1.25e+02, kdiss: 2.45e−02
2582_ID.4642_Y4_SLLYHLIGL
KD: 9.03e−09
Kass: 1.93e+05, kdiss: 1.74e−03
2583_ID.4643_A7_SLLQHLAGL
KD: 2.77e−08
Kass: 1.80e+05, kdiss: 4.99e−03
2584_ID.4644_T8_SLLQHLITL
KD: 5.77e−09
Kass: 1.87e+05, kdiss: 1.08e−03
2585_ID.4645_S8_SLLQHLISL
KD: 7.59e−13
Kass: 1.74e+05, kdiss: 1.32e−07
2586_ID.4647_P1_PLLQHLIGL
KD: 7.22e−10
Kass: 2.13e+05, kdiss: 1.54e−04
2587_ID.465_L7_SLLQHLLGL
KD: 6.20e−09
Kass: 1.52e+05, kdiss: 9.43e−04
2588_ID.4651_S6_SLLQHSIGL
KD: 7.94e−09
Kass: 2.03e+05, kdiss: 1.62e−03
2589_ID.4652_D5_SLLQDLIGL
KD: 1.38e−07
Kass: 7.13e+04, kdiss: 9.84e−03
2590_ID.4653_F7_SLLQHLFGL
KD: 4.00e−08
Kass: 1.53e+05, kdiss: 6.11e−03
2591_ID.4654_S5_SLLQSLIGL
KD: 1.82e−07
Kass: 9.55e+04, kdiss: 1.74e−02
2592_ID.4655_N1_NLLQHLIGL
KD: 3.29e−09
Kass: 1.88e+05, kdiss: 6.18e−04
2593_ID.4657_H8_SLLQHLIHL
KD: 2.66e−06
Kass: 4.27e+03, kdiss: 1.14e−02
2594_ID.4658_G5_SLLQGLIGL
KD:−
Kass:−, , kdiss: 3.72e−02
2595_ID.4659_N7_SLLQHLNGL
KD:−
Kass:−, , kdiss: 3.16e−02
2596_ID.4660_D3_SLDQHLIGL
KD:−
Kass:−, , kdiss: 8.20e−01
2597_ID.4662_R5_SLLQRLIGL
KD:−
Kass:−, , kdiss: 5.10e−02
2598_ID.4665_V7_SLLQHLVGL
KD: 4.40e−09
Kass: 1.56e+05, kdiss: 6.86e−04
2599_ID.4667_F6_SLLQHFIGL
KD: 4.29e−09
Kass: 1.65e+05, kdiss: 7.07e−04
2600_ID.4668_K5_SLLQKLIGL
KD:−
Kass:−, , kdiss: 1.86e−02
value
20000
15000
10000
5000
0
0
250
500
750
1000
Time

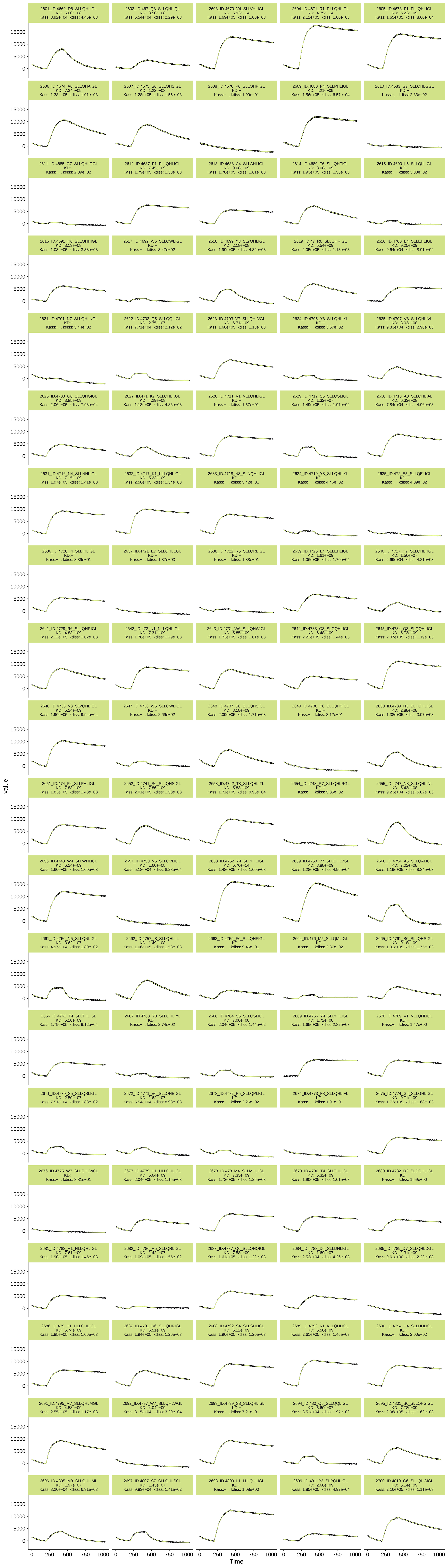

2601_ID.4669_D8_SLLQHLIDL
KD: 5.00e−08
Kass: 8.92e+04, kdiss: 4.46e−03
2602_ID.467_Q8_SLLQHLIQL
KD: 3.50e−08
Kass: 6.54e+04, kdiss: 2.29e−03
2603_ID.4670_V4_SLLVHLIGL
KD: 5.93e−14
Kass: 1.69e+05, kdiss: 1.00e−08
2604_ID.4671_R1_RLLQHLIGL
KD: 4.75e−14
Kass: 2.11e+05, kdiss: 1.00e−08
2605_ID.4673_F1_FLLQHLIGL
KD: 5.22e−09
Kass: 1.65e+05, kdiss: 8.60e−04
2606_ID.4674_A6_SLLQHAIGL
KD: 7.34e−09
Kass: 1.38e+05, kdiss: 1.01e−03
2607_ID.4675_S6_SLLQHSIGL
KD: 1.22e−08
Kass: 1.28e+05, kdiss: 1.55e−03
2608_ID.4676_P6_SLLQHPIGL
KD:−
Kass:−, , kdiss: 1.99e−01
2609_ID.4680_P4_SLLPHLIGL
KD: 4.21e−09
Kass: 1.56e+05, kdiss: 6.57e−04
2610_ID.4683_G7_SLLQHLGGL
KD:−
Kass:−, , kdiss: 2.33e−02
2611_ID.4685_G7_SLLQHLGGL
KD:−
Kass:−, , kdiss: 2.89e−02
2612_ID.4687_F1_FLLQHLIGL
KD: 7.45e−09
Kass: 1.79e+05, kdiss: 1.33e−03
2613_ID.4688_A4_SLLAHLIGL
KD: 9.08e−09
Kass: 1.78e+05, kdiss: 1.61e−03
2614_ID.4689_T6_SLLQHTIGL
KD: 8.08e−09
Kass: 1.93e+05, kdiss: 1.56e−03
2615_ID.4690_L5_SLLQLLIGL
KD:−
Kass:−, , kdiss: 3.88e−02
2616_ID.4691_H6_SLLQHHIGL
KD: 3.13e−08
Kass: 1.08e+05, kdiss: 3.38e−03
2617_ID.4692_W5_SLLQWLIGL
KD:−
Kass:−, , kdiss: 3.47e−02
2618_ID.4699_Y3_SLYQHLIGL
KD: 2.18e−08
Kass: 1.99e+05, kdiss: 4.32e−03
2619_ID.47_R6_SLLQHRIGL
KD: 5.54e−09
Kass: 2.05e+05, kdiss: 1.13e−03
2620_ID.4700_E4_SLLEHLIGL
KD: 9.25e−09
Kass: 9.64e+04, kdiss: 8.91e−04
2621_ID.4701_N7_SLLQHLNGL
KD:−
Kass:−, , kdiss: 5.44e−02
2622_ID.4702_Q5_SLLQQLIGL
KD: 2.75e−07
Kass: 7.71e+04, kdiss: 2.12e−02
2623_ID.4703_V7_SLLQHLVGL
KD: 6.71e−09
Kass: 1.68e+05, kdiss: 1.13e−03
2624_ID.4705_Y8_SLLQHLIYL
KD:−
Kass:−, , kdiss: 3.67e−02
2625_ID.4707_V8_SLLQHLIVL
KD: 3.03e−08
Kass: 9.83e+04, kdiss: 2.98e−03
2626_ID.4708_G6_SLLQHGIGL
KD: 3.85e−09
Kass: 2.06e+05, kdiss: 7.93e−04
2627_ID.471_K7_SLLQHLKGL
KD: 4.29e−08
Kass: 1.13e+05, kdiss: 4.86e−03
2628_ID.4711_V1_VLLQHLIGL
KD:−
Kass:−, , kdiss: 1.57e−01
2629_ID.4712_S5_SLLQSLIGL
KD: 1.32e−07
Kass: 1.49e+05, kdiss: 1.97e−02
2630_ID.4713_A8_SLLQHLIAL
KD: 6.33e−08
Kass: 7.84e+04, kdiss: 4.96e−03
2631_ID.4716_N4_SLLNHLIGL
KD: 7.15e−09
Kass: 1.97e+05, kdiss: 1.41e−03
2632_ID.4717_K1_KLLQHLIGL
KD: 5.23e−09
Kass: 2.56e+05, kdiss: 1.34e−03
2633_ID.4718_N3_SLNQHLIGL
KD:−
Kass:−, , kdiss: 5.42e−01
2634_ID.4719_Y8_SLLQHLIYL
KD:−
Kass:−, , kdiss: 4.46e−02
2635_ID.472_E5_SLLQELIGL
KD:−
Kass:−, , kdiss: 4.09e−02
2636_ID.4720_I4_SLLIHLIGL
KD:−
Kass:−, , kdiss: 8.39e−01
2637_ID.4721_E7_SLLQHLEGL
KD:−
Kass:−, , kdiss: 1.37e−03
2638_ID.4722_R5_SLLQRLIGL
KD:−
Kass:−, , kdiss: 1.88e−01
2639_ID.4726_E4_SLLEHLIGL
KD: 1.61e−09
Kass: 1.06e+05, kdiss: 1.70e−04
2640_ID.4727_H7_SLLQHLHGL
KD: 1.56e−07
Kass: 2.69e+04, kdiss: 4.21e−03
2641_ID.4729_R6_SLLQHRIGL
KD: 4.83e−09
Kass: 2.12e+05, kdiss: 1.02e−03
2642_ID.473_N1_NLLQHLIGL
KD: 7.31e−09
Kass: 1.76e+05, kdiss: 1.29e−03
2643_ID.4731_W6_SLLQHWIGL
KD: 5.85e−09
Kass: 1.73e+05, kdiss: 1.01e−03
2644_ID.4733_G3_SLGQHLIGL
KD: 6.48e−09
Kass: 2.22e+05, kdiss: 1.44e−03
2645_ID.4734_Q3_SLQQHLIGL
KD: 5.73e−09
Kass: 2.07e+05, kdiss: 1.19e−03
2646_ID.4735_V3_SLVQHLIGL
KD: 5.24e−09
Kass: 1.90e+05, kdiss: 9.94e−04
2647_ID.4736_W5_SLLQWLIGL
KD:−
Kass:−, , kdiss: 2.69e−02
2648_ID.4737_S6_SLLQHSIGL
KD: 8.18e−09
Kass: 2.09e+05, kdiss: 1.71e−03
2649_ID.4738_P6_SLLQHPIGL
KD:−
Kass:−, , kdiss: 3.12e−01
2650_ID.4739_H3_SLHQHLIGL
KD: 2.88e−08
Kass: 1.38e+05, kdiss: 3.97e−03
2651_ID.474_F4_SLLFHLIGL
KD: 7.83e−09
Kass: 1.83e+05, kdiss: 1.43e−03
2652_ID.4741_S6_SLLQHSIGL
KD: 7.86e−09
Kass: 2.01e+05, kdiss: 1.58e−03
2653_ID.4742_T8_SLLQHLITL
KD: 5.83e−09
Kass: 1.71e+05, kdiss: 9.95e−04
2654_ID.4743_R7_SLLQHLRGL
KD:−
Kass:−, , kdiss: 5.85e−02
2655_ID.4747_N8_SLLQHLINL
KD: 5.43e−08
Kass: 9.23e+04, kdiss: 5.02e−03
2656_ID.4748_W4_SLLWHLIGL
KD: 6.24e−09
Kass: 1.60e+05, kdiss: 1.00e−03
2657_ID.4750_V5_SLLQVLIGL
KD: 1.60e−08
Kass: 5.18e+04, kdiss: 8.28e−04
2658_ID.4752_Y4_SLLYHLIGL
KD: 6.76e−14
Kass: 1.48e+05, kdiss: 1.00e−08
2659_ID.4753_V7_SLLQHLVGL
KD: 3.88e−09
Kass: 1.28e+05, kdiss: 4.96e−04
2660_ID.4754_A5_SLLQALIGL
KD: 7.02e−08
Kass: 1.19e+05, kdiss: 8.34e−03
2661_ID.4756_N5_SLLQNLIGL
KD: 3.62e−07
Kass: 4.97e+04, kdiss: 1.80e−02
2662_ID.4757_I8_SLLQHLIIL
KD: 1.49e−08
Kass: 1.06e+05, kdiss: 1.58e−03
2663_ID.4759_F6_SLLQHFIGL
KD:−
Kass:−, , kdiss: 9.46e−01
2664_ID.476_M5_SLLQMLIGL
KD:−
Kass:−, , kdiss: 3.87e−02
2665_ID.4761_S6_SLLQHSIGL
KD: 9.18e−09
Kass: 1.91e+05, kdiss: 1.75e−03
2666_ID.4762_T4_SLLTHLIGL
KD: 5.10e−09
Kass: 1.79e+05, kdiss: 9.12e−04
2667_ID.4763_Y8_SLLQHLIYL
KD:−
Kass:−, , kdiss: 2.74e−02
2668_ID.4764_S5_SLLQSLIGL
KD: 7.06e−08
Kass: 2.04e+05, kdiss: 1.44e−02
2669_ID.4766_Y4_SLLYHLIGL
KD: 1.72e−08
Kass: 1.65e+05, kdiss: 2.82e−03
2670_ID.4769_V1_VLLQHLIGL
KD:−
Kass:−, , kdiss: 1.47e+00
2671_ID.4770_S5_SLLQSLIGL
KD: 2.50e−07
Kass: 7.51e+04, kdiss: 1.88e−02
2672_ID.4771_E6_SLLQHEIGL
KD: 1.62e−07
Kass: 5.54e+04, kdiss: 8.98e−03
2673_ID.4772_P5_SLLQPLIGL
KD:−
Kass:−, , kdiss: 2.26e−02
2674_ID.4773_F8_SLLQHLIFL
KD:−
Kass:−, , kdiss: 1.91e−01
2675_ID.4774_G4_SLLGHLIGL
KD: 9.71e−09
Kass: 1.73e+05, kdiss: 1.68e−03
2676_ID.4775_W7_SLLQHLWGL
KD:−
Kass:−, , kdiss: 3.81e−01
2677_ID.4779_H1_HLLQHLIGL
KD: 5.64e−09
Kass: 2.04e+05, kdiss: 1.15e−03
2678_ID.478_M4_SLLMHLIGL
KD: 7.33e−09
Kass: 1.72e+05, kdiss: 1.26e−03
2679_ID.4780_T4_SLLTHLIGL
KD: 5.32e−09
Kass: 1.90e+05, kdiss: 1.01e−03
2680_ID.4782_D3_SLDQHLIGL
KD:−
Kass:−, , kdiss: 1.59e+00
2681_ID.4783_H1_HLLQHLIGL
KD: 7.61e−09
Kass: 1.90e+05, kdiss: 1.45e−03
2682_ID.4786_R5_SLLQRLIGL
KD: 1.42e−07
Kass: 1.09e+05, kdiss: 1.55e−02
2683_ID.4787_Q6_SLLQHQIGL
KD: 7.58e−09
Kass: 1.61e+05, kdiss: 1.22e−03
2684_ID.4788_D4_SLLDHLIGL
KD: 1.69e−07
Kass: 2.52e+04, kdiss: 4.26e−03
2685_ID.4789_D7_SLLQHLDGL
KD: 2.31e−09
Kass: 9.61e+00, kdiss: 2.22e−08
2686_ID.479_H1_HLLQHLIGL
KD: 5.74e−09
Kass: 1.85e+05, kdiss: 1.06e−03
2687_ID.4791_R6_SLLQHRIGL
KD: 6.51e−09
Kass: 1.94e+05, kdiss: 1.26e−03
2688_ID.4792_S4_SLLSHLIGL
KD: 6.12e−09
Kass: 1.96e+05, kdiss: 1.20e−03
2689_ID.4793_K1_KLLQHLIGL
KD: 5.58e−09
Kass: 2.61e+05, kdiss: 1.46e−03
2690_ID.4794_H4_SLLHHLIGL
KD:−
Kass:−, , kdiss: 2.00e−02
2691_ID.4795_M7_SLLQHLMGL
KD: 4.58e−09
Kass: 2.55e+05, kdiss: 1.17e−03
2692_ID.4797_W7_SLLQHLWGL
KD: 4.04e−09
Kass: 8.15e+04, kdiss: 3.29e−04
2693_ID.4799_S8_SLLQHLISL
KD:−
Kass:−, , kdiss: 7.21e−01
2694_ID.480_Q5_SLLQQLIGL
KD: 5.60e−07
Kass: 3.51e+04, kdiss: 1.97e−02
2695_ID.4801_S6_SLLQHSIGL
KD: 7.78e−09
Kass: 2.08e+05, kdiss: 1.62e−03
2696_ID.4805_M8_SLLQHLIML
KD: 1.97e−07
Kass: 3.20e+04, kdiss: 6.31e−03
2697_ID.4807_S7_SLLQHLSGL
KD: 1.43e−07
Kass: 9.83e+04, kdiss: 1.41e−02
2698_ID.4809_L1_LLLQHLIGL
KD:−
Kass:−, , kdiss: 1.08e+00
2699_ID.481_P3_SLPQHLIGL
KD: 2.66e−09
Kass: 1.85e+05, kdiss: 4.92e−04
2700_ID.4810_G6_SLLQHGIGL
KD: 5.14e−09
Kass: 2.16e+05, kdiss: 1.11e−03
value
15000
10000
5000
0
0
250
500
750
1000
Time

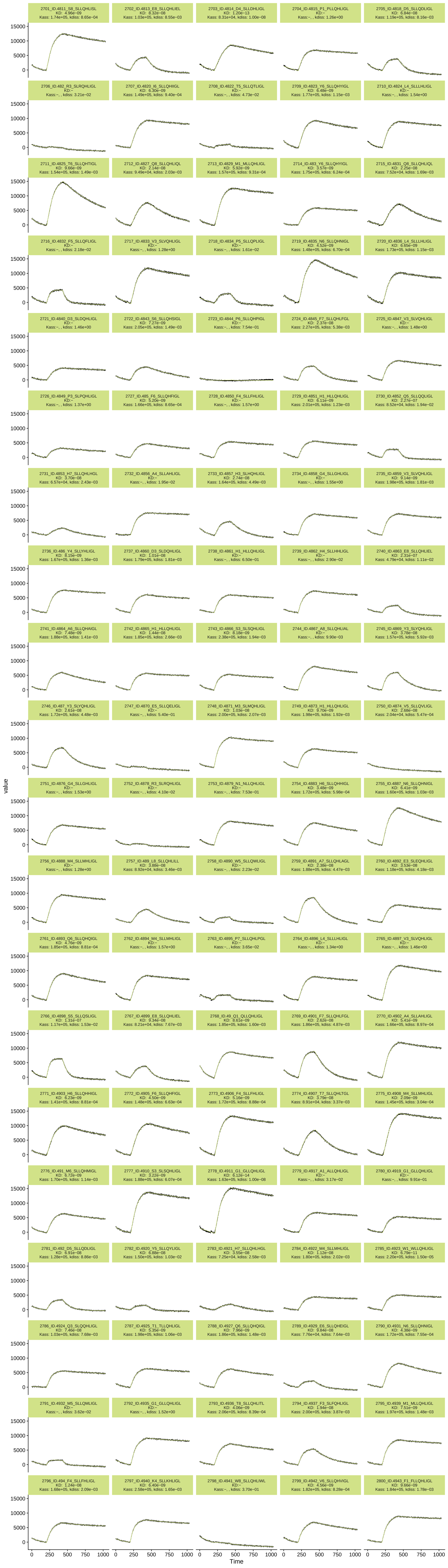

2701_ID.4811_S8_SLLQHLISL
KD: 4.96e−09
Kass: 1.74e+05, kdiss: 8.65e−04
2702_ID.4813_E8_SLLQHLIEL
KD: 8.32e−08
Kass: 1.03e+05, kdiss: 8.55e−03
2703_ID.4814_D4_SLLDHLIGL
KD: 1.20e−13
Kass: 8.31e+04, kdiss: 1.00e−08
2704_ID.4815_P1_PLLQHLIGL
KD:−
Kass:− , kdiss: 1.26e+00
2705_ID.4818_D5_SLLQDLIGL
KD: 6.84e−08
Kass: 1.19e+05, kdiss: 8.16e−03
2706_ID.482_R3_SLRQHLIGL
KD:−
Kass:− , kdiss: 3.21e−02
2707_ID.4820_I6_SLLQHIIGL
KD: 6.30e−09
Kass: 1.49e+05, kdiss: 9.40e−04
2708_ID.4822_T5_SLLQTLIGL
KD:−
Kass:− , kdiss: 4.73e−02
2709_ID.4823_Y6_SLLQHYIGL
KD: 6.48e−09
Kass: 1.77e+05, kdiss: 1.15e−03
2710_ID.4824_L4_SLLLHLIGL
KD:−
Kass:− , kdiss: 1.54e+00
2711_ID.4825_T6_SLLQHTIGL
KD: 9.66e−09
Kass: 1.54e+05, kdiss: 1.49e−03
2712_ID.4827_Q8_SLLQHLIQL
KD: 2.14e−08
Kass: 9.49e+04, kdiss: 2.03e−03
2713_ID.4829_M1_MLLQHLIGL
KD: 5.92e−09
Kass: 1.57e+05, kdiss: 9.31e−04
2714_ID.483_Y6_SLLQHYIGL
KD: 3.57e−09
Kass: 1.75e+05, kdiss: 6.24e−04
2715_ID.4831_Q8_SLLQHLIQL
KD: 2.25e−08
Kass: 7.52e+04, kdiss: 1.69e−03
2716_ID.4832_F5_SLLQFLIGL
KD:−
Kass:− , kdiss: 2.18e−02
2717_ID.4833_V3_SLVQHLIGL
KD:−
Kass:− , kdiss: 1.28e+00
2718_ID.4834_P5_SLLQPLIGL
KD:−
Kass:− , kdiss: 1.61e−02
2719_ID.4835_N6_SLLQHNIGL
KD: 4.53e−09
Kass: 1.48e+05, kdiss: 6.70e−04
2720_ID.4836_L4_SLLLHLIGL
KD: 6.65e−09
Kass: 1.73e+05, kdiss: 1.15e−03
2721_ID.4840_D3_SLDQHLIGL
KD:−
Kass:− , kdiss: 1.46e+00
2722_ID.4843_S6_SLLQHSIGL
KD: 7.27e−09
Kass: 2.05e+05, kdiss: 1.49e−03
2723_ID.4844_P6_SLLQHPIGL
KD:−
Kass:− , kdiss: 7.54e−01
2724_ID.4845_F7_SLLQHLFGL
KD: 2.37e−08
Kass: 2.27e+05, kdiss: 5.38e−03
2725_ID.4847_V3_SLVQHLIGL
KD:−
Kass:− , kdiss: 1.48e+00
2726_ID.4849_P3_SLPQHLIGL
KD:−
Kass:− , kdiss: 1.37e+00
2727_ID.485_F6_SLLQHFIGL
KD: 5.20e−09
Kass: 1.66e+05, kdiss: 8.65e−04
2728_ID.4850_F4_SLLFHLIGL
KD:−
Kass:− , kdiss: 1.57e+00
2729_ID.4851_H1_HLLQHLIGL
KD: 6.11e−09
Kass: 2.01e+05, kdiss: 1.23e−03
2730_ID.4852_Q5_SLLQQLIGL
KD: 2.27e−07
Kass: 8.52e+04, kdiss: 1.94e−02
2731_ID.4853_H7_SLLQHLHGL
KD: 3.70e−08
Kass: 6.57e+04, kdiss: 2.43e−03
2732_ID.4856_A4_SLLAHLIGL
KD:−
Kass:− , kdiss: 1.95e−02
2733_ID.4857_H3_SLHQHLIGL
KD: 2.74e−08
Kass: 1.64e+05, kdiss: 4.49e−03
2734_ID.4858_G4_SLLGHLIGL
KD:−
Kass:− , kdiss: 1.55e+00
2735_ID.4859_V3_SLVQHLIGL
KD: 9.14e−09
Kass: 1.98e+05, kdiss: 1.81e−03
2736_ID.486_Y4_SLLYHLIGL
KD: 8.15e−09
Kass: 1.67e+05, kdiss: 1.36e−03
2737_ID.4860_D3_SLDQHLIGL
KD: 1.01e−08
Kass: 1.79e+05, kdiss: 1.81e−03
2738_ID.4861_H1_HLLQHLIGL
KD:−
Kass:− , kdiss: 6.50e−01
2739_ID.4862_H4_SLLHHLIGL
KD:−
Kass:− , kdiss: 2.90e−02
2740_ID.4863_E8_SLLQHLIEL
KD: 2.31e−07
Kass: 4.79e+04, kdiss: 1.11e−02
2741_ID.4864_A6_SLLQHAIGL
KD: 7.48e−09
Kass: 1.88e+05, kdiss: 1.41e−03
2742_ID.4865_H1_HLLQHLIGL
KD: 1.44e−08
Kass: 1.85e+05, kdiss: 2.66e−03
2743_ID.4866_S3_SLSQHLIGL
KD: 8.18e−09
Kass: 2.38e+05, kdiss: 1.94e−03
2744_ID.4867_A8_SLLQHLIAL
KD:−
Kass:− , kdiss: 9.90e−03
2745_ID.4869_Y3_SLYQHLIGL
KD: 3.78e−08
Kass: 1.57e+05, kdiss: 5.92e−03
2746_ID.487_Y3_SLYQHLIGL
KD: 2.61e−08
Kass: 1.72e+05, kdiss: 4.48e−03
2747_ID.4870_E5_SLLQELIGL
KD:−
Kass:− , kdiss: 5.40e−01
2748_ID.4871_M3_SLMQHLIGL
KD: 1.03e−08
Kass: 2.00e+05, kdiss: 2.07e−03
2749_ID.4873_H1_HLLQHLIGL
KD: 9.70e−09
Kass: 1.98e+05, kdiss: 1.92e−03
2750_ID.4874_V5_SLLQVLIGL
KD: 2.68e−08
Kass: 2.04e+04, kdiss: 5.47e−04
2751_ID.4876_G4_SLLGHLIGL
KD:−
Kass:− , kdiss: 1.53e+00
2752_ID.4878_R3_SLRQHLIGL
KD:−
Kass:− , kdiss: 4.10e−02
2753_ID.4879_N1_NLLQHLIGL
KD:−
Kass:− , kdiss: 7.53e−01
2754_ID.4883_H6_SLLQHHIGL
KD: 3.48e−09
Kass: 1.72e+05, kdiss: 5.98e−04
2755_ID.4887_N6_SLLQHNIGL
KD: 6.41e−09
Kass: 1.60e+05, kdiss: 1.03e−03
2756_ID.4888_M4_SLLMHLIGL
KD:−
Kass:− , kdiss: 1.28e+00
2757_ID.489_L8_SLLQHLILL
KD: 3.88e−08
Kass: 8.92e+04, kdiss: 3.46e−03
2758_ID.4890_W5_SLLQWLIGL
KD:−
Kass:− , kdiss: 2.23e−02
2759_ID.4891_A7_SLLQHLAGL
KD: 2.38e−08
Kass: 1.88e+05, kdiss: 4.47e−03
2760_ID.4892_E3_SLEQHLIGL
KD: 3.53e−08
Kass: 1.18e+05, kdiss: 4.18e−03
2761_ID.4893_Q6_SLLQHQIGL
KD: 4.76e−09
Kass: 1.85e+05, kdiss: 8.81e−04
2762_ID.4894_M4_SLLMHLIGL
KD:−
Kass:− , kdiss: 1.57e+00
2763_ID.4895_P7_SLLQHLPGL
KD:−
Kass:− , kdiss: 3.65e−02
2764_ID.4896_L4_SLLLHLIGL
KD:−
Kass:− , kdiss: 1.34e+00
2765_ID.4897_V3_SLVQHLIGL
KD:−
Kass:− , kdiss: 1.46e+00
2766_ID.4898_S5_SLLQSLIGL
KD: 1.31e−07
Kass: 1.17e+05, kdiss: 1.53e−02
2767_ID.4899_E8_SLLQHLIEL
KD: 9.34e−08
Kass: 8.21e+04, kdiss: 7.67e−03
2768_ID.49_Q1_QLLQHLIGL
KD: 8.61e−09
Kass: 1.85e+05, kdiss: 1.60e−03
2769_ID.4901_F7_SLLQHLFGL
KD: 2.62e−08
Kass: 1.86e+05, kdiss: 4.87e−03
2770_ID.4902_A4_SLLAHLIGL
KD: 5.41e−09
Kass: 1.66e+05, kdiss: 8.97e−04
2771_ID.4903_H6_SLLQHHIGL
KD: 6.23e−09
Kass: 1.41e+05, kdiss: 8.81e−04
2772_ID.4905_F6_SLLQHFIGL
KD: 4.50e−09
Kass: 1.48e+05, kdiss: 6.63e−04
2773_ID.4906_F4_SLLFHLIGL
KD: 5.16e−09
Kass: 1.72e+05, kdiss: 8.88e−04
2774_ID.4907_T7_SLLQHLTGL
KD: 3.79e−08
Kass: 8.91e+04, kdiss: 3.37e−03
2775_ID.4908_M4_SLLMHLIGL
KD: 2.09e−09
Kass: 1.45e+05, kdiss: 3.04e−04
2776_ID.491_M6_SLLQHMIGL
KD: 6.72e−09
Kass: 1.70e+05, kdiss: 1.14e−03
2777_ID.4910_S3_SLSQHLIGL
KD: 3.22e−09
Kass: 1.88e+05, kdiss: 6.07e−04
2778_ID.4911_G1_GLLQHLIGL
KD: 6.12e−14
Kass: 1.63e+05, kdiss: 1.00e−08
2779_ID.4917_A1_ALLQHLIGL
KD:−
Kass:− , kdiss: 3.17e−02
2780_ID.4919_G1_GLLQHLIGL
KD:−
Kass:− , kdiss: 9.91e−01
2781_ID.492_D5_SLLQDLIGL
KD: 6.91e−08
Kass: 1.28e+05, kdiss: 8.86e−03
2782_ID.4920_Y5_SLLQYLIGL
KD: 6.88e−08
Kass: 1.50e+05, kdiss: 1.03e−02
2783_ID.4921_H7_SLLQHLHGL
KD: 3.55e−08
Kass: 7.25e+04, kdiss: 2.58e−03
2784_ID.4922_M4_SLLMHLIGL
KD: 1.12e−08
Kass: 1.80e+05, kdiss: 2.02e−03
2785_ID.4923_W1_WLLQHLIGL
KD: 6.79e−11
Kass: 2.20e+05, kdiss: 1.50e−05
2786_ID.4924_Q3_SLQQHLIGL
KD: 7.46e−08
Kass: 1.03e+05, kdiss: 7.68e−03
2787_ID.4925_T1_TLLQHLIGL
KD: 5.35e−09
Kass: 1.98e+05, kdiss: 1.06e−03
2788_ID.4927_Q6_SLLQHQIGL
KD: 7.96e−09
Kass: 1.86e+05, kdiss: 1.48e−03
2789_ID.4929_E8_SLLQHLIEL
KD: 9.84e−08
Kass: 7.76e+04, kdiss: 7.64e−03
2790_ID.4931_N6_SLLQHNIGL
KD: 4.38e−09
Kass: 1.72e+05, kdiss: 7.55e−04
2791_ID.4932_M5_SLLQMLIGL
KD:−
Kass:− , kdiss: 3.62e−02
2792_ID.4935_G1_GLLQHLIGL
KD:−
Kass:− , kdiss: 1.52e+00
2793_ID.4936_T8_SLLQHLITL
KD: 4.06e−09
Kass: 2.06e+05, kdiss: 8.39e−04
2794_ID.4937_F3_SLFQHLIGL
KD: 1.94e−08
Kass: 2.00e+05, kdiss: 3.87e−03
2795_ID.4939_M1_MLLQHLIGL
KD: 7.51e−09
Kass: 1.97e+05, kdiss: 1.48e−03
2796_ID.494_F4_SLLFHLIGL
KD: 1.24e−08
Kass: 1.68e+05, kdiss: 2.09e−03
2797_ID.4940_K4_SLLKHLIGL
KD: 6.40e−09
Kass: 2.58e+05, kdiss: 1.65e−03
2798_ID.4941_W8_SLLQHLIWL
KD:−
Kass:− , kdiss: 3.70e−01
2799_ID.4942_V6_SLLQHVIGL
KD: 4.56e−09
Kass: 1.82e+05, kdiss: 8.28e−04
2800_ID.4943_F1_FLLQHLIGL
KD: 9.66e−09
Kass: 1.84e+05, kdiss: 1.78e−03
value
15000
10000
5000
0
Time
0
250
500
750
1000

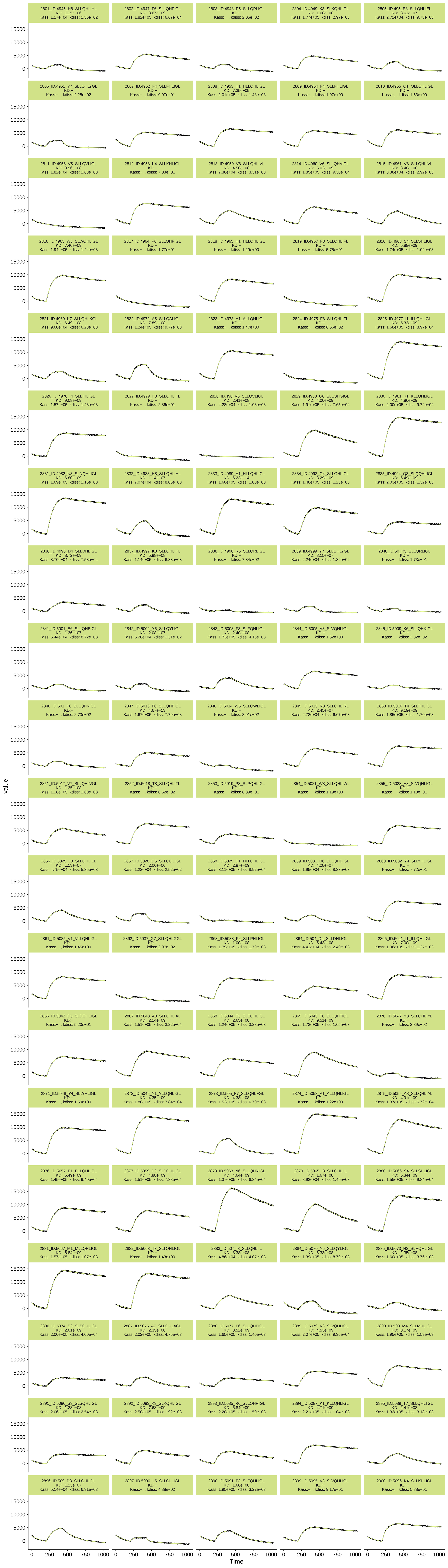

2801_ID.4945_H8_SLLQHLIHL KD: 1.15e−06 Kass: 1.17e+04, kdiss: 1.35e−02
2802_ID.4947_F6_SLLQHFIGL KD: 3.67e−09 Kass: 1.82e+05, kdiss: 6.67e−04
2803_ID.4948_P5_SLLQPLIGL KD:− Kass:−, , kdiss: 2.05e−02
2804_ID.4949_K3_SLKQHLIGL KD: 1.68e−08 Kass: 1.77e+05, kdiss: 2.97e−03
2805_ID.495_E8_SLLQHLIEL KD: 3.61e−07 Kass: 2.71e+04, kdiss: 9.78e−03
2806_ID.4951_Y7_SLLQHLYGL KD:− Kass:−, , kdiss: 2.28e−02
2807_ID.4952_F4_SLLFHLIGL KD:− Kass:−, , kdiss: 9.07e−01
2808_ID.4953_H1_HLLQHLIGL KD: 7.35e−09 Kass: 2.01e+05, kdiss: 1.48e−03
2809_ID.4954_F4_SLLFHLIGL KD:− Kass:−, , kdiss: 1.07e+00
2810_ID.4955_Q1_QLLQHLIGL KD:− Kass:−, , kdiss: 1.53e+00
2811_ID.4956_V5_SLLQVLIGL KD: 8.96e−08 Kass: 1.82e+04, kdiss: 1.63e−03
2812_ID.4958_K4_SLLKHLIGL KD:− Kass:−, , kdiss: 7.03e−01
2813_ID.4959_V8_SLLQHLIVL KD: 4.50e−08 Kass: 7.36e+04, kdiss: 3.31e−03
2814_ID.4960_V6_SLLQHVIGL KD: 5.02e−09 Kass: 1.85e+05, kdiss: 9.30e−04
2815_ID.4961_V8_SLLQHLIVL KD: 3.48e−08 Kass: 8.38e+04, kdiss: 2.92e−03
2816_ID.4963_W3_SLWQHLIGL KD: 7.40e−09 Kass: 1.94e+05, kdiss: 1.44e−03
2817_ID.4964_P6_SLLQHPIGL KD:− Kass:−, , kdiss: 1.77e−01
2818_ID.4965_H1_HLLQHLIGL KD:− Kass:−, , kdiss: 1.29e+00
2819_ID.4967_F8_SLLQHLIFL KD:− Kass:−, , kdiss: 5.75e−01
2820_ID.4968_S4_SLLSHLIGL KD: 5.88e−09 Kass: 1.74e+05, kdiss: 1.02e−03
2821_ID.4969_K7_SLLQHLKGL KD: 6.49e−08 Kass: 9.60e+04, kdiss: 6.23e−03
2822_ID.4972_A5_SLLQALIGL KD: 7.89e−08 Kass: 1.24e+05, kdiss: 9.77e−03
2823_ID.4973_A1_ALLQHLIGL KD:− Kass:−, , kdiss: 1.47e+00
2824_ID.4975_F8_SLLQHLIFL KD:− Kass:−, , kdiss: 6.56e−02
2825_ID.4977_I1_ILLQHLIGL KD: 5.33e−09 Kass: 1.68e+05, kdiss: 8.97e−04
2826_ID.4978_I4_SLLIHLIGL KD: 9.08e−09 Kass: 1.57e+05, kdiss: 1.43e−03
2827_ID.4979_F8_SLLQHLIFL KD:− Kass:−, , kdiss: 2.86e−01
2828_ID.498_V5_SLLQVLIGL KD: 2.41e−08 Kass: 4.28e+04, kdiss: 1.03e−03
2829_ID.4980_G6_SLLQHGIGL KD: 4.00e−09 Kass: 1.91e+05, kdiss: 7.65e−04
2830_ID.4981_K1_KLLQHLIGL KD: 4.86e−09 Kass: 2.00e+05, kdiss: 9.74e−04
2831_ID.4982_N3_SLNQHLIGL KD: 6.80e−09 Kass: 1.69e+05, kdiss: 1.15e−03
2832_ID.4983_H8_SLLQHLIHL KD: 1.14e−07 Kass: 7.07e+04, kdiss: 8.06e−03
2833_ID.4989_H1_HLLQHLIGL KD: 6.23e−14 Kass: 1.60e+05, kdiss: 1.00e−08
2834_ID.4992_G4_SLLGHLIGL KD: 8.29e−09 Kass: 1.48e+05, kdiss: 1.23e−03
2835_ID.4994_Q3_SLQQHLIGL KD: 6.49e−09 Kass: 2.03e+05, kdiss: 1.32e−03
2836_ID.4996_D4_SLLDHLIGL KD: 8.72e−09 Kass: 8.70e+04, kdiss: 7.58e−04
2837_ID.4997_K8_SLLQHLIKL KD: 5.98e−08 Kass: 1.14e+05, kdiss: 6.83e−03
2838_ID.4998_R5_SLLQRLIGL KD:− Kass:−, , kdiss: 7.34e−02
2839_ID.4999_Y7_SLLQHLYGL KD: 8.15e−07 Kass: 2.24e+04, kdiss: 1.82e−02
2840_ID.50_R5_SLLQRLIGL KD:− Kass:−, , kdiss: 1.73e−01
2841_ID.5001_E6_SLLQHEIGL KD: 1.36e−07 Kass: 6.44e+04, kdiss: 8.72e−03
2842_ID.5002_Y5_SLLQYLIGL KD: 2.08e−07 Kass: 6.28e+04, kdiss: 1.31e−02
2843_ID.5003_F3_SLFQHLIGL KD: 2.40e−08 Kass: 1.73e+05, kdiss: 4.16e−03
2844_ID.5005_V3_SLVQHLIGL KD:− Kass:−, , kdiss: 1.52e+00
2845_ID.5009_K6_SLLQHKIGL KD:− Kass:−, , kdiss: 2.32e−02
2846_ID.501_K6_SLLQHKIGL KD:− Kass:−, , kdiss: 2.73e−02
2847_ID.5013_F6_SLLQHFIGL KD: 4.67e−13 Kass: 1.67e+05, kdiss: 7.79e−08
2848_ID.5014_W5_SLLQWLIGL KD:− Kass:−, , kdiss: 3.91e−02
2849_ID.5015_R8_SLLQHLIRL KD: 2.45e−07 Kass: 2.72e+04, kdiss: 6.67e−03
2850_ID.5016_T4_SLLTHLIGL KD: 9.19e−09 Kass: 1.85e+05, kdiss: 1.70e−03
2851_ID.5017_V7_SLLQHLVGL KD: 1.35e−08 Kass: 1.18e+05, kdiss: 1.60e−03
2852_ID.5018_T8_SLLQHLITL KD:− Kass:−, , kdiss: 6.62e−02
2853_ID.5019_P3_SLPQHLIGL KD:− Kass:−, , kdiss: 8.89e−01
2854_ID.5021_W8_SLLQHLIWL KD:− Kass:−, , kdiss: 1.19e+00
2855_ID.5023_V3_SLVQHLIGL KD:− Kass:−, , kdiss: 1.13e−01
2856_ID.5025_L8_SLLQHLILL KD: 1.13e−07 Kass: 4.75e+04, kdiss: 5.35e−03
2857_ID.5028_Q5_SLLQQLIGL KD: 2.06e−06 Kass: 1.22e+04, kdiss: 2.52e−02
2858_ID.5029_D1_DLLQHLIGL KD: 2.87e−09 Kass: 3.11e+05, kdiss: 8.92e−04
2859_ID.5031_D6_SLLQHDIGL KD: 4.28e−07 Kass: 1.95e+04, kdiss: 8.33e−03
2860_ID.5032_Y4_SLLYHLIGL KD:− Kass:−, , kdiss: 7.72e−01
2861_ID.5035_V1_VLLQHLIGL KD:− Kass:−, , kdiss: 1.45e+00
2862_ID.5037_G7_SLLQHLGGL KD:− Kass:−, , kdiss: 2.97e−02
2863_ID.5038_P4_SLLPHLIGL KD: 1.00e−08 Kass: 1.79e+05, kdiss: 1.79e−03
2864_ID.504_D4_SLLDHLIGL KD: 5.43e−08 Kass: 4.41e+04, kdiss: 2.40e−03
2865_ID.5041_I1_ILLQHLIGL KD: 7.00e−09 Kass: 1.96e+05, kdiss: 1.37e−03
2866_ID.5042_D3_SLDQHLIGL KD:− Kass:−, , kdiss: 5.20e−01
2867_ID.5043_A8_SLLQHLIAL KD: 2.14e−09 Kass: 1.51e+05, kdiss: 3.22e−04
2868_ID.5044_E3_SLEQHLIGL KD: 2.65e−08 Kass: 1.24e+05, kdiss: 3.28e−03
2869_ID.5045_T6_SLLQHTIGL KD: 9.51e−09 Kass: 1.73e+05, kdiss: 1.65e−03
2870_ID.5047_Y8_SLLQHLIYL KD:− Kass:−, , kdiss: 2.89e−02
2871_ID.5048_Y4_SLLYHLIGL KD:− Kass:−, , kdiss: 1.59e+00
2872_ID.5049_Y1_YLLQHLIGL KD: 4.35e−09 Kass: 1.80e+05, kdiss: 7.84e−04
2873_ID.505_F7_SLLQHLFGL KD: 4.38e−08 Kass: 1.53e+05, kdiss: 6.70e−03
2874_ID.5053_A1_ALLQHLIGL KD:− Kass:−, , kdiss: 1.22e+00
2875_ID.5055_A8_SLLQHLIAL KD: 4.91e−09 Kass: 1.37e+05, kdiss: 6.72e−04
2876_ID.5057_E1_ELLQHLIGL KD: 6.49e−09 Kass: 1.45e+05, kdiss: 9.40e−04
2877_ID.5059_P3_SLPQHLIGL KD: 4.88e−09 Kass: 1.51e+05, kdiss: 7.38e−04
2878_ID.5063_N6_SLLQHNIGL KD: 4.64e−09 Kass: 1.37e+05, kdiss: 6.34e−04
2879_ID.5065_I8_SLLQHLIIL KD: 1.67e−08 Kass: 8.92e+04, kdiss: 1.49e−03
2880_ID.5066_S4_SLLSHLIGL KD: 6.34e−09 Kass: 1.55e+05, kdiss: 9.84e−04
2881_ID.5067_M1_MLLQHLIGL KD: 6.84e−09 Kass: 1.57e+05, kdiss: 1.07e−03
2882_ID.5068_T3_SLTQHLIGL KD:− Kass:−, , kdiss: 1.43e+00
2883_ID.507_I8_SLLQHLIIL KD: 8.38e−08 Kass: 4.86e+04, kdiss: 4.07e−03
2884_ID.5070_Y5_SLLQYLIGL KD: 6.33e−08 Kass: 1.39e+05, kdiss: 8.79e−03
2885_ID.5073_H3_SLHQHLIGL KD: 2.35e−08 Kass: 1.60e+05, kdiss: 3.76e−03
2886_ID.5074_S3_SLSQHLIGL KD: 2.01e−09 Kass: 2.00e+05, kdiss: 4.00e−04
2887_ID.5075_A7_SLLQHLAGL KD: 2.35e−08 Kass: 2.02e+05, kdiss: 4.75e−03
2888_ID.5077_F6_SLLQHFIGL KD: 8.52e−09 Kass: 1.65e+05, kdiss: 1.40e−03
2889_ID.5079_V3_SLVQHLIGL KD: 4.53e−09 Kass: 2.07e+05, kdiss: 9.36e−04
2890_ID.508_M4_SLLMHLIGL KD: 8.17e−09 Kass: 1.95e+05, kdiss: 1.59e−03
2891_ID.5080_S3_SLSQHLIGL KD: 1.23e−08 Kass: 2.06e+05, kdiss: 2.54e−03
2892_ID.5083_K3_SLKQHLIGL KD: 7.68e−09 Kass: 2.50e+05, kdiss: 1.92e−03
2893_ID.5085_R6_SLLQHRIGL KD: 6.84e−09 Kass: 2.20e+05, kdiss: 1.50e−03
2894_ID.5087_K1_KLLQHLIGL KD: 4.71e−09 Kass: 2.21e+05, kdiss: 1.04e−03
2895_ID.5089_T7_SLLQHLTGL KD: 2.41e−08 Kass: 1.32e+05, kdiss: 3.18e−03
2896_ID.509_D8_SLLQHLIDL KD: 1.23e−07 Kass: 5.14e+04, kdiss: 6.31e−03
2897_ID.5090_L5_SLLQLLIGL KD:− Kass:−, , kdiss: 4.88e−02
2898_ID.5091_F3_SLFQHLIGL KD: 1.66e−08 Kass: 1.95e+05, kdiss: 3.22e−03
2899_ID.5095_V3_SLVQHLIGL KD:− Kass:−, , kdiss: 9.17e−01
2900_ID.5096_K4_SLLKHLIGL KD:− Kass:−, , kdiss: 5.88e−01
value
15000
10000
5000
0
0
250
500
750
1000
Time

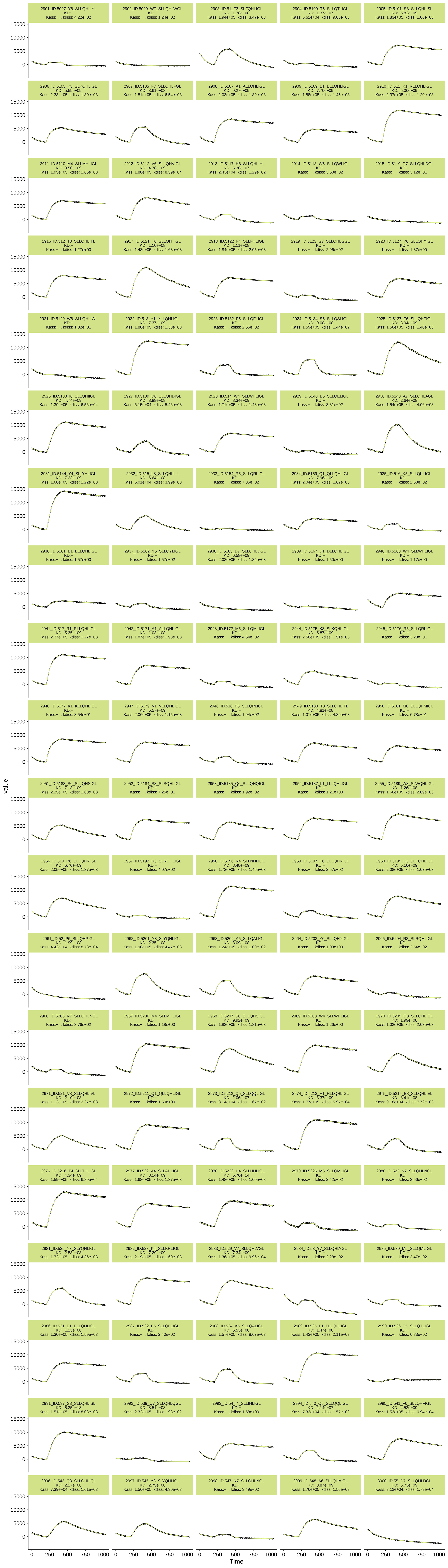
value
Time
0
5000
10000
15000
0
250
500
750
1000

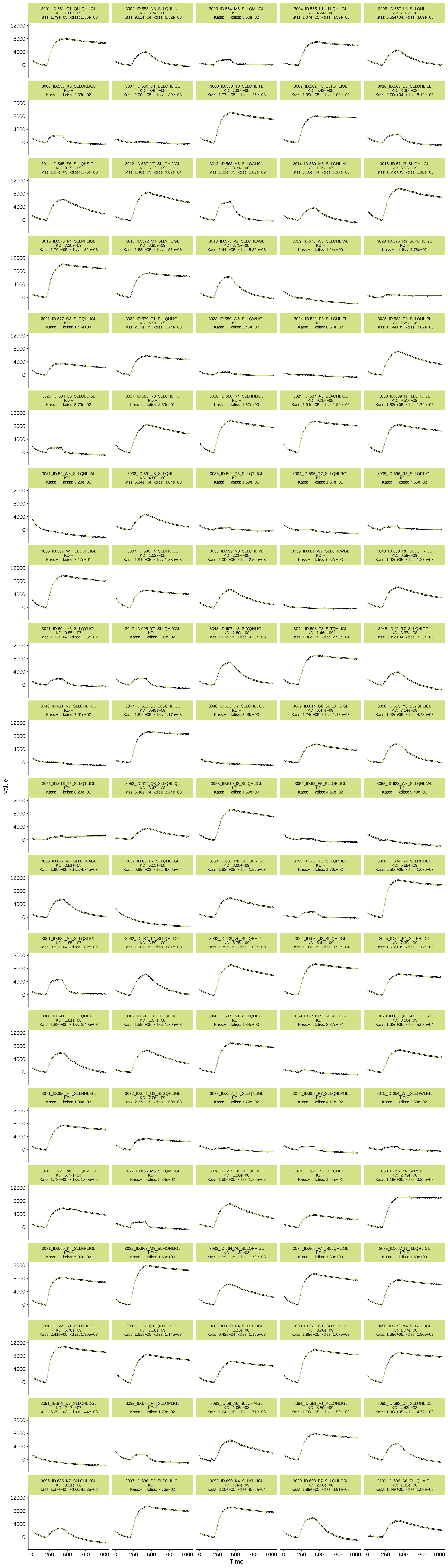

3001_ID.551_Q1_QLLQHLIGL KD: 7.60e−09 Kass: 1.79e+05, kdiss: 1.36e−03
3002_ID.553_N8_SLLQHLINL KD: 5.74e−08 Kass: 9.62e+04, kdiss: 5.52e−03
3003_ID.554_M5_SLLQMLIGL KD:− Kass:−, kdiss: 3.50e−02
3004_ID.555_L1_LLLQHLIGL KD: 4.24e−08 Kass: 1.07e+05, kdiss: 4.52e−03
3005_ID.557_L8_SLLQHLILL KD: 7.32e−08 Kass: 5.59e+04, kdiss: 4.09e−03
3006_ID.558_K5_SLLQKLIGL KD:− Kass:−, kdiss: 2.33e−02
3007_ID.559_D1_DLLQHLIGL KD: 9.46e−09 Kass: 2.00e+05, kdiss: 1.89e−03
3008_ID.560_T8_SLLQHLITL KD: 7.09e−09 Kass: 1.77e+05, kdiss: 1.26e−03
3009_ID.562_T3_SLTQHLIGL KD: 5.43e−09 Kass: 1.99e+05, kdiss: 1.08e−03
3010_ID.563_E8_SLLQHLIEL KD: 8.36e−08 Kass: 9.70e+04, kdiss: 8.12e−03
3011_ID.565_S6_SLLQHSIGL KD: 9.35e−09 Kass: 1.87e+05, kdiss: 1.75e−03
3012_ID.567_V7_SLLQHLVGL KD: 6.22e−09 Kass: 1.46e+05, kdiss: 9.07e−04
3013_ID.568_A5_SLLQALIGL KD: 8.31e−08 Kass: 1.31e+05, kdiss: 1.09e−02
3014_ID.569_M8_SLLQHLIML KD: 1.95e−07 Kass: 3.16e+04, kdiss: 6.17e−03
3015_ID.57_I3_SLIQHLIGL KD: 6.52e−09 Kass: 1.69e+05, kdiss: 1.10e−03
3016_ID.570_P4_SLLPHLIGL KD: 7.38e−09 Kass: 1.79e+05, kdiss: 1.32e−03
3017_ID.572_V4_SLLVHLIGL KD: 9.00e−09 Kass: 1.68e+05, kdiss: 1.51e−03
3018_ID.573_A7_SLLQHLAGL KD: 3.73e−08 Kass: 1.44e+05, kdiss: 5.36e−03
3019_ID.575_W8_SLLQHLIWL KD:− Kass:−, kdiss: 1.24e+00
3020_ID.576_R3_SLRQHLIGL KD:− Kass:−, kdiss: 4.78e−02
3021_ID.577_G3_SLGQHLIGL KD:− Kass:−, kdiss: 1.46e+00
3022_ID.579_P1_PLLQHLIGL KD: 5.91e−09 Kass: 2.11e+05, kdiss: 1.24e−03
3023_ID.580_W5_SLLQWLIGL KD:− Kass:−, kdiss: 3.45e−02
3024_ID.581_F8_SLLQHLIFL KD:− Kass:−, kdiss: 6.67e−02
3025_ID.583_P8_SLLQHLIPL KD: 2.29e−08 Kass: 1.14e+05, kdiss: 2.62e−03
3026_ID.584_L5_SLLQLLIGL KD:− Kass:−, kdiss: 5.79e−02
3027_ID.585_R8_SLLQHLIRL KD:− Kass:−, kdiss: 8.89e−01
3028_ID.586_N4_SLLNHLIGL KD:− Kass:−, kdiss: 1.57e+00
3029_ID.587_A3_SLAQHLIGL KD: 9.25e−09 Kass: 1.94e+05, kdiss: 1.80e−03
3030_ID.589_I1_ILLQHLIGL KD: 9.51e−09 Kass: 1.83e+05, kdiss: 1.74e−03
3031_ID.59_W8_SLLQHLIWL KD:− Kass:−, kdiss: 5.28e−01
3032_ID.591_I8_SLLQHLIIL KD: 4.80e−08 Kass: 6.34e+04, kdiss: 3.04e−03
3033_ID.592_T5_SLLQTLIGL KD:− Kass:−, kdiss: 1.59e−01
3034_ID.595_R7_SLLQHLRGL KD:− Kass:−, kdiss: 1.57e−01
3035_ID.596_R5_SLLQRLIGL KD:− Kass:−, kdiss: 7.55e−02
3036_ID.597_W7_SLLQHLWGL KD:− Kass:−, kdiss: 7.17e−01
3037_ID.598_I4_SLLIHLIGL KD: 1.02e−08 Kass: 1.84e+05, kdiss: 1.88e−03
3038_ID.599_V8_SLLQHLIVL KD: 2.28e−08 Kass: 1.09e+05, kdiss: 2.50e−03
3039_ID.601_W7_SLLQHLWGL KD:− Kass:−, kdiss: 6.57e−03
3040_ID.603_R6_SLLQHRIGL KD: 6.59e−09 Kass: 1.93e+05, kdiss: 1.27e−03
3041_ID.604_Y5_SLLQYLIGL KD: 9.85e−07 Kass: 1.37e+04, kdiss: 1.35e−02
3042_ID.605_Y7_SLLQHLYGL KD:− Kass:−, kdiss: 2.05e−02
3043_ID.607_Y3_SLYQHLIGL KD: 2.80e−08 Kass: 1.61e+05, kdiss: 4.50e−03
3044_ID.608_T3_SLTQHLIGL KD: 1.46e−09 Kass: 1.98e+05, kdiss: 2.90e−04
3045_ID.61_T7_SLLQHLTGL KD: 3.67e−08 Kass: 9.05e+04, kdiss: 3.33e−03
3046_ID.611_R7_SLLQHLRGL KD:− Kass:−, kdiss: 7.61e−02
3047_ID.612_S3_SLSQHLIGL KD: 6.48e−09 Kass: 1.81e+05, kdiss: 1.17e−03
3048_ID.613_D7_SLLQHLDGL KD:− Kass:−, kdiss: 2.06e−03
3049_ID.614_G6_SLLQHGIGL KD: 6.47e−09 Kass: 1.74e+05, kdiss: 1.13e−03
3050_ID.615_Y3_SLYQHLIGL KD: 3.14e−08 Kass: 1.42e+05, kdiss: 4.48e−03
3051_ID.616_T5_SLLQTLIGL KD:− Kass:−, kdiss: 8.28e−01
3052_ID.617_Q8_SLLQHLIQL KD: 3.47e−08 Kass: 6.46e+04, kdiss: 2.24e−03
3053_ID.619_I3_SLIQHLIGL KD:− Kass:−, kdiss: 1.59e+00
3054_ID.62_E5_SLLQELIGL KD:− Kass:−, kdiss: 4.31e−02
3055_ID.623_W8_SLLQHLIWL KD:− Kass:−, kdiss: 5.43e−01
3056_ID.627_A7_SLLQHLAGL KD: 2.81e−08 Kass: 1.69e+05, kdiss: 4.74e−03
3057_ID.63_E7_SLLQHLEGL KD: 6.15e−08 Kass: 9.85e+03, kdiss: 6.06e−04
3058_ID.631_R6_SLLQHRIGL KD: 8.08e−09 Kass: 1.88e+05, kdiss: 1.52e−03
3059_ID.632_P5_SLLQPLIGL KD:− Kass:−, kdiss: 1.70e−02
3060_ID.634_R4_SLLRHLIGL KD: 6.68e−09 Kass: 2.50e+05, kdiss: 1.67e−03
3061_ID.636_S5_SLLQSLIGL KD: 1.85e−07 Kass: 9.83e+04, kdiss: 1.82e−02
3062_ID.637_T7_SLLQHLTGL KD: 3.60e−08 Kass: 1.06e+05, kdiss: 3.81e−03
3063_ID.638_V6_SLLQHVIGL KD: 5.70e−09 Kass: 1.75e+05, kdiss: 1.00e−03
3064_ID.639_I3_SLIQHLIGL KD: 5.39e−09 Kass: 1.76e+05, kdiss: 9.50e−04
3065_ID.64_F4_SLLFHLIGL KD: 7.69e−09 Kass: 1.52e+05, kdiss: 1.17e−03
3066_ID.641_F3_SLFQHLIGL KD: 1.81e−08 Kass: 1.88e+05, kdiss: 3.40e−03
3067_ID.643_T6_SLLQHTIGL KD: 1.07e−08 Kass: 1.59e+05, kdiss: 1.70e−03
3068_ID.647_W1_WLLQHLIGL KD:− Kass:−, kdiss: 1.34e+00
3069_ID.648_R3_SLRQHLIGL KD:− Kass:−, kdiss: 2.87e−02
3070_ID.65_Q6_SLLQHQIGL KD: 3.50e−09 Kass: 1.62e+05, kdiss: 5.68e−04
3071_ID.650_H4_SLLHHLIGL KD:− Kass:−, kdiss: 1.84e−02
3072_ID.651_G3_SLGQHLIGL KD: 7.07e−09 Kass: 2.27e+05, kdiss: 1.60e−03
3073_ID.652_T5_SLLQTLIGL KD:− Kass:−, kdiss: 1.72e−02
3074_ID.653_P7_SLLQHLPGL KD:− Kass:−, kdiss: 4.47e−02
3075_ID.654_W5_SLLQWLIGL KD:− Kass:−, kdiss: 3.60e−02
3076_ID.655_W6_SLLQHWIGL KD: 5.77e−14 Kass: 1.73e+05, kdiss: 1.00e−08
3077_ID.656_M5_SLLQMLIGL KD:− Kass:−, kdiss: 5.84e−02
3078_ID.657_T6_SLLQHTIGL KD: 1.16e−08 Kass: 1.55e+05, kdiss: 1.80e−03
3079_ID.659_P3_SLPQHLIGL KD:− Kass:−, kdiss: 1.44e−01
3080_ID.66_Y4_SLLYHLIGL KD: 2.73e−08 Kass: 1.19e+05, kdiss: 3.25e−03
3081_ID.660_K4_SLLKHLIGL KD:− Kass:−, kdiss: 5.95e−02
3082_ID.663_M3_SLMQHLIGL KD:− Kass:−, kdiss: 1.59e+00
3083_ID.664_A6_SLLQHAIGL KD: 1.13e−08 Kass: 1.59e+05, kdiss: 1.79e−03
3084_ID.665_W7_SLLQHLWGL KD:− Kass:−, kdiss: 1.30e+00
3085_ID.667_I1_ILLQHLIGL KD:− Kass:−, kdiss: 1.50e+00
3086_ID.669_R1_RLLQHLIGL KD: 5.78e−09 Kass: 2.41e+05, kdiss: 1.39e−03
3087_ID.67_Q1_QLLQHLIGL KD: 7.10e−09 Kass: 1.61e+05, kdiss: 1.14e−03
3088_ID.670_E4_SLLEHLIGL KD: 1.23e−08 Kass: 9.42e+04, kdiss: 1.16e−03
3089_ID.671_G1_GLLQHLIGL KD: 8.90e−09 Kass: 1.88e+05, kdiss: 1.67e−03
3090_ID.672_A4_SLLAHLIGL KD: 1.07e−08 Kass: 1.69e+05, kdiss: 1.80e−03
3091_ID.673_D7_SLLQHLDGL KD: 2.17e−07 Kass: 6.65e+03, kdiss: 1.44e−03
3092_ID.676_P5_SLLQPLIGL KD:− Kass:−, kdiss: 1.73e−02
3093_ID.68_A6_SLLQHAIGL KD: 1.05e−08 Kass: 1.64e+05, kdiss: 1.71e−03
3094_ID.681_A1_ALLQHLIGL KD: 8.50e−09 Kass: 1.78e+05, kdiss: 1.52e−03
3095_ID.683_D8_SLLQHLIDL KD: 4.42e−08 Kass: 1.08e+05, kdiss: 4.77e−03
3096_ID.685_K7_SLLQHLKGL KD: 3.31e−08 Kass: 1.37e+05, kdiss: 4.52e−03
3097_ID.686_S3_SLSQHLIGL KD:− Kass:−, kdiss: 7.76e−02
3098_ID.690_K4_SLLKHLIGL KD: 4.44e−09 Kass: 2.20e+05, kdiss: 9.75e−04
3099_ID.693_F7_SLLQHLFGL KD: 2.60e−08 Kass: 1.89e+05, kdiss: 4.91e−03
3100_ID.696_A6_SLLQHAIGL KD: 1.32e−08 Kass: 1.44e+05, kdiss: 1.89e−03
value
0 4000 8000 12000
0 250 500 750 1000
Time

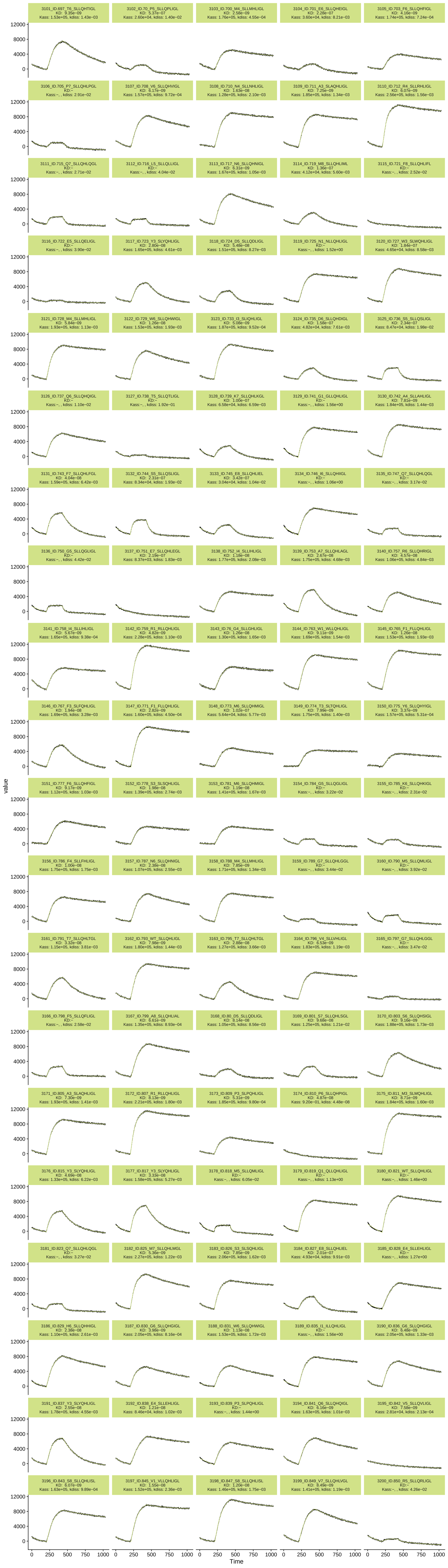
value
12000
8000
4000
0
3101_ID.697_T6_SLLQHTIGL
KD: 9.35e−09
Kass: 1.53e+05, kdiss: 1.43e−03
3102_ID.70_P5_SLLQPLIGL
KD: 5.37e−07
Kass: 2.60e+04, kdiss: 1.40e−02
3103_ID.700_M4_SLLMHLIGL
KD: 2.58e−09
Kass: 1.76e+05, kdiss: 4.55e−04
3104_ID.701_E6_SLLQHEIGL
KD: 2.28e−07
Kass: 3.60e+04, kdiss: 8.21e−03
3105_ID.703_F6_SLLQHFIGL
KD: 4.16e−09
Kass: 1.74e+05, kdiss: 7.24e−04
3106_ID.705_P7_SLLQHLPGL
KD:−
Kass:−, , kdiss: 2.91e−02
3107_ID.708_V6_SLLQHVIGL
KD: 6.17e−09
Kass: 1.57e+05, kdiss: 9.72e−04
3108_ID.710_N4_SLLNHLIGL
KD: 1.63e−08
Kass: 1.28e+05, kdiss: 2.10e−03
3109_ID.711_A3_SLAQHLIGL
KD: 7.25e−09
Kass: 1.85e+05, kdiss: 1.34e−03
3110_ID.712_R4_SLLRHLIGL
KD: 6.07e−09
Kass: 2.56e+05, kdiss: 1.56e−03
3111_ID.715_Q7_SLLQHLQGL
KD:−
Kass:−, , kdiss: 2.71e−02
3112_ID.716_L5_SLLQLLIGL
KD:−
Kass:−, , kdiss: 4.04e−02
3113_ID.717_N6_SLLQHNIGL
KD: 6.31e−09
Kass: 1.67e+05, kdiss: 1.05e−03
3114_ID.719_M8_SLLQHLIML
KD: 1.36e−07
Kass: 4.12e+04, kdiss: 5.60e−03
3115_ID.721_F8_SLLQHLIFL
KD:−
Kass:−, , kdiss: 2.52e−02
3116_ID.722_E5_SLLQELIGL
KD:−
Kass:−, , kdiss: 3.90e−02
3117_ID.723_Y3_SLYQHLIGL
KD: 2.80e−08
Kass: 1.65e+05, kdiss: 4.61e−03
3118_ID.724_D5_SLLQDLIGL
KD: 5.48e−08
Kass: 1.51e+05, kdiss: 8.27e−03
3119_ID.725_N1_NLLQHLIGL
KD:−
Kass:−, , kdiss: 1.52e+00
3120_ID.727_W3_SLWQHLIGL
KD: 1.84e−07
Kass: 4.65e+04, kdiss: 8.58e−03
3121_ID.728_M4_SLLMHLIGL
KD: 5.84e−09
Kass: 1.93e+05, kdiss: 1.13e−03
3122_ID.729_W6_SLLQHWIGL
KD: 1.26e−08
Kass: 1.53e+05, kdiss: 1.93e−03
3123_ID.733_I3_SLIQHLIGL
KD: 5.08e−09
Kass: 1.87e+05, kdiss: 9.52e−04
3124_ID.735_D6_SLLQHDIGL
KD: 1.58e−07
Kass: 4.82e+04, kdiss: 7.61e−03
3125_ID.736_S5_SLLQSLIGL
KD: 2.34e−07
Kass: 8.47e+04, kdiss: 1.98e−02
3126_ID.737_Q6_SLLQHQIGL
KD:−
Kass:−, , kdiss: 1.10e−02
3127_ID.738_T5_SLLQTLIGL
KD:−
Kass:−, , kdiss: 1.92e−01
3128_ID.739_K7_SLLQHLKGL
KD: 1.00e−07
Kass: 6.58e+04, kdiss: 6.59e−03
3129_ID.741_G1_GLLQHLIGL
KD:−
Kass:−, , kdiss: 1.56e+00
3130_ID.742_A4_SLLAHLIGL
KD: 7.81e−09
Kass: 1.84e+05, kdiss: 1.44e−03
3131_ID.743_F7_SLLQHLFGL
KD: 4.04e−08
Kass: 1.59e+05, kdiss: 6.42e−03
3132_ID.744_S5_SLLQSLIGL
KD: 2.31e−07
Kass: 8.34e+04, kdiss: 1.93e−02
3133_ID.745_E8_SLLQHLIEL
KD: 3.42e−07
Kass: 3.04e+04, kdiss: 1.04e−02
3134_ID.746_I6_SLLQHIIGL
KD:−
Kass:−, , kdiss: 1.06e+00
3135_ID.747_Q7_SLLQHLQGL
KD:−
Kass:−, , kdiss: 3.17e−02
3136_ID.750_G5_SLLQGLIGL
KD:−
Kass:−, , kdiss: 4.42e−02
3137_ID.751_E7_SLLQHLEGL
KD: 2.19e−07
Kass: 8.37e+03, kdiss: 1.83e−03
3138_ID.752_I4_SLLIHLIGL
KD: 1.18e−08
Kass: 1.77e+05, kdiss: 2.08e−03
3139_ID.753_A7_SLLQHLAGL
KD: 2.67e−08
Kass: 1.75e+05, kdiss: 4.68e−03
3140_ID.757_R6_SLLQHRIGL
KD: 4.57e−08
Kass: 1.06e+05, kdiss: 4.84e−03
3141_ID.758_I4_SLLIHLIGL
KD: 5.67e−09
Kass: 1.65e+05, kdiss: 9.38e−04
3142_ID.759_R1_RLLQHLIGL
KD: 4.82e−09
Kass: 2.28e+05, kdiss: 1.10e−03
3143_ID.76_G4_SLLGHLIGL
KD: 1.26e−08
Kass: 1.30e+05, kdiss: 1.65e−03
3144_ID.763_W1_WLLQHLIGL
KD: 9.11e−09
Kass: 1.69e+05, kdiss: 1.54e−03
3145_ID.765_F1_FLLQHLIGL
KD: 1.26e−08
Kass: 1.53e+05, kdiss: 1.93e−03
3146_ID.767_F3_SLFQHLIGL
KD: 1.94e−08
Kass: 1.69e+05, kdiss: 3.28e−03
3147_ID.771_F1_FLLQHLIGL
KD: 2.82e−09
Kass: 1.60e+05, kdiss: 4.50e−04
3148_ID.773_M6_SLLQHMIGL
KD: 1.02e−07
Kass: 5.64e+04, kdiss: 5.77e−03
3149_ID.774_T3_SLTQHLIGL
KD: 7.99e−09
Kass: 1.75e+05, kdiss: 1.40e−03
3150_ID.775_Y6_SLLQHYIGL
KD: 3.37e−09
Kass: 1.57e+05, kdiss: 5.31e−04
3151_ID.777_F6_SLLQHFIGL
KD: 9.17e−09
Kass: 1.12e+05, kdiss: 1.03e−03
3152_ID.778_S3_SLSQHLIGL
KD: 1.98e−08
Kass: 1.39e+05, kdiss: 2.74e−03
3153_ID.781_M6_SLLQHMIGL
KD: 1.19e−08
Kass: 1.41e+05, kdiss: 1.67e−03
3154_ID.784_G5_SLLQGLIGL
KD:−
Kass:−, , kdiss: 3.22e−02
3155_ID.785_K6_SLLQHKIGL
KD:−
Kass:−, , kdiss: 2.31e−02
3156_ID.786_F4_SLLFHLIGL
KD: 1.00e−08
Kass: 1.75e+05, kdiss: 1.75e−03
3157_ID.787_N6_SLLQHNIGL
KD: 2.38e−08
Kass: 1.07e+05, kdiss: 2.55e−03
3158_ID.788_M4_SLLMHLIGL
KD: 7.85e−09
Kass: 1.71e+05, kdiss: 1.34e−03
3159_ID.789_G7_SLLQHLGGL
KD:−
Kass:−, , kdiss: 3.44e−02
3160_ID.790_M5_SLLQMLIGL
KD:−
Kass:−, , kdiss: 3.92e−02
3161_ID.791_T7_SLLQHLTGL
KD: 3.32e−08
Kass: 1.15e+05, kdiss: 3.81e−03
3162_ID.793_WT_SLLQHLIGL
KD: 7.98e−09
Kass: 1.80e+05, kdiss: 1.44e−03
3163_ID.795_T7_SLLQHLTGL
KD: 2.88e−08
Kass: 1.27e+05, kdiss: 3.66e−03
3164_ID.796_V4_SLLVHLIGL
KD: 6.53e−09
Kass: 1.83e+05, kdiss: 1.19e−03
3165_ID.797_G7_SLLQHLGGL
KD:−
Kass:−, , kdiss: 3.47e−02
3166_ID.798_F5_SLLQFLIGL
KD:−
Kass:−, , kdiss: 2.58e−02
3167_ID.799_A8_SLLQHLIAL
KD: 6.61e−09
Kass: 1.35e+05, kdiss: 8.93e−04
3168_ID.80_D5_SLLQDLIGL
KD: 8.14e−08
Kass: 1.05e+05, kdiss: 8.56e−03
3169_ID.801_S7_SLLQHLSGL
KD: 9.68e−08
Kass: 1.25e+05, kdiss: 1.21e−02
3170_ID.803_S6_SLLQHSIGL
KD: 9.16e−09
Kass: 1.88e+05, kdiss: 1.73e−03
3171_ID.805_A3_SLAQHLIGL
KD: 7.30e−09
Kass: 1.93e+05, kdiss: 1.41e−03
3172_ID.807_R1_RLLQHLIGL
KD: 8.13e−09
Kass: 2.21e+05, kdiss: 1.80e−03
3173_ID.809_P3_SLPQHLIGL
KD: 5.31e−09
Kass: 1.85e+05, kdiss: 9.80e−04
3174_ID.810_P6_SLLQHPIGL
KD: 4.87e−08
Kass: 9.20e−01, kdiss: 4.48e−08
3175_ID.811_M3_SLMQHLIGL
KD: 8.71e−09
Kass: 1.84e+05, kdiss: 1.60e−03
3176_ID.815_Y3_SLYQHLIGL
KD: 4.69e−08
Kass: 1.33e+05, kdiss: 6.22e−03
3177_ID.817_Y3_SLYQHLIGL
KD: 3.33e−08
Kass: 1.58e+05, kdiss: 5.27e−03
3178_ID.818_M5_SLLQMLIGL
KD:−
Kass:−, , kdiss: 6.05e−02
3179_ID.819_Q1_QLLQHLIGL
KD:−
Kass:−, , kdiss: 1.13e+00
3180_ID.821_WT_SLLQHLIGL
KD:−
Kass:−, , kdiss: 1.46e+00
3181_ID.823_Q7_SLLQHLQGL
KD:−
Kass:−, , kdiss: 3.27e−02
3182_ID.825_M7_SLLQHLMGL
KD: 5.36e−09
Kass: 2.27e+05, kdiss: 1.22e−03
3183_ID.826_S3_SLSQHLIGL
KD: 7.85e−09
Kass: 2.06e+05, kdiss: 1.62e−03
3184_ID.827_E8_SLLQHLIEL
KD: 2.01e−07
Kass: 4.93e+04, kdiss: 9.91e−03
3185_ID.828_E4_SLLEHLIGL
KD:−
Kass:−, , kdiss: 1.27e+00
3186_ID.829_H6_SLLQHHIGL
KD: 2.38e−08
Kass: 1.10e+05, kdiss: 2.61e−03
3187_ID.830_G6_SLLQHGIGL
KD: 3.98e−09
Kass: 2.05e+05, kdiss: 8.16e−04
3188_ID.831_W6_SLLQHWIGL
KD: 1.13e−08
Kass: 1.53e+05, kdiss: 1.72e−03
3189_ID.835_I1_ILLQHLIGL
KD:−
Kass:−, , kdiss: 1.56e+00
3190_ID.836_G6_SLLQHGIGL
KD: 6.48e−09
Kass: 2.05e+05, kdiss: 1.33e−03
3191_ID.837_Y3_SLYQHLIGL
KD: 2.55e−08
Kass: 1.78e+05, kdiss: 4.55e−03
3192_ID.838_E4_SLLEHLIGL
KD: 1.21e−08
Kass: 8.46e+04, kdiss: 1.02e−03
3193_ID.839_P3_SLPQHLIGL
KD:−
Kass:−, , kdiss: 1.44e+00
3194_ID.841_Q6_SLLQHQIGL
KD: 6.16e−09
Kass: 1.63e+05, kdiss: 1.01e−03
3195_ID.842_V5_SLLQVLIGL
KD: 7.58e−09
Kass: 2.81e+04, kdiss: 2.13e−04
3196_ID.843_S8_SLLQHLISL
KD: 6.07e−09
Kass: 1.63e+05, kdiss: 9.89e−04
3197_ID.845_V1_VLLQHLIGL
KD: 1.55e−08
Kass: 1.52e+05, kdiss: 2.36e−03
3198_ID.847_S8_SLLQHLISL
KD: 1.20e−08
Kass: 1.46e+05, kdiss: 1.75e−03
3199_ID.849_V7_SLLQHLVGL
KD: 8.49e−09
Kass: 1.41e+05, kdiss: 1.19e−03
3200_ID.850_R5_SLLQRLIGL
KD:−
Kass:−, , kdiss: 4.26e−02
0 250 500 750 1000
Time

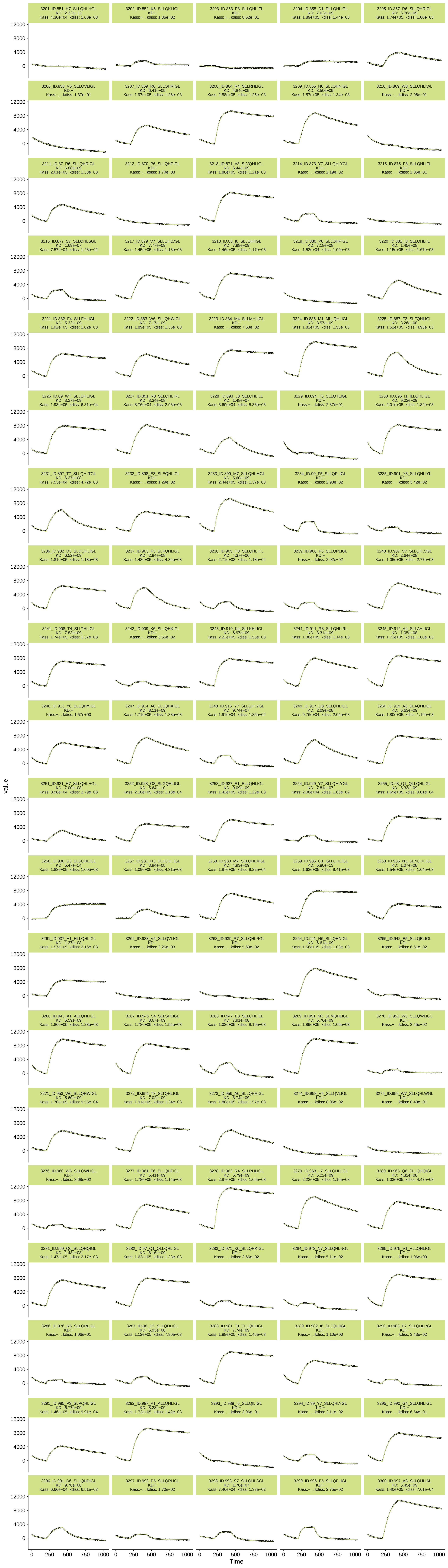

value
Time
0
250
500
750
1000
12000
8000
4000
0

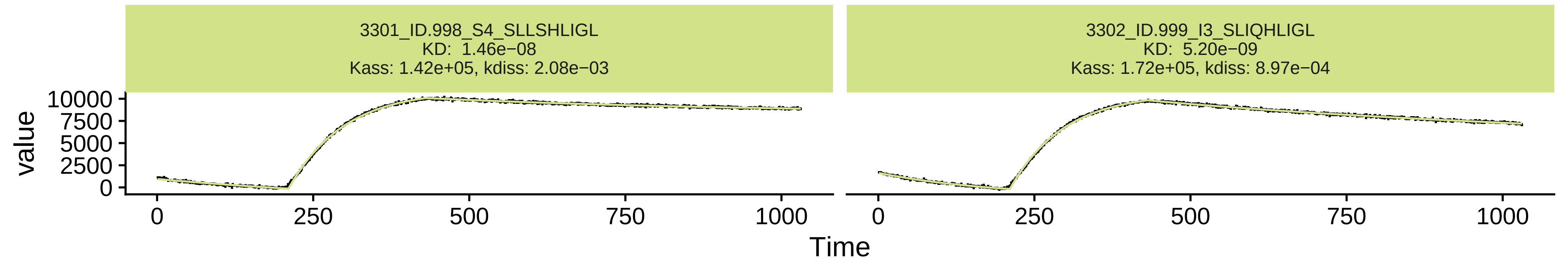
3301_ID.998_S4_SLLSHLIGL
KD: 1.46e−08
Kass: 1.42e+05, kdiss: 2.08e−03
3302_ID.999_I3_SLIQHLIGL
KD: 5.20e−09
Kass: 1.72e+05, kdiss: 8.97e−04
value
10000
7500
5000
2500
0
0
250
500
750
1000
Time
